# Boron-Catalyzed, Diastereo- and Enantioselective Allylation of Ketones with Allenes

**DOI:** 10.1021/acscatal.2c03158

**Published:** 2022-08-22

**Authors:** Kieran Nicholson, Yuxuan Peng, Natalia Llopis, Dominic R. Willcox, Gary S. Nichol, Thomas Langer, Alejandro Baeza, Stephen P. Thomas

**Affiliations:** † EaStCHEM School of Chemistry, The University of Edinburgh, Joseph Black Building, Edinburgh EH9 3FJ, United Kingdom; ‡ Pharmaceutical Technology & Development, Chemical Development U.K., AstraZeneca, Silk Road, Macclesfield SK10 2NA, United Kingdom; § Instituto de Síntesis Orgánica and Dpto. de Química Orgánica, Universidad de Alicante, Apdo. 99, 03080 Alicante, Spain

**Keywords:** Allylation, Boron, Catalysis, Transborylation, Ketone

## Abstract

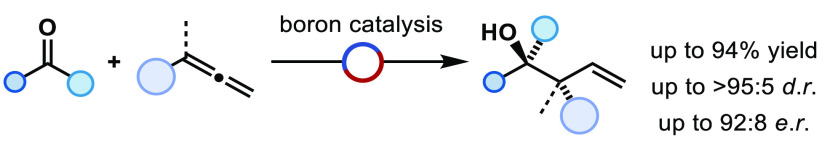

The diastereo- and enantioselective allylation of ketones remains a synthetic challenge, with transition metal catalysis offering the most applied methods. Here, a boron-catalyzed allylation of ketones with allenes is presented. Excellent yield, regioselectivity, and diastereoselectivity were found across functionalized substrates. The reaction was further developed to accommodate an enantioenriched boron catalyst and thus gave asymmetric ketone allylation in good yield, diastereoselectivity, and enantioselectivity. Mechanistic studies supported a hydroboration–allylation–transborylation pathway.

The allylation of ketones provides a general route to tertiary homoallylic alcohols containing contiguous stereocenters which are widely found in biologically active compounds (Figure 1a).^1^ Despite numerous methods, including asymmetric and catalytic variants, for the allylation of aldehydes,^2−7^ the allylation of ketones is far less developed.^8,9^ Even the simplest, stoichiometric, achiral allylations of ketones with allylmetal reagents suffer from poor functional group tolerance.^10^ Allylic borane reagents highlight the increased challenges of ketone allylation compared to the allylation of aldehydes; although an allylic borane will readily react with an aldehyde at −78 °C, stoichiometric allylation of a ketone requires higher temperatures.^11−13^ Ketones often require a chelating group to achieve good diastereoselectivity for stoichiometric allylboration.^14−16^ The stoichiometric diastereo- and enantioselective synthesis of homoallylic alcohols has also been achieved using enantioenriched α-substituted allylic boranes^17^ and allylic boronic esters.^18^

**Figure 1:**
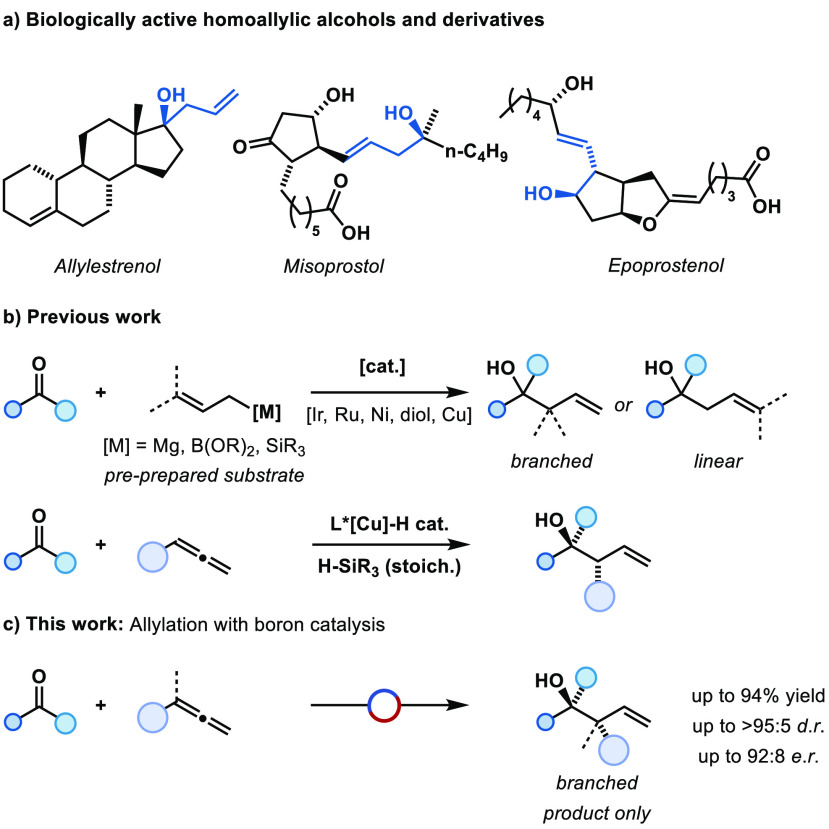
(a) Biologically active homoallylic alcohols and derivatives. (b) Previous examples of catalytic allylation of ketones. (c) This work showing new strategies for the stereoselective allylation of ketones.

The prior preparation of an allylic coupling partner was required for many catalytic allylation reactions.^4,19−30^ Typically these prefunctionalized substrates are prepared by transition metal catalysis or using Grignard reagents.^31^ The only exception being copper-catalyzed examples which use allenes and hydrosilanes as the terminal reductant to give stereoselective ketone allylation (Figure 1b).^32−39^ There are no examples of this reaction using a main-group catalyst or applications to allylboration.

Typically, allylic boranes or allylic silanes will be used as substrates with the catalyst activating these reagents using Lewis acid/base interactions to enhance nucleophilicity. Allylic boranes can be accessed by allene hydroboration,^40−43^ but this process has yet to be reported in a catalytic manner. If this reaction could be rendered catalytic, it would allow the use of commercially available allenes to be used as allylation coupling partners and negate the need for the prior synthesis of the allylic boron reagents. Assuming the catalytic generation of the allylic borane could be sufficiently controlled, it could be coupled to a ketone allylation reaction with B–O transborylation enabling catalytic turnover (Figure 1c). Transborylation offers a redox-neutral turnover strategy tailored to main-group catalysis.

However, several reactivity and stereochemical challenges must be overcome for success: (1) (*E*)/(*Z* ) Isomerizations of the allylic borane by a series of 1,3-boratropic shifts must be controlled. (2) Linear/branched isomerizations of the allylic borane must be suppressed. (3) The rate of hydroboration of the allene, by the catalyst, must exceed that of the ketone (direct ketone reduction). (4) Turnover must occur on oxygen and not carbon (deactivation of the allylic borane).^44^ Furthermore, and unlike the Lewis acid/base catalysis, this method would represent a mechanistically unique allylboration whereby the main-group catalyst is directly bonded to the coupling partner in a manner far more akin to transition metal catalysis. Herein, we report a boron-catalyzed allylation of ketones from allenes.

Investigations began by testing the secondary boranes, 9-borabicyclo[3.3.1]nonane ([H-*B*-9-BBN]_2_), dicyclohexylborane (HBCy_2_), and borane dimethylsulfide (Me_2_S·BH_3_), as catalysts (10 mol %) for the allylation of acetophenone with cyclohexylallene at room temperature in *n*-hexane (0.5 M) (see Supporting Information). [H-*B*-9-BBN]_2_ gave the best results, whereas HBCy_2_ and Me_2_S·BH_3_ gave reduced yields and diastereoselectivity. Increasing the reaction temperature (69 °C) improved the yield (>95%) and diastereoselectivities (>95:5 *d*.*r*.) to give the branched homoallylic alcohol with no observed linear product. Presumably, the higher temperature increased the rate of allylic borane isomerization from (*Z*)-allyllic borane to (*E*)-allyllic borane and thus gave the homoallylic alcohol, (2*SR*,3*RS*)-3-cyclohexyl-2-phenylpent-4-en-2-ol, with improved diastereoselectivity.^45,46^ Using [H-*B*-9-BBN]_2_ as the catalyst, the reaction conditions were optimized (see Supporting Information, Table S1). A range of solvents were screened, with the best results observed using *n*-hexane (>95% yield, > 95:5 *d*.*r.*) or THF (>95% yield, > 95:5 *d.r*.). Increasing the allene stoichiometry reduced the diastereocontrol with no increase in yield (2 equiv. of allene gave 75:25 *d.r.*, 3 equiv. of allene gave 60:40 *d.r.*). Finally, the catalyst loading could be reduced to 5 mol % while maintaining excellent yield and diastereoselectivity (>95% yield, >95:5 *d.r.*).

The optimized conditions were then applied to a diverse substrate scope of allenes and ketones (Table 1). The reaction of acetophenone with cyclohexylallene gave (2*SR*,3*RS*)-3-cyclohexyl-2-phenylpent-4-en-2-ol **3a** in excellent isolated yield and diastereoselectivity (91% yield, >95:5 *d.r.*). Application to other monosubstituted allenes including penta-3,4-dienylbenzene (**3b**, 65% yield, 85:15 *d.r.*), nona-1,2-diene (**3c**, 63% yield, 79:21 *d.r*.), and hexa-5,6-dienylbenzene (**3d**, 49% yield, 74:26 *d.r.*) gave the corresponding homoallylic alcohols in moderate to good yields and good diastereoselectivities. The diastereoselectivity of susbtrates **3b**–**3d** was presumably lower than that of substrate **3a** due to lower steric constraints of the allylic borane. The reaction could be applied to the ester-functionalized allene ethyl 2,3-butadienoate, which gave the functionalized homoallylic alcohol (**3e**, 90%, 65:35 *d.r.*) without ester reduction.^64^ The allylation protocol was applied to 1,1-disubstituted allenes to give homoallylic alcohols with contiguous quaternary centers in good yields (**3f**, 76%, **3g**, 49%, **3h**, 56%). Other ketones, including 1-phenyl-1-propanone, were successfully used as coupling partners including to give homoallylic alcohol **3i** (82%, >95:5 *d.r.*). α-Chloro-substituted (**3j**, 58%, 78:22 *d.r*.) and α-fluoro-substituted (**3k**, 66%, 82:18 *d.r.*) ketones were successfully reacted in good yields, though the diastereoselectivity appeared to be affected by the steric bulk of the α-substituents. Fluoro (**3l**, 65%, >95:5 *d.r*.) and bromo (**3m**, 59%, >95:5 *d.r.*) substituents around the arene of the ketone were tolerated on the arene with good yields and diastereoselectivities obtained. Substrates bearing electron-withdrawing trifluoromethyl (**3n**, 88%, 94:6 *d.r.*) and electron-donating methoxy (**3o**, 50%, >95:5 *d.r.*) groups underwent successful allylation with excellent diastereoselectivities. Methoxy substituents on the *meta*-position (**3p**, 57%, >95:5 *d.r.*) and *ortho*-position (**3q**, 38%, >95:5 *d.r*.) of the arene also gave the corresponding homoallylic alcohols in moderate yield and excellent diastereoselectivity. The allylation protocol was also applied to alkylketones, giving homoallylic alcohols in good yields and diastereoselectivities (**3r**, 70%, **3s**, 76% >95:5 *d.r.*). Additionally, an alkylketone bearing an alkene functionality, sulcatone, a biologically active mosquito attractant, underwent chemoselective allylation to give the homoallylic alcohol in excellent yield and diastereoselectivity (**3t**, 93%, >95:5 *d.r.*) with no observed alkene reduction. The allylation protocol tolerated alkyne functionalities with good yields and moderate diastereoselectivity (**3u**, 66%, 33:67 *d.r.*) and no observed alkyne reduction; curiously, the *syn*-diastereomer was the major product, presumably due to the very low steric parameter of the alkyne adjacent to the ketone.^47^ Conversely, a more sterically congested adamantyl ketone was reacted with excellent diastereoselectivity (**3v**, 86%, >95:5 *d.r.*). A further reducible functionality,^48^ an ester was tolerated in the reaction to give the homoallylic alcohol product in good yield but low diastereoselectivity (**3w**, 80%, 60:40 *d.r*.). Other competent aryl ketone coupling partners included 1-indanone (**3x**, 65%, 66:34 *d.r.*), 2-furyl (**3y**, 87%, >95:5 *d.r.*), and 3-thiophenyl ketones (**3z**, 88%, >95:5 *d.r.*). Methylene dioxy bearing arylketone was reacted to give the homoallylic alcohol in reduced yield but excellent diastereoselectivity (**3aa**, 59%, >95:5 *d.r.*). The reaction of acetylferrocene gave the corresponding homoallylic alcohol in good yields and diastereoselectivity (**3ab**, 66%, >95:5 *d.r.*) with single-crystal X-ray analysis used to confirm the relative steroechemical configuration (Scheme 1a). The reaction could be applied to biologically active molecules including human sex hormone estrone, which was reacted in good yields and excellent diastereoselectivity (**3ac**, 78%, >95:5 *d.r.*) with no observed reduction of the alkene functionality or deleterious side reaction by the acidic aryl alcohol. Nabumetone, an anti-inflammatory medication, underwent successful allylation in good yield and excellent diastereoselectivity (**3ad**, 55%, 94:6 *d.r*.). Pentoxifylline, a drug used to treat peripheral artery disease, underwent chemoselective allylation in excellent yield and diastereoselectivity (**3ae**, 90%, 93:7 *d.r.*), with the xanthene functionality, which is found in numerous bioactive molecules, tolerated. Finally, haloperidol, an antipsychotic found on the WHO list of essential medicines, underwent successful allylation in excellent yield and diastereoselectivity (**3af**, 91%, >95:5 *d.r.*).

**Table 1:**
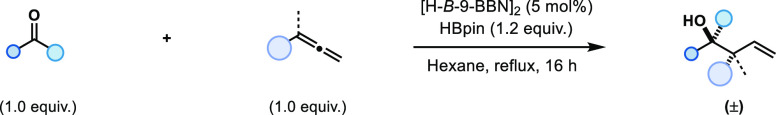

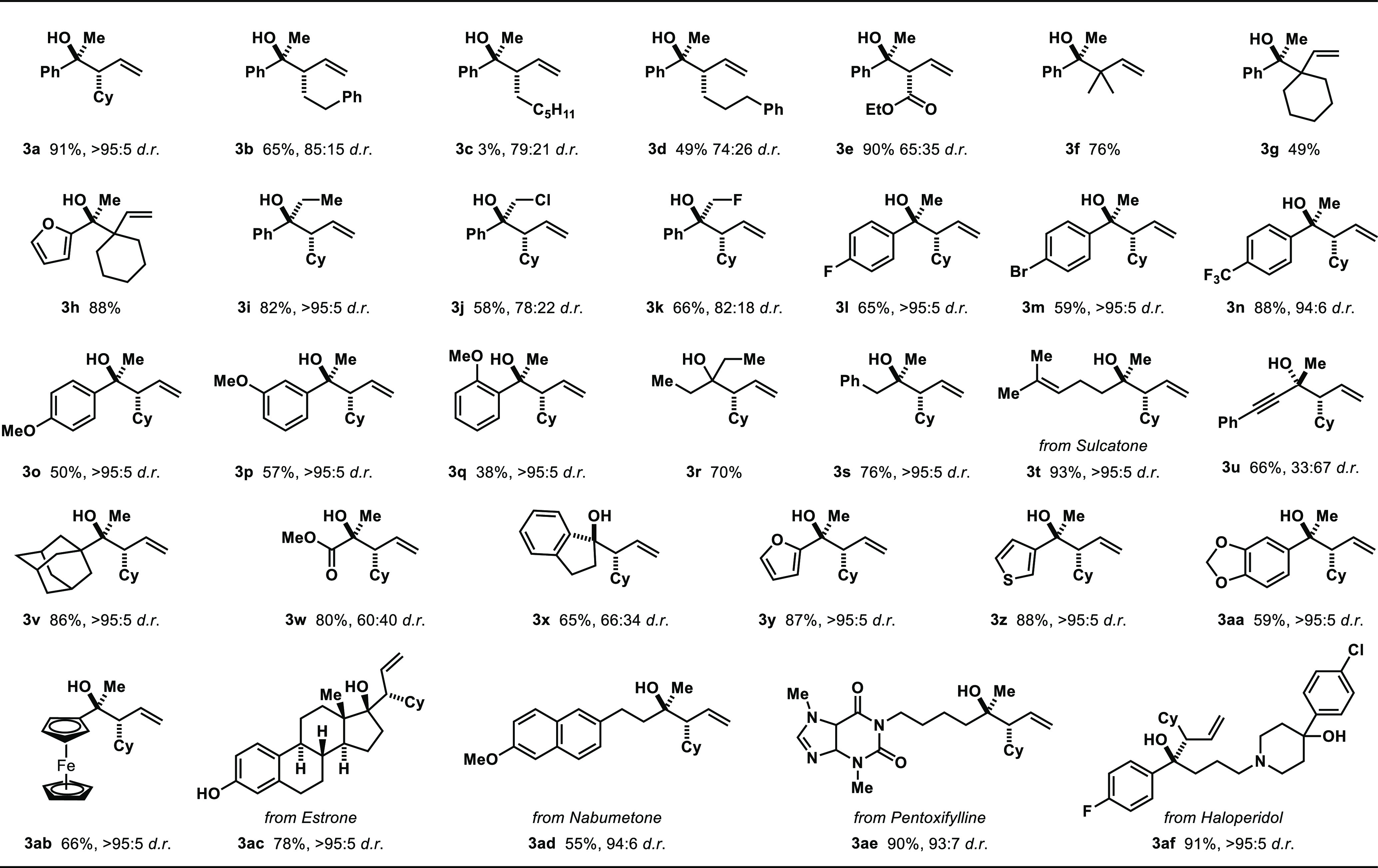
Substrate Scope of Boron-Catalyzed Allylation of Ketones^a^

a Reaction conditions unless stated otherwise: [H-*B*-9-BBN]_2_ (5 mol %), HBpin (1.2 equiv), ketone (1.0 equiv), allene (1.0 equiv), 16 h, hexane, reflux. Diastereoselectivity determined by ^1^H NMR spectroscopy of the crude reaction mixture.

**Scheme 1:**
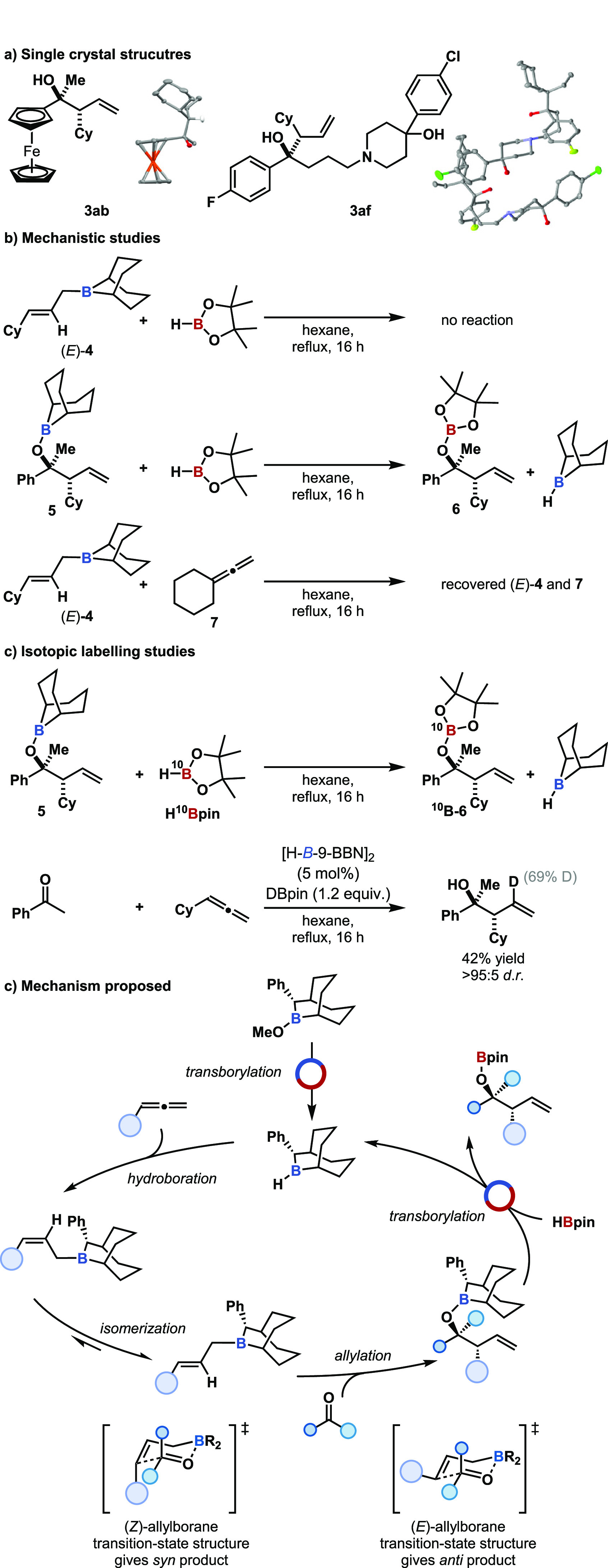
(a) Single-Crystal X-ray Structures of Products **3ab** and **3af**. (b) Mechanistic Studies. (c) Proposed Reaction Mechanism. Thermal ellipsoids for crystal structures of **3ab** and **3af** are shown at the 50% probability level; red = oxygen, orange = iron, gray = carbon, white = hydrogen, yellow = fluorine, orange = chlorine.

After developing the diastereoselective boron-catalyzed allylation of ketones, attention turned to the development of an enantioselective process. Very few stoichiometric allylic borane reagents are reported to react with ketones in good enantioselectivity,^12^ with one exception being Soderquist’s enantioenriched 9-borabicyclo[3.3.2]decane reagents.^49^ These secondary boranes were shown to require low temperatures to achieve high enantioselectivity (−78 °C up to >99:1 *e.r.*) in the allylation of ketones; however, only a moderate loss of stereoselectivity was observed when the reaction temperature was increased to 0 °C (95:5 *e.r.*). Application of Soderquist’s boranes to this catalysis protocol would require enantio- and diastereoselectivity to be maintained at significantly higher reaction temperatures for effective catalyst turnover by B–O transborylation. Unlike Soderquist’s study, where the allylic 9-borabicyclo[3.3.2]decanes were prepared prior to reaction, here, allene hydroboration would be used to generate the allylic borane in situ and thus the secondary borane was required. This was easily accessed by B–O transborylation from the *B*-methoxy-9-borabicyclo[3.3.2]decane precatalyst with HBpin.

Use of *B*-methoxytrimethylsilyl-9-borabicyclo[3.3.2]decane showed no turnover (see Supporting Information). Switching to the phenyl-substituted variant, (*S*)-*B*-methoxy-phenyl-9-borabicyclo[3.3.2]decane [(*S*)-Ph-BBD-OMe], and neat reaction conditions (see Supporting Information Table S2 for details), asymmetric allylation was achieved using (*S*)-Ph-BBD-OMe (10 mol %) as a catalyst to give the enantioenriched homoallylic alcohol, (2*R*,3*S*)-3-cyclohexyl-2-phenylpent-4-en-2-ol, in good yield, excellent diastereoselectivity, and good enantioselectivity [(2*R*,3*S*)**-3a**, 71%, >95:5 *d.r.*, 89:11 *e.r.*]. Increasing the reaction temperature to 80 °C reduced reaction times (16 h); however, reduced diastereoselectivity and enantioselectivity (75:25 *d.r.*, 78:22 *e.r.*) were also observed. Reaction at 60 °C resulted in a slightly reduced stereoselectivity (90:10 *d.r.*, 85:15 *e.r*.). Application of alternative turnover reagents was unsuccessful; ^*i*^Pr_2_NBH_2_ resulted in recovery of starting materials, and HBcat gave 1-phenylethanol by direct reduction (1,2-hydroboration) of acetophenone to 1-phenylethanol. The optimized conditions were applied to a range of allenes and ketones (Table 2).

**Table 2:**
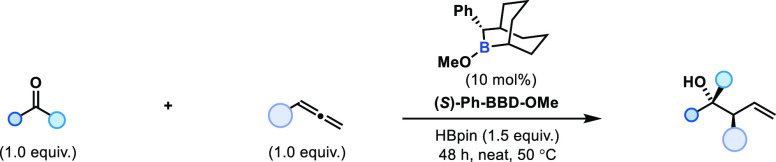

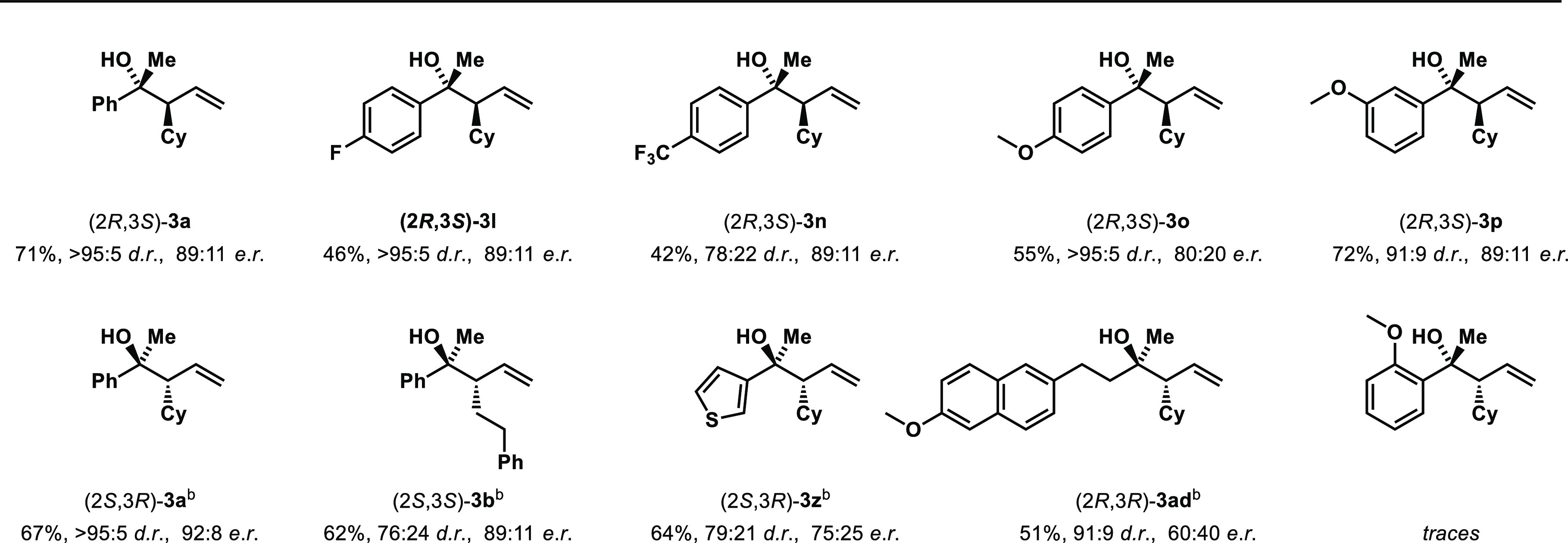
Substrate Scope of Asymmetric Boron-Catalyzed Allylation of Ketones^a^

a Reaction conditions unless stated otherwise: (*S*)-Ph-BBD-OMe (10 mol %), HBpin (1.4 equiv.), ketone (1.0 equiv.), allene (1.0 equiv.), 48 h, 50 °C. Diastereoselectivity determined by ^1^H NMR spectroscopy of the crude reaction mixture, and enantioselectivity determined by chiral HPLC.

b Reaction using (*R*)-Ph-BBD-OMe (10 mol %) as precatalyst.

Other ketones were successfully reacted including those bearing fluoro [(2*R*,3*S*)-**3l**, 46%, >95:5 *d.r.*, 89:11 *e.r*.] and trifluoromethyl [(2*R*,3*S*)**-3n**, 42%, 78:22 *d.r.*, 89:11 *e.r*.] substituents. 4-Methoxyacetophenone was reacted in reduced enantioselectivity [(2*R*,3*S*)**-3o**, 55%, >95:5 *d.r*., 80:20 *e.r.*]; however, 3-methoxyacetophenone was reacted in enantioselectivity comparable to that of other substrates [(2*R*,3*S*)-**3p**, 72%, 91:9 *d.r.*, 89:11 *e.r*.]. *ortho*-Substituted 2-methoxyacetophenone was unreactive despite being a viable substrate for the previous achiral reaction, possibly due to the increased steric bulk of phenyl-BBD compared to that of [H-*B*-9-BBN]_2_. The (*R*)-enantiomer of the catalyst could be used to give products of the opposite enantiomer [(2*S*,3*R*)-**3a**, 67%, >95:5 *d.r*., 92:8 *e.r*.] with equal levels of enantioselectivity and diastereoselectivity. Coupling using penta-3,4-dienylbenzene gave moderate diastereoselectivity and good enantioselectivity [(2*S*,3*S*)-**3b**, 62%, 76:24 *d.r*., 89:11 *e.r*.] of the homoallylic alcohol; however, application to di- and trisubstituted allenes was unsuccessful. Thiophene-bearing ketone reacted with reduced enantioselectivity [(2*S*,3*R*)**-3z**, 64%, 79:21 *d.r.*, 75:25 *e.r.*]. The asymmetric allylation of Nabumetone resulted in poor enantioselectivity [(2*R*,3*R*)**-3ad**, 51%, 91:9 *d.r*., 60:40 *e.r*.], presumably due to the minimal steric bias between methyl and the alkyl chain of the ketone.

The mechanism of catalytic turnover was investigated as both allylic borane **4** and borinic ester **5** could plausibly undergo transborylation, B–C and B–O transborylation respectively, with only transborylation of the borinic ester **5** enabling turnover and catalyst regeneration.^50−59^ Reaction of allylic borane **4** with HBpin under catalytic reaction conditions gave no B–C transborylation with only the recovery of starting material (Scheme 1b). Reaction of borinic ester **5** with HBpin under catalytic reaction conditions gave boronate ester **6** and regeneration of the catalyst H-*B*-9-BBN, observed by ^11^B NMR spectroscopy (see Supporting Information). It was therefore proposed that the catalytic protocol proceeds by B–O transborylation. To confirm that the isomerization of allylic borane diastereomers was an intramolecular process, a crossover experiment was carried out between allylic borane **4** and vinylidenecyclohexane. No crossover was observed, confirming the intramolecular nature of isomerization of (*Z*)-allylic borane to the (*E*)-allylic borane (Scheme 1b). Single turnover experiments were used to identify and characterize in solution each intermediate on the catalytic cycle (Scheme 1c).

A catalytic cycle for the allylation of ketones was thus proposed, whereby, in the case of the asymmetric reaction, the precatalyst was activated in situ by reaction with HBpin (Scheme 1c). The dialkylborane reacted with the allene to give a (*Z*)-allylic borane which isomerized to the (*E*)-allylic borane (*E*)**-4** by a series of 1,3-boratropic shifts and with a *d.r*. reflective of thermal isomerization.^40,60^ The (*E*)-allylic borane then reacted with the ketone, likely through a Zimmerman–Traxler-type transition-state structure^61^ controlling diastereo- and enantioselectivity, giving the branched homoallylic borinic ester **5** only. Diastereoselectivity was reflective of the (*E*)-allylic borane *d.r.* and the pseudoaxial versus pseudoequatorial positioning of the ketone substituents in the Zimmerman–Traxler-type transition-state structure. In line with stoichiometric reports^62,63^ the (*E*)-allylic borane gave an *anti*-homoallylic borinic ester. Reaction with HBpin regenerated the catalyst and gave the product as a Bpin-protected alcohol, alkoxy boronic ester **6**.

In summary, a protocol for the boron-catalyzed allylation of ketones with allenes has been developed, giving homoallylic alcohol products in excellent yields, diastereoselectivity, and enantioselectivity. The unique mechanism of catalysis allows for the application of allenes rather than preformed allylic metal(loid) species, with an allylic borane formed in situ through direct reaction with the borane catalyst. This reactivity provides the first example of transborylation in carbon–carbon bond forming reactions and is the first example of a main-group-catalyzed ketone allylation with allenes. The reaction was applied to a variety of electronically and sterically differentiated allenes and ketones exhibiting excellent functional group tolerance, including across a range of reducible functionalities. This protocol was expanded to an asymmetric variant using Soderquist’s borane to give homoallylic alcohols with good diastereoselectivity and enantioselectivity.

## Supporting Information Available

Additional discussion, experimental procedures, characterization data and NMR spectra (PDF)
X-ray crystallographic files for **3af** (ZIP)
X-ray crystallographic files for **3ab** (ZIP)
