# Supplementary material for: Boron-Catalyzed, Diastereo- and Enantioselective Allylation of Ketones with Allenes
Source: ACS Catal. 2022 Aug 22;12(17):10887–93. doi: 10.1021/acscatal.2c03158 (PMC9442582; doi:10.1021/acscatal.2c03158)
Supplement: Supplementary file 1 — cs2c03158_si_001.pdf [file cs2c03158_si_001.pdf]

Supporting Information

A Boron-catalyzed, Diastereo- and Enantioselective Allylation of Ketones with Allenes

Kieran Nicholson,[a] Yuxuan Peng, [a] Natalia Llopis, [a] Dominic Willcox, [a] Gary Nichol, [a] Thomas Langer,[b] Stephen P. Thomas*[a]

[a] EaStCHEM School of Chemistry, The University of Edinburgh, Joseph Black Building, Edinburgh, EH9 3FJ, UK.

[b] Pharmaceutical Technology & Development, Chemical Development UK, AstraZeneca, Silk Road, Macclesfield, SK10 2NA, UK.

[c] Instituto de Síntesis Orgánica and Dpto. de Química Orgánica, Universidad de Alicante, Apdo. 99, 03080 Alicante, Spain.

E-mail: stephen.thomas@ed.ac.uk

Contents

S1 General Experimental Information 3

S2 Optimization of reaction conditions 5

S3 Synthesis of allenes 7

S4 Preparation of enantiopure catalyst 9

S5 General experimental procedures 15

S6 Substrate scope 16

S7 Mechanistic studies 57

S8 NMR Data 59

S9 HPLC data 137

S10 Determination of reaction diastereoselectivity 157

S11 Crystallographic data 183

S12 References 206

S1 General Experimental Information

Reaction Setup: All reactions were carried out in oven (185 °C) dried glassware, which had been cleaned using base (KOH in propan-2-ol) and acid (HCl in H_2O) baths. All air- and moisture sensitive reactions were carried out using an argon atmosphere glovebox or a Schleck line (nitrogen). All reported reaction temperatures corresponded to external bath temperatures. Room temperature was approximately 20 °C. "Brine" refers to a saturated solution of sodium chloride in H_2O.

NMR Spectroscopy: ^{1}H, ^{13}C, ^{11}B and ^{19}F NMR Spectrum were recorded on Bruker Avance III 400 and 500 MHz; Bruker AVI 400 MHz; Bruker Avance I 600 MHz spectrometers. Chemical shifts are reported in parts per million (ppm). ^{1}H NMR Spectrum were referenced to the residual proteosolvent peak ($CHCl_3$: 7.26 ppm, CH_2Cl_2: 5.32 ppm, THF: 1.73 ppm). ^{13}C NMR Spectrum were referenced to the solvent peak ($CDCl_3$: 77.00 ppm). Multiplicities are shows as s (singlet), d (doublet), t (triplet), q (quartet), quin. (quintet), sext. (sextet), sept. (septet), non. (nonet), ap. (apparent). Coupling constants, *J*, are reported in Hertz, rounded to the nearest 0.1 Hz and as observed. Integration of peaks is provided with the assignments indicated where appropriate.

Infrared Spectroscopy: Infra-red (IR) Spectrum were recorded on a Perkin-Elmer Spectrum One FT-IR, or Shimadzu IRAffinity-1 spectrometer (serial no. A213749) spectrometer. Peaks are reported in cm^{-1} with indicated relative intensities: s (strong, 0-33% T), m (medium, 34-66% T), w (weak, 67-100% T), and br (broad).

Chromatography: Column chromatography was carried out on a Teledyne ISCO CombiFlash NextGen 300+ using RediSep R_f normal phase silica flash columns (12, 25, 40, or 80 g; 20-40 microns). Substrates were purified using 40/60 petroleum ether and EtOAc on a gradient of 100:0 to 0:100 with flow rates of 10-110 mL min^{-1} depending on the size of column and ΔR_f.

***Mass Spectrometry*:** Mass spectrometry (MS) was performed by the University of Edinburgh, School of Chemistry, Mass Spectrometry Laboratory. High resolution mass Spectrum were recorded on a VG autospec, or Thermo/Finnigan MAT 900, mass spectrometer. Electron Impact (EI^+) Spectrum were performed at 70 eV using methane as the carrier gas, with either a double focusing sector field (DFSF) or time-of-flight (TOF) mass analyzer. Chemical Ionization (CI^+) Spectrum were performed with methane reagent gas, with either a double focusing sector field (DFSF) or time-of-flight (TOF) mass analyzer. Electrospray Ionization (ESI^+) Spectrum were performed using a time-of-flight (TOF) mass analyzer. Data are reported in the form of m/z (intensity relative to the base peak = 100).

Melting Points: Melting points were determined using a Stuart Scientific SMP10, or Griffin Gallankamp and are uncorrected.

***Chemicals**:* All reagents were purchased from Sigma Aldrich, Acros Organics or Alfa Aesar, or were synthesised in the laboratory.

Solvents: All solvents for air- and moisture sensitive techniques were obtained from an anhydrous solvent system (Innovative Technology). Reaction solvents tetrahydrofuran (THF) (Fisher, HPLC grade), ether (Et_2O) (Fisher, BHT stabilized ACS grade), and dichloromethane (CH_2Cl_2) (Fisher, unstabilised HPLC grade) were dried by percolation through two columns packed with neutral alumina under a positive pressure of argon. Toluene (ACS grade) was dried by percolation through a column packed with neutral alumina and a column packed with Q5 reactant (supported copper catalyst for scavenging oxygen) under a positive pressure of argon. Solvents for filtration, transfers, chromatography, and recrystallization were dichloromethane (CH_2Cl_2) (ACS grade), ether (Et_2O) (Fisher, BHT stabilised ACS grade), ethyl acetate (EtOAc) (Fisher, ACS grade), *n*-hexane (Optima), methanol (MeOH) (ACS grade), pentane (ACS grade), and petroleum ether (40–60°C, ACS grade).

Diastereoselectivity: Diastereoselectivity was determined by ^{1}H NMR of the crude reaction mixture.

S2 Optimization of reaction conditions

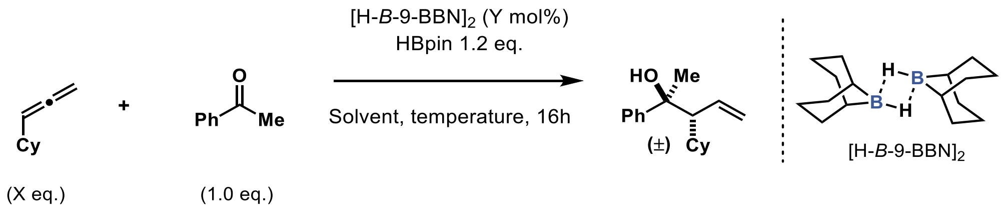

Entry	Allene (X. Eq.)	Catalyst (Y mol%)	Solvent	Temp. (°C)	Yield (%)	*d.r.*
1	1	10	THF	20	82	41:59
2	1	10	THF	40	87	12:88
3	1	10	THF	60	56	8:92
4	1	10	THF	80	80	5:95
5	1	5	THF	80	73	5:95
6	1	2.5	THF	80	80	5:95
7	1	5	*n*-Hexane	80	>95	5:95
8	1	5	Pentane	80	>95	5:95
9	1	5	CH_2Cl_2	80	>95	5:95
10	1	5	Toluene	80	>95	5:95
11	1	5	EtOAc	80	90	5:95
12	1	5	*n*-Hexane	70	86	12:88
13	1	5	*n*-Hexane	60	91	20:80
14	1	5	*n*-Hexane	50	>95	15:85
15	1.5	5	*n*-Hexane	80	>95	5:95
16	2	5	*n*-Hexane	80	>95	25:75
17	3	5	*n*-Hexane	80	>95	40:60
18	1	0	THF	80	0	-

Table S1: Yields determined by ^{1}H NMR spectroscopy with comparison to 1,3,5-trimethoxybenzene as an internal standard. Temperatures refer to that of the external bath. Diastereomeric ratio determined by ^{1}H NMR spectroscopy of the crude reaction mixtures after quenching with SiO_2.

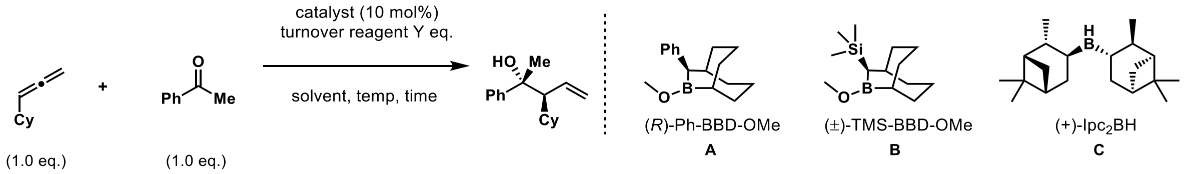

Entry	Cat.	Solvent	Turnover reagent (Y. eq)	Temp. (°C)	Time	Yield (%)	*d.r.*	*e.r.*
1	B	THF	HBpin (1.5 eq.)	80	16 h	trace	-	-
2	B	neat	HBpin (1.5 eq.)	80	16 h	trace	-	-
3	C	THF	HBpin (1.5 eq.)	20	16 h	trace	-	-
4	C	THF	HBpin (1.5 eq.)	80	16 h	trace	-	-
5	A	neat	HBcat (1.5 eq.)	20	16 h	0[a]	-	-
6	A	neat	$^{i}Pr_2NBH_2$ (10 eq.)	20	16 h	0	-	-
7	A	neat	HBpin (1.5 eq.)	20	16 h	0	-	-
8	A	neat	$^{i}Pr_2NBH_2$ (10 eq.)	80	16 h	trace	-	-
9	A	neat	HBpin (1.5 eq.)	80	16 h	70%	25:75	78:22
10	A	neat	HBpin (1.5 eq.)	40	48 h	trace	-	-
11	A	neat	HBpin (1.5 eq.)	50	48 h	81%	5:95	89:11
12	A	neat	HBpin (1.5 eq.)	60	48 h	85%	10:90	85:15
13	A	neat	HBpin (1.5 eq.)	50	16 h	25%	-	-

Table S2: Yields determined by ^{1}H NMR spectroscopy with comparison to 1,3,5-trimethoxybenzene as an internal standard. Temperatures refer to that of the external bath. Diastereomeric ratio determined by ^{1}H NMR spectroscopy of the crude reaction mixtures after quenching with SiO_2. Enantiomeric ratio determined by HPLC of the isolated product.

S3 Synthesis of allenes

Penta-3,4-dienyl-benzene

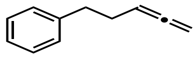

According to the procedure of Chanthamath,[1] but-3-ynylbenzene (3.5 mL, 25 mmol) and dicyclohexylamine (9 mL, 45 mmol) were added to a stirred solution of paraformaldehyde (1.88 g, 72.5 mmol) and CuI (2.4 g, 12.5 mmol), in dioxane (150 mL) . The resulting mixture was heated under reflux under an inert atmosphere for 16 hours. Upon cooling to room temperature the crude reaction mixture was filtered, aqueous HCl (2 M) was added until the pH = 2 when the mixture was extracted with diethyl ether (3 x 20 mL). The combined organic phases were washed with brine (20 mL) and dried (Na_2SO_4). The crude product was concentrated under reduced pressure and purified by flash column chromatography (CombiFlash Isco NextGen300+, 24 g SiO_2, 50 mm Ø, petroleum ether/ ethyl acetate 4:1) to give the allene as a colourless oil (3.08 g, 21.4 mmol, 86%).

^{1}H NMR (600 MHz, $CDCl_3$) δ 7.30 (td, *J* = 7.2, 1.5 Hz, 2H), 7.23 – 7.17 (m, 3H), 5.16 (*app* p, *J* = 6.7 Hz, 1H), 4.69 (m, 2H), 2.78 – 2.68 (m, 2H), 2.37 – 2.28 (m, 2H).

^{13}C NMR (126 MHz, $CDCl_3$) 208.5, 141.6, 128.4, 128.2, 125.8, 89.3, 75.0, 35.3, 29.9

The data were in accordance with those previously reported.[1]

Trideca-1,2-diene

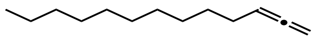

Aqueous NaOH (50 mol%, 50 mL) was added dropwise to a solution of dodec-1-ene (9.6 mL, 44 mmol), bromoform (9.7 mL, 110 mmol) and *N*-benzyl-*N*,*N*,*N*-triethylammonium bromide (1.2 g, 4.4 mmol) in dichloromethane (50 mL). After addition the mixture was heated under reflux overnight. Upon cooling water (50 mL) was added to the crude reaction mixture and then extracted with dichloromethane (3 x 50 mL), the combined organic extracts were dried ($MgSO_4$) and concentrated. The crude product, a brown oil (7.5 g) was used in the next step without further purification. EtMgBr (6 mL, 3M, 18 mmol) was added dropwise to a solution of crude 1,1-dibrom-2-decylcyclopropan (5.5 g, 16 mmol) in THF (50 mL) at 0 °C. After stirring for 16 hours the reaction mixture was warmed to room temperature and quenched with aqueous HCl (2 M, 20 mL), diluted with diethyl ether (100 mL), and washed with water (3 x 20 mL). The organic phases were concentrated, dried ($MgSO_4$) and purified by flash column chromatography (CombiFlash Isco NextGen300+, 24 g SiO_2, 50 mm Ø, petroleum ether) to give the allene (2.7 g, 15.0 mmol, 94%) as a colourless oil.

^{1}H NMR (400 MHz, $CDCl_3$) 5.12 (p, *J* = 6.8 Hz, 1H), 4.67 (dt, *J* = 6.6, 3.2 Hz, 2H), 2.09 – 1.95 (m, 2H), 1.40 – 1.24 (m, 13H), 0.95 – 0.82 (m, 6H).

^{13}C NMR (126 MHz, $CDCl_3$) 208.5, 90.1, 74.5, 31.9, 29.6, 29.6, 29.5, 29.4, 29.2, 29.1, 28.3, 22.7, 14.1.

Data were in accordance with those previously recorded.[2]

S4 Preparation of enantiopure catalyst

Benzyl *N*,*N*-di*iso*propylcarbamate

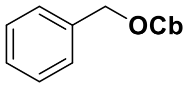

According to the procedure of Aggarwal,[3] benzyl alcohol (13.2 mL, 127 mmol) was added to a stirred solution of *N*,*N*-di*iso*propylcarbamoyl chloride (25.0 g, 153 mmol) and triethylamine (23.0 mL, 165 mmol) in dichloromethane (130 mL) . The resulting mixture was refluxed under an inert atmosphere for 24 hours. Upon cooling to room temperature, the reaction was diluted with water (200 mL), and then extracted with dichloromethane (3 × 100 mL). The combined organic phases were dried (Na_2SO_4) and concentrated *in vacuo*. The crude oil was purified by flash column chromatography (SiO_2, 50 mm Ø, petroleum ether/ethyl acetate 9:1) to give benzyl *N*,*N*-di*iso*propylcarbamate as a colourless oil (22.6 g, 96 mmol, 76%).

^{1}H NMR (600 MHz, $CDCl_3$) 7.38 – 7.33 (m, 4H), 7.32 – 7.28 (m, 1H), 5.14 (s, 2H), 3.94 (br d, *J* = 124 Hz, 2H), 1.22 (d, *J* = 6.8 Hz, 12H).

^{13}C NMR (126 MHz, $CDCl_3$) 155.6, 137.3, 128.6, 128.0, 127.9, 66.6, 46.2 (br), 21.3 (br).

The data were in accordance with those previously reported.[3]

(±)-*B*-Methoxy-10-phenyl-9-borabicyclo[3.3.2]decane

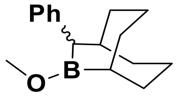

sec-Butyl lithium (72.0 mL, 1.34 M in hexanes, 96.0 mmol) was added dropwise to a pre-cooled solution of benzyl *N*,*N*-di*iso*propylcarbamate (22.6 g, 96.0 mmol) in diethyl ether (300 mL) at –78 °C and stirred for 4 hours at –78 °C, to give an orange solution. MeO-*B*-9-BBN (96.0 mL, 1.00 M in hexanes, 96.0 mmol) was added dropwise and left to stir for 30 minutes at –78 °C. The solution was allowed to warm to room temperature and left to stir for 16 hours. The resulting suspension was filtered and the solid washed with diethyl ether (3 × 50 mL), the solvent was removed at atmospheric pressure and the resulting oil was distilled (92 °C, 5.0×10^{-2} mbar) to give (±)-*B*-methoxy-10-phenyl-9-borabicyclo[3.3.2]decane as a colourless oil (14.9 g, 60.6 mmol, 63%).

Note: Any remaining benzyl N,N-*diisopropylcarbamate will co-distil with the product, but will not affect the subsequent resolution if contamination is <20%.*

^{1}H NMR (600 MHz, C_6D_6) 7.21 – 7.16 (m, 2H), 7.11 – 7.04 (m, 3H), 3.22 (s, 3H), 2.45 (d, *J* = 3.54 Hz, 1H), 2.32 – 2.24 (m, 1H), 1.89 – 1.27 (m, 13H).

^{11}B NMR (128 MHz, C_6D_6) 54.8.

^{13}C NMR (126 MHz, C_6D_6) 145.4, 130.9, 128.6, 128.4, 125.4, 53.6, 43.5 (br), 39.4, 32.0 (br), 29.6, 28.6, 27.0, 24.8, 22.1.

The data were in accordance with those previously reported.[4]

(+)-*B*-[(1*S*,2*S*)-*N*-Methylpseudoephedrinyl)]-(10*S*)-phenyl-9-borabicyclo[3.3.2]decane

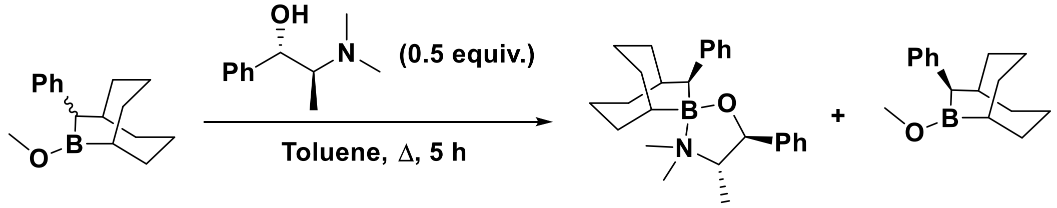

According to the modified procedure of Soderquist,[4] (1*S*,2*S*)-*N*-methylpseudoephedrine (5.50 g, 31.0 mmol) was dissolved in the minimum volume of toluene (*ca.* 20 mL), then added dropwise to (±)-*B*-methoxy-10-phenyl-9-borabicyclo[3.3.2]decane (14.9 g, 60.6 mmol). The resulting solution was refluxed for 5 hours, then slowly cooled to room temperature, resulting in colourless, needle-like crystals. The supernatant was decanted, the crystals washed with toluene (10 mL) and pentane (3 × 30 mL), and dried *in vacuo* giving (+)-*B*-[(1*S*,2*S*)-*N*-methylpseudoephedrinyl)]-(10*S*)-phenyl-9-borabicyclo[3.3.2]decane as colourless, needle-like crystals (2.04 g, 5.23 mmol, 17%). The solvent was removed from the filtrate *in vacuo* and the resulting gum was recrystallized from toluene to give two further crops of crystals, which were combined to give (+)-*B*-((1*S*,2*S*)-*N*-methylpseudoephedrinyl))-(10*S*)-phenyl-9-borabicyclo[3.3.2]decane as colourless needles (combined yield: 10.3 g, 26.4 mmol, 85%).

^{1}H NMR (600 MHz, C_6D_6) 7.78 – 7.52 (m, 1.8H), 7.37 – 7.18 (m, 5.8H), 7.16 – 7.10 (m, 2.4H), 4.50 (d, *J* = 8.0 Hz, 0.1H), 4.07 (d, *J* = 9.6 Hz, 0.9H), 2.75 – 2.66 (m, 0.8H), 2.46 – 2.39 (m, 0.9H), 2.35 – 2.29 (m 1.2H), 2.26 – 2.16 (m, 1.8H), 2.13 – 1.99 (m, 2.2), 1.95 – 1.69 (m, 13.3H), 1.60 – 1.52 (m, 1.2H), 1.14 – 1.06 (m, 0.9H), 0.45 (d, *J* = 6.7 Hz, 0.5H), 0.11 (d, *J* = 6.8 Hz, 2.5H).

^{11}B NMR (128 MHz, C_6D_6) 54.5 (minor), 19.2 (major).

^{13}C NMR (126 MHz, C_6D_6) 152.7, 143.4, 131.7, 131.4, 128.5, 128.4, 128.3, 127.8, 127.4, 124.2, 81.3, 67.6, 42.2, 41.9, 40.9, 32.5, 31.7, 30.2, 30.1, 29.6, 25.3, 25.1, 22.8, 22.7, 11.1, 7.2.

In solution the compound exists as an "open" and "closed" adduct, leading to two sets of signals for each environment.

The data were in accordance with those previously reported.[4]

(-)-*B*-[(1*R*,2*R*)-*N*-Methylpseudoephedrinyl)]-(10*R*)-phenyl-9-borabicyclo[3.3.2]decane

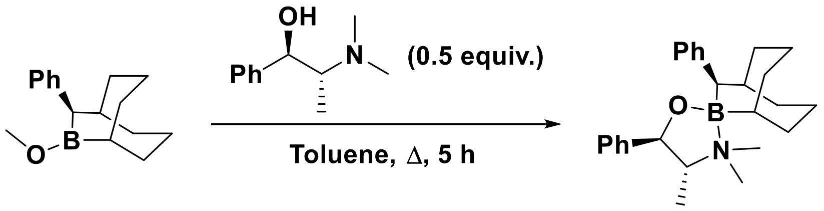

According to the modified procedure of Soderquist,[4] the filtrate was concentrated *in vacuo* to give a viscous oil. (1*R*,2*R*)-*N*-Methylpseudoephedrine (5.50 g, 31.0 mmol) was dissolved in the minimum volume of toluene (*ca.* 20 mL), then added dropwise to the oil. The resulting solution was heated under reflux for 5 hours, then slowly cooled to room temperature, resulting in the formation of colourless, needle-like crystals. The supernatant was decanted, the crystals washed with toluene (10 mL) and pentane (3 × 30 mL), and dried *in vacuo* giving (+)-*B*-[(1*R*,2*R*)-*N*-methylpseudoephedrinyl)]-(10*R*)-phenyl-9-borabicyclo[3.3.2]decane as colourless needles (10.3 g, 24.6 mmol, 85%).

^{1}H NMR (600 MHz, C_6D_6) 7.78 – 7.52 (m, 1.8H), 7.37 – 7.18 (m, 5.8H), 7.16 – 7.10 (m, 2.4H), 4.50 (d, *J* = 8.0 Hz, 0.1H), 4.07 (d, *J* = 9.6 Hz, 0.9H), 2.75 – 2.66 (m, 0.8H), 2.46 – 2.39 (m, 0.9H), 2.35 – 2.29 (m 1.2H), 2.26 – 2.16 (m, 1.8H), 2.13 – 1.99 (m, 2.2), 1.95 – 1.69 (m, 13.3H), 1.60 – 1.52 (m, 1.2H), 1.14 – 1.06 (m, 0.9H), 0.45 (d, *J* = 6.7 Hz, 0.5H), 0.11 (d, *J* = 6.8 Hz, 2.5H).

^{11}B NMR (128 MHz, C_6D_6) 54.5 (minor), 19.2 (major).

^{13}C NMR (126 MHz, C_6D_6) 152.7, 143.4, 131.7, 131.4, 128.5, 128.4, 128.3, 127.8, 127.4, 124.2, 81.3, 67.6, 42.2, 41.9, 40.9, 32.5, 31.7, 30.2, 30.1, 29.6, 25.3, 25.1, 22.8, 22.7, 11.1, 7.2.

In solution the compound exists as an "open" and "closed" adduct, leading to two sets of signals for each environment.

The data were in accordance with those previously reported.[4]

B-Methoxy-(10S)-phenyl-9-borabicyclo[3.3.2]decane

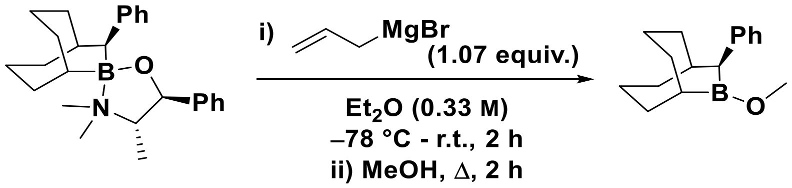

A stirred suspension of (+)-*B*-[(1*S*,2*S*)-*N*-methylpseudoephedrinyl)]-(10*S*)-phenyl-9-borabicyclo[3.3.2]decane (3.68 g, 9.45 mmol) in diethyl ether (30 mL) was cooled to –78 °C. Allyl magnesium bromide (10.1 mL, 1.0 M in diethyl ether, 10.1 mmol) was added dropwise, then the solution was warmed to room temperature and stirred for 2 hours. The solution was concentrated *in vauco* and diluted with pentane (10 mL). The pentane solution was filtered through a Celite® plug and the filter cake washed with pentane (2 × 10 mL). Volatiles were removed *in vacuo* to give a colourless oil, to which methanol (20 mL) was slowly added, and the mixture was heated under reflux for 2 hours. Volatiles were removed *in vacuo* and the resulting oil was distilled (92 °C, 5.0 × 10^{-2} mbar) to give *B*-methoxy-(10*S*)-phenyl-9-borabicyclo[3.3.2]decane as a colourless oil (1.43 g, 5.88 mmol, 62%).

^{1}H NMR (600 MHz, C_6D_6) 7.21 – 7.16 (m, 2H), 7.11 – 7.04 (m, 3H), 3.22 (s, 3H), 2.45 (d, *J* = 3.54 Hz, 1H), 2.32 – 2.24 (m, 1H), 1.89 – 1.27 (m, 13H).

^{11}B NMR (128 MHz, C_6D_6) 54.8.

^{13}C NMR (126 MHz, C_6D_6) 145.4, 130.9, 128.6, 128.4, 125.4, 53.6, 43.5 (br), 39.4, 32.0 (br), 29.6, 28.6, 27.0, 24.8, 22.1.

The data were in accordance with those previously reported.[4]

***B*-Methoxy-(10*R*)-phenyl-9-borabicyclo[3.3.2]decane**

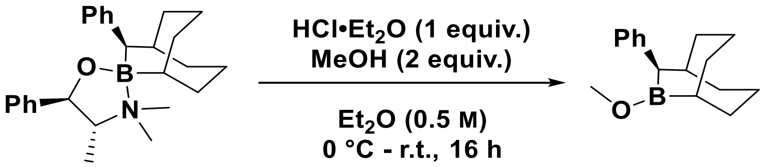

To a stirred suspension of (+)-*B*-[(1*R*,2*R*)-*N*-methylpseudoephedrinyl)]-(10*R*)-phenyl-9-borabicyclo[3.3.2]decane (1.00 g, 2.60 mmol) was added methanol (0.210 mL, 5.20 mmol) and the solution was cooled to 0 °C. A freshly prepared solution of HCl in diethyl ether (2.30 mL, 1.10 M, 2.60 mmol) was added dropwise and left to stir for 16 hours. The solution was filtered and the solid was washed with diethyl ether (3 × 5 mL). The filtrate was concentrated *in vacuo* and the resulting oil was distilled (92 °C, 5.0 × 10^{-2} mbar) to give *B*-methoxy-(10*R*)-phenyl-9-borabicyclo[3.3.2]decane as a colourless oil (470 mg, 1.94 mmol, 75%).

^{1}H NMR (600 MHz, C_6D_6) 7.21 – 7.16 (m, 2H), 7.11 – 7.04 (m, 3H), 3.22 (s, 3H), 2.45 (d, *J* = 3.54 Hz, 1H), 2.32 – 2.24 (m, 1H), 1.89 – 1.27 (m, 13H).

^{11}B NMR (128 MHz, C_6D_6) 54.8.

^{13}C NMR (126 MHz, C_6D_6) 145.4, 130.9, 128.6, 128.4, 125.4, 53.6, 43.5 (br), 39.4, 32.0 (br), 29.6, 28.6, 27.0, 24.8, 22.1.

The data were in accordance with those previously reported.[4]

S5 General experimental procedures

General procedure A

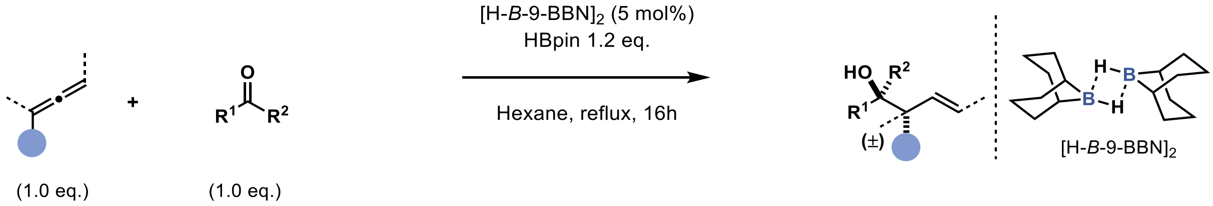

Ketone (1.0 eq.) was added to 9-borobicyclo(3.3.1)nonane [H-*B*-9-BBN]$_2$ (5 mol%), allene (1.0 eq.), HBpin (1.2 eq.) and hexane (0.5 M) were reacted in an argon filled vial under reflux for 16 hours. The reactions were quenched with silica (SiO_2, 0.1 g) and the diastereoselectivity was determined by analysis of the crude reaction mixture by ^{1}H NMR spectroscopy. The product was purified by flash column chromatography.

General Procedure B

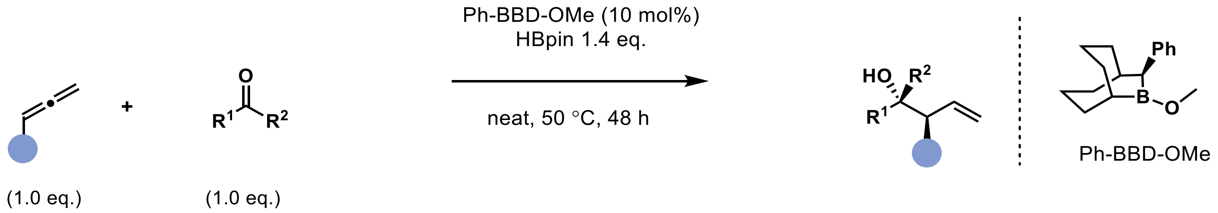

Ketone (1.0 eq.), was added to (*S*)-*B*-OMe-10-Ph-9-borabicyclo[3.3.2]decane (10 mol%) (*reaction also ran with (R) enantiomer of catalyst)*, allene (1.0 eq.) and HBpin (1.4 eq.) were reacted in an argon filled vial at 50 °C for 48 hours. The reactions were diluted with Et_2O and quenched with silica (SiO_2, 0.1 g) and the diastereoselectivity was determined by analysis of the crude reaction mixture by ^{1}H NMR spectroscopy. The product was purified by flash column chromatography and the enantiopurity measured using HPLC.

S6 Substrate scope

(2*SR*,3*RS*)-3-Cyclohexyl-2-phenylpent-4-en-2-ol **3a**

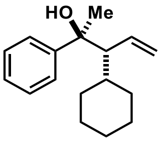

According to general procedure A, cyclohexylallene (72 µL, 0.5 mmol), acetophenone (59 µL, 0.5 mmol), HBpin (87 µL, 0.6 mmol), [H-*B*-9-BBN]$_2$ (6 mg, 0.025 mmol) were reacted in *n*-hexane (1 mL) and heated under reflux for 16 hours. The crude reaction mixture was quenched (SiO_2) and purified by flash column chromatography (CombiFlash Isco NextGen300+, 12 g SiO_2, 50 mm Ø, petroleum ether/ ethyl acetate 10:1) to give the alcohol as a colourless oil (111 mg, 0.45 mmol, 91% yield, *anti:syn* >95:5).

^{1}H NMR (600 MHz, $CDCl_3$) δ 7.46 – 7.41 (m, 2H), 7.35 – 7.29 (m, 2H), 7.25 – 7.20 (m, 1H), 5.78 (app. dt, *J* = 17.0, 10.3 Hz, 1H), 5.13 (dd, *J* = 10.2, 2.4 Hz, 1H), 4.98 (ddd, *J* = 17.0, 2.4, 0.7 Hz, 1H), 2.14 (dd, *J* = 10.5, 2.5 Hz, 1H), 1.93 (s, 1H), 1.59 (m, 5H), 1.45 – 0.77 (m, 9H).

^{13}C NMR (101 MHz, $CDCl_3$) 147.1, 136.3, 127.8, 126.6, 125.7, 118.9, 76.1, 62.6, 37.4, 34.0, 29.4, 27.5, 26.9, 26.6, 26.3.

Data were in accordance with those previously reported.[5]

(2*RS*,3*RS*)-3-(1-Ethyl-2-phenyl)-2-phenylpent-4-en-2-ol **3b**

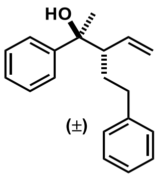

According to general procedure A, penta-3,4-dienyl-benzene (100 µL, 0.5 mmol), acetophenone (59 mg, 0.5 mmol), HBpin (87 µL, 0.6 mmol), [H-*B*-9-BBN]$_2$ (6 mg, 0.025 mmol) were reacted in *n*-hexane (1 mL) and heated under reflux for 16 hours. The crude reaction mixture was quenched (SiO_2) and purified by flash column chromatography (CombiFlash Isco NextGen300+, 12 g SiO_2, 50 mm Ø, petroleum ether/ ethyl acetate 10:1) to give the *alcohol* as a colourless oil (87 mg, 0.33 mmol, 65% yield, *anti:syn* 85:15).

^{1}H NMR (600 MHz, $CDCl_3$) 7.46 – 7.34 (*anti* + *syn*, m, 4H), 7.31 (*anti* + *syn*, m, 3H), 7.22 – 7.18 (*anti* + *syn*, m, 1H), 7.11 – 7.04 (*anti* + *syn*, m, 2H), 5.76 (*syn*, dt, *J* = 17.2, 9.8 Hz, 0.16H), 5.69 (*anti*, dt, *J* = 17.1, 10.2 Hz, 0.85 H), 5.35 – 5.30 (*anti* + *syn*, m, 1H), 5.23 (*anti*, dd, *J* = 17.2, 1.9 Hz, 0.86H), 5.18 (*syn*, dd, *J* = 17.2, 2.0 Hz, 0.16H), 2.66 (*anti* + *syn*, ddd, *J* = 14.4, 10.2, 4.6 Hz, 1H), 2.43 – 2.32 (*anti* + *syn*, m, 2H), 2.2 – 1.9 (*anti* + *syn*, br s, 1H), 1.86 (*anti* + *syn*, ddd, *J* = 13.0, 10.0, 7.3 Hz, 1H), 1.60 (*anti*, s, 2.6 H), 1.54 (*syn*, s, 0.5H), 1.42 (*anti* + *syn*, m, 1H)

^{13}C NMR (151 MHz, $CDCl_3$) **Major** (*anti*): 147.1, 139.4, 128.3, 127.1, 126.4, 119.3, 76.0, 57.1, 32.2, 29.6, 29.1, 28.2, 25.9, 23.1, 14.5. **Minor** (*syn*): 147.7, 139.1, 128.3, 126.4, 125.7, 118.5, 76.5, 56.1, 32.2 (obsc), 29.5, 29.5, 29.1, 28.1, 25.9 (obsc), 23.1 (obsc), 14.5 (obsc).

HRMS (EI^+) m/z: Calcd for $C_{19}H_{22}O$ 266.1665; Found 266.1663.

IR ν_{max} (neat) 3567 (w), 3025 (w), 2975 (w), 2861 (w), 1637 (w), 1603 (w), 1495 (m), 1446 (m).

(2*SR*,3*RS*)-3-Hexyl-2-phenylpent-4-en-2-ol **3c**

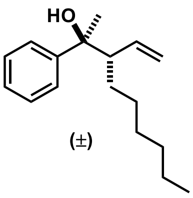

According to general procedure A, nona-1,2-diene (71 μL, 0.5 mmol), acetophenone (59 mg, 0.5 mmol), HBpin (87 μL, 0.6 mmol), [H-*B*-9-BBN]$_2$ (6 mg, 0.025 mmol) were reacted in *n*-hexane (1 mL) and heated under reflux for 16 hours. The crude reaction mixture was quenched (SiO_2) and purified by flash column chromatography (CombiFlash Isco NextGen300+, 12 g SiO_2, 50 mm Ø, petroleum ether/ ethyl acetate 10:1) to give the *alcohol* as a colourless oil (77 mg, 0.32 mmol, 63% yield *anti:syn* 79:21).

^{1}H NMR (400 MHz, $CDCl_3$) 7.48 – 7.44 (*anti*, m, 1.5 H), 7.42 – 7.39 (*syn*, m, 0.50 H), 7.38 – 7.33 (*anti* + *syn*, m, 2H), 7.25-7.15 (*anti* + *syn*, m, 1H), 5.66 – 5.55 (*anti* + *syn*, m, 1H), 5.24 – 5.20 (*anti* + *syn*, m, 1H), 5.16 (*anti*, dd, *J* = 17.1, 2.1 Hz, 0.75 H), 5.09 (*syn*, dd, *J* = 17.2, 2.2 Hz, 0.25 H), 2.33 – 2.26 (*anti* + *syn*, m, 1H), 2.11 (*anti*, br s, 0.60 H), 1.95 (*syn*, br s 0.28 H), 1.56 (*anti*, s, 2.30H), 1.54 (*syn*, s, 0.80 H), 1.43 – 1.34 (*anti* + *syn*, m, 1H), 1.33 – 0.99 (*anti* + *syn*, m, 15 H), 0.88 (*anti* + *syn*, t, 7.1 Hz, 3H).

^{13}C NMR (101 MHz, $CDCl_3$) **Major** (*anti*): 146.6, 139.0, 127.8, 126.7, 125.9, 118.8, 75.5, 56.7, 31.9, 29.5, 29.3, 28.7, 27.8, 25.5, 22.7, 14.1. **Minor** (*syn*): 147.2, 138.6, 127.9, 126.4, 125.2, 118.1, 76.0, 56.6, 31.9 (obsc), 29.5, 29.0, 28.5, 27.7, 25.5 (obsc), 22.7 (obsc), 14.1 (obsc).

HRMS (EI^+) m/z: Calcd for $C_{17}H_{26}O$ 246.1978; Found 246.1978.

IR ν_{max} (neat) 3345 (w), 3002 (w), 2934 (w), 1641 (m), 1599 (m).

(2*SR*,3*RS*)-3-(1-Propyl-3-phenyl)-2-phenylpent-4-en-2-ol **3d**

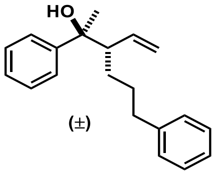

According to general procedure A, hexa-4,5-dienyl-benzene (112 µL, 0.5 mmol), acetophenone (59 mg, 0.5 mmol), HBpin (87 µL, 0.6 mmol), [H-*B*-9-BBN]$_2$ (6 mg, 0.025 mmol) were reacted in *n*-hexane (1 mL) and heated under reflux for 16 hours. The crude reaction mixture was quenched (SiO_2) and purified by flash column chromatography (CombiFlash Isco NextGen300+, 12 g SiO_2, 50 mm Ø, petroleum ether/ ethyl acetate 10:1) to give the *alcohol* as a colourless oil (69 mg, 0.25 mmol, 49% yield, *anti:syn* 74:26).

^{1}H NMR (600 MHz, $CDCl_3$) δ 7.51 – 7.42 (*anti* + *syn*, m, 2H), 7.42 – 7.37 (*anti* + *syn*, m, 2H), 7.33 – 7.25 (*anti* + *syn*, m, 3H), 7.23 – 7.17 (*anti* + *syn*, m, 1H), 7.16 – 7.06 (*anti* + *syn*, m, 2H), 5.64 (*anti* + *syn*, m, 1H), 5.25 (*anti* + *syn*, m, 1H), 5.19 (*anti*, dd, J = 17.1, 2.1, 0.76H) 5.13 (*syn*, m, 0.25H), 2.59 (*anti* + *syn*, m, 1H), 2.46 (*anti* + *syn*, m, 1H), 2.38 (*anti* + *syn*, ddd, J = 11.6, 9.4, 2.6 Hz, 1H), 1.70 – 1.61 (*anti* + *syn*, m, 1H), 1.59 (*anti*, s, 2.47H), 1.56 (*syn*, s, 0.69H) 1.54 – 1.37 (*anti* + *syn*, m, 2H), 1.24 – 1.14 (*anti* + *syn*, m, 1H) 1.02 – 0.94 (*anti*, m, 0.64H).

^{13}C NMR (151 MHz, $CDCl_3$) **Major** (*anti*): 146.6, 142.6, 138.8, 128.4, 128.3, 127.9, 126.8, 126.0, 125.7, 119.0, 75.6, 56.6, 35.8, 29.8, 28.4, 25.8. **Minor** (*syn*): 147.3, 142.7, 138.5, 128.4, 128.3, 128.0, 126.5, 125.3, 125.3, 118.3, 76.1, 55.6, 42.0, 29.7, 29.0, 28.3.

HRMS (EI$^+$) m/z: Calcd for $C_{20}H_{23}O$ 279.1743; Found 279.1743.

IR ν_{max} (neat) 3547 (w), 3060 (w), 3025 (w), 2933 (w), 2858 (w), 1637 (w), 1602 (w), 1494 (m), 1446 (m).

(3*SR*,4*RS*)-Ethyl-3-hydorxy-3-phenyl-2-vinylbutyrate **3e**

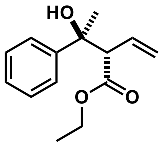

(±)

According to general procedure A, ethyl 2,3-butadienoate (58 µL, 0.5 mmol), acetophenone (59 mg, 0.5 mmol), HBpin (87 µL, 0.6 mmol), [H-*B*-9-BBN]$_2$ (6 mg, 0.025 mmol) were reacted in *n*-hexane (1 mL) and heated under reflux for 16 hours. The crude reaction mixture was quenched (SiO_2) and purified by flash column chromatography (CombiFlash Isco NextGen300+, 12 g SiO_2, 50 mm Ø, petroleum ether/ ethyl acetate 10:1) to give the alcohol as a colourless oil (105 mg, 0.45 mmol, 90% yield, *anti:syn* 65:35).

^{1}H NMR (600 MHz, $CDCl_3$) 7.46 – 7.38 (*anti* + *syn*, m, 2H), 7.36 – 7.30 (*anti* + *syn*, m, 2H), 7.25 – 7.19 (*anti* + *syn*, m, 1H), 6.12 (*syn*, ddd, *J* = 17.1, 10.2, 9.1 Hz, 0.34H), 5.77 (*anti*, *J* = 17.2, 10.3, 8.5 Hz, 0.65H), 5.40 – 5.33 (*syn*, 0.70H), 5.05 (*anti*, dd, *J* = 10.4, 1.4 Hz, 0.65H), 4.97 (*anti*, dd, *J* = 17.3, 1.2 Hz, 0.66 Hz), 4.33 (*syn*, m, 0.35H), 4.20 (*anti*, m, 1.34H), 4.07 (*anti*, s, 0.64H), (*syn*, m, 0.72H), 3.54 (*syn*, *J* = 8.5 Hz, 0.34H), 3.42 (*anti*, *J* = 8.5 Hz, 0.65H), 1.61 (*anti*, s, 2H), 1.43 (*syn*, s, 1H), 1.26 (*anti*, t, *J* = 8.5 Hz, 2H), 0.92 (*syn*, t, *J* = 8.5 Hz, 1H).

^{13}C NMR (151 MHz, $CDCl_3$) **Major** (*anti*): 174.0, 144.7, 132.1, 128.0, 126.8, 125.1, 75.1, 61.1, 60.3, 29.2, 14.1 **Minor** (*syn*): 173.9, 147.0, 132.1, 128.1, 126.9, 124.7, 74.8, 60.8, 59.7, 27.9, 13.7.

HRMS (EI^+) m/z: Calcd for $C_{14}H_{18}O_3$ 234.1251; Found 234.1249.

Data wre in accordance with those previously reported.[6]

3,3-Dimethyl-2-phenylpent-4-en-2-ol **3f**

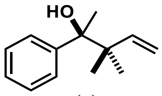

According to general procedure A, dimethylallene (50 µL, 0.5 mmol), acetophenone (59 µL, 0.5 mmol), HBpin (87 µL, 0.6 mmol), [H-*B*-9-BBN]$_2$ (6 mg, 0.025 mmol) were reacted in *n*-hexane (1 mL) and heated under reflux for 16 hours. The crude reaction mixture was quenched (SiO_2) and purified by flash column chromatography (CombiFlash Isco NextGen300+, 12 g SiO_2, 50 mm Ø, petroleum ether/ ethyl acetate 10:1) to give the alcohol as a colourless oil (72 mg, 0.38 mmol, 76% yield).

^{1}H NMR (600 MHz, $CDCl_3$) 7.47 – 7.42 (m, 2H), 7.36 – 7.30 (m, 2H), 7.30 – 7.23 (m, 1H), 5.98 (dd, *J* = 17.5, 10.8 Hz, 1H), 5.14 (dd, *J* = 10.8, 1.5 Hz, 1H), 5.08 (dd, *J* = 17.6, 1.5 Hz, 1H), 1.94 (s, 1H), 1.61 (s, 3H), 1.05 (s, 3H), 1.01 (s, 3H).

^{13}C NMR (151 MHz, $CDCl_3$) 145.3, 145.1, 127.1, 127.1, 126.5, 113.8, 77.6, 44.4, 25.4, 22.7, 22.5.

Data were in accordance with those previously reported.[7]

1-(1-Ethenylcyclohexyl)-1-phenylethan-1-ol **3g**

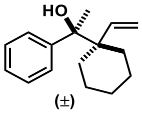

According to general procedure A, vinylidenecyclohexane (64 µL, 0.5 mmol), acetophenone (59 µL, 0.5 mmol), HBpin (87 µL, 0.6 mmol), [H-*B*-9-BBN]$_2$ (6 mg, 0.025 mmol) were reacted in *n*-hexane (1 mL) and heated under reflux for 16 hours. The crude reaction mixture was quenched (SiO_2) and purified by flash column chromatography (CombiFlash Isco NextGen300+, 12 g SiO_2, 50 mm Ø, petroleum ether/ ethyl acetate 10:1) to give the alcohol as a colourless oil (56 mg, 0.24 mmol, 49% yield).

^{1}H NMR (400 MHz, $CDCl_3$) 7.42 – 7.35 (m, 2H), 7.30 (ddd, *J* = 7.8, 6.8, 1.4 Hz, 2H), 7.26 – 7.18 (m, 1H), 5.55 – 5.44 (m, 1H), 5.43 (dd, *J* = 11.0, 2.5 Hz, 1H), 5.09 (dd, *J* = 17.0, 2.5 Hz, 1H), 2.09 (s, 1H), 1.90 – 1.82 (m, 1H), 1.65 – 1.57 (m, 1H), 1.54 (s, 3H), 1.55 – 1.46 (m, 1H), 1.50 – 1.31 (m, 2H), 1.34 – 1.19 (m, 3H), 0.97 (dddd, *J* = 16.6, 13.0, 8.5, 3.8 Hz, 1H).

^{13}C NMR (101 MHz, $CDCl_3$) 145.1, 142.5, 127.5, 126.9, 126.3, 118.0, 77.7, 48.3, 30.4, 29.0, 26.2, 25.0, 22.6, 22.3.

Data were in accordance with those previously reported.[5]

1-(1-Ethenylcyclohexyl)-1-(2-furyl)ethan-1-ol **3h**

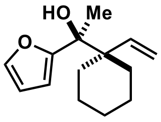

(±)

According to general procedure A, vinylidenecyclohexane (64 µL, 0.5 mmol), 2-acetylfuran (55 mg, 0.5 mmol), HBpin (87 µL, 0.6 mmol), $[H\text{-}B\text{-}9\text{-}BBN]_2$ (6 mg, 0.025 mmol) were reacted in *n*-hexane (1 mL) and heated under reflux for 16 hours. The crude reaction mixture was quenched (SiO_2) and purified by flash column chromatography (CombiFlash Isco NextGen300+, 12 g SiO_2, 50 mm Ø, petroleum ether/ ethyl acetate 10:1) to give the *alcohol* as a colourless oil (97 mg, 0.44 mmol, 88% yield).

^{1}H NMR (500 MHz, $CDCl_3$) 7.35 (dd, *J* = 1.9, 0.9 Hz, 1H), 6.34 (dd, *J* = 3.3, 1.8 Hz, 1H), 6.14 (dd, *J* = 3.3, 0.9 Hz, 1H), 5.54 (dd, *J* = 17.9, 11.1 Hz, 1H), 5.43 (dd, *J* = 11.1, 1.6 Hz, 1H), 5.11 (dd, *J* = 17.9, 1.7 Hz, 1H), 2.44 (s, 1H), 1.76 – 1.50 (m, 9H), 1.43 – 1.29 (m, 3H), 1.06 (m, 1H).

^{13}C NMR (126 MHz, $CDCl_3$) 158.7, 142.0, 140.8, 118.0, 110.0, 106.4, 76.0, 48.8, 29.9, 29.4, 26.3, 24.6, 22.4.

IR ν_{max} 3484 (br), 3091 (w), 2918 (s), 1605 (m), 1581 (m).

HRMS (EI^+) m/z: Calcd for $C_{14}H_{20}O_2$ 220.1458; Found 220.1464.

(3*RS*,4*SR*)-4-Cyclohexyl-3-phenylhex-5-en-3-ol **3i**

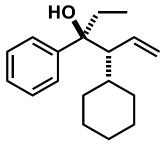

(±)

According to general procedure A, cyclohexylallene (72 μL, 0.5 mmol), propiophenone (67 μL, 0.5 mmol), HBpin (87 μL, 0.6 mmol), [H-*B*-9-BBN]$_2$ (6 mg, 0.025 mmol) were reacted in *n*-hexane (1 mL) and heated under reflux for 16 hours. The crude reaction mixture was quenched (SiO_2) and purified by flash column chromatography (CombiFlash Isco NextGen300+, 12 g SiO_2, 50 mm Ø, petroleum ether/ ethyl acetate 10:1) to give the *alcohol* as a colourless oil (106 mg, 0.41 mmol, 82% yield, *anti:syn* >95:5).

^{1}H NMR (500 MHz, $CDCl_3$) 7.42 (dd, *J* = 8.4, 1.4 Hz, 2H), 7.34 (dd, *J* = 8.5, 7.0 Hz, 2H), 7.27 – 7.22 (m, 1H), 5.75 (dt, *J* = 17.0, 10.3 Hz, 1H), 5.09 (dd, *J* = 10.1, 2.4 Hz, 1H), 4.94 (dd, *J* = 17.0, 2.4 Hz, 1H), 2.16 (dd, *J* = 10.5, 2.1 Hz, 1H), 2.08 (dq, *J* = 14.8, 7.4 Hz, 1H), 1.86 (dd, *J* = 14.3, 7.3 Hz, 1H), 1.83 – 0.96 (m, 12H), 0.77 (t, *J* = 7.4 Hz, 3H).

^{13}C NMR (126 MHz, $CDCl_3$) 144.7, 136.4, 127.8, 126.4, 126.3, 118.1, 78.8, 62.2, 37.0, 34.3, 32.7, 29.4, 27.1, 26.7, 26.5, 7.6.

IR ν_{max} (neat) 3486 (br), 2922 (s), 2850 (m), 1446 (m).

HRMS (EI^+) m/z: Calcd for $C_{18}H_{26}O$ 258.1987; Found 258.1978.

(2*SR*,3*RS*)-3-Cyclohexyl-1-chloro-2-phenylpent-4-en-2-ol **3j**

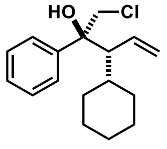

According to general procedure A, cyclohexylallene (72 µL, 0.5 mmol), 2-chloroacetophenone (77 mg, 0.5 mmol), HBpin (87 µL, 0.6 mmol), [H-*B*-9-BBN]$_2$ (6 mg, 0.025 mmol) were reacted in *n*-hexane (1 mL) and heated under reflux for 16 hours. The crude reaction mixture was quenched (SiO_2) and purified by flash column chromatography (CombiFlash Isco NextGen300+, 12 g SiO_2, 50 mm Ø, petroleum ether/ ethyl acetate 10:1) to give the *alcohol* as a colourless oil (81 mg, 0.29 mmol, 58% yield, *anti:syn* 78:22).

^{1}H NMR (500 MHz, $CDCl_3$) 7.45 – 7.31 (m, *anti* + *syn*, 5H), 5.79 – 5.65 (m, *anti* + *syn*, 1H), 5.17 – 5.12 (m, *anti* + *syn*, 1H), 5.11 – 5.05 (m, *anti* + *syn*, 1H), 4.04 – 3.94 (m, *anti*, 1.5H), 2.89 – 2.70 (m, *syn*, 0.5H), 2.64 (m, *anti* + *syn*, 1H), 2.30 (dd, *anti*, *J* = 10.6, 2.3 Hz, 0.75H), 2.05 – 0.81 (m, *anti* + *syn*, 11H), 0.77 – 0.68 (m, *anti*, 0.75H), 0.60 (qd, *anti*, *J* = 12.3, 3.3 Hz, 0.75H).

^{13}C NMR (126 MHz, $CDCl_3$) **Major (*anti*):** 141.6, 135.3, 128.0, 127.4, 126.1, 118.8, 59.5, 54.5, 52.6, 36.8, 34.0, 29.1, 26.9, 26.4, 26.3 **Minor (*syn*)**: 139.3, 137.4, 128.0, 127.5, 126.8, 117.5, 63.0, 57.3, 53.6, 38.1, 32.3, 30.7, 26.5, 26.2, 26.2.

HRMS (EI^+) m/z: Calcd for $C_{17}H_{23}OClNa$ 301.1330; Found 301.1328.

IR $\mathbf{\nu_{max}}$ 3256 (w), 3121 (w), 2984 (w), 2976 (w), 1656 (m), 1603 (w), 1497 (m).

(2*SR*,3*RS*)-3-Cyclohexyl-1-fluoro-2-phenylpent-4-en-2-ol **3k**

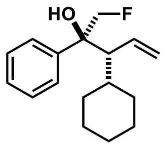

(±)

According to general procedure A, cyclohexylallene (72 µL, 0.5 mmol), 2-chloroacetophenone (69 mg, 0.5 mmol), HBpin (87 µL, 0.6 mmol), [H-*B*-9-BBN]$_2$ (6 mg, 0.025 mmol) were reacted in *n*-hexane (1 mL) and heated under reflux for 16 hours. The crude reaction mixture was quenched (SiO_2) and purified by flash column chromatography (CombiFlash Isco NextGen300+, 12 g SiO_2, 50 mm Ø, petroleum ether/ ethyl acetate 10:1) to give the *alcohol* as a colourless oil (87 mg, 0.33 mmol, 66% yield, *anti:syn* 82:18).

^{1}H NMR (500 MHz, $CDCl_3$) 7.49-7.45 (*anti* + *syn*, m, 2H), 7.43-7.36 (*anti* + *syn*, m, 2H), 7.34-7.29 (*anti* + *syn*, m, 1H), 6.05 (*syn*, dt, *J* = 17.1, 10.2 Hz, 0.18 H), 5.75 (*anti*, dt, *J* = 17.0, 10.3 Hz, 0.82H), 5.25 (*syn*, dd, *J* = 10.1, 2.2 Hz, 0.17H), 5.17 (*anti*, *J* = 10.1, 2.3 Hz, 0.83H), 5.14 – 5.05 (*anti* + *syn*, m, 1H), 4.80 – 4.50 (*anti* + *syn*, m, 2H), 2.56 (*anti*, br, s, 0.76 H), 2.46 (*syn*, br, s, 0.14H), 2.32 (*anti*, dd, *J* = 10.6, 2.5 Hz, 0.83H), 2.23 (*syn*, dd, *J* = 10.2, 2.3 Hz, 0.18H), 1.8 – 1.45 (*anti* + *syn*, m, 5H), 1.36 – 0.88 (*anti* + *syn*, m, 6H), 0.70 (*anti*, qd, *J* = 12.4, 3.5 Hz, 0.87H).

^{13}C NMR (126 MHz, $CDCl_3$) **Major (*anti*)** 141.0 (d, *J* = 3.9 Hz), 135.3, 128.1, 127.4, 125.9, 118.8, 88.1 (d, *J* = 172.5 Hz), 77.0 (d, *J* = 17.4 Hz), 58.0, 36.5, 33.9, 29.3, 26.9, 26.4, 26.3, **Minor (*syn*)** 142.8 (d, *J* = 2.5 Hz), 135.5, 128.3, 127.1, 125.5, 118.4, 89.0 (d, *J* = 173.1 Hz), 78.7, (d, *J* = 17.2 Hz), 56.2, 37.5, 33.2, 29.1, 26.7, 26.5, 26.2.

^{19}F NMR (376 MHz, $CDCl_3$) −109.0 (s).

IR ν_{max} (neat) 3586 (br), 2922 (s), 2851 (m), 1448 (m).

HRMS (EI^+) m/z: Calcd for $C_{17}H_{23}OF$ 262.1728; Found 262.1754.

(2*SR*,3*RS*)-3-Cyclohexyl-2-(4-fluorophenyl)pent-4-en-2-ol **3l**

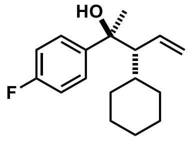

(±)

According to general procedure A, cyclohexylallene (72 µL, 0.5 mmol), 4'-fluoroacetophenone (69 mg, 0.5 mmol), HBpin (87 µL, 0.6 mmol), $[H\text{-}B\text{-}9\text{-}BBN]_2$ (6 mg, 0.025 mmol) were reacted in *n*-hexane (1 mL) and heated under reflux for 16 hours. The crude reaction mixture was quenched (SiO_2) and purified by flash column chromatography (CombiFlash Isco NextGen300+, 12 g SiO_2, 50 mm Ø, petroleum ether/ ethyl acetate 10:1) to give the *alcohol* as a colourless oil (85 mg, 0.32 mmol, 65% yield, *anti:syn* >95:5).

^{1}H NMR (500 MHz, $CDCl_3$) 7.44 – 7.36 (m, 2H), 7.04 – 6.95 (m, 2H), 5.76 (dt, *J* = 17.0, 10.3 Hz, 1H), 5.15 (dd, *J* = 10.2, 2.3 Hz, 1H), 4.99 (ddd, *J* = 17.0, 2.3, 0.7 Hz, 1H), 2.10 (dd, *J* = 10.5, 2.5 Hz, 1H), 2.06 – 1.94 (m, 1H), 1.60 (dddd, *J* = 11.2, 4.9, 3.2, 1.7 Hz, 2H), 1.56 (s, 3H), 1.51 – 0.81 (m, 9H).

^{13}C NMR (126 MHz, $CDCl_3$) 161.6, (d, *J* = 244.8 Hz), 142.7 (d, *J* = 3.1 Hz), 136.0, 127.3 (d, *J* = 7.9 Hz), 119.1, 114.4 (d, *J* = 21.1 Hz), 75.6, 62.7, 37.3, 33.9, 29.3, 27.3, 26.7, 26.5, 26.2.

^{19}F NMR (471 MHz, $CDCl_3$) −116.8 (s).

IR ν_{max} (neat) 3464 (br), 2924 (s), 2851 (m), 1602 (m), 1508 (s).

HRMS (EI^+) m/z: Calcd for $C_{17}H_{23}OF$ 262.1728; Found 262.1735.

(2*SR*,3*RS*)-3-Cyclohexyl-2-(4-bromophenyl)pent-4-en-2-ol **3m**

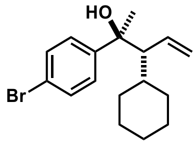

According to general procedure A, cyclohexylallene (72 µL, 0.5 mmol), 4'-bromoacetophenone (99 mg, 0.5 mmol), HBpin (87 µL, 0.6 mmol), [H-*B*-9-BBN]$_2$ (6 mg, 0.025 mmol) were reacted in *n*-hexane (1 mL) and heated under reflux for 16 hours. The crude reaction mixture was quenched (SiO_2) and purified by flash column chromatography (CombiFlash Isco NextGen300+, 12 g SiO_2, 50 mm Ø, petroleum ether/ ethyl acetate 10:1) to give the *alcohol* as a colourless oil (96 mg, 0.30 mmol, 59% yield, *anti:syn* >95:5).

^{1}H NMR (500 MHz, $CDCl_3$) 7.50 – 7.42 (m, 2H), 7.37 – 7.29 (m, 2H), 5.77 (dt, *J* = 17.0, 10.3 Hz, 1H), 5.16 (dd, *J* = 10.1, 2.3 Hz, 1H), 4.99 (ddd, *J* = 17.0, 2.3, 0.7 Hz, 1H), 2.11 (dd, *J* = 10.4, 2.5 Hz, 1H), 1.99 (s, 1H), 1.57 (s, 3H), 1.57 (s, 1H), 1.69 – 1.44 (m, 3H), 1.35 – 0.82 (m, 7H).

^{13}C NMR (126 MHz, $CDCl_3$) 146.7, 136.3, 131.3, 128.1, 121.0, 119.8, 76.3, 62.9, 37.8, 34.5, 29.8, 27.9, 27.3, 27.0, 26.7.

IR ν_{max} (neat) 3463 (br), 2922 (s), 2850 (m), 1590 (w), 1484 (m).

HRMS (EI^+) m/z: Calcd for $C_{17}H_{23}OBrNa$ 345.0825; Found 345.0816.

(2*SR*,3*RS*)-3-Cyclohexyl-2-(4-(trifluoromethyl)phenyl)pent-4-en-2-ol **3n**

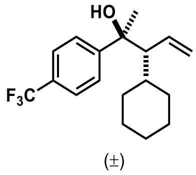

According to general procedure A, cyclohexylallene (72 μL, 0.5 mmol), 4'-(trifluoromethyl)acetophenone (94 mg, 0.5 mmol), HBpin (87 μL, 0.6 mmol), [H-*B*-9-BBN]$_2$ (6 mg, 0.025 mmol) were reacted in *n*-hexane (1 mL) and heated under reflux for 16 hours. The crude reaction mixture was quenched (SiO_2) and purified by flash column chromatography (CombiFlash Isco NextGen300+, 12 g SiO_2, 50 mm Ø, petroleum ether/ ethyl acetate 10:1) to give the *alcohol* as a colourless oil (138 mg, 0.44 mmol, 88% yield, *anti:syn* 94:6).

^{1}H NMR (500 MHz, $CDCl_3$) 7.62 – 7.52 (m, 4H), 5.75 (dt, *J* = 17.0, 10.3 Hz, 1H), 5.13 (dd, *J* = 10.2, 2.2 Hz, 1H), 4.94 (ddd, *J* = 17.0, 2.2, 0.7 Hz, 1H), 2.13 (dd, *J* = 10.4, 2.5 Hz, 1H), 1.97 (s, 1H), 1.66 – 1.45 (m, 9H), 1.31 – 0.95 (m, 7H), 0.88 (td, *J* = 12.4, 3.3 Hz, 1H).

^{13}C NMR (126 MHz, $CDCl_3$) 151.3, 135.6, 128.8 (q, *J* = 32.3 Hz), 126.1, 124.7 (q, *J* = 3.8 Hz), 124.3 (q, *J* = 271.8 Hz), 119.5, 76.1, 62.4, 37.4, 34.0, 29.4, 27.6, 26.8, 26.6, 26.2.

^{19}F NMR (376 MHz, $CDCl_3$) −62.3 (s).

IR ν_{max} (neat) 3469 (br), 2925 (m), 2852 (w), 1619 (w), 1451 (w).

HRMS (EI^+) m/z: Calcd for $C_{18}H_{23}OF_3Na$ 335.1593; Found 335.1593.

(2*SR*,3*RS*)-3-Cyclohexyl-2-(4-methoxyphenyl)pent-4-en-2-ol **3o**

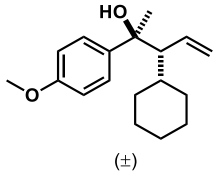

According to general procedure A, cyclohexylallene (72 µL, 0.5 mmol), 4'-methoxyacetophenone (75 mg, 0.5 mmol), HBpin (87 µL, 0.6 mmol), [H-*B*-9-BBN]$_2$ (6 mg, 0.025 mmol) were reacted in *n*-hexane (1 mL) and heated under reflux for 16 hours. The crude reaction mixture was quenched (SiO_2) and purified by flash column chromatography (CombiFlash Isco NextGen300+, 12 g SiO_2, 50 mm Ø, petroleum ether/ ethyl acetate 10:1) to give the *alcohol* as a colourless oil (69 mg, 0.25 mmol, 50% yield, *anti:syn* >95:5).

^{1}H NMR (500 MHz, $CDCl_3$) 7.43 – 7.34 (m, 2H), 6.93 – 6.85 (m, 2H), 5.80 (dt, *J* = 17.0, 10.3 Hz, 1H), 5.17 (dd, *J* = 10.1, 2.4 Hz, 1H), 5.03 (ddd, *J* = 17.0, 2.4, 0.6 Hz, 1H), 3.83 (s, 3H), 2.14 (dd, *J* = 10.4, 2.5 Hz, 1H), 1.94 (s, 1H), 1.66 – 1.48 (m, 8H), 1.48 – 1.41 (m, 1H), 1.28 (dtt, *J* = 10.8, 3.3, 1.8 Hz, 1H), 1.24 – 0.95 (m, 4H), 0.86 (qd, *J* = 12.5, 3.3 Hz, 1H).

^{13}C NMR (126 MHz, $CDCl_3$) 158.7, 139.6, 136.9, 127.3, 119.3, 113.5, 76.1, 63.2, 55.6, 37.8, 34.5, 29.8, 27.8, 27.3, 27.0, 26.7.

IR ν_{max} (neat) 3480 (br), 2922 (s), 2850 (m), 1612 (m), 1512 (s).

HRMS (EI$^+$) m/z: Calcd for $C_{17}H_{23}N$ 274.1927; Found 274.1917.

(2*SR*,3*RS*)-3-Cyclohexyl-2-(3-methoxyphenyl)pent-4-en-2-ol **3p**

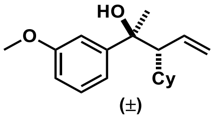

According to general procedure A, cyclohexylallene (72 µL, 0.5 mmol), 3-methoxyacetophenone (69 µL, 0.5 mmol), HBpin (87 µL, 0.6 mmol), [H-*B*-9-BBN]$_2$ (6 mg, 0.025 mmol) were reacted in *n*-hexane (1 mL) and heated under reflux for 16 hours. The crude reaction mixture was quenched (SiO_2) and purified by flash column chromatography (CombiFlash Isco NextGen300+, 12 g SiO_2, 50 mm Ø, petroleum ether/ ethyl acetate 10:1) to give the *alcohol* as a colourless oil (78 mg, 0.28 mmol, 57% yield, *anti:syn* >95:5).

^{1}H NMR (600 MHz, $CDCl_3$) 7.26 (t, *J* = 8.0 Hz, 1H), 7.07 – 7.00 (m, 2H), 6.80 (dd, *J* = 8.2, 2.6 Hz, 1H), 5.81 (dt, *J* = 16.9, 10.3 Hz, 1H), 5.15 (dd, *J* = 10.2, 2.3 Hz, 1H), 5.00 (dd, *J* = 17.0, 2.4 Hz, 1H), 3.84 (s, 3H), 2.16 (dd, *J* = 10.5, 2.4 Hz, 1H), 1.95 (s, 1H), 1.67 – 1.54 (m, 6H), 1.46 (dq, *J* = 12.9, 2.3, 1.9 Hz, 1H), 1.37 – 0.79 (m, 7H).

^{13}C NMR (126 MHz, $CDCl_3$) 159.5, 149.0, 136.4, 128.9, 119.0, 118.4, 112.0, 111.9, 76.2, 62.6, 55.4, 37.5, 34.2, 29.5, 27.8, 27.0, 26.8, 26.5.

IR ν_{max} (neat) 3484 (br), 3072 (w), 2922 (s), 2849 (m), 1600 (m), 1582 (m), 1488 (m), 1449 (m), 1432 (m), 1288 (m).

HRMS (EI^+) m/z: Calcd for $C_{18}H_{26}O_2$ 274.1927; Found 27.19337.

(2*SR*,3*RS*)-3-Cyclohexyl-2-(2-methoxyphenyl)pent-4-en-2-ol **3q**

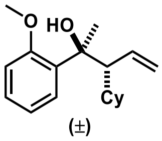

According to general procedure A, cyclohexylallene (72 µL, 0.5 mmol), 2-methoxyacetophenone (69 µL, 0.5 mmol), HBpin (87 µL, 0.6 mmol), [H-*B*-9-BBN]$_2$ (6 mg, 0.025 mmol) were reacted in *n*-hexane (1 mL) and heated under reflux for 16 hours. The crude reaction mixture was quenched (SiO_2) and purified by flash column chromatography (CombiFlash Isco NextGen300+, 12 g SiO_2, 50 mm Ø, petroleum ether/ ethyl acetate 10:1) to give the alcohol as a colourless oil (52 mg, 0.19 mmol, 38% yield, *anti:syn* >95:5).

^{1}H NMR (500 MHz, $CDCl_3$) 7.30 (dd, *J* = 7.8, 1.7 Hz, 1H), 7.24 (ddd, *J* = 8.1, 7.3, 1.7 Hz, 1H), 6.96 (td, *J* = 7.6, 1.2 Hz, 1H), 6.91 (dd, *J* = 8.2, 1.1 Hz, 1H), 5.84 (dt, *J* = 17.0, 10.3 Hz, 1H), 5.07 (dd, *J* = 10.1, 2.5 Hz, 1H), 4.90 (ddd, *J* = 17.0, 2.5, 0.7 Hz, 1H), 4.17 (s, 1H), 3.90 (s, 3H), 2.50 (dd, *J* = 10.4, 2.5 Hz, 1H), 1.77 – 1.61 (m, 4H), 1.61 – 1.40 (m, 3H), 1.29 – 0.89 (m, 7H).

^{13}C NMR (126 MHz, $CDCl_3$) 157.1, 137.1, 135.1, 128.2, 127.8, 120.9, 117.4, 111.6, 77.8, 60.1, 55.5, 38.3, 34.3, 29.8, 27.1, 26.9, 26.5, 25.2.

Data were in accordance with those previously reported.[5]

4-Cyclohexyl-3-ethylhex-5-en-3-ol **3r**

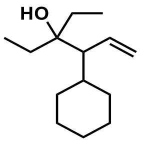

According to general procedure A, cyclohexylallene (72 µL, 0.5 mmol), pentan-3-one (53 µL, 0.5 mmol), HBpin (87 µL, 0.6 mmol), [H-*B*-9-BBN]$_2$ (6 mg, 0.025 mmol) were reacted in *n*-hexane (1 mL) and heated under reflux for 16 hours. The crude reaction mixture was quenched (SiO_2) and purified by flash column chromatography (CombiFlash Isco NextGen300+, 12 g SiO_2, 50 mm Ø, petroleum ether/ ethyl acetate 10:1) to give the *alcohol* as a colourless oil (74 mg, 0.35 mmol, 70% yield).

^{1}H NMR (600 MHz, $CDCl_3$) 5.83 (dt, *J* = 17.1, 10.3 Hz, 1H), 5.14 (dd, *J* = 10.2, 2.5 Hz, 1H), 4.97 (ddd, *J* = 17.1, 2.5, 0.7 Hz, 1H), 1.93 – 1.85 (m, 2H), 1.74 – 1.65 (m, 2H), 1.66 – 1.59 (m, 2H), 1.58 – 1.52 (m, 2H), 1.51 (qd, *J* = 7.5, 1.6 Hz, 2H), 1.48 – 1.38 (m, 1H), 1.35 – 1.24 (m, 2H), 1.22 – 1.15 (m, 2H), 1.12 – 0.96 (m, 2H), 0.85 (t, *J* = 7.5 Hz, 3H), 0.81 (t, *J* = 7.5 Hz, 3H).

^{13}C NMR (126 MHz, $CDCl_3$) 136.7, 118.1, 76.6, 56.9, 37.3, 34.2, 29.9, 29.5, 28.8, 27.2, 27.0, 26.6, 8.0, 7.8.

IR ν_{max} (neat) 3484 (br), 2967 (m), 2923 (s), 1636 (w), 1451 (m).

HRMS (EI^+) m/z: Calcd for $C_{14}H_{26}ONa$ 233.1876; Found 233.1875.

(2*SR*,3*RS*)-3-Cyclohexyl-2-benzylpent-4-en-2-ol **3s**

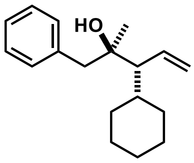

(±)

According to general procedure A, cyclohexylallene (72 μL, 0.5 mmol), 2-phenylacetone (66 μL, 0.5 mmol), HBpin (87 μL, 0.6 mmol), [H-*B*-9-BBN]$_2$ (6 mg, 0.025 mmol) were reacted in *n*-hexane (1 mL) and heated under reflux for 16 hours. The crude reaction mixture was quenched (SiO_2) and purified by flash column chromatography (CombiFlash Isco NextGen300+, 12 g SiO_2, 50 mm Ø, petroleum ether/ ethyl acetate 10:1) to give the *alcohol* as a colourless oil (98 mg, 0.38 mmol, 76% yield, *anti:syn* >95:5).

^{1}H NMR (500 MHz, $CDCl_3$) 7.37 – 7.30 (m, 2H), 7.27 (ddd, *J* = 9.2, 3.7, 2.5 Hz, 3H), 5.94 (dt, *J* = 17.0, 10.3 Hz, 1H), 5.25 (dd, *J* = 10.2, 2.5 Hz, 1H), 5.07 (dd, *J* = 17.1, 2.4 Hz, 1H), 2.95 (d, *J* = 13.4 Hz, 1H), 2.74 (d, *J* = 13.4 Hz, 1H), 2.00 – 1.89 (m, 2H), 1.79 (td, *J* = 10.4, 8.9, 4.3 Hz, 2H), 1.76 (dd, *J* = 3.6, 1.8 Hz, 1H), 1.69 (ddq, *J* = 12.4, 3.5, 1.8 Hz, 1H), 1.51 (ddt, *J* = 12.5, 3.5, 1.8 Hz, 1H), 1.44 – 1.18 (m, 4H), 1.15 (s, 3H), 1.14 – 1.04 (m, 2H).

^{13}C NMR (126 MHz, $CDCl_3$) 137.6, 136.4, 130.7, 127.9, 126.2, 118.6, 74.2, 60.0, 46.2, 37.8, 33.9, 29.5, 26.9, 26.7, 26.3, 25.4.

IR ν_{max} (neat) 3577 (br), 2922 (s), 2850 (m), 1636 (w), 1495 (w), 1450 (m).

HRMS (EI$^+$) m/z: Calcd for $C_{18}H_{26}O$ 258.1978; Found 258.1966.

(3*SR*,4*RS*)-3-Cyclohexyl-4-methylnon-1,7-dien-4-ol **3t**

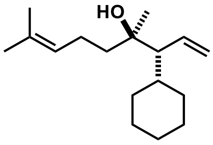

(±)

According to general procedure A, cyclohexylallene (72 µL, 0.5 mmol), 4'-fluoroacetophenone (73 mg, 0.5 mmol), HBpin (87 µL, 0.6 mmol), $[H\text{-}B\text{-}9\text{-}BBN]_2$ (6 mg, 0.025 mmol) were reacted in *n*-hexane (1 mL) and heated under reflux for 16 hours. The crude reaction mixture was quenched (SiO_2) and purified by flash column chromatography (CombiFlash Isco NextGen300+, 12 g SiO_2, 50 mm Ø, petroleum ether/ ethyl acetate 10:1) to give the *alcohol* as a colourless oil (116 mg, 0.46 mmol, 93% yield, *anti:syn* >95:5).

^{1}H NMR (500 MHz, $CDCl_3$ 5.81 (dt, *J* = 17.0, 10.3 Hz, 1H), 5.17 (dd, *J* = 10.1, 2.5 Hz, 1H), 5.11 (ddt, *J* = 8.6, 7.2, 1.6 Hz, 1H), 5.06 – 4.93 (m, 1H), 2.12 – 2.00 (m, 2H), 1.88 – 1.78 (m, 2H), 1.73 – 1.66 (m, 5H), 1.64 – 1.42 (m, 9H), 1.35 – 0.93 (m, 9H).

^{13}C NMR (126 MHz, $CDCl_3$) 136.3, 131.4, 124.5, 118.6, 74.08, 59.9, 40.5, 37.6, 34.1, 29.6, 26.9, 26.7, 26.3, 25.6, 25.1, 22.2, 17.5.

HRMS (EI^+) m/z: Calcd for $C_{17}H_{30}O$ 250.2291; Found 250.227.9.

IR ν_{max} (neat) 3563 (br), 3082 (w), 2929 (w), 1637 (w), 1604 (w).

(3*RS*,4*RS*)- 4-Cyclohexyl-3-methyl-1-phenylhex-1-yne-5-en-3-ol **3u**

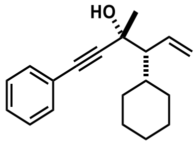

(±)

According to general procedure A, cyclohexylallene (72 μL, 0.5 mmol), 4-phenyl-3-butyn-2-one (72 μL, 0.5 mmol), HBpin (87 μL, 0.6 mmol), [H-*B*-9-BBN]$_2$ (6 mg, 0.025 mmol) were reacted in *n*-hexane (1 mL) and heated under reflux for 16 hours. The crude reaction mixture was quenched (SiO_2) and purified by flash column chromatography (CombiFlash Isco NextGen300+, 12 g SiO_2, 50 mm Ø, petroleum ether/ ethyl acetate 10:1) to give the *alcohol* as a colourless oil (88 mg, 0.33 mmol, 66% yield, *syn:anti* 67:33).

^{1}H NMR (500 MHz, $CDCl_3$) **Major (*syn*):** δ 7.46 – 7.40 (m, 2H), 7.36 – 7.30 (m, 3H), 5.88 (dt, *J* = 17.0, 10.2 Hz, 1H), 5.21 (dd, *J* = 10.2, 2.3 Hz, 1H), 5.11 (ddd, *J* = 17.0, 2.2, 0.7 Hz, 1H), 2.22 – 2.16 (m, 1H), 2.14 (s, 1H), 2.10 (dd, *J* = 10.3, 3.2 Hz, 1H), 1.94 (tq, *J* = 11.9, 3.3 Hz, 1H), 1.79 – 1.72 (m, 2H), 1.70 – 1.61 (m, 2H), 1.56 (s, 3H), 1.42 –1.02 (m, 5H) **Minor (*anti*):** 7.46 – 7.39 (m, 2H), 7.33 (tt, *J* = 3.7, 2.5 Hz, 3H), 6.04 (dt, *J* = 17.1, 10.2 Hz, 1H), 5.34 (dd, *J* = 10.2, 2.3 Hz, 1H), 5.19 (ddd, *J* = 17.0, 2.3, 0.7 Hz, 1H), 2.36 (s, 1H), 2.23 – 2.16 (m, 1H), 2.04 (dd, *J* = 10.3, 3.0 Hz, 1H), 1.87 – 1.72 (m, 3H), 1.67 (m, 1H), 1.40 – 1.04 (m, 5H).

^{13}C NMR (126 MHz, $CDCl_3$) **Major (*syn*)** 136.5, 135.9, 131.5, 128.3, 120.2, 118.5, 92.8, 84.7, 69.5, 62.2, 38.8, 33.8, 28.8, 28.2, 26.9, 26.8, 26.4 **Minor (*anti*)** 144.6, 136.3, 131.6, 128.2, 127.7, 126.2, 92.8, 78.7, 69.8, 61.6, 38.0, 33.7, 30.1, 29.3, 27.0, 26.7, 26.5.

IR ν_{max} (neat) 3436 (br), 2922 (s), 2851 (m), 1597 (w), 1489 (m).

HRMS (EI^+) m/z: Calcd for $C_{19}H_{24}O$ 268.1822; Found 268.1837.

(2*SR*,3*RS*)-3-Cyclohexyl-2-adamantylpent-4-en-2-ol **3v**

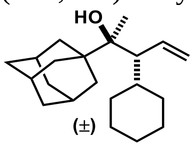

According to general procedure A, cyclohexylallene (72 μL, 0.5 mmol), 1-acetyladamantane (179 mg, 0.5 mmol), HBpin (87 μL, 0.6 mmol), $[H\text{-}B\text{-}9\text{-}BBN]_2$ (6 mg, 0.025 mmol) were reacted in *n*-hexane (1 mL) and heated under reflux for 16 hours. The crude reaction mixture was quenched (SiO_2) and purified by flash column chromatography (CombiFlash Isco NextGen300+, 12 g SiO_2, 50 mm Ø, petroleum ether/ ethyl acetate 10:1) to give the *alcohol* as a colourless oil (130 mg, 0.43 mmol, 86% yield, *anti:syn* >95:5).

^{1}H NMR (500 MHz, $CDCl_3$) δ 5.88 (dt, *J* = 17.0, 10.2 Hz, 1H), 5.16 (dd, *J* = 10.0, 2.4 Hz, 1H), 4.98 (ddd, *J* = 17.0, 2.5, 0.6 Hz, 1H), 2.28 – 2.22 (m, 1H), 2.00 (p, *J* = 3.3 Hz, 3H), 1.87 – 1.79 (m, 1H), 1.68 (s, 8H), 1.77 – 1.57 (m, 15H), 1.53 – 1.47 (m, 1H), 1.47 (s, 1H), 1.31 – 1.18 (m, 2H), 1.17 (s, 3H), 1.14 – 0.81 (m, 4H).

^{13}C NMR (126 MHz, $CDCl_3$) 138.1, 118.4, 77.0, 56.2, 41.5, 39.5, 37.6, 37.2, 34.2, 30.2, 29.2, 27.6, 27.3, 26.8, 19.3.

IR ν_{max} (neat) 3563 (w), 3484 (w), 2901 (s), 2845 (m), 1699 (w), 1634 (w).

HRMS (EI^+) m/z: Calcd for $C_{21}H_{34}ONa$ 325.2502; Found 325.2497.

(2*SR*,3*RS*)-Methyl 3-cyclohexyl-2-hydroxy-2-methylpent-4-enoate **3w**

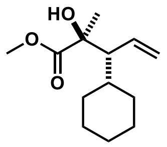

According to general procedure A, cyclohexylallene (72 µL, 0.5 mmol), pyruvic acid methyl ester (51 µL, 0.5 mmol), HBpin (87 µL, 0.6 mmol), [H-*B*-9-BBN]$_2$ (6 mg, 0.025 mmol) were reacted in *n*-hexane (1 mL) and heated under reflux for 16 hours. The crude reaction mixture was quenched (SiO_2) and purified by flash column chromatography (CombiFlash Isco NextGen300+, 12 g SiO_2, 50 mm Ø, petroleum ether/ ethyl acetate 10:1) to give the *alcohol* as a colourless oil (91 mg, 0.40 mmol, 80% yield, *anti:syn* 60:40).

^{1}H NMR (500 MHz, $CDCl_3$) 5.82 (dt, *anti* + *syn*, *J* = 17.1, 10.2 Hz, 1H), 5.21 (dd, *syn*, *J* = 10.1, 2.4 Hz, 0.39H), 5.08 (dd, *anti*, *J* = 10.2, 2.4 Hz, 0.59H), 5.03 (dd, *syn*, *J* = 17.1, 2.4 Hz, 0.42H), 4.95 (dd, *anti*, *J* = 17.1, 2.4, 0.65), 3.80 (s, *syn*, 1.19 H), 3.74 (s, *anti*, 1.80H), 2.18-2.10 (*anti* + *syn,* m, 1H), 1.90 - 1.84 (*anti* + *syn,* m, 1H), 1.80 – 1.48 (*anti* + *syn,* m, 5H), 1.42 (*anti* + *syn,* s, 3H), 1.33 - 0.9 (*anti* + *syn,* m, 7H).

^{13}C NMR (126 MHz, $CDCl_3$) *anti* + *syn*: 178.3 (*syn*), 177.7 (*anti*), 135.7 (*anti*), 135.2 (*syn*), 118.8 (*syn*), 118.0 (*anti*), 77.3 (*anti*), 77.05 (*syn*), 57.9 (*anti*), 57.6 (*syn*), 52.6 (*anti*), 52.6 (*syn*), 39.4 (*anti* + *syn*), 36.7 (*anti* + *syn*), 33.5 (*anti* + *syn*), 32.9 (*anti* + *syn*), 29.9 (*anti* + *syn*), 28.8 (*anti* + *syn*), 26.9 (*anti*), 26.8 (*syn*), 26.6 (*anti*), 26.6 (*syn*), 26.3 (*anti*), 26.3 (*syn*), 24.6 (*anti* + *syn*).

IR ν_{max} (neat) 3521 (br), 2922 (s), 2851 (m), 1729 (s), 1449 (m).

HRMS (EI^+) m/z: Calcd for $C_{13}H_{22}O_3$ 226.1564; Found 226.1572.

(2*SR*,3*RS*)-1-(3-Cyclohexylprop-1-ene)indanol **3x**

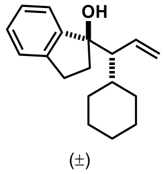

According to general procedure A, cyclohexylallene (72 μL, 0.5 mmol), 1-indanone (66 mg, 0.5 mmol), HBpin (87 μL, 0.6 mmol), [H-*B*-9-BBN]$_2$ (6 mg, 0.025 mmol) were reacted in *n*-hexane (1 mL) and heated under reflux for 16 hours. The crude reaction mixture was quenched (SiO_2) and purified by flash column chromatography (CombiFlash Isco NextGen300+, 12 g SiO_2, 50 mm Ø, petroleum ether/ ethyl acetate 10:1) to give the *alcohol* as a colourless oil (83 mg, 0.32 mmol, 65% yield, *anti:syn* 66:34).

^{1}H NMR (500 MHz, $CDCl_3$) 7.34 – 7.28 (*anti* + *syn*, m, 1H), 7.26 – 7.18 (*anti* + *syn*, m, 3H), 5.96 – 5.80 (*anti* + *syn*, m, 1H), 5.27 (*anti*, dd, *J* = 10.1, 2.3 Hz, 0.76H), 5.16 (*anti*, dd, *J* = 17.0, 2.2 Hz, 0.76H), 5.12 (*syn*, dd, *J* = 10.2, 2.3 Hz, 0.24H), 4.98 (*syn*, dd, *J* = 17.0, 2.3 Hz, 0.25), 3.03 (*anti*, ddd, *J* = 16.2, 8.8, 5.1 Hz, 0.76H), 2.96 (*syn*, ddd, *J* = 16.1, 8.8, 3.7 Hz, 0.27H), 2.83 (*anti*, ddd, *J* = 16.0, 8.5, 5.6 Hz, 1H), 2.55 – 2.43 (*anti* + *syn*, m, 1H), 2.36 (*anti*, dd, *J* = 10.4, 3.0 Hz, 0.75H), 2.29 (*syn*, dd, *J* = 10.3, 2.7 Hz, 0.25H), 2.11-2.00 (*anti* + *syn*, m, 1H), 1.80 - 1.74 (*anti* + *syn*, m, 1H), 1.71 – 1.51 (*anti* + *syn*, m, 4H), 1.38 – 0.86 (*anti* + *syn*, m, 8H).

^{13}C NMR (126 MHz, $CDCl_3$) **Major (*anti*)** 146.5, 143.7, 136.8, 128.2, 126.5, 124.8, 123.6, 119.4, 85.2, 59.6, 38.4, 37.6, 34.0, 30.5, 29.9, 26.8, 26.6, 26.3 **Minor (*syn*)** 147.3, 143.2, 136.1, 128.0 126.4, 124.9, 124.2, 118.8, 85.6, 59.0, 39.4, 37.7, 33.8, 30.0, 29.2, 26.9, 26.8, 26.4.

IR ν_{max} (neat) 3424 (br), 2921 (s), 2850 (m), 1636 (w), 1449 (m).

HRMS (EI$^+$) m/z: Calcd for $C_{19}H_{24}O$ 256.1822; Found 256.1818.

(2*SR*,3*RS*)-2-(Fur-2-yl)-3-cyclohexylpent-4-en-2-ol **3y**

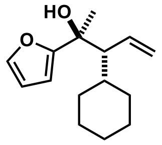

(±)

According to general procedure A, cyclohexylallene (72 µL, 0.5 mmol), 2-acetylfuran (55 mg, 0.5 mmol), HBpin (87 µL, 0.6 mmol), [H-*B*-9-BBN]$_2$ (6 mg, 0.025 mmol) were reacted in *n*-hexane (1 mL) and heated under reflux for 16 hours. The crude reaction mixture was quenched (SiO_2) and purified by flash column chromatography (CombiFlash Isco NextGen300+, 12 g SiO_2, 50 mm Ø, petroleum ether/ ethyl acetate 10:1) to give the *alcohol* as a colourless oil (102 mg, 0.44 mmol, 87% yield, *anti:syn* >95:5).

^{1}H NMR (500 MHz, $CDCl_3$) 7.37 (dd, *J* = 1.8, 0.9 Hz, 1H), 6.34 (dd, *J* = 3.2, 1.8 Hz, 1H), 6.23 (dd, *J* = 3.2, 0.9 Hz, 1H), 5.84 (dt, *J* = 17.0, 10.3 Hz, 1H), 5.21 (dd, *J* = 10.1, 2.3 Hz, 1H), 5.11 (ddd, *J* = 17.0, 2.3, 0.7 Hz, 1H), 2.36 (dd, *J* = 10.5, 3.0 Hz, 1H), 2.26 (s, 1H), 1.68 – 1.54 (m, 5H), 1.46 (m, 1H), 1.41 – 1.34 (m, 3H), 1.07 (m, 3H), 0.96 – 0.81 (m, 2H).

^{13}C NMR (126 MHz, $CDCl_3$) 159.0, 141.2, 136.1, 119.1, 110.2, 105.5, 73.0, 60.6, 37.8, 33.4, 29.5, 26.8, 26.6, 26.3, 25.0.

IR ν_{max} (neat) 3473 (br), 2922 (s), 1851 (m), 1635 (w), 1504 (w).

HRMS (EI$^+$) m/z: Calcd for $C_{15}H_{22}O_2$ 234.1614; Found 234.1617.

(2*SR*,3*RS*)-2-(Thiophen-2-yl)-3-cyclohexylpent-4-en-2-ol **3z**

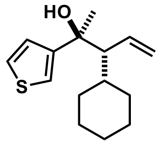

(±)

According to general procedure A, cyclohexylallene (72 μL, 0.5 mmol), 3-acetylthiophene (63 mg, 0.5 mmol), HBpin (87 μL, 0.6 mmol), [H-*B*-9-BBN]$_2$ (6 mg, 0.025 mmol) were reacted in *n*-hexane (1 mL) and heated under reflux for 16 hours. The crude reaction mixture was quenched (SiO_2) and purified by flash column chromatography (CombiFlash Isco NextGen300+, 12 g SiO_2, 50 mm Ø, petroleum ether/ ethyl acetate 10:1) to give the *alcohol* as a colourless oil (110 mg, 0.44 mmol, 88% yield, *anti:syn* >95:5).

^{1}H NMR (500 MHz, $CDCl_3$) 7.25 (dd, *J* = 4.6, 2.5 Hz, 1H), 7.15 (dd, *J* = 3.0, 1.4 Hz, 1H), 7.07 (dd, *J* = 5.0, 1.4 Hz, 1H), 5.78 (dt, *J* = 16.9, 10.3 Hz, 1H), 5.16 (dd, *J* = 10.1, 2.3 Hz, 1H), 5.04 (ddd, *J* = 17.0, 2.4, 0.7 Hz, 1H), 2.13 (dd, *J* = 10.4, 2.5 Hz, 1H), 2.00 (s, 1H), 1.69 – 1.56 (m, 3H), 1.55 (s, 3H), 1.40 – 1.28 (m, 2H), 1.28 – 1.13 (m, 1H), 1.15 – 0.94 (m, 4H), 0.83 (qd, *J* = 12.5, 3.4 Hz, 1H).

^{13}C NMR (126 MHz, $CDCl_3$) 148.8, 136.2, 126.2, 125.0, 120.1, 118.8, 74.9, 62.3, 37.5, 33.9, 29.2, 27.7, 26.8, 26.5, 26.2.

IR ν_{max} (neat) 3456 (br), 2922 (s), 2850 (m), 1634 (w), 1449 (m).

HRMS (EI^+) m/z: Calcd for $C_{15}H_{22}OS$ 250.1386; Found 250.1386.

(2*SR*,3*RS*)-3-Cyclohexyl-2-(benzo[d][1,3]dioxol-6-yl)pent-4-en-2-ol **3aa**

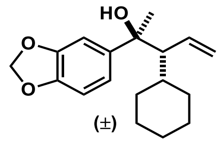

According to general procedure A, cyclohexylallene (72 µL, 0.5 mmol), 1-(benzo[d][1,3]dioxol-6-yl)ethanone (82 mg, 0.5 mmol), HBpin (87 µL, 0.6 mmol), [H-*B*-9-BBN]$_2$ (6 mg, 0.025 mmol) were reacted in *n*-hexane (1 mL) and heated under reflux for 16 hours. The crude reaction mixture was quenched (SiO_2) and purified by flash column chromatography (CombiFlash Isco NextGen300+, 12 g SiO_2, 50 mm Ø, petroleum ether/ ethyl acetate 10:1) to give the *alcohol* as a colourless oil (85 mg, 0.29 mmol, 59% yield *anti:syn* >95:5).

^{1}H NMR (500 MHz, $CDCl_3$) δ 6.96 (d, *J* = 1.9 Hz, 1H), 6.89 (dd, *J* = 8.2, 1.9 Hz, 1H), 6.75 (d, *J* = 8.2 Hz, 1H), 5.95 (q, *J* = 1.5 Hz, 2H), 5.78 (dt, *J* = 16.9, 10.3 Hz, 1H), 5.16 (dd, *J* = 10.1, 2.4 Hz, 1H), 5.01 (ddd, *J* = 17.0, 2.4, 0.7 Hz, 1H), 2.09 (dd, *J* = 10.4, 2.4 Hz, 1H), 1.92 (s, 1H), 1.64 – 1.54 (m, 3H), 1.54 (s, 3H), 1.52 – 1.44 (m, 2H), 1.31 – 0.94 (m, 5H), 0.92 – 0.80 (m, 1H).

^{13}C NMR (126 MHz, $CDCl_3$) 147.3, 146.0, 141.2, 136.2, 119.0, 118.8, 107.3, 106.7, 100.8, 75.8, 62.7, 37.3, 33.9, 29.3, 27.3, 26.8, 26.5, 26.2.

IR ν_{max} (neat) 3473 (br), 2922 (m), 2851 (m), 1503 (m), 1486 (s).

HRMS (EI^+) m/z: Calcd for $C_{18}H_{24}O_3$ 288.1720; Found 288.1736.

(2*SR*,3*RS*)-3-Cyclohexyl-2-ferrocenylpent-4-en-2-ol **3ab**

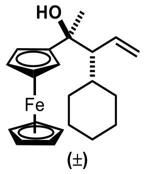

According to general procedure A, cyclohexylallene (72 µL, 0.5 mmol), acetylferrocene (114 mg, 0.5 mmol), HBpin (87 µL, 0.6 mmol), [H-*B*-9-BBN]$_2$ (6 mg, 0.025 mmol) were reacted in *n*-hexane (1 mL) and heated under reflux for 16 hours. The crude reaction mixture was quenched (SiO_2) and purified by flash column chromatography (CombiFlash Isco NextGen300+, 12 g SiO_2, 50 mm Ø, petroleum ether/ ethyl acetate 10:1) to give the *alcohol* as a colourless oil (127 mg, 0.33 mmol, 66% yield, *anti:syn* >95:5).

^{1}H NMR (500 MHz, $CDCl_3$) 5.68 (dt, *J* = 17.0, 10.3 Hz, 1H), 5.08 (dd, *J* = 10.1, 2.4 Hz, 1H), 4.95 (dd, *J* = 2.4, 0.6 Hz, 1H), 4.34 (dt, *J* = 2.6, 1.4 Hz, 1H), 4.23 (s, 5H), 4.21 (td, *J* = 2.5, 1.4 Hz, 1H), 4.16 (td, *J* = 2.4, 1.3 Hz, 1H), 4.07 (dt, *J* = 2.6, 1.4 Hz, 1H), 2.23 (s, 1H), 1.96 (dd, *J* = 10.5, 2.2 Hz, 2H), 1.67 – 1.45 (m, 9H), 1.41 – 1.28 (m, 2H), 1.38 – 1.14 (m, 4H), 1.14 – 0.89 (m, 3H), 0.77 (qd, *J* = 12.4, 3.3 Hz, 1H).

^{13}C NMR (126 MHz, $CDCl_3$) 137.0, 117.2, 98.4, 72.7, 68.2, 68.1, 67.5, 67.5, 65.7, 61.7, 37.3, 34.1, 29.4, 27.7, 26.8, 26.5, 26.3.

IR ν_{max} (neat) 3541 (m), 2928 (s), 2911 (s), 2848 (m), 1707 (w), 1656 (w).

HRMS (EI^+) m/z: Calcd for $C_{21}H_{28}OFe$ 352.1484; Found 352.1485.

3-Cylohexylprop-1-en-3-estradiol **3ac**

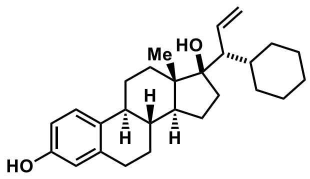

According to general procedure A, cyclohexylallene (72 μL, 0.5 mmol), estrone (135 mg, 0.5 mmol), HBpin (174 μL, 1.2 mmol), [H-*B*-9-BBN]$_2$ (6 mg, 0.025 mmol) were reacted in *n*-hexane (1 mL) and heated under reflux for 16 hours. The crude reaction mixture was quenched (SiO_2) and purified by flash column chromatography (CombiFlash Isco NextGen300+, 12 g SiO_2, 50 mm Ø, petroleum ether/ ethyl acetate 10:1) to give the *alcohol* as a colourless oil (154 mg, 0.39 mmol, 78% yield, *anti:syn* >95:5).

^{1}H NMR (500 MHz, $CDCl_3$) δ 7.17 (*anti* + *syn*, dd, *J* = 8.5, 1.1 Hz, 1H), 6.64 (*syn* +*anti*, dd, *J* = 8.4, 2.9 Hz, 1H), 6.58 (*anti* + *syn*, d, *J* = 2.9 Hz, 1H), 6.01 (*anti*, dt, *J* = 17.3, 10.2 Hz, 0.85H), 5.90 (*syn*, dt, *J* = 17.2, 10.3 Hz, 0.15H), 5.23 (*anti*, dd, *J* = 10.4, 2.3 Hz, 0.82H), 5.14 (*syn*, dd, *J* = 10.2, 2.4 Hz, 0.18H), 5.05 (*anti*, dd, *J* = 17.5, 2.3 Hz, 0.82H) 4.98 (*syn*, dd, *J* = 17.2, 2.4 Hz, 0.18H) 4.65 (*anti* + *syn*, br s, 1H) 2.92 – 2.79 (*anti* + *syn*, m, 2H), 2.35 (*anti* + *syn*, m, 2H), 2.21 – 2.13 (*anti* + *syn*, m, 2H), 1.98 – 0.96 (*anti* + *syn*, m, 26H), 0.92 (*anti*, s, 2.38H), 0.78 (*syn*, s, 0.74H).

^{13}C NMR (126 MHz, $CDCl_3$) **Major (*anti*):** 153.3, 138.3, 137.6, 132.8, 126.5, 118.2, 115.2, 112.7, 85.0, 55.4, 49.7, 48.8, 43.7, 40.0, 39.8, 34.8, 33.3, 33.2, 29.7, 28.7, 27.6, 27.1, 26.9, 26.5, 26.5, 24.9, 23.7, 15.5 **Minor (*syn*):** 153.2, 138.4, 137.2, 133.0, 126.5, 116.4, 112.6, 86.7, 75.1, 55.8, 49.6, 48.3, 43.4, 39.7, 39.1, 37.4, 33.6, 31.7, 30.0, 27.7, 27.0, 26.3, 24.6, 23.0, 14.5.

IR ν_{max} (neat) 3420 (br), 2922 (s), 2851 (m), 1613 (w), 1586 (w).

HRMS (EI^+) m/z: Calcd for $C_{27}H_{38}O_2Na$ 417.2764; Found 417.2745.

(2*SR*,3*SR*)-4-(2-(7-Methoxynaphthalene)-2-ethenylcyclohexyl-butan-2-ol **3ad**

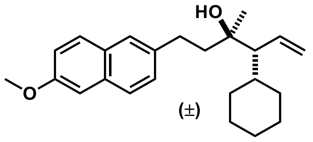

According to general procedure A, cyclohexylallene (72 μL, 0.5 mmol), nabumetone (114 mg, 0.5 mmol), HBpin (87 μL, 0.6 mmol), [H-*B*-9-BBN]$_2$ (6 mg, 0.025 mmol) were reacted in *n*-hexane (1 mL) and heated under reflux for 16 hours. The crude reaction mixture was quenched (SiO_2) and purified by flash column chromatography (CombiFlash Isco NextGen300+, 12 g SiO_2, 50 mm Ø, petroleum ether/ ethyl acetate 10:1) to give the *alcohol* as a colourless oil (97 mg, 0.28 mmol, 55% yield, *anti:syn* 94:6).

^{1}H NMR (600 MHz, $CDCl_3$) δ 7.71 (d, *J* = 8.6 Hz, 2H), 7.60 (s, 1H), 7.35 (d, *J* = 8.4 Hz, 1H), 7.16 (d, *J* = 13.6 Hz, 2H), 5.90 (dt, *J* = 17.0, 10.3 Hz, 1H), 5.27 (d, *J* = 10.1 Hz, 1H), 5.14 (d, *J* = 17.1 Hz, 1H), 3.94 (s, 3H), 2.86 (t, *J* = 7.9 Hz, 2H), 2.00 (d, *J* = 10.6 Hz, 1H), 1.91 (dtt, *J* = 25.4, 17.6, 9.5 Hz, 3H), 1.79 – 1.59 (m, 5H), 1.54 (d, *J* = 13.0 Hz, 1H), 1.35 (d, *J* = 12.4 Hz, 1H), 1.38 – 1.17 (m, 6H), 1.17 – 1.10 (m, 1H), 1.10 – 1.03 (m, 2H).

^{13}C NMR (151 MHz, $CDCl_3$) 157.2, 138.1, 136.3, 133.0, 129.2, 128.9, 127.9, 126.8, 126.2, 119.2, 118.7, 105.7, 74.1, 60.3, 55.3, 43.1, 37.8, 34.4, 30.1, 29.8, 27.0, 26.8, 26.4, 25.2.

IR ν_{max} (neat) 3323 (br), 2967 (m), 2932 (m), 2851 (m), 1634 (m), 1603 (s).

HRMS (EI^+) m/z: Calcd for $C_{24}H_{32}O_2$ 353.2397; Found 353.2397.

(5*SR*,6*RS*)-1-(6-Cyclohexyl-5-hydroxy-5-methyloct-7-enyl)-7-methylxanthine **3ae**

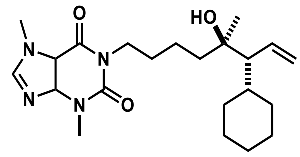

According to general procedure A, cyclohexylallene (72 μL, 0.5 mmol), pentoxifylline (139 mg, 0.5 mmol), HBpin (87 μL, 0.6 mmol), [H-*B*-9-BBN]$_2$ (6 mg, 0.025 mmol) were reacted in *n*-hexane (1 mL) and heated under reflux for 16 hours. The crude reaction mixture was quenched (SiO_2) and purified by flash column chromatography (CombiFlash Isco NextGen300+, 12 g SiO_2, 50 mm Ø, petroleum ether/ ethyl acetate 10:1) to give the *alcohol* as a colourless oil (183 mg, 0.45 mmol, 90% yield, *anti:syn* 93:7).

^{1}H NMR (500 MHz, $CDCl_3$) 7.51 (s, 1H), 5.82 (dt, *J* = 17.0, 10.3 Hz, 1H), 5.17 (dd, *J* = 10.1, 2.5 Hz, 1H), 5.02 (ddd, *J* = 17.0, 2.4, 0.6 Hz, 1H), 4.03 (ddd, *J* = 8.1, 6.8, 1.4 Hz, 2H), 3.99 (s, 3H), 3.58 (s, 3H), 1.88 – 1.81 (m, 2H), 1.75 – 1.49 (m, 9H), 1.48 – 1.36 (m, 3H), 1.36 – 1.18 (m, 4H), 1.18 – 1.11 (m, 5H), 1.14 – 0.95 (m, 2H).

^{13}C NMR (126 MHz, $CDCl_3$) 155.4, 151.5, 148.7, 141.4, 136.5, 118.6, 107.7, 74.1, 60.1, 41.2, 40.2, 37.7, 34.2, 33.6, 29.7, 28.4, 27.0, 26.8, 26.4, 25.1, 24.9, 20.7.

IR ν_{max} (neat) 3476 (br), 2924 (m), 2851 (w), 1702 (m), 1699 (m, 1656 (s), 1650 (s), 1550 (m).

HRMS (EI$^+$) m/z: Calcd for $C_{23}H_{35}O_3N_4$ 403.2704; Found 403.2720.

(2*SR*,3*RS*)-3-Cyclohexyl-2-haloperidiol **3af**

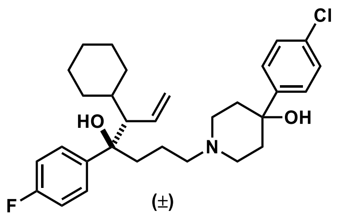

According to general procedure A, cyclohexylallene (72 µL, 0.5 mmol), haloperidol (187 mg, 0.5 mmol), HBpin (174 µL, 1.2 mmol), [H-*B*-9-BBN]$_2$ (6 mg, 0.025 mmol) were reacted in *n*-hexane (1 mL) and heated under reflux for 16 hours. The crude reaction mixture was quenched (SiO_2) and purified by flash column chromatography (CombiFlash Isco NextGen300+, 12 g SiO_2, 50 mm Ø, petroleum ether/ ethyl acetate 10:1) to give the *alcohol* as a colourless oil (227 mg, 0.45 mmol, 91% yield, *anti:syn* >95:5).

^{1}H NMR (500 MHz, $CDCl_3$) 7.69 – 7.52 (m, 1H), 7.51 – 7.44 (m, 2H), 7.43 – 7.34 (m, 4H), 6.98 (t, *J* = 8.8 Hz, 2H), 5.68 (dt, *J* = 17.0, 10.4 Hz, 1H), 5.00 (dd, *J* = 10.2, 2.5 Hz, 1H), 4.83 (dd, *J* = 17.0, 2.5 Hz, 1H), 2.77 (d, *J* = 11.2 Hz, 1H), 2.59 – 2.48 (m, 1H), 2.48 – 2.40 (m, 2H), 2.31 – 2.19 (m, 3H), 2.13 – 2.07 (m, 1H), 2.07 – 1.98 (m, 2H), 1.94 (dddd, *J* = 13.6, 6.6, 3.3, 1.6 Hz, 1H), 1.83 – 1.73 (m, 2H), 1.71 – 1.59 (m, 2H), 1.62 – 1.43 (m, 5H), 1.42 – 0.98 (m, 5H), 0.94 – 0.86 (m, 1H), 0.64 (qd, *J* = 12.4, 3.3 Hz, 1H).

^{13}C NMR (126 MHz, $CDCl_3$) 162.2 (d, *J* = 244.2 Hz), 146.5, 142.2 (d, *J* = 3.0 Hz), 137.3, 133.1, 128.6, 128.3 (d, *J* = 7.6 Hz), 126.2, 116.9, 114.1 (d, *J* = 20.8 Hz), 76.2, 70.8, 63.0, 60.4, 59.1, 50.3, 47.6, 40.7, 37.8, 37.7, 34.2, 29.0, 27.1, 26.6, 26.5, 21.1.

IR ν_{max} (neat) 3399 (br), 3070 (w), 2932 (s), 2848 (m), 1600 (m), 1505 (s).

HRMS (EI^+) m/z: Calcd for C30$H_{40}O_2$NClF 500.2726; Found 500.2745.

(2*R*,3*S*)-3-Cyclohexyl-2-phenylpent-4-en-2-ol **(2*R*,3*S*)-3a**

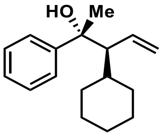

According to general procedure B, cyclohexylallene (72 μL, 0.5 mmol), acetophenone (59 μL, 0.5 mmol), HBpin (100 μL, 0.69 mmol), (*S*)-*B*-OMe-10-Ph-9-borabicyclo[3.3.2]decane (6 mg, 0.05 mmol) were reacted at 50 °C for 16 hours. The crude reaction mixture was quenched (SiO_2) and purified by flash column chromatography (CombiFlash Isco NextGen300+, 12 g SiO_2, 50 mm Ø, petroleum ether/ ethyl acetate 10:1) to give the *alcohol* as a colourless oil (87 mg, 0.36 mmol, 71% yield, *anti:syn* >95:5).

^{1}H NMR (600 MHz, $CDCl_3$) δ 7.46 – 7.41 (m, 2H), 7.35 – 7.29 (m, 2H), 7.25 – 7.20 (m, 1H), 5.78 (dt, *J* = 17.0, 10.3 Hz, 1H), 5.13 (dd, *J* = 10.2, 2.4 Hz, 1H), 4.98 (ddd, *J* = 17.0, 2.4, 0.7 Hz, 1H), 2.14 (dd, *J* = 10.5, 2.5 Hz, 1H), 1.93 (s, 1H), 1.59 (m, 5H), 1.45 – 0.77 (m, 9H).

^{13}C NMR (101 MHz, $CDCl_3$) 147.1, 136.3, 127.8, 126.6, 125.7, 118.9, 76.1, 62.6, 37.4, 34.0, 29.4, 27.5, 26.9, 26.6, 26.3.

Data were in accordance with those previously reported.[5]

HPLC: 89:11 *e.r.* [Chiralpak ODH (0.46 x 25 cm), particle size = 5 μm heptane/2-propanol = 95/5, ν = 1.0 mL min^{-1}, λ = 220 nm, t (major) = 6.279 minutes, t (minor) = 8.703 minutes].

$[\alpha]_D^{20}$ −30.0 (c 0.40, CH_2Cl_2).

(2*R*,3*S*)-3-Cyclohexyl-2-(4-fluorophenyl)pent-4-en-2-ol **(2*R*,3*S*)-3l**

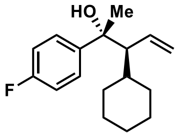

According to general procedure B, cyclohexylallene (72 µL, 0.5 mmol), 4-fluoroacetophenone (69 mg, 0.5 mmol), HBpin (100 µL, 0.69 mmol), (*S*)-*B*-OMe-10-Ph-9-borabicyclo[3.3.2]decane (6 mg, 0.05 mmol) were reacted at 50 °C for 16 hours. The crude reaction mixture was quenched (SiO_2) and purified by flash column chromatography (CombiFlash Isco NextGen300+, 12 g SiO_2, 50 mm Ø, petroleum ether/ ethyl acetate 10:1) to give the *alcohol* as a colourless oil (61 mg, 0.23 mmol, 46% yield, *anti:syn* >95:5).

^{1}H NMR (500 MHz, $CDCl_3$) 7.44 – 7.36 (m, 2H), 7.04 – 6.95 (m, 2H), 5.76 (dt, *J* = 17.0, 10.3 Hz, 1H), 5.15 (dd, *J* = 10.2, 2.3 Hz, 1H), 4.99 (ddd, *J* = 17.0, 2.3, 0.7 Hz, 1H), 2.10 (dd, *J* = 10.5, 2.5 Hz, 1H), 2.06 – 1.94 (m, 1H), 1.60 (dddd, *J* = 11.2, 4.9, 3.2, 1.7 Hz, 2H), 1.56 (s, 3H), 1.51 – 0.81 (m, 9H).

^{13}C NMR (126 MHz, $CDCl_3$) 161.6, (d, *J* = 244.8 Hz), 142.7 (d, *J* = 3.1 Hz), 136.0, 127.3 (d, *J* = 7.9 Hz), 119.1, 114.4 (d, *J* = 21.1 Hz), 75.6, 62.7, 37.3, 33.9, 29.3, 27.3, 26.7, 26.5, 26.2.

^{19}F NMR (471 MHz, $CDCl_3$) −116.8 (s).

HPLC: 89:11 *e.r.* [Chiralpak ODH (0.46 x 25 cm), particle size = 5 µm heptane/2-propanol = 99/1, ν = 1.0 mL min^{-1}, λ = 220 nm, t (minor) = 21.156 minutes, t (major) = 34.935 minutes].

$[\alpha]_D^{20}$ −40.0 (c 0.40, CH_2Cl_2).

(2*R*,3*S*)-3-Cyclohexyl-2-(4-(trifluoromethyl)phenyl)pent-4-en-2-ol **(2*R*,3*S*)-3p**

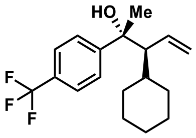

According to general procedure B, cyclohexylallene (72 μL, 0.5 mmol), 4-(trifluoromethyl)acetophenone (94 mg, 0.5 mmol), HBpin (100 μL, 0.69 mmol), (*S*)-*B*-OMe-10-Ph-9-borabicyclo[3.3.2]decane (6 mg, 0.05 mmol) were reacted at 50 °C for 16 hours. The crude reaction mixture was quenched (SiO_2) and purified by flash column chromatography (CombiFlash Isco NextGen300+, 12 g SiO_2, 50 mm Ø, petroleum ether/ ethyl acetate 10:1) to give the alcohol as a colourless oil (66 mg, 0.21 mmol, 42% yield, *anti:syn* 78:22).

^{1}H NMR (500 MHz, $CDCl_3$) 7.62 – 7.52 (m, 4H), 5.75 (dt, *J* = 17.0, 10.3 Hz, 1H), 5.13 (dd, *J* = 10.2, 2.2 Hz, 1H), 4.94 (ddd, *J* = 17.0, 2.2, 0.7 Hz, 1H), 2.13 (dd, *J* = 10.4, 2.5 Hz, 1H), 1.97 (s, 1H), 1.66 – 1.45 (m, 9H), 1.31 – 0.95 (m, 7H), 0.88 (td, *J* = 12.4, 3.3 Hz, 1H).

^{13}C NMR (126 MHz, $CDCl_3$) 151.3, 135.6, 128.8 (q, *J* = 32.3 Hz), 126.1, 124.7 (q, *J* = 3.8 Hz), 124.3 (q, *J* = 271.8 Hz), 119.5, 76.1, 62.4, 37.4, 34.0, 29.4, 27.6, 26.8, 26.6, 26.2.

^{19}F NMR (376 MHz, $CDCl_3$) -62.3 (s).

HPLC: 89:11 *e.r.* [Chiralpak ODH (0.46 x 25 cm), particle size = 5 μm heptane/2-propanol = 99/1, ν = 1.0 mL min^{-1}, λ = 220 nm, t (minor) = 21.768 minutes, t (major) = 23.276 minutes].

$[\alpha]_D^{20}$ −62.5 (c 0.80, CH_2Cl_2).

(2*R*,3*S*)-3-Cyclohexyl-2-(4-methoxyphenyl)pent-4-en-2-ol **(2*R*,3*S*)-3o**

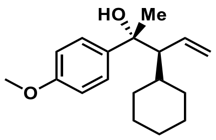

According to general procedure B, cyclohexylallene (72 µL, 0.5 mmol), 4-methoxyacetophenone (75 mg, 0.5 mmol), HBpin (100 µL, 0.69 mmol), (*S*)-*B*-OMe-10-Ph-9-borabicyclo[3.3.2]decane (6 mg, 0.05 mmol) were reacted at 50 °C for 16 hours. The crude reaction mixture was quenched (SiO_2) and purified by flash column chromatography (CombiFlash Isco NextGen300+, 12 g SiO_2, 50 mm Ø, petroleum ether/ ethyl acetate 10:1) to give the *alcohol* as a colourless oil (75 mg, 0.27 mmol, 55% yield, *anti:syn* >95:5).

^{1}H NMR (500 MHz, $CDCl_3$) 7.43 – 7.34 (m, 2H), 6.93 – 6.85 (m, 2H), 5.80 (dt, *J* = 17.0, 10.3 Hz, 1H), 5.17 (dd, *J* = 10.1, 2.4 Hz, 1H), 5.03 (ddd, *J* = 17.0, 2.4, 0.6 Hz, 1H), 3.83 (s, 3H), 2.14 (dd, *J* = 10.4, 2.5 Hz, 1H), 1.94 (s, 1H), 1.66 – 1.48 (m, 8H), 1.48 – 1.41 (m, 1H), 1.28 (dtt, *J* = 10.8, 3.3, 1.8 Hz, 1H), 1.24 – 0.95 (m, 4H), 0.86 (qd, *J* = 12.5, 3.3 Hz, 1H).

^{13}C NMR (126 MHz, $CDCl_3$) 158.7, 139.6, 136.9, 127.3, 119.3, 113.5, 76.1, 63.2, 55.6, 37.8, 34.5, 29.8, 27.8, 27.3, 27.0, 26.7.

HPLC: 80:20 *e.r.* [Chiralpak ODH (0.46 x 25 cm), particle size = 5 µm heptane/2-propanol = 99/1, ν = 1.0 mL min^{-1}, λ = 254 nm, t (minor) = 9.571 minutes, t (major) = 10.534 minutes].

$[\alpha]_D^{20}$ −42.9 (c 0.14, CH_2Cl_2).

(2*R*,3*S*)-3-Cyclohexyl-2-(3-methoxyphenyl)pent-4-en-2-ol **(2*R*,3*S*)-3p**

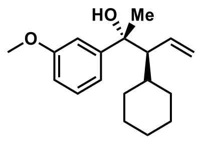

According to general procedure A, cyclohexylallene (72 µL, 0.5 mmol), 3-methoxyacetophenone (69 µL, 0.5 mmol), HBpin (87 µL, 0.6 mmol), (*S*)-*B*-OMe-10-Ph-9-borabicyclo[3.3.2]decane (6 mg, 0.05 mmol) were reacted in at 50 °C for 16 hours. The crude reaction mixture was quenched (SiO_2) and purified by flash column chromatography (CombiFlash Isco NextGen300+, 12 g SiO_2, 50 mm Ø, petroleum ether/ ethyl acetate 10:1) to give the alcohol as a colourless oil (99 mg, 0.36 mmol, 72% yield, *anti:syn* 91:9).

^{1}H NMR (600 MHz, $CDCl_3$) 7.26 (t, *J* = 8.0 Hz, 1H), 7.07 – 7.00 (m, 2H), 6.80 (dd, *J* = 8.2, 2.6 Hz, 1H), 5.81 (dt, *J* = 16.9, 10.3 Hz, 1H), 5.15 (dd, *J* = 10.2, 2.3 Hz, 1H), 5.00 (dd, *J* = 17.0, 2.4 Hz, 1H), 3.84 (s, 3H), 2.16 (dd, *J* = 10.5, 2.4 Hz, 1H), 1.95 (s, 1H), 1.67 – 1.54 (m, 6H), 1.46 (dq, *J* = 12.9, 2.3, 1.9 Hz, 1H), 1.37 – 0.79 (m, 7H).

^{13}C NMR (126 MHz, $CDCl_3$) 159.5, 149.0, 136.4, 128.9, 119.0, 118.4, 112.0, 111.9, 76.2, 62.6, 55.4, 37.5, 34.2, 29.5, 27.8, 27.0, 26.8, 26.5.

HPLC: 89:11 *e.r.* [Chiralpak ODH (0.46 x 25 cm), particle size = 5 µm heptane/2-propanol = 95/5, ν = 1.0 mL min^{-1}, λ = 220 nm, t (major) = 9.987 minutes, t (minor) = 16.852 minutes].

$[\alpha]_D^{20}$ −34.0 (c 0.23, CH_2Cl_2).

(2*S*,3*R*)-3-Cyclohexyl-2-phenylpent-4-en-2-ol **(2*S*,3*R*)-3a**

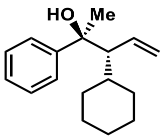

According to general procedure B, cyclohexylallene (72 μL, 0.5 mmol), acetophenone (59 μL, 0.5 mmol), HBpin (100 μL, 0.69 mmol), (*R*)-*B*-OMe-10-Ph-9-borabicyclo[3.3.2]decane (6 mg, 0.05 mmol) were reacted at 50 °C for 16 hours. The crude reaction mixture was quenched (SiO_2) and purified by flash column chromatography (CombiFlash Isco NextGen300+, 12 g SiO_2, 50 mm Ø, petroleum ether/ ethyl acetate 10:1) to give the alcohol as a colourless oil (82 mg, 0.34 mmol, 67% yield, *anti:syn* >95:5).

^{1}H NMR (600 MHz, $CDCl_3$) δ 7.46 – 7.41 (m, 2H), 7.35 – 7.29 (m, 2H), 7.25 – 7.20 (m, 1H), 5.78 (dt, *J* = 17.0, 10.3 Hz, 1H), 5.13 (dd, *J* = 10.2, 2.4 Hz, 1H), 4.98 (ddd, *J* = 17.0, 2.4, 0.7 Hz, 1H), 2.14 (dd, *J* = 10.5, 2.5 Hz, 1H), 1.93 (s, 1H), 1.59 (m, 5H), 1.45 – 0.77 (m, 9H).

^{13}C NMR (101 MHz, $CDCl_3$) 147.1, 136.3, 127.8, 126.6, 125.7, 118.9, 76.1, 62.6, 37.4, 34.0, 29.4, 27.5, 26.9, 26.6, 26.3.

Data were in accordance with those previously reported.[5]

HPLC: 92:8 *e.r.* [Chiralpak ODH (0.46 x 25 cm), particle size = 5 μm heptane/2-propanol = 95/5, ν = 1.0 mL min^{-1}, λ = 220 nm, t (minor) = 6.979 minutes, t (major) = 8.245 minutes].

$[\alpha]_D^{20}$ +26.3 (c 0.19, CH_2Cl_2).

(2*S*,3*S*)-3-(1-Ethyl-2-phenyl)-2-phenylpent-4-en-2-ol **(2*S*,3*S*)-3b**

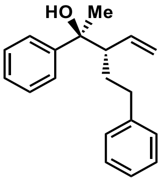

According to general procedure B, penta-3,4-dienyl-benzene (100 μL, 0.5 mmol), acetophenone (59 μL, 0.5 mmol), HBpin (100 μL, 0.69 mmol), (*R*)-*B*-OMe-10-Ph-9-borabicyclo[3.3.2]decane (6 mg, 0.05 mmol) were reacted at 50 °C for 16 hours. The crude reaction mixture was quenched (SiO_2) and purified by flash column chromatography (CombiFlash Isco NextGen300+, 12 g SiO_2, 50 mm Ø, petroleum ether/ ethyl acetate 10:1) to give the *alcohol* as a colourless oil (82 mg, 0.31 mmol, 62% yield, *anti:syn* 76:24).

^{1}H NMR (600 MHz, $CDCl_3$) 7.47 – 7.34 (m, 4H), 7.36 (m, 3H), 7.22 – 7.18 (m, 1H), 7.11 – 7.04 (m, 2H), 5.69 (dt, *J* = 17.1, 10.2 Hz, 1 H), 5.35 – 5.30 (m, 1H), 5.23 (dd, *J* = 17.2, 1.9 Hz, 1H), 2.66 (ddd, *J* = 14.4, 10.2, 4.6 Hz, 1H), 2.43 – 2.32 (m, 2H), 2.2 – 1.9 (br s, 1H), 1.88 (ddd, *J* = 13.0, 10.0, 7.3 Hz, 1H), 1.60 (s, 3 H), 1.42 (m, 1H)

^{13}C NMR (151 MHz, $CDCl_3$) 147.1, 139.4, 128.3, 127.1, 126.4, 119.3, 76.0, 57.1, 32.2, 29.6, 29.1, 28.2, 25.9, 23.1, 14.5.

HPLC: 89:11 *e.r.* [Chiralpak ADH (0.46 x 25 cm), particle size = 5 μm heptane/2-propanol = 95/5, ν = 1.0 mL min^{-1}, λ = 220 nm, t (minor) = 8.240 minutes, t (major) = 8.875 minutes].

$[\alpha]_D^{20}$ +18.1 (c 0.10, CH_2Cl_2).

(2*S*,3*R*)-2-(Thiophen-2-yl)-3-cyclohexylpent-4-en-2-ol **(2*S*,3*R*)-3z**

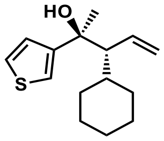

According to general procedure B, cyclohexylallene (72 µL, 0.5 mmol), 3-acetylthiophene (63 mg, 0.5 mmol), HBpin (100 µL, 0.69 mmol), (*R*)-*B*-OMe-10-Ph-9-borabicyclo[3.3.2]decane (6 mg, 0.05 mmol) were reacted at 50 °C for 16 hours. The crude reaction mixture was quenched (SiO_2) and purified by flash column chromatography (CombiFlash Isco NextGen300+, 12 g SiO_2, 50 mm Ø, petroleum ether/ ethyl acetate 10:1) to give the *alcohol* as a colourless oil (80 mg, 0.32 mmol, 64% yield, *anti:syn* 79:21).

^{1}H NMR (500 MHz, $CDCl_3$) 7.25 (dd, *J* = 4.6, 2.5 Hz, 1H), 7.15 (dd, *J* = 3.0, 1.4 Hz, 1H), 7.07 (dd, *J* = 5.0, 1.4 Hz, 1H), 5.78 (dt, *J* = 16.9, 10.3 Hz, 1H), 5.16 (dd, *J* = 10.1, 2.3 Hz, 1H), 5.04 (ddd, *J* = 17.0, 2.4, 0.7 Hz, 1H), 2.13 (dd, *J* = 10.4, 2.5 Hz, 1H), 2.00 (s, 1H), 1.69 – 1.56 (m, 3H), 1.55 (s, 3H), 1.40 – 1.28 (m, 2H), 1.28 – 1.13 (m, 1H), 1.15 – 0.94 (m, 4H), 0.83 (qd, *J* = 12.5, 3.4 Hz, 1H).

^{13}C NMR (126 MHz, $CDCl_3$) 148.8, 136.2, 126.2, 125.0, 120.1, 118.8, 74.9, 62.3, 37.5, 33.9, 29.2, 27.7, 26.8, 26.5, 26.2.

HPLC: 75:25 *e.r.* [Chiralpak ODH (0.46 x 25 cm), particle size = 5 µm heptane/2-propanol = 99/1, ν = 1.0 mL min^{-1}, λ = 254 nm, t (major) = 36.439 minutes, t (minor) = 43.499 minutes].

$[\alpha]_D^{20}$ +31.4 (c 0.50, CH_2Cl_2).

(2*R*,3*R*)-4-(2-(7-Methoxynaphthalene)-2-ethenylcyclohexyl-butan-2-ol **(2*R*,3*R*)-3ad**

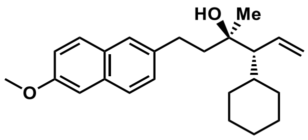

According to general procedure B, cyclohexylallene (72 μL, 0.5 mmol), nabumetone (114 mg, 0.5 mmol), HBpin (100 μL, 0.69 mmol), (*R*)-*B*-OMe-10-Ph-9-borabicyclo[3.3.2]decane (6 mg, 0.05 mmol) were reacted at 50 °C for 16 hours. The crude reaction mixture was quenched (SiO_2) and purified by flash column chromatography (CombiFlash Isco NextGen300+, 12 g SiO_2, 50 mm Ø, petroleum ether/ ethyl acetate 10:1) to give the *alcohol* as a colourless oil (90 mg, 0.26 mmol, 51% yield, *anti:syn* 91:9).

^{1}H NMR (600 MHz, $CDCl_3$) δ 7.71 (d, *J* = 8.6 Hz, 2H), 7.60 (s, 1H), 7.35 (d, *J* = 8.4 Hz, 1H), 7.16 (d, *J* = 13.6 Hz, 2H), 5.90 (dt, *J* = 17.0, 10.3 Hz, 1H), 5.27 (d, *J* = 10.1 Hz, 1H), 5.14 (d, *J* = 17.1 Hz, 1H), 3.94 (s, 3H), 2.86 (t, *J* = 7.9 Hz, 2H), 2.00 (d, *J* = 10.6 Hz, 1H), 1.91 (dtt, *J* = 25.4, 17.6, 9.5 Hz, 3H), 1.79 – 1.59 (m, 5H), 1.54 (d, *J* = 13.0 Hz, 1H), 1.35 (d, *J* = 12.4 Hz, 1H), 1.38 – 1.17 (m, 6H), 1.17 – 1.10 (m, 1H), 1.10 – 1.03 (m, 2H).

^{13}C NMR (151 MHz, $CDCl_3$) 157.2, 138.1, 136.3, 133.0, 129.2, 128.9, 127.9, 126.8, 126.2, 119.2, 118.7, 105.7, 74.1, 60.3, 55.3, 43.1, 37.8, 34.4, 30.1, 29.8, 27.0, 26.8, 26.4, 25.2.

HPLC: 60:40 *e.r.* [Chiralpak ODH (0.46 x 25 cm), particle size = 5 μm heptane/2-propanol = 98/2, ν = 1.0 mL min^{-1}, λ = 220 nm, t (minor) = 15.183 minutes, t (major) = 19.478 minutes].

$[\alpha]_D^{20}$ +6.9 (c 4.9, CH_2Cl_2).

S7 Mechanistic studies

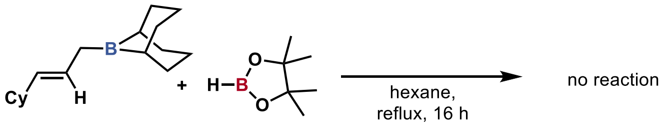

Cyclohexylallene (15 µL, 0.1 mmol) and [H-*B*-9-BBN]$_2$ (12 mg, 0.05 mmol) were reacted in THF for 16 hours under reflux in an argon atmosphere. The resulting allylicborane was analysed by ^{1}H NMR spectroscopy with the data in accordance with those previously reported.[8] To this solution was added HBpin (87 µL, 0.6 mmol) and the mixture was heated under reflux for 16 hours. The solution was analysed by ^{1}H NMR spectroscopy with no boron-boron exchange observed and the allylic-*B*-9-BBN recovered.

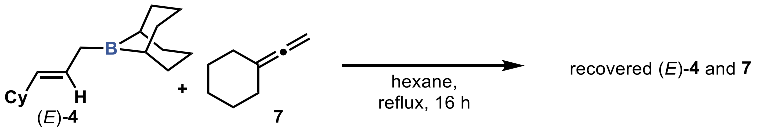

Cyclohexylallene (15 µL, 0.1 mmol) and [H-*B*-9-BBN]$_2$ (12 mg, 0.05 mmol) were reacted in THF for 16 hours under reflux in an argon atmosphere. The resulting allylicborane was analysed by ^{1}H NMR spectroscopy with the data in accordance with those previously reported.[8] To this solution was added vinylidenecyclohexane (11 mg, 0.1 mmol), and the mixture was heated under reflux for 16 hours. The solution was analysed by ^{1}H NMR spectroscopy with no boron-boron exchange observed and allylic-*B*-9-BBN recovered.

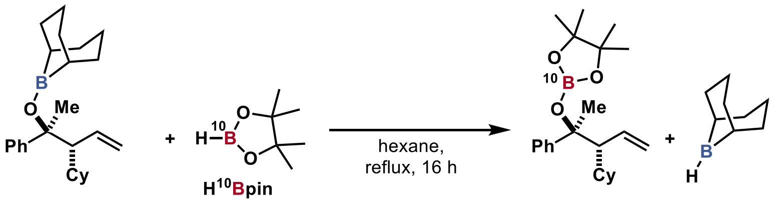

Cyclohexylallene (37 µL, 0.25 mmol) and [H-*B*-9-BBN]$_2$ (30 mg, 0.125 mmol) were reacted in hexane for 16 hours under reflux in an argon atmosphere. The resulting allylicborane was analysed by ^{11}B NMR spectroscopy (δ 39 ppm). To the allylic-*B*-9-BBN was added acetophenone (30 µL, 0.25 mmol) which gave the corresponding homoallylic borinic ester (^{11}B NMR = 56 ppm). The homoallylic borinic ester was added ^{10}B-HBpin (88 µL) and the resulting solution was heated under reflux for 16 hours to give the homoallylic boronic ester (^{10}B NMR = 22 ppm).

(2*SR*,3*RS*)-2-Deuterio-3-cyclohexyl-2-phenylpent-4-en-2-ol **3aD**

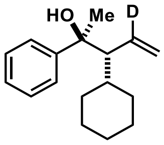

(±)

According to general procedure A, cyclohexylallene (72 µL, 0.5 mmol), acetophenone (59 µL, 0.5 mmol), DBpin (88 µL, 0.6 mmol), [H-*B*-9-BBN]$_2$ (6 mg, 0.025 mmol) were reacted in *n*-hexane (1 mL) and heated under reflux for 16 hours. The crude reaction mixture was quenched (SiO_2) and purified by flash column chromatography (CombiFlash Isco NextGen300+, 12 g SiO_2, 50 mm Ø, petroleum ether/ ethyl acetate 10:1) to give the alcohol as a colourless oil (51 mg, 0.21 mmol, 42% yield, *anti:syn* >95:5).

^{1}H NMR (500 MHz, $CDCl_3$) 7.51 – 7.44 (m, 2H), 7.35 (dd, *J* = 8.4, 6.9 Hz, 2H), 7.28 – 7.24 (m, 1H), 5.81 (dt, *J* = 17.0, 10.3 Hz, 0.31H), 5.16 (ddd, *J* = 5.5, 4.4, 2.4 Hz, 1H), 5.03 – 4.97 (m, 1H), 2.20 – 2.14 (m, 1H), 2.01 (s, 1H), 1.69 – 1.55 (m, 7H), 1.47 – 1.40 (m, 1H), 1.30 (dtd, *J* = 11.5, 3.6, 1.9 Hz, 1H), 1.25 – 0.97 (m, 4H), 0.92 – 0.81 (m, 1H).

^{2}H NMR (77 MHz, $CHCl_3$) 5.83 (br s).

^{13}C NMR (126 MHz, $CDCl_3$) 147.1, 136.0 (*t*, *J* = 22.5 Hz), 127.8, 126.6, 125.7, 118.9, 76.1, 62.5, 37.4, 34.1, 29.4, 27.5, 26.9, 26.6, 26.3.

S8 NMR Data

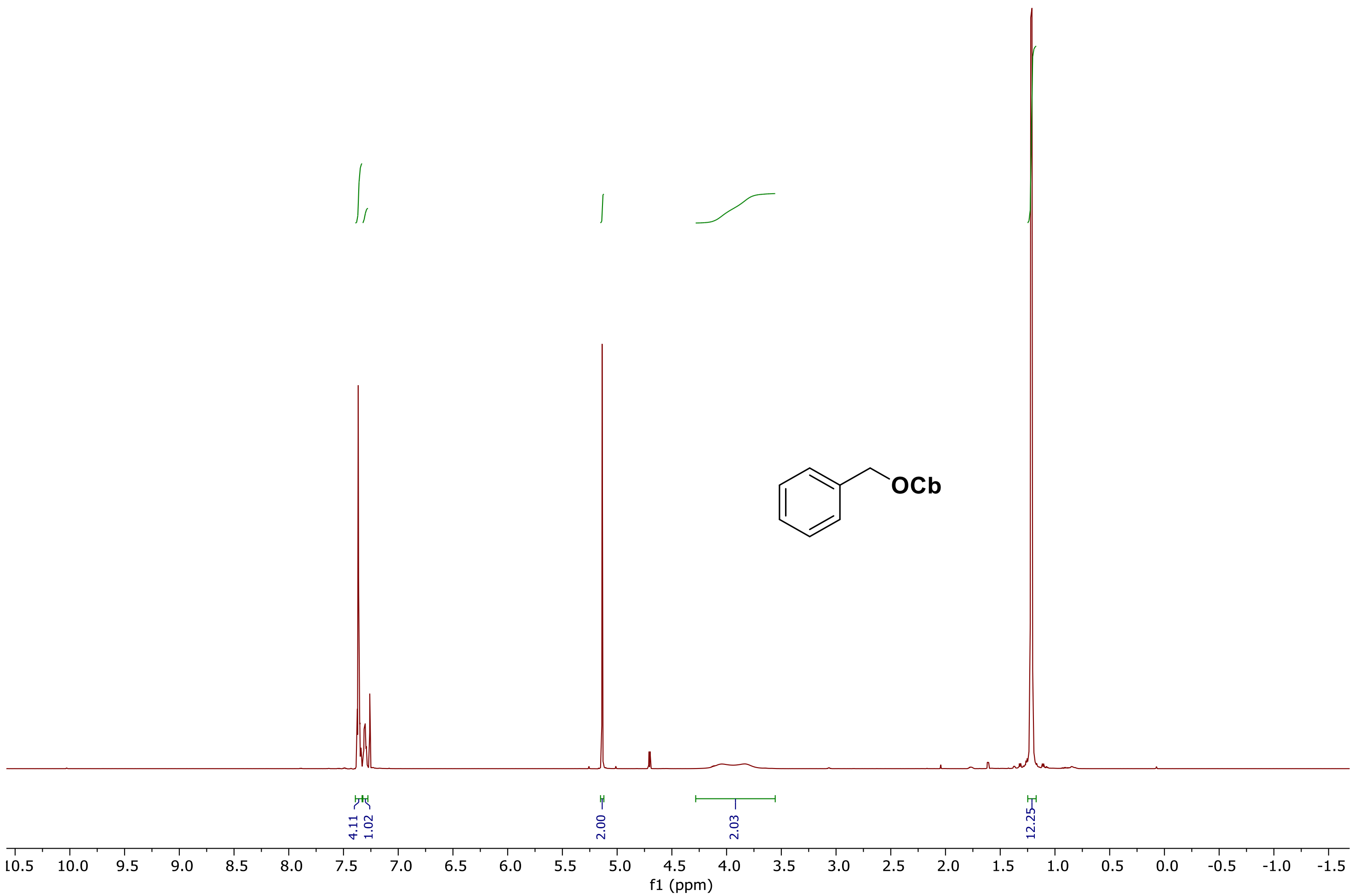

^{1}H NMR (600 MHz, $CDCl_3$) Spectra of benzyl *N*,*N*-di*iso*propylcarbamate

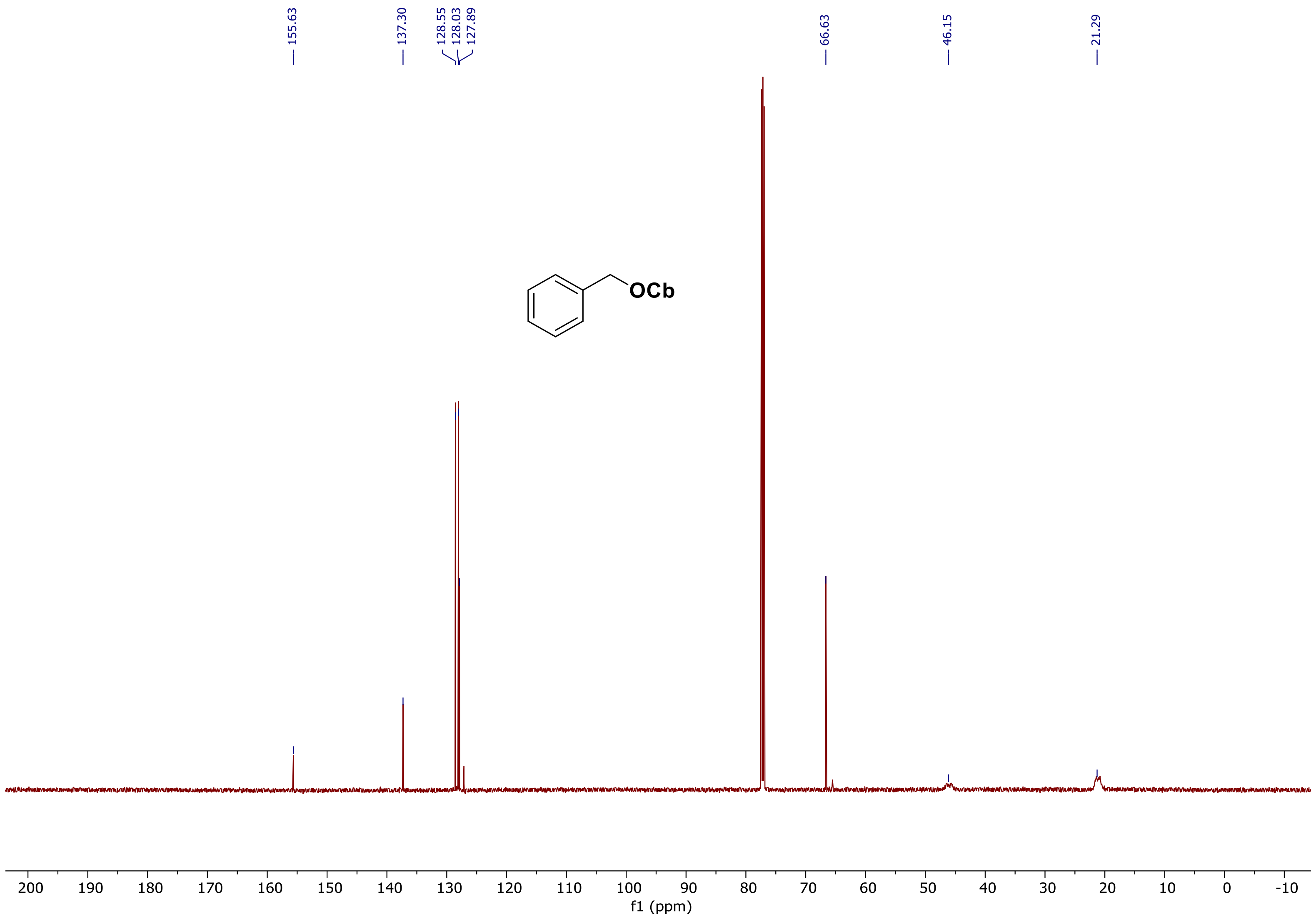

^{13}C NMR (126 MHz, $CDCl_3$) Spectra of benzyl *N*,*N*-di*iso*propylcarbamate

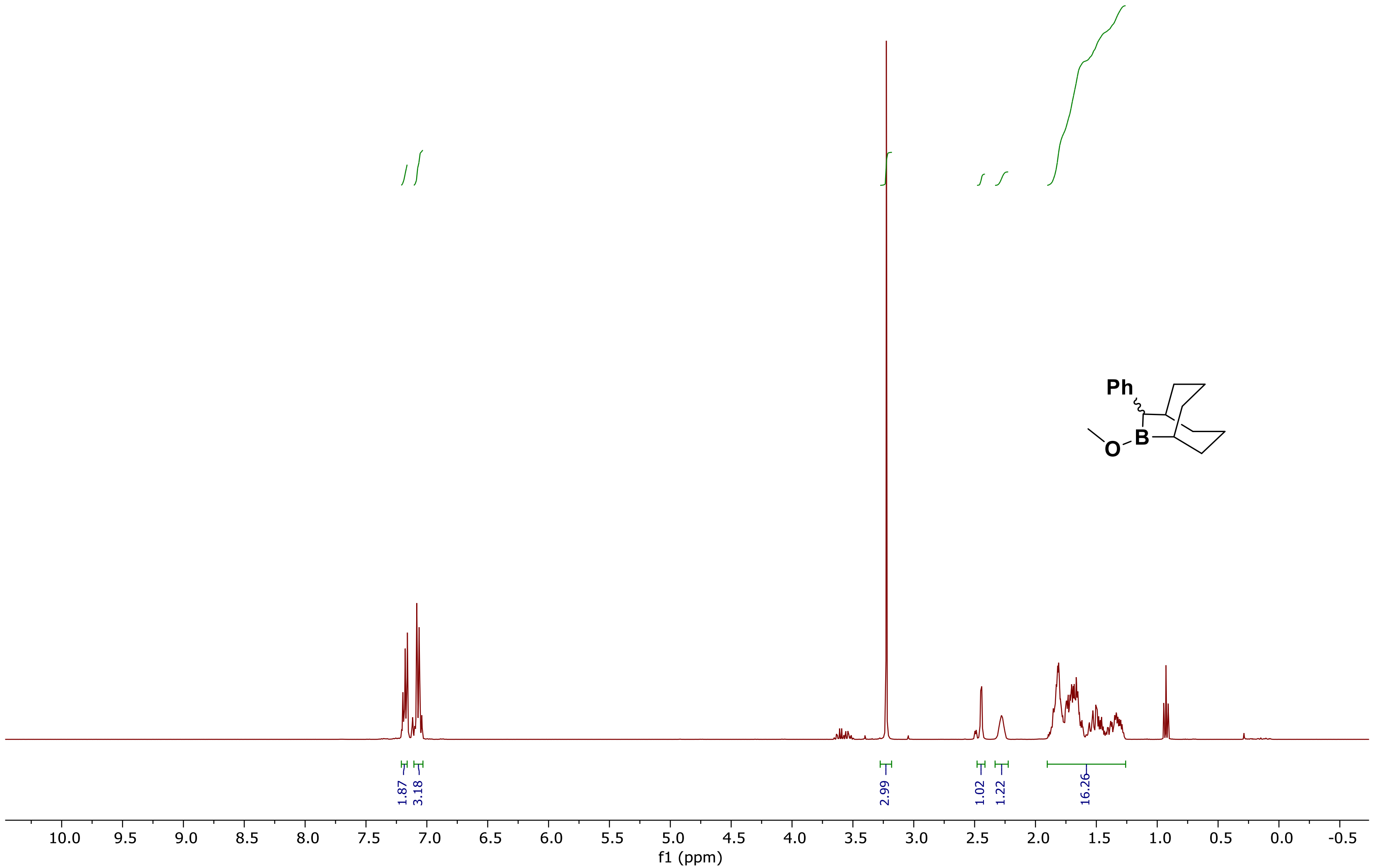

^{1}H NMR (600 MHz, C_6D_6) Spectra of (±)-*B*-methoxy-10-phenyl-9-borabicyclo[3.3.2]decane

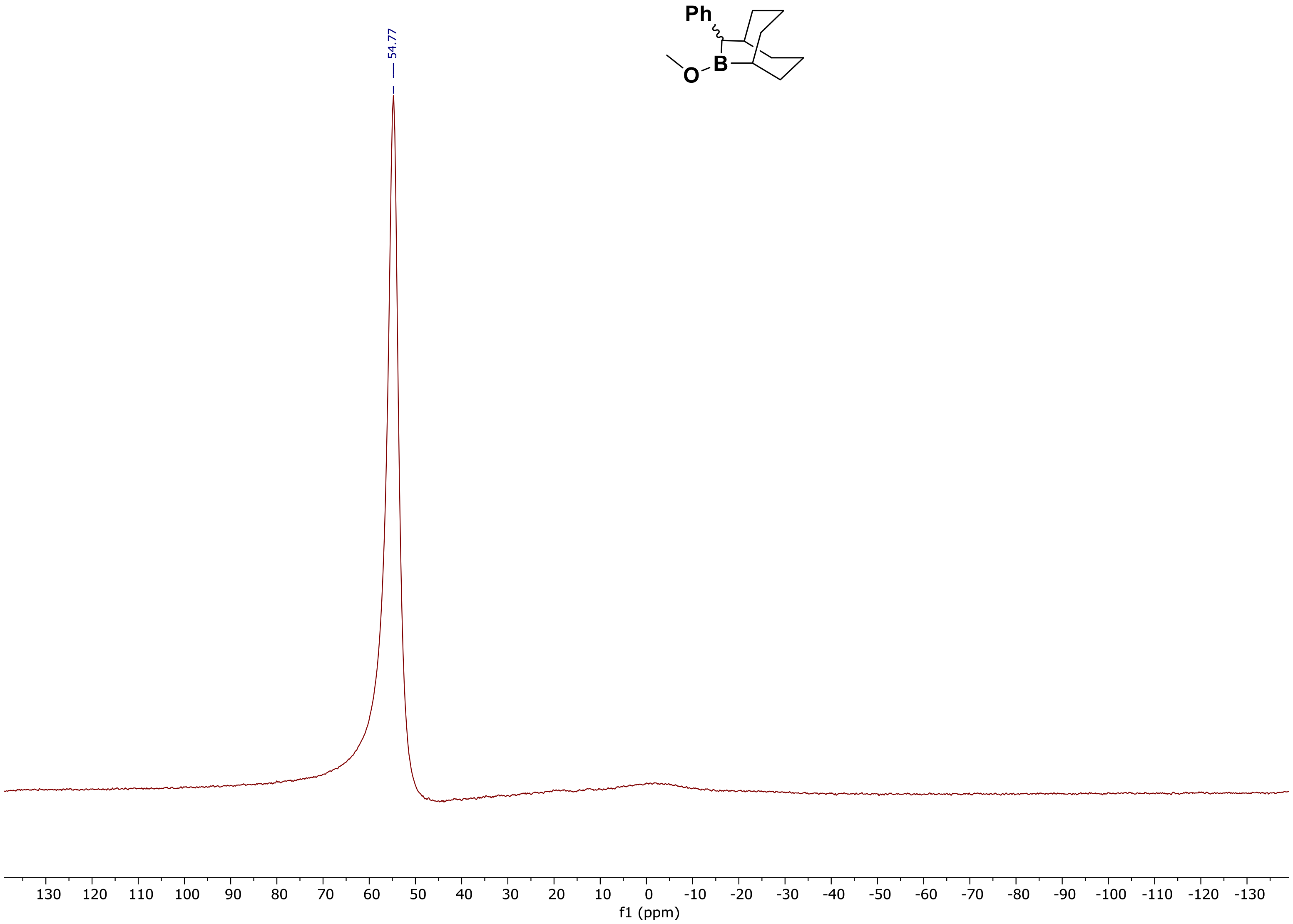

^{11}B NMR (128 MHz, C_6D_6) Spectra of (±)-*B*-methoxy-10-phenyl-9-borabicyclo[3.3.2]decane

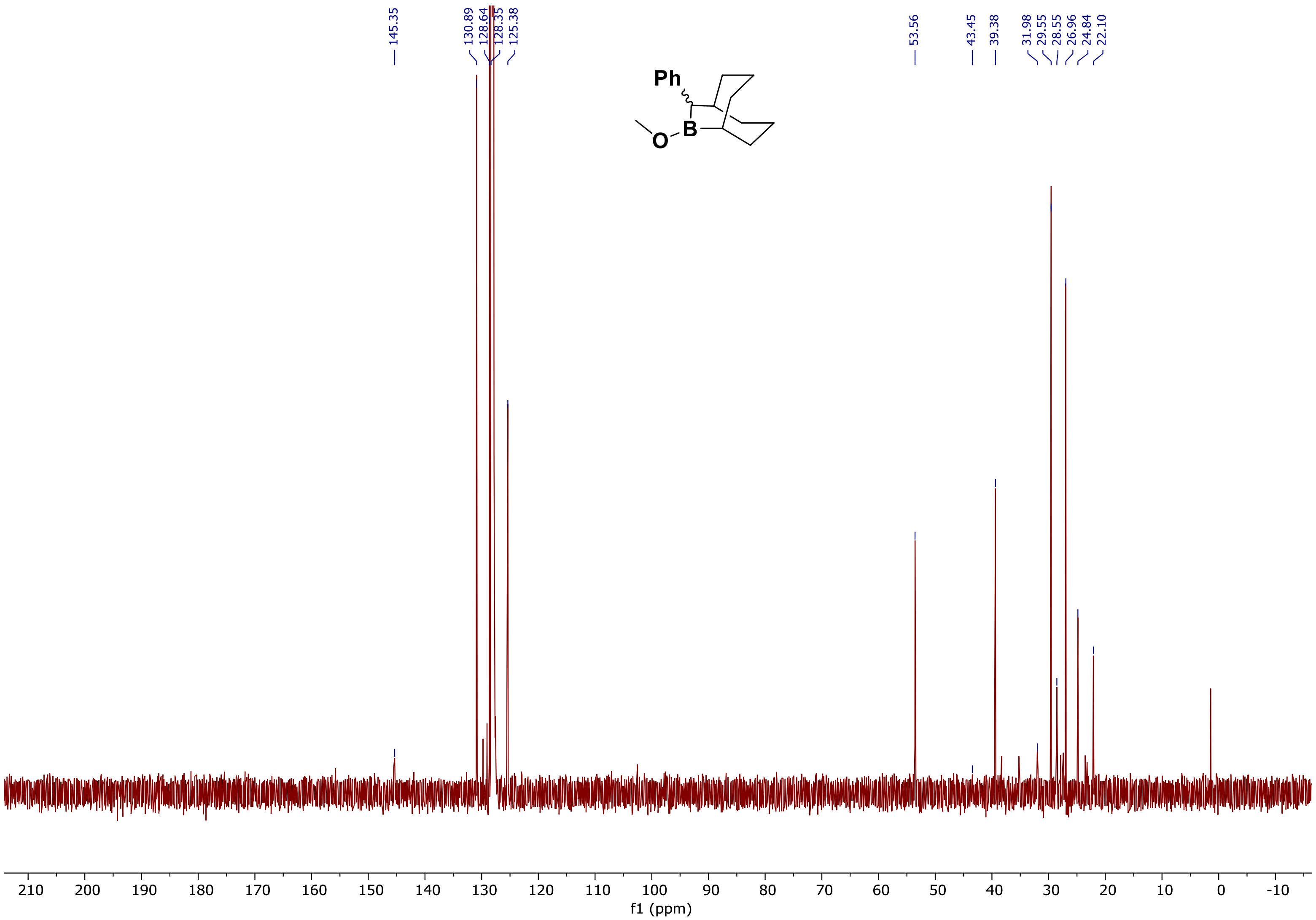

^{13}C NMR (126 MHz, C_6D_6) Spectra of (±)-*B*-methoxy-10-phenyl-9-borabicyclo[3.3.2]decane

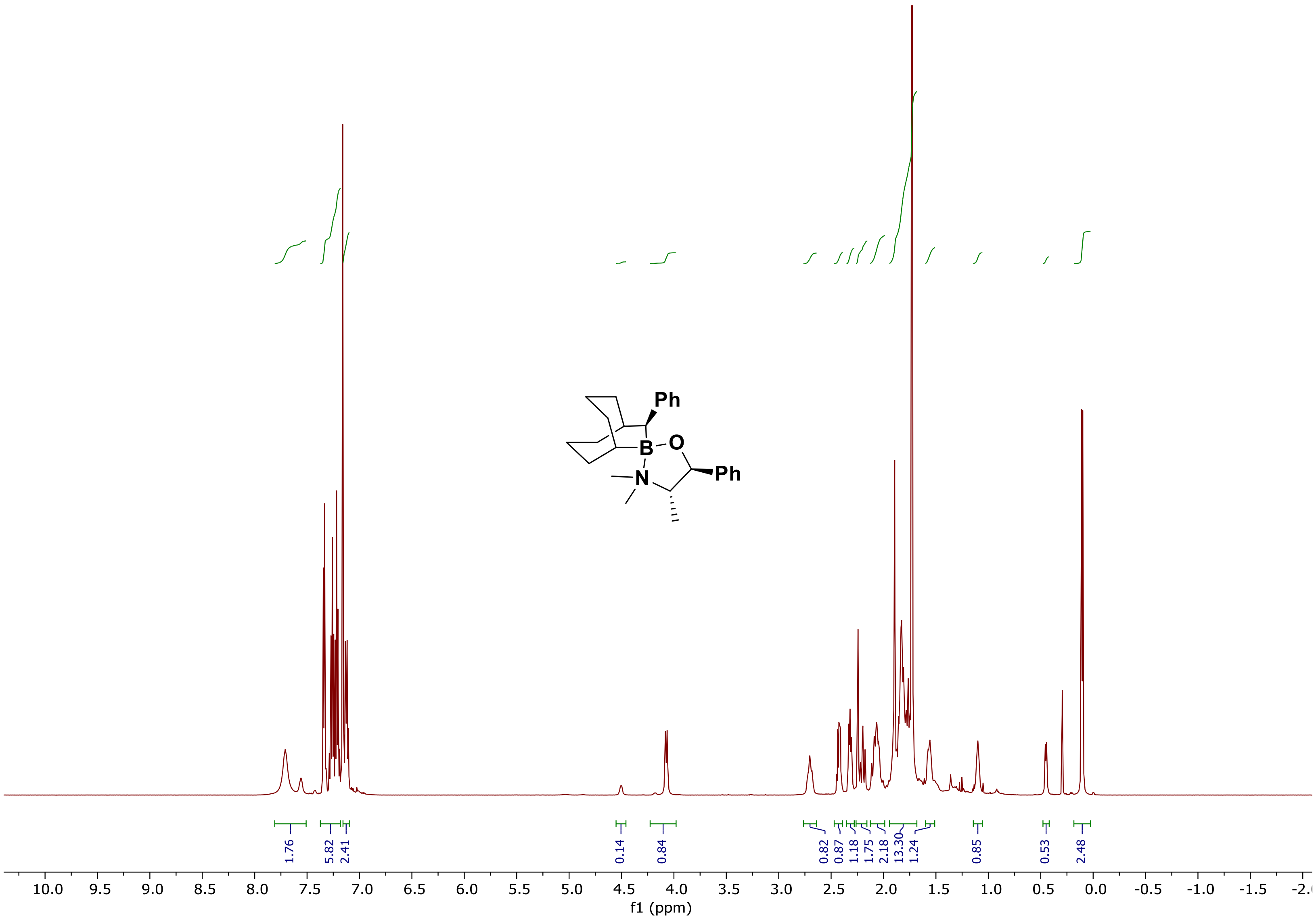

^{1}H NMR (600 MHz, C_6D_6) Spectra of (+)-*B*-((1S,2S)-*N*-methylpseudoephedrinyl))-(10S)-phenyl-9-borabicyclo[3.3.2]decane

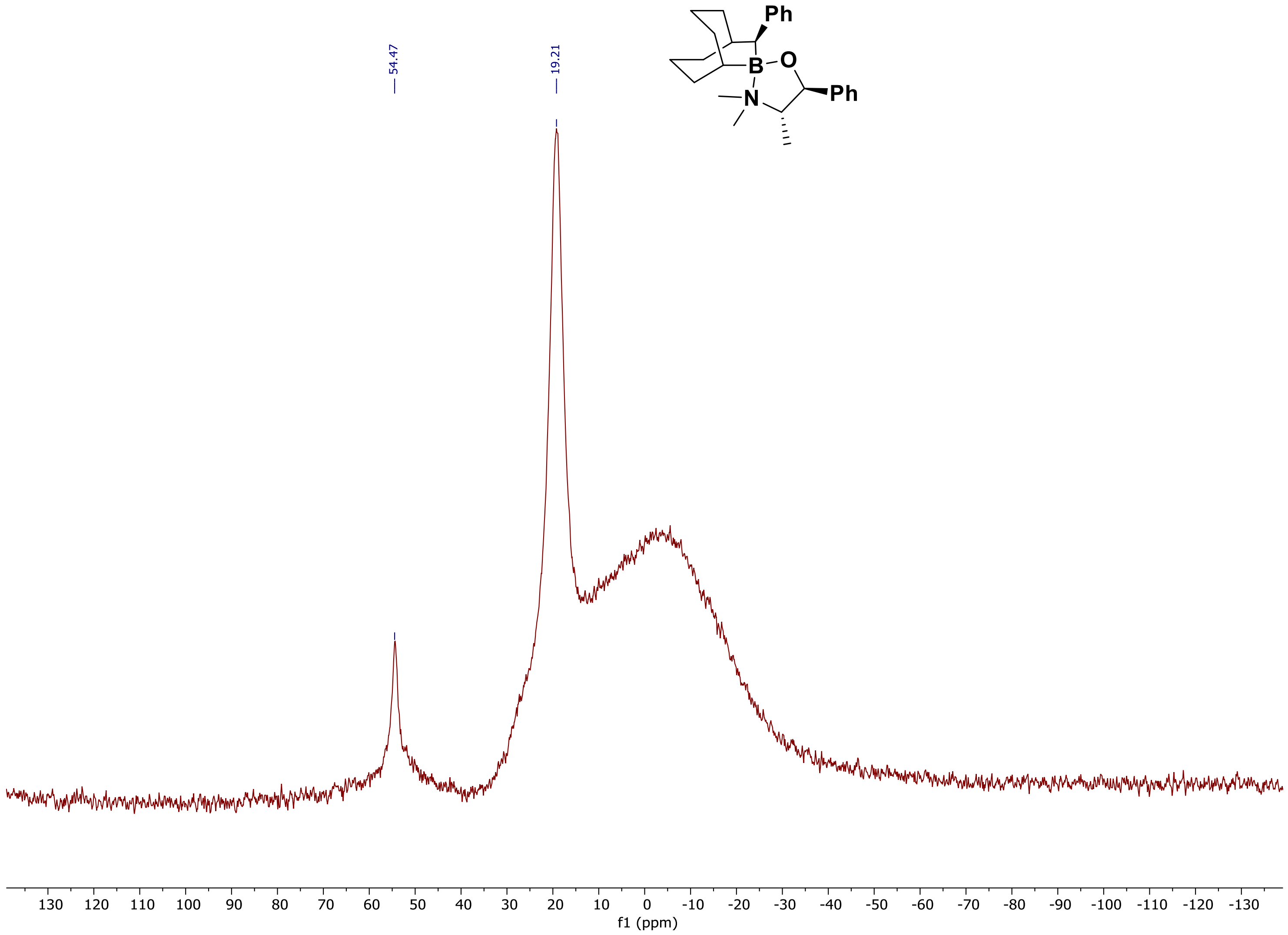

^{11}B NMR (128 MHz, C_6D_6) Spectra of (+)-*B*-((1S,2S)-*N*-methylpseudoephedrinyl))-(10S)-phenyl-9-borabicyclo[3.3.2]decane

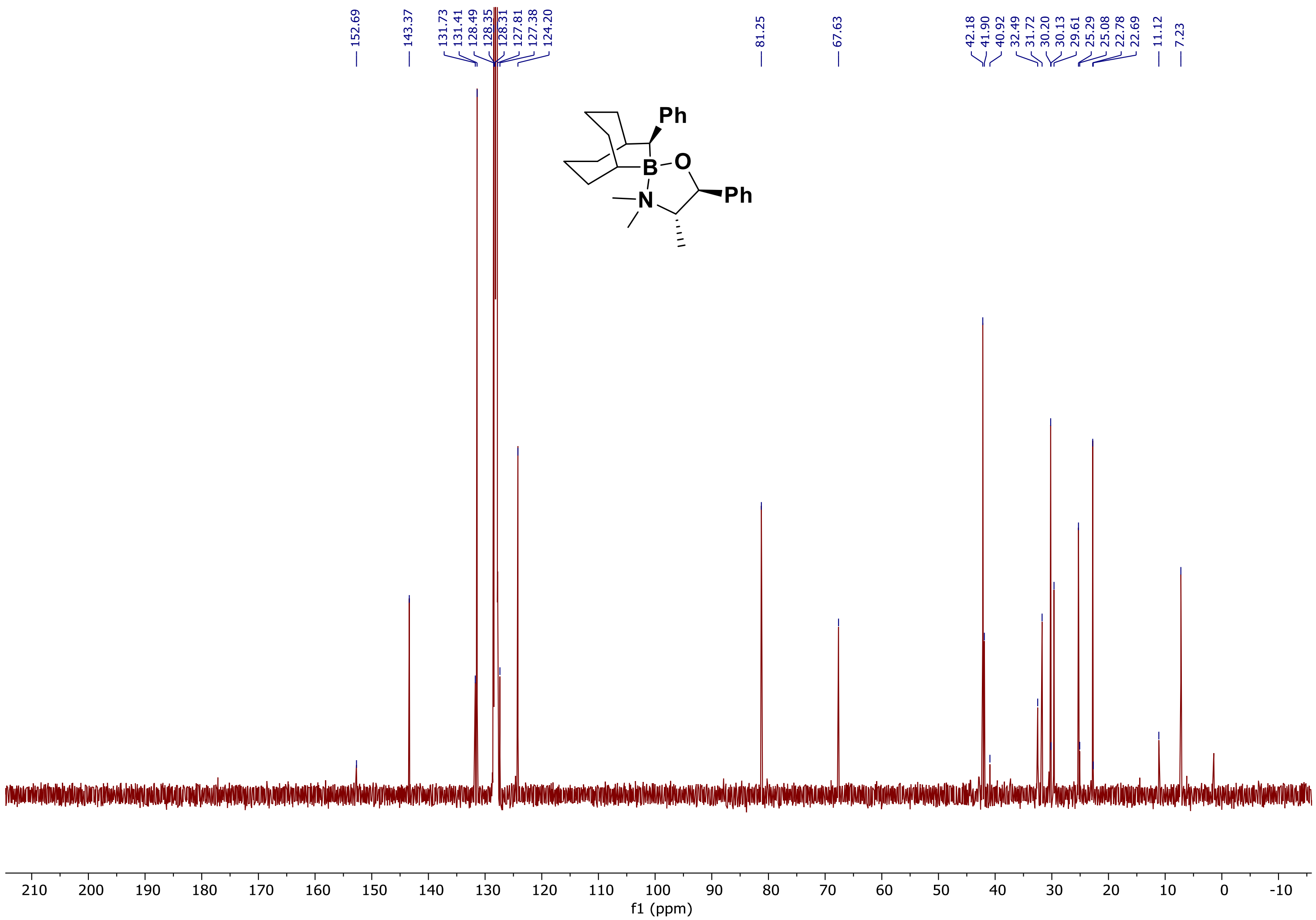

^{13}C NMR (126 MHz, C_6D_6) Spectra of (+)-*B*-((1S,2S)-*N*-methylpseudoephedrinyl))-(10S)-phenyl-9-borabicyclo[3.3.2]decane

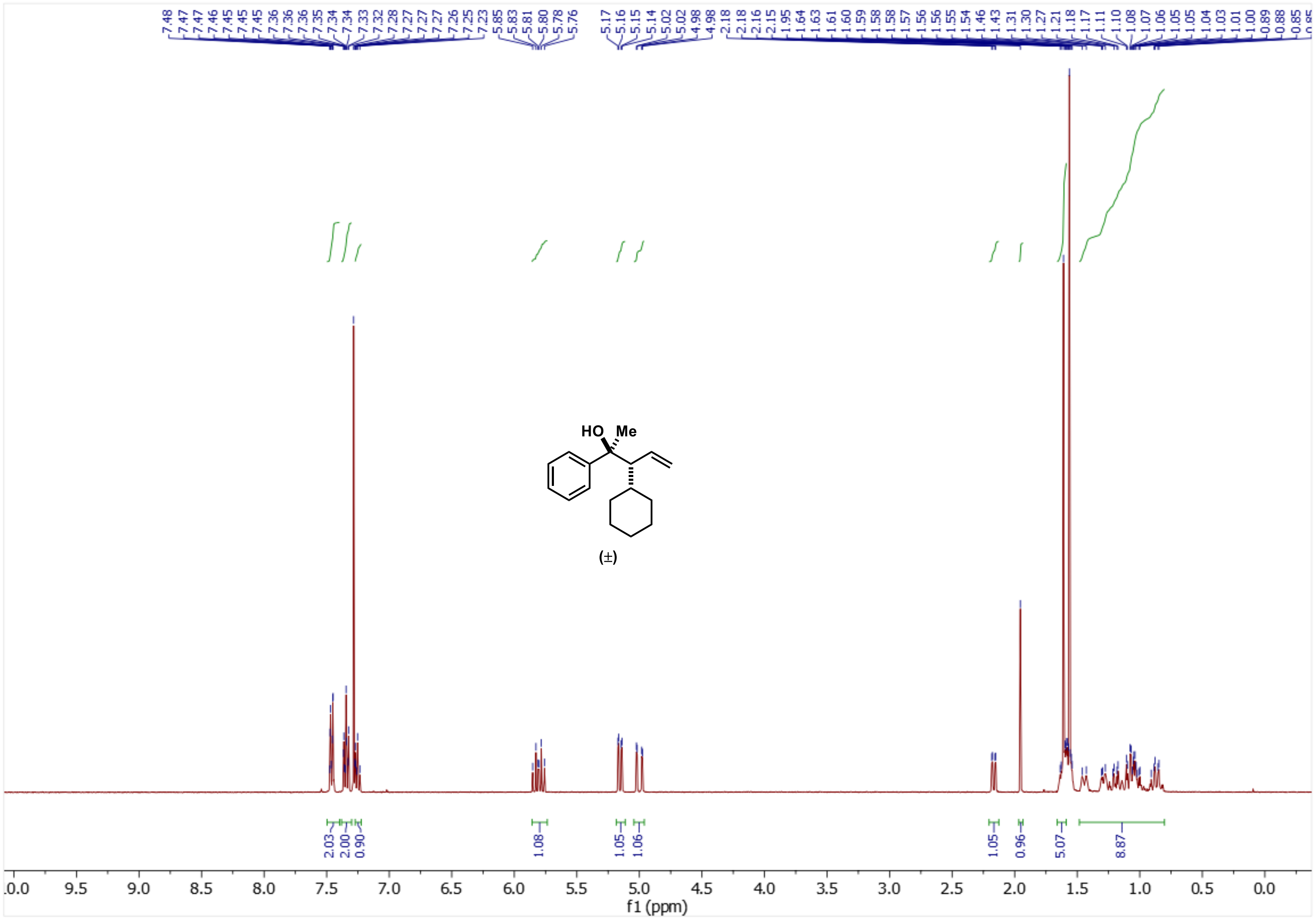

^{1}H NMR (400 MHz, $CDCl_3$) Spectra of (2*SR*,3*RS*)-3-cyclohexyl-2-phenylpent-4-en-2-ol

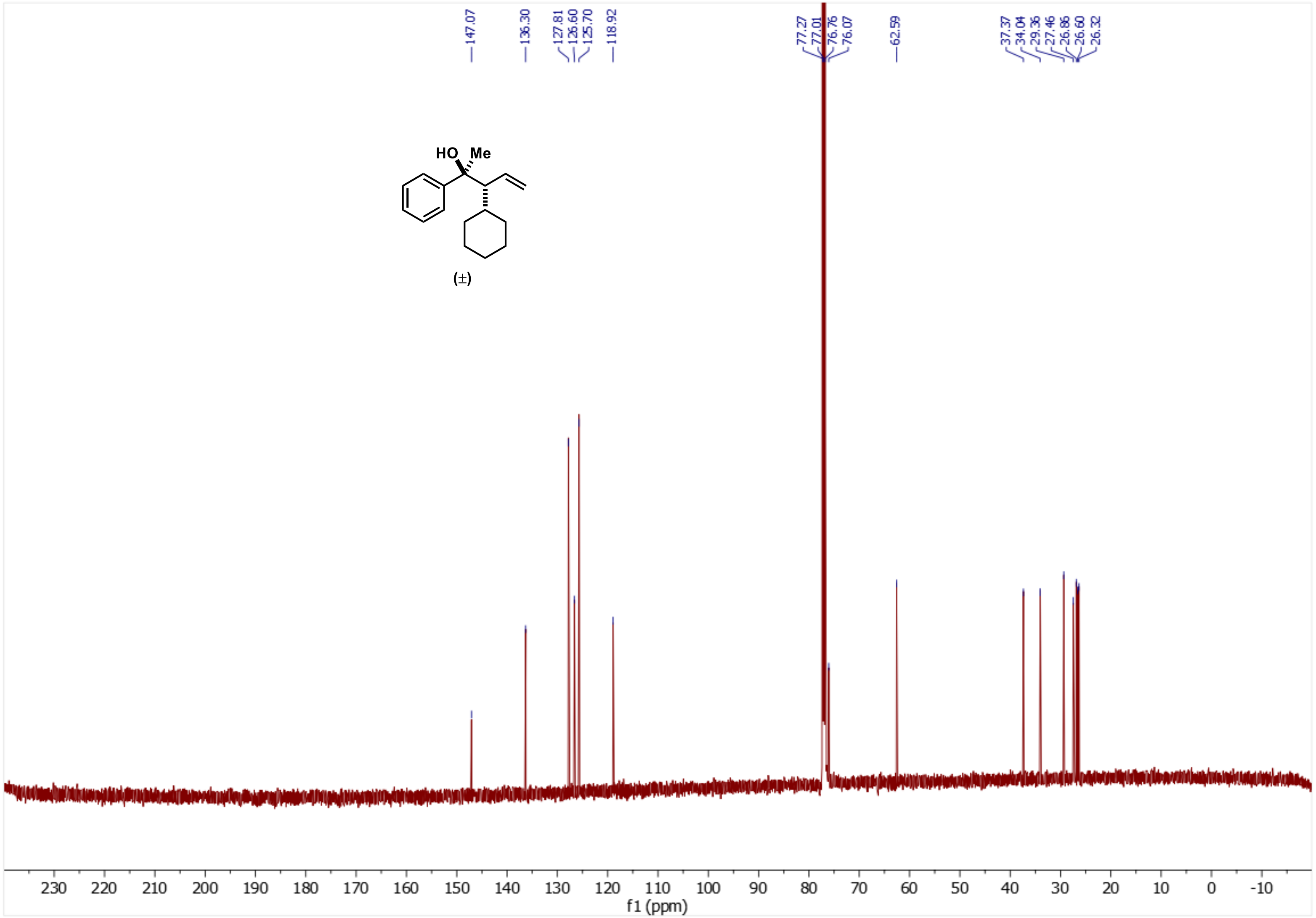

^{13}C NMR (126 MHz, $CDCl_3$) Spectra of (2*SR*,3*RS*)-3-cyclohexyl-2-phenylpent-4-en-2-ol

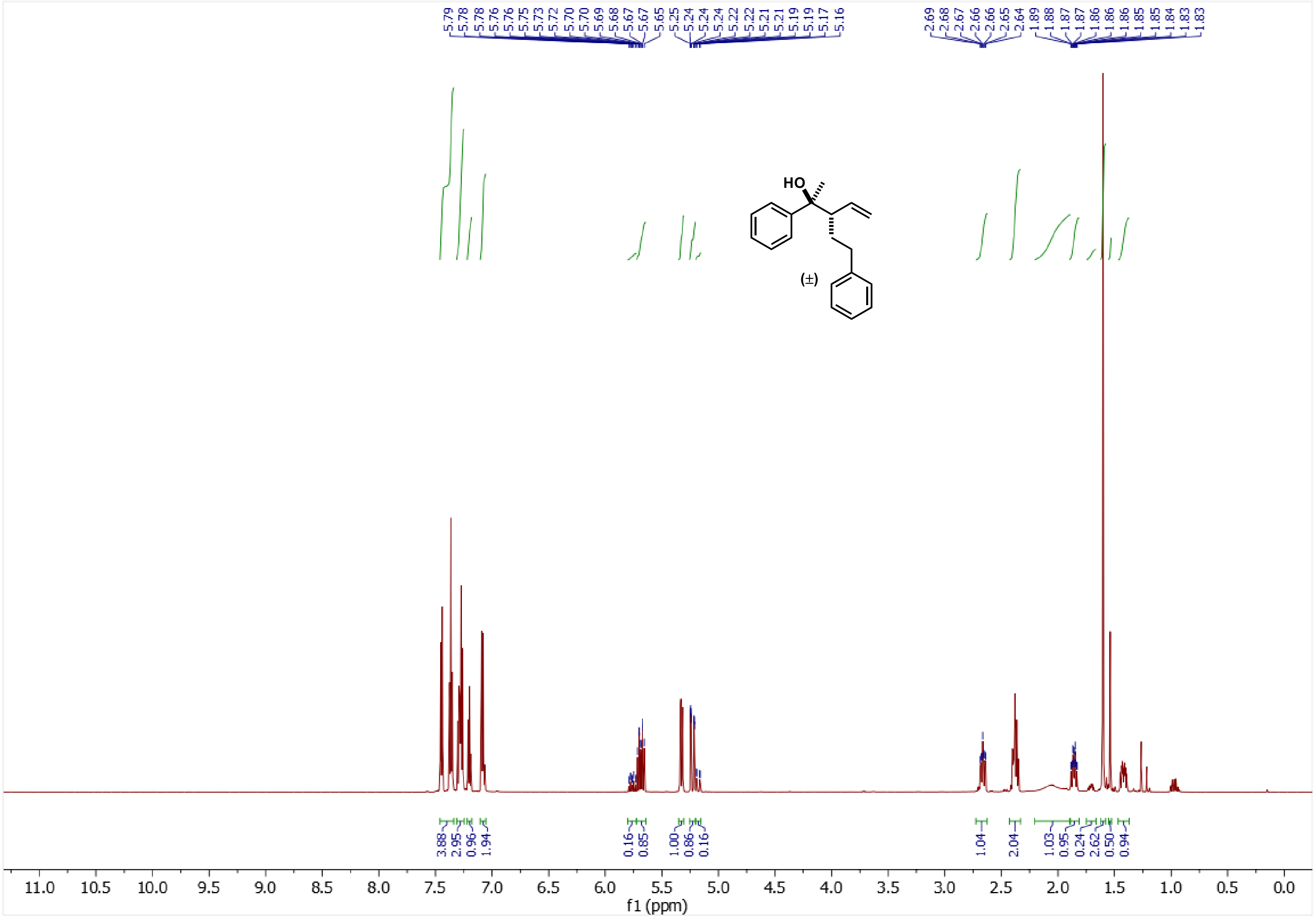

^{1}H NMR (600 MHz, $CDCl_3$) Spectra of (2*RS*,3*RS*)-3-(1-ethyl-2-phenyl)-2-phenylpent-4-en-2-ol

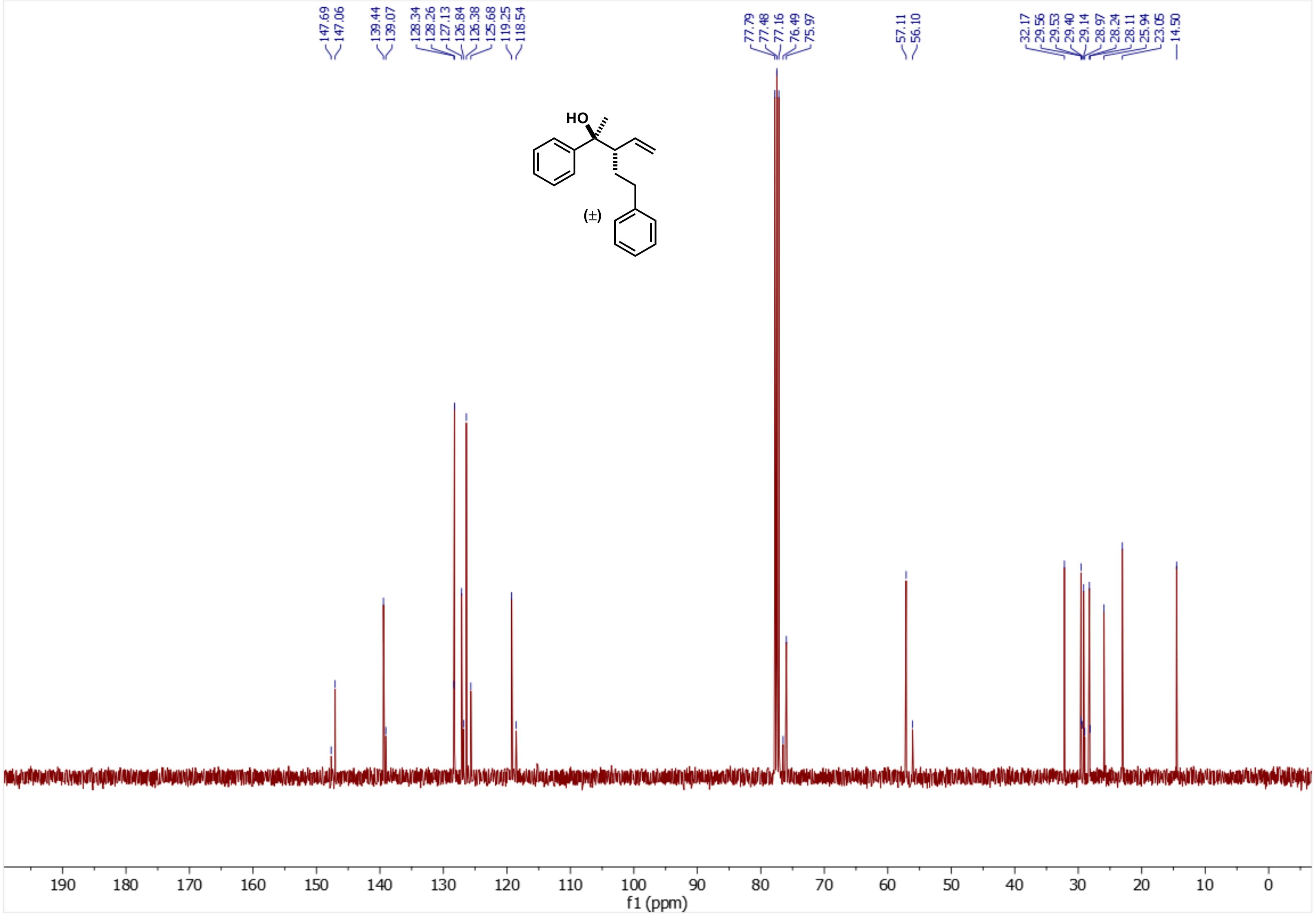

^{13}C NMR (151 MHz, $CDCl_3$) Spectra of (2*RS*,3*RS*)-3-(1-ethyl-2-phenyl)-2-phenylpent-4-en-2-ol

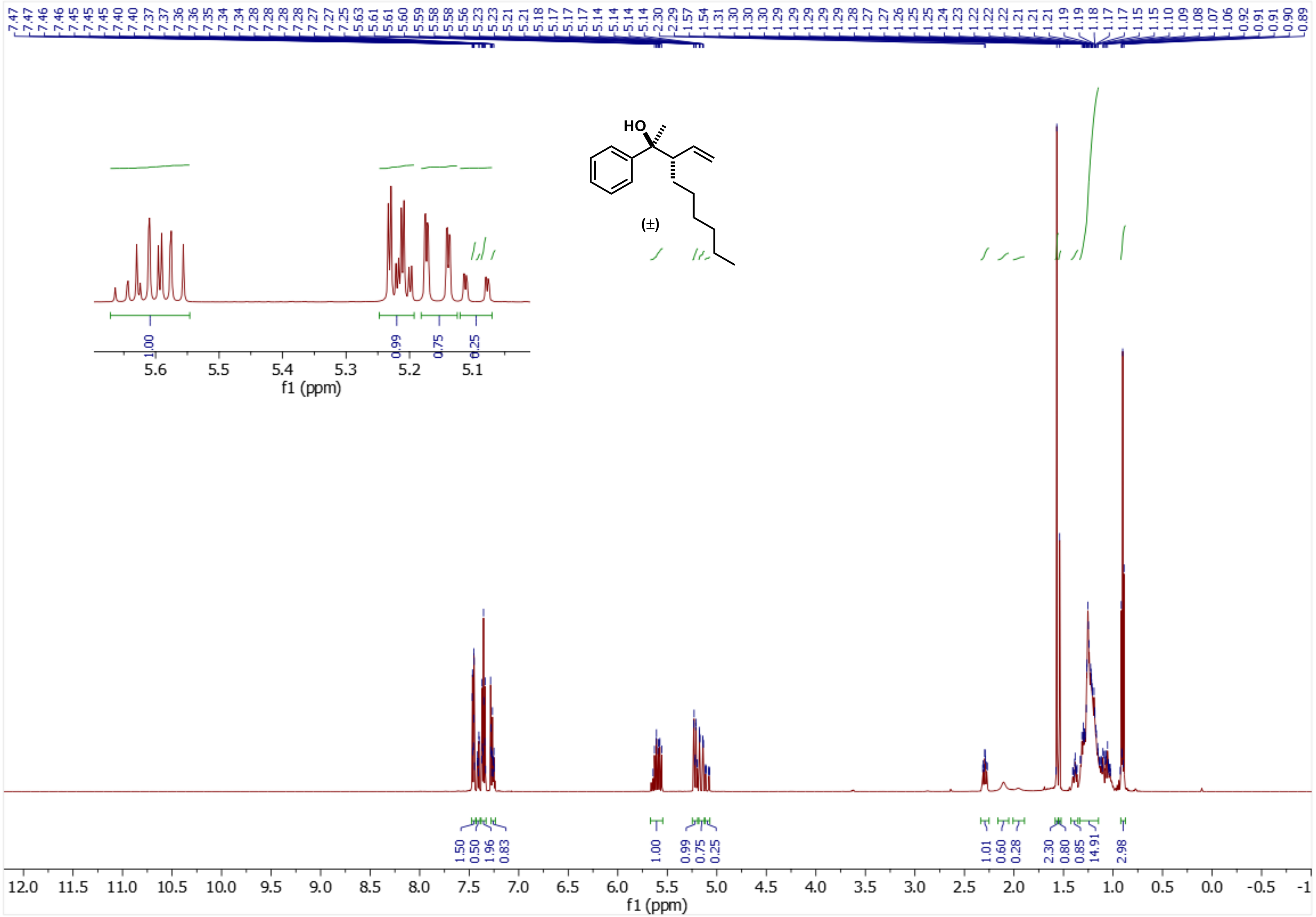

¹H NMR (400 MHz, $CDCl_3$) Spectra of (2*SR*,3*RS*)-3-hexyl-2-phenylpent-4-en-2-ol

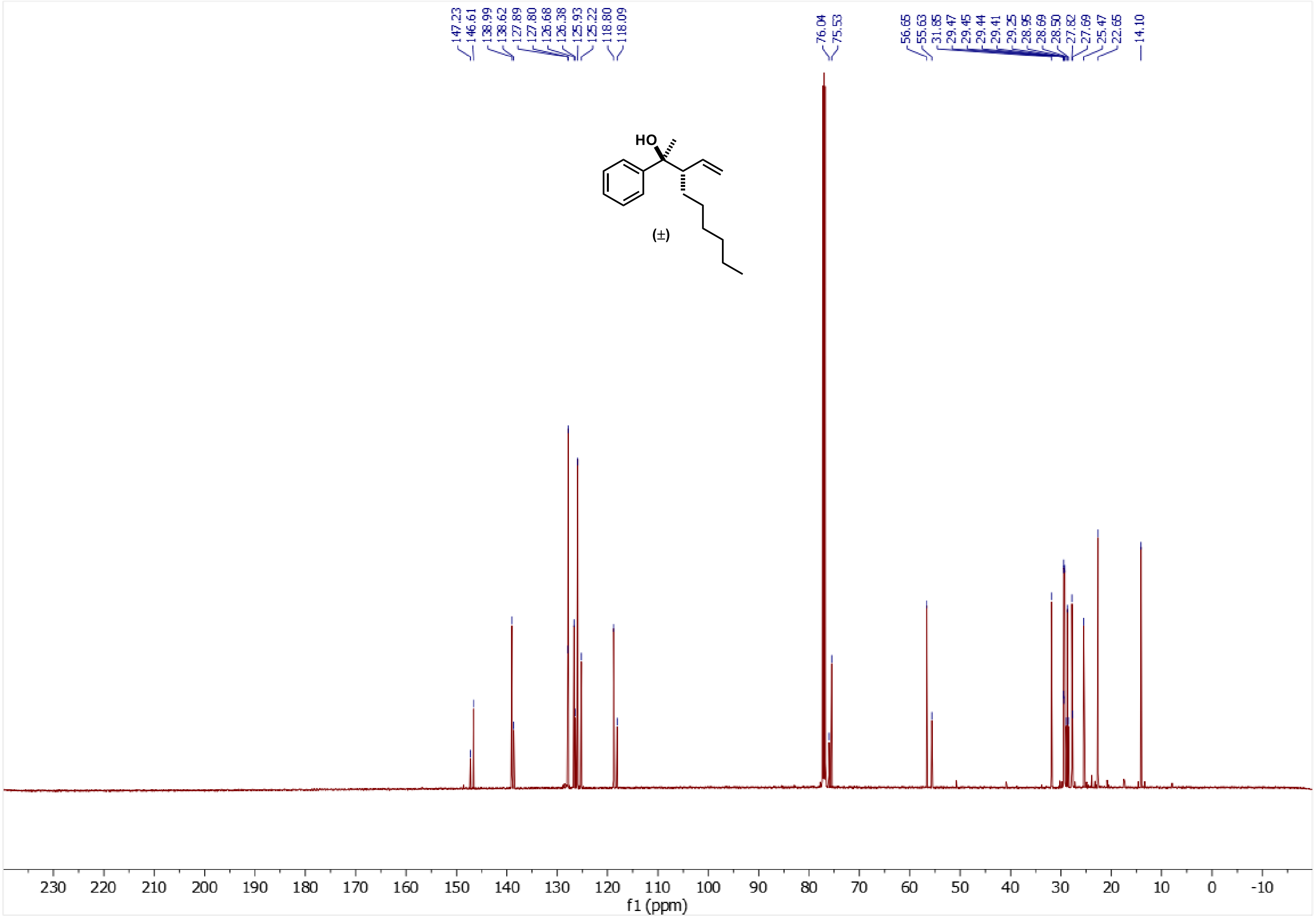

^{13}C NMR (101 MHz, $CDCl_3$) Spectra of (2*SR*,3*RS*)-3-hexyl-2-phenylpent-4-en-2-ol

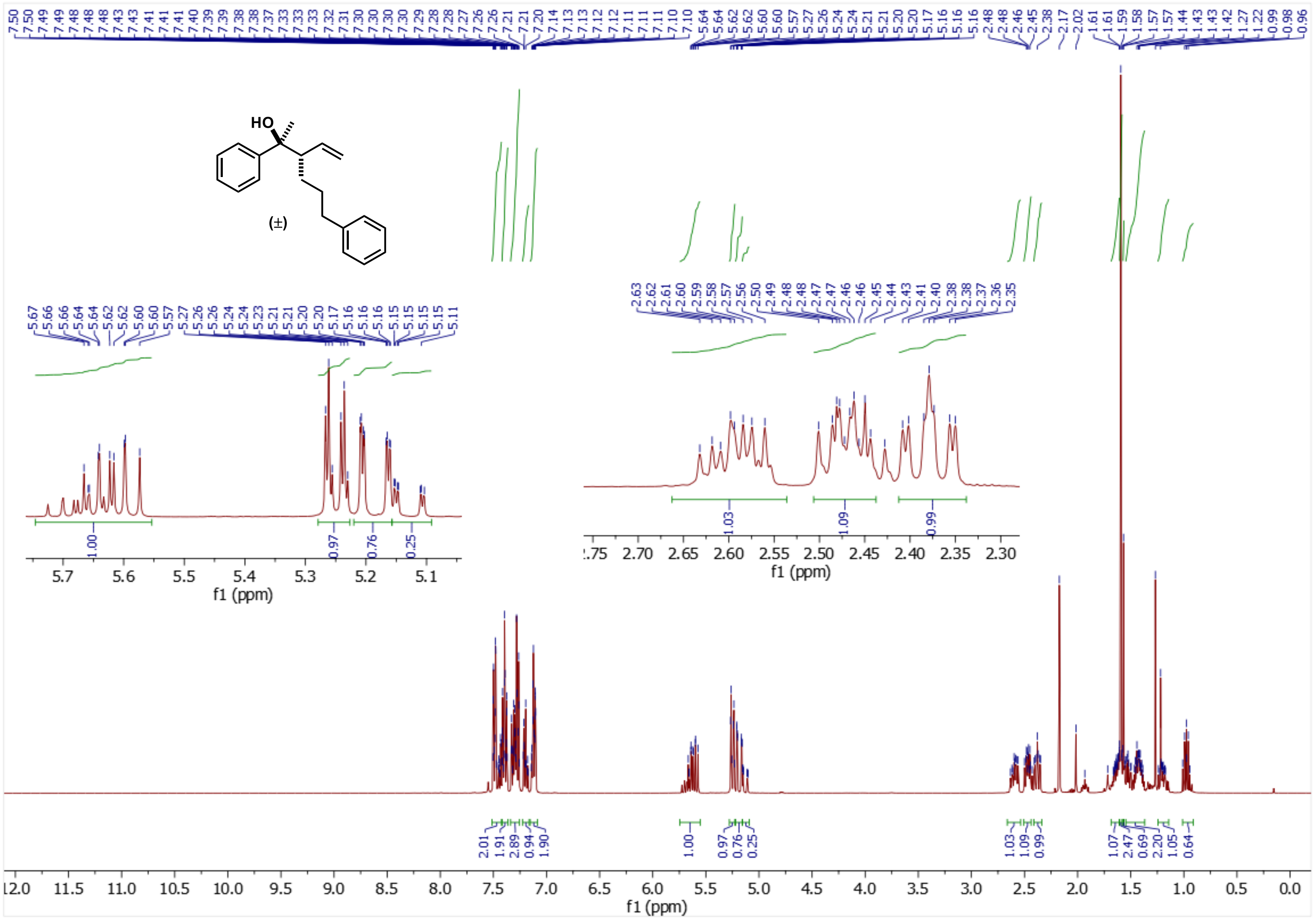

¹H NMR (600 MHz, $CDCl_3$) Spectra of (2*SR*,3*RS*)-3-(1-propyl-3-phenyl)-2-phenylpent-4-en-2-ol

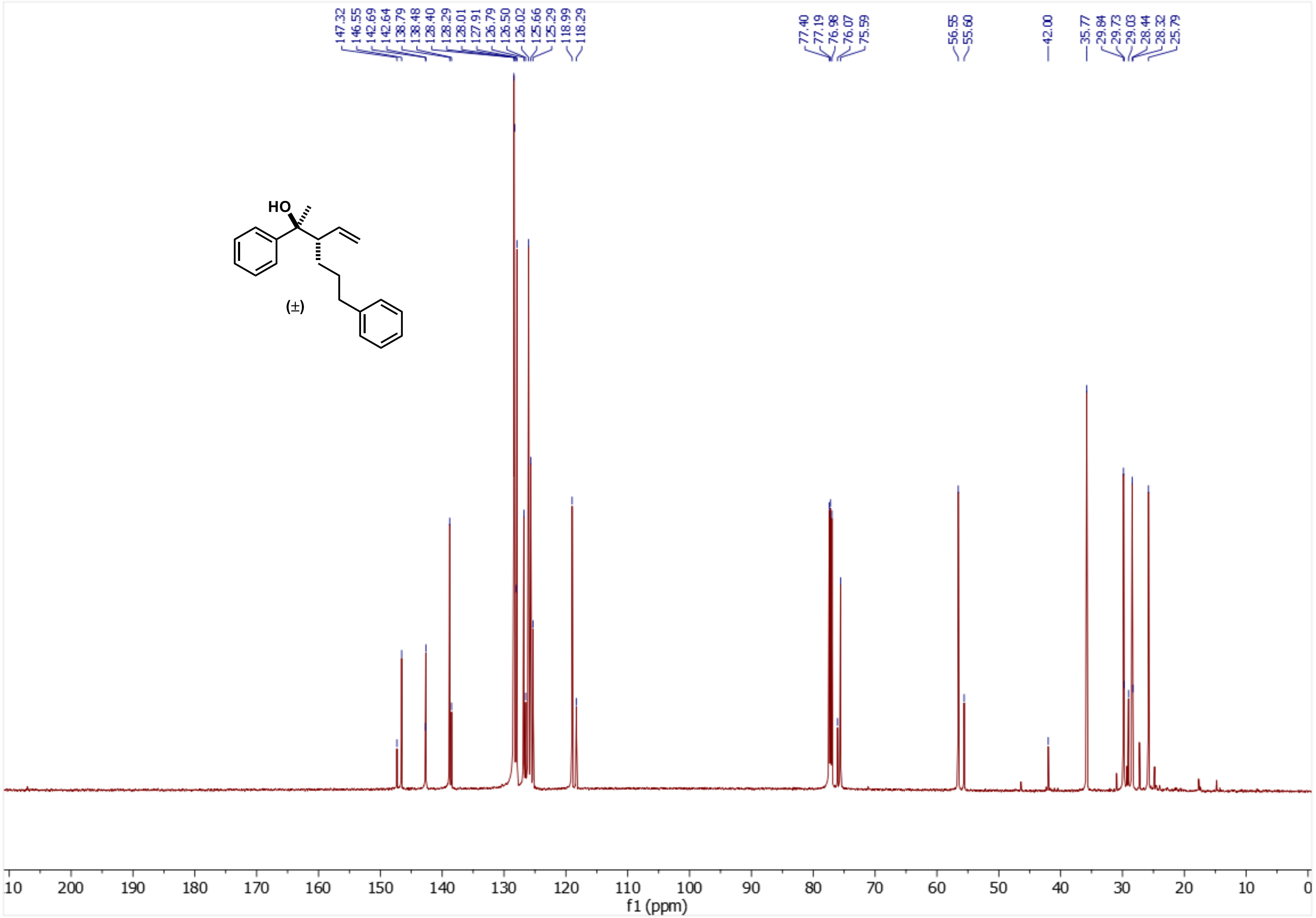

^{13}C NMR (151 MHz, $CDCl_3$) Spectra of (2*SR*,3*RS*)-3-(1-Propyl-3-phenyl)-2-phenylpent-4-en-2-ol

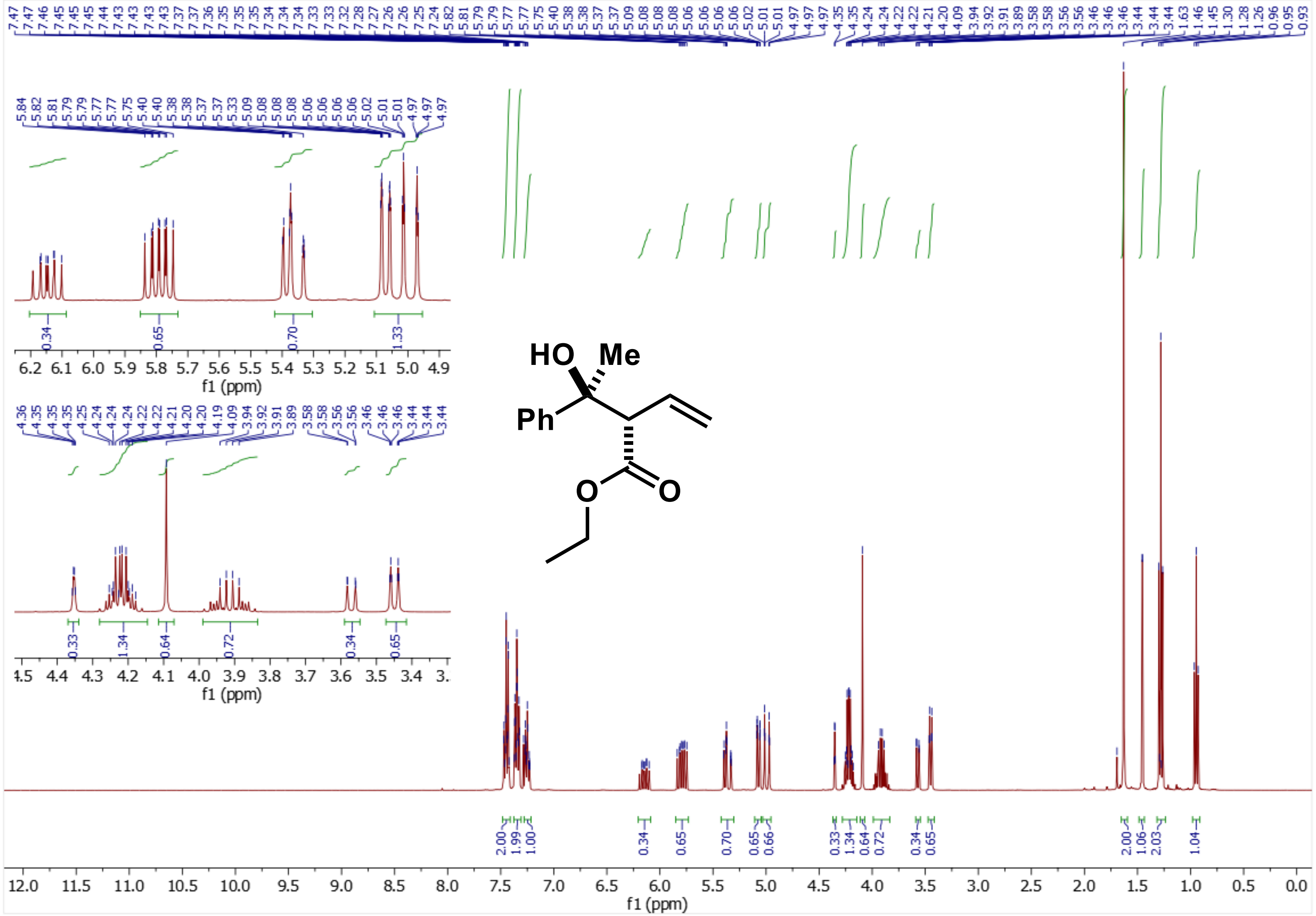

^{1}H NMR (600 MHz, $CDCl_3$) Spectra of (3*SR*,4*RS*)-ethyl-3-hydorxy-3-phenyl-2-vinylbutyrate

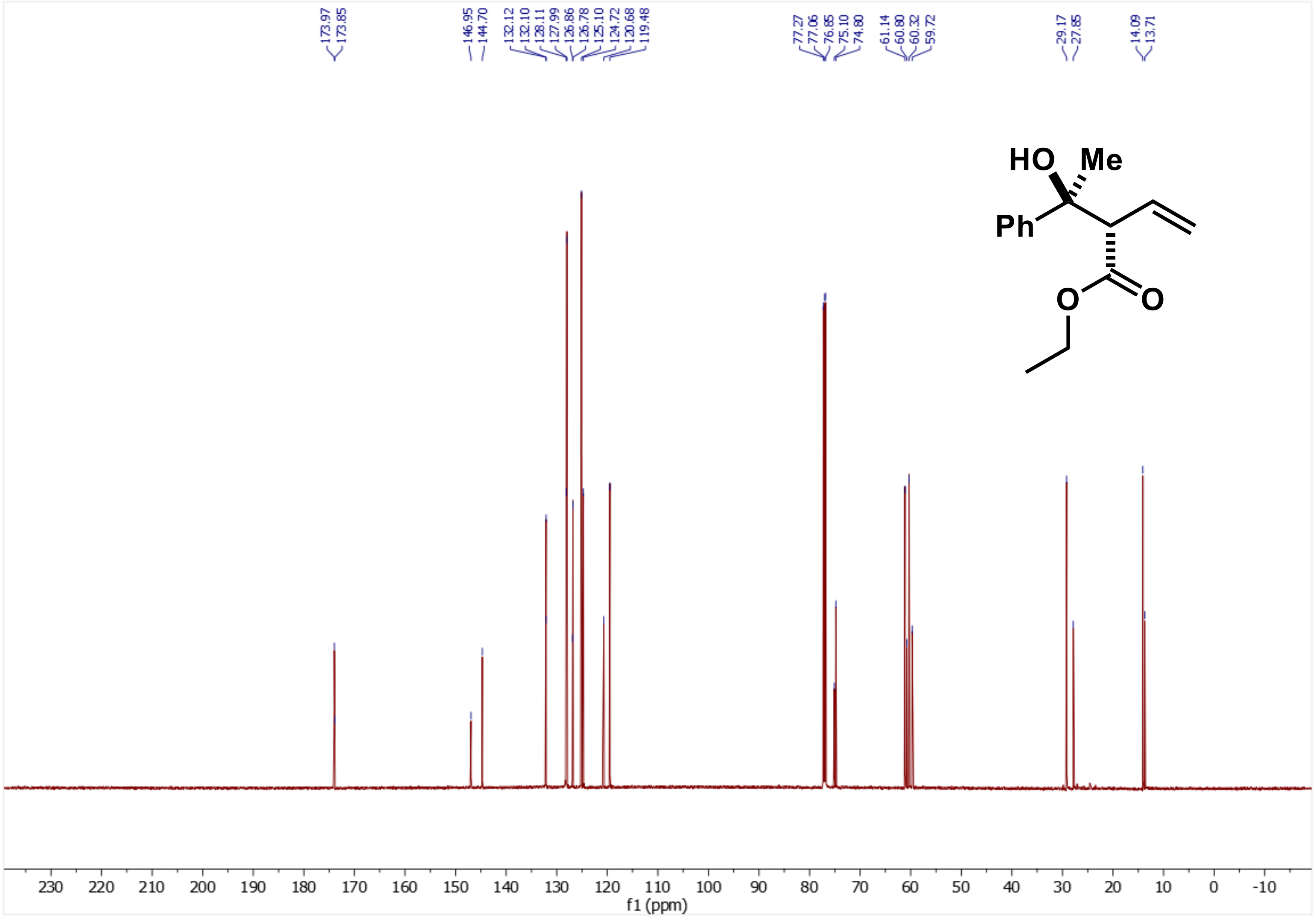

[1]**H NMR** (600 MHz, $CDCl_3$) Spectra of (3*SR*,4*RS*)-ethyl-3-hydorxy-3-phenyl-2-vinylbutyrate

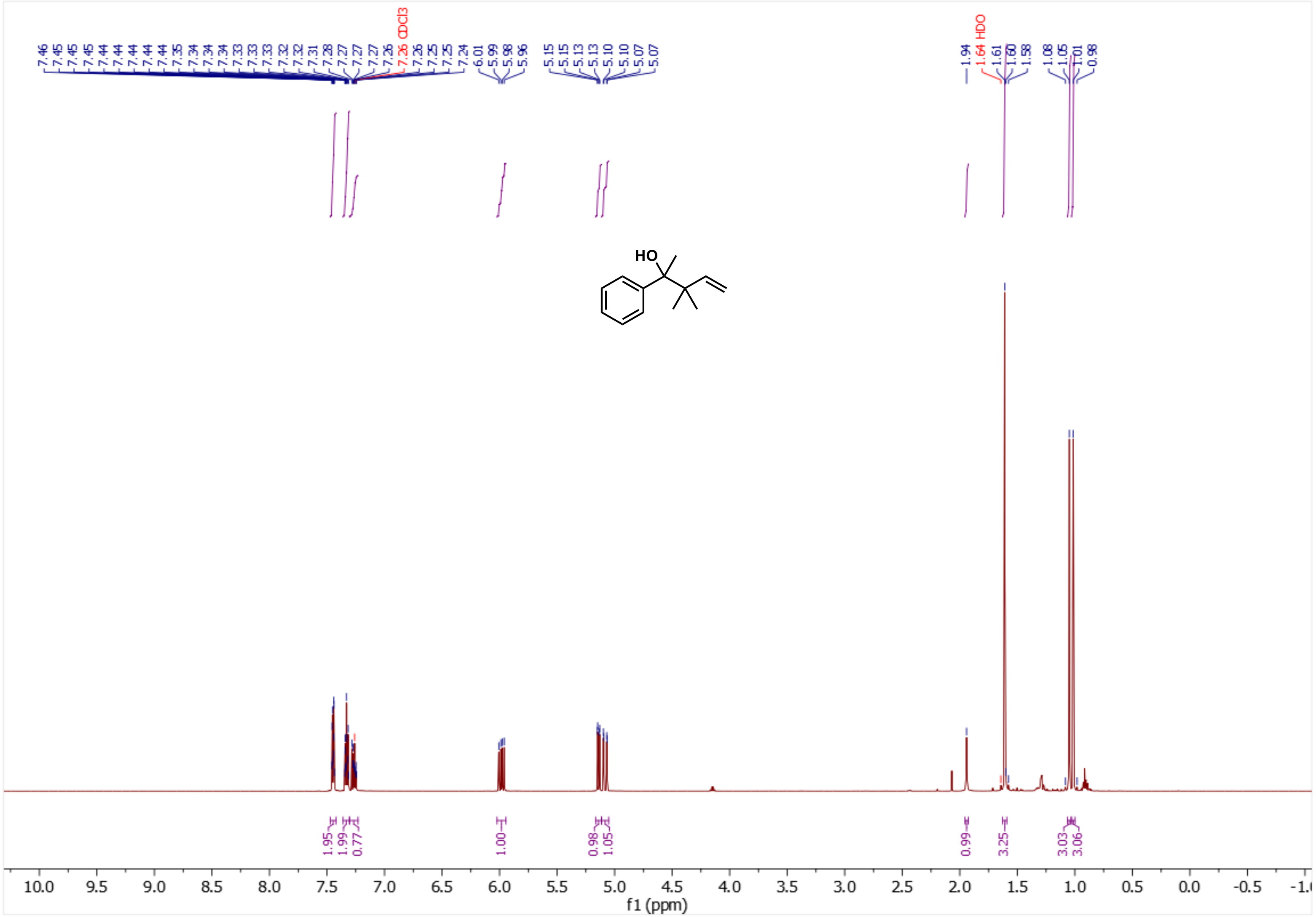

^{1}H NMR (600 MHz, $CDCl_3$) Spectra of 3,3-dimethyl-2-phenylpent-4-en-2-ol

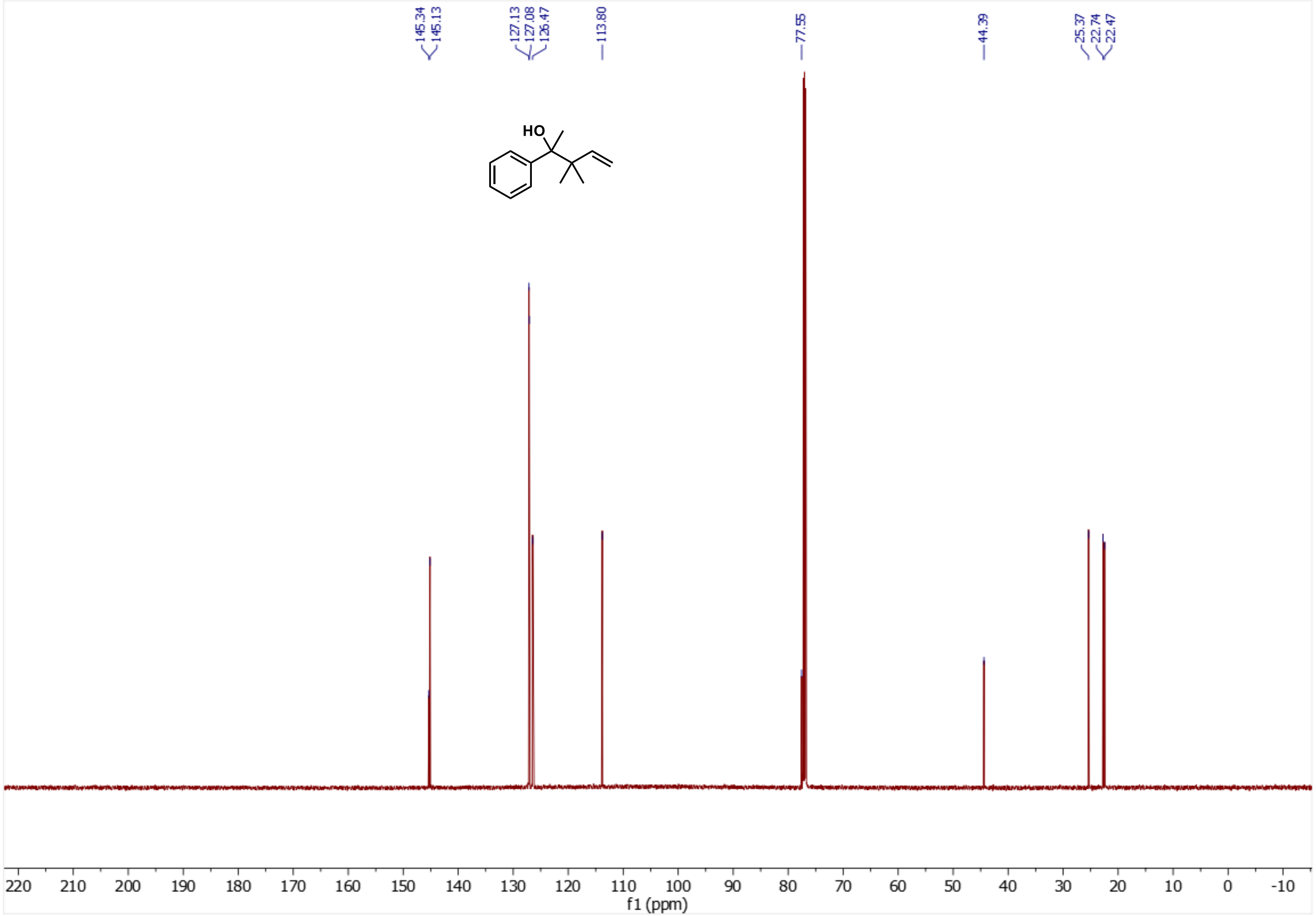

^{13}C NMR (151 MHz, $CDCl_3$) Spectra of 3,3-dimethyl-2-phenylpent-4-en-2-ol

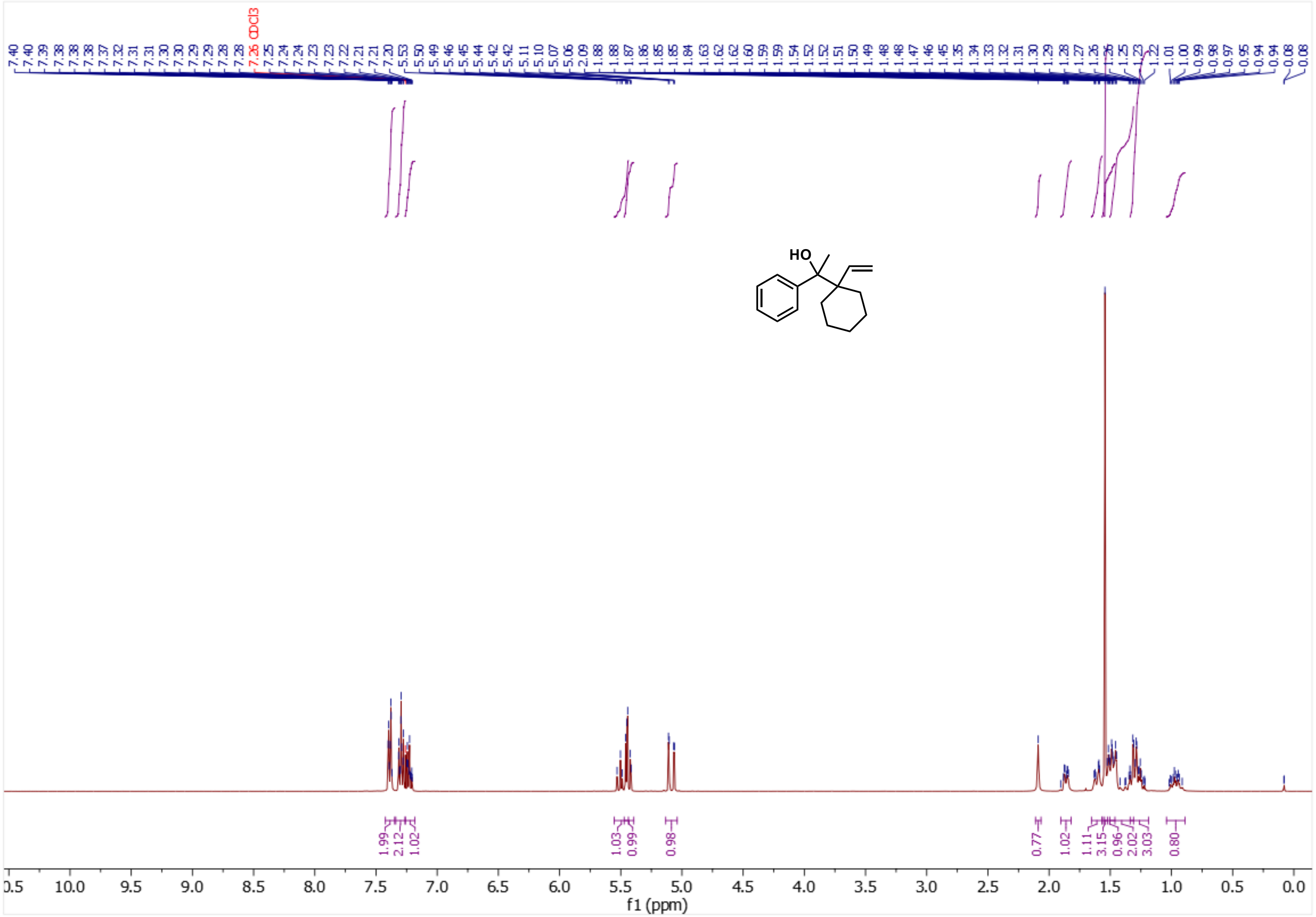

¹H NMR (400 MHz, $CDCl_3$) Spectra of 1-(1-ethenylcyclohexyl)-1-phenylethan-1-ol

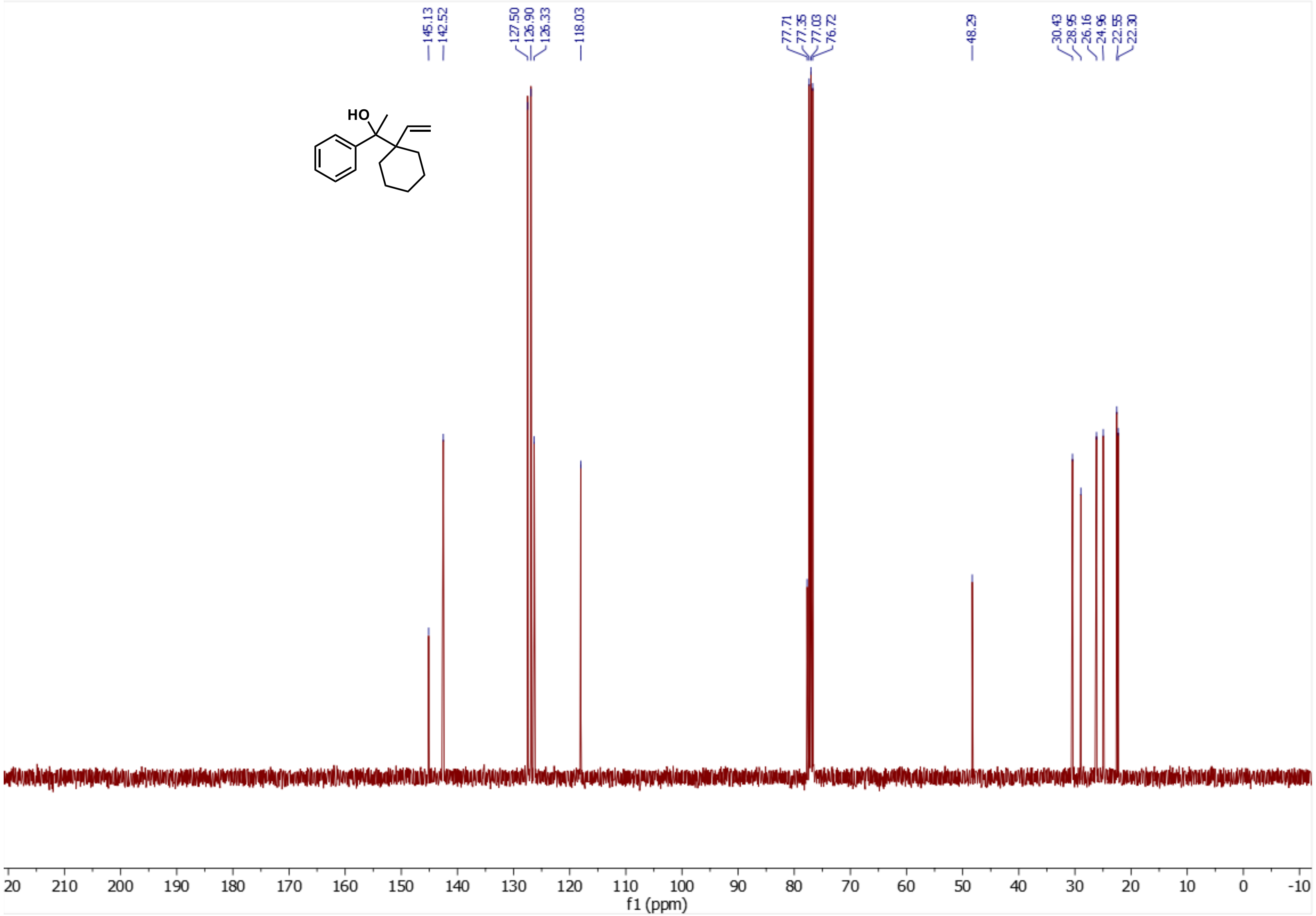

^{13}C NMR (101 MHz, $CDCl_3$) Spectra of 1-(1-ethenylcyclohexyl)-1-phenylethan-1-ol

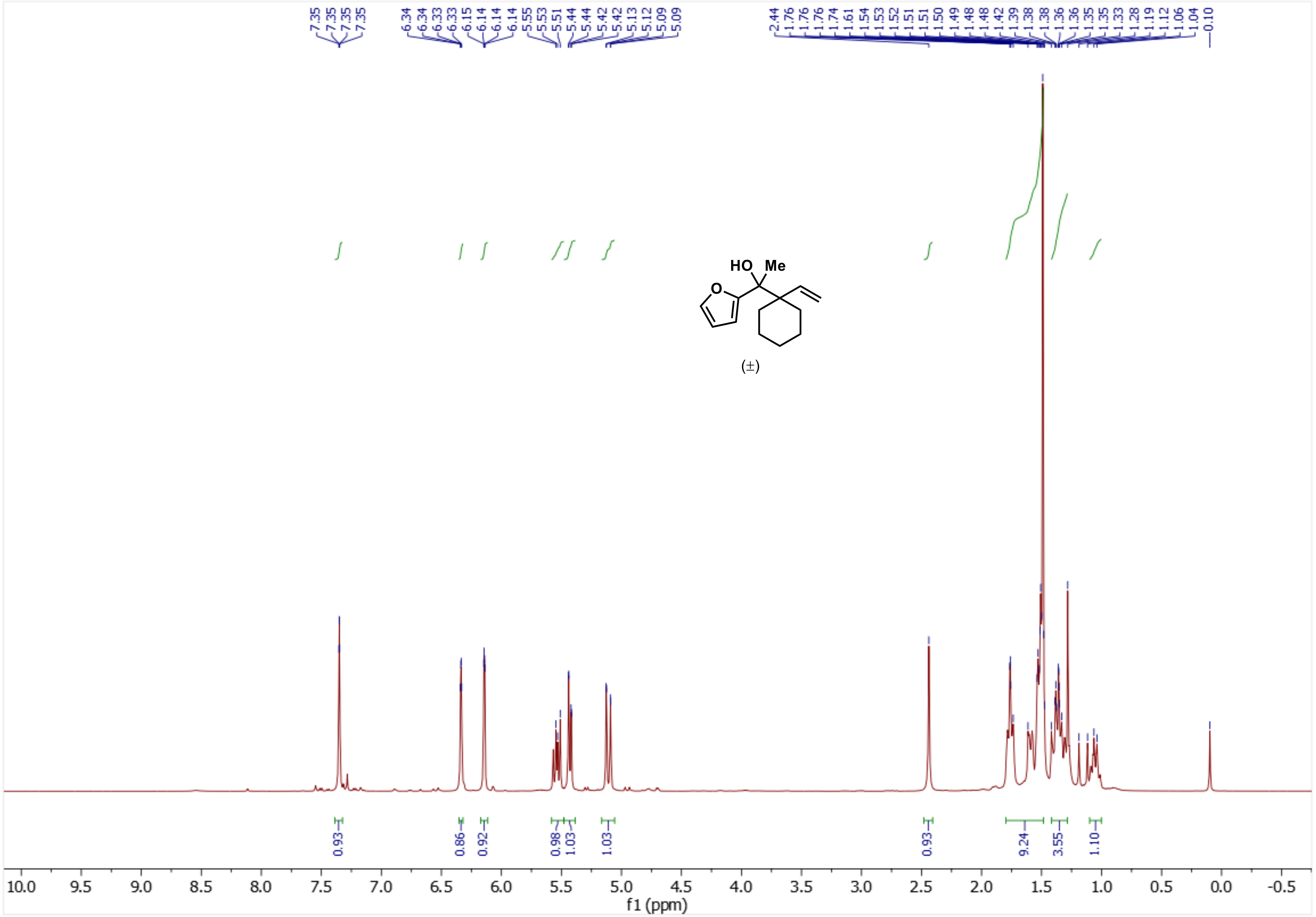

^{1}H NMR (500 MHz, $CDCl_3$) Spectra of 1-(1-ethenylcyclohexyl)-1-(2-furyl)ethan-1-ol

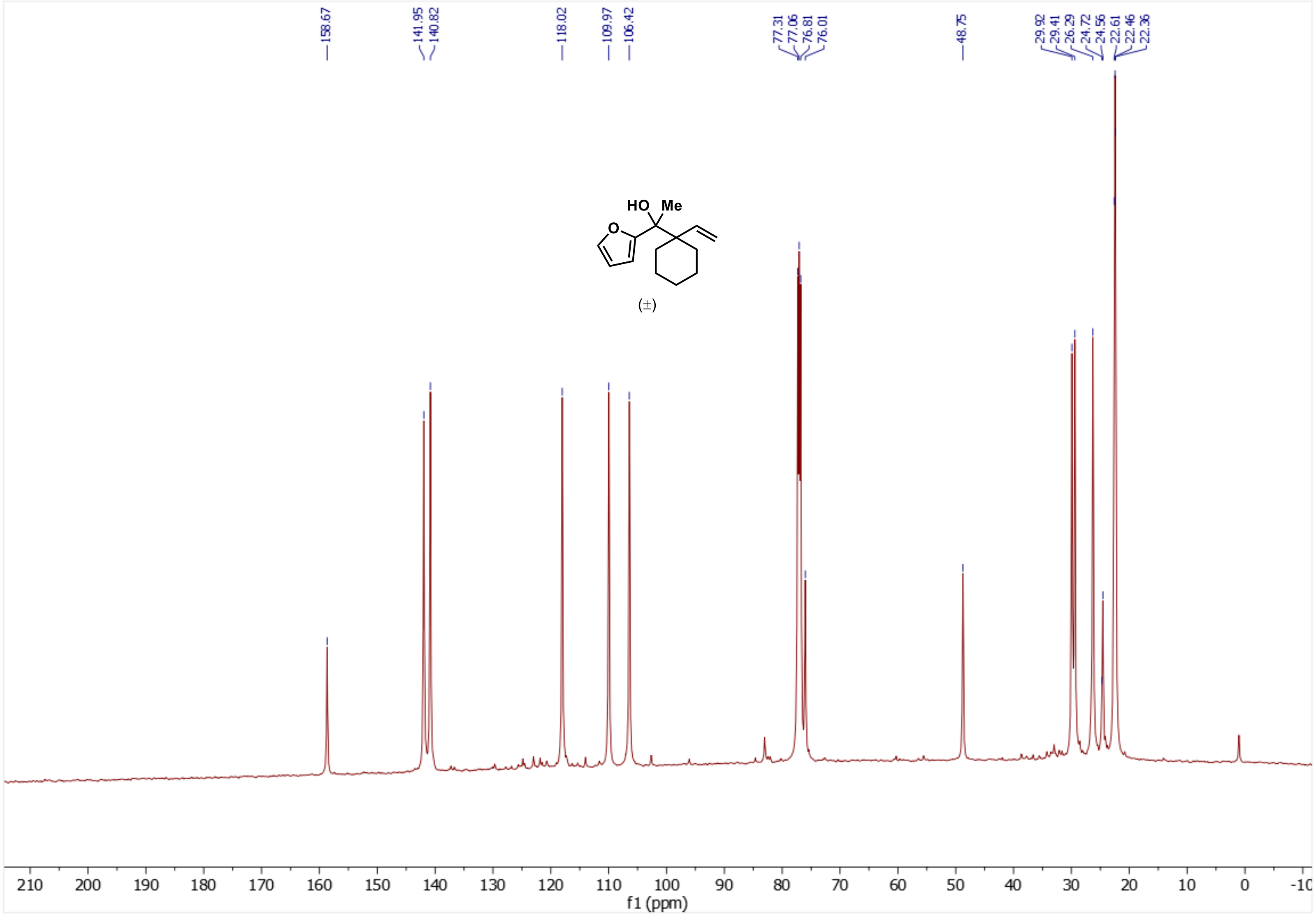

^{13}C NMR (126 MHz, $CDCl_3$) Spectra of 1-(1-ethenylcyclohexyl)-1-(2-furyl)ethan-1-ol

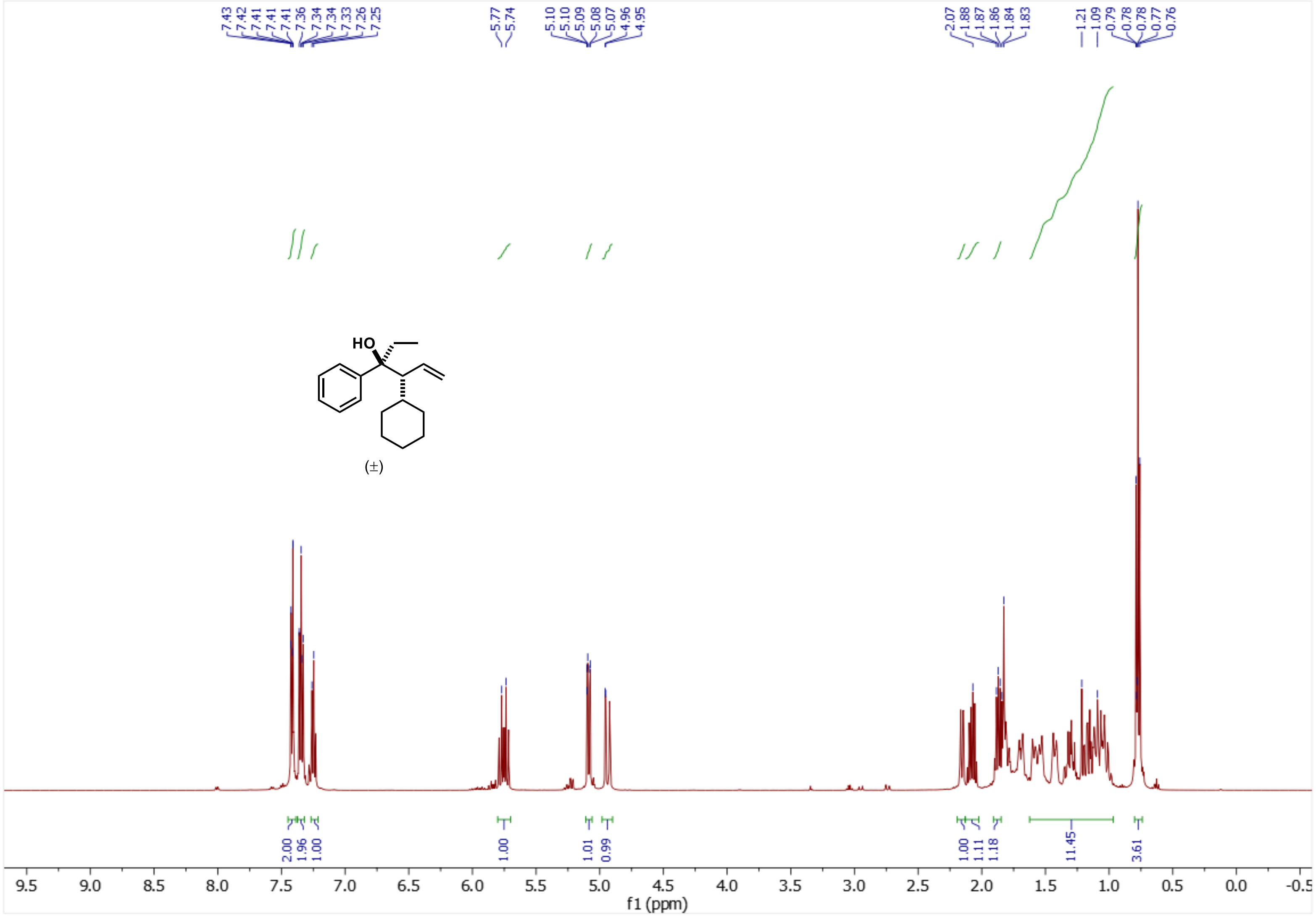

^{1}H NMR (500 MHz, $CDCl_3$) Spectra of (3*RS*,4*SR*)-4-Cyclohexyl-3-phenylhex-5-en-3-ol

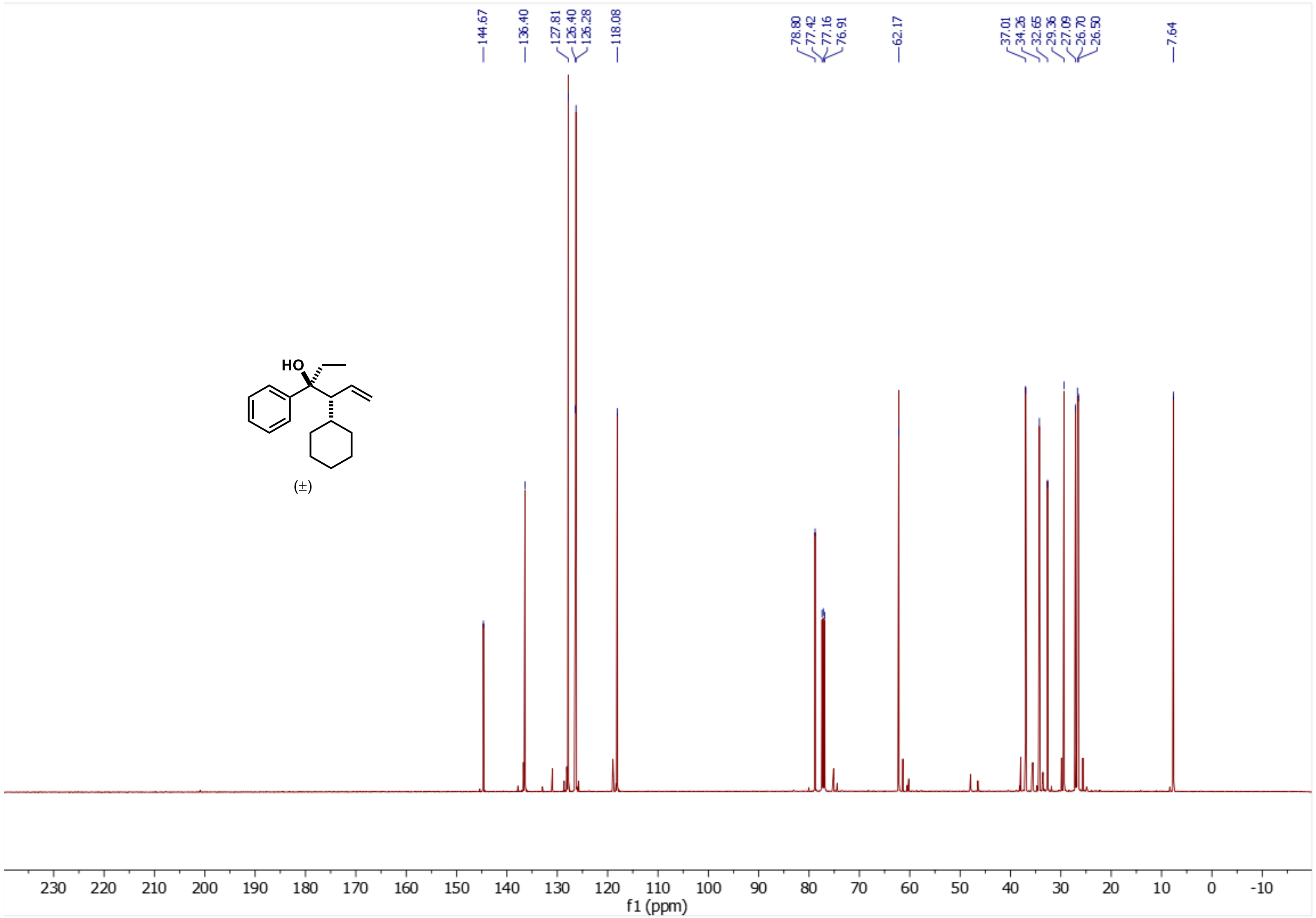

^{13}C NMR (126 MHz, $CDCl_3$) Spectra of (3*RS*,4*SR*)-4-cyclohexyl-3-phenylhex-5-en-3-ol

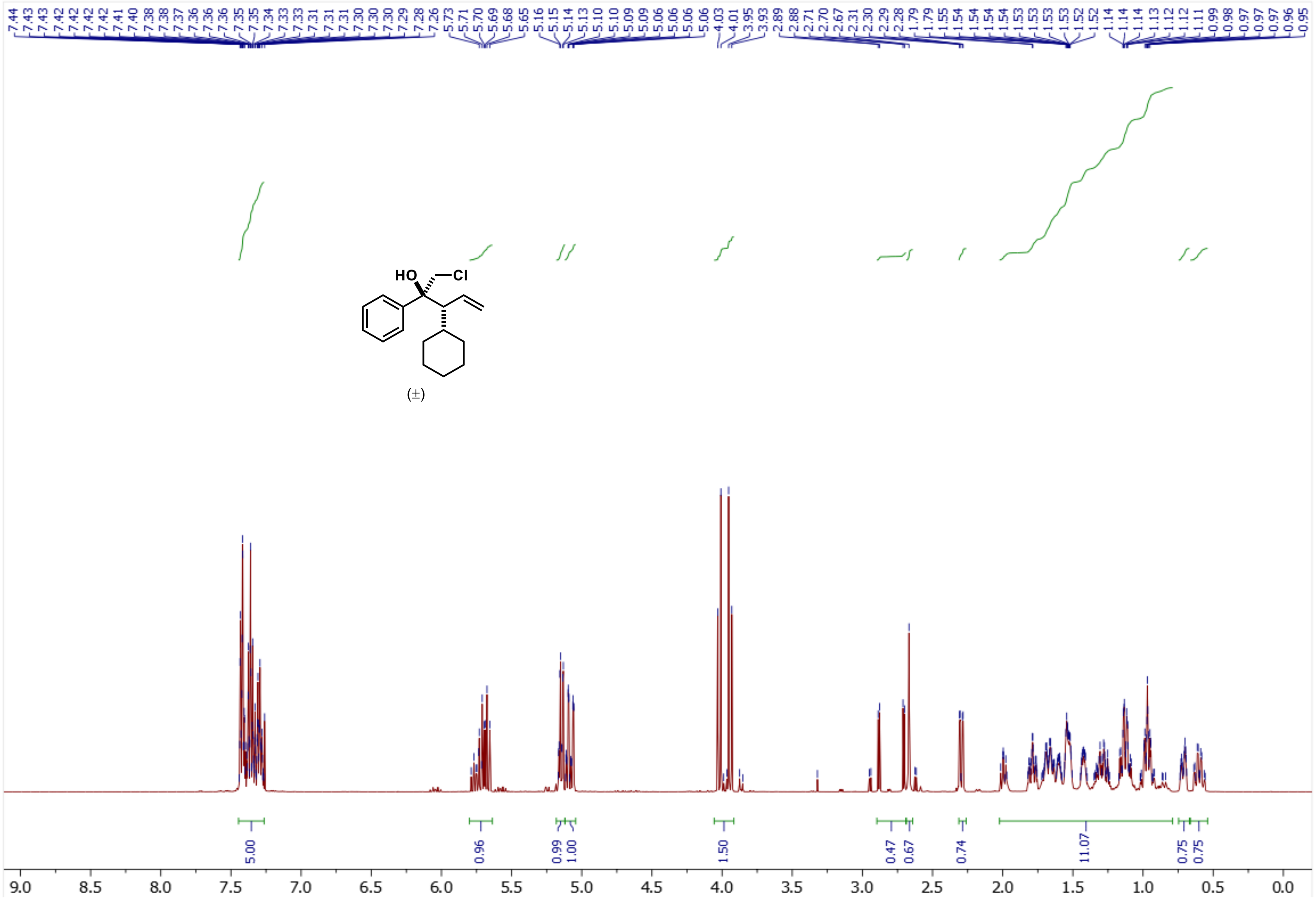

^{1}H NMR (500 MHz, $CDCl_3$) Spectra of (2*SR*,3*RS*)-3-cyclohexyl-1-chloro-2-phenylpent-4-en-2-ol

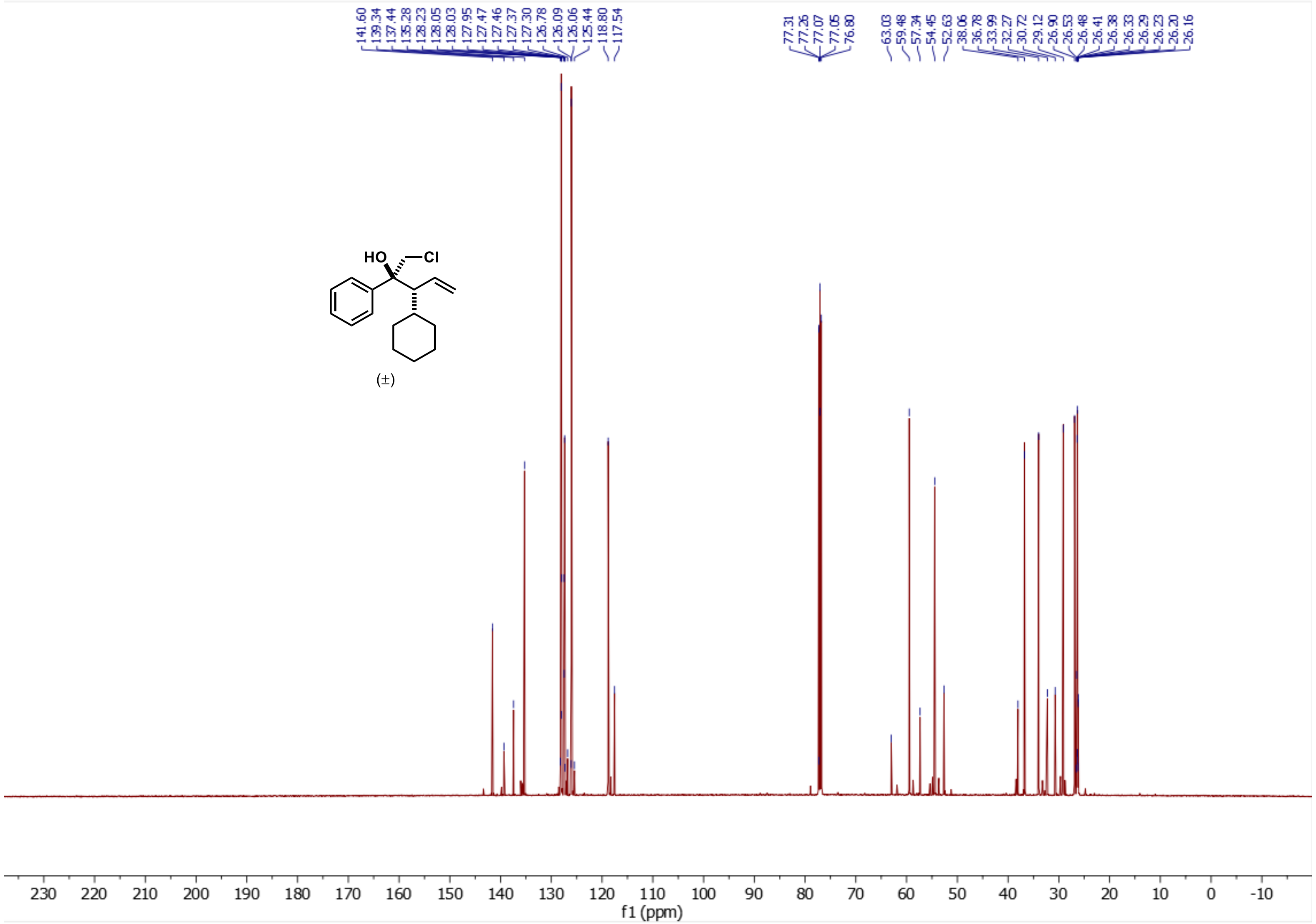

^{13}C NMR (126 MHz, $CDCl_3$) Spectra of (2*SR*,3*RS*)-3-cyclohexyl-1-chloro-2-phenylpent-4-en-2-ol

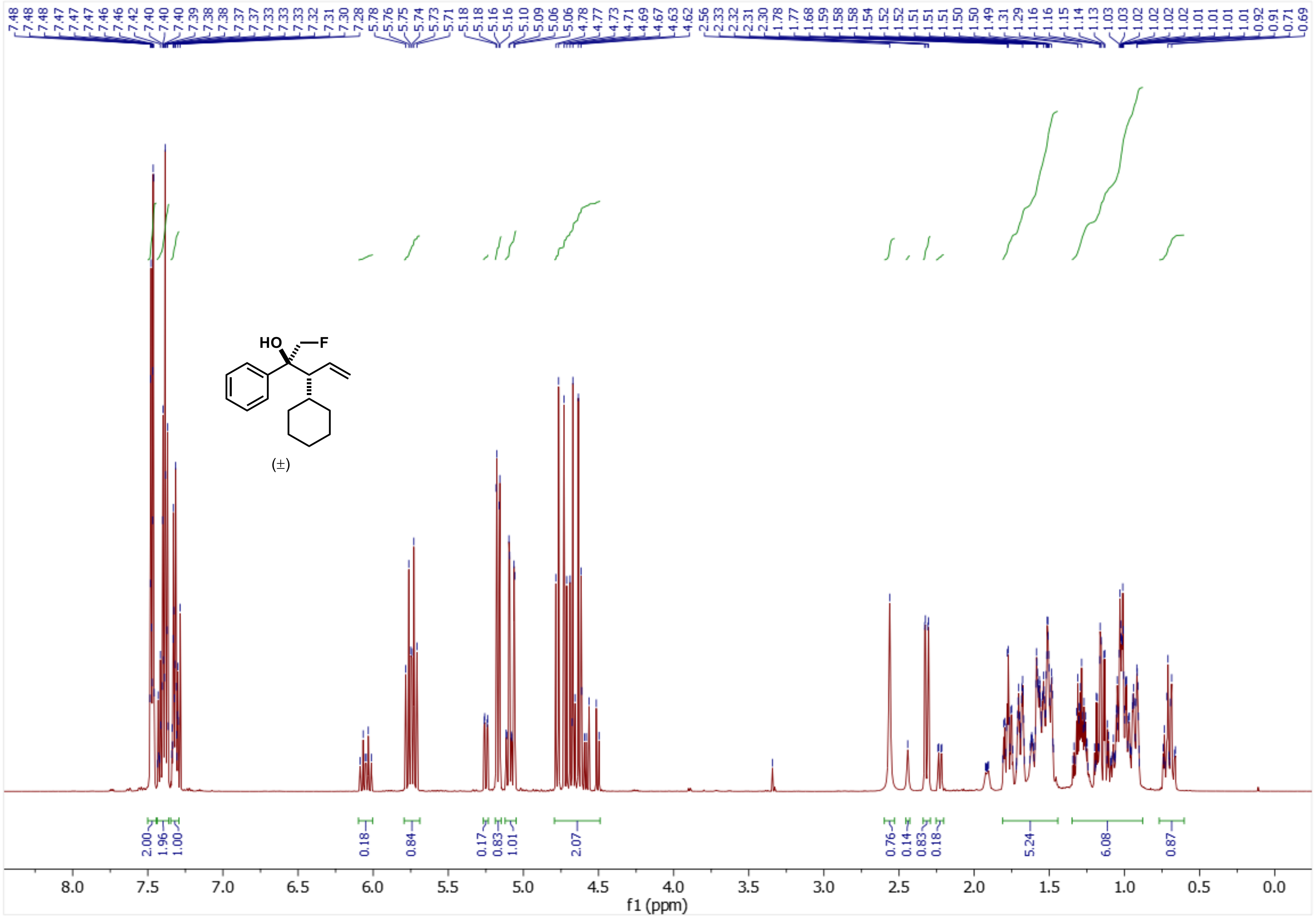

^{1}H NMR (500 MHz, $CDCl_3$) Spectra of (2*SR*,3*RS*)-3-cyclohexyl-1-fluoro-2-phenylpent-4-en-2-ol

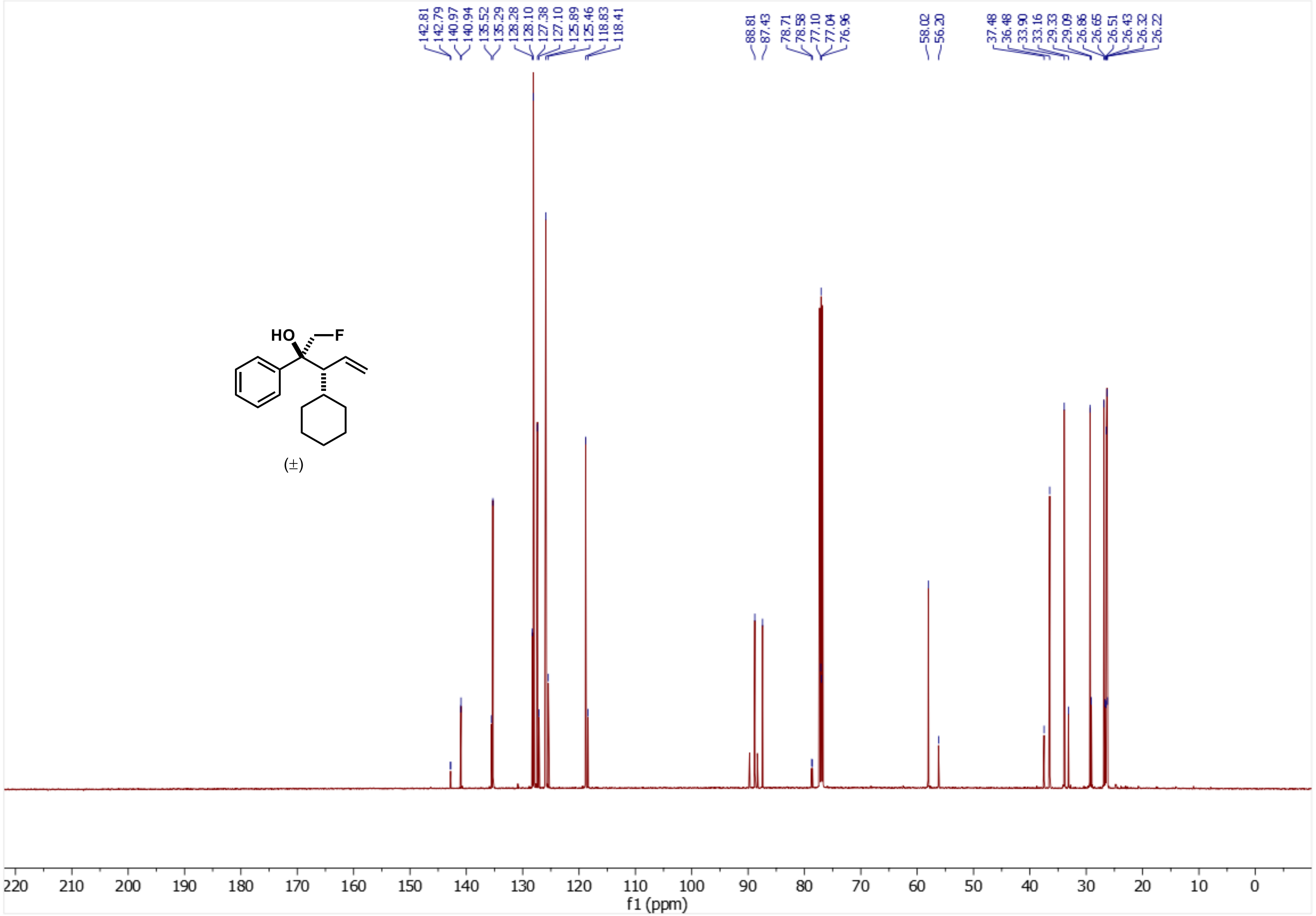

^{13}C NMR (126 MHz, $CDCl_3$) Spectra of (2*SR*,3*RS*)-3-cyclohexyl-1-fluoro-2-phenylpent-4-en-2-ol

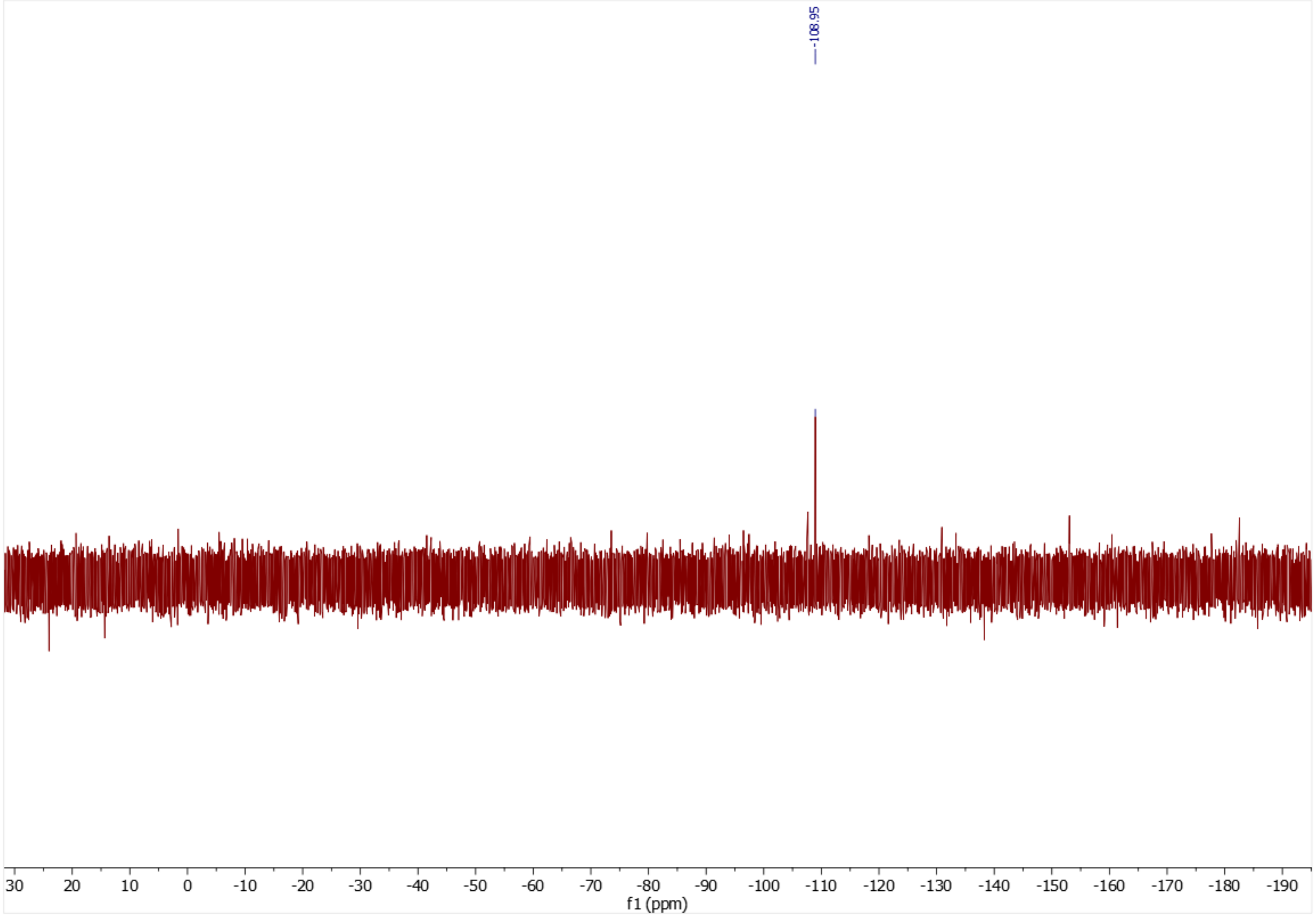

^{19}F NMR (376 MHz, $CDCl_3$) Spectra of (2*SR*,3*RS*)-3-cyclohexyl-1-fluoro-2-phenylpent-4-en-2-ol

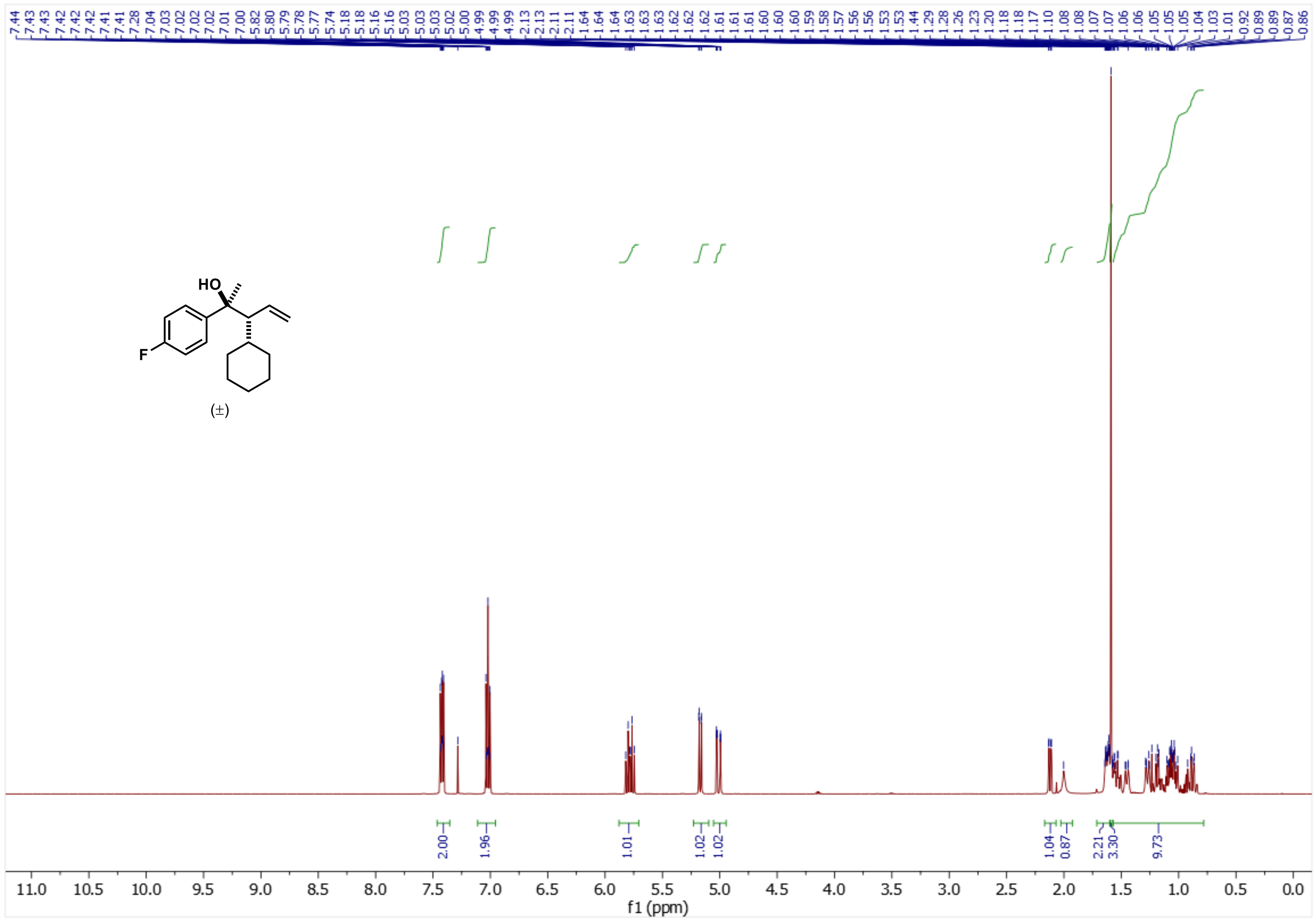

^{1}H NMR (500 MHz, $CDCl_3$) Spectra of (2*SR*,3*RS*)-3-cyclohexyl-2-(4-fluorophenyl)pent-4-en-2-ol

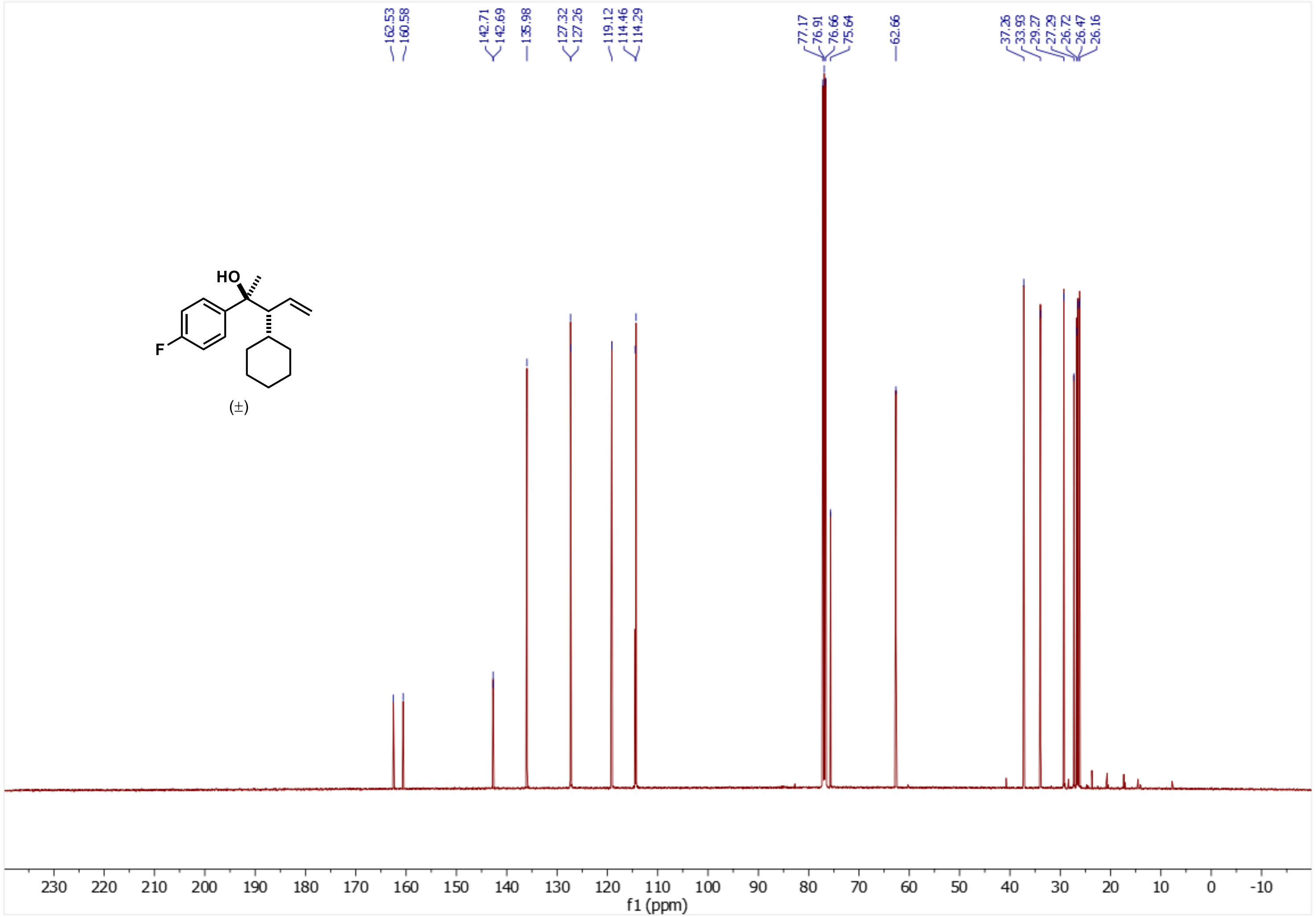

^{13}C NMR (126 MHz, $CDCl_3$) Spectra of (2*SR*,3*RS*)-3-cyclohexyl-2-(4-fluorophenyl)pent-4-en-2-ol

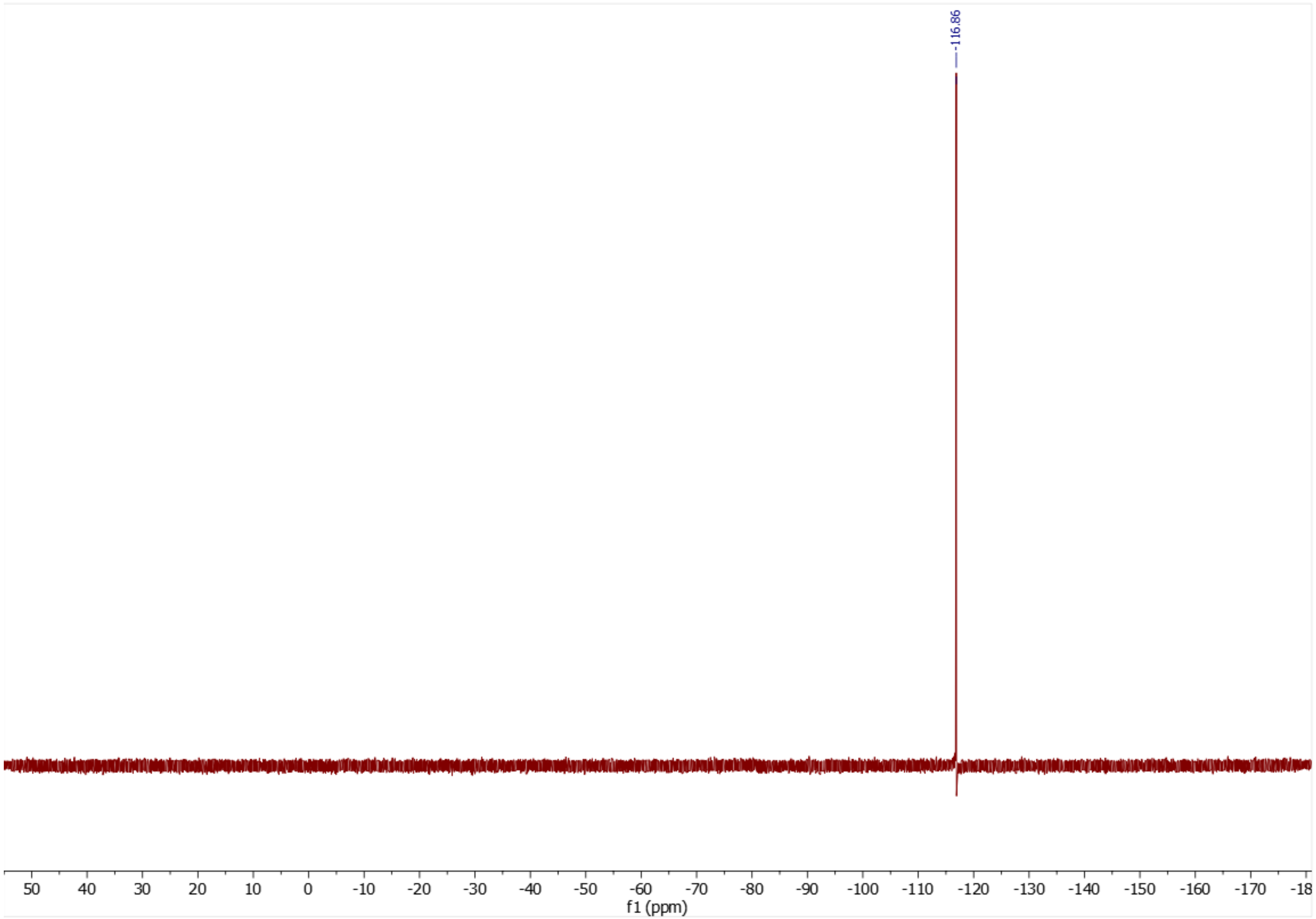

^{19}**F NMR** (471 MHz, $CDCl_3$) Spectra of (2*SR*,3*RS*)-3-cyclohexyl-2-(4-fluorophenyl)pent-4-en-2-ol

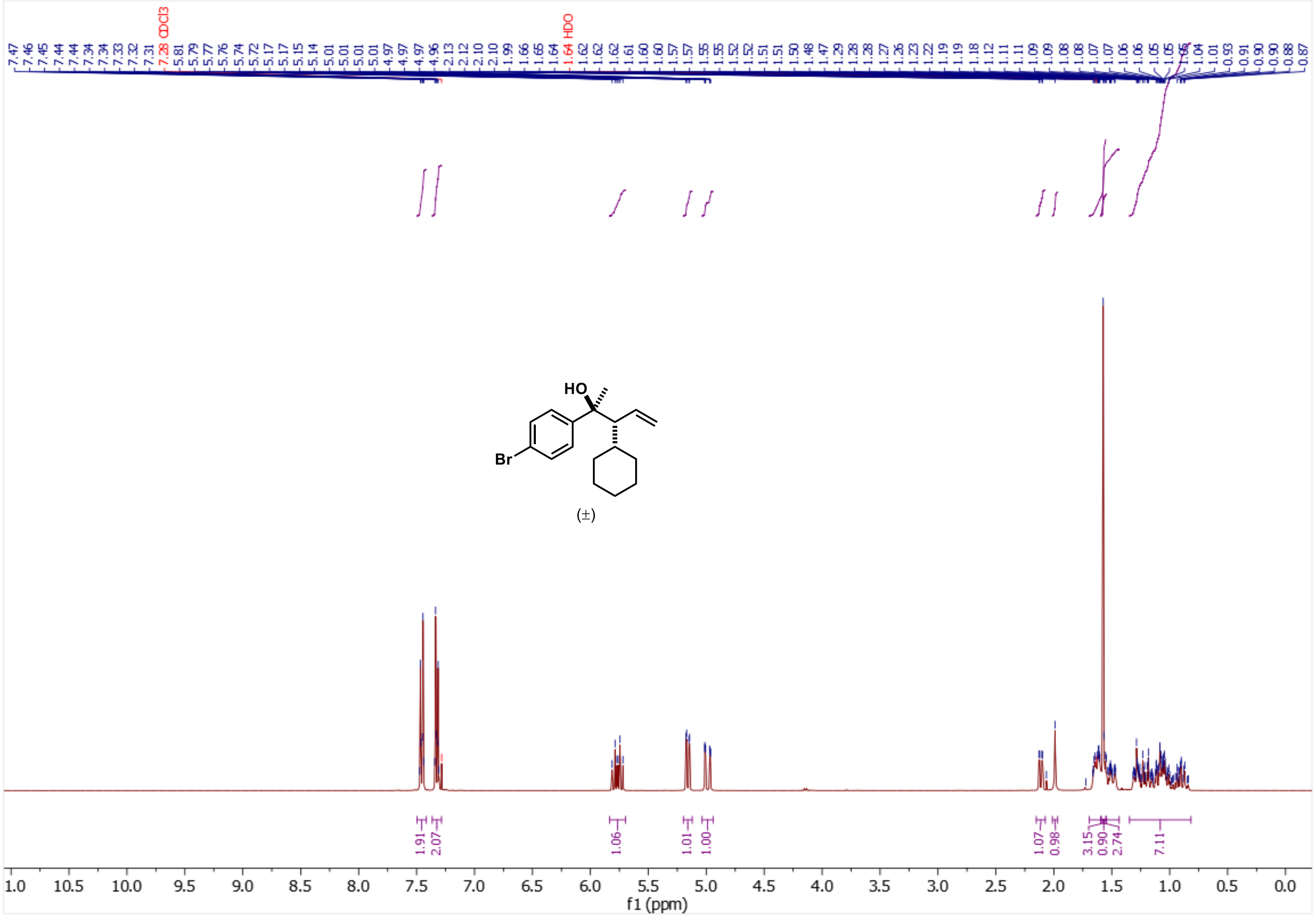

^{1}H NMR (500 MHz, $CDCl_3$) Spectra of (2*RS*,3*SR*)-3-cyclohexyl-2-(4-bromophenyl)pent-4-en-2-ol

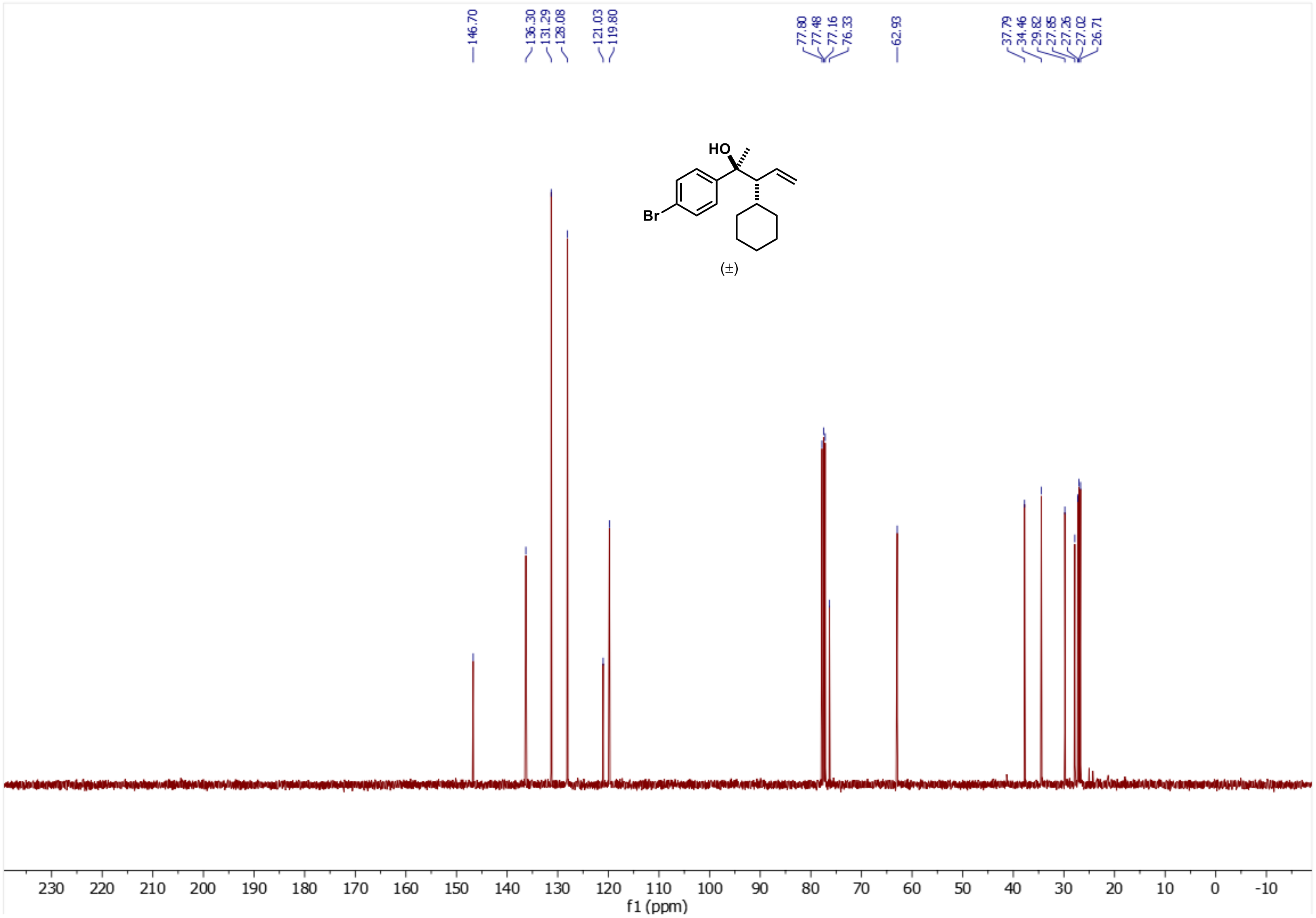

^{13}C NMR (126 MHz, $CDCl_3$) Spectra of (2*RS*,3*SR*)-3-cyclohexyl-2-(4-bromophenyl)pent-4-en-2-ol

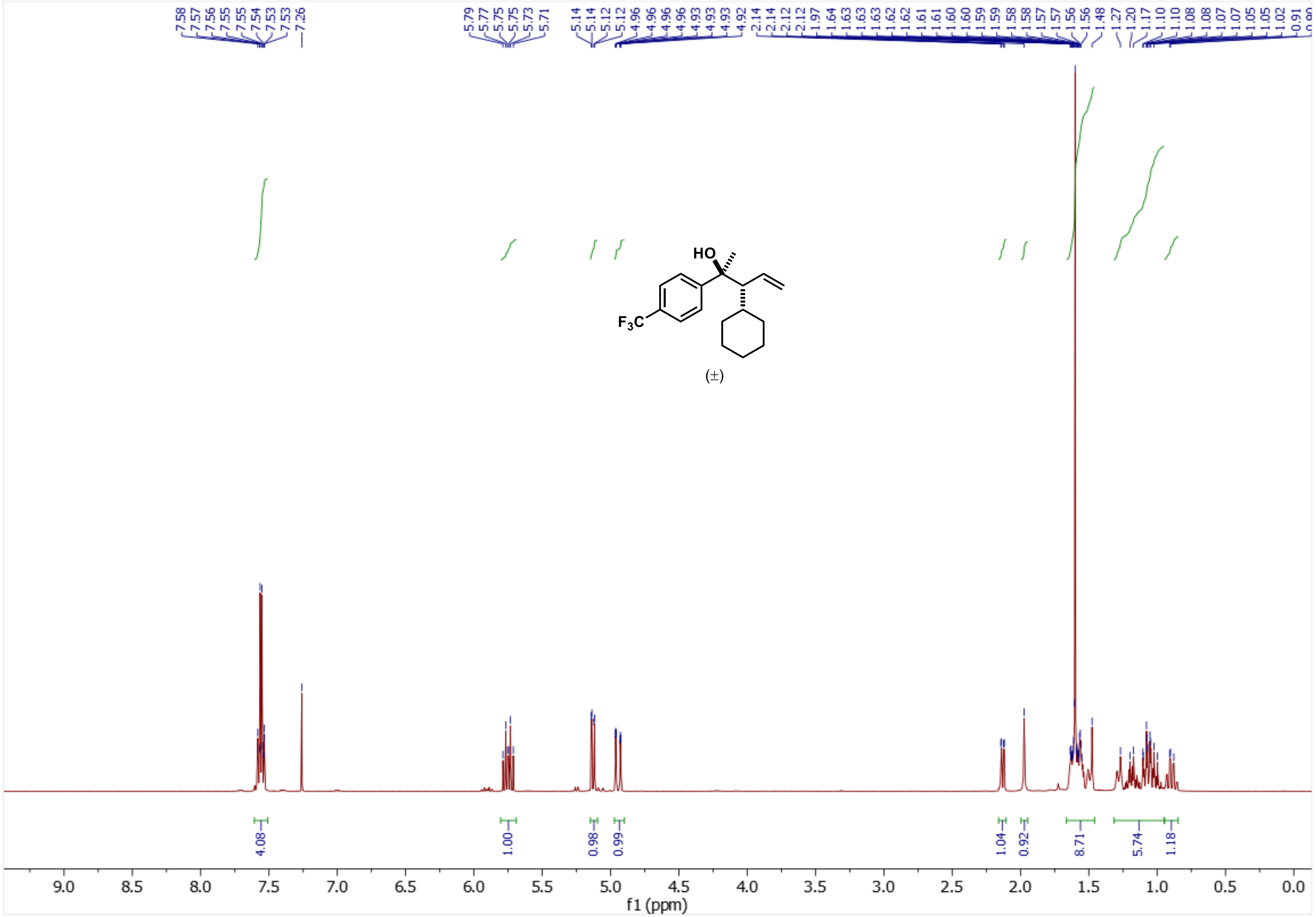

^{1}H NMR (500 MHz, $CDCl_3$) Spectra of (2*SR*,3*RS*)-3-cyclohexyl-2-(4-(trifluoromethyl)phenyl)pent-4-en-2-ol

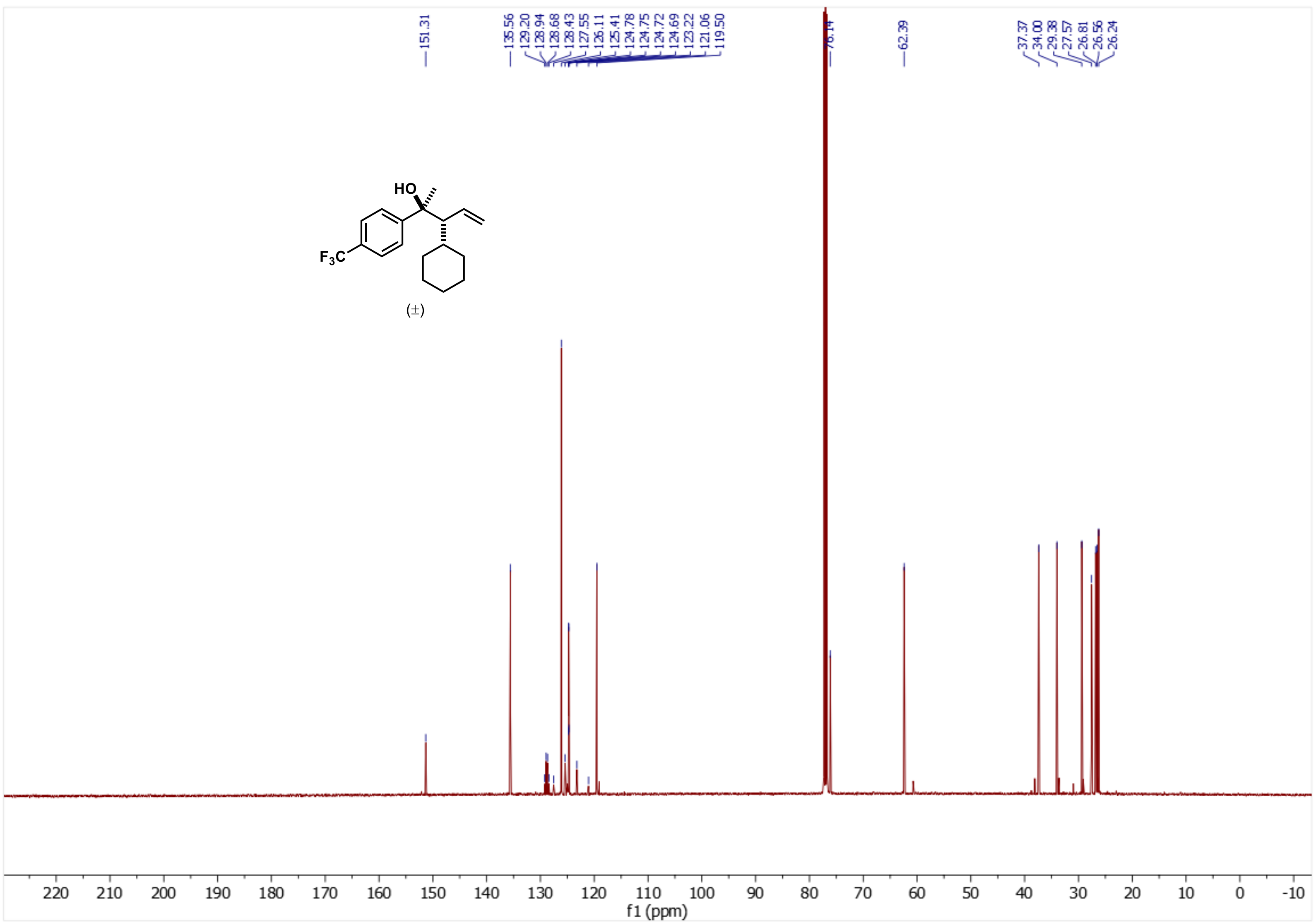

13C NMR (126 MHz, $CDCl_3$) Spectra of (2*SR*,3*RS*)-3-cyclohexyl-2-(4-(trifluoromethyl)phenyl)pent-4-en-2-ol

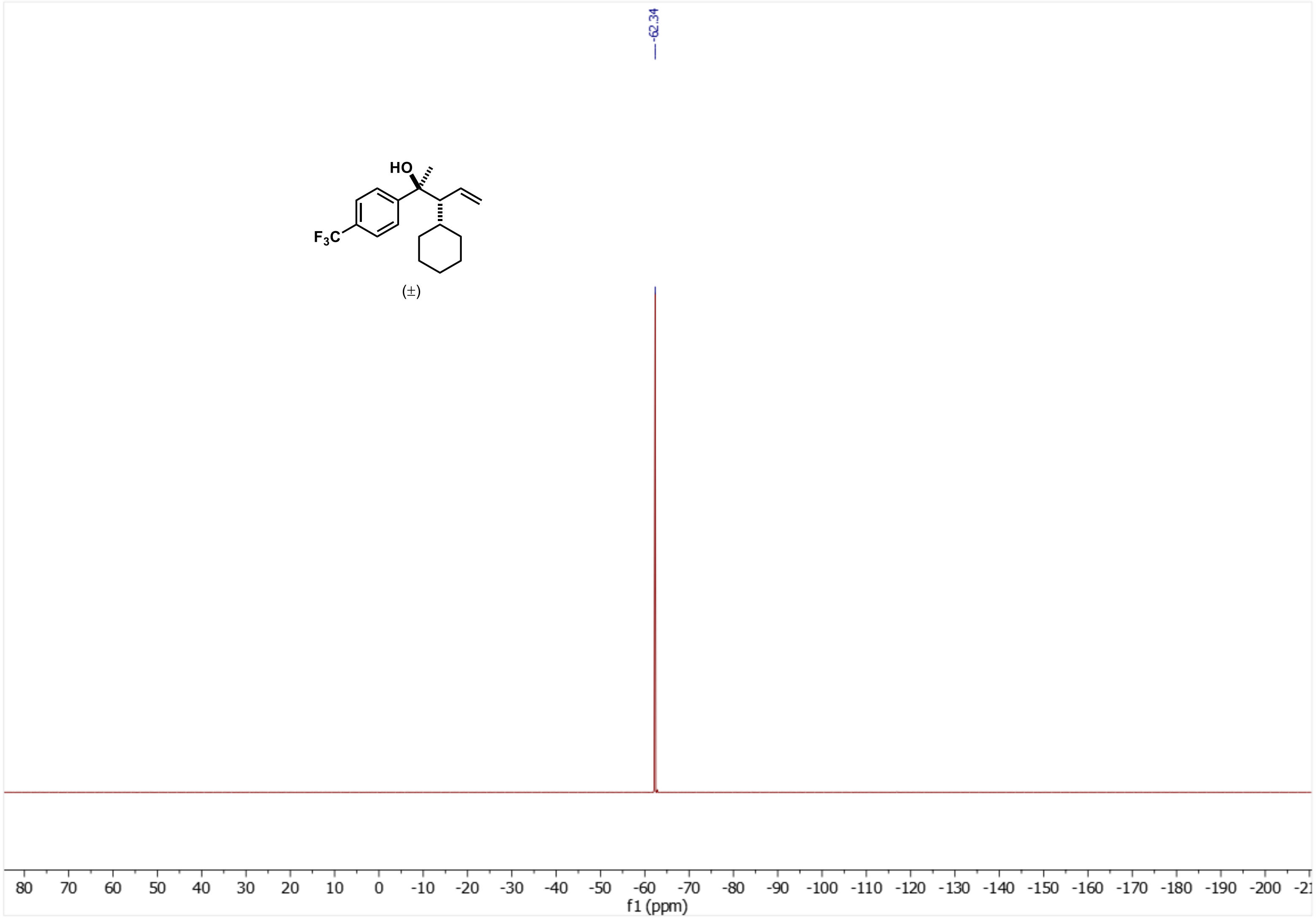

^{19}F NMR (376 MHz, $CDCl_3$) Spectra of (2*SR*,3*RS*)-3-cyclohexyl-2-(4-(trifluoromethyl)phenyl)pent-4-en-2-ol

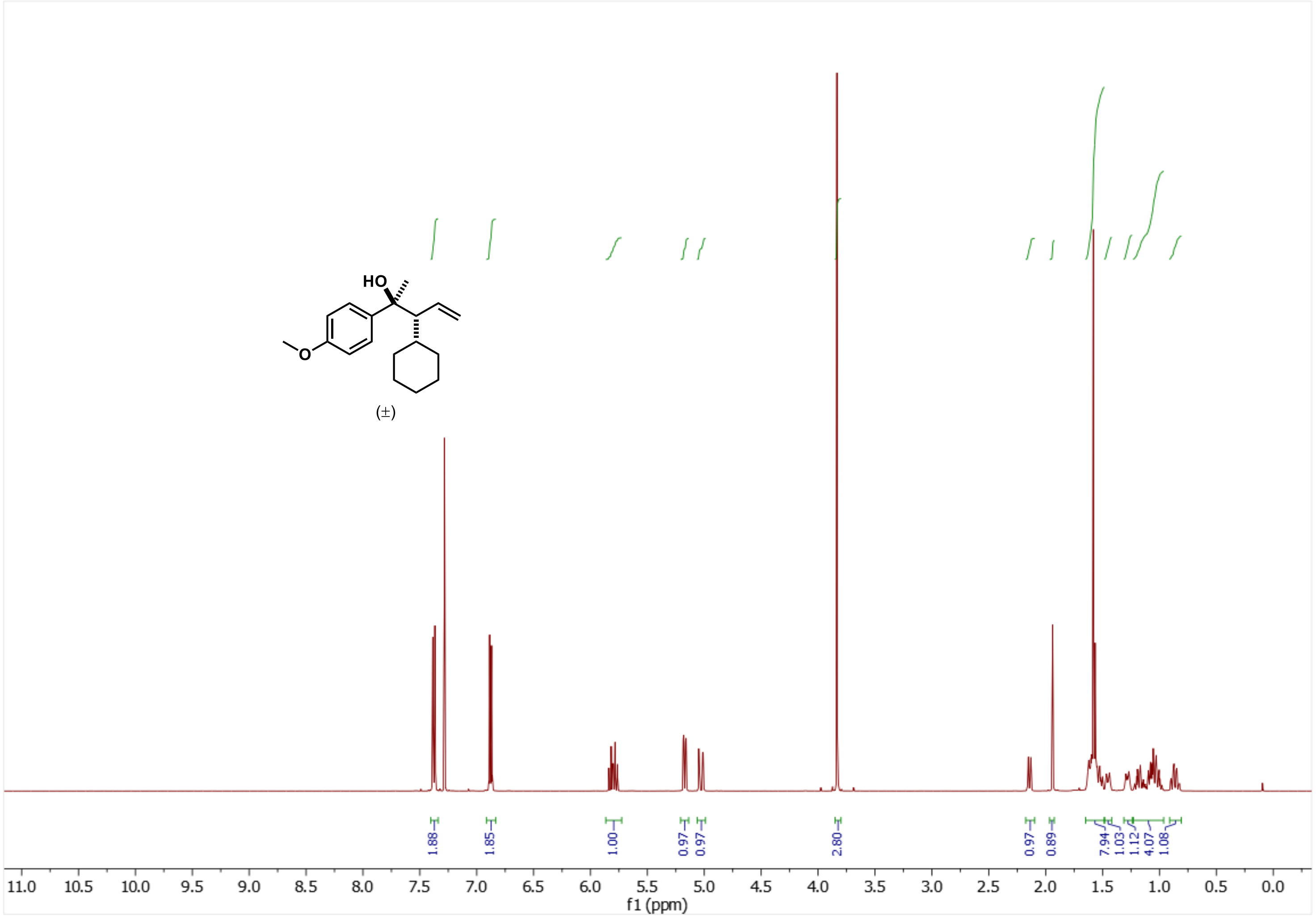

^{1}H NMR (500 MHz, $CDCl_3$) Spectra of (2*SR*,3*RS*)-3-cyclohexyl-2-(4-methoxyphenyl)pent-4-en-2-ol

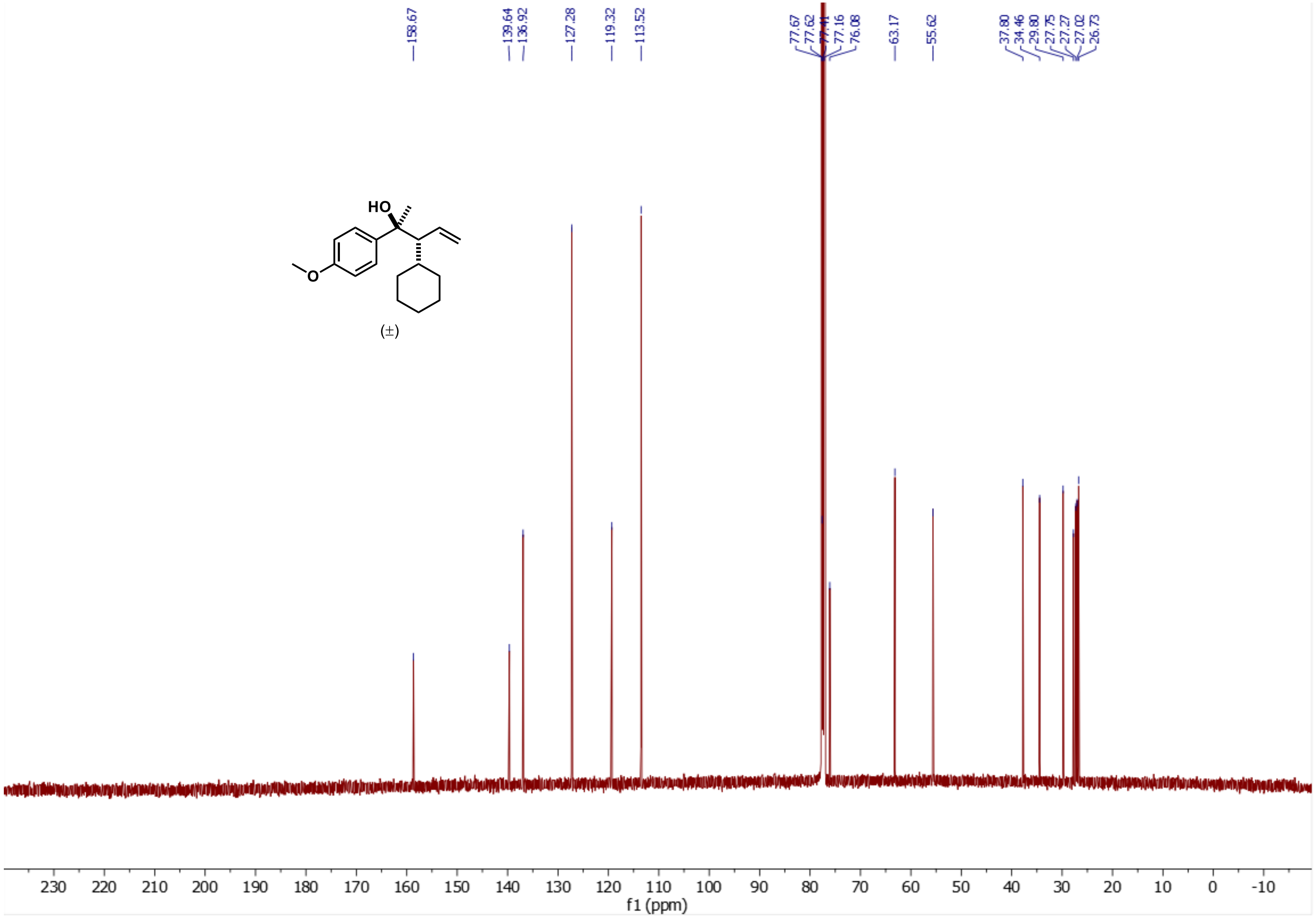

^{13}C NMR (126 MHz, $CDCl_3$) Spectra of (2*SR*,3*RS*)-3-cyclohexyl-2-(4-methoxyphenyl)pent-4-en-2-ol

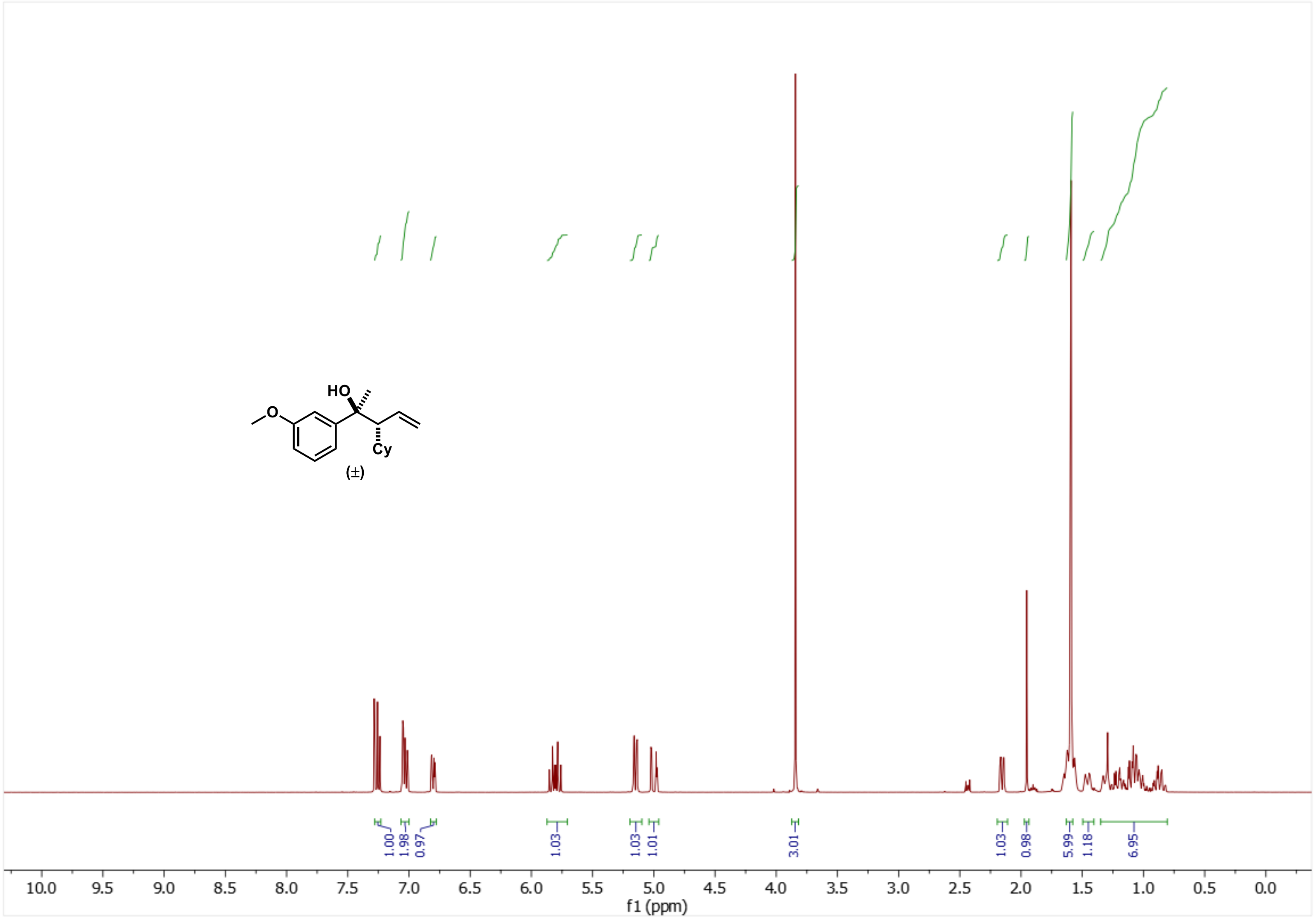

^{1}H NMR (500 MHz, $CDCl_3$) Spectra of (2*SR*,3*RS*)-3-cyclohexyl-2-(3-methoxyphenyl)pent-4-en-2-ol

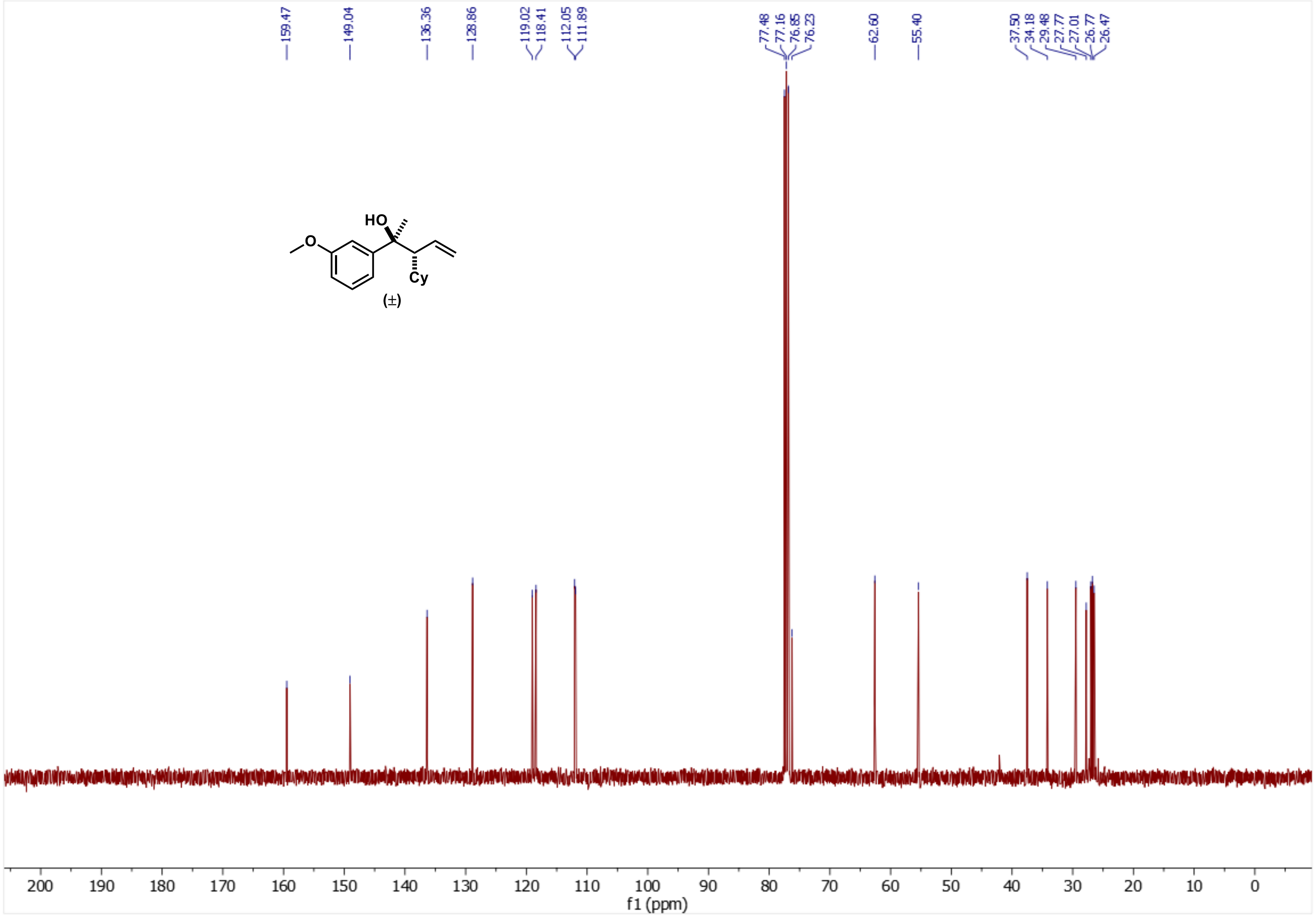

[13]**C NMR** (126 MHz, $CDCl_3$) Spectra of (2*SR*,3*RS*)-3-cyclohexyl-2-(3-methoxyphenyl)pent-4-en-2-ol

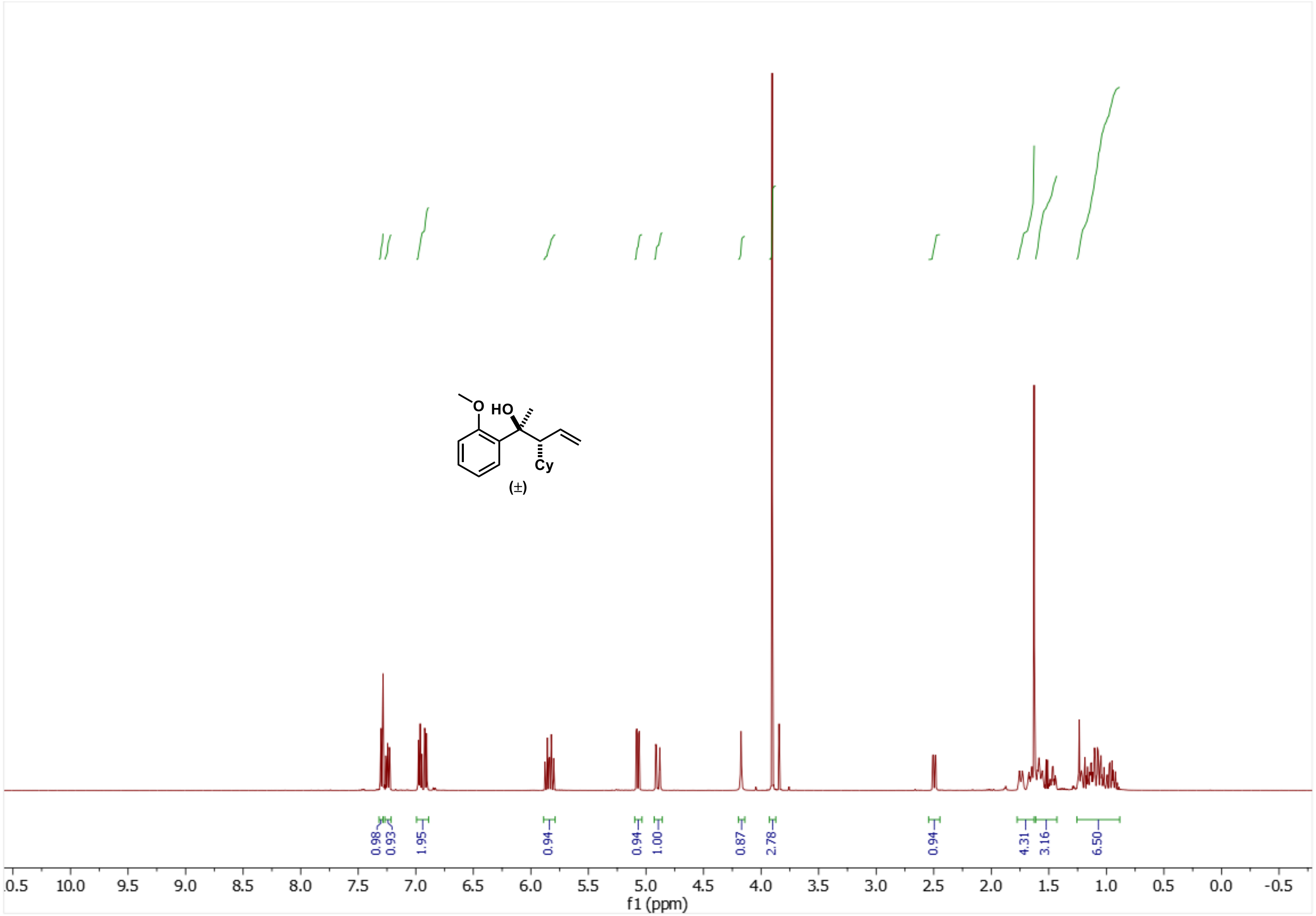

^{1}H NMR (500 MHz, $CDCl_3$) Spectra of (2*SR*,3*RS*)-3-cyclohexyl-2-(2-methoxyphenyl)pent-4-en-2-ol

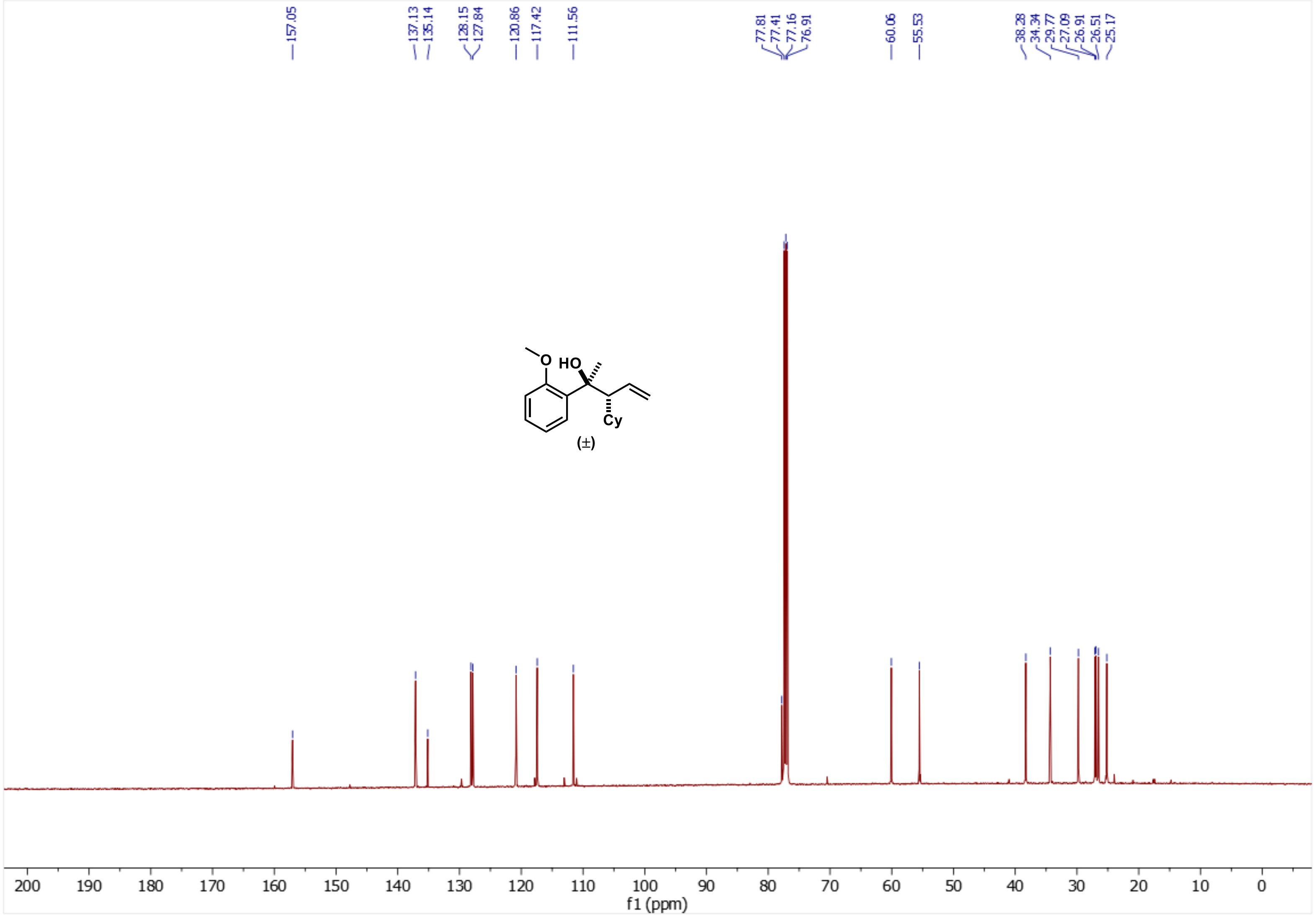

^{13}C NMR (126 MHz, $CDCl_3$) Spectra of (2*SR*,3*RS*)-3-cyclohexyl-2-(2-methoxyphenyl)pent-4-en-2-ol

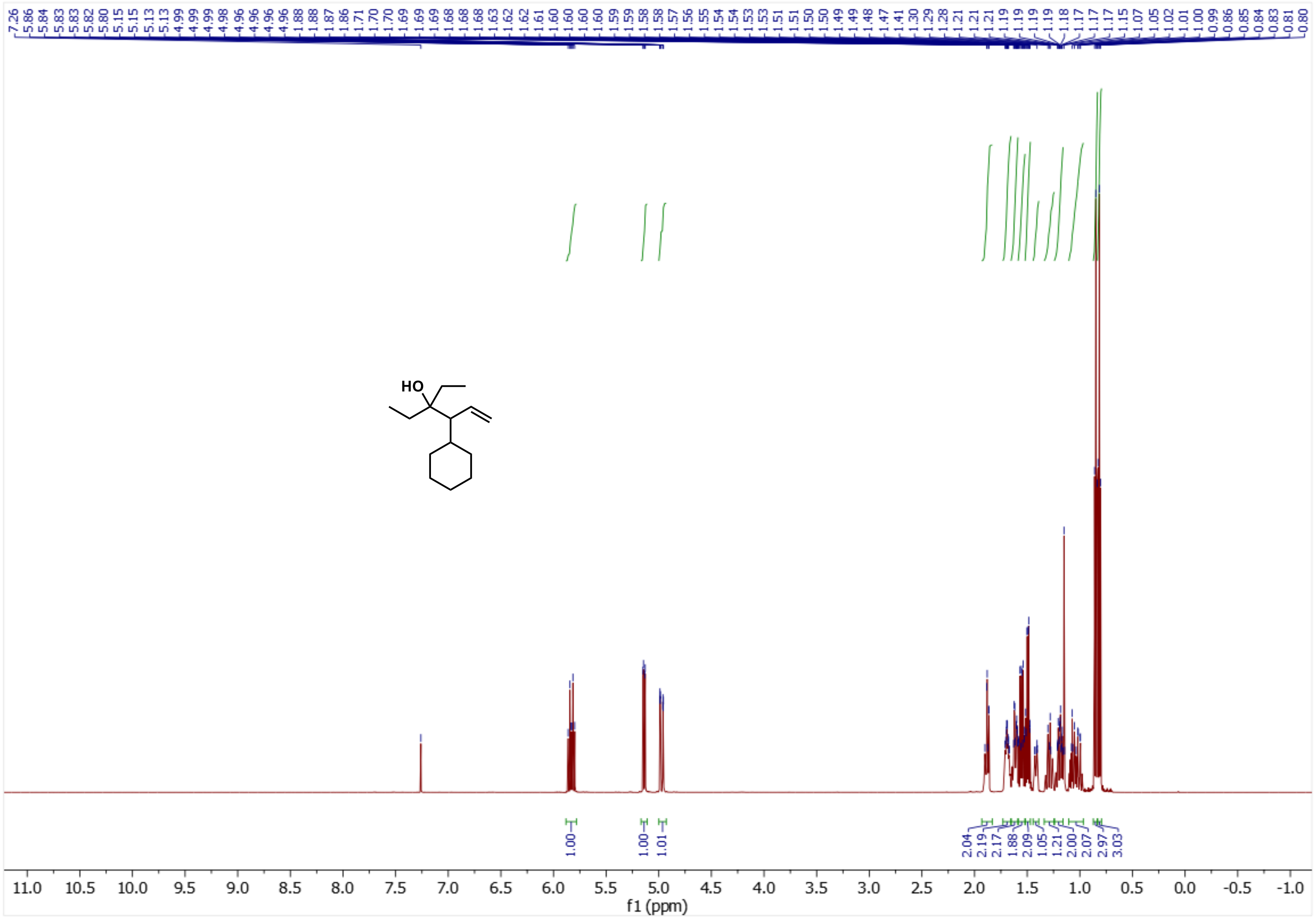

^{1}H NMR (500 MHz, $CDCl_3$) Spectra of 4-cyclohexyl-3-ethylhex-5-en-3-ol

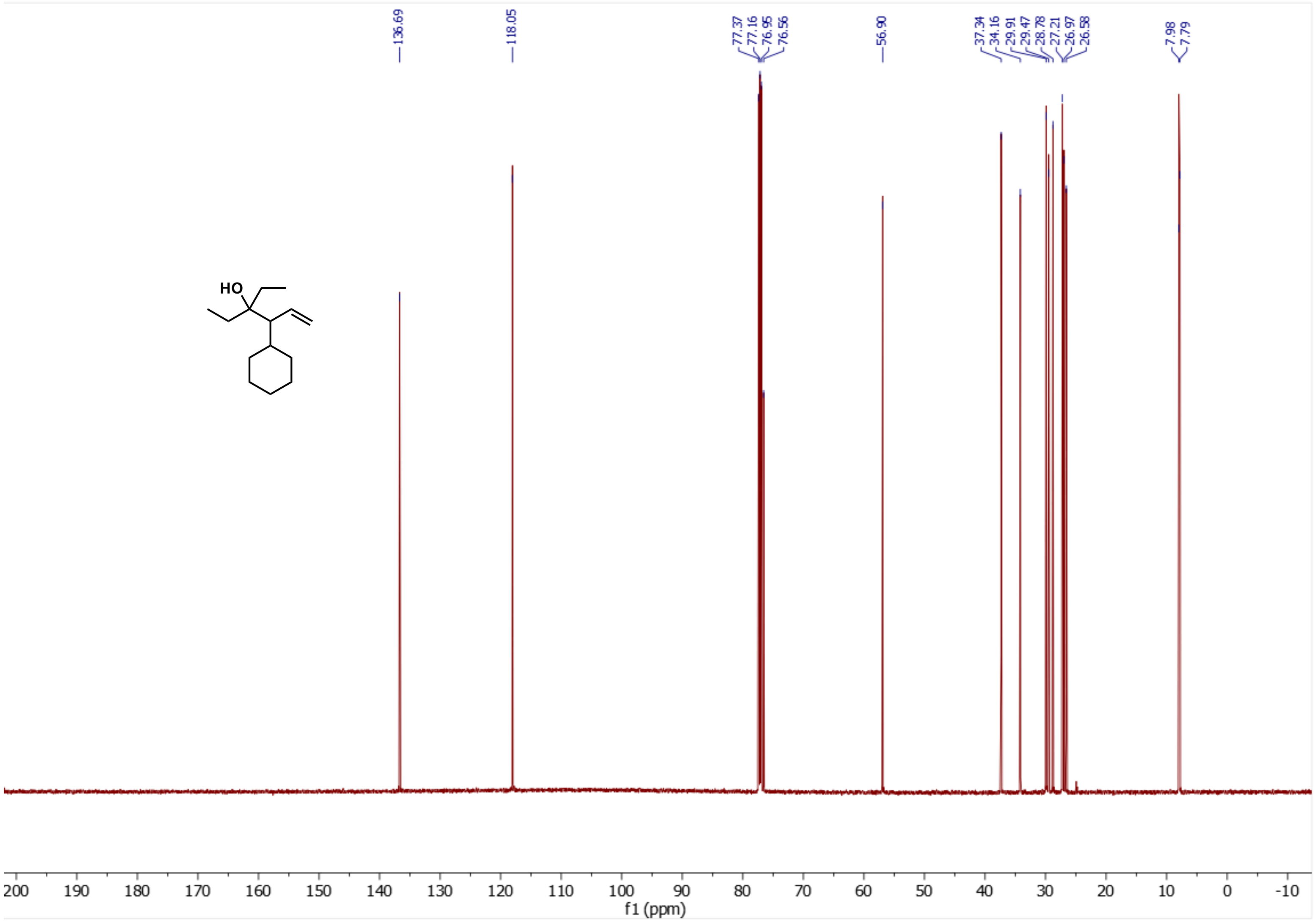

^{13}C NMR (126 MHz, $CDCl_3$) Spectra of 4-cyclohexyl-3-ethylhex-5-en-3-ol

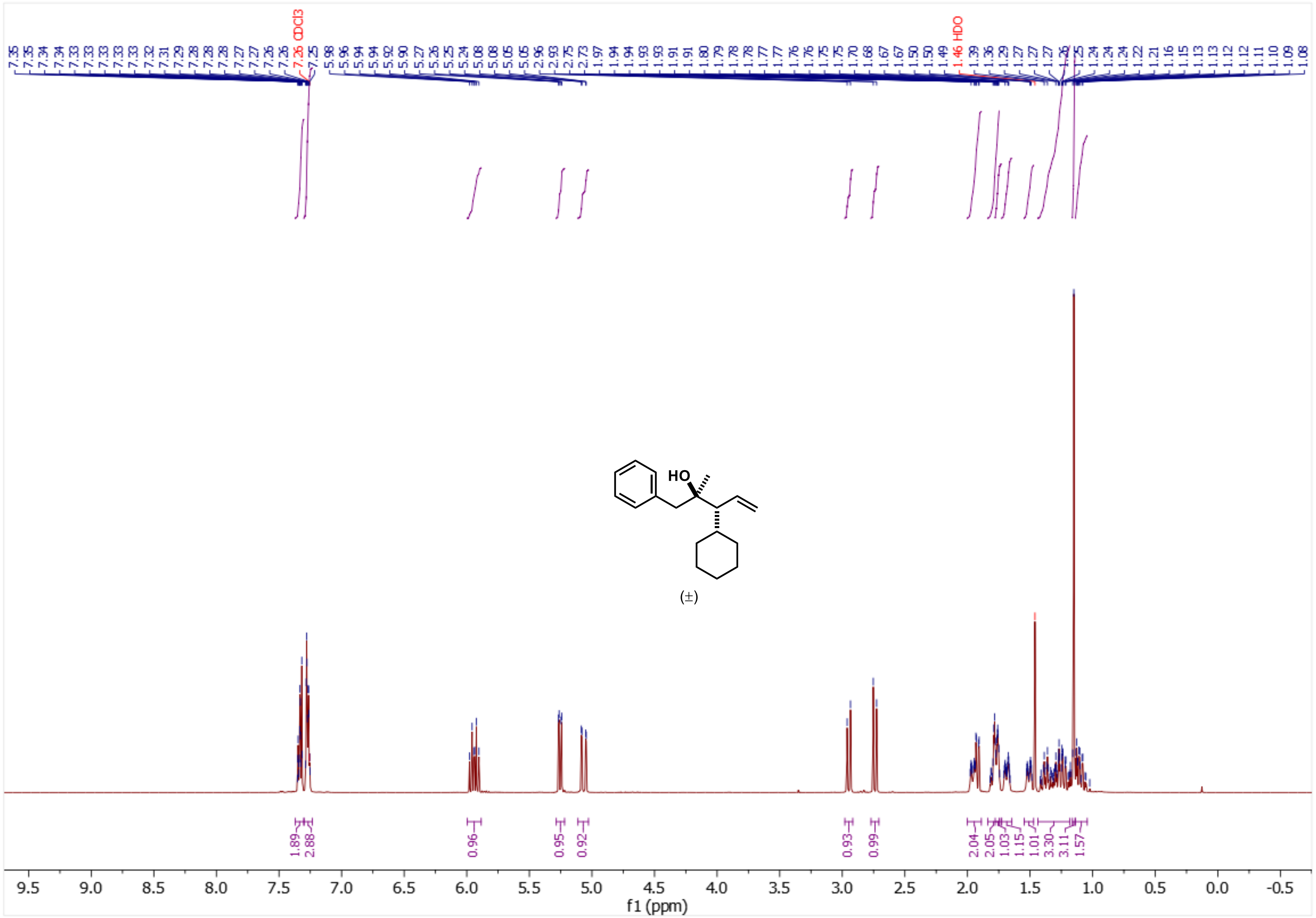

^{1}H NMR (500 MHz, $CDCl_3$) Spectra of (2*SR*,3*RS*)-3-cyclohexyl-2-benzylpent-4-en-2-ol

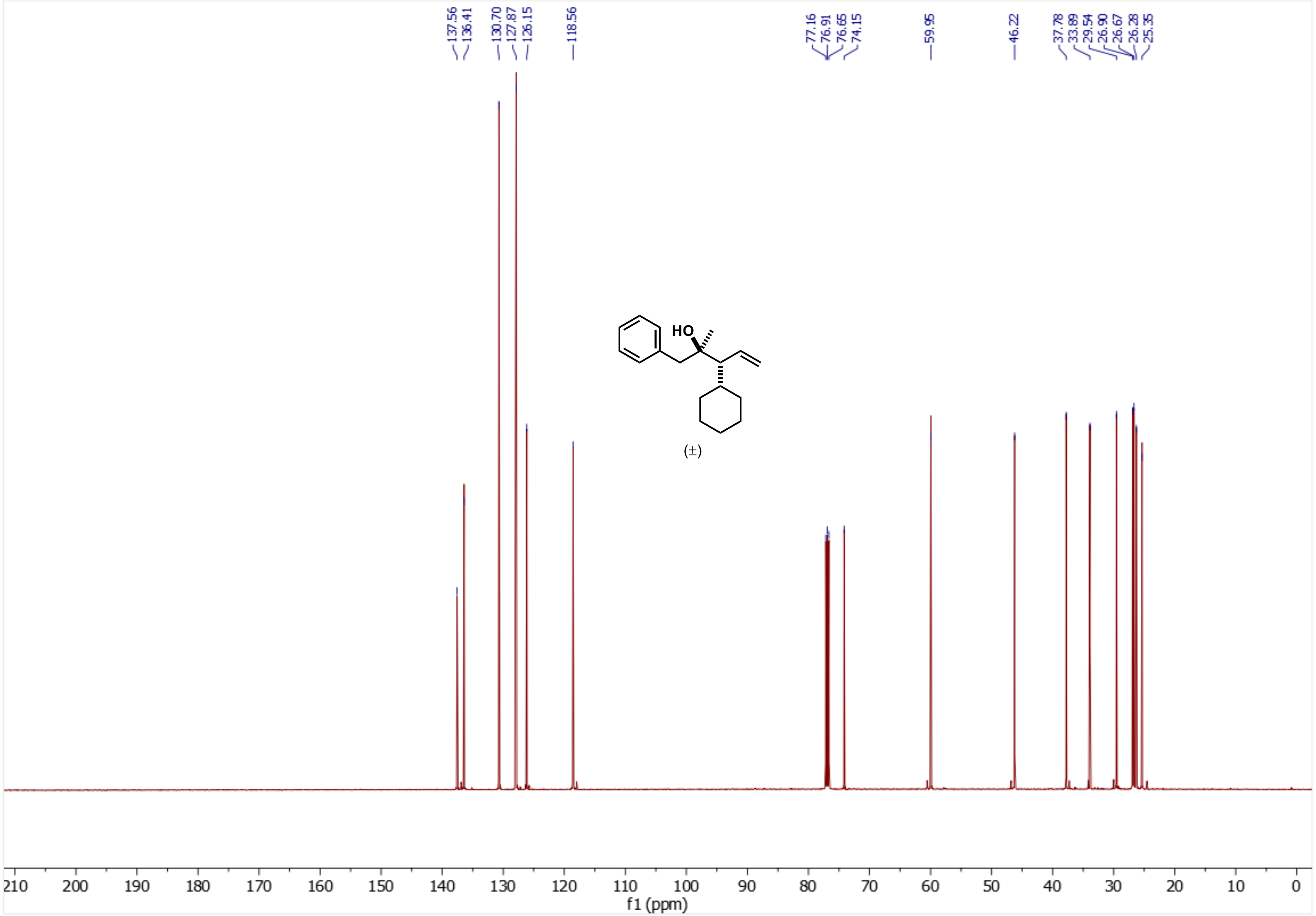

^{13}C NMR (126 MHz, $CDCl_3$) Spectra of (2*SR*,3*RS*)-3-cyclohexyl-2-benzylpent-4-en-2-ol

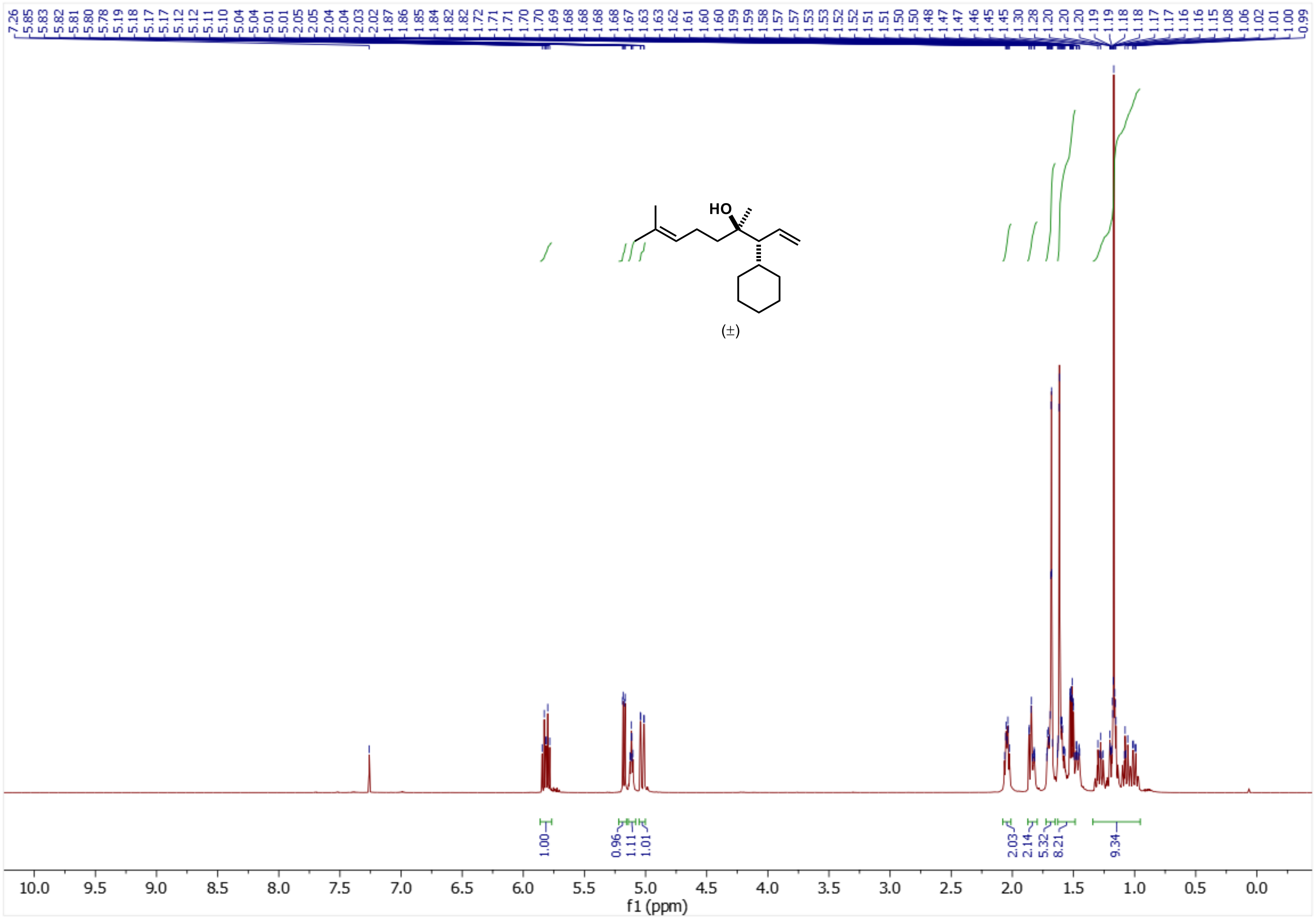

^{1}H NMR (500 MHz, $CDCl_3$) Spectra of (3*SR*, 4*RS*)-3-cyclohexyl-4-methylnon-1,7-dien-4-ol

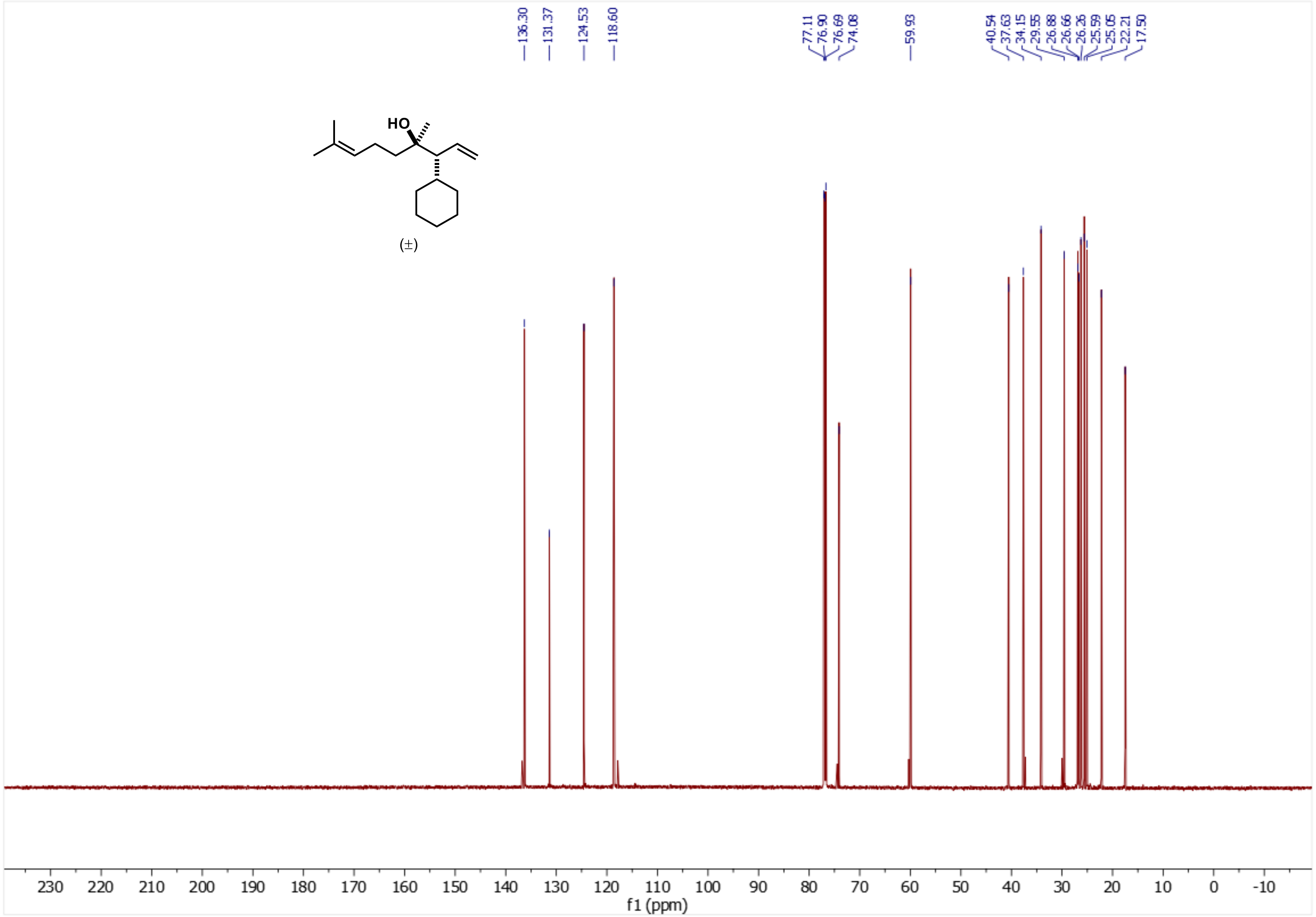

^{13}C NMR (126 MHz, $CDCl_3$) Spectra of (3*SR*,4*RS*)-3-cyclohexyl-4-methylnon-1,7-dien-4-ol

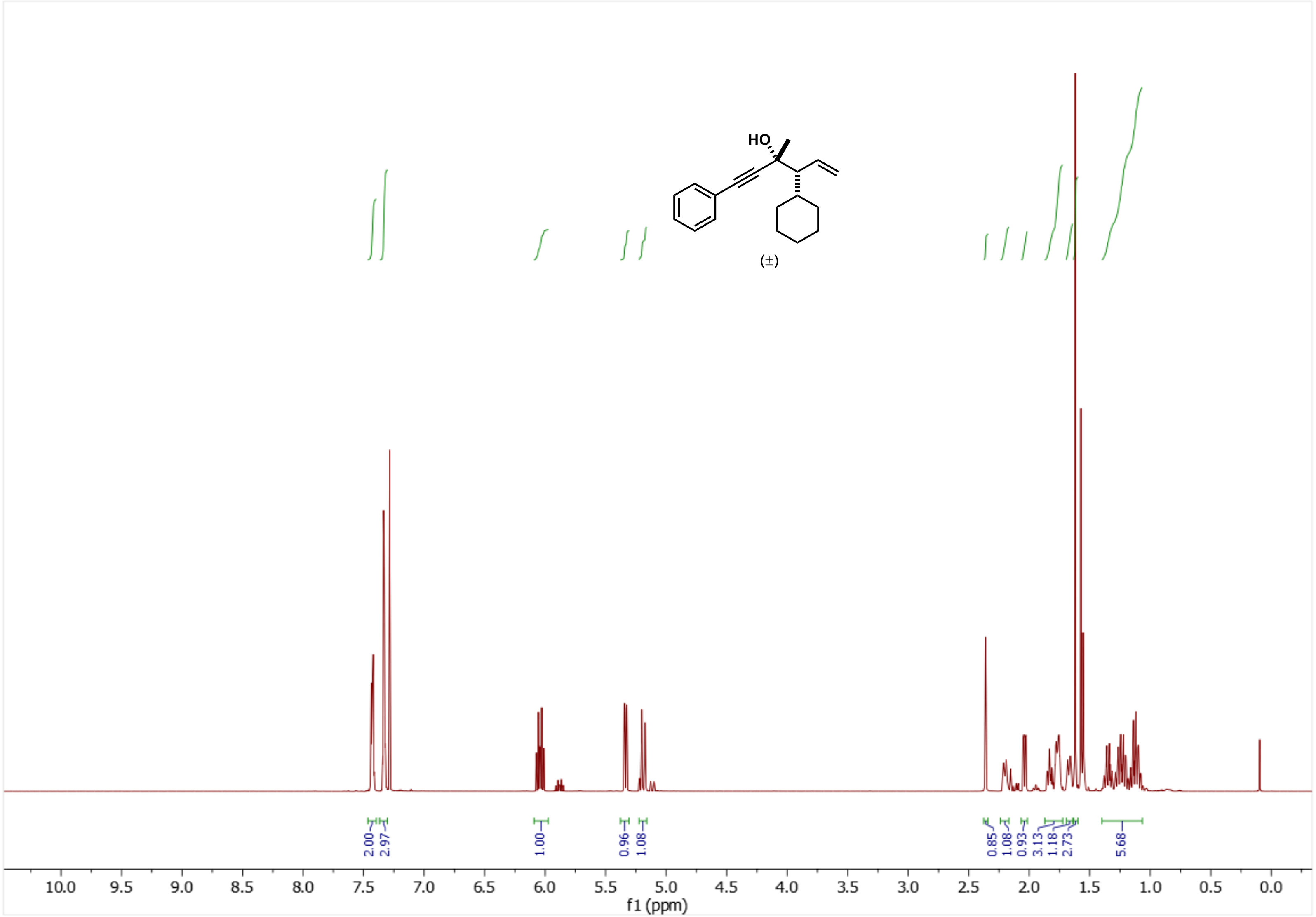

^{1}H NMR (500 MHz, $CDCl_3$) Spectra of (3*RS*,4*RS*)-4-cyclohexyl-3-methyl-1-phenylhex-1-yne-5-en-3-ol

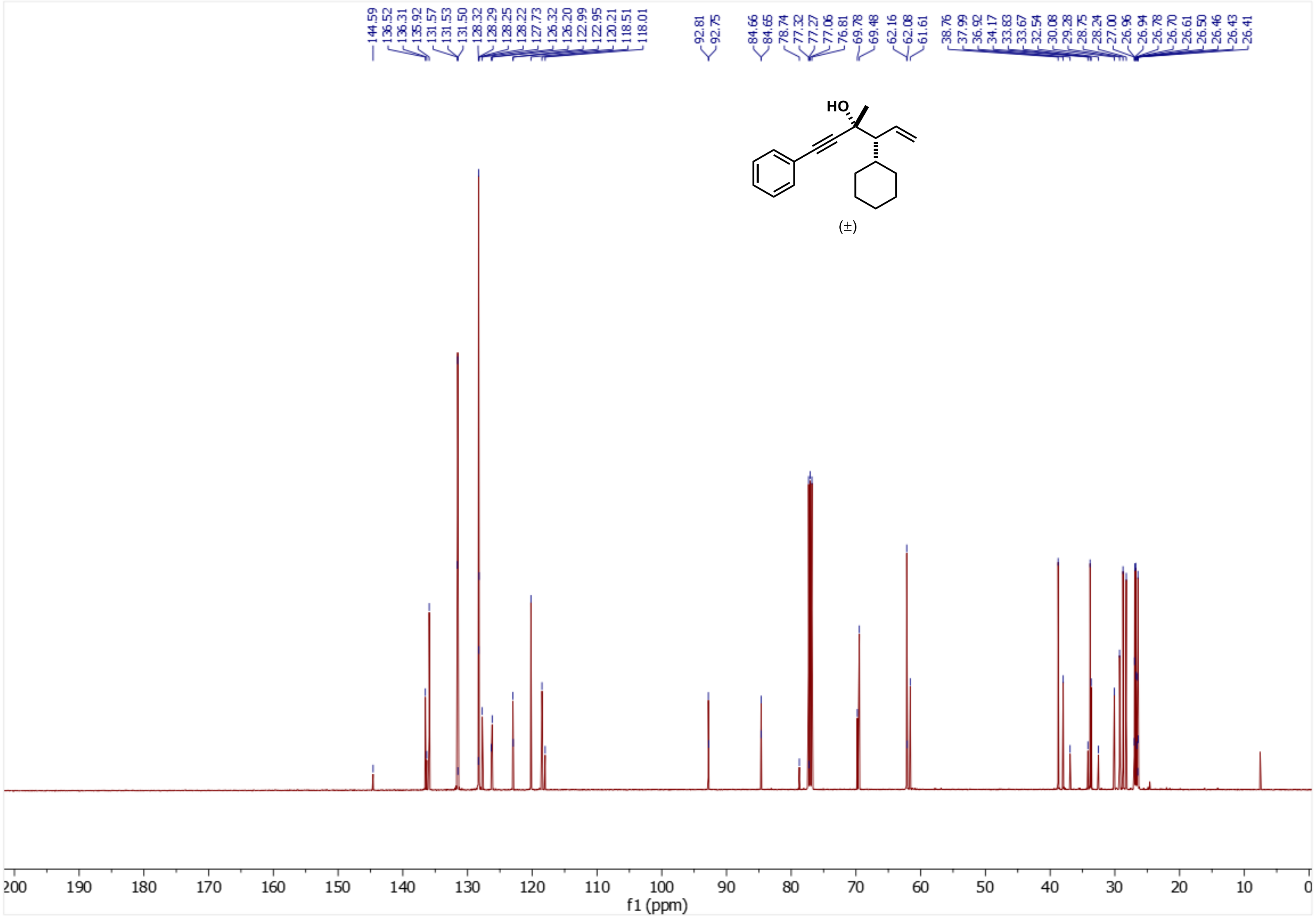

^{13}C NMR (126 MHz, $CDCl_3$) Spectra of (3*RS*,4*RS*)- 4-cyclohexyl-3-methyl-1-phenylhex-1-yne-5-en-3-ol

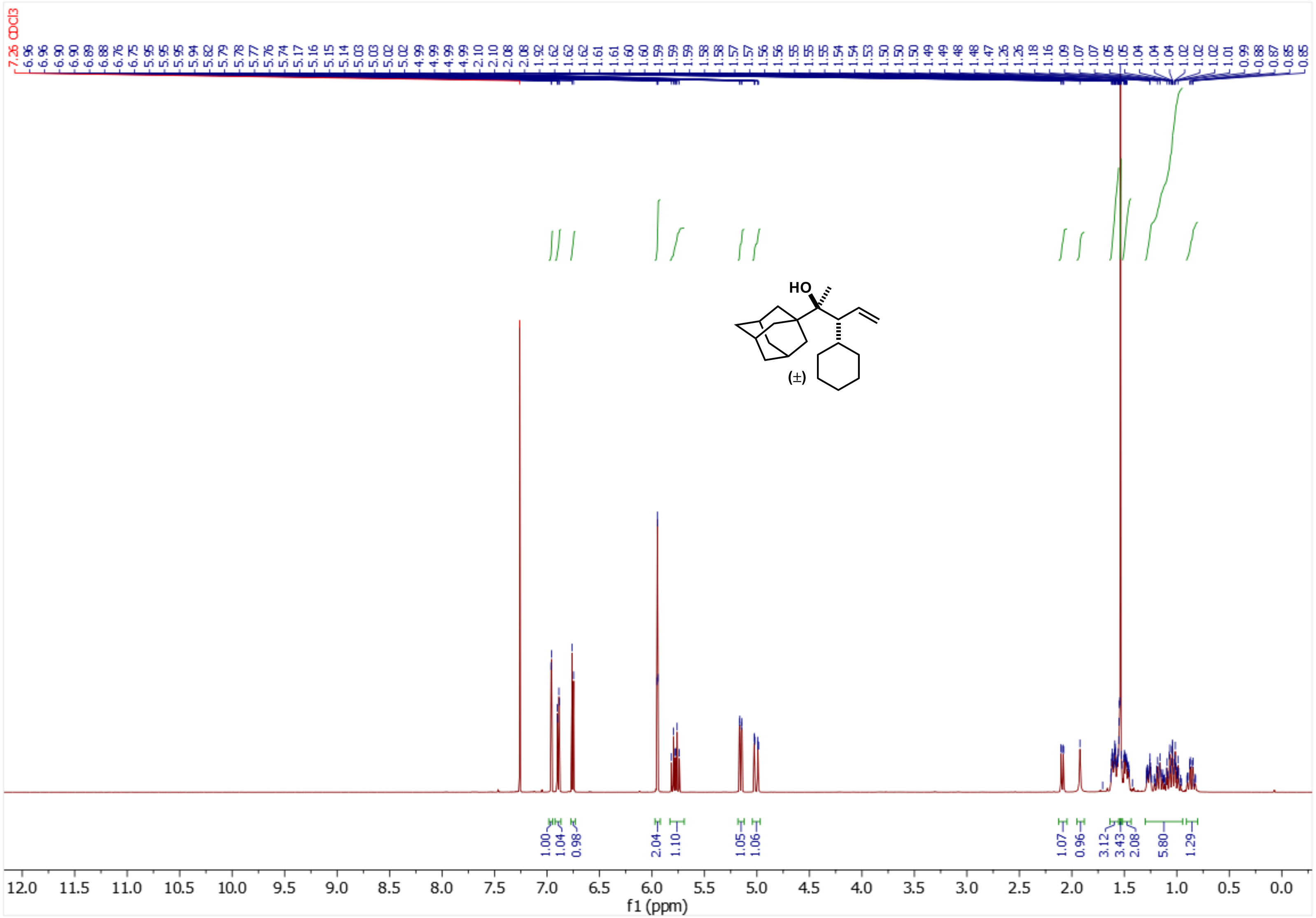

^{1}H NMR (500 MHz, $CDCl_3$) Spectra of (2*SR*,3*RS*)-3-cyclohexyl-2-adamantylpent-4-en-2-ol

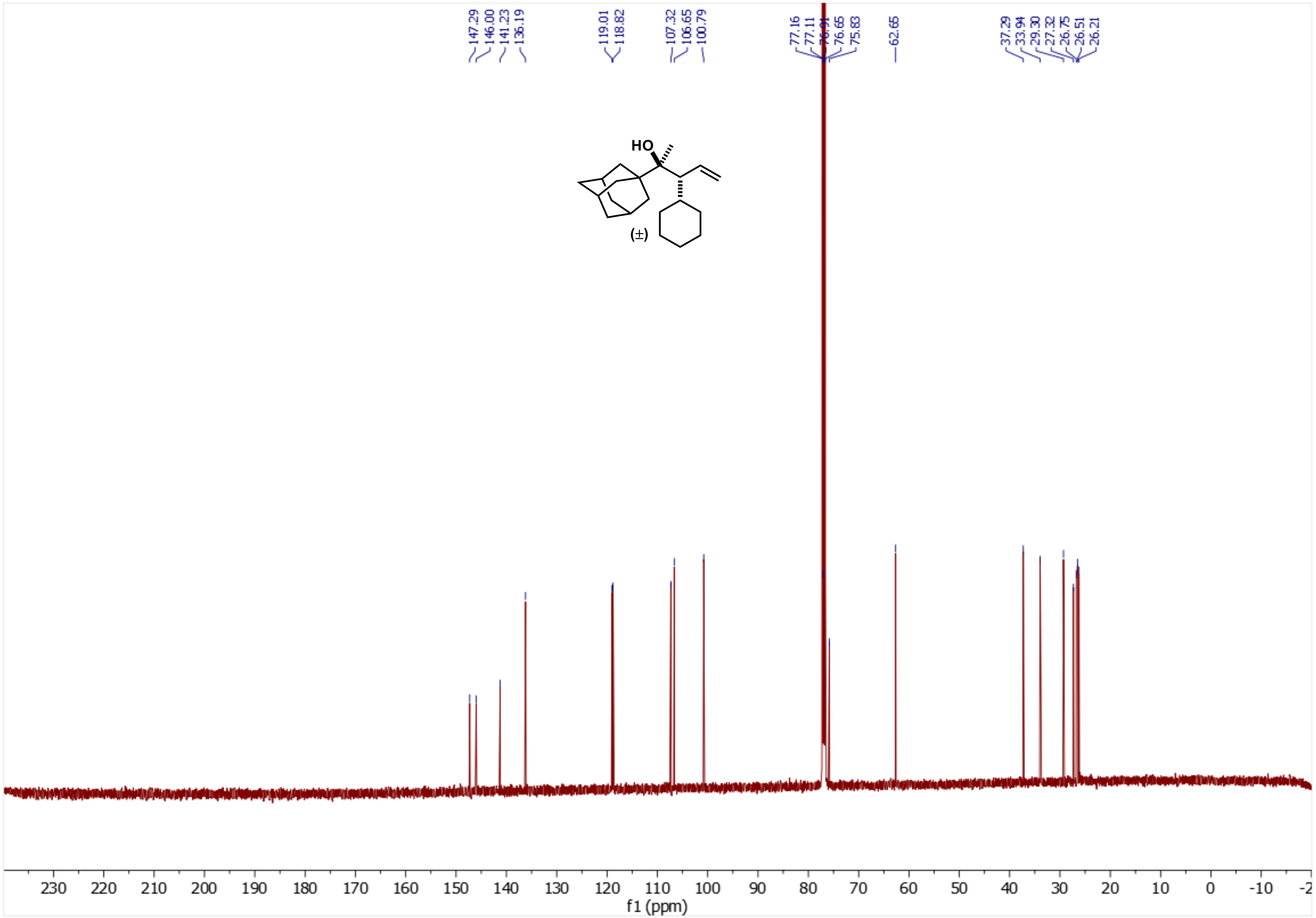

^{13}C NMR (126 MHz, $CDCl_3$) Spectra of (2*SR*,3*RS*)-3-cyclohexyl-2-adamantylpent-4-en-2-ol

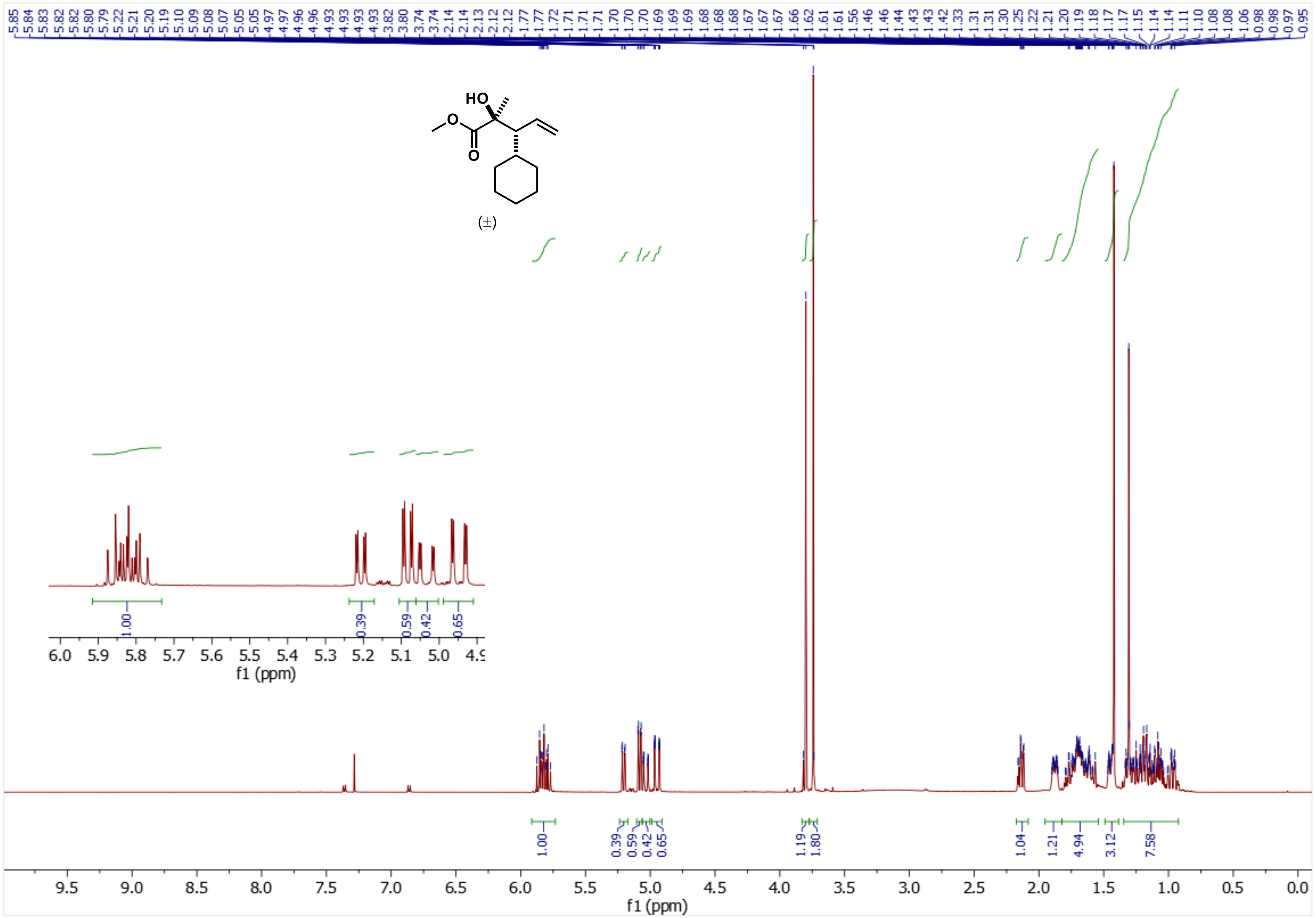

[1]**H NMR** (500 MHz, $CDCl_3$) Spectra of (2*SR*,3*RS*)-methyl 3-cyclohexyl-2-hydroxy-2-methylpent-4-enoate

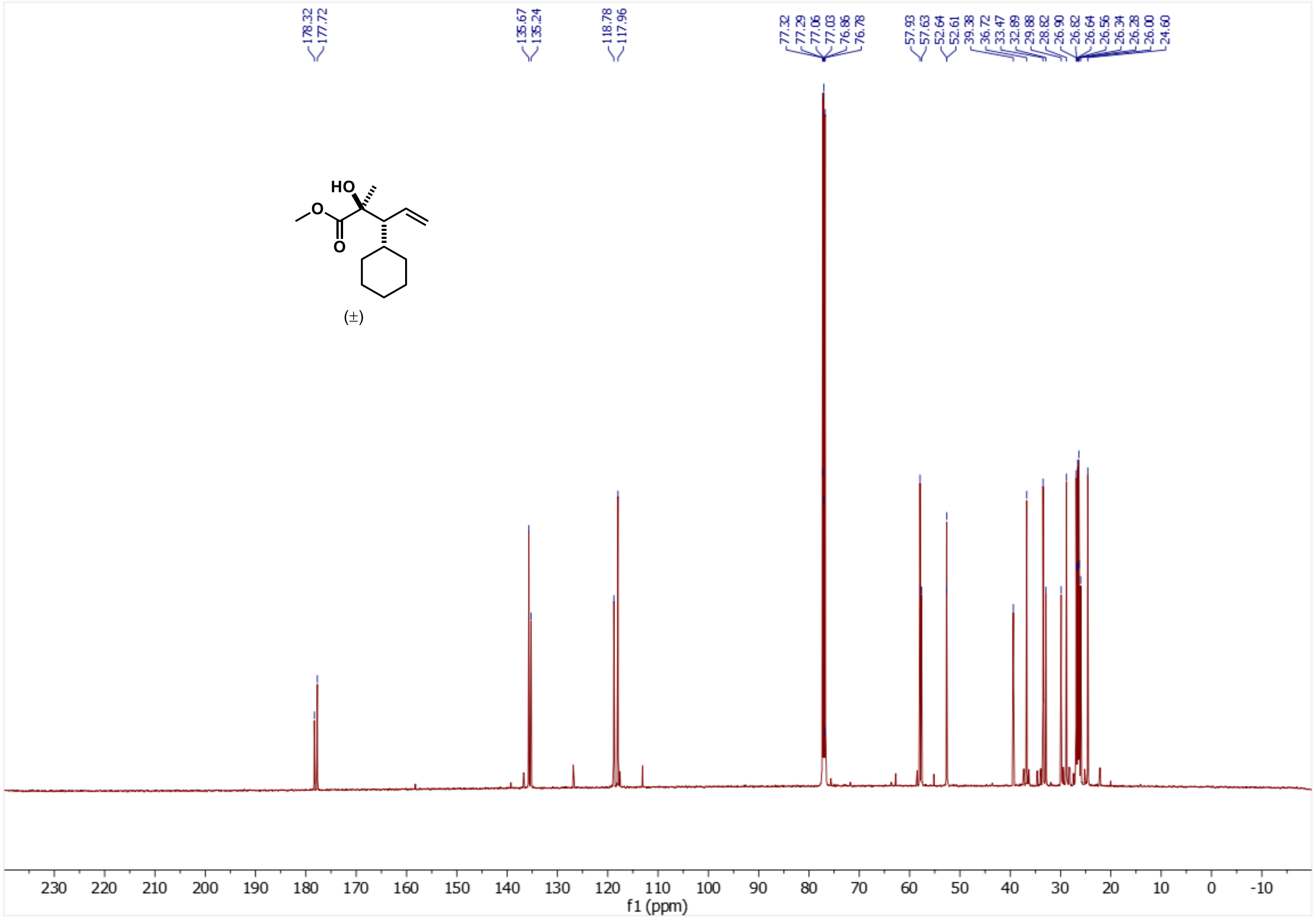

^{13}C NMR (126 MHz, $CDCl_3$) Spectra of (2*SR*,3*RS*)-methyl 3-cyclohexyl-2-hydroxy-2-methylpent-4-enoate

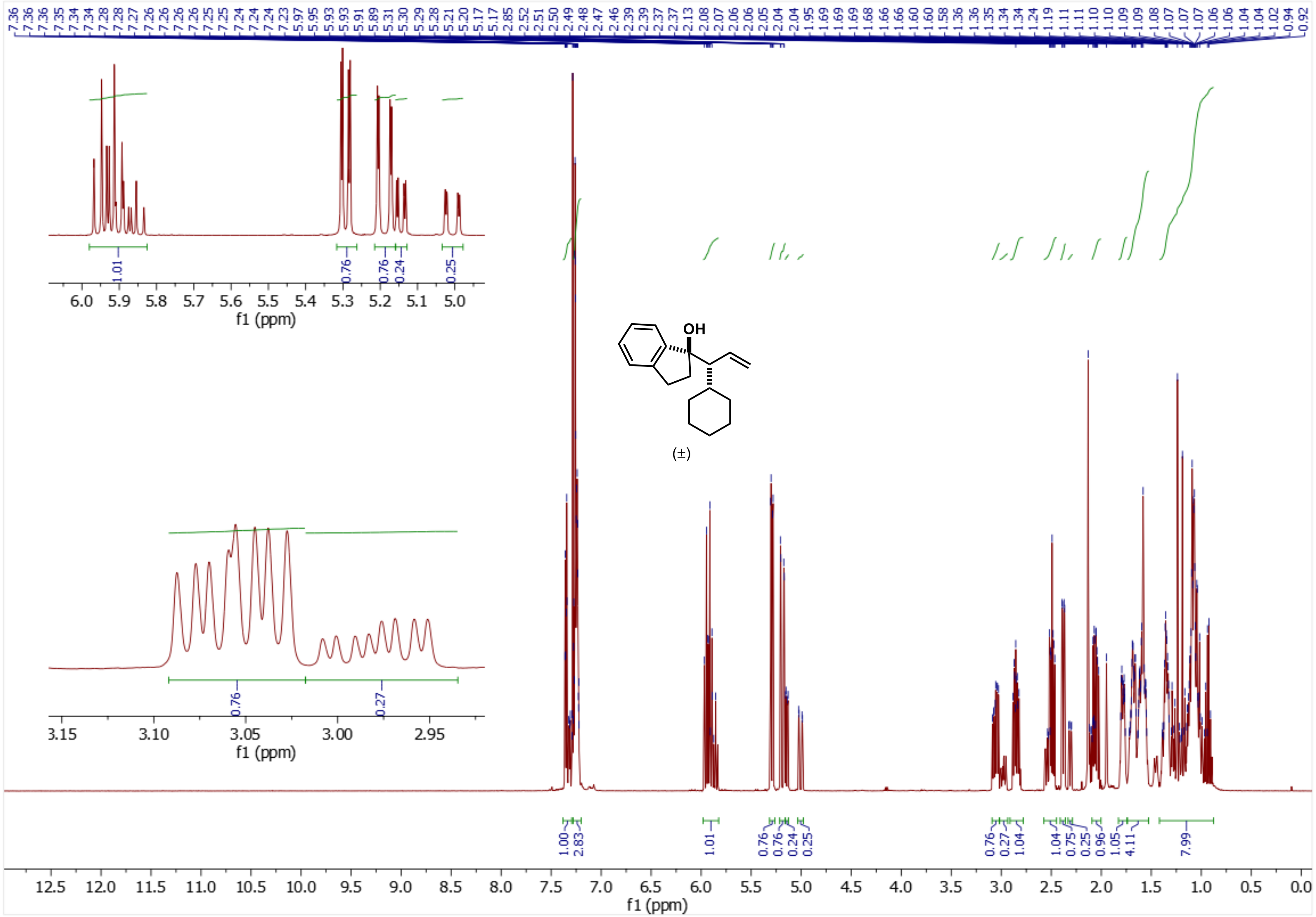

^{1}H NMR (500 MHz, $CDCl_3$) Spectra of (2*SR*,3*RS*)-1-(3-cyclohexylprop-1-ene)indanol

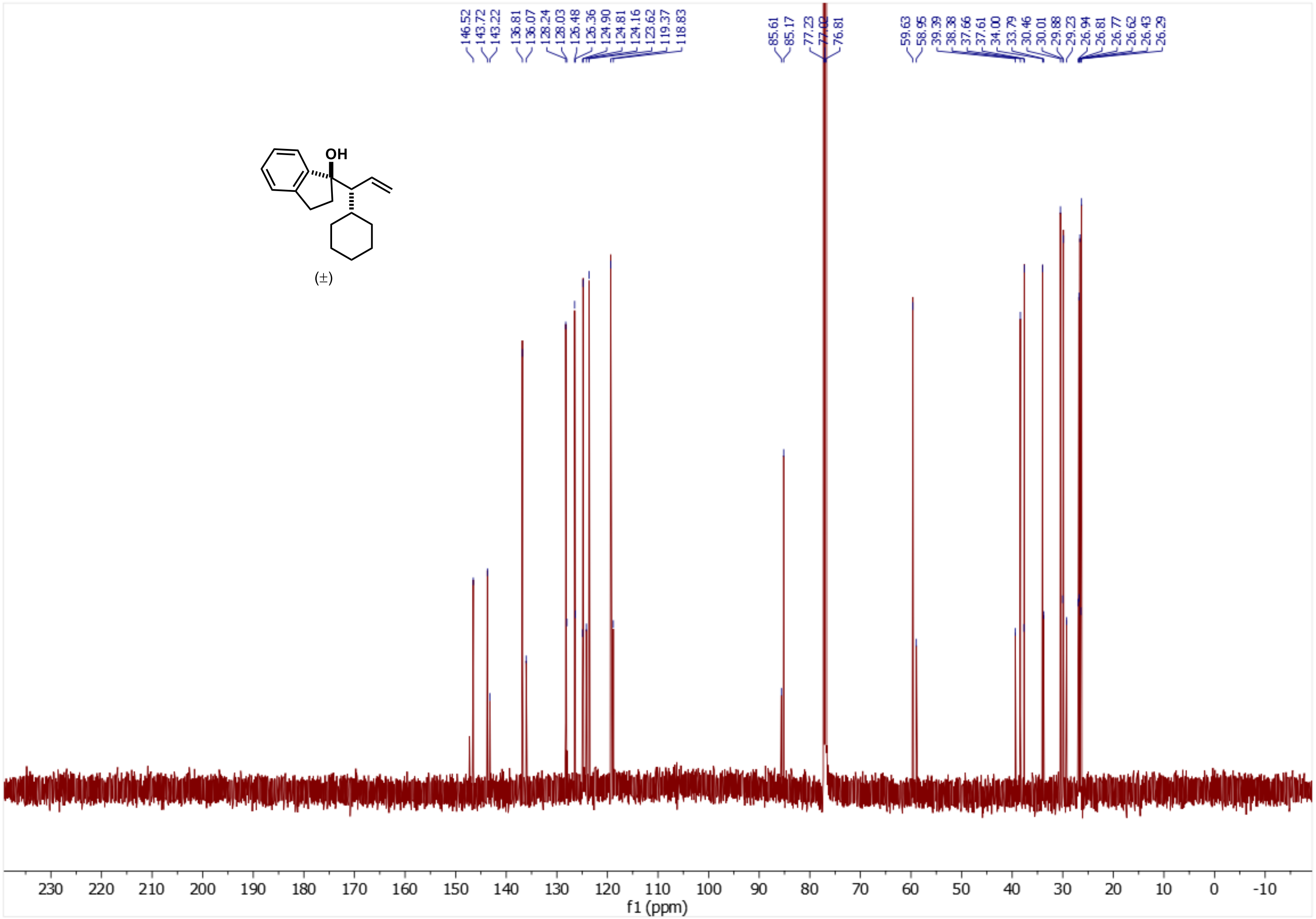

13C NMR (126 MHz, $CDCl_3$) Spectra of (2*SR*,3*RS*)-1-(3-cyclohexylprop-1-ene)indanol

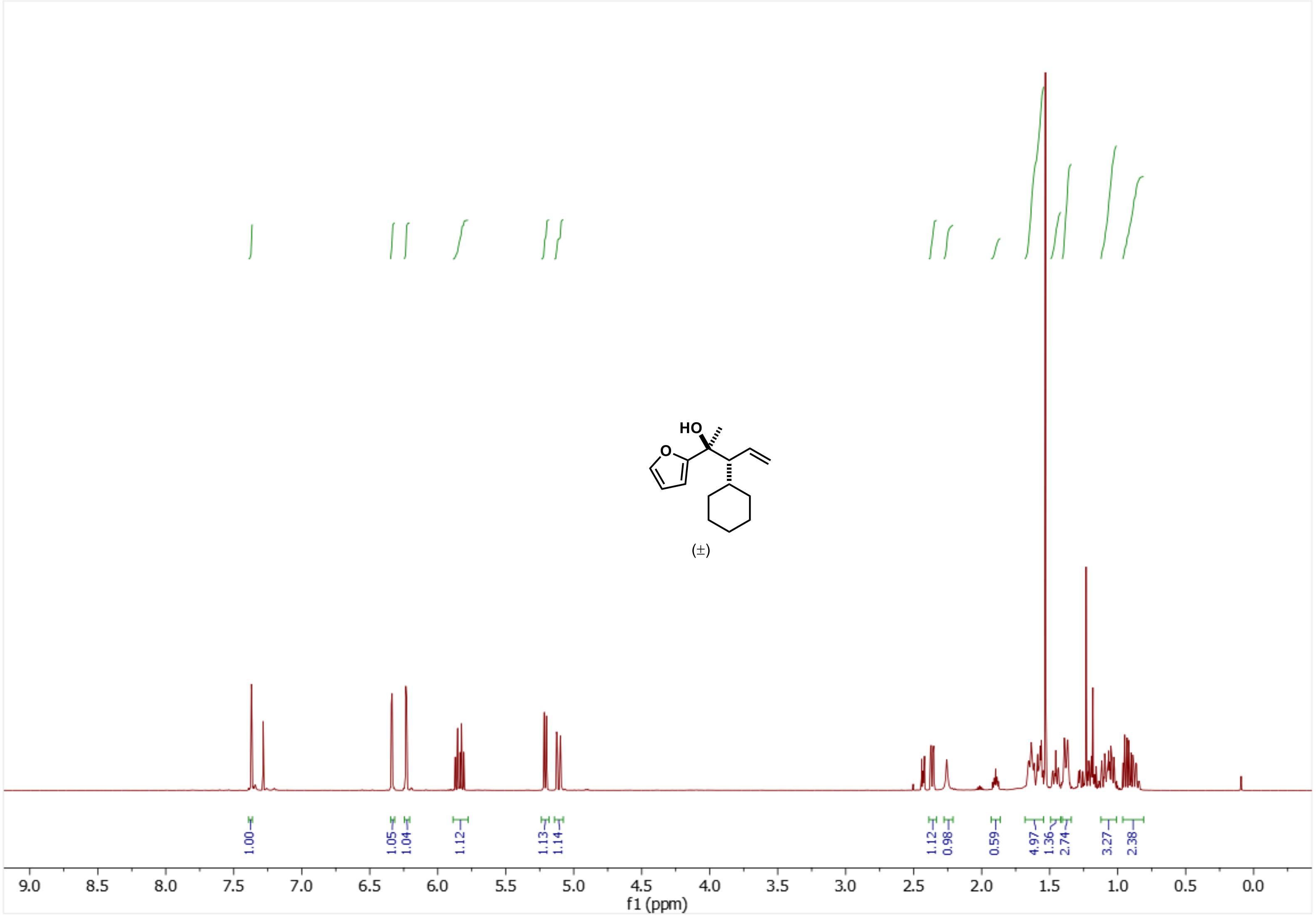

^{1}H NMR (500 MHz, $CDCl_3$) Spectra of (2*SR*,3*RS*)-2-(fur-2-yl)-3-cyclohexylpent-4-en-2-ol

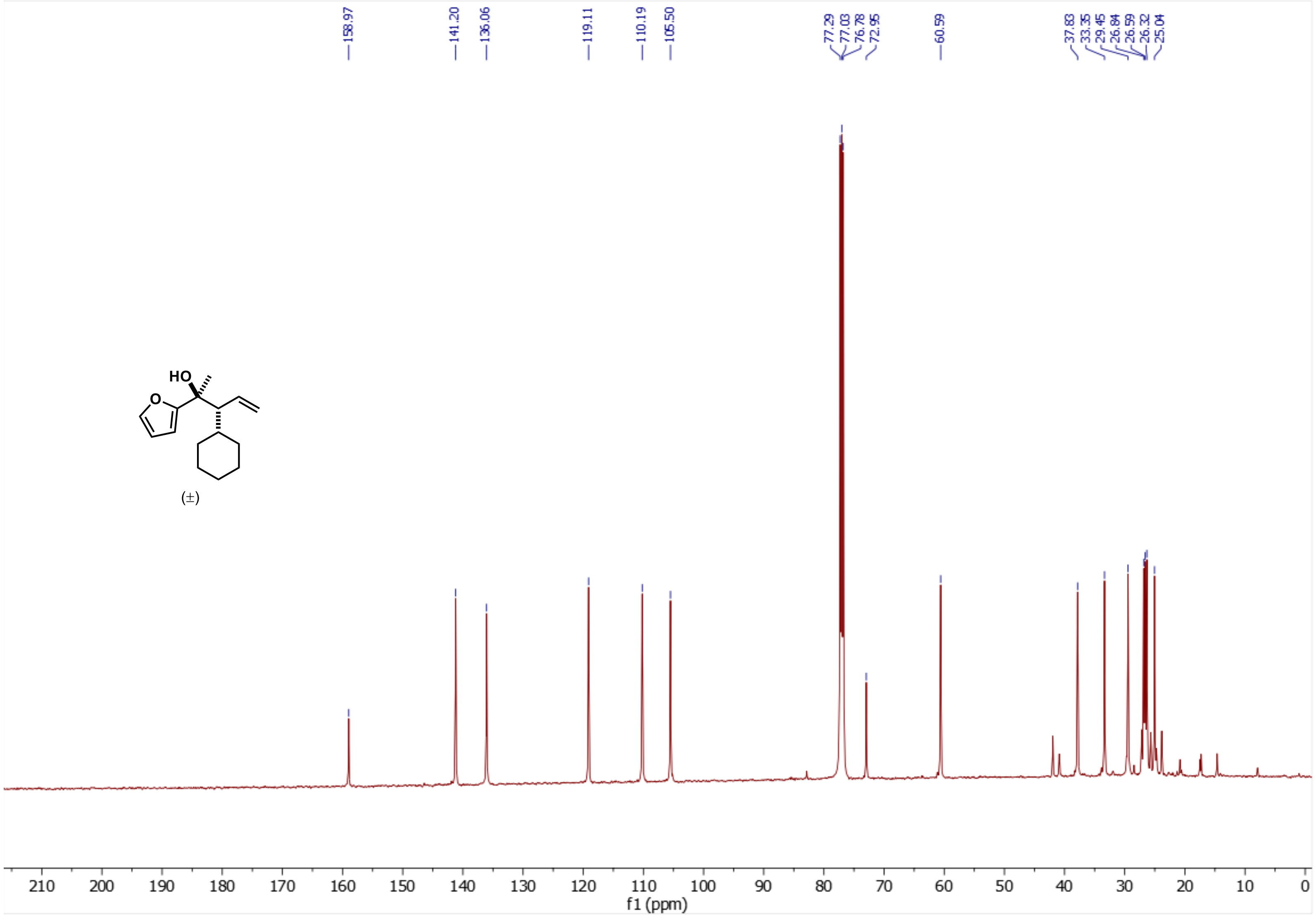

^{13}C NMR (126 MHz, $CDCl_3$) Spectra of (2*SR*,3*RS*)-2-(fur-2-yl)-3-cyclohexylpent-4-en-2-ol

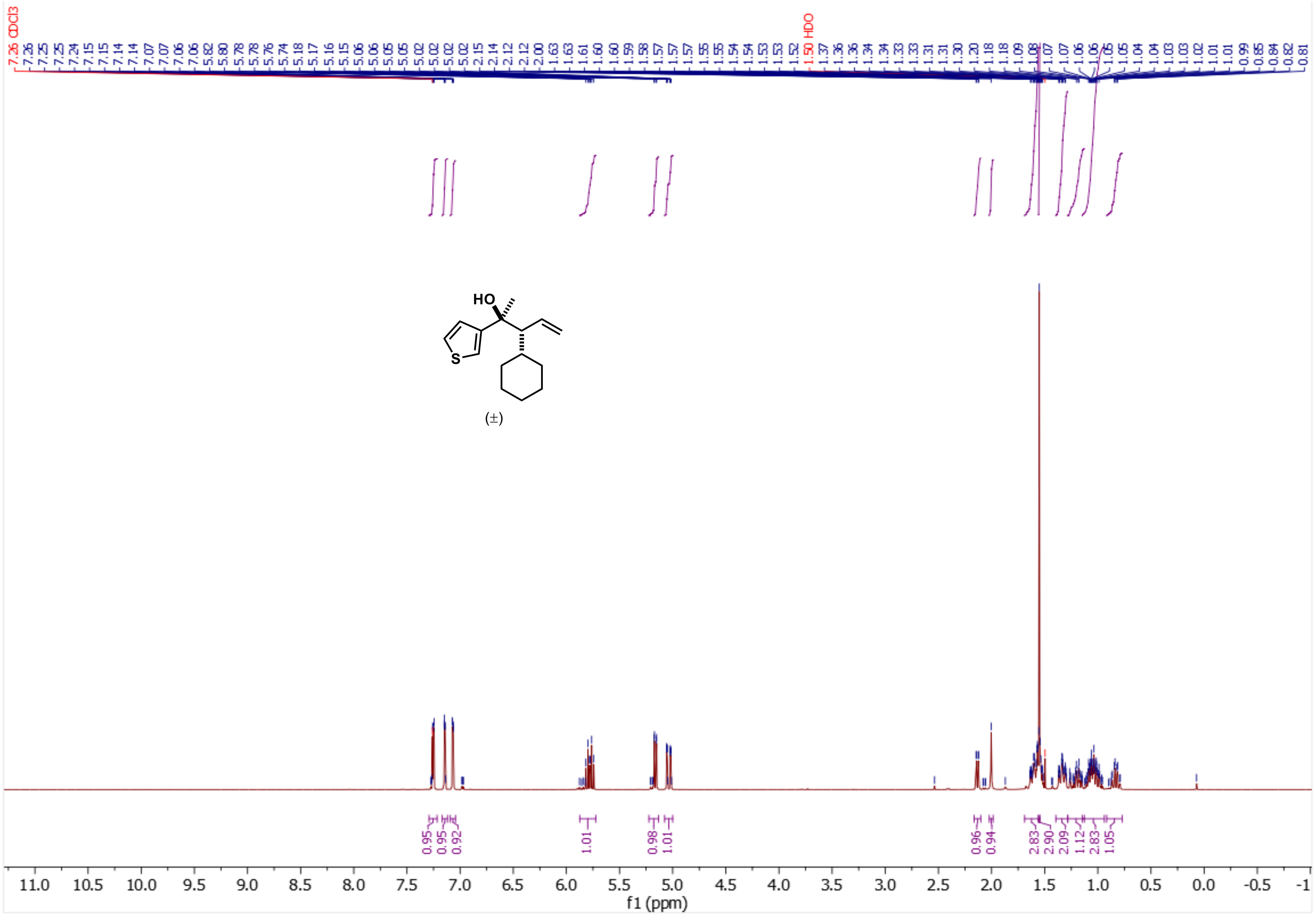

^{1}H NMR (500 MHz, $CDCl_3$) Spectra of (2*SR*,3*RS*)-2-(thiophen-2-yl)-3-cyclohexylpent-4-en-2-ol

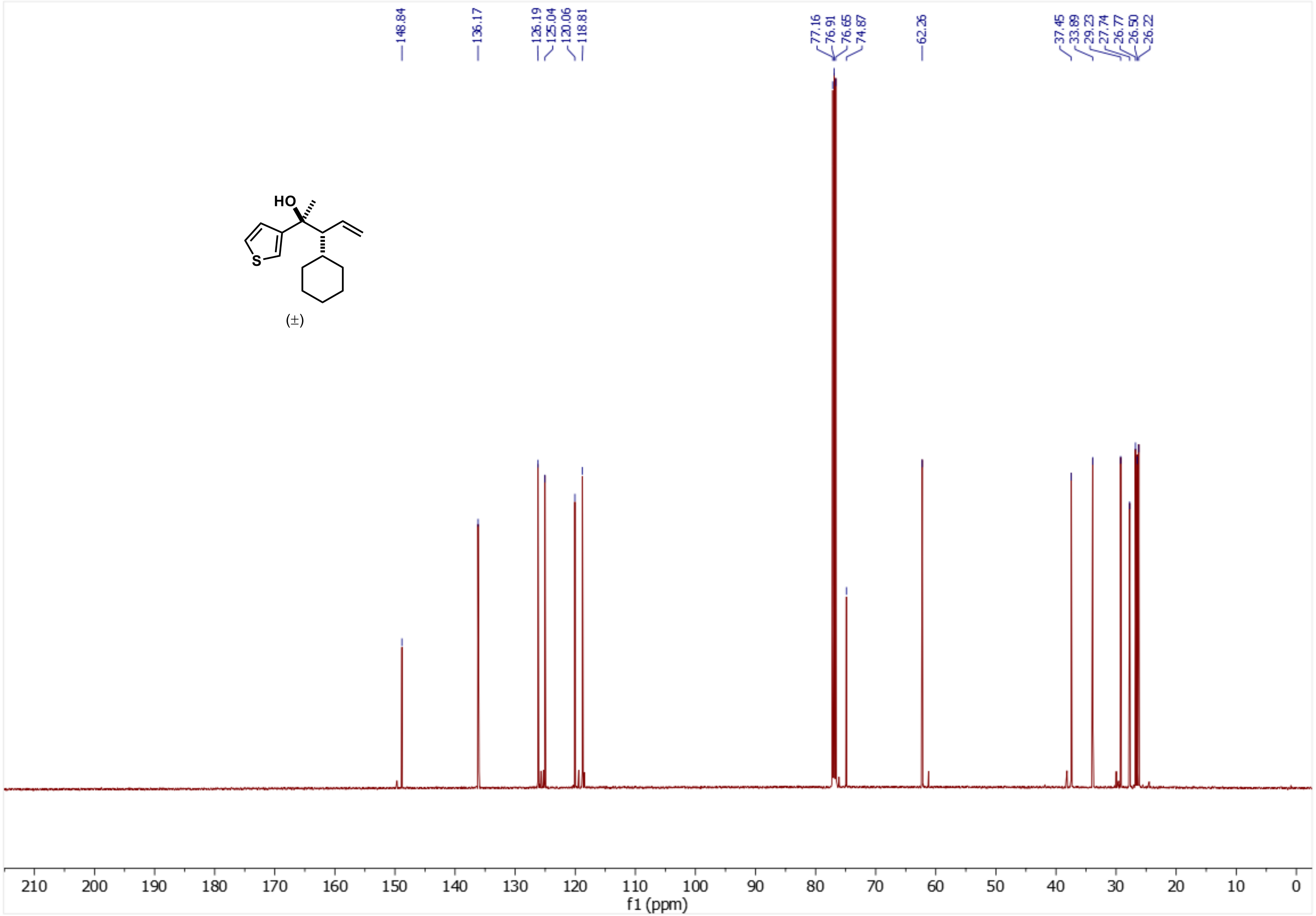

^{13}C NMR (126 MHz, $CDCl_3$) Spectra of (2*SR*,3*RS*)-2-(thiophen-2-yl)-3-cyclohexylpent-4-en-2-ol

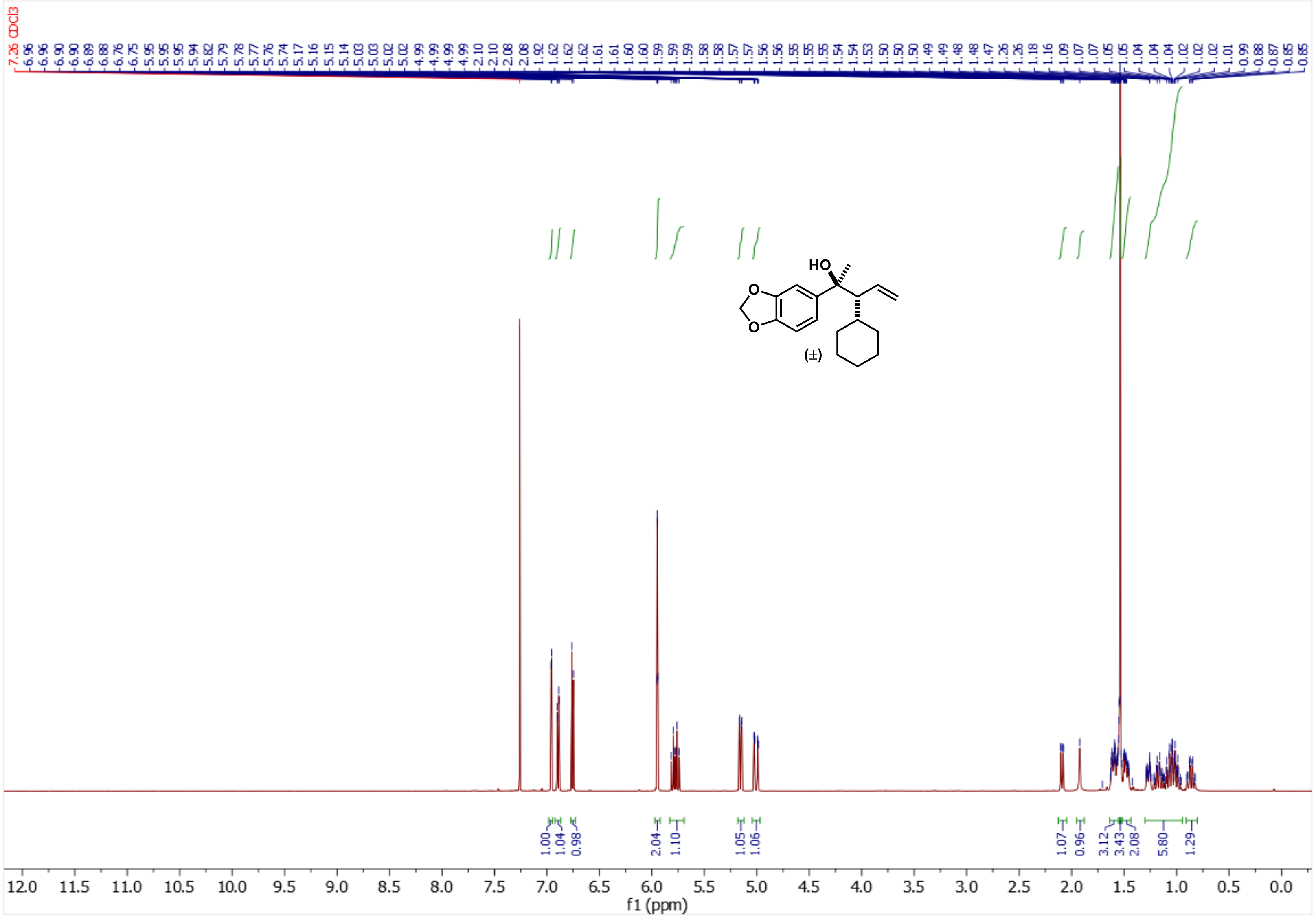

^{1}H NMR (500 MHz, $CDCl_3$) Spectra of (2*SR*,3*RS*)-3-cyclohexyl-2-(benzo[d][1,3]dioxol-6-yl))pent-4-en-2-ol

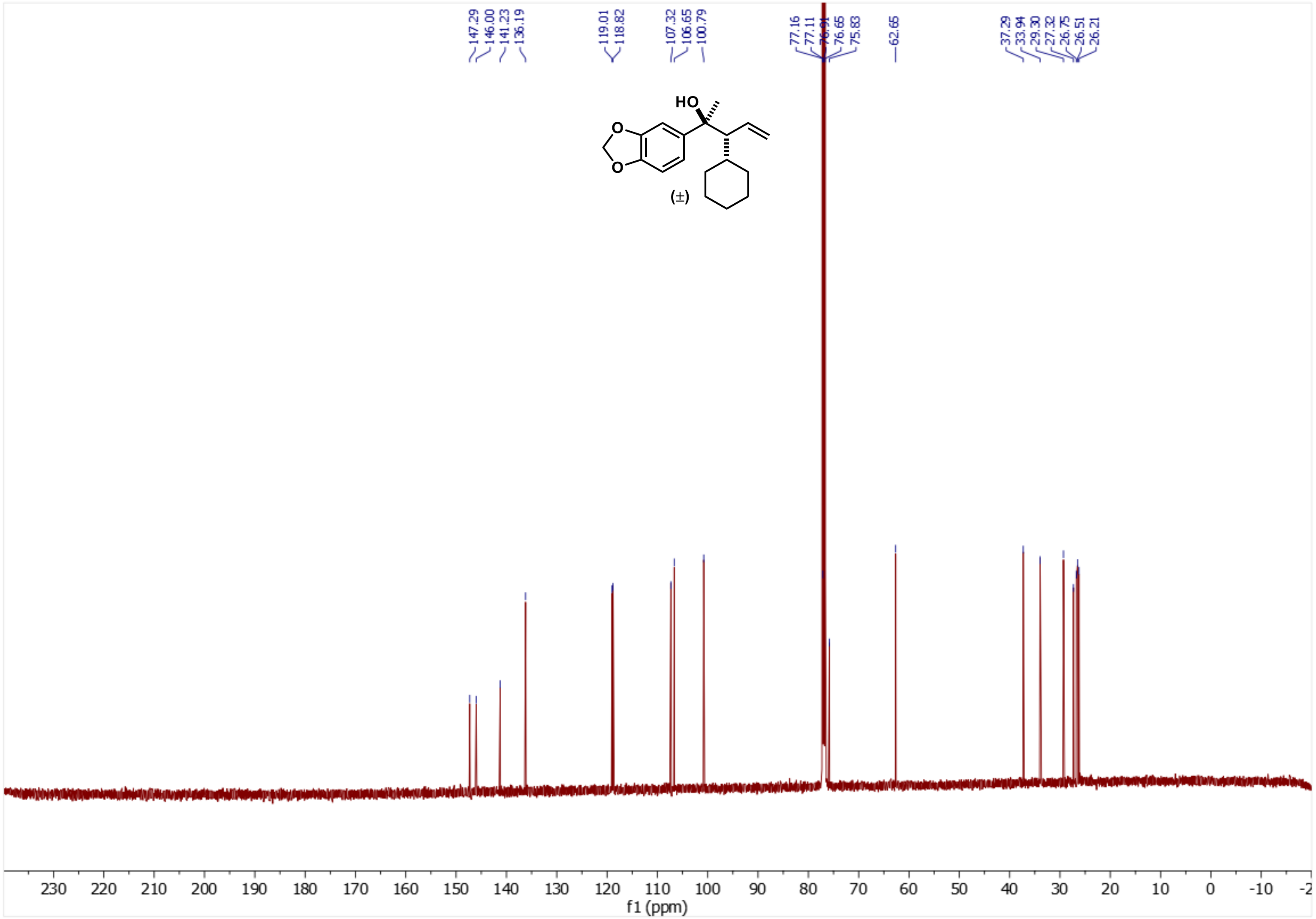

^{13}C NMR (126 MHz, $CDCl_3$) Spectra of (2*SR*,3*RS*)-3-cyclohexyl-2-(benzo[d][1,3]dioxol-6-yl))pent-4-en-2-ol

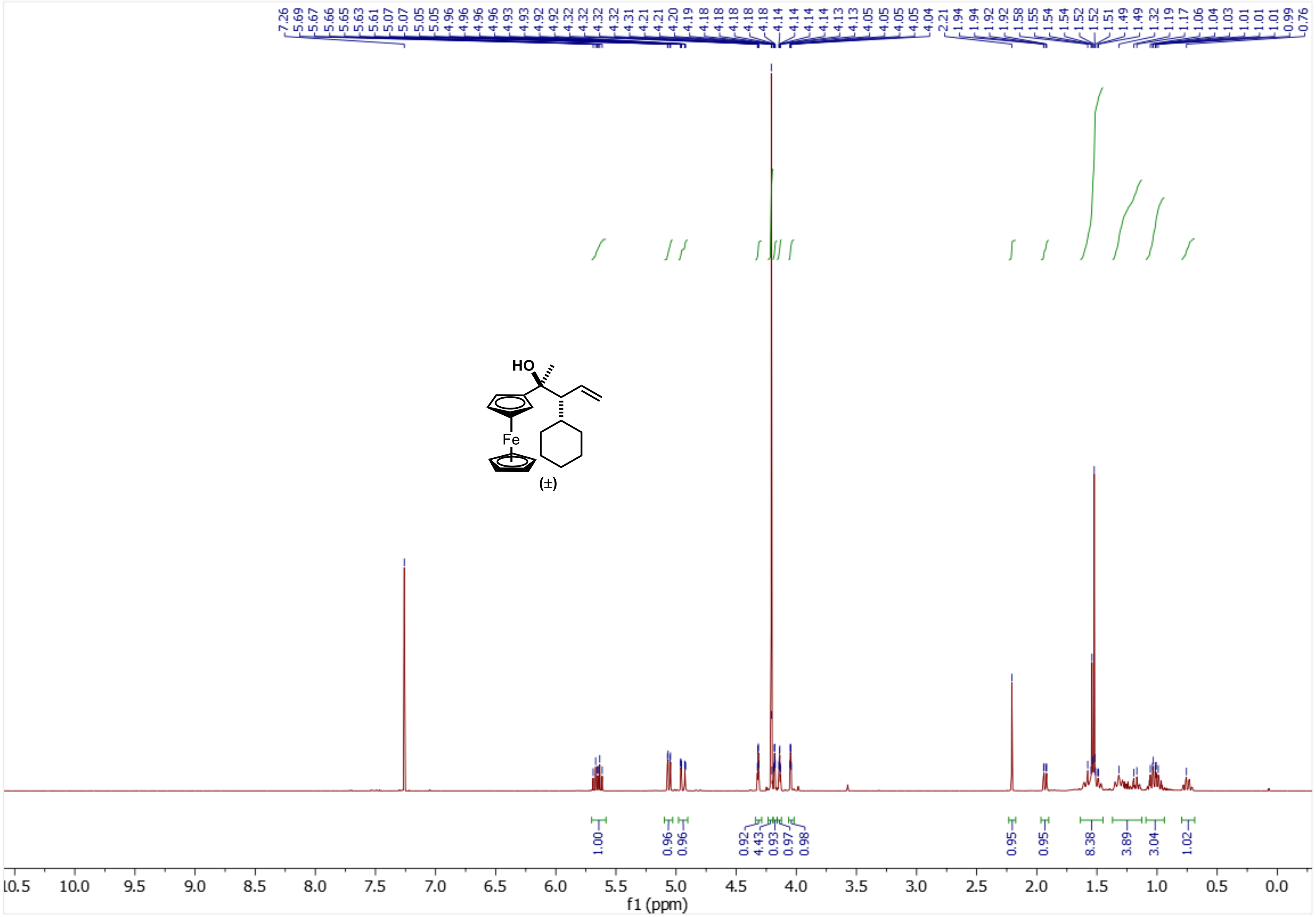

^{1}H NMR (500 MHz, $CDCl_3$) Spectra of (2*SR*,3*RS*)-3-cyclohexyl-2-ferrocenylpent-4-en-2-ol

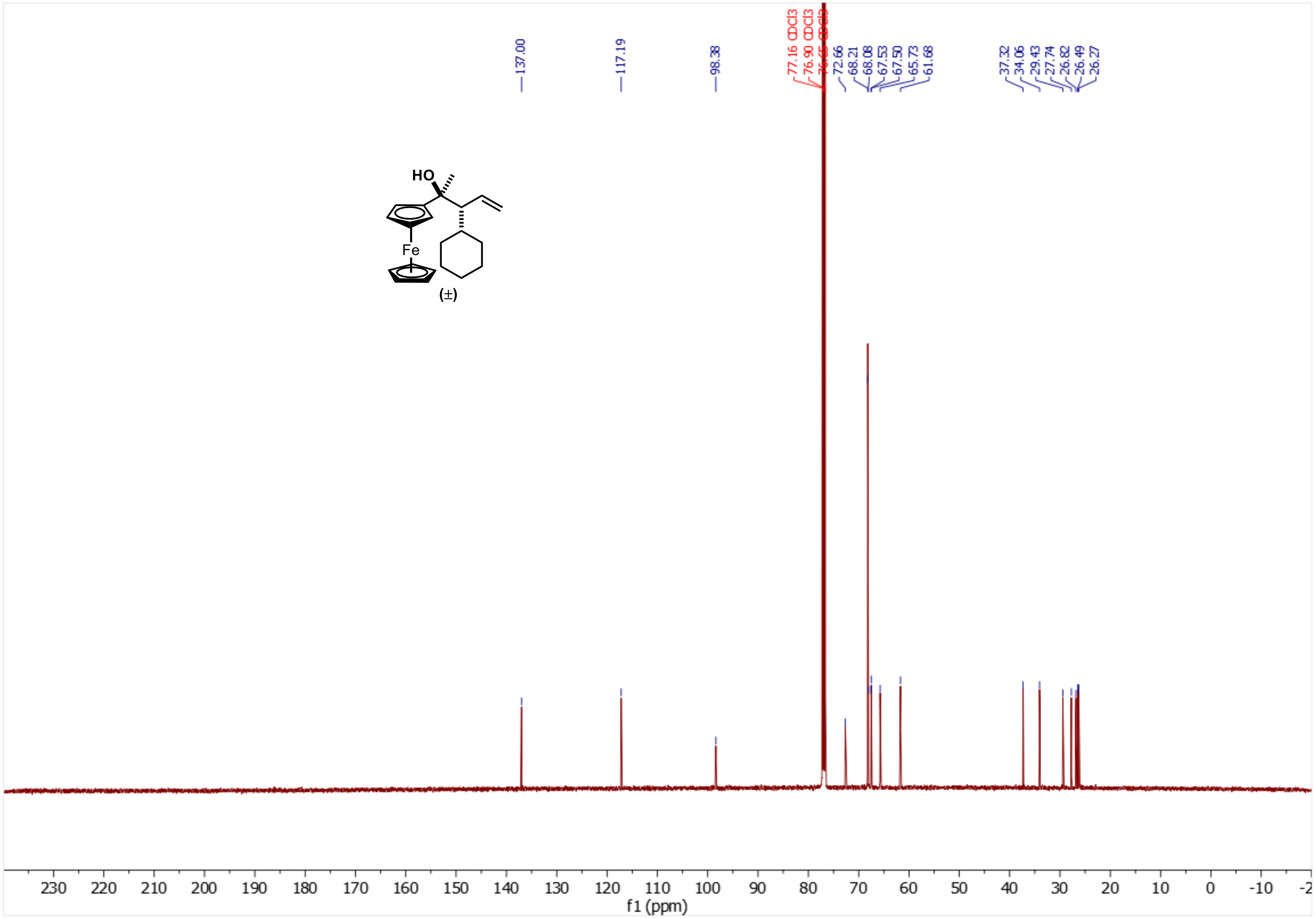

^{13}C NMR (126 MHz, $CDCl_3$) Spectra of (2*SR*,3*RS*)-3-cyclohexyl-2-ferrocenylpent-4-en-2-ol

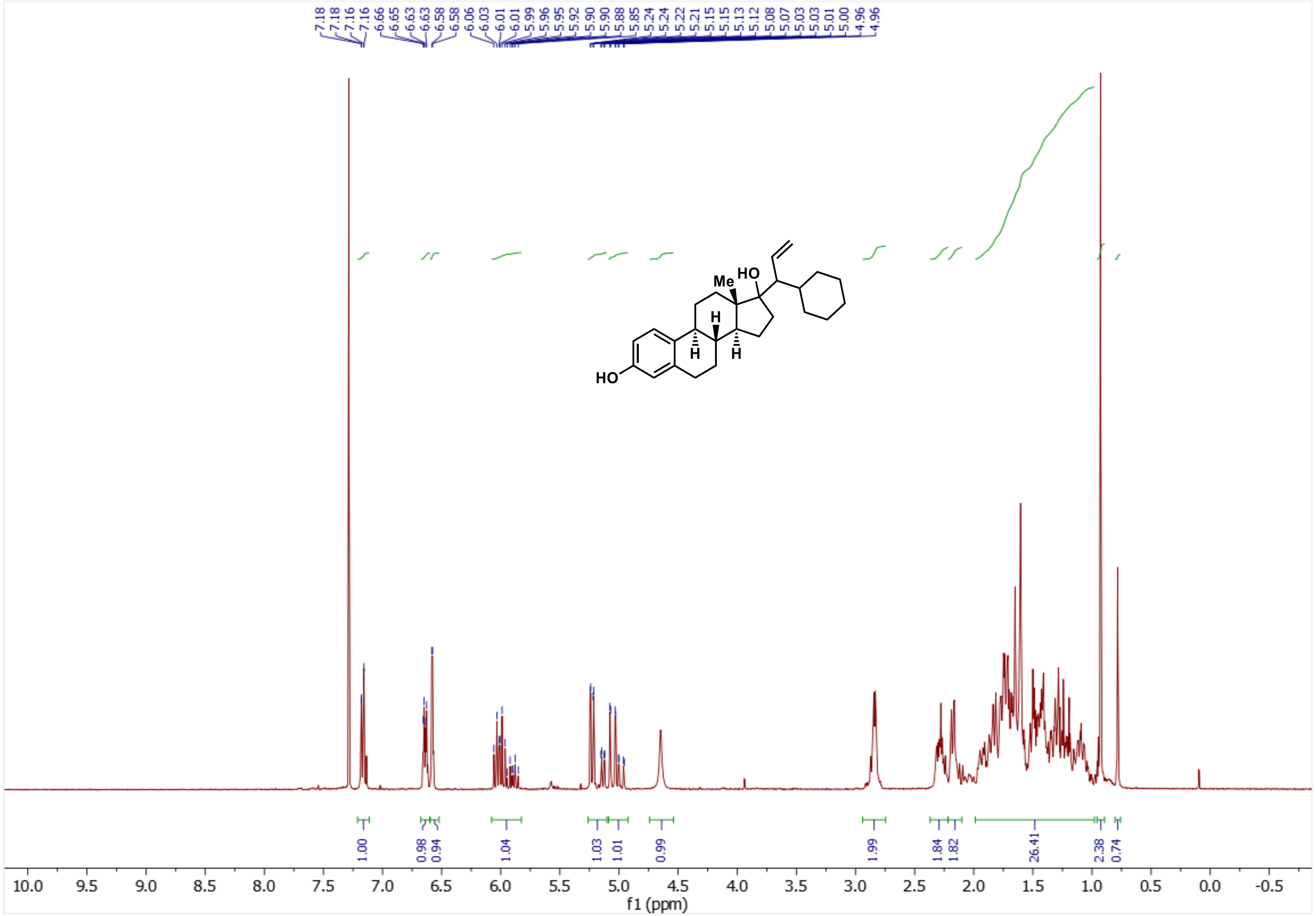

^{1}H NMR (500 MHz, $CDCl_3$) Spectra of 3-cylohexylprop-1-en-3-estradiol

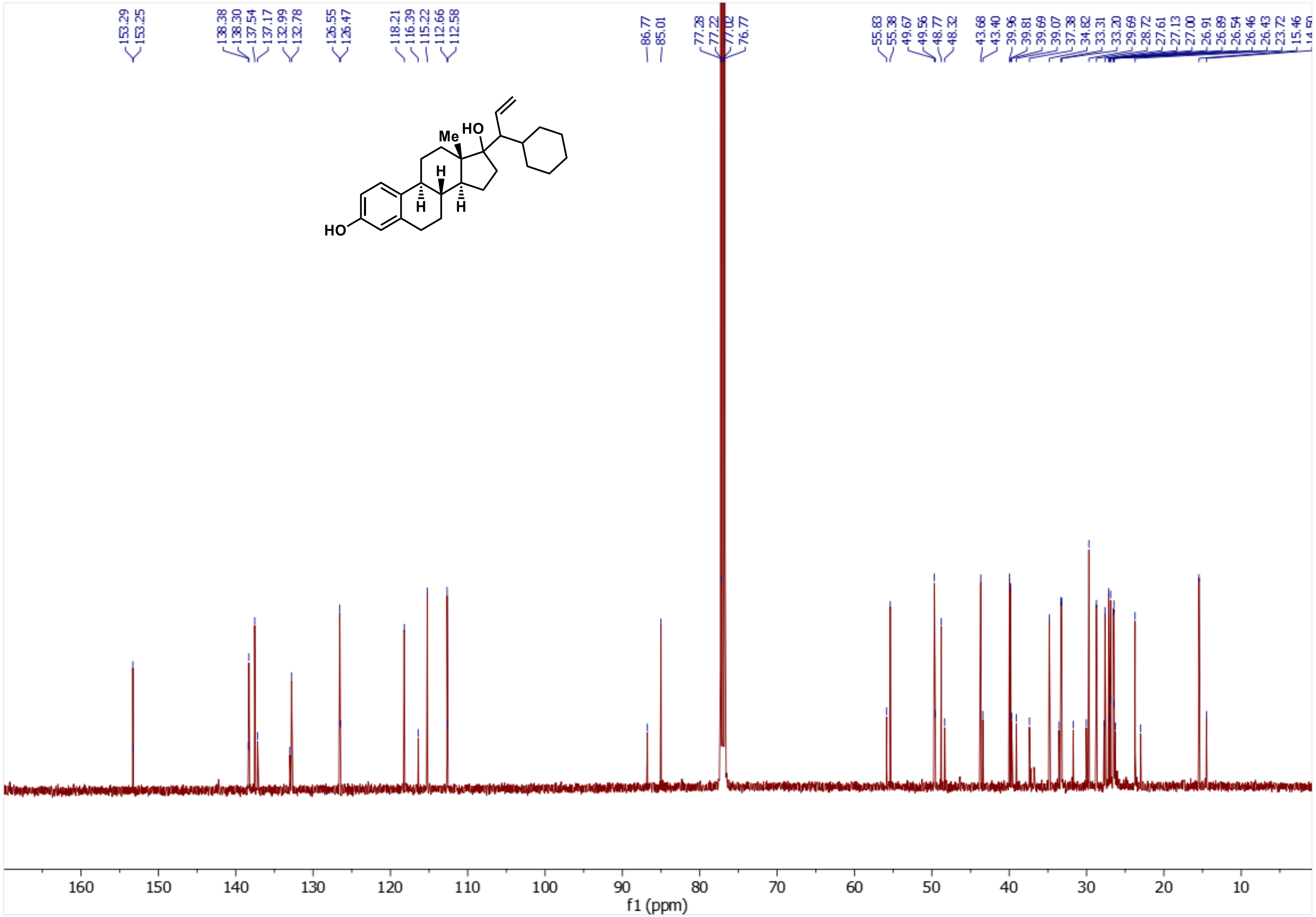

^{13}C NMR (126 MHz, $CDCl_3$) Spectra of 3-cylohexylprop-1-en-3-estradiol

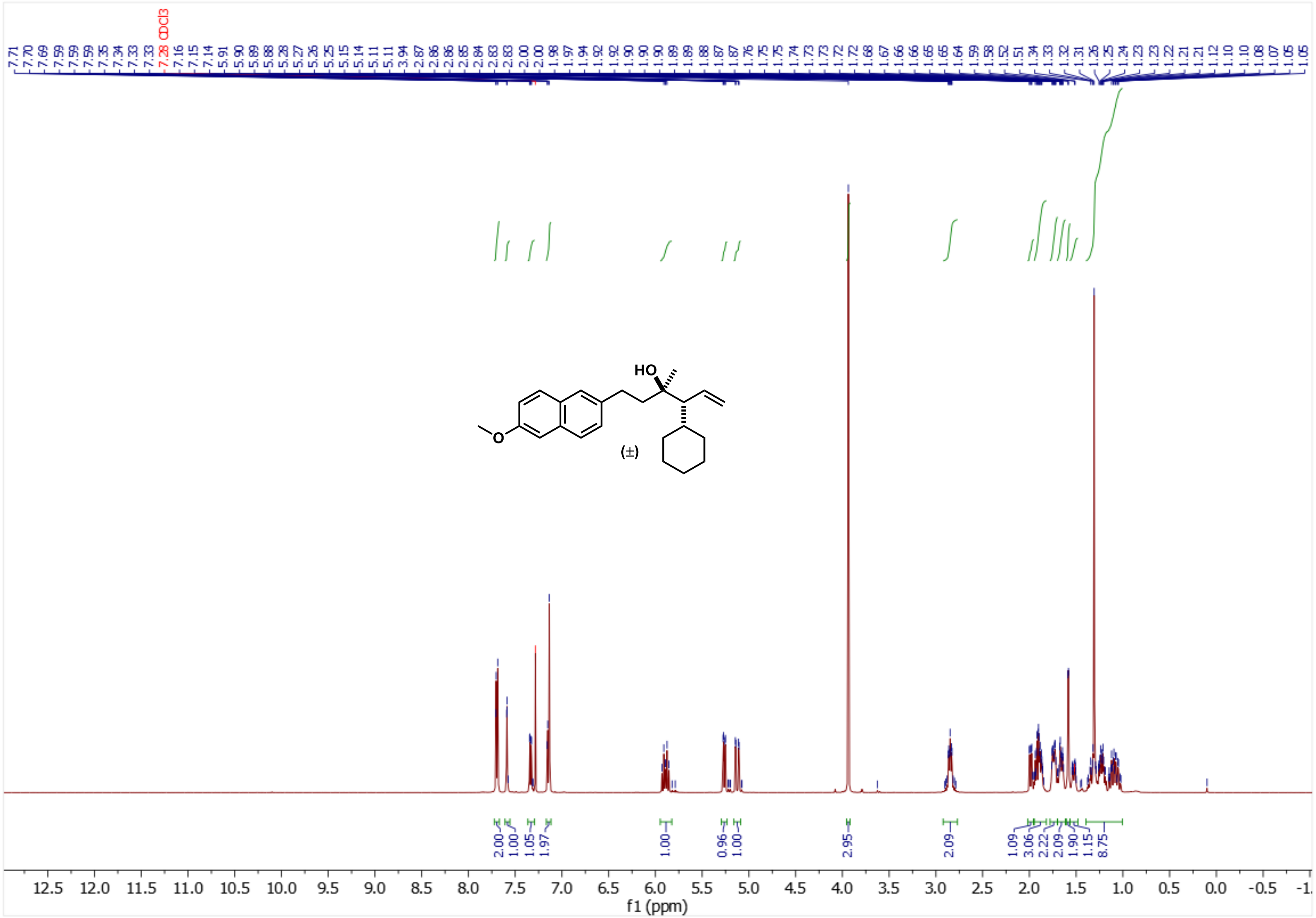

^{1}H NMR (600 MHz, $CDCl_3$) Spectra of (2*SR*,3*SR*)4-(2-(7-methoxynaphthalene)-2- ethenylcyclohexyl-butan-2-ol

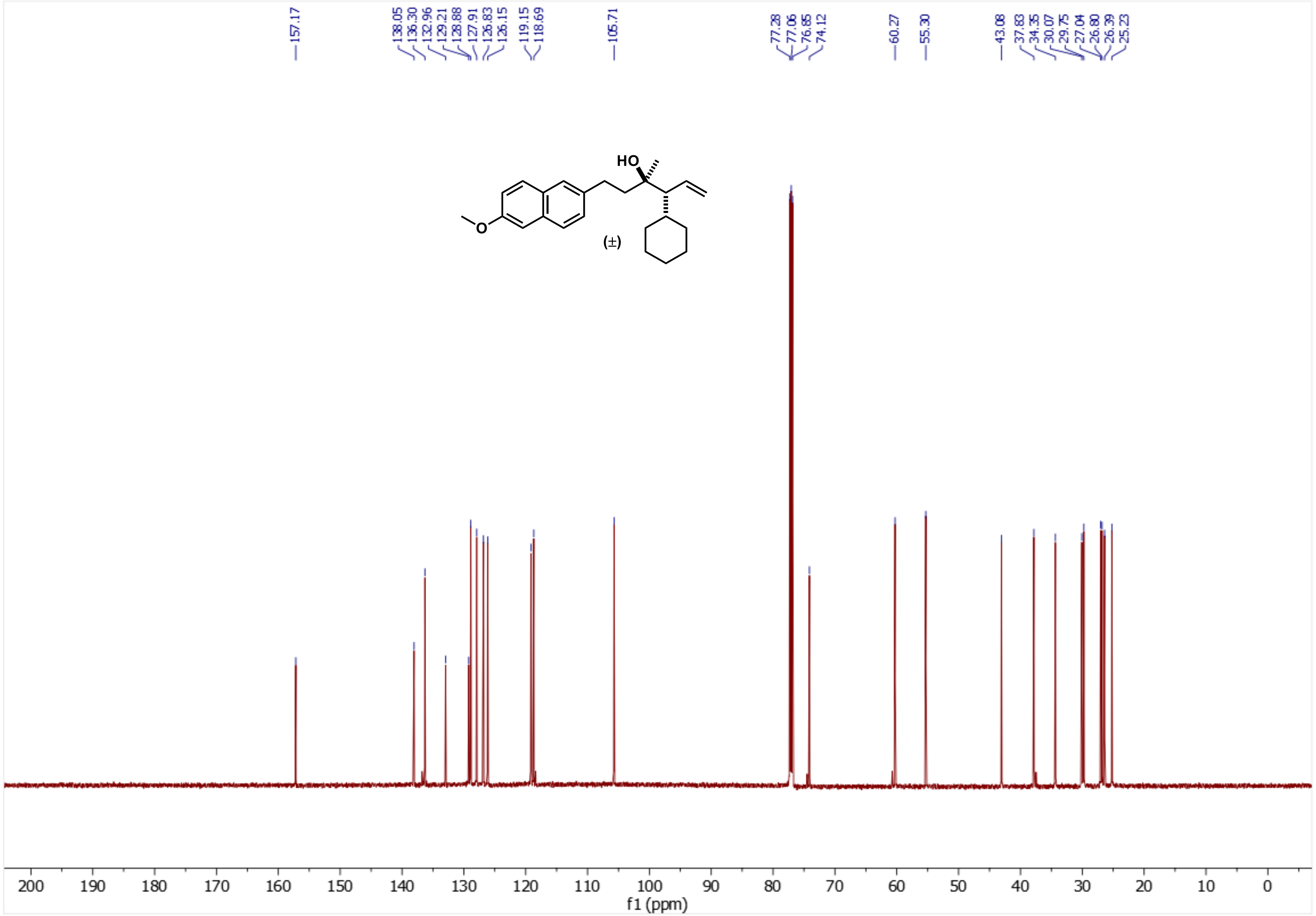

^{13}C NMR (151 MHz, $CDCl_3$) Spectra of (2*SR*,3*SR*)4-(2-(7-methoxynaphthalene)-2- ethenylcyclohexyl-butan-2-ol

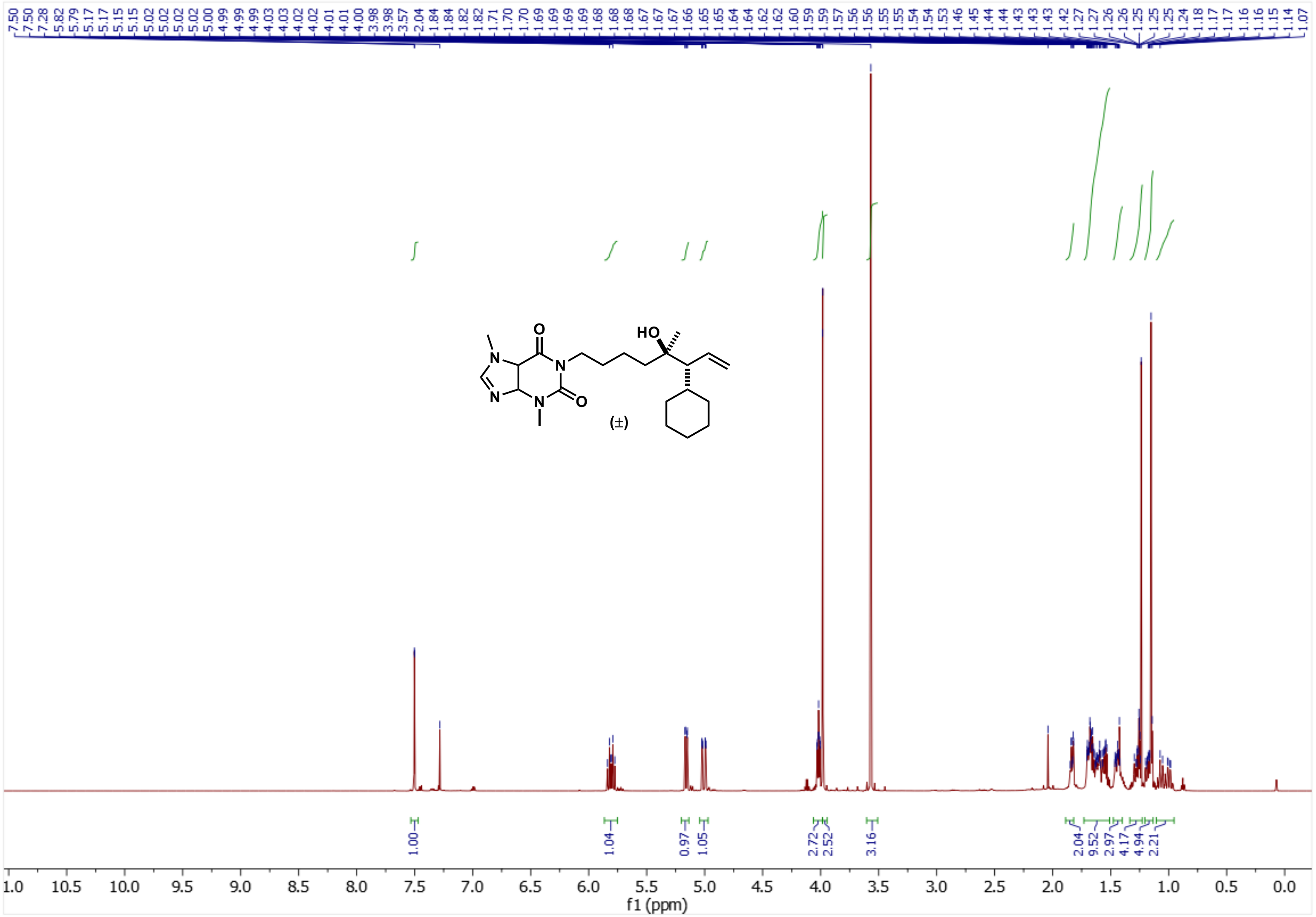

^{1}H NMR (500 MHz, $CDCl_3$) Spectra of (5*SR*,6*RS*)-1-(6-cyclohexyl-5-hydroxy-5-methyloct-7-enyl)-7-methylxanthine

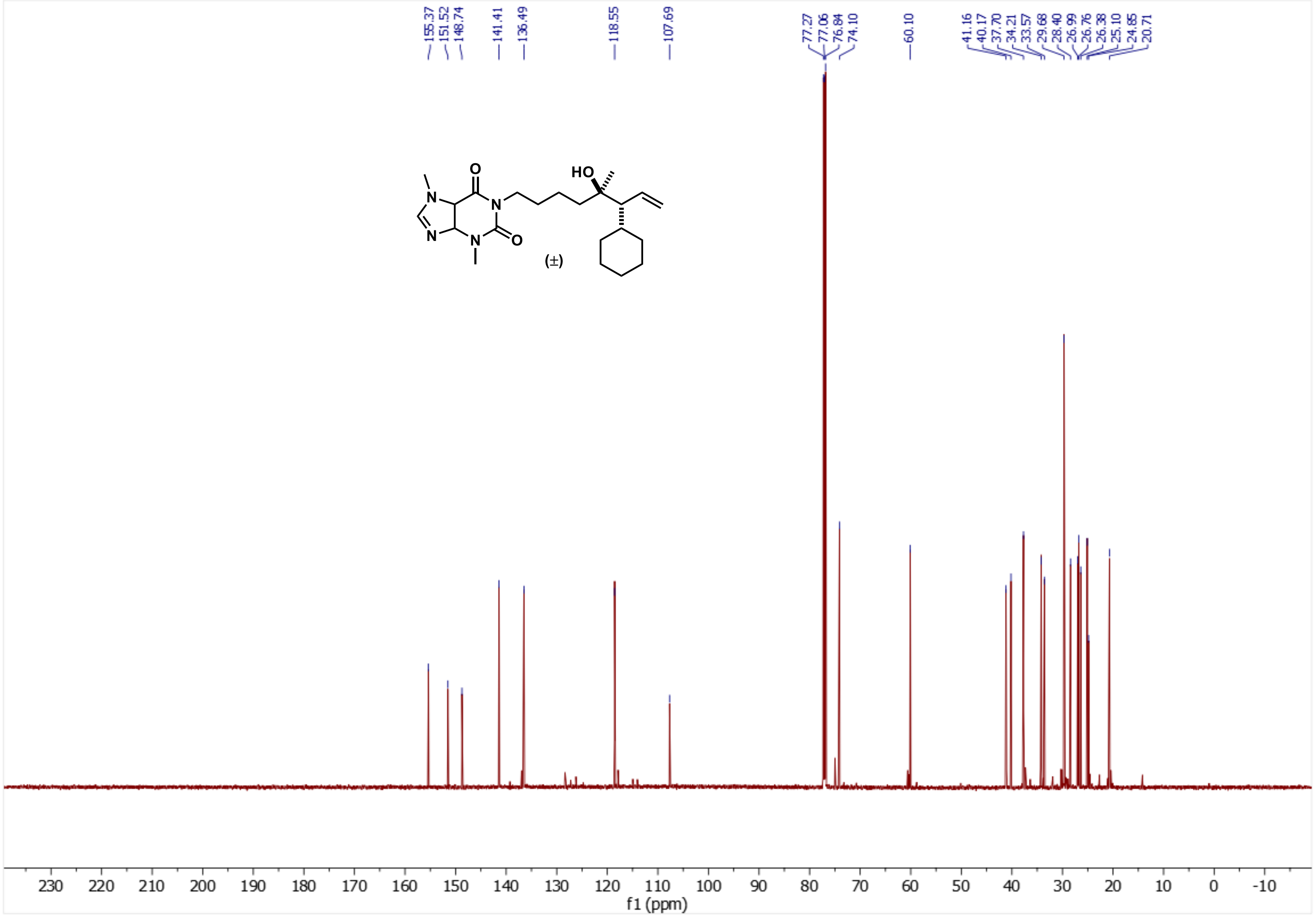

13C NMR (126 MHz, $CDCl_3$) Spectra of (5*SR*,6*RS*)-1-(6-cyclohexyl-5-hydroxy-5-methyloct-7-enyl)-7-methylxanthine

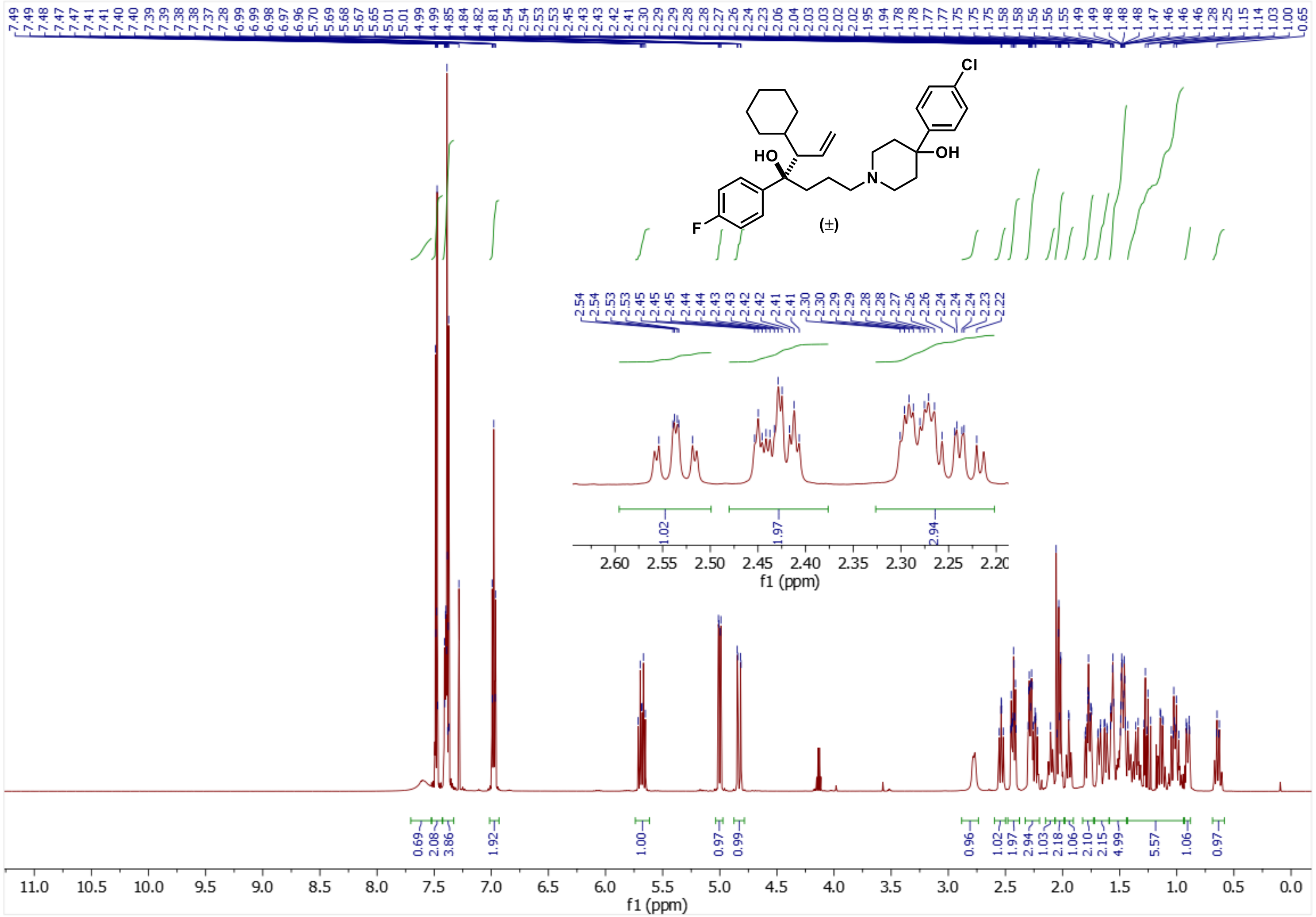

^{1}H NMR (500 MHz, $CDCl_3$) Spectra of (2*SR*,3*RS*)-3-cyclohexyl-2-haloperidiol

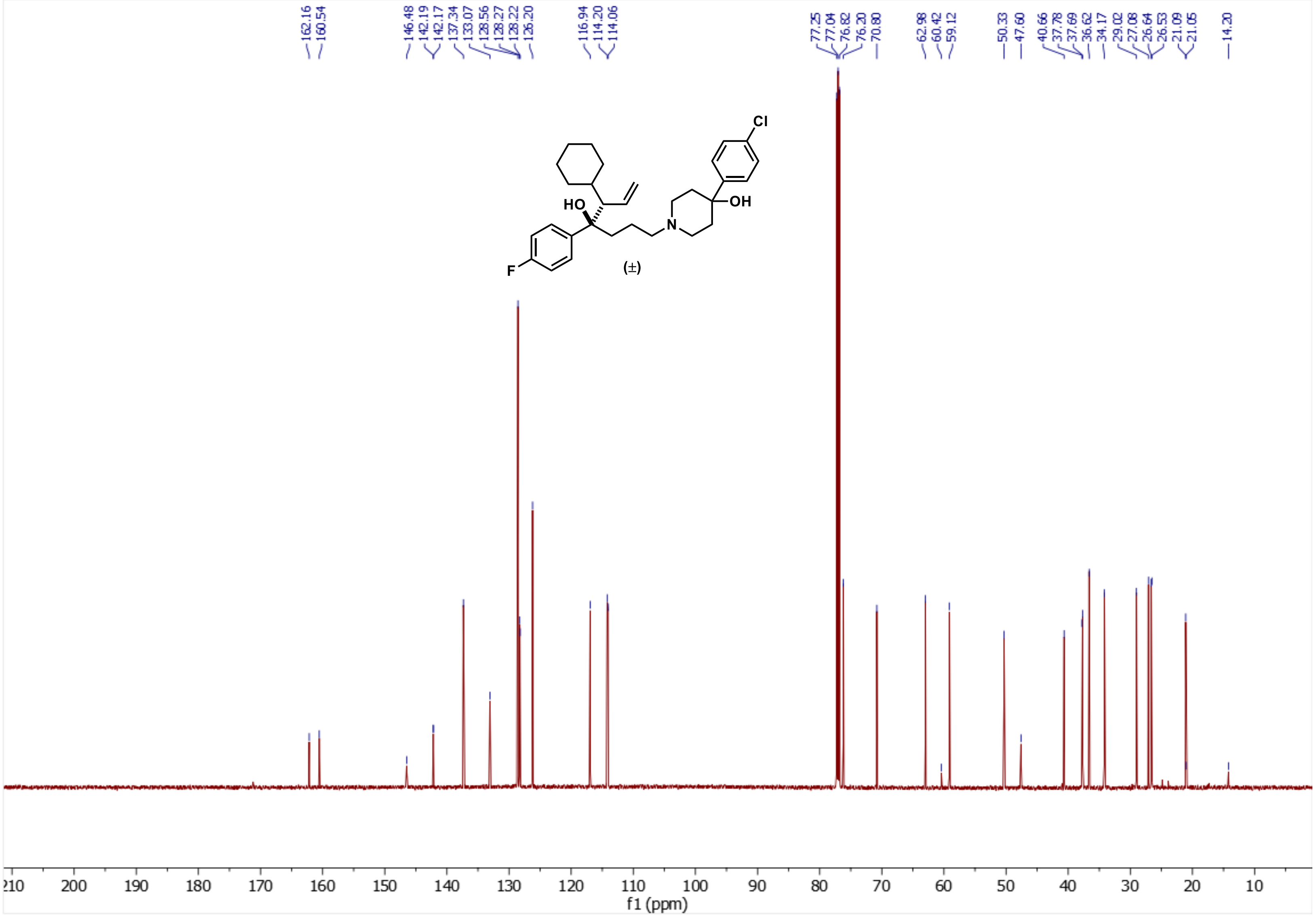

^{13}C NMR (126 MHz, $CDCl_3$) Spectra of (2*SR*,3*RS*)-3-cyclohexyl-2-haloperidiol

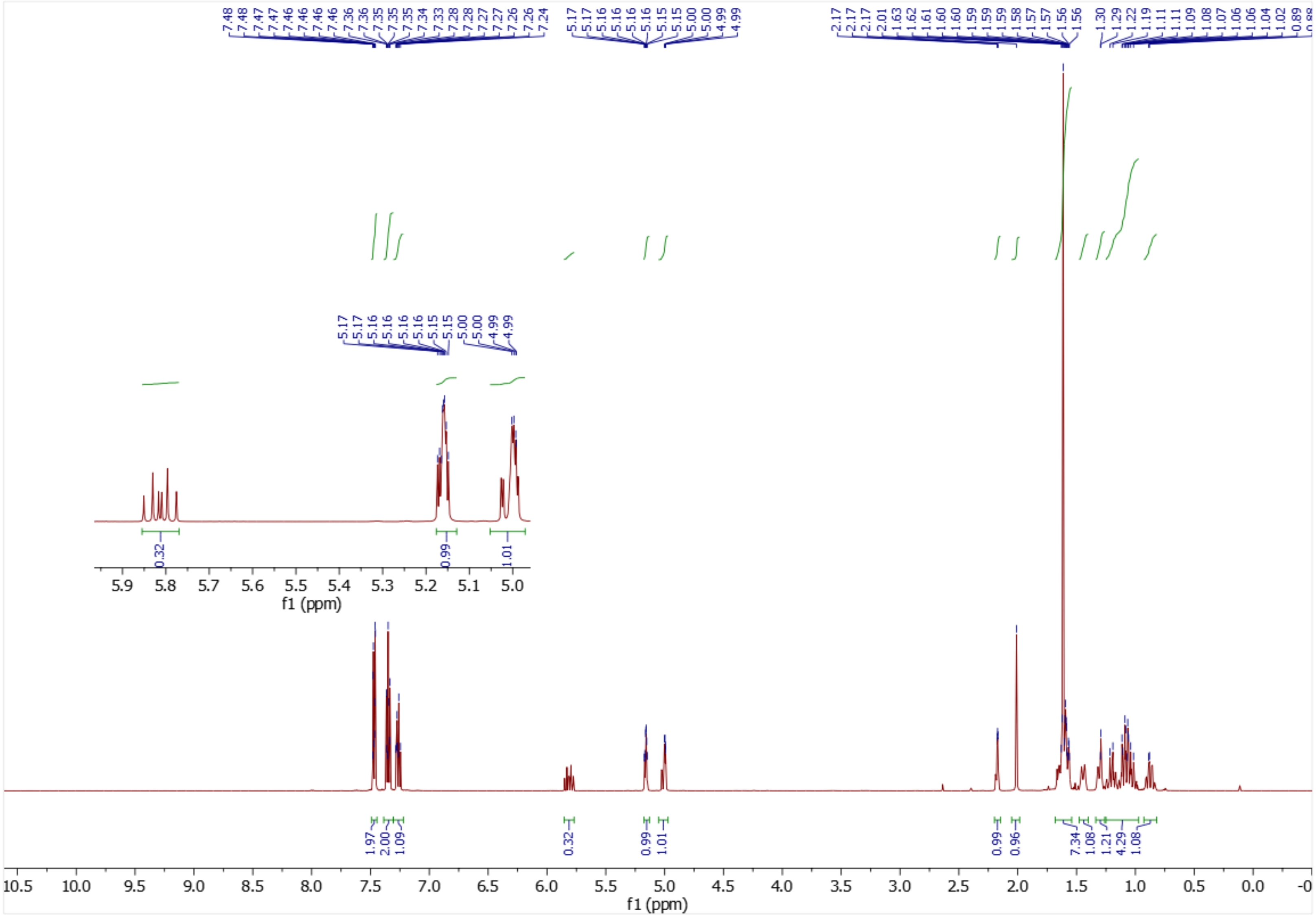

^{1}H NMR (500 MHz, $CDCl_3$) Spectra of (2*SR*,3*RS*)-2-Deuterio-3-cyclohexyl-2-phenylpent-4-en-2-ol **3aD**

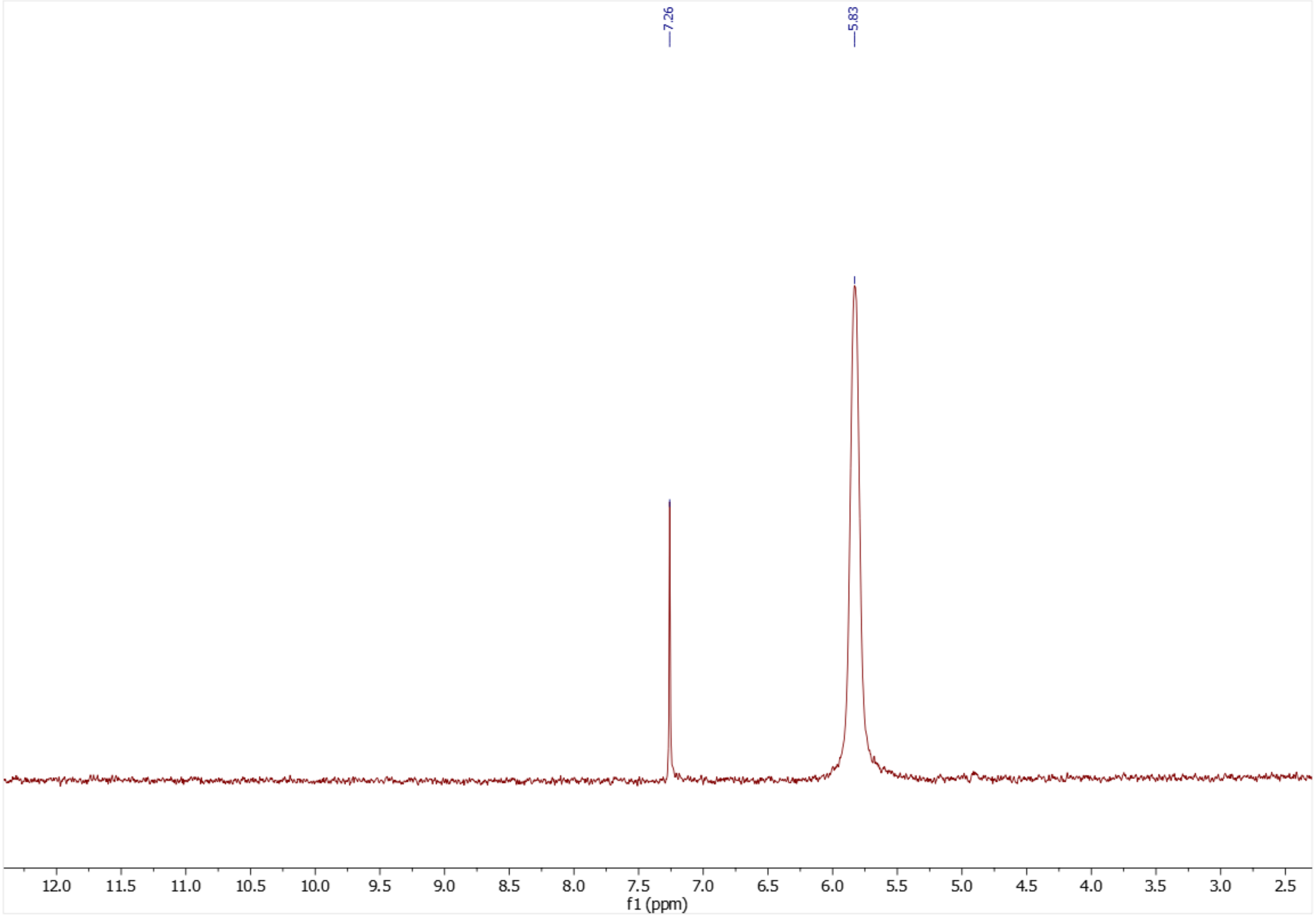

^{2}H NMR (77 MHz, $CDCl_3$) Spectra of (2*SR*,3*RS*)-2-Deuterio-3-cyclohexyl-2-phenylpent-4-en-2-ol **3aD**

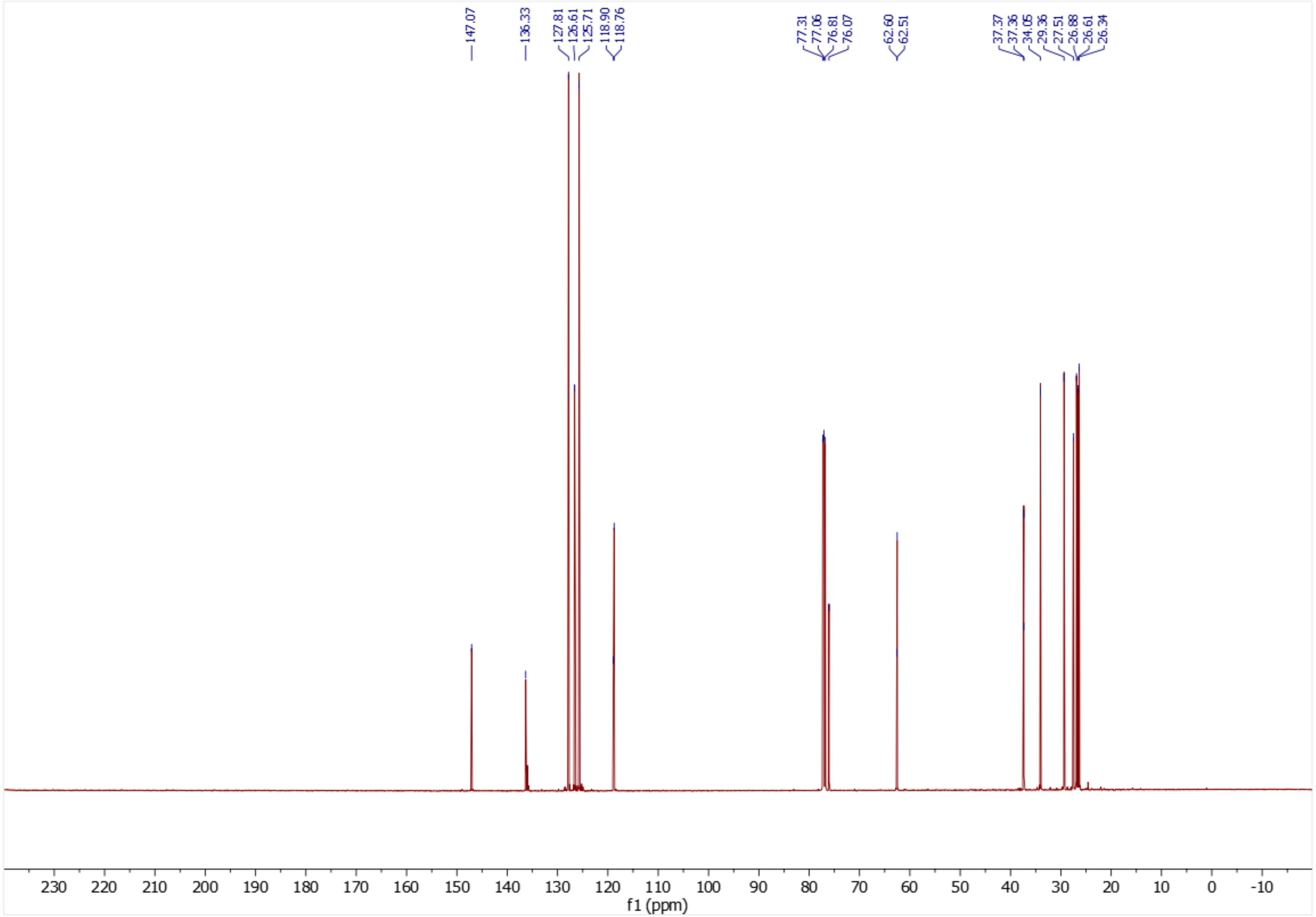

^{13}C NMR (126 MHz, $CDCl_3$) Spectra of (2*SR*,3*RS*)-2-Deuterio-3-cyclohexyl-2-phenylpent-4-en-2-ol **3aD**

S9 HPLC data

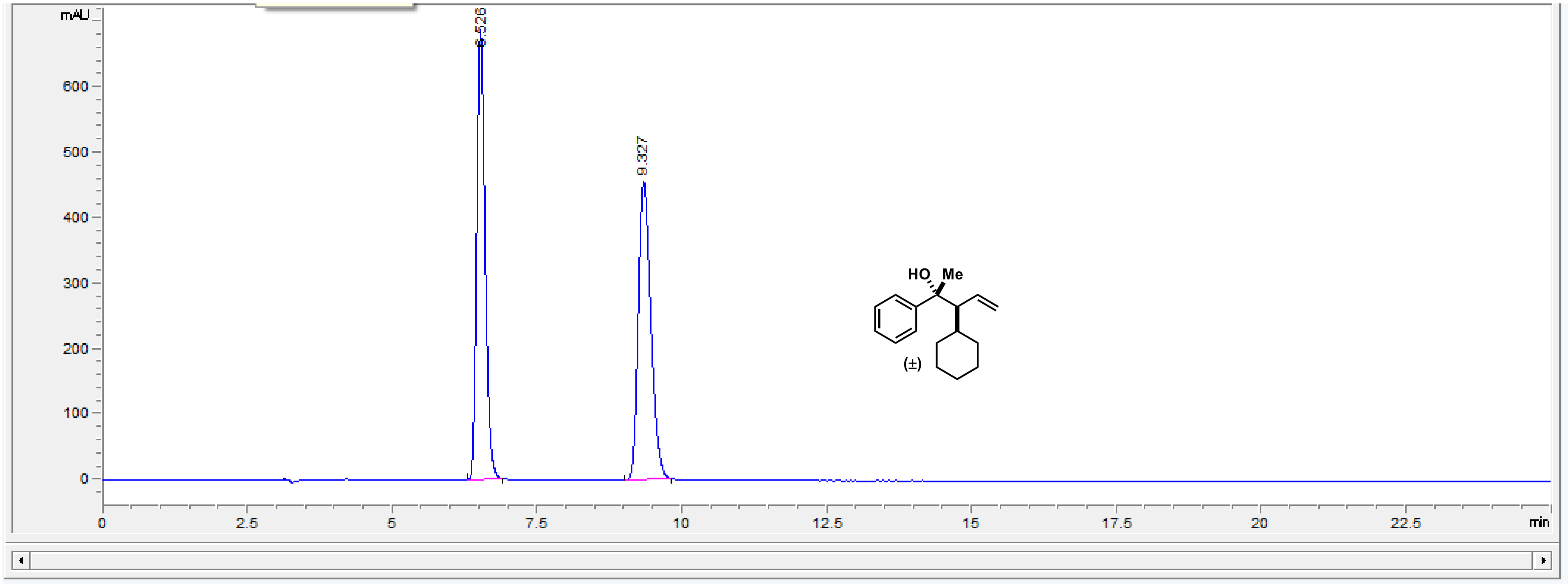

File Information	
LC-File	KN08-076-RAC.D
File Path	C:\CHEM32\1\DATA\DC\JOANNE 2022-03-03 15-16-21\

#	Time	Area	Height	Width	Area%	Symmetry
1	6.526	6737.8	688	0.1503	49.702	0.783
2	9.327	6818.5	456.6	0.1997	50.298	0.698

95/5 ODH-(2*RS*,3*SR*)-3-cyclohexyl-2-phenylpent-4-en-2-ol

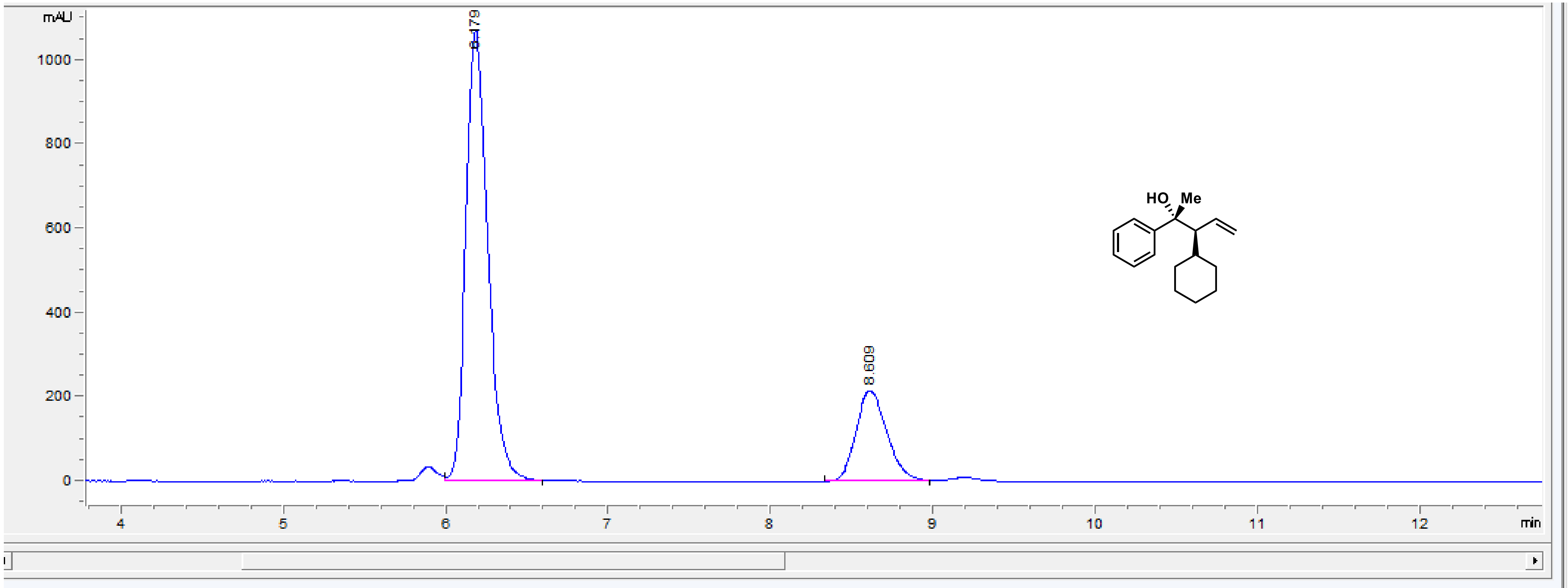

	File Information
LC-File	KN08-153A.D
File Path	C:\CHEM32\1\DATA\DC\JOANNE 2022-03-08 15-07-19\

#	Time	Area	Height	Width	Area%	Symmetry
1	6.179	10138.8	1070.1	0.139	78.104	0.761
2	8.609	2842.4	214.5	0.1756	21.896	0.823

95/5 ODH-(2*R*,3*S*)-3-cyclohexyl-2-phenylpent-4-en-2-ol (from reaction at 80 °C, Table S2 entry 9)

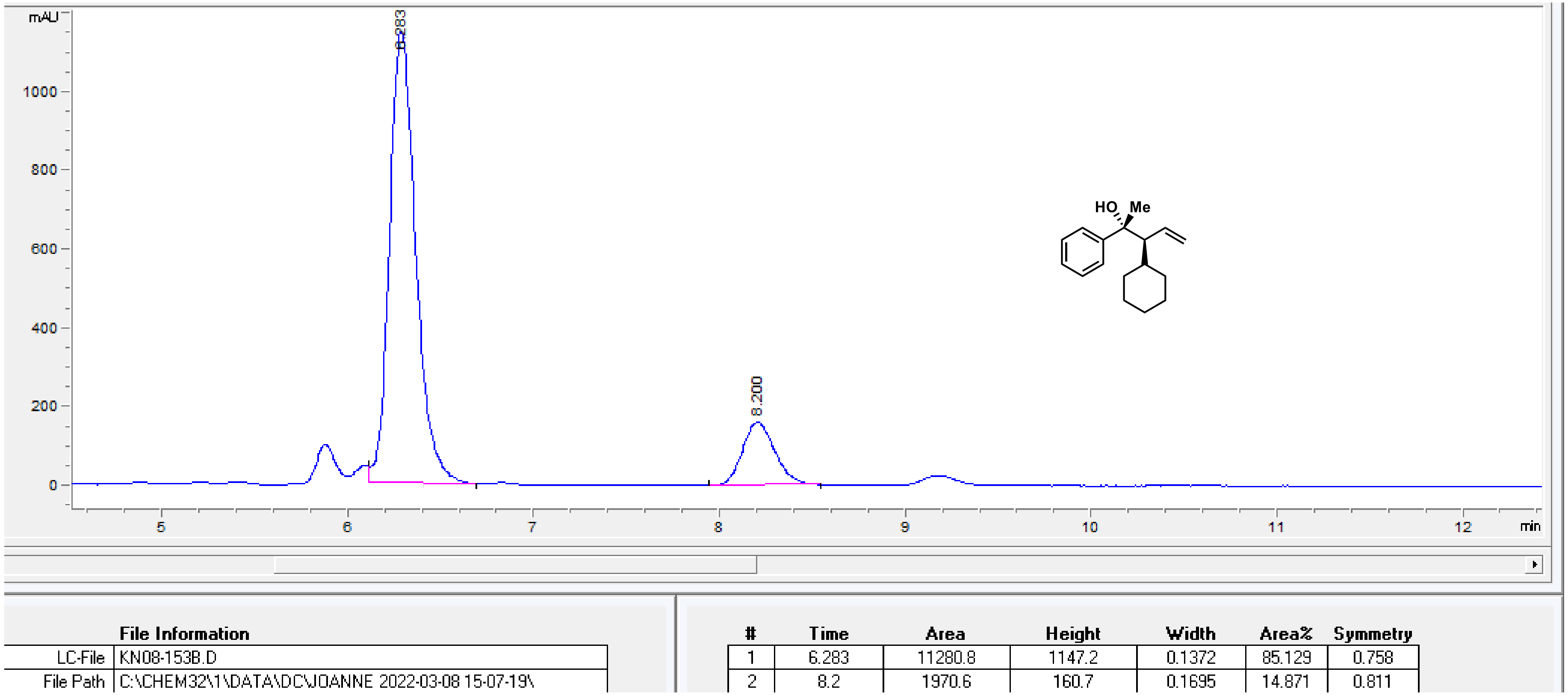

	File Information
LC-File	KN08-153B.D
File Path	C:\CHEM32\1\DATA\DC\JOANNE 2022-03-08 15-07-19\

#	Time	Area	Height	Width	Area%	Symmetry
1	6.283	11280.8	1147.2	0.1372	85.129	0.758
2	8.2	1970.6	160.7	0.1695	14.871	0.811

95/5 ODH-(2*R*,3*S*)-3-cyclohexyl-2-phenylpent-4-en-2-ol (from reaction at 60 °C, Table S2 entry 12)

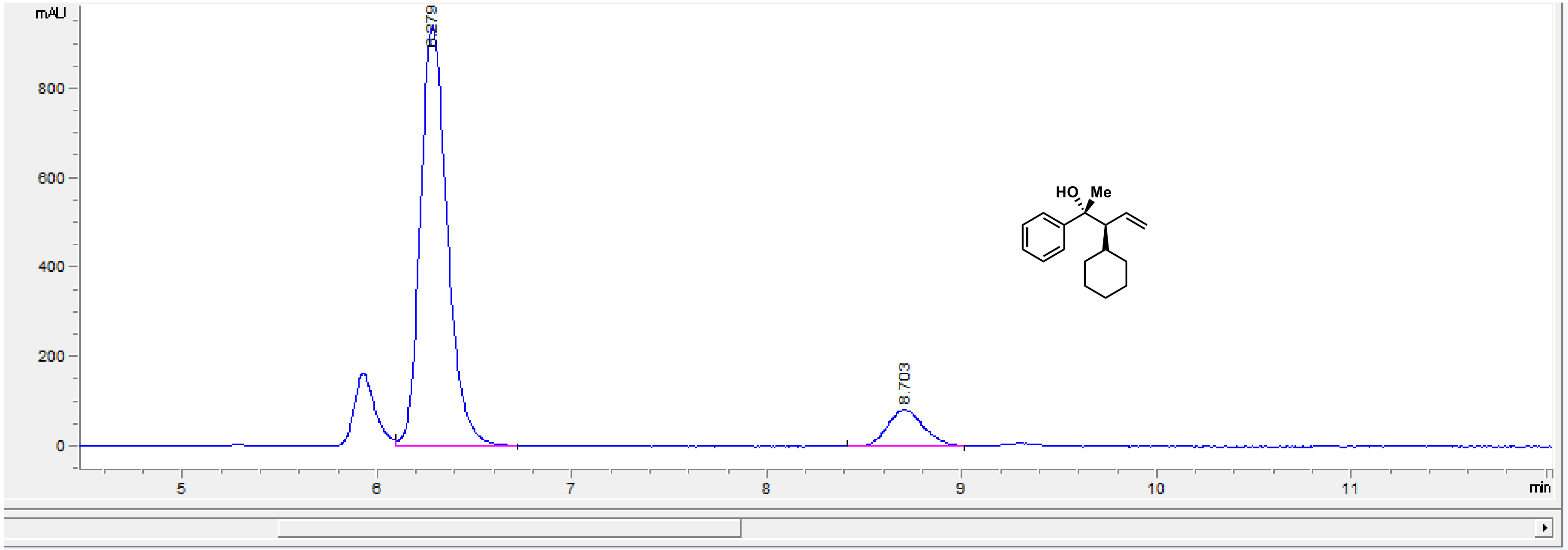

	File Information
LC-File	KN08-154B-2.D
File Path	C:\CHEM32\1\DATA\DC\JOANNE 2022-03-11 13-03-01\

#	Time	Area	Height	Width	Area%	Symmetry
1	6.279	9028.7	939.9	0.1421	89.430	0.761
2	8.703	1067.1	82	0.1553	10.570	0.885

95/5 ODH-(2*R*,3*S*)-3-cyclohexyl-2-phenylpent-4-en-2-ol (from standard reaction conditions Table S2 entry 11)

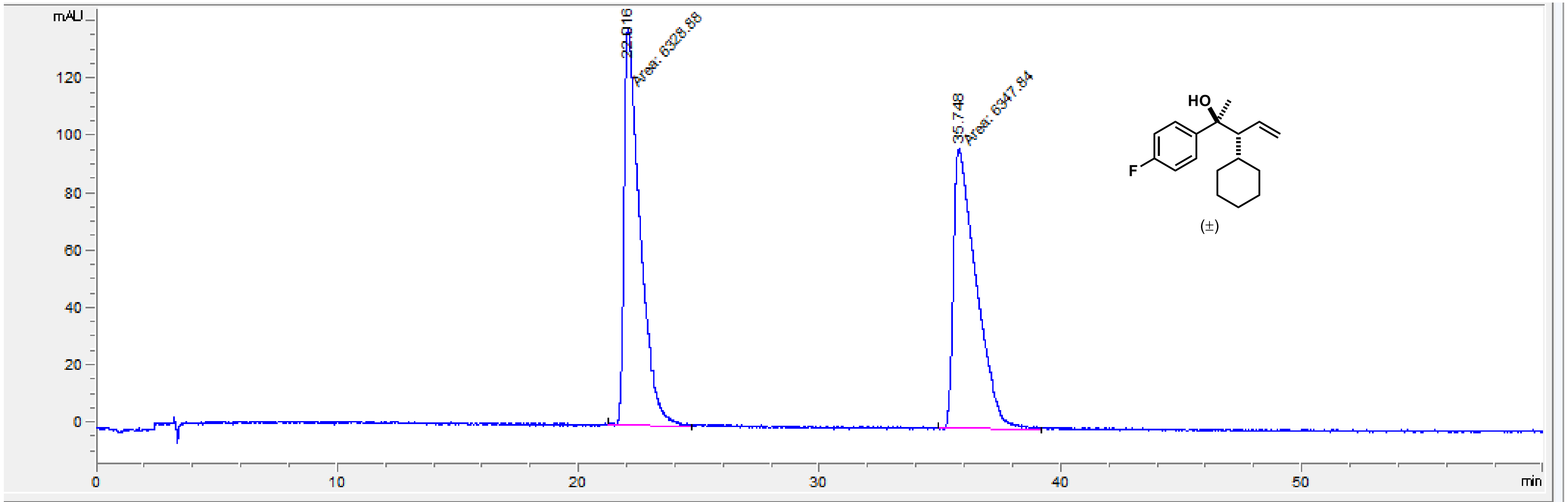

File Information

LC-File	KN08-087.D
File Path	C:\CHEM32\1\DATA\SVEN 2022-03-15 14-15-35\

#	Time	Area	Height	Width	Area%	Symmetry
1	22.016	6328.9	138.3	0.7628	49.925	0.322
2	35.748	6347.8	97.5	1.0846	50.075	0.31

99/1 ODH-(2*SR*,3*RS*)-3-Cyclohexyl-2-(4-fluorophenyl)pent-4-en-2-ol

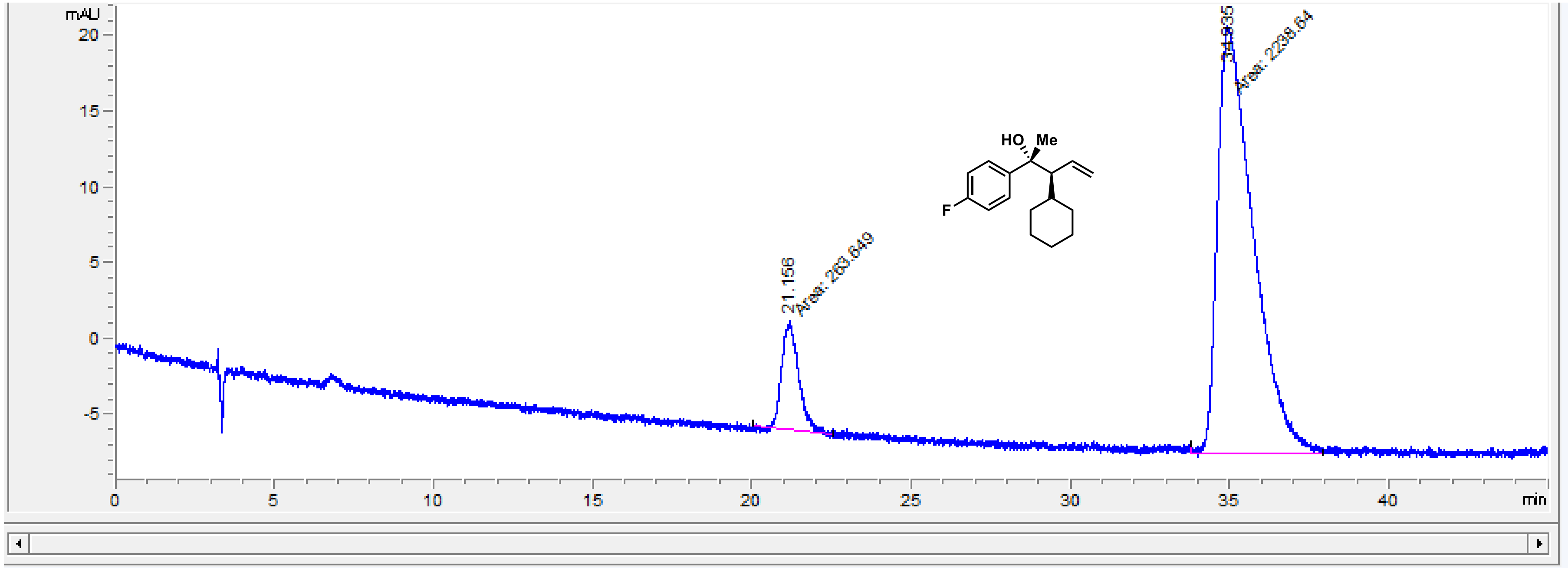

	File Information
LC-File	KN08-160B.D
File Path	C:\CHEM32\1\DATA\DC\JOANNE 2022-07-26 09-51-16\

#	Time	Area	Height	Width	Area%	Symmetry
1	21.156	263.6	7.3	0.6027	10.536	0.716
2	34.935	2238.6	28.2	1.3247	89.464	0.431

99/1 ODH-(2*R*,3*S*)-3-Cyclohexyl-2-(4-fluorophenyl)pent-4-en-2-ol

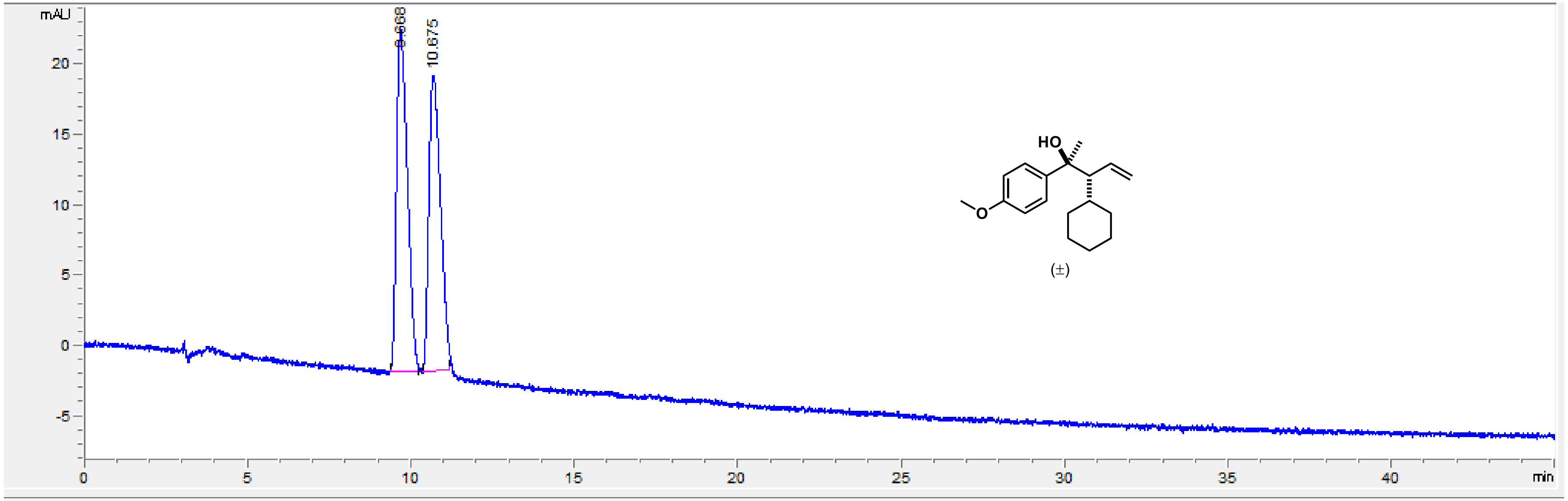

File Information	
LC-File	KN08-092A.D
File Path	C:\CHEM32\1\DATA\DC\JOANNE 2022-03-22 09-09-15\

#	Time	Area	Height	Width	Area%	Symmetry
1	9.668	559.2	24.5	0.2673	51.733	0.531
2	10.675	521.7	21.1	0.295	48.267	0.621

99/1 ODH-(2*SR*,3*RS*)-3-Cyclohexyl-2-(4-methoxyphenyl)pent-4-en-2-ol

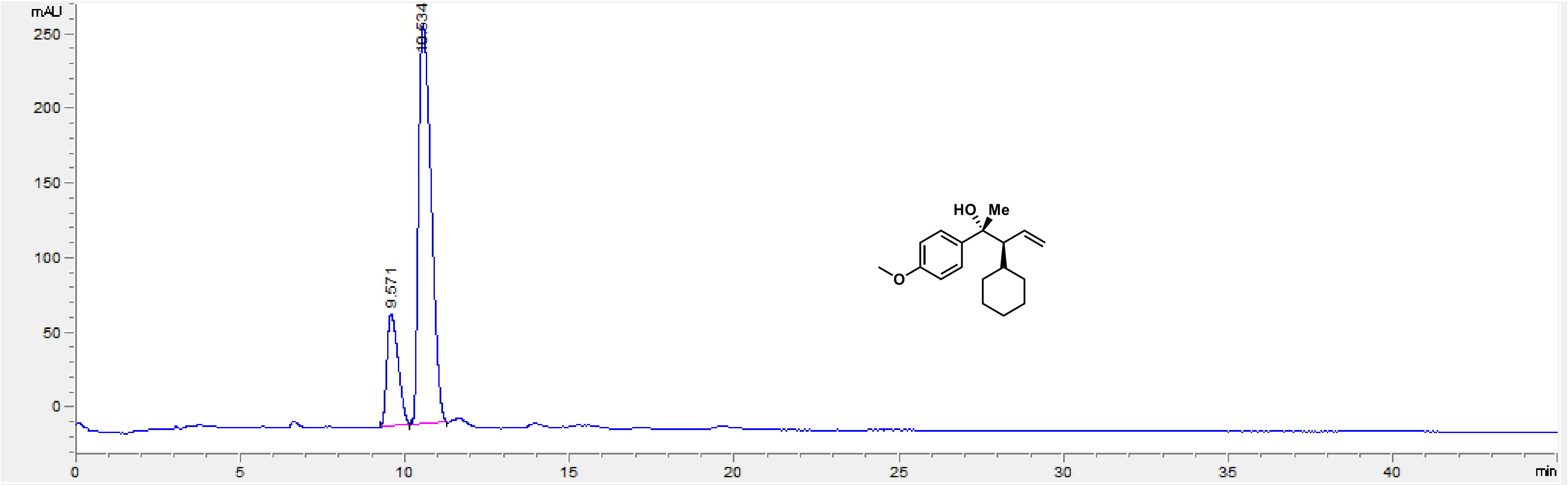

File Information	
LC-File	KN08-162A.D
File Path	C:\CHEM32\1\DATA\DC\JOANNE 2022-03-22 10-22-58\

#	Time	Area	Height	Width	Area%	Symmetry
1	9.571	1798.3	75.7	0.2805	20.221	0.557
2	10.534	7094.7	268.4	0.3114	79.779	0.521

99/1 ODH-(2*R*,3*S*)-3-Cyclohexyl-2-(4-methoxyphenyl)pent-4-en-2-ol

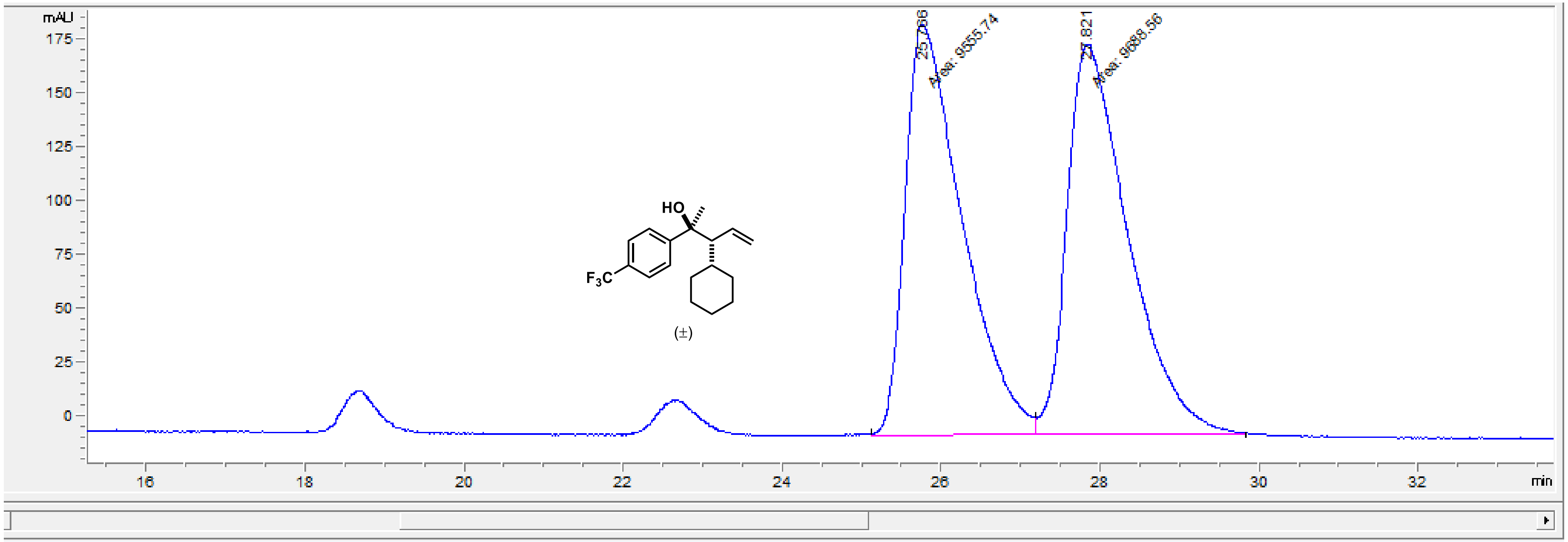

File Information	
LC-File	KN08-092.D
File Path	C:\CHEM32\1\DATA\DC\JOANNE 2022-03-14 11-28-50\

#	Time	Area	Height	Width	Area%	Symmetry
1	25.766	9555.7	190.9	0.8342	49.655	0.46
2	27.821	9688.6	181	0.8919	50.345	0.467

99/1 ODH-(2*SR*,3*RS*)-3-Cyclohexyl-2-(4-(trifluoromethyl)phenyl)pent-4-en-2-ol

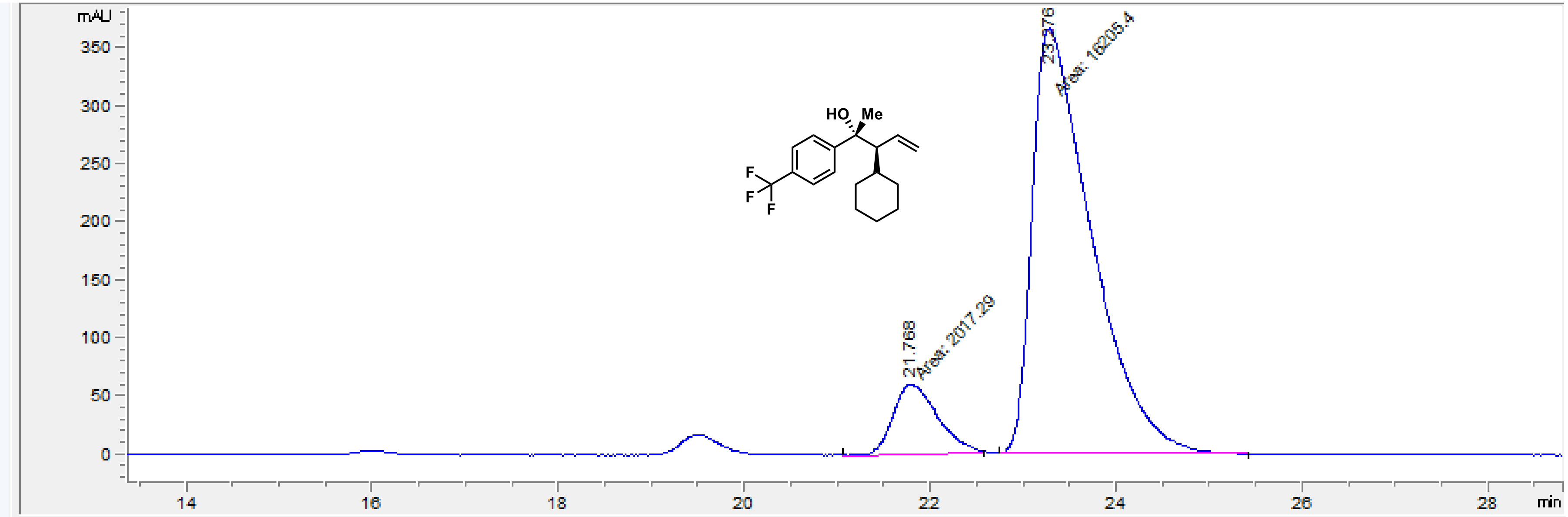

File Information

LC-File	KN08-164D.D
File Path	C:\CHEM32\1\DATA\DC\JOANNE 2022-07-26 09-51-16\

#	Time	Area	Height	Width	Area%	Symmetry
1	21.768	2017.3	60.6	0.5551	11.070	0.591
2	23.276	16205.4	366.6	0.7368	88.930	0.395

99/1 ODH-(2*R*,3*S*)-3-Cyclohexyl-2-(4-(trifluoromethyl)phenyl)pent-4-en-2-ol

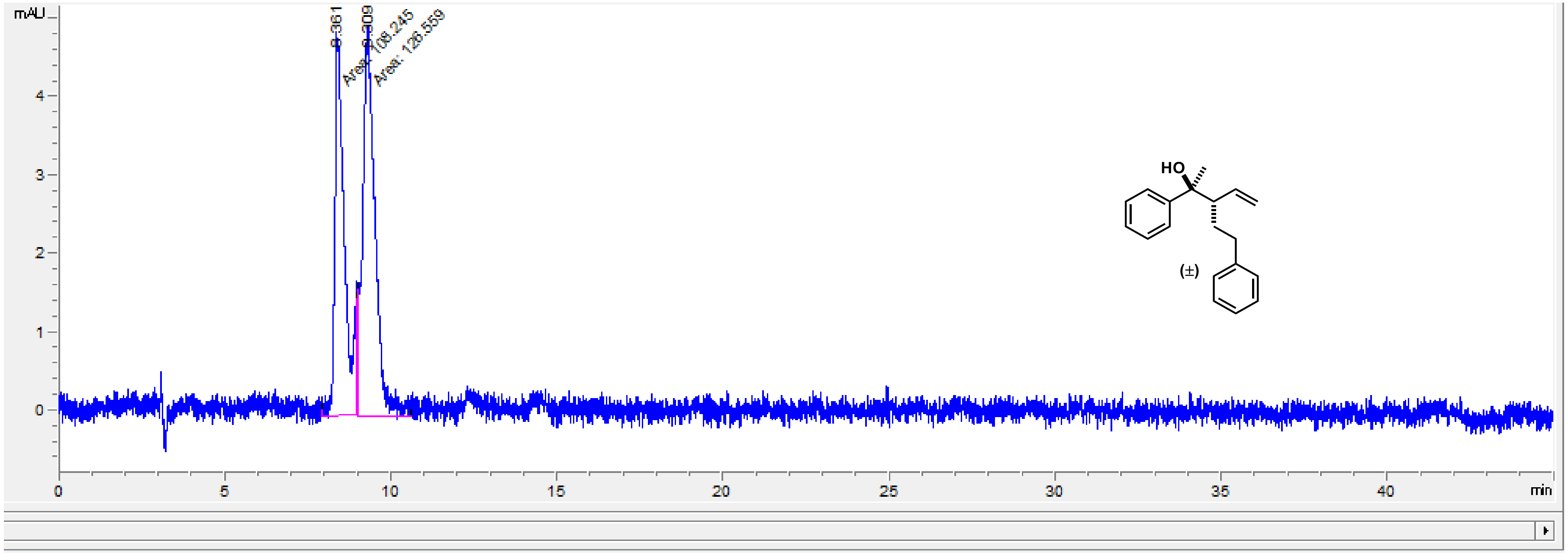

File Information	
LC-File	KN08-020.D
File Path	C:\CHEM32\1\DATA\DC\JOANNE 2022-03-22 12-20-10\

#	Time	Area	Height	Width	Area%	Symmetry
1	8.361	108.2	4.9	0.3673	46.100	0.372
2	9.309	126.6	5	0.4233	53.900	0.678

95/5 ADH-(2*RS*,3*RS*)-3-(1-ethyl-2-phenyl)-2-phenylpent-4-en-2-ol

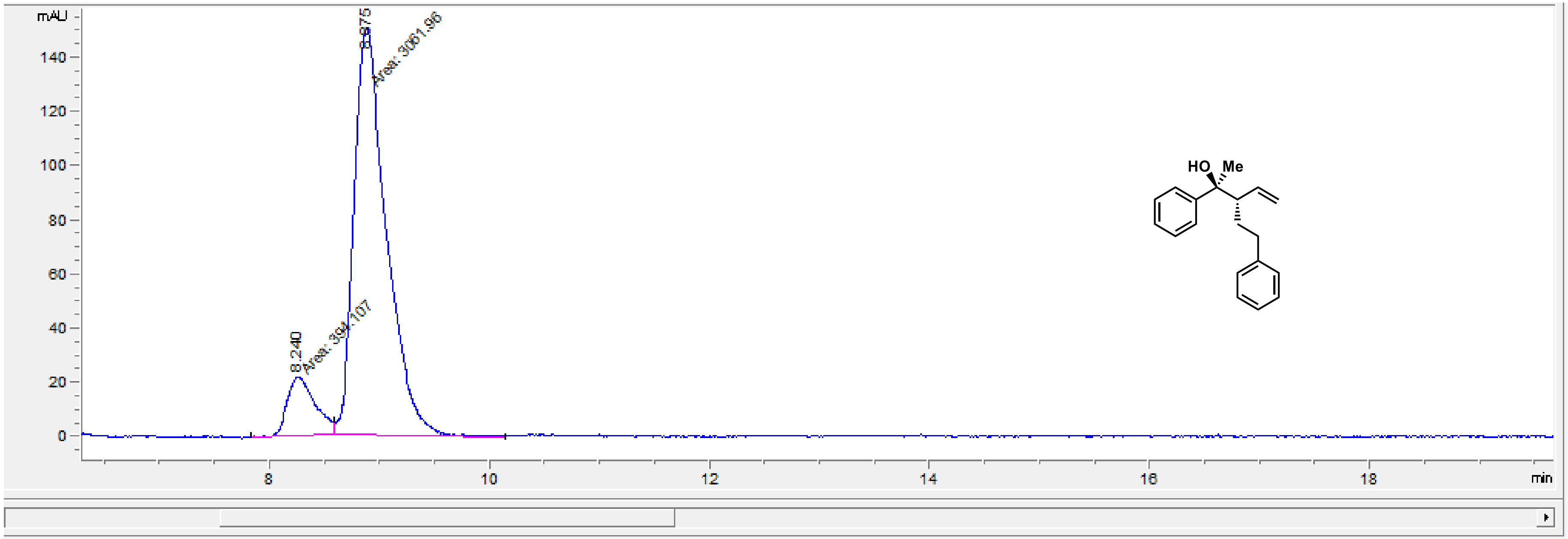

	File Information
LC-File	KN08-187.D
File Path	C:\CHEM32\1\DATA\DC\JOANNE 2022-04-07 13-27-31\

#	Time	Area	Height	Width	Area%	Symmetry
1	8.24	394.1	21.8	0.3008	11.403	0.494
2	8.875	3062	150	0.3403	88.597	0.6

95/5 ADH-(2*S*,3*S*)-3-(1-ethyl-2-phenyl)-2-phenylpent-4-en-2-ol

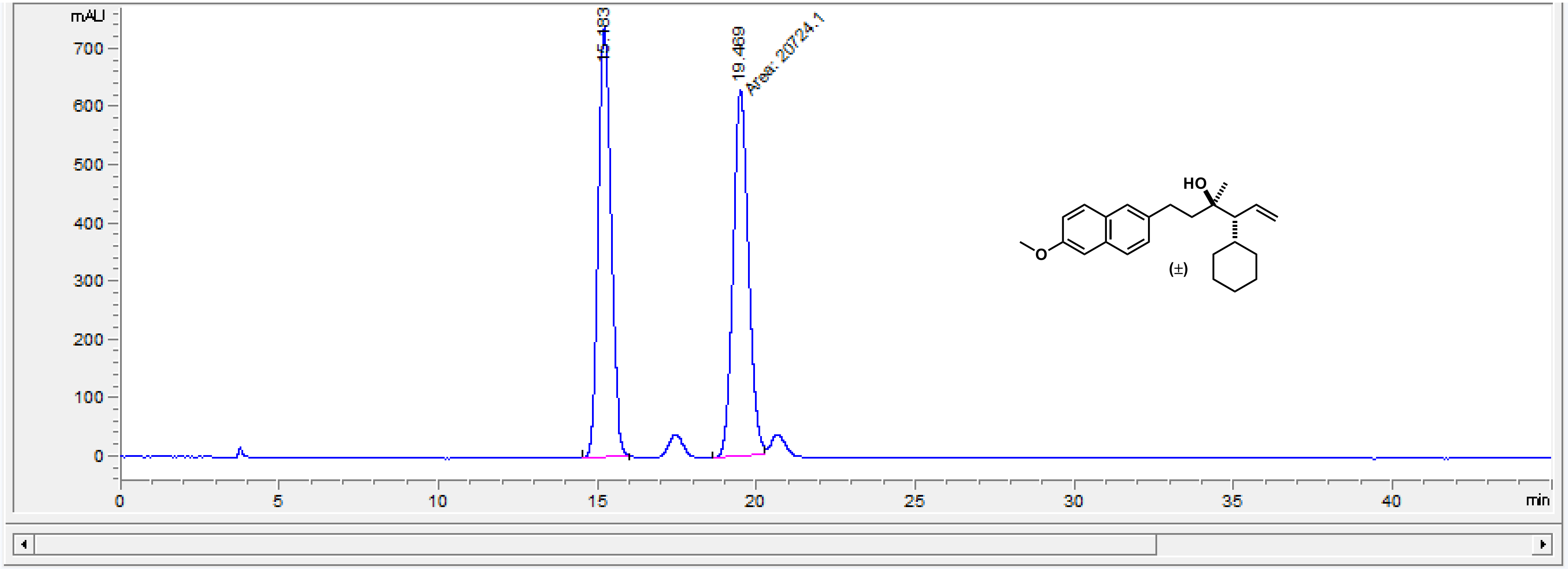

File Information	
LC-File	KN07-121A.D
File Path	C:\CHEM32\1\DATA\DC\JOANNE 2022-07-26 09-51-16\

#	Time	Area	Height	Width	Area%	Symmetry
1	15.183	20732.2	736.8	0.3328	50.010	0.866
2	19.469	20724.1	630.4	0.5479	49.990	0.838

98/2 ODH-(2*SR*,3*SR*)4-(2-(7-methoxynaphthalene)-2- ethenylcyclohexyl-butan-2-ol

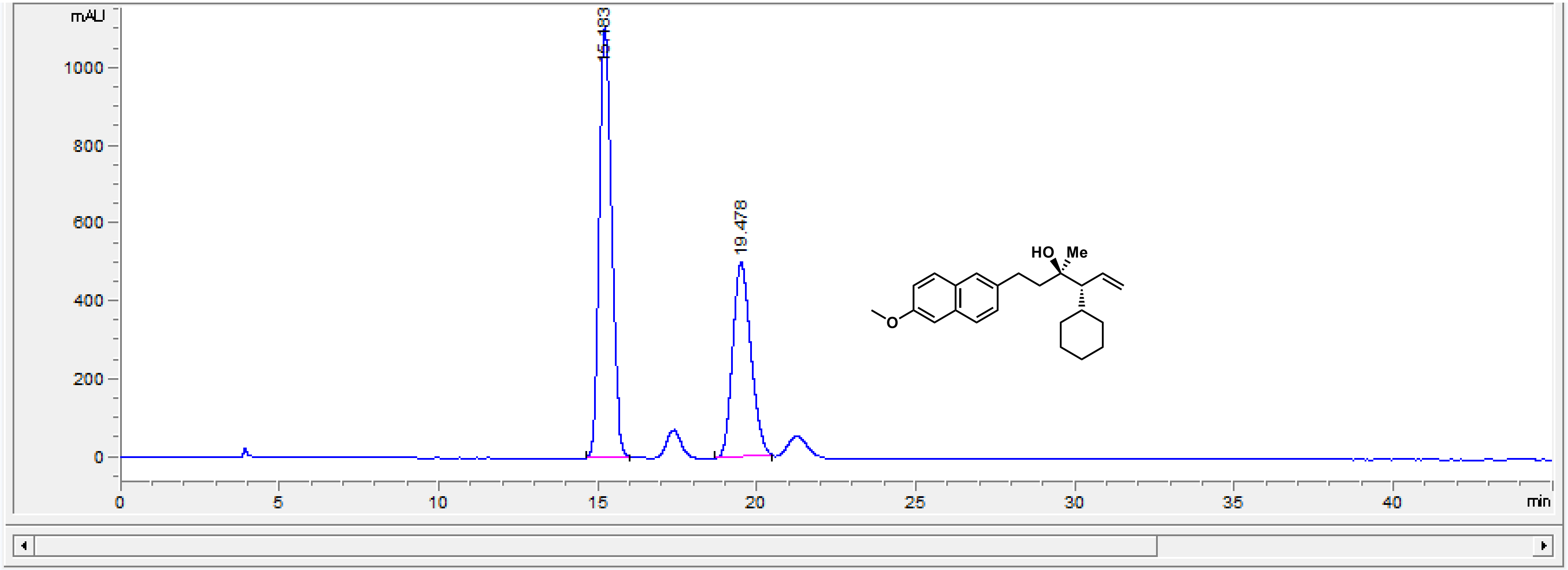

File Information

LC-File	KN08-189A.D
File Path	C:\CHEM32\1\DATA\DC\JOANNE 2022-07-26 09-51-16\

#	Time	Area	Height	Width	Area%	Symmetry
1	15.183	29812.4	1103.6	0.3182	60.278	0.759
2	19.478	19646.1	501.1	0.4589	39.722	0.806

98/2 ODH-(2*R*,3*R*)-4-(2-(7-methoxynaphthalene)-2-ethenylcyclohexyl-butan-2-ol

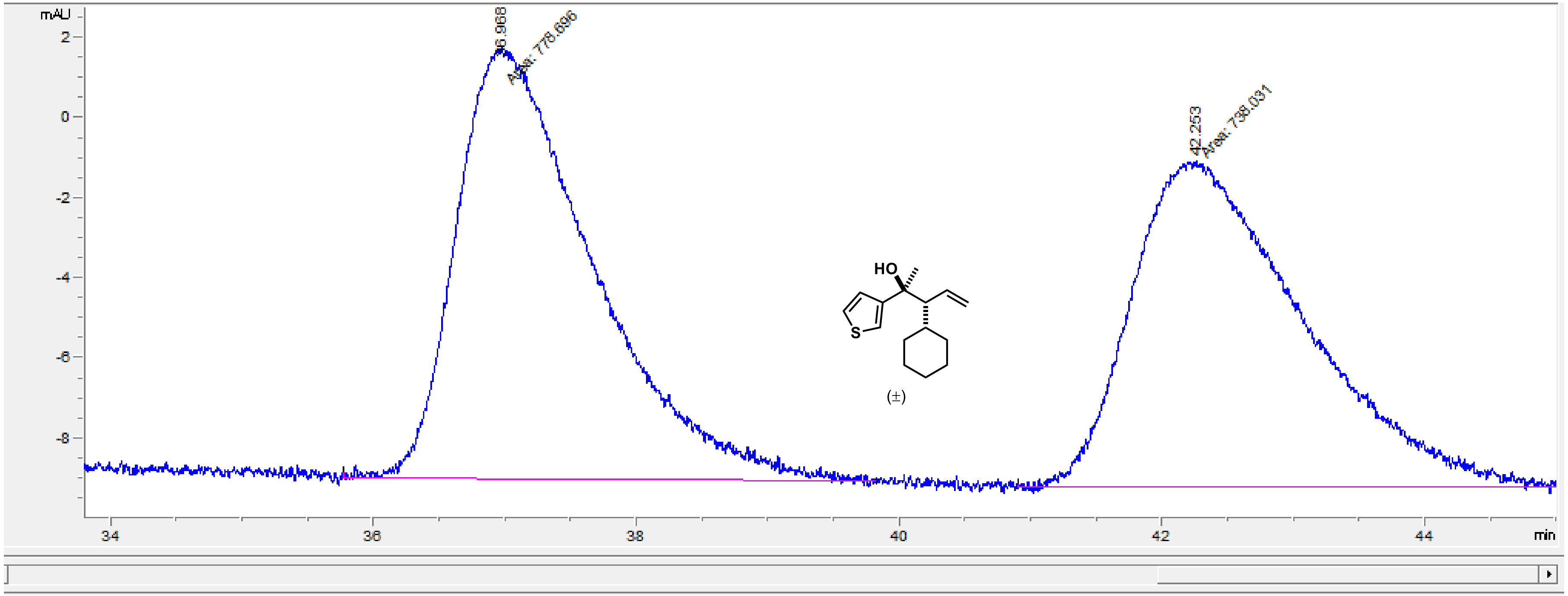

	File Information
LC-File	KN07-106.D
File Path	C:\CHEM32\1\DATA\DC\JOANNE 2022-04-11 11-44-57\
[illegible]	[illegible]

#	Time	Area	Height	Width	Area%	Symmetry
1	36.968	778.7	10.8	1.1992	51.341	0.493
2	42.253	738	8.1	1.5105	48.659	0.59

99/1 ODH-(2*SR*,3*RS*)-2-(Thiophen-2-yl)-3-cyclohexylpent-4-en-2-ol

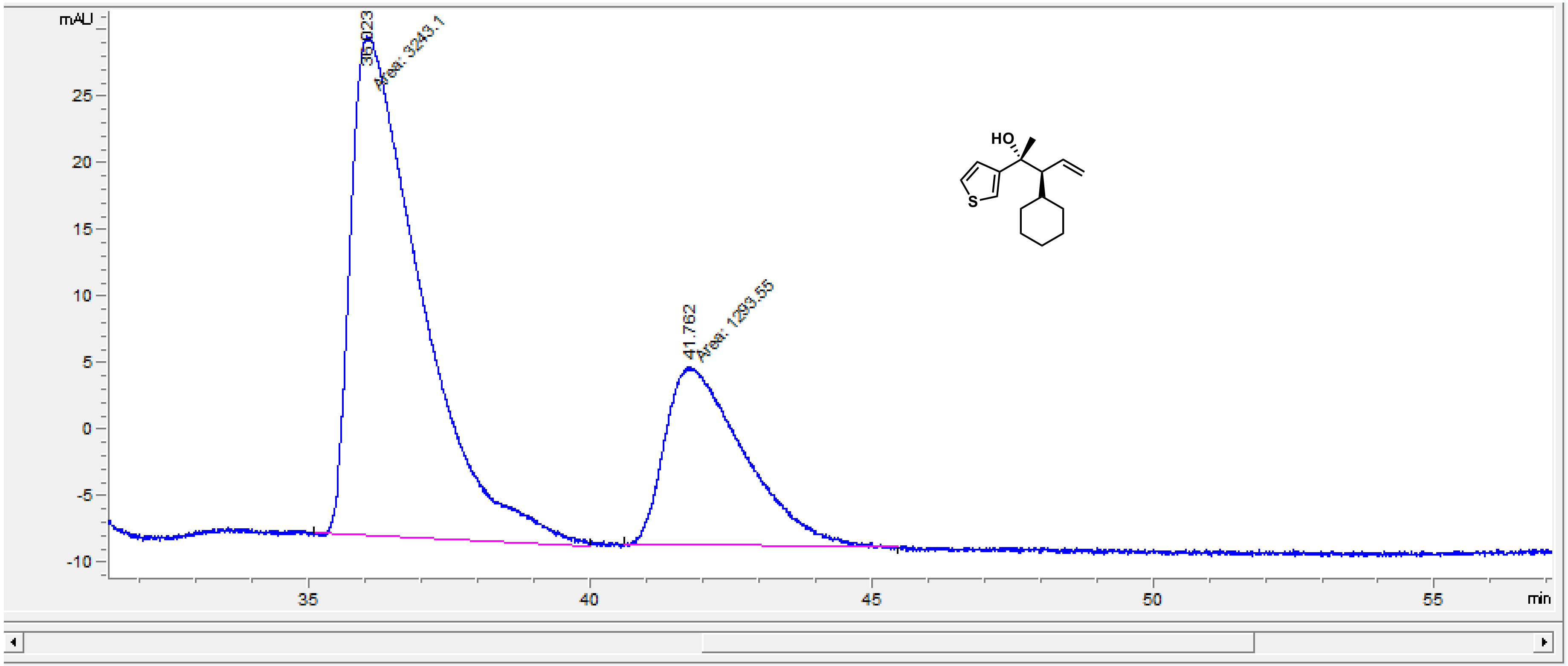

File Information

LC-File	KN08-190.D
File Path	C:\CHEM32\1\DATA\DC\JOANNE 2022-04-13 13-15-26\

#	Time	Area	Height	Width	Area%	Symmetry
1	36.023	3243.1	37.5	1.4428	71.487	0.307
2	41.762	1293.6	13.4	1.6093	28.513	0.459

99/1 ODH-(2*S*,3*R*)-2-(Thiophen-2-yl)-3-cyclohexylpent-4-en-2-ol

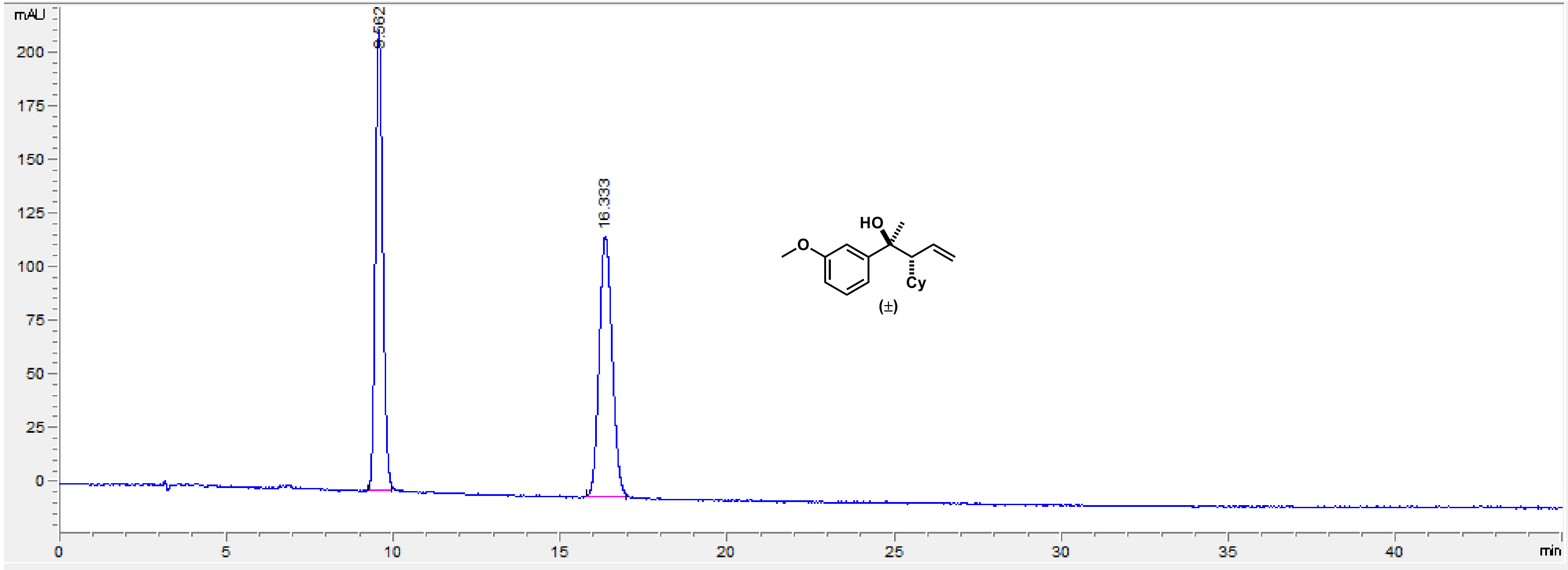

File Information	
LC-File	KN08-192.D
File Path	C:\CHEM32\1\DATA\DC\JOANNE 2022-04-14 13-12-35\

#	Time	Area	Height	Width	Area%	Symmetry
1	9.562	3278.1	214.4	0.1819	50.098	0.85
2	16.333	3265.2	121.8	0.3144	49.902	0.917

95/5 ODH-(2*RS*,3*SR*)-3-Cyclohexyl-2-(3-methoxyphenyl)pent-4-en-2-ol

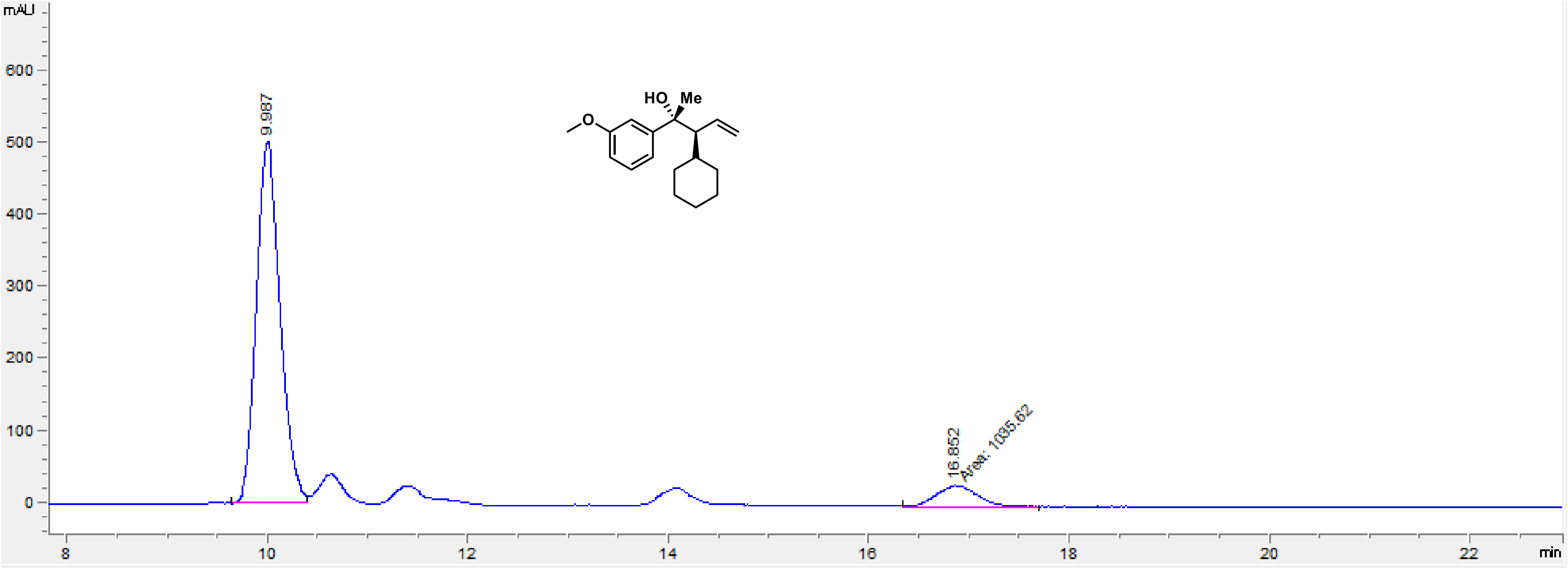

File Information	
LC-File	KN09-001.D
File Path	C:\CHEM32\1\DATA\DC\JOANNE 2022-04-19 11-06-25\

#	Time	Area	Height	Width	Area%	Symmetry
1	9.987	8350.6	503.8	0.2054	88.967	0.766
2	16.852	1035.6	31.9	0.5419	11.033	0.794

95/5 ODH-(2*R*,3*S*)-3-Cyclohexyl-2-(3-methoxyphenyl)pent-4-en-2-ol

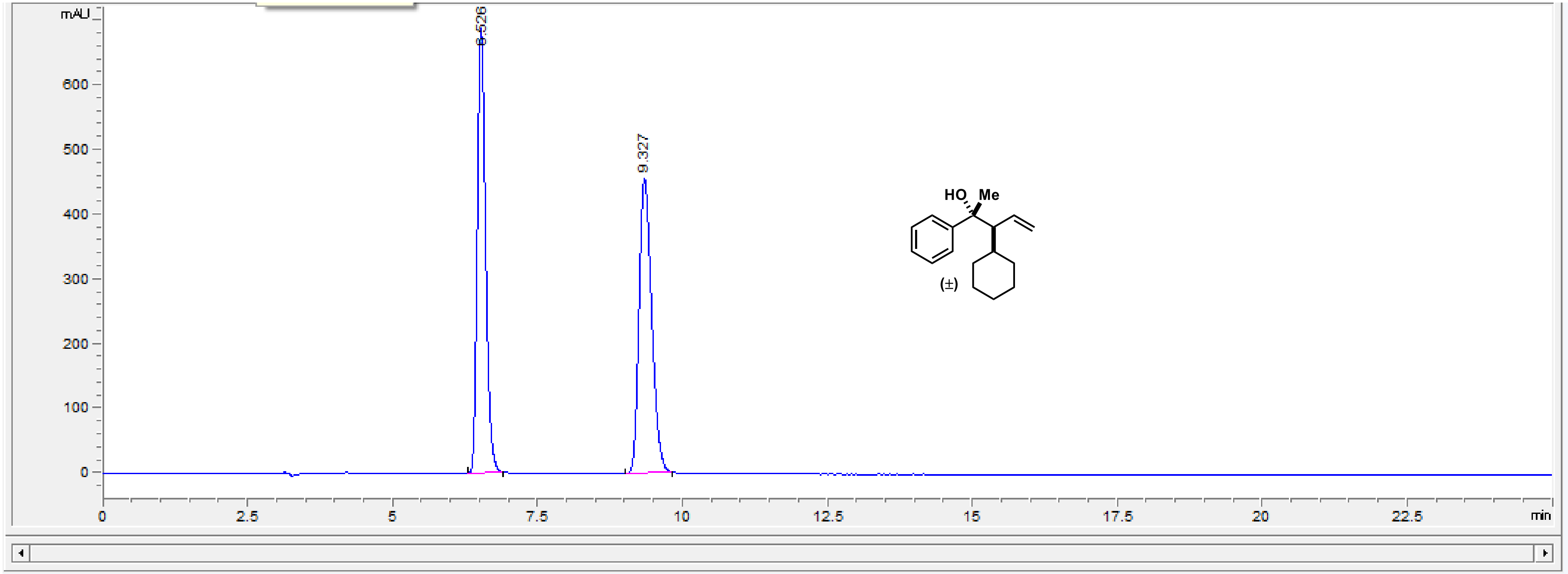

File Information	
LC-File	KN08-076-RAC.D
File Path	C:\CHEM32\1\DATA\DC\JOANNE 2022-03-03 15-16-21\

#	Time	Area	Height	Width	Area%	Symmetry
1	6.526	6737.8	688	0.1503	49.702	0.783
2	9.327	6818.5	456.6	0.1997	50.298	0.698

95/5 ODH-(2*RS*,3*SR*)-3-cyclohexyl-2-phenylpent-4-en-2-ol

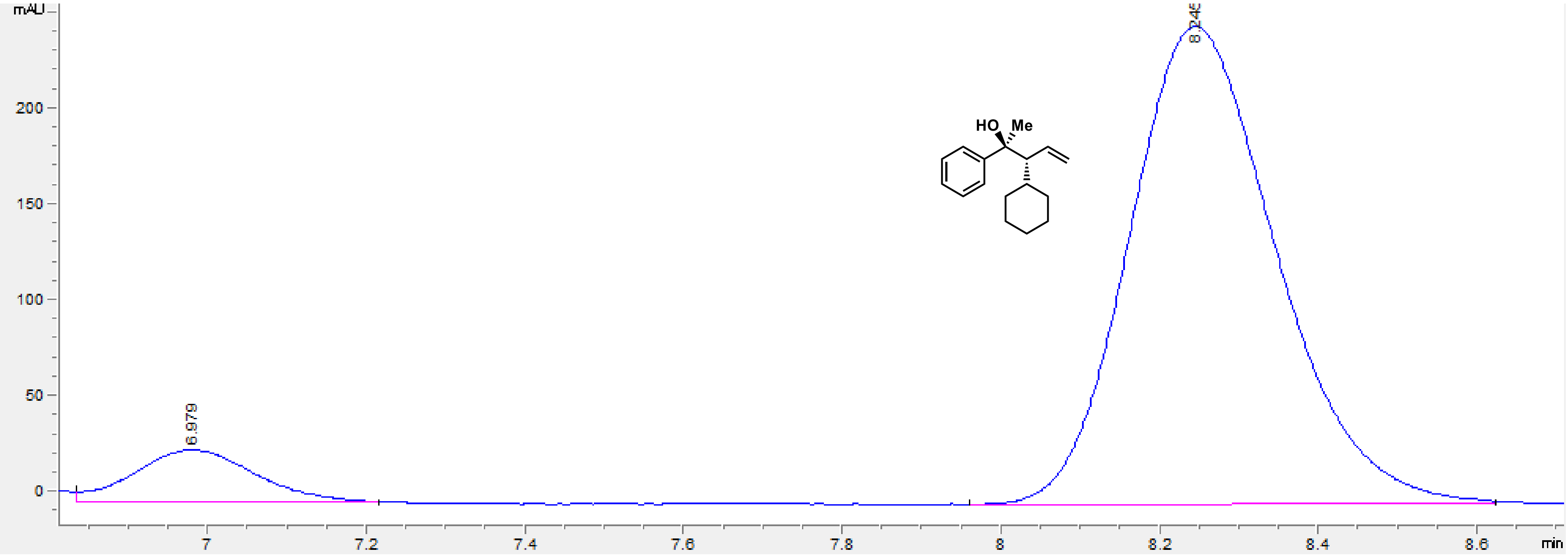

	File Information
LC-File	KN08-179.D
File Path	C:\CHEM32\1\DATA\DC\JOANNE 2022-04-19 09-03-08\
[illegible]	[illegible]

#	Time	Area	Height	Width	Area%	Symmetry
1	6.979	297	27.5	0.1297	8.463	0.875
2	8.245	3212.8	249.5	0.1837	91.537	0.795

(2*S*,3*R*)-3-cyclohexyl-2-phenylpent-4-en-2-ol

S10 Determination of reaction diastereoselectivity

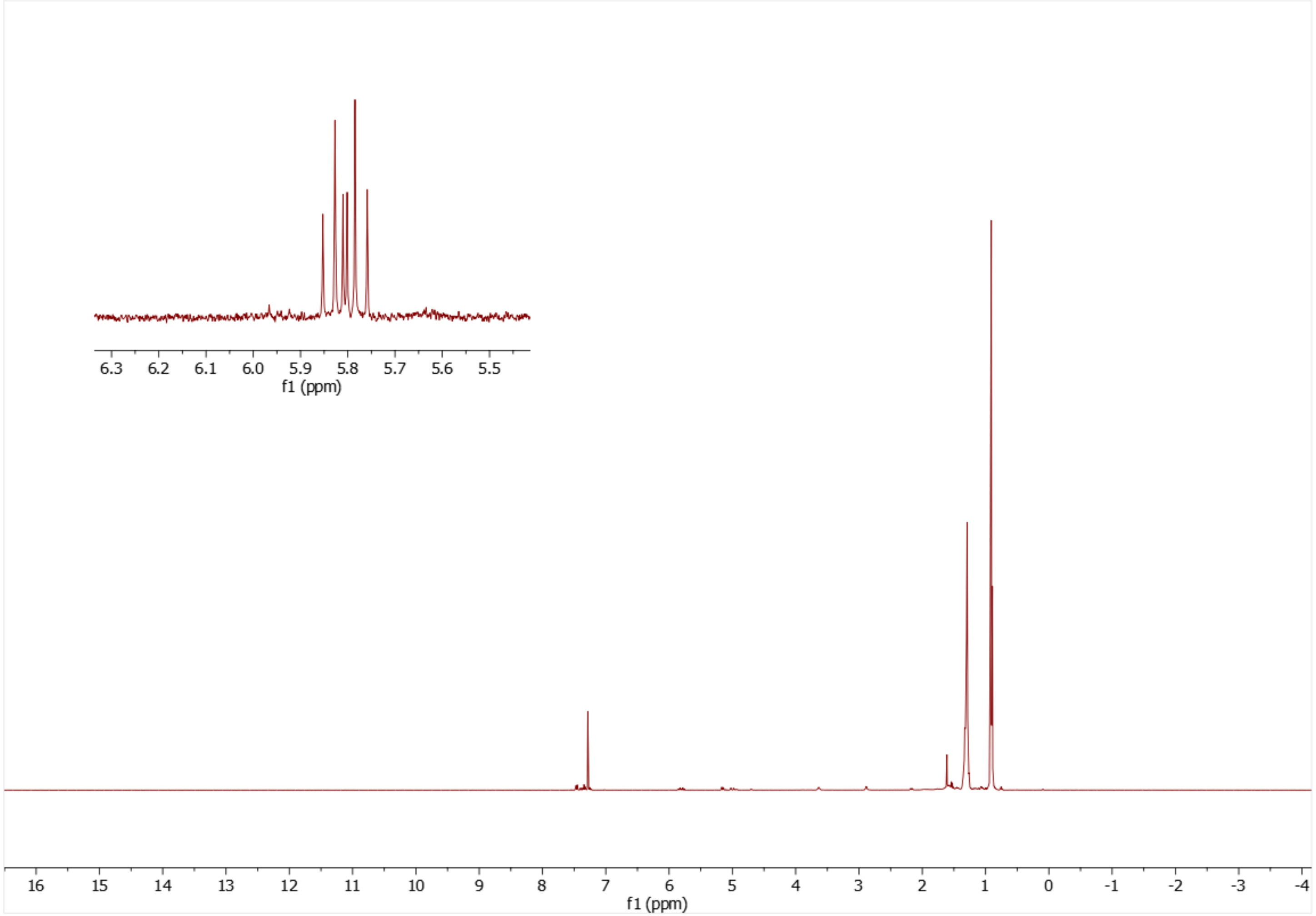

^{1}H NMR (400 MHz, $CDCl_3$) Spectrum of the crude reaction mixture for **3a** >95:5 *d.r.*

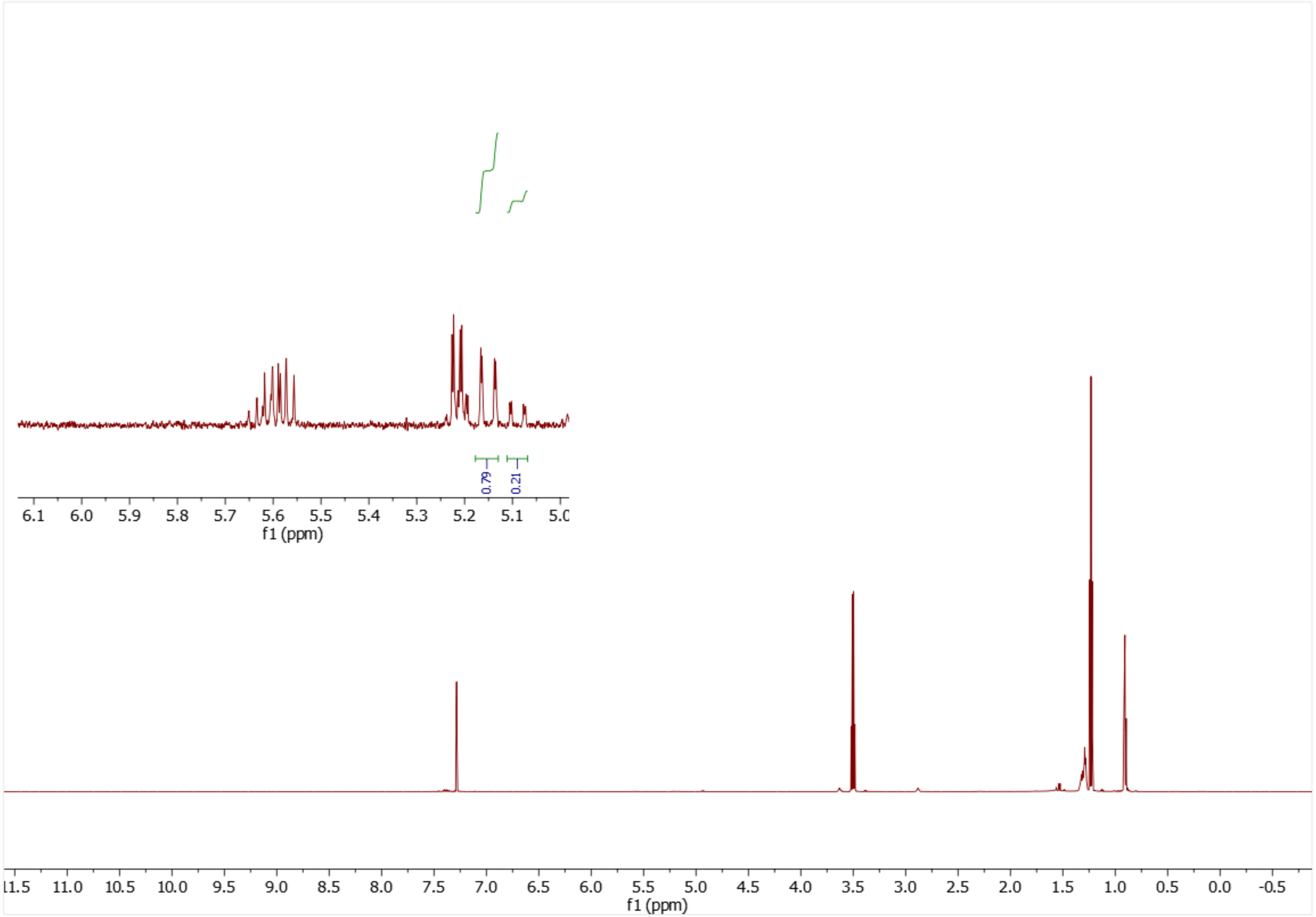

^{1}H NMR (400 MHz, $CDCl_3$) Spectrum of the crude reaction mixture for **3c** 79:21 *d.r.*

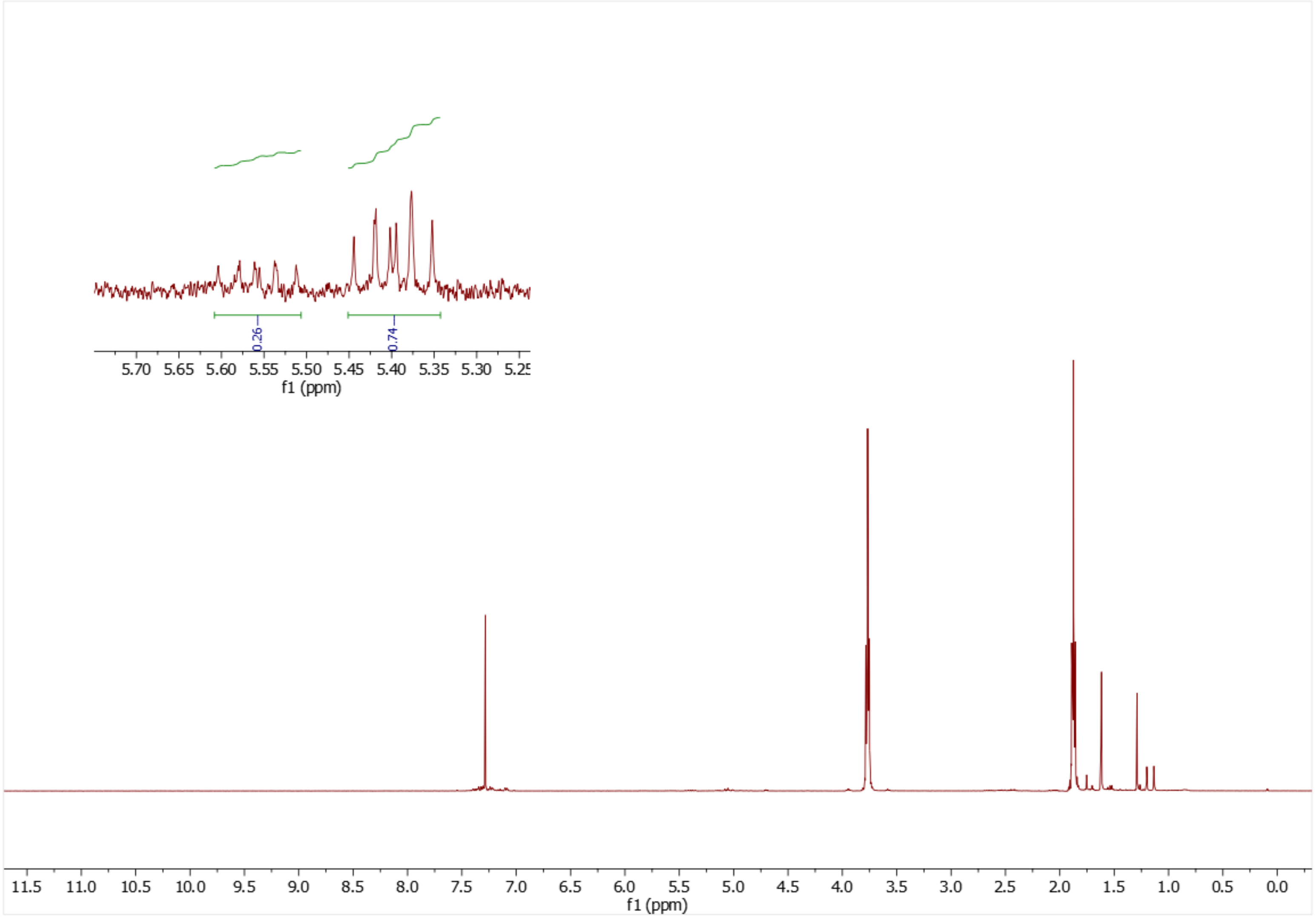

^{1}H NMR (400 MHz, $CDCl_3$) Spectrum of the crude reaction mixture for **3d** 74:26 *d.r.*

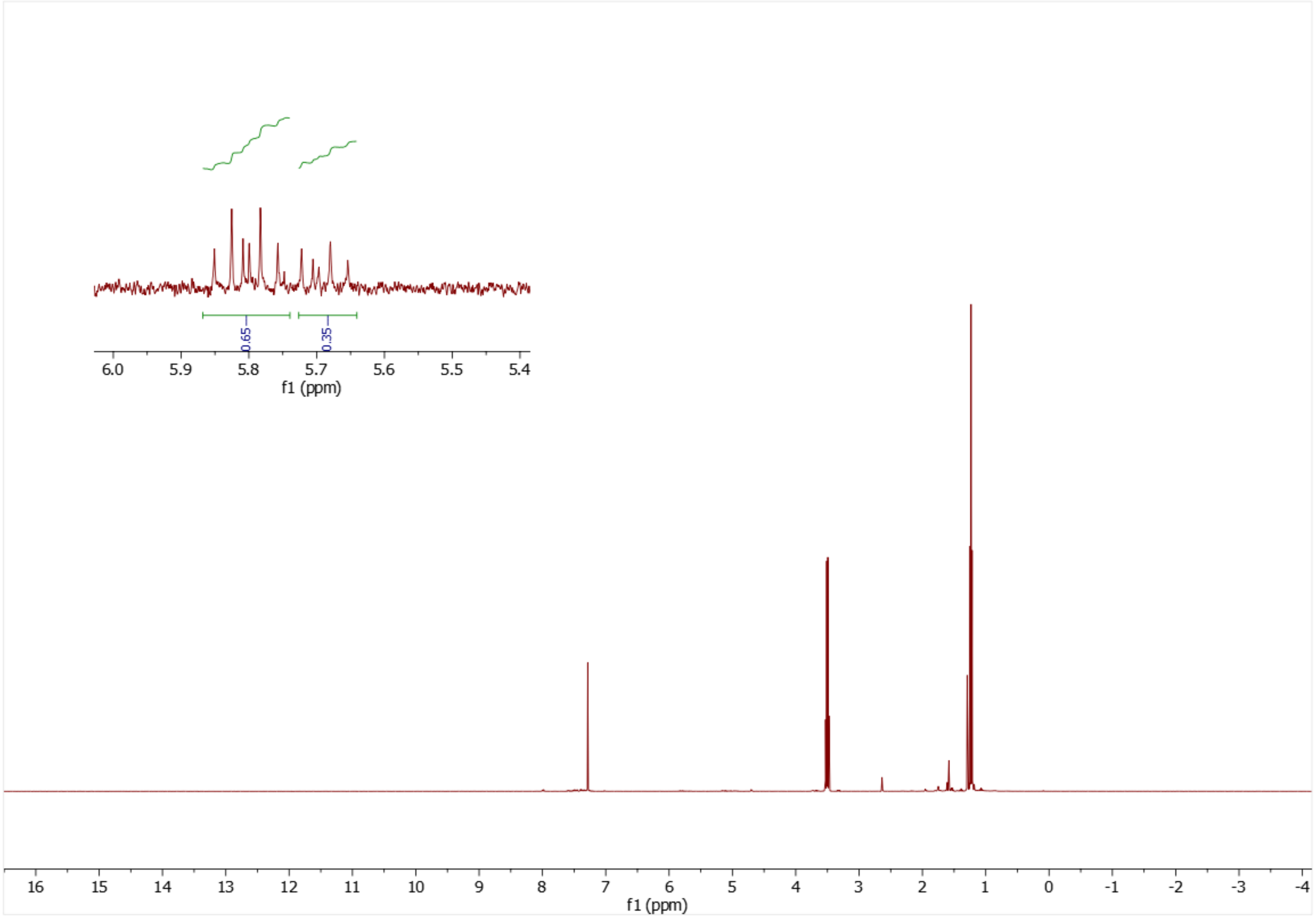

^{1}**H NMR** (400 MHz, $CDCl_3$) Spectrum of the crude reaction mixture for **3e** 65:35 *d.r.*

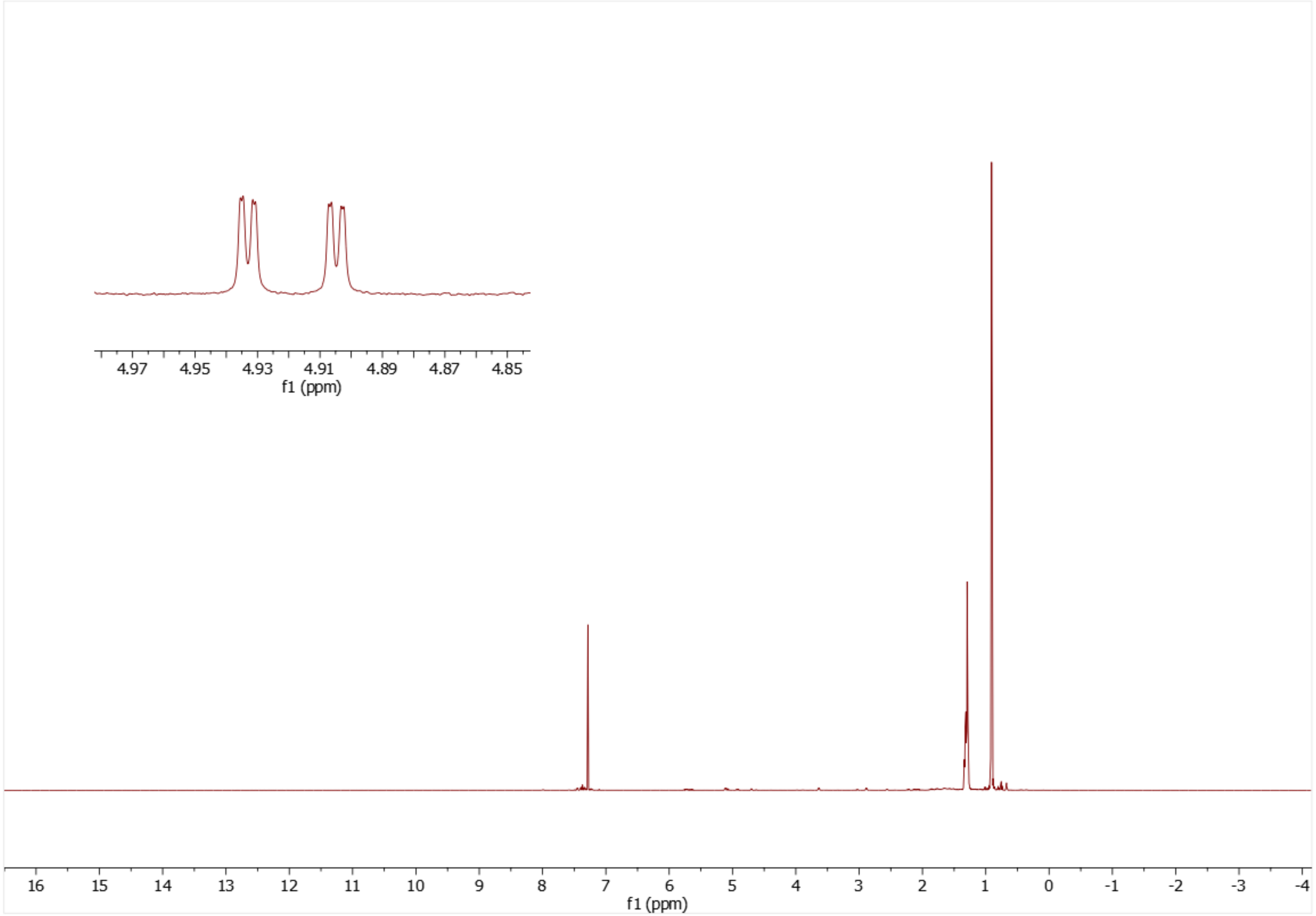

^{1}H NMR (400 MHz, $CDCl_3$) Spectrum of the crude reaction mixture for **3i** >95:5 *d.r.*

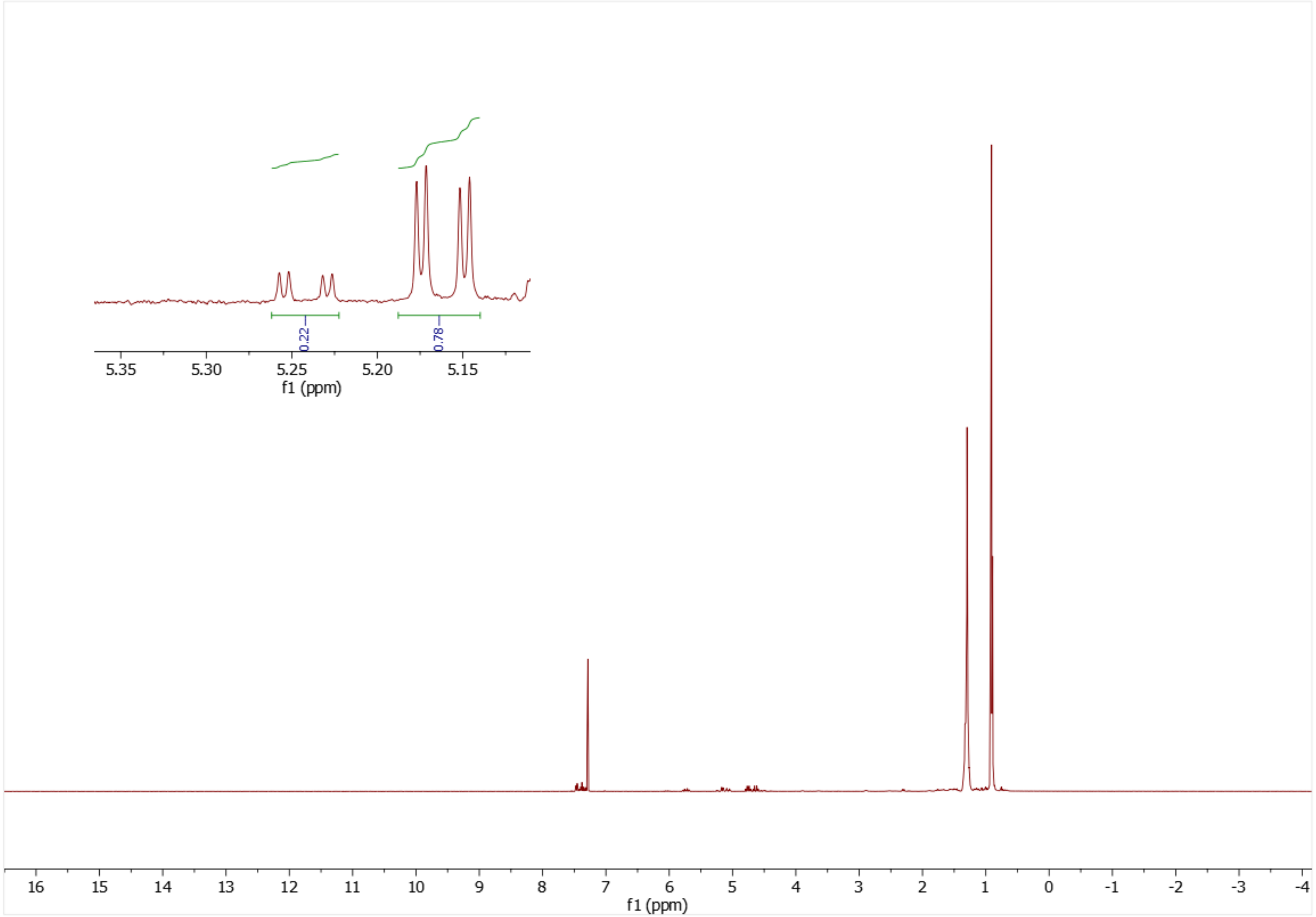

[1]**H NMR** (400 MHz, $CDCl_3$) Spectrum of the crude reaction mixture for **3j** 78:22 *d.r.*

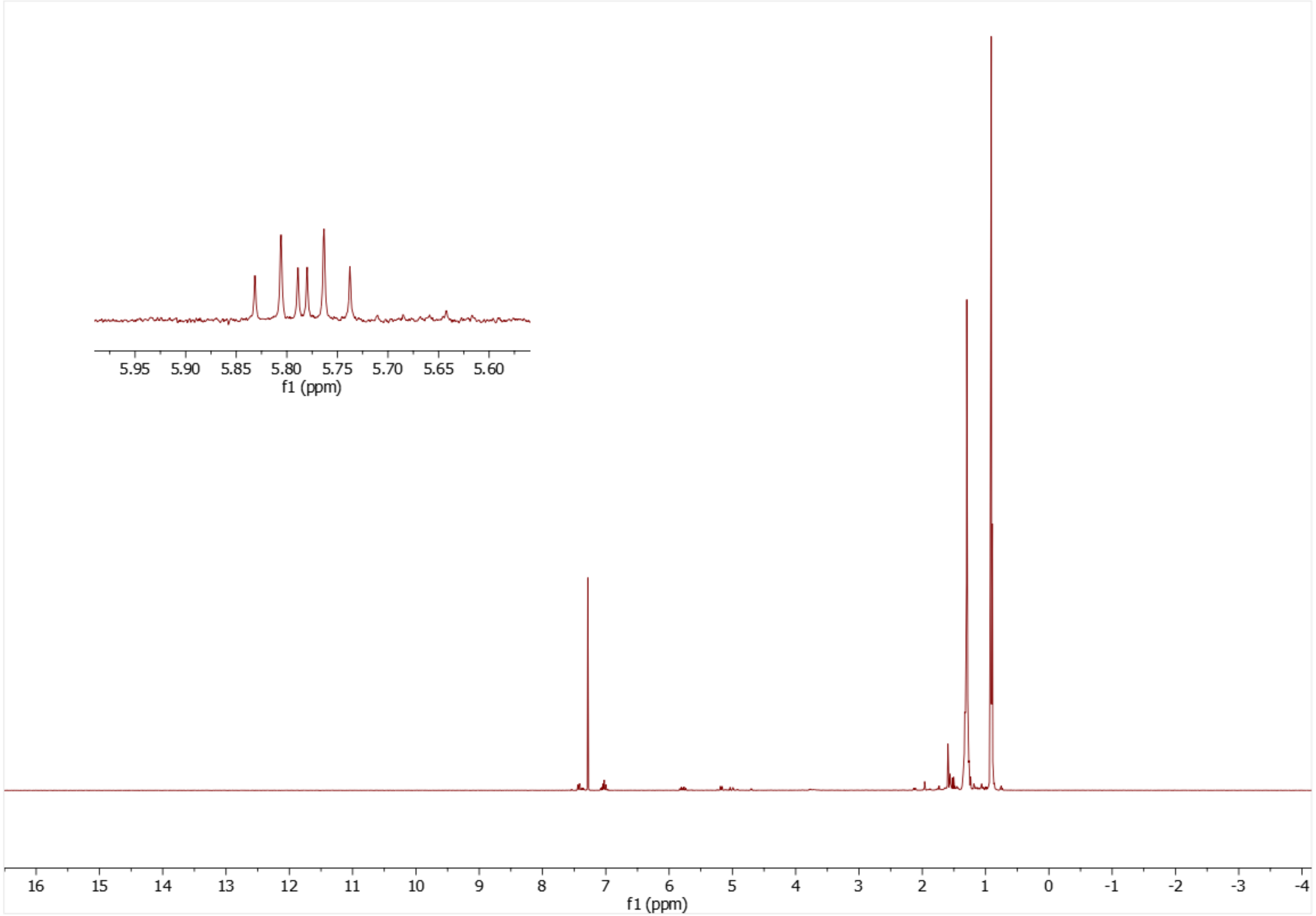

^{1}H NMR (400 MHz, $CDCl_3$) Spectrum of the crude reaction mixture for **3l** >95:5 *d.r.*

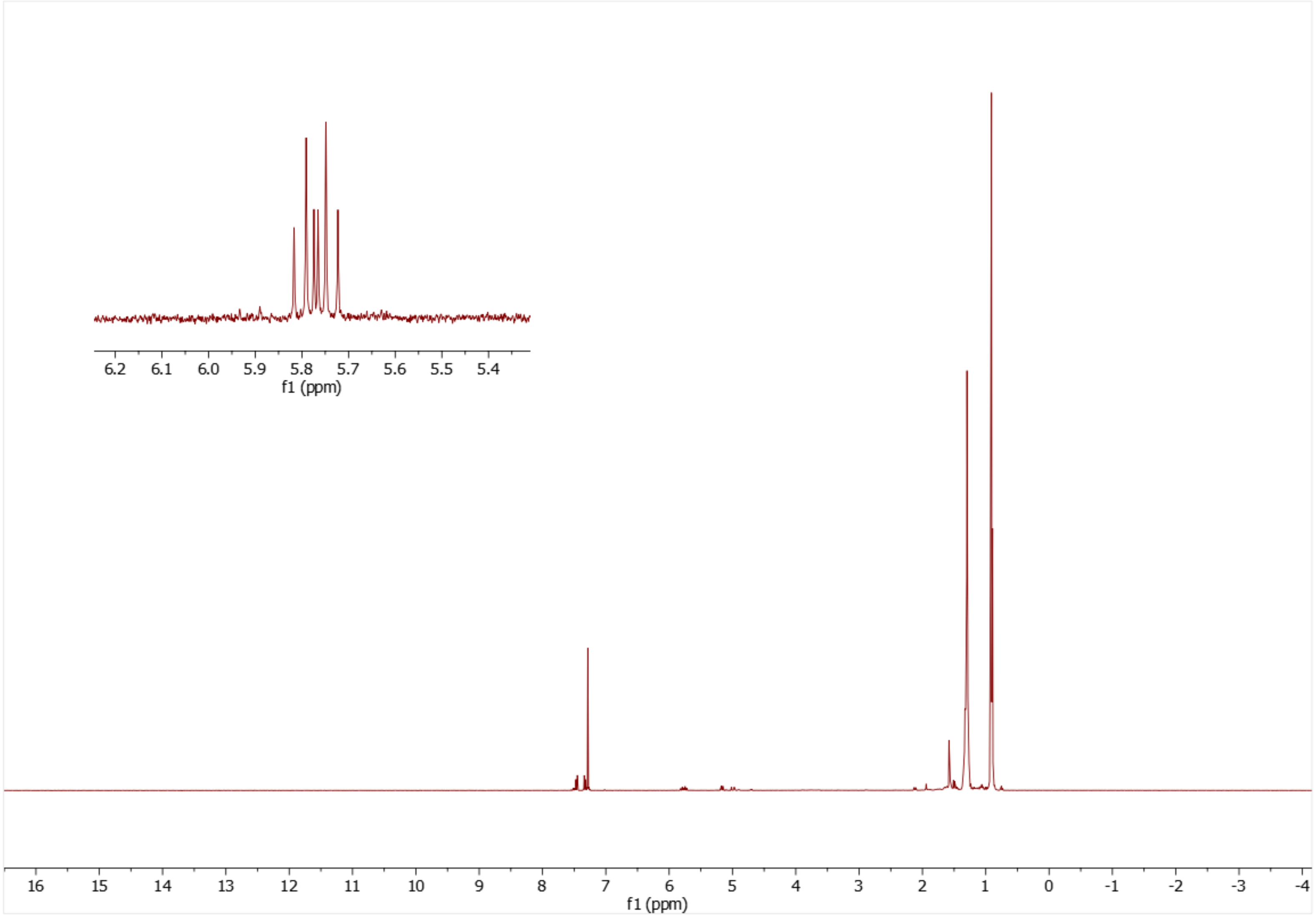

^{1}H NMR (400 MHz, $CDCl_3$) Spectrum of the crude reaction mixture for **3m** >95:5 *d.r.*

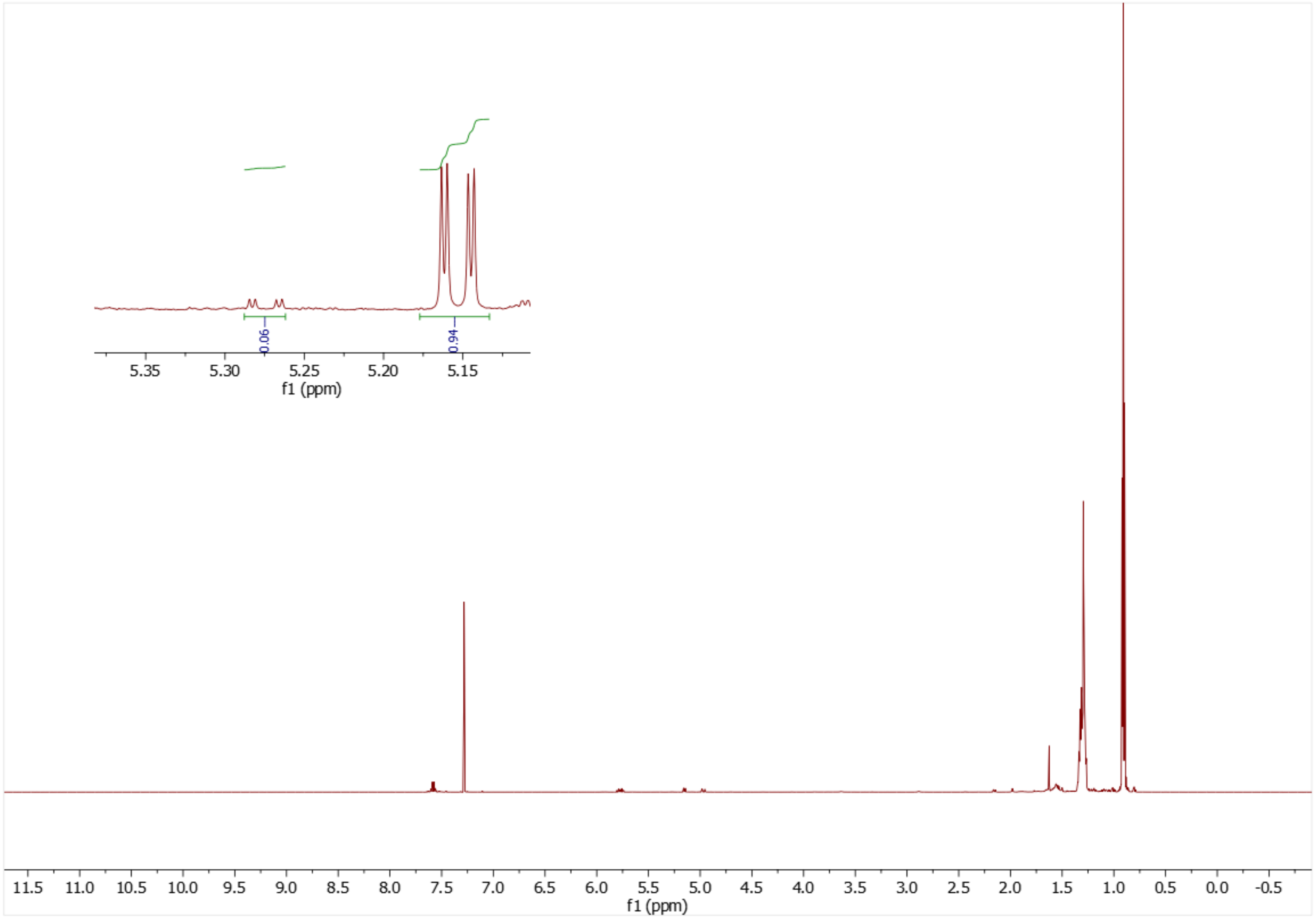

^{1}H NMR (400 MHz, $CDCl_3$) Spectrum of the crude reaction mixture for **3n** 94:6 *d.r.*

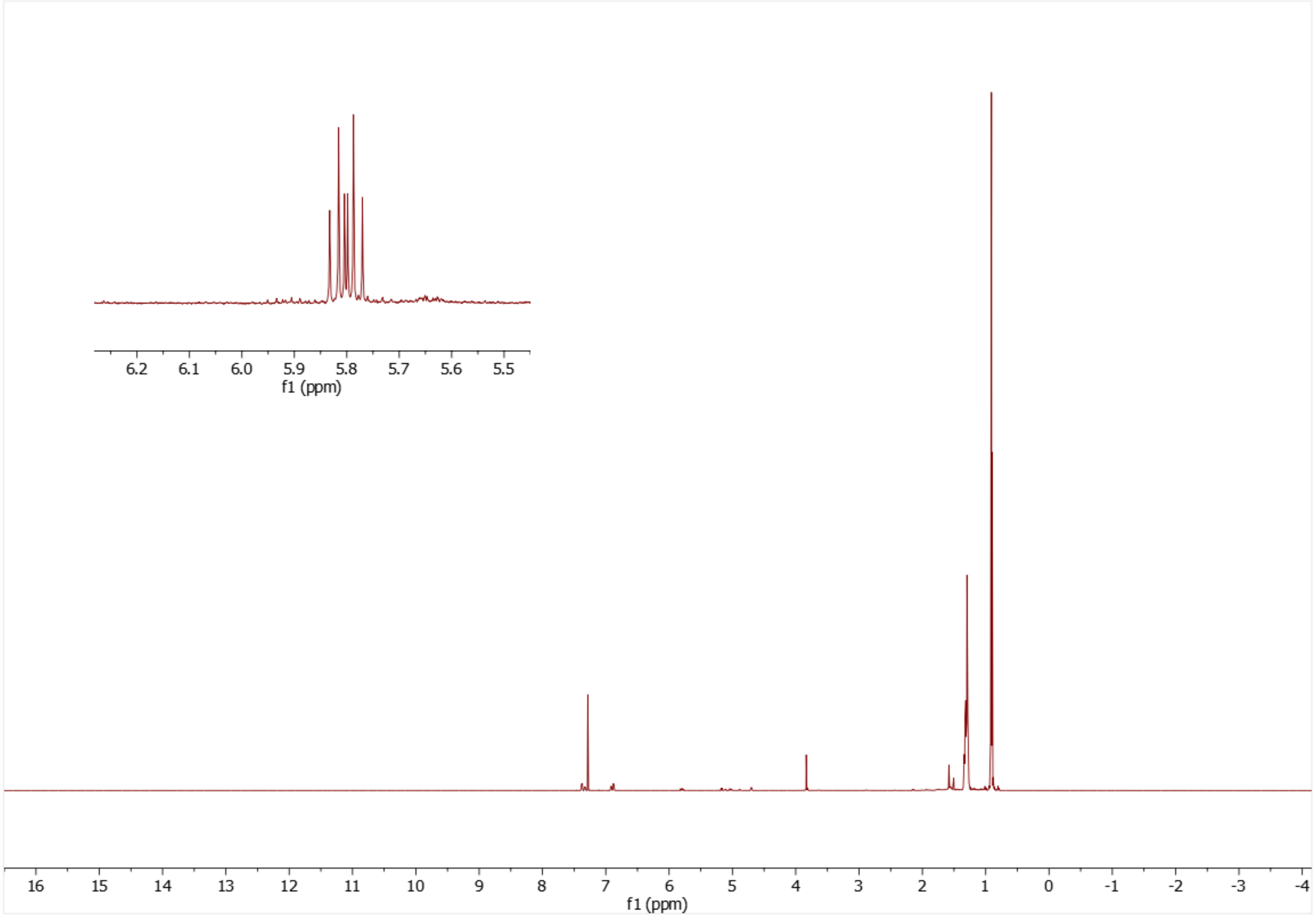

^{1}H NMR (400 MHz, $CDCl_3$) Spectrum of the crude reaction mixture for **3o** >95:5 *d.r.*

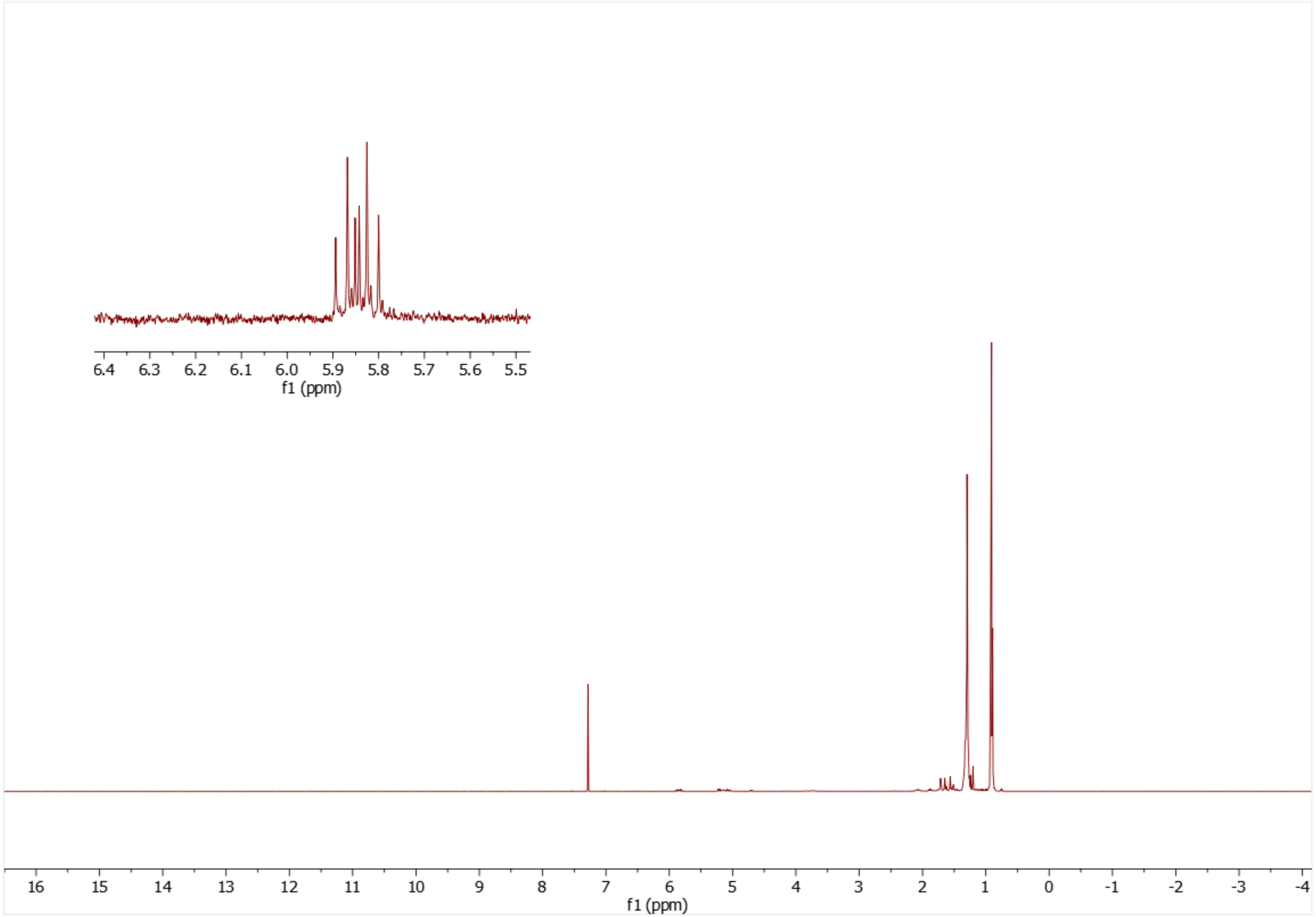

^{1}H NMR (400 MHz, $CDCl_3$) Spectrum of the crude reaction mixture for **3q** >95:5 *d.r.*

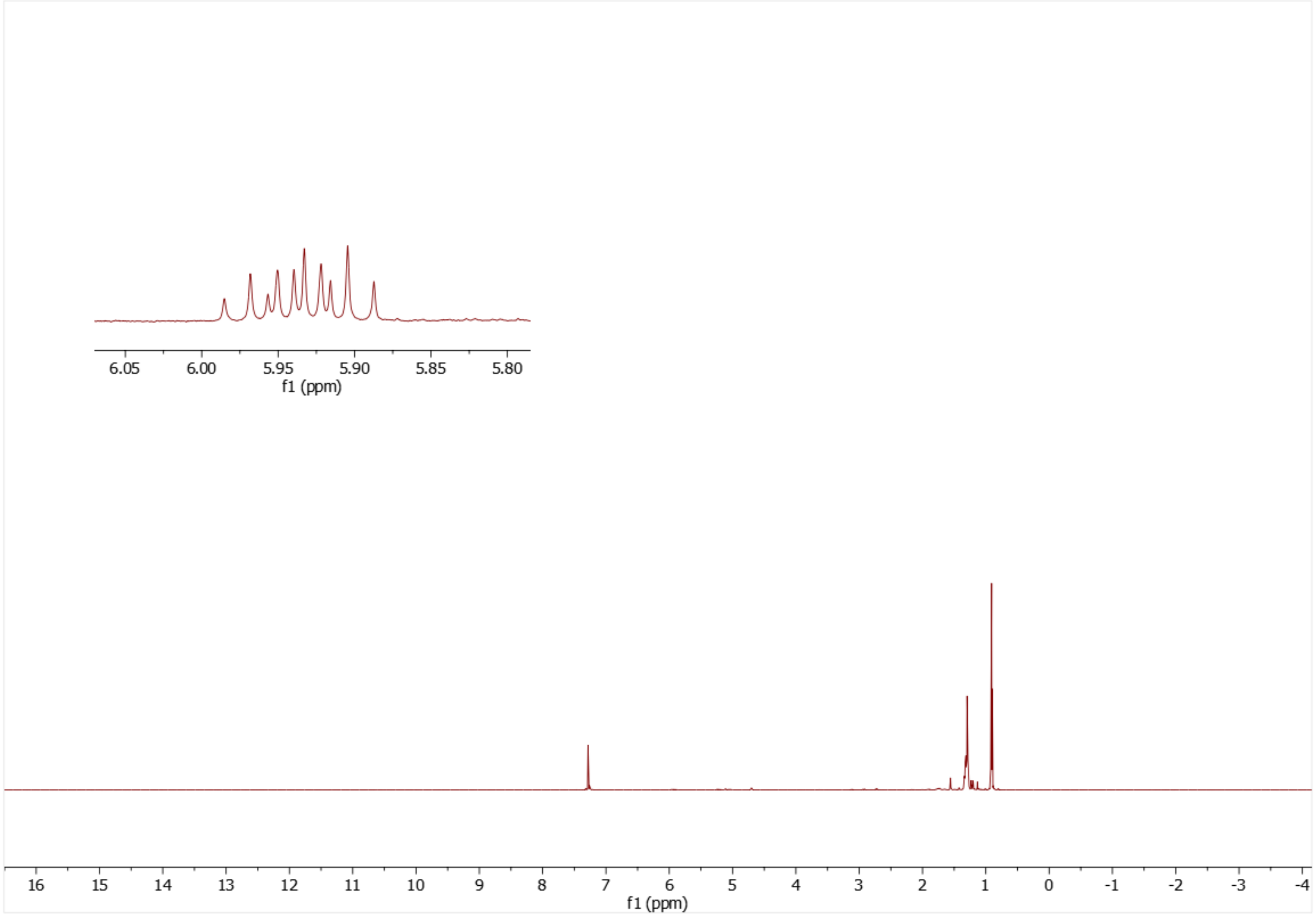

^{1}H NMR (400 MHz, $CDCl_3$) Spectrum of the crude reaction mixture for **3s** >95:5 *d.r.*

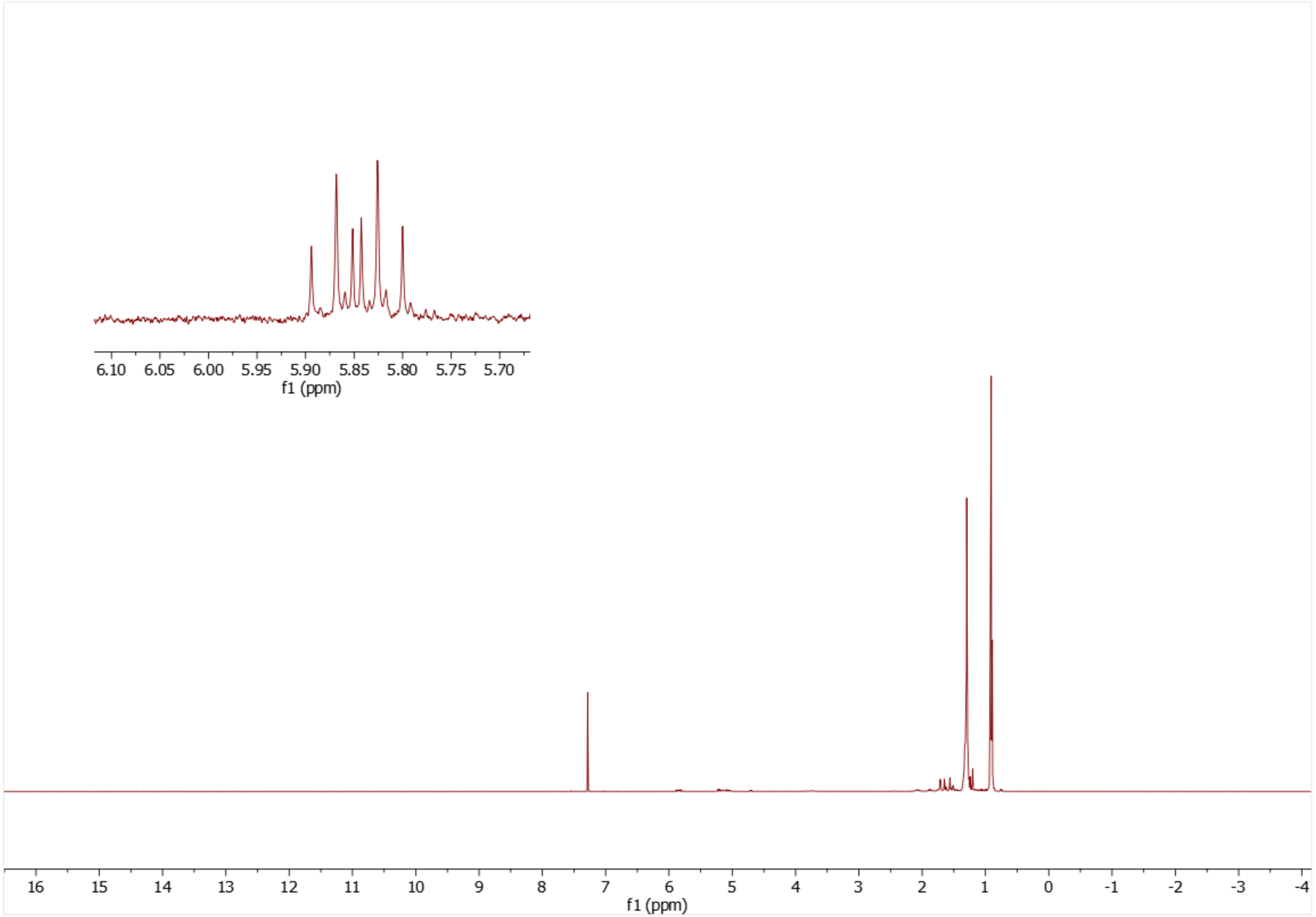

^{1}H NMR (400 MHz, $CDCl_3$) Spectrum of the crude reaction mixture for **3t** >95:5 *d.r.*

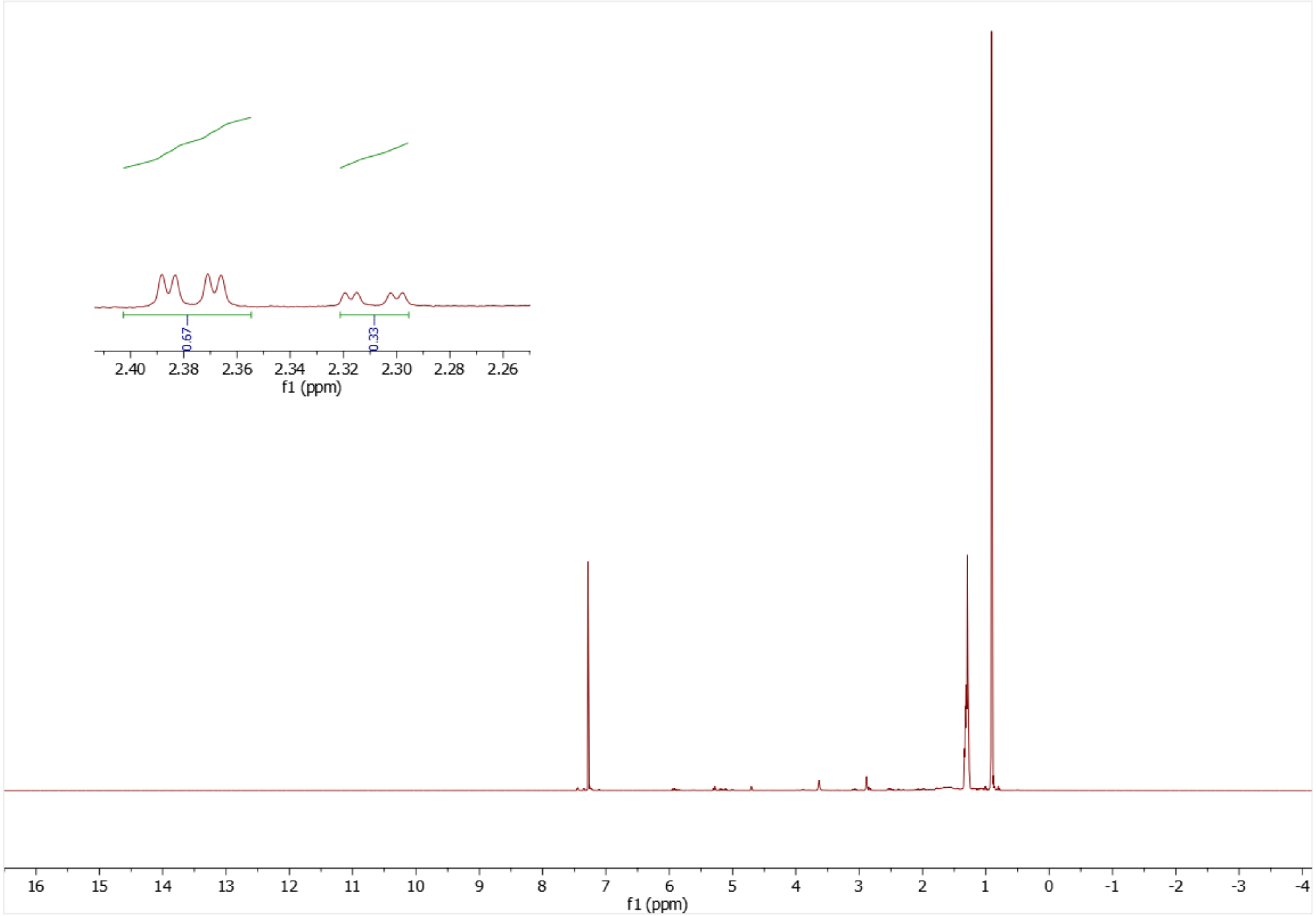

^{1}**H NMR** (400 MHz, $CDCl_3$) Spectrum of the crude reaction mixture for **3u** 67:33 *d.r.*

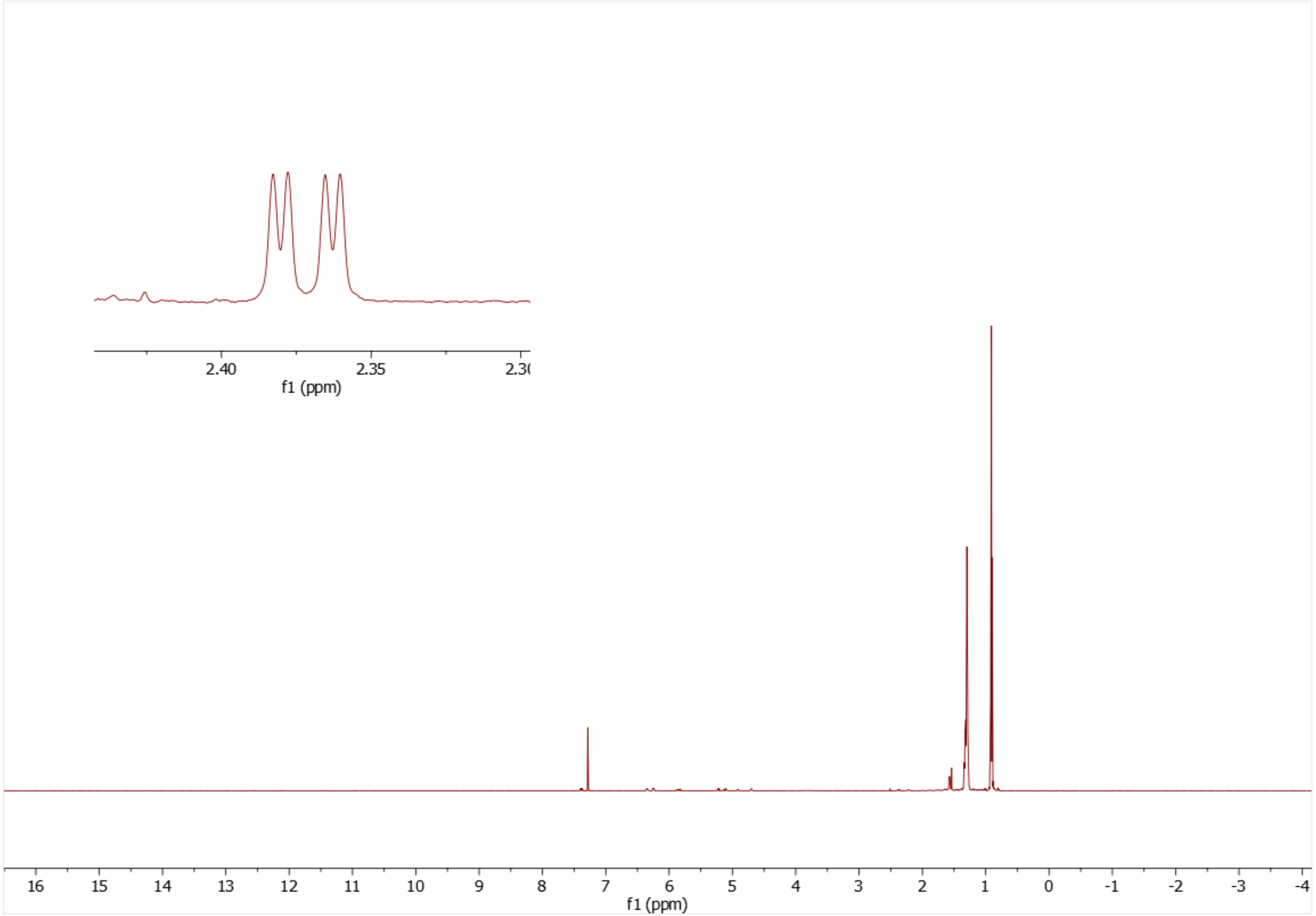

^{1}H NMR (400 MHz, $CDCl_3$) Spectrum of the crude reaction mixture for **3v** >95:5 *d.r*.

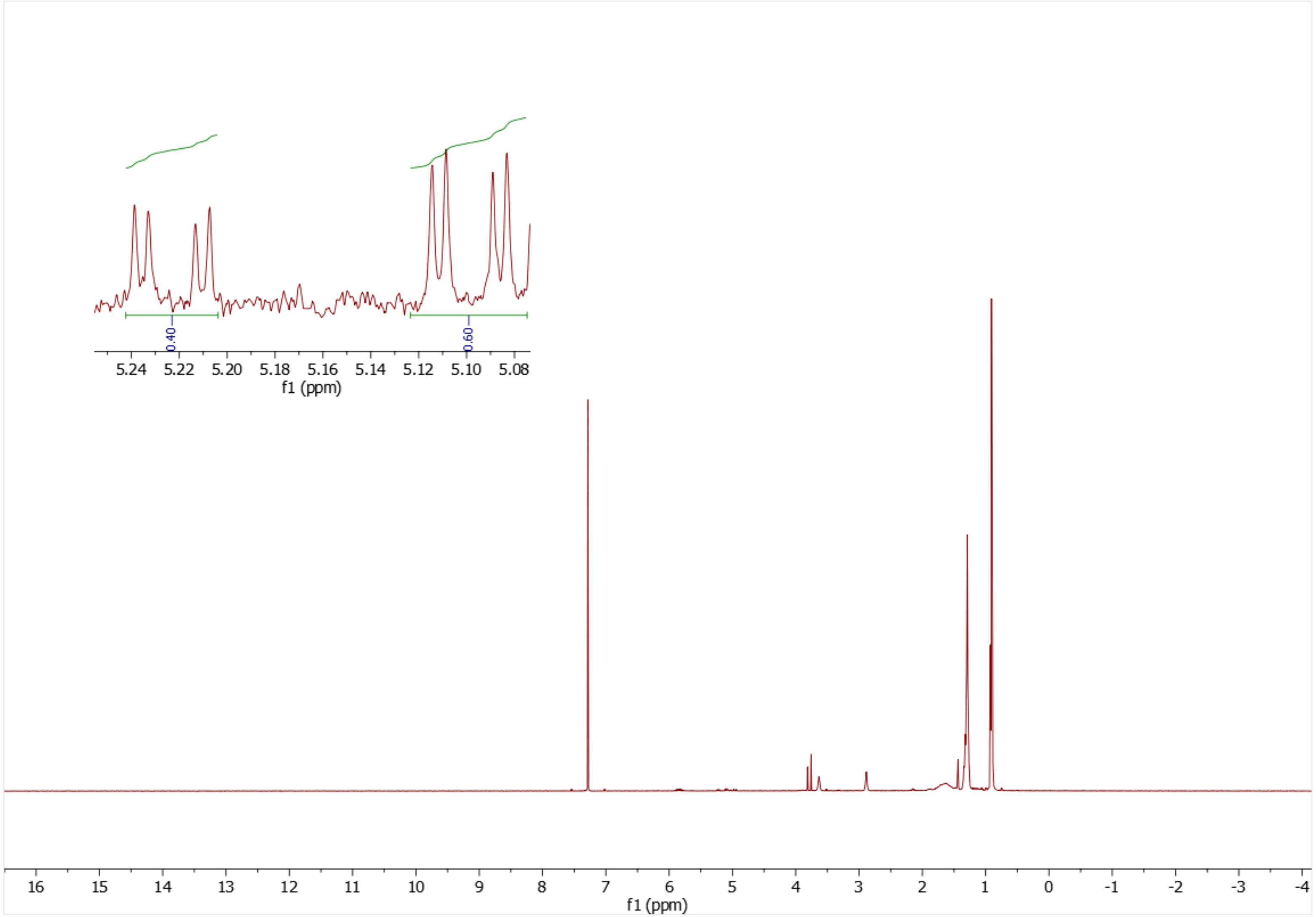

[1]**H NMR** (400 MHz, $CDCl_3$) Spectrum of the crude reaction mixture for **3w** >95:5 *d.r.*

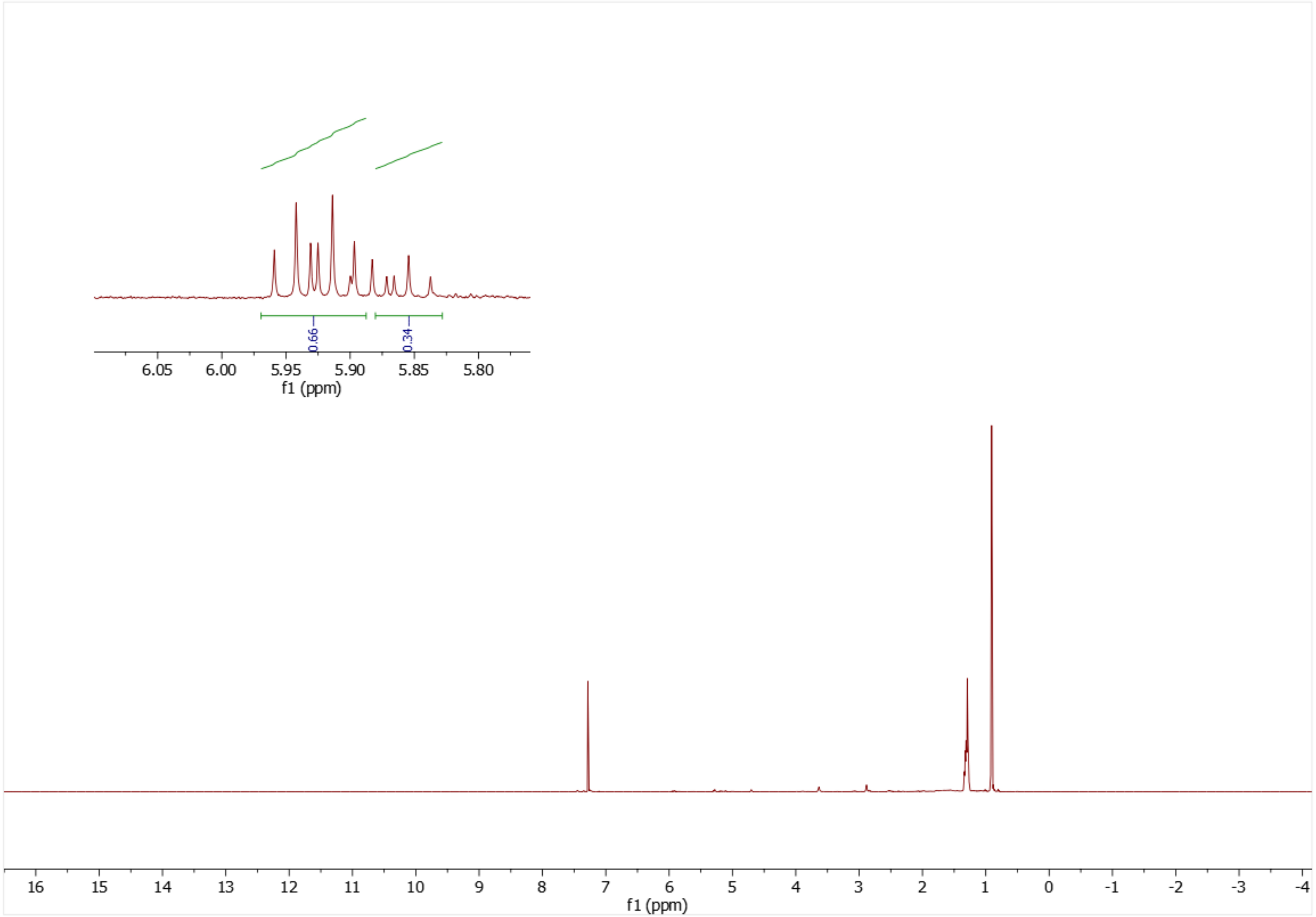

^{1}H NMR (400 MHz, $CDCl_3$) Spectrum of the crude reaction mixture for **3x** 66:34 *d.r.*

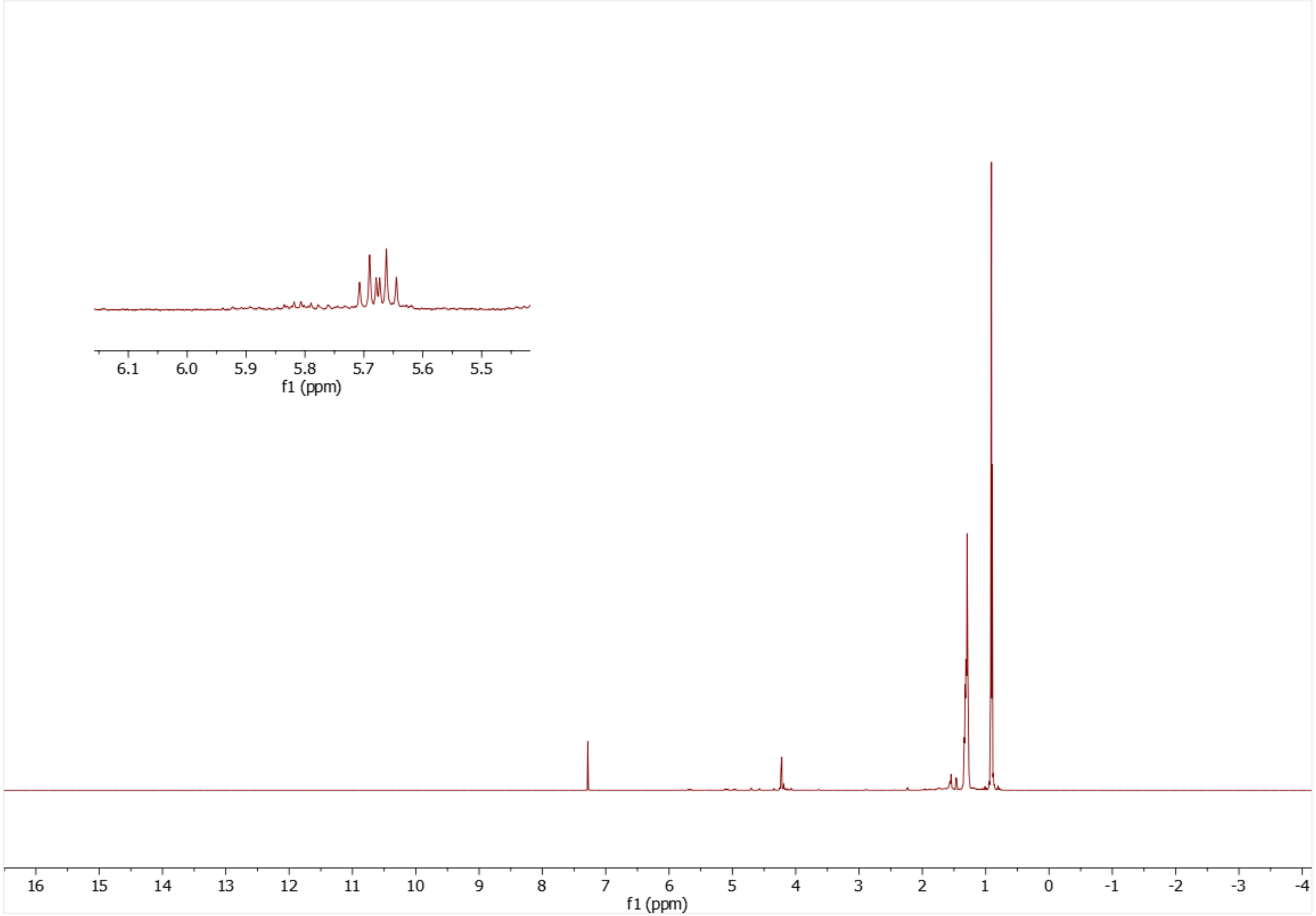

¹H NMR (400 MHz, $CDCl_3$) Spectrum of the crude reaction mixture for **3y** >95:5 *d.r.*

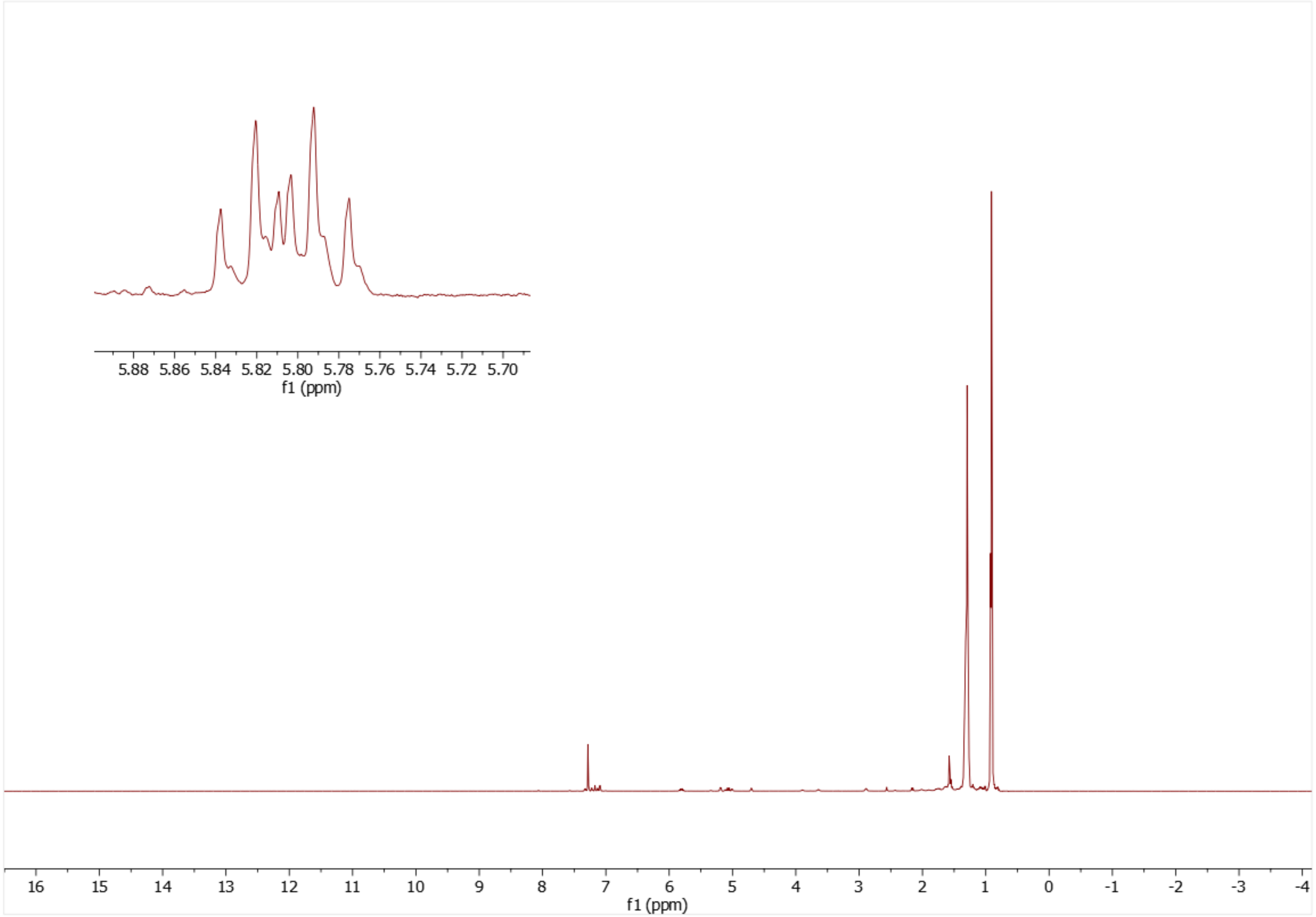

[1]**H NMR** (400 MHz, $CDCl_3$) Spectrum of the crude reaction mixture for **3z** >95:5 *d.r.*

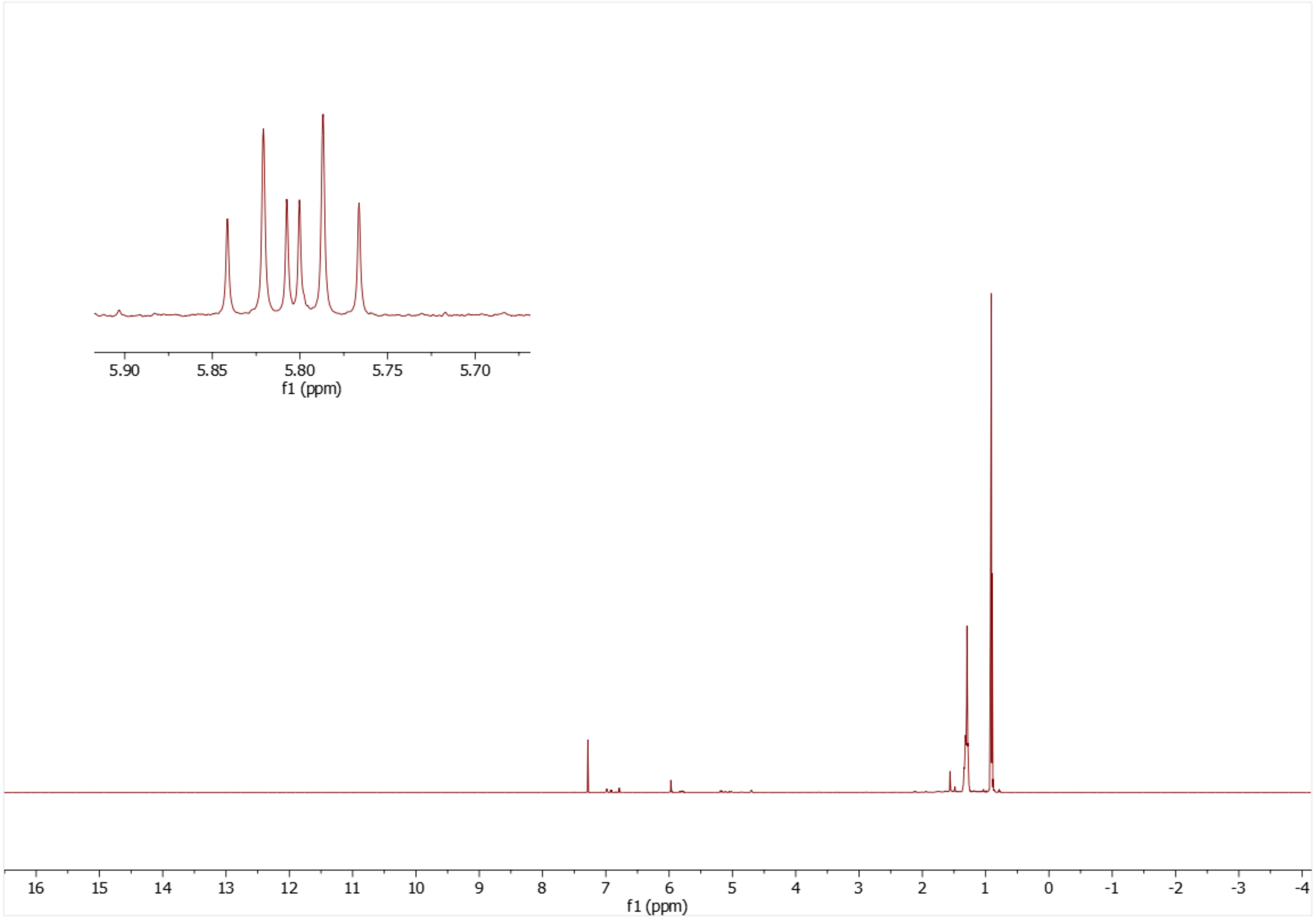

^{1}H NMR (400 MHz, $CDCl_3$) Spectrum of the crude reaction mixture for **3aa** >95:5 *d.r.*

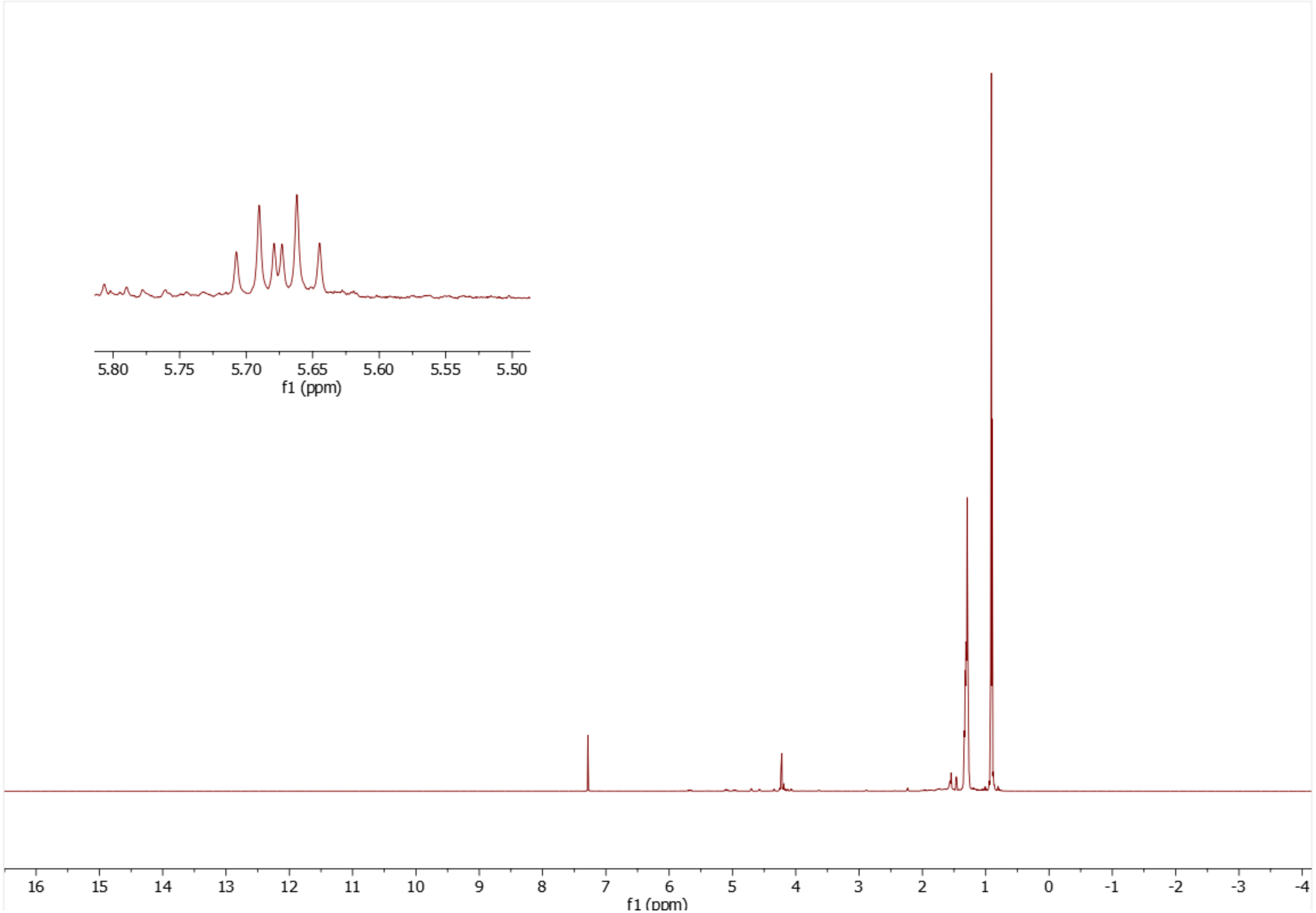

^{1}**H NMR** (400 MHz, $CDCl_3$) Spectrum of the crude reaction mixture for **3ab** >95:5 *d.r*.

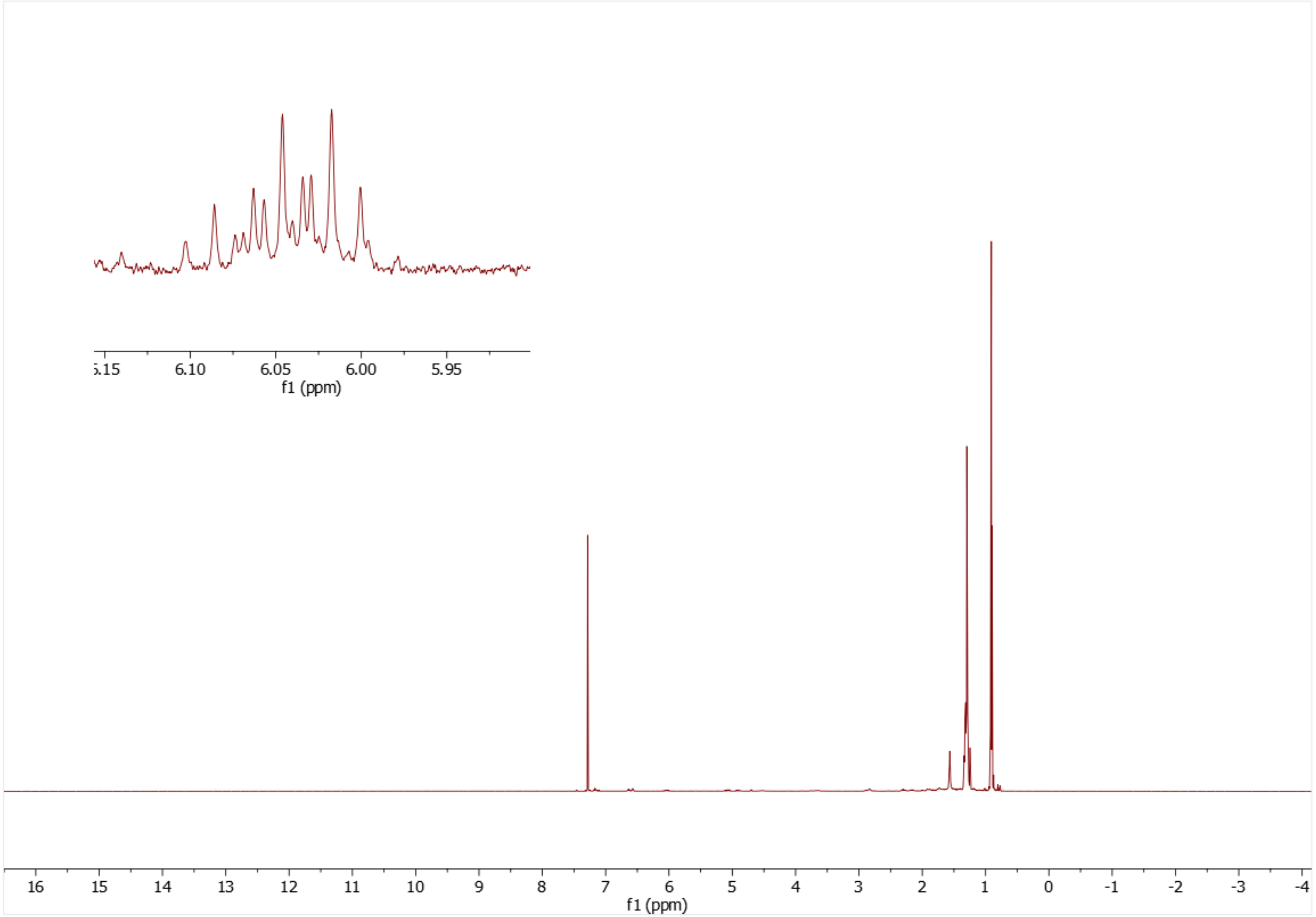

^{1}H NMR (400 MHz, $CDCl_3$) Spectrum of the crude reaction mixture for **3ac** >95:5 *d.r.*

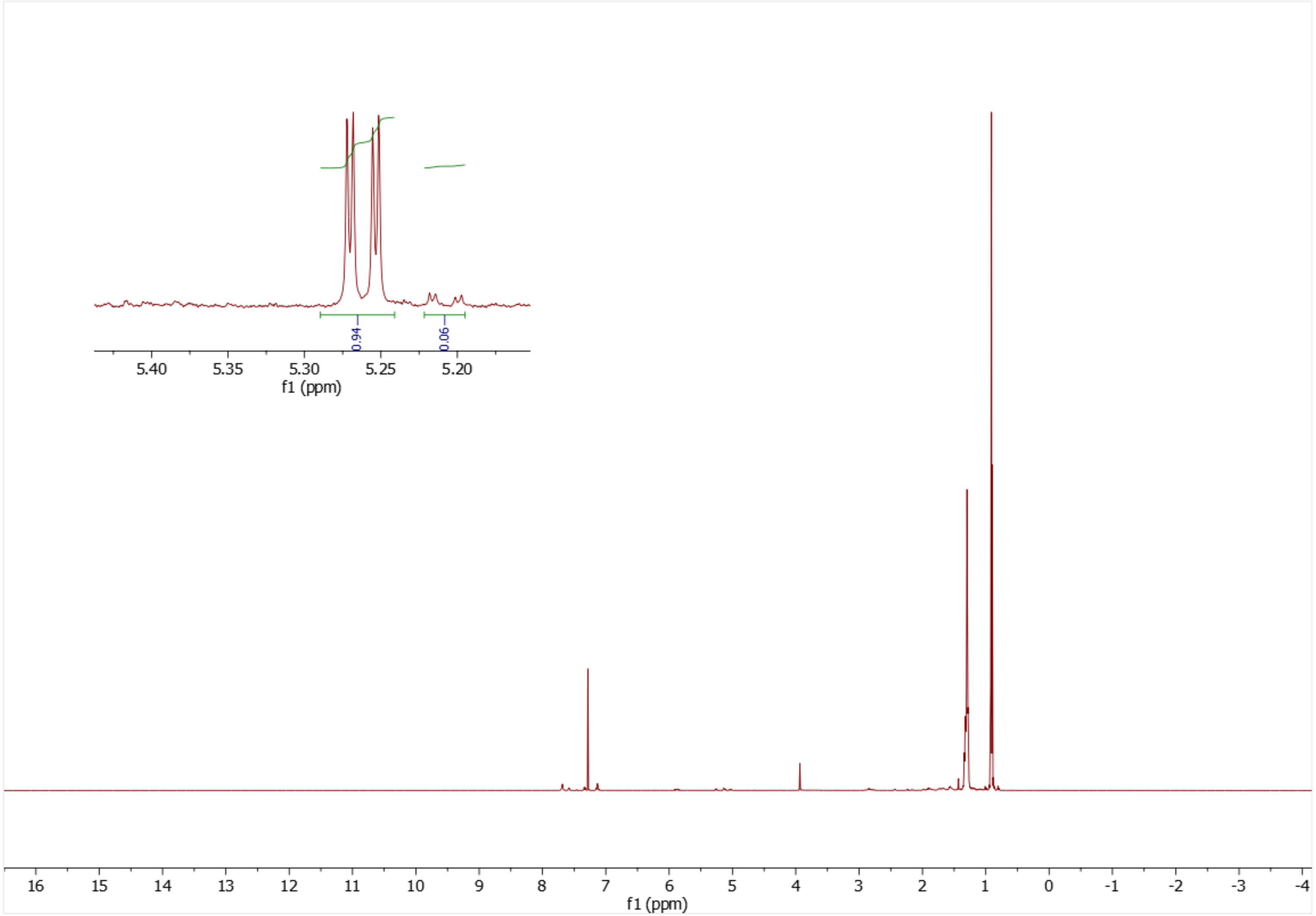

[1]**H NMR** (400 MHz, $CDCl_3$) Spectrum of the crude reaction mixture for **3ad** 94:6 *d.r.*

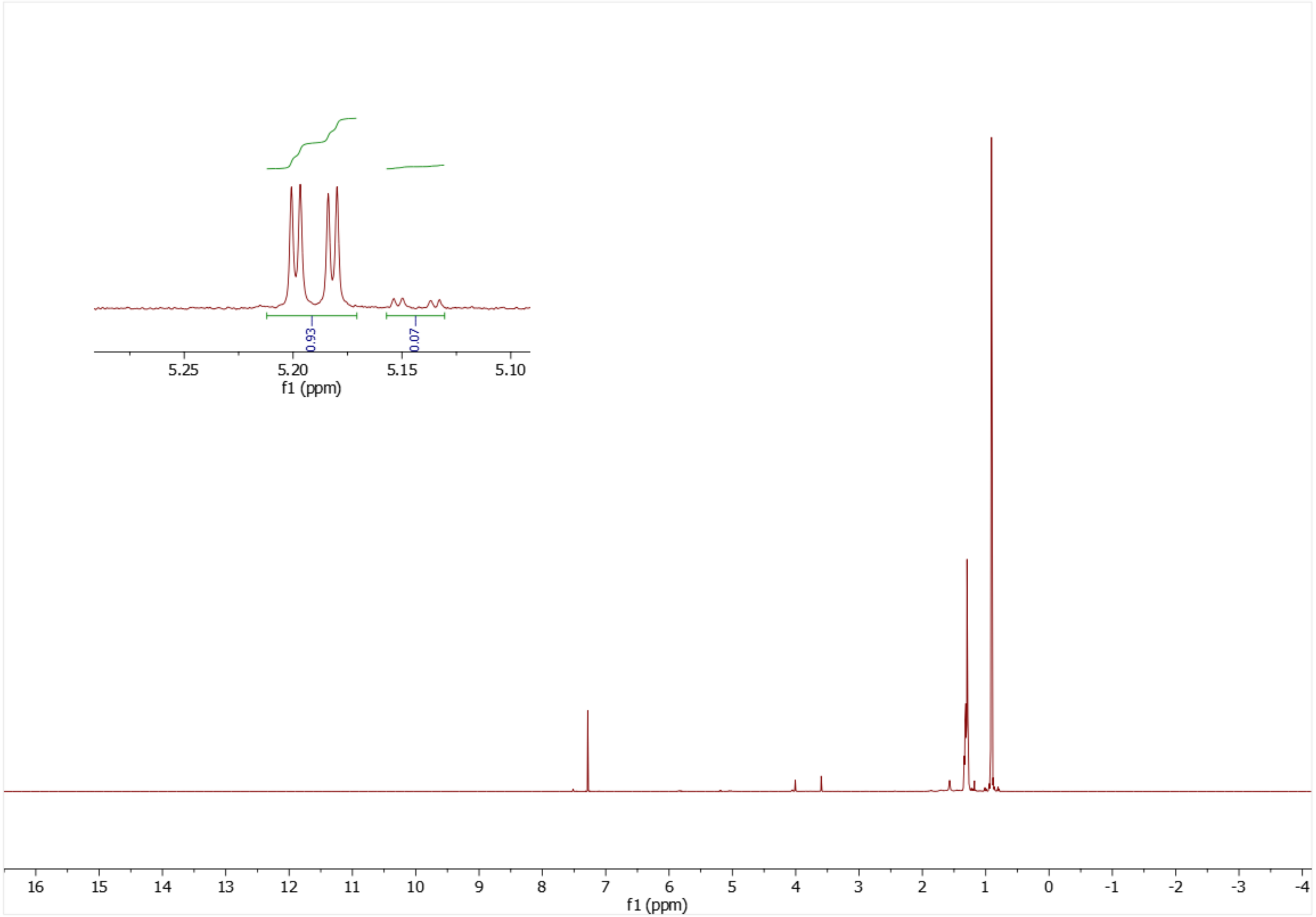

^{1}H NMR (400 MHz, $CDCl_3$) Spectrum of the crude reaction mixture for **3ae** 93:7 *d.r.*

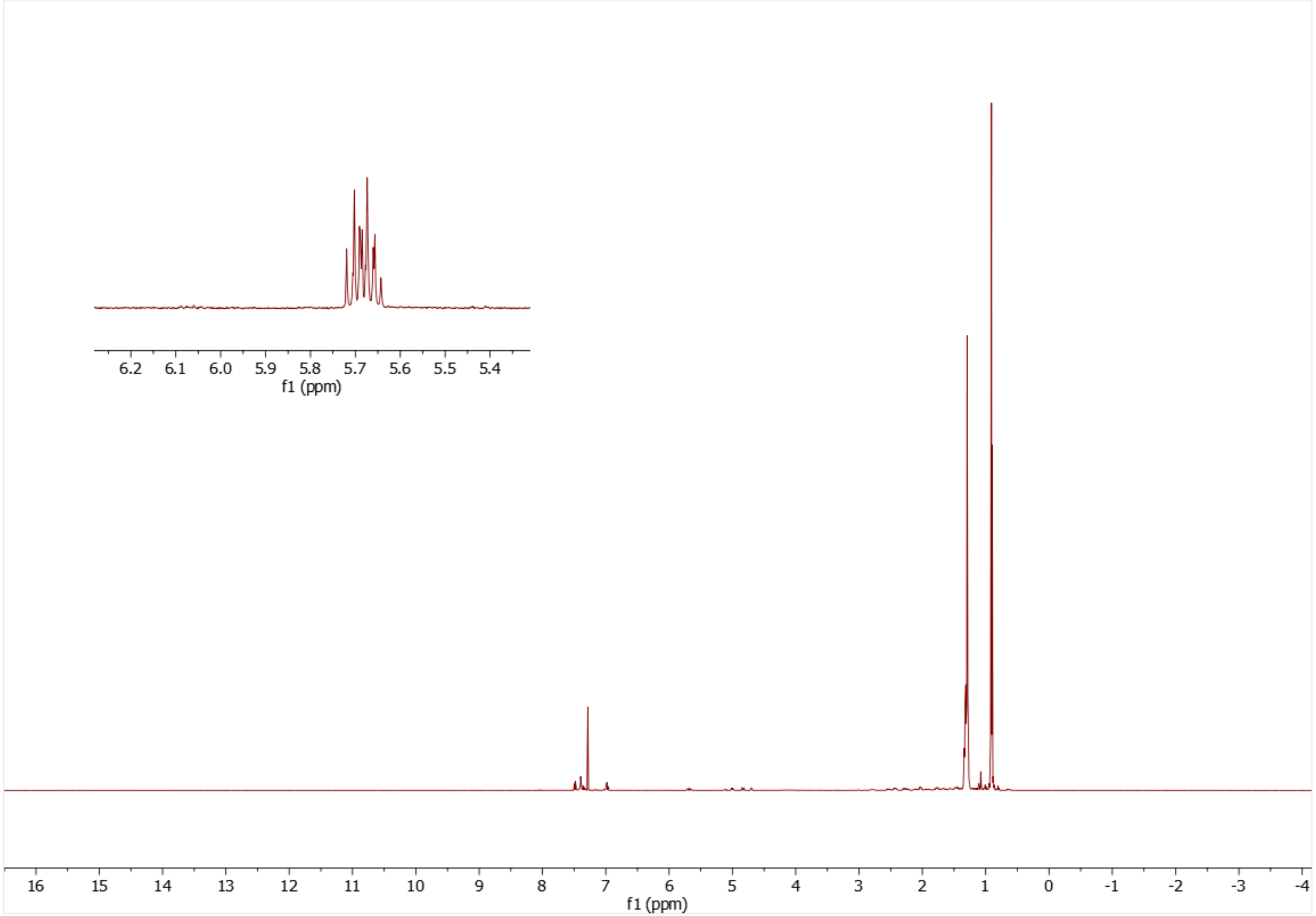

^{1}H NMR (400 MHz, $CDCl_3$) Spectrum of the crude reaction mixture for **3af** >95:5 *d.r.*

S11 Crystallographic data

3af

THE UNIVERSITY of EDINBURGH
Crystal Structure Service

Dr Gary S. Nichol
Crystallography Service Manager
Phone: +44 (0)131 650 4806
E–mail: g.s.nichol@ed.ac.uk

Submitted by: **Kieran Nicholson**

R_1=4.92%

Solved by: **Gary S Nichol**

Sample ID: **KN07-120**

Compound KN07-120 was provided as crystals suitable for single crystal X-ray diffraction, yielding structure 3af.

Crystal Data and Experimental

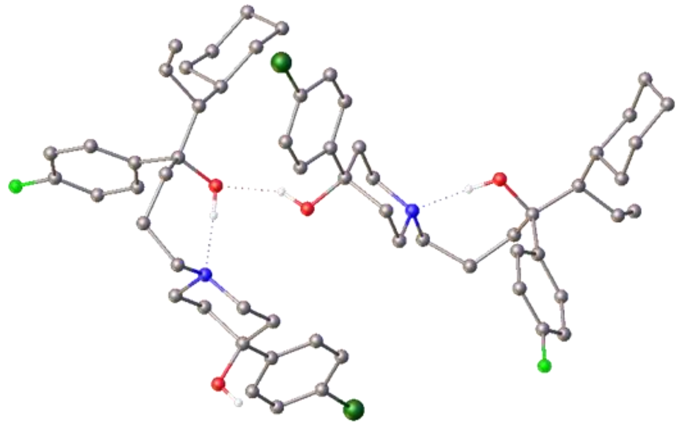

Experimental. Single colourless block-shaped crystals of **3af** recrystallised from a mixture of dichloromethane and petroleum ether by slow evaporation. A suitable crystal with dimensions 0.28 × 0.23 × 0.21 mm^3 was selected and mounted on a MITIGEN holder in Paratone oil on a Rigaku Oxford Diffraction XCalibur diffractometer. The crystal was kept at a steady T = 120.01(12) K during data collection. The structure was solved with the **ShelXT** 2018/2 (Sheldrick, 2018) solution program using dual methods and by using **Olex2** 1.5-beta (Dolomanov et al., 2009) as the graphical interface. The model was refined with **olex2.refine** 1.5-beta (Bourhis et al., 2015) using full matrix least squares minimisation on F^2.

Crystal Data. $C_{30}H_{39}ClFNO_2$, M_r = 500.100, monoclinic, $P2_1/c$ (No. 14), a = 26.4305(9) Å, b = 10.5769(3) Å, c = 19.9763(8) Å, β = 106.275(4)°, α = γ = 90°, V = 5360.6(3) Å^3, T = 120.01(12) K, Z = 8, Z' = 2, μ(Mo K$_\alpha$) = 0.177, 64971 reflections measured, 15011 unique (R_{int} = 0.0500) which were used in all calculations. The final wR_2 was 0.0784 (all data) and R_1 was 0.0492 (I≥2 σ(I)).

**Compound**	**3af**
Formula	$C_{30}H_{39}ClFNO_2$
$D_{calc.}$/ g cm^{-3}	1.239
μ/mm^{-1}	0.177
Formula Weight	500.100
Colour	colourless
Shape	block-shaped
Size/mm^3	0.28×0.23×0.21
T/K	120.01(12)
Crystal System	monoclinic
Space Group	$P2_1/c$
a/Å	26.4305(9)
b/Å	10.5769(3)
c/Å	19.9763(8)
α/°	90
β/°	106.275(4)
γ/°	90
V/Å^3	5360.6(3)
Z	8
Z'	2
Wavelength/Å	0.71073
Radiation type	Mo K_α
Θ_{min}/°	3.34
Θ_{max}/°	29.57
Measured Refl's.	64971
Indep't Refl's	15011
Refl's I≥2 σ(I)	11607
R_{int}	0.0500
Parameters	1333
Restraints	6
Largest Peak	0.4704
Deepest Hole	-0.5670
GooF	1.0921
wR_2 (all data)	0.0784
wR_2	0.0719
R_1 (all data)	0.0724
R_1	0.0492

Structure Quality Indicators

**Reflections:**	d min (Mo) 2Θ=59.1° 0.72	I/σ(I) 19.6	Rint 5.00%	Full 50.5° 99.7
**Refinement:**	Shift -0.000	Max Peak 0.5	Min Peak -0.6	GooF 1.092

A colourless block-shaped-shaped crystal with dimensions 0.28 × 0.23 × 0.21 mm^3 was mounted on a MITIGEN holder in Paratone oil. Data were collected using a Rigaku Oxford Diffraction XCalibur diffractometer equipped with an Oxford Cryosystems Cryostream 700+ low-temperature device operating at T = 120.01(12) K.

Data were measured using ω scans with Mo K_α radiation. The diffraction pattern was indexed and the total number of runs and images was based on the strategy calculation from the program CrysAlisPro 1.171.41.99a (Rigaku OD, 2021). The maximum resolution that was achieved was Θ = 29.57° (0.72 Å).

The unit cell was refined using CrysAlisPro 1.171.41.99a (Rigaku OD, 2021) on 12631 reflections, 19% of the observed reflections.

Data reduction, scaling and absorption corrections were performed using CrysAlisPro 1.171.41.99a (Rigaku OD, 2021). The final completeness is 99.74 % out to 29.57° in Θ. A multi-scan absorption correction was performed using CrysAlisPro 1.171.41.99a (Rigaku Oxford Diffraction, 2021) Empirical absorption correction using spherical harmonics, implemented in SCALE3 ABSPACK scaling algorithm.. The absorption coefficient μ of this material is 0.177 mm^{-1} at this wavelength (λ = 0.71073Å) and the minimum and maximum transmissions are 0.981 and 1.000.

The structure was solved and the space group $P2_1/c$ (# 14) determined by the ShelXT 2018/2 (Sheldrick, 2018) structure solution program using using dual methods and refined by full matrix least squares minimisation on $\boldsymbol{F}^2$ using version of **olex2.refine** 1.5-beta (Bourhis et al., 2015). All non-hydrogen atoms were refined anisotropically. Hydrogen atom positions were calculated geometrically and refined using the riding model.

_refine_special_details: H atoms were all identified from a difference map. The NoSpherA2 routine of Olex2 was used for refinement and details are given elsewhere.#===#>>> The Following Improvement and Query ALERTS were generated - (Acta-Mode) <<<#=== Format: alert-number_ALERT_alert-type_alert-level text417_ALERT_2_B Short Inter D-H..H-D H1 ..H52 . 2.05 Ang. x,y,z = 1_555 Check417_ALERT_2_B Short Inter D-H..H-D H2 ..H51 . 2.07 Ang. x,-1+y,z = 1_545 CheckH atoms identified from a difference map and refined using NoSpherA2910_ALERT_3_B Missing # of FCF Reflection(s) Below Theta(Min). 24 Note#===351_ALERT_3_C Long C-H (X0.96,N1.08A) C5 - H5 . 1.11 Ang.351_ALERT_3_C Long C-H (X0.96,N1.08A) C12 - H12B . 1.11 Ang.351_ALERT_3_C Long C-H (X0.96,N1.08A) C27 - H27B . 1.11 Ang.411_ALERT_2_C Short Inter H...H Contact H3B ..H24 . 2.13 Ang. -x,-y,-z = 3_555 CheckH atoms identified from a difference map and refined using NoSpherA2906_ALERT_3_C Large K Value in the Analysis of Variance ...... 9.347 Check906_ALERT_3_C Large K Value in the Analysis of Variance ...... 2.068 Check#===

The value of Z' is 2. This means that there are two independent molecules in the asymmetric unit.

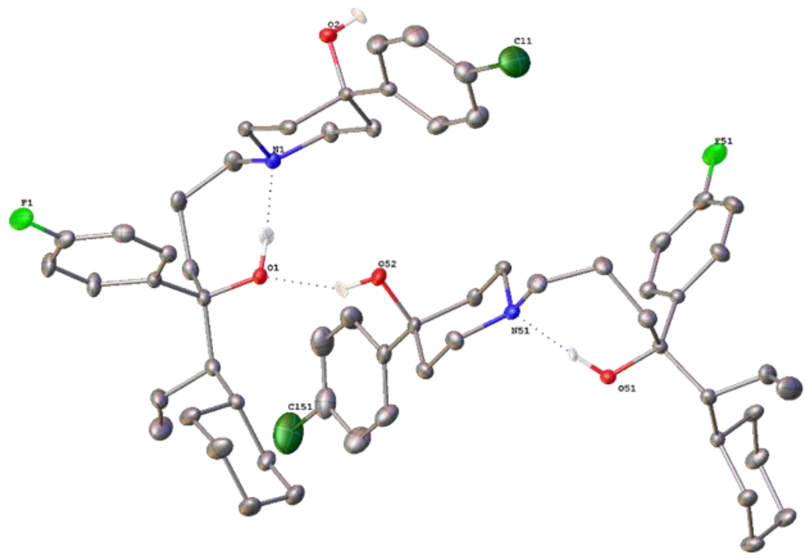

Figure S1: The asymmetric unit of 3af. Displacment ellipsoids are at the 50% probability level; C-bound H atoms are not shown.

Reflection Statistics

Total reflections (after filtering)	66728	Unique reflections	15011
Completeness	1.0	Mean I/σ	13.17
hkl_{max} collected	(36, 14, 25)	hkl_{min} collected	(-36, -14, -27)
hkl_{max} used	(35, 14, 27)	hkl_{min} used	(-36, 0, 0)
Lim d_{max} collected	100.0	Lim d_{min} collected	0.36
d_{max} used	6.1	d_{min} used	0.72
Friedel pairs	4598	Friedel pairs merged	1
Inconsistent equivalents	5	R_{int}	0.0504
R_{sigma}	0.0511	Intensity transformed	0
Omitted reflections	0	Omitted by user (OMIT hkl)	5
Multiplicity	(12118, 12967, 6073, 1970, 381, 112)	Maximum multiplicity	20
Removed systematic absences	1757	Filtered off (Shel/OMIT)	0

Table S3: Fractional Atomic Coordinates (×10^4) and Equivalent Isotropic Displacement Parameters (Å^2×10^3) for **3af.** U_{eq} is defined as 1/3 of the trace of the orthogonalised U_{ij}.

Atom	x	y	z	U_{eq}
Cl1	5332.86(13)	-310.3(4)	2951.5(2)	46.22(11)
F1	1377.9(3)	-2052.6(7)	-1568.0(4)	31.35(18)
O1	1689.8(3)	1115.8(8)	1169.4(4)	15.06(17)
O2	2978.1(3)	-2747.1(8)	2633.0(5)	20.51(19)
N1	1865.9(3)	-969.6(8)	1995.9(5)	14.88(19)
C1	1322.6(5)	-1374.4(13)	1927.4(7)	20.4(3)
C2	931.4(5)	-1123.5(12)	1219.8(7)	19.9(3)
C3	811.4(5)	269.2(12)	1028.4(7)	18.6(2)
C4	1183.4(4)	978.3(10)	683.0(6)	13.8(2)
C5	969.1(4)	2357.0(11)	519.9(6)	16.1(2)
C6	415.0(5)	2435.6(12)	48.6(7)	23.1(3)
C7	16.6(5)	2953.2(13)	231.3(9)	33.1(3)
C8	2162.6(5)	-992.3(13)	2739.8(6)	18.6(2)
C9	2736.5(5)	-616.7(11)	2863.5(6)	16.7(2)

Atom	x	y	z	U_{eq}
C10	3010.9(4)	-1458.9(10)	2449.5(6)	13.6(2)
C11	2691.7(5)	-1359.0(11)	1681.5(6)	15.7(2)
C12	2124.2(5)	-1774.0(11)	1587.5(6)	16.4(2)
C13	3590.4(4)	-1116.5(10)	2559.8(6)	15.3(2)
C14	3805.2(5)	37.0(11)	2827.4(7)	21.1(3)
C15	4340.7(5)	293.7(13)	2950.4(7)	26.6(3)
C16	4663.2(5)	-617.2(12)	2795.8(7)	25.9(3)
C17	4462.0(5)	-1771.2(13)	2516.8(7)	29.4(3)
C18	3928.4(5)	-2013.0(12)	2403.5(7)	24.1(3)
C19	1234.6(4)	224.7(10)	47.9(6)	14.0(2)
C20	1728.0(4)	-81.9(11)	-29.7(6)	16.3(2)
C21	1779.4(5)	-848.4(11)	-574.4(6)	20.8(3)
C22	1330.1(5)	-1298.5(11)	-1045.7(6)	21.7(3)
C23	834.6(5)	-1015.2(12)	-993.3(7)	24.6(3)
C24	791.6(5)	-258.4(12)	-444.5(6)	21.6(3)
C25	1340.0(4)	3276.1(10)	278.4(6)	15.9(2)
C26	1339.9(6)	3170.0(13)	-486.5(7)	26.0(3)
C27	1733.3(7)	4092.9(13)	-645.9(8)	31.4(3)
C28	1606.6(7)	5453.7(13)	-501.7(7)	30.7(3)
C29	1583.1(6)	5601.5(13)	245.8(7)	26.4(3)
C30	1209.1(5)	4644.3(12)	424.2(7)	22.7(3)
Cl51	-242.43(14)	4084.2(5)	2263.1(3)	58.40(14)
F51	3737.2(3)	2925.2(7)	6654.0(4)	37.3(2)
O51	3329.0(3)	6243.9(8)	3951.3(4)	14.64(17)
O52	2177.5(3)	2202.9(8)	2417.7(5)	23.7(2)
N51	3219.5(3)	4166.3(8)	3123.1(5)	14.61(19)
C51	3775.0(5)	3918.4(13)	3171.2(7)	20.6(3)
C52	4164.8(5)	4191.9(12)	3880.1(7)	19.7(3)
C53	4232.5(5)	5589.7(11)	4088.3(7)	18.4(2)
C54	3837.2(4)	6172.3(10)	4441.2(6)	13.3(2)
C55	4008.4(4)	7580.1(10)	4627.2(6)	15.0(2)
C56	4547.2(5)	7729.6(12)	5132.4(7)	22.8(3)
C57	4955.7(6)	8242.5(14)	4972.3(10)	35.0(4)
C58	3002.9(5)	3216.8(11)	3503.8(6)	16.9(2)
C59	2420.3(5)	3451.3(12)	3426.9(6)	17.6(2)
C60	2100.3(4)	3430.2(10)	2658.6(6)	16.2(2)
C61	2337.2(5)	4424.6(11)	2279.4(6)	17.9(2)
C62	2919.3(5)	4183.6(13)	2380.8(6)	18.9(2)
C63	1512.1(4)	3636.6(11)	2569.4(6)	18.6(2)
C64	1199.7(5)	2614.3(14)	2641.5(7)	31.0(3)
C65	662.1(6)	2747.9(15)	2554.8(8)	36.4(4)
C66	431.6(5)	3921.0(15)	2384.1(7)	33.6(3)
C67	729.5(6)	4950.8(15)	2309.8(8)	36.0(3)
C68	1268.9(5)	4800.5(13)	2404.6(7)	27.6(3)
C69	3810.1(4)	5350.2(10)	5062.7(5)	13.4(2)
C70	4266.8(5)	4941.4(12)	5557.8(6)	21.7(3)
C71	4248.2(5)	4134.5(12)	6099.5(7)	27.0(3)
C72	3761.4(5)	3724.6(11)	6140.4(6)	24.6(3)
C73	3300.1(5)	4100.2(11)	5664.0(6)	22.3(3)
C74	3328.2(5)	4914.7(11)	5128.8(6)	16.7(2)
C75	3596.9(4)	8410.7(10)	4842.4(6)	14.8(2)
C76	3712.2(5)	9813.6(11)	4755.9(7)	20.5(3)
C77	3290.9(6)	10675.2(13)	4895.7(7)	27.1(3)
C78	3217.9(6)	10431.3(12)	5614.9(7)	26.7(3)
C79	3116.4(6)	9040.6(12)	5714.6(8)	25.7(3)
C80	3550.8(5)	8205.8(12)	5583.2(6)	20.5(3)

Table S4: Anisotropic Displacement Parameters (×10[4]) for **3af.** The anisotropic displacement factor exponent takes the form: $-2\pi^2[h^2a^{*2} \times U_{11}+ ... +2hka^* \times b^* \times U_{12}]$

Atom	U_{11}	U_{22}	U_{33}	U_{23}	U_{13}	U_{12}
Cl1	19.93(17)	55.0(3)	65.4(3)	1.23(16)	14.74(18)	2.2(2)
F1	52.1(5)	24.3(4)	20.2(4)	5.2(3)	14.4(4)	-3.7(3)
O1	15.3(4)	13.4(4)	14.6(4)	-2.4(3)	1.1(3)	-0.9(3)
H1	19(9)	36(12)	37(12)	-17(9)	10(9)	3(10)
O2	26.3(5)	12.9(4)	18.5(4)	0.4(3)	-0.0(4)	3.2(4)
H2	68(13)	45(12)	17(10)	9(9)	-14(10)	12(9)
N1	18.7(5)	12.6(5)	13.0(5)	-2.4(4)	3.8(4)	1.5(4)
C1	22.4(6)	21.9(7)	16.8(6)	-5.9(5)	5.5(5)	3.4(5)
H1a	28(9)	78(13)	38(10)	-6(8)	17(8)	-14(9)
H1b	37(9)	37(10)	70(13)	-5(8)	7(9)	26(9)
C2	17.7(6)	21.8(6)	18.9(6)	-8.0(5)	3.0(5)	-0.3(5)
H2a	35(9)	66(12)	42(10)	-22(8)	5(8)	12(9)
H2b	52(10)	36(9)	24(9)	1(8)	19(8)	-15(7)
C3	14.7(6)	23.5(6)	16.9(6)	-2.4(5)	3.5(5)	-2.0(5)
H3a	57(10)	47(10)	30(10)	-5(8)	23(8)	-6(8)
H3b	9(7)	46(10)	51(10)	-2(7)	4(7)	18(8)
C4	12.8(5)	14.8(5)	12.7(5)	-0.6(4)	1.8(4)	-1.8(4)
C5	15.5(5)	16.8(6)	14.6(6)	1.6(4)	1.9(5)	-2.6(5)
H5	34(8)	28(8)	24(9)	-6(6)	12(7)	-10(7)
C6	17.0(6)	22.4(6)	25.1(7)	3.2(5)	-2.0(5)	-3.4(5)
H6	36(9)	66(12)	35(10)	3(8)	-5(8)	-11(9)
C7	16.2(7)	28.7(7)	50.3(10)	3.2(6)	2.6(7)	-6.8(7)
H7a	61(11)	59(12)	65(13)	4(9)	26(10)	-39(11)
H7b	16(8)	67(12)	99(15)	3(8)	-19(9)	-2(11)
C8	20.8(6)	23.0(7)	12.3(5)	-1.9(5)	5.2(5)	-0.3(5)
H8a	42(9)	26(9)	33(9)	-15(7)	9(7)	17(7)
H8b	42(10)	69(12)	40(10)	-9(9)	20(8)	-13(9)
C9	19.9(6)	17.3(6)	12.0(6)	0.1(5)	3.1(5)	-2.4(5)
H9a	52(9)	13(8)	25(9)	4(7)	20(7)	5(7)
H9b	35(9)	72(12)	22(9)	-21(8)	5(7)	0(8)
C10	18.6(6)	10.1(5)	10.7(5)	1.3(4)	2.0(4)	0.6(4)
C11	23.1(6)	12.9(6)	11.6(5)	3.5(5)	5.7(5)	0.6(4)
H11a	66(11)	40(10)	38(10)	15(8)	22(9)	-10(8)
H11b	44(9)	9(8)	50(10)	4(7)	14(8)	15(7)
C12	22.6(6)	12.7(6)	11.8(5)	-0.9(5)	1.2(5)	0.4(4)
H12a	31(8)	42(9)	19(9)	1(7)	-1(7)	-11(7)
H12b	62(10)	6(8)	50(11)	-15(7)	11(8)	6(7)
C13	19.4(6)	13.5(5)	12.7(5)	3.0(4)	4.3(4)	1.4(4)
C14	21.0(6)	15.8(6)	28.9(7)	1.4(5)	11.2(5)	-1.5(5)
H14	47(10)	22(9)	90(14)	-3(7)	39(9)	-28(9)
C15	22.8(7)	22.6(7)	35.7(8)	-2.7(5)	10.4(6)	-1.2(6)
H15	57(10)	31(10)	71(13)	-27(8)	24(9)	-23(9)
C16	20.4(6)	30.7(7)	27.3(7)	3.7(5)	8.0(5)	3.9(6)
C17	24.5(7)	29.3(7)	36.0(8)	11.0(6)	11.1(6)	-1.2(6)
H17	62(11)	51(11)	101(16)	37(9)	47(11)	0(10)
C18	24.3(7)	20.1(7)	27.5(7)	5.2(5)	6.8(5)	-3.6(5)
H18	57(11)	32(10)	73(13)	-20(8)	21(9)	-26(9)
C19	14.4(5)	12.7(5)	13.8(5)	-2.0(4)	2.3(4)	-0.6(4)
C20	15.4(6)	16.2(6)	17.4(6)	-1.1(4)	4.5(5)	0.8(4)
H20	23(8)	57(10)	27(9)	-1(7)	3(7)	-19(8)
C21	24.4(7)	20.4(6)	21.2(6)	4.3(5)	12.5(5)	2.4(5)
H21	54(10)	60(11)	65(12)	8(9)	45(9)	-12(9)
C22	33.9(7)	16.2(6)	16.7(6)	1.7(5)	9.9(5)	-1.1(5)
C23	27.0(7)	25.8(7)	18.5(6)	-4.7(5)	2.4(5)	-9.6(5)
H23	60(11)	78(13)	44(11)	-15(10)	-10(9)	-43(10)
C24	16.0(6)	27.6(7)	20.1(6)	-3.8(5)	3.2(5)	-9.0(5)
H24	28(9)	56(11)	55(11)	-11(8)	12(8)	-22(9)
C25	16.9(6)	15.3(5)	14.2(5)	3.0(4)	1.9(5)	0.7(4)
H25	13(4)	44(9)	34(8)	5(3)	-1(3)	9(6)
C26	45.5(9)	15.5(6)	16.9(6)	0.3(6)	8.5(6)	-0.1(5)
H26a	55(11)	47(11)	28(10)	-8(9)	-12(8)	8(8)

Atom	U_{11}	U_{22}	U_{33}	U_{23}	U_{13}	U_{12}
H26b	127(16)	29(10)	68(13)	-10(10)	60(12)	-2(9)
C27	53.7(10)	22.4(7)	23.0(7)	-1.8(7)	18.6(7)	4.2(6)
H27a	145(17)	49(12)	34(11)	-23(11)	43(12)	-6(9)
H27b	44(11)	66(13)	60(13)	-1(9)	19(10)	11(10)
C28	46.8(9)	17.4(7)	24.6(7)	-3.6(6)	4.4(7)	5.3(6)
H28a	61(11)	63(12)	38(11)	15(9)	-10(9)	10(9)
H28b	76(12)	49(11)	41(11)	-23(10)	32(10)	-2(9)
C29	33.7(8)	16.9(7)	25.7(7)	-4.0(6)	3.5(6)	-2.5(5)
H29a	35(10)	71(13)	44(11)	-17(9)	-7(9)	14(9)
H29b	85(13)	12(9)	80(14)	0(8)	29(11)	5(9)
C30	23.8(7)	18.2(6)	24.4(7)	0.6(5)	4.1(6)	-6.0(5)
H30a	83(13)	58(12)	31(10)	4(9)	35(10)	-12(9)
H30b	34(9)	30(9)	64(12)	6(7)	11(9)	-4(8)
Cl51	25.90(19)	79.9(3)	74.4(3)	-8.09(19)	22.3(2)	-25.4(3)
F51	70.6(6)	21.1(4)	20.4(4)	-7.8(4)	12.7(4)	4.1(3)
O51	16.2(4)	12.1(4)	14.3(4)	1.9(3)	1.9(3)	0.2(3)
H51	20(9)	26(11)	22(11)	4(8)	-9(8)	-7(9)
O52	28.1(5)	14.2(4)	22.0(5)	0.3(4)	-4.0(4)	-4.9(4)
H52	72(14)	38(11)	3(9)	9(10)	-8(9)	-3(8)
N51	19.4(5)	11.5(5)	12.7(4)	1.3(4)	4.1(4)	-1.3(4)
C51	23.6(6)	20.5(7)	19.0(6)	4.4(5)	8.0(5)	-3.2(5)
H51a	46(10)	37(10)	53(11)	13(8)	5(8)	-22(9)
H51b	33(9)	59(11)	36(10)	-3(8)	11(8)	13(9)
C52	16.9(6)	20.9(6)	20.7(6)	6.6(5)	4.2(5)	-1.0(5)
H52a	47(9)	43(10)	27(9)	-9(8)	15(8)	9(8)
H52b	18(8)	46(10)	67(12)	17(7)	8(8)	-13(9)
C53	16.5(6)	19.7(6)	19.6(6)	2.0(5)	5.9(5)	0.2(5)
H53a	52(10)	40(10)	38(10)	6(8)	21(8)	17(8)
H53b	25(8)	43(10)	35(10)	9(7)	1(7)	-9(8)
C54	12.4(5)	13.1(5)	13.1(5)	0.8(4)	1.7(4)	0.8(4)
C55	15.6(5)	14.5(5)	14.3(6)	-1.3(4)	3.3(5)	1.6(4)
H55	32(8)	47(10)	10(8)	9(7)	9(7)	5(7)
C56	16.9(6)	21.8(6)	25.9(7)	-3.9(5)	-0.4(5)	1.6(5)
H56	71(12)	74(13)	19(10)	-8(9)	-4(9)	8(9)
C57	18.2(7)	30.3(8)	52.8(10)	-6.0(6)	3.8(7)	7.1(7)
H57a	54(11)	87(15)	62(13)	-3(10)	8(10)	35(12)
H57b	7(8)	72(13)	103(15)	-19(8)	-9(9)	5(11)
C58	24.2(6)	12.0(6)	12.3(6)	0.0(5)	1.6(5)	1.1(5)
H58a	42(9)	45(10)	14(8)	1(7)	-2(7)	1(7)
H58b	44(9)	24(9)	39(10)	12(7)	5(8)	-6(7)
C59	24.4(6)	15.3(6)	11.9(5)	-5.3(5)	3.3(5)	0.8(5)
H59a	38(9)	32(9)	42(10)	-1(7)	4(8)	-25(8)
H59b	42(9)	46(10)	48(11)	-22(8)	19(8)	23(8)
C60	21.4(6)	12.9(5)	11.5(5)	-2.5(4)	0.3(5)	-1.5(4)
C61	22.6(6)	17.0(6)	12.5(6)	0.8(5)	2.6(5)	2.2(5)
H61a	49(10)	26(9)	52(11)	7(8)	4(8)	-7(8)
H61b	38(9)	58(11)	12(8)	3(7)	-12(7)	5(7)
C62	23.3(6)	20.8(6)	12.7(6)	1.0(5)	5.5(5)	1.0(5)
H62a	44(10)	66(12)	26(9)	11(8)	8(8)	16(8)
H62b	56(10)	34(10)	31(9)	2(8)	19(8)	-21(8)
C63	22.4(6)	18.6(6)	13.4(5)	-4.7(5)	2.7(5)	-2.5(4)
C64	27.8(7)	28.2(8)	32.8(8)	-9.3(6)	1.7(6)	7.2(6)
H64	58(12)	44(12)	101(16)	0(9)	10(11)	32(11)
C65	30.2(8)	43.1(9)	35.6(8)	-16.5(7)	8.9(7)	0.6(7)
H65	57(11)	54(12)	128(18)	-28(9)	35(12)	38(12)
C66	26.3(7)	45.8(9)	30.9(7)	-6.9(6)	11.5(6)	-13.3(7)
C67	28.2(8)	31.8(8)	51.2(9)	2.5(6)	16.4(7)	-10.2(7)
H67	46(11)	39(12)	160(20)	7(9)	51(12)	-17(12)
C68	25.6(7)	20.1(7)	40.0(8)	-0.9(5)	13.8(6)	-6.5(6)
H68	43(10)	20(10)	146(19)	4(8)	42(11)	8(10)
C69	14.4(5)	12.1(5)	12.7(5)	1.4(4)	2.1(4)	0.2(4)
C70	19.0(6)	23.1(6)	19.9(6)	2.1(5)	0.3(5)	4.8(5)

Atom	U_{11}	U_{22}	U_{33}	U_{23}	U_{13}	U_{12}
H70	22(8)	56(11)	58(11)	-9(8)	3(8)	18(9)
C71	34.0(8)	22.7(7)	18.2(6)	5.0(6)	-2.6(6)	5.4(5)
H71	53(11)	70(13)	50(12)	15(9)	-13(9)	31(10)
C72	44.4(8)	14.2(6)	14.8(6)	-1.9(5)	7.9(6)	0.2(5)
C73	32.6(7)	16.7(6)	21.3(6)	-6.5(5)	13.8(6)	-2.1(5)
H73	61(11)	56(11)	61(12)	-21(9)	33(9)	14(9)
C74	18.9(6)	15.2(6)	16.2(6)	-0.6(5)	5.4(5)	-0.6(4)
H74	29(9)	53(11)	46(10)	-1(7)	-5(8)	24(8)
C75	16.0(6)	13.8(5)	13.0(5)	-1.3(4)	1.6(5)	-0.5(4)
H75	18(7)	32(9)	31(9)	8(6)	7(7)	-13(7)
C76	28.2(7)	15.0(6)	18.3(6)	-0.9(5)	6.3(5)	2.6(5)
H76a	79(11)	51(11)	25(5)	5(9)	27(3)	12(4)
H76b	32(9)	46(10)	49(11)	-15(7)	-8(8)	-3(8)
C77	43.5(9)	15.6(6)	21.9(7)	6.9(6)	8.6(6)	3.0(5)
H77a	51(11)	77(13)	18(9)	22(9)	-7(8)	-8(9)
H77b	106(14)	18(9)	68(13)	-2(9)	37(11)	11(9)
C78	43.8(9)	15.5(6)	20.7(7)	3.7(6)	9.1(6)	-2.8(5)
H78a	70(12)	35(10)	45(11)	-5(9)	8(10)	-11(8)
H78b	88(13)	32(10)	73(13)	26(9)	50(11)	1(9)
C79	40.7(8)	17.6(6)	23.9(7)	-0.6(6)	17.4(6)	-3.3(5)
H79a	30(9)	62(12)	57(12)	-2(8)	12(9)	-36(10)
H79b	110(14)	39(10)	35(10)	2(9)	45(10)	5(8)
C80	34.0(7)	11.9(6)	17.2(6)	-1.2(5)	9.9(6)	0.3(5)
H80a	78(11)	21(9)	40(10)	-5(8)	35(9)	-1(7)
H80b	47(10)	47(10)	21(9)	-7(8)	2(8)	-8(8)

Table S5: Bond Lengths in Å for **3af.**

Atom	Atom	Length/Å
Cl1	C16	1.7396(12)
F1	C22	1.3471(13)
O1	C4	1.4239(12)
O2	C10	1.4200(13)
N1	C1	1.4675(15)
N1	C8	1.4735(14)
N1	C12	1.4724(15)
C1	C2	1.5227(17)
C2	C3	1.5325(17)
C3	C4	1.5446(16)
C4	C5	1.5654(15)
C4	C19	1.5360(15)
C5	C6	1.5051(16)
C5	C25	1.5506(16)
C6	C7	1.3256(19)
C8	C9	1.5195(16)
C9	C10	1.5297(16)
C10	C11	1.5333(15)
C10	C13	1.5283(15)
C11	C12	1.5231(16)
C13	C14	1.3880(16)
C13	C18	1.3969(16)
C14	C15	1.3937(17)
C15	C16	1.3777(18)
C16	C17	1.3845(18)
C17	C18	1.3881(18)
C19	C20	1.3941(15)
C19	C24	1.3978(15)
C20	C21	1.3936(16)
C21	C22	1.3772(17)

Atom	Atom	Length/Å
C22	C23	1.3758(17)
C23	C24	1.3877(17)
C25	C26	1.5320(17)
C25	C30	1.5348(16)
C26	C27	1.5235(19)
C27	C28	1.5234(19)
C28	C29	1.5198(19)
C29	C30	1.5253(19)
Cl51	C66	1.7380(13)
F51	C72	1.3449(13)
O51	C54	1.4252(12)
O52	C60	1.4189(13)
N51	C51	1.4677(15)
N51	C58	1.4693(14)
N51	C62	1.4729(14)
C51	C52	1.5273(17)
C52	C53	1.5327(17)
C53	C54	1.5431(16)
C54	C55	1.5702(15)
C54	C69	1.5338(15)
C55	C56	1.5054(16)
C55	C75	1.5496(16)
C56	C57	1.3251(19)
C58	C59	1.5243(17)
C59	C60	1.5309(15)
C60	C61	1.5290(16)
C60	C63	1.5301(16)
C61	C62	1.5161(17)
C63	C64	1.3922(17)
C63	C68	1.3850(17)

Atom	Atom	Length/Å
C64	C65	1.390(2)
C65	C66	1.382(2)
C66	C67	1.376(2)
C67	C68	1.3937(19)
C69	C70	1.3976(15)
C69	C74	1.3949(15)
C70	C71	1.3898(17)
C71	C72	1.3814(18)

Atom	Atom	Length/Å
C72	C73	1.3784(17)
C73	C74	1.3912(16)
C75	C76	1.5342(16)
C75	C80	1.5339(16)
C76	C77	1.5239(18)
C77	C78	1.5241(19)
C78	C79	1.5184(18)
C79	C80	1.5284(18)

Table S6: Bond Angles in ° for **3af**.

Atom	Atom	Atom	Angle/°
C8	N1	C1	108.64(9)
C12	N1	C1	111.92(9)
C12	N1	C8	110.55(9)
C2	C1	N1	115.33(10)
C3	C2	C1	115.99(10)
C4	C3	C2	117.71(10)
C3	C4	O1	109.82(9)
C5	C4	O1	105.25(8)
C5	C4	C3	107.86(9)
C19	C4	O1	109.46(8)
C19	C4	C3	109.36(9)
C19	C4	C5	114.96(9)
C6	C5	C4	114.43(9)
C25	C5	C4	115.16(9)
C25	C5	C6	111.10(10)
C7	C6	C5	124.00(13)
C9	C8	N1	112.62(10)
C10	C9	C8	111.27(10)
C9	C10	O2	110.28(9)
C11	C10	O2	105.55(9)
C11	C10	C9	106.83(9)
C13	C10	O2	108.75(8)
C13	C10	C9	113.07(9)
C13	C10	C11	112.09(9)
C12	C11	C10	110.50(10)
C11	C12	N1	111.03(9)
C14	C13	C10	123.30(10)
C18	C13	C10	119.00(10)
C18	C13	C14	117.68(11)
C15	C14	C13	121.65(12)
C16	C15	C14	119.07(12)
C15	C16	Cl1	119.46(10)
C17	C16	Cl1	119.55(10)
C17	C16	C15	120.98(12)
C18	C17	C16	119.09(12)
C17	C18	C13	121.52(12)
C20	C19	C4	120.95(9)
C24	C19	C4	121.37(10)
C24	C19	C20	117.51(11)
C21	C20	C19	121.40(11)
C22	C21	C20	118.74(11)
C21	C22	F1	118.96(11)
C23	C22	F1	119.07(11)
C23	C22	C21	121.97(11)
C24	C23	C22	118.44(11)
C23	C24	C19	121.93(12)

Atom	Atom	Atom	Angle/°
C26	C25	C5	116.17(10)
C30	C25	C5	109.64(10)
C30	C25	C26	108.74(10)
C27	C26	C25	110.61(11)
C28	C27	C26	111.51(13)
C29	C28	C27	110.77(11)
C30	C29	C28	111.65(11)
C29	C30	C25	112.71(11)
C58	N51	C51	111.83(9)
C62	N51	C51	108.36(9)
C62	N51	C58	110.57(9)
C52	C51	N51	115.43(10)
C53	C52	C51	115.56(10)
C54	C53	C52	117.82(10)
C53	C54	O51	109.63(9)
C55	C54	O51	105.41(8)
C55	C54	C53	107.44(9)
C69	C54	O51	109.70(8)
C69	C54	C53	109.55(9)
C69	C54	C55	114.95(9)
C56	C55	C54	114.45(9)
C75	C55	C54	115.16(9)
C75	C55	C56	111.11(10)
C57	C56	C55	124.26(13)
C59	C58	N51	111.46(9)
C60	C59	C58	110.94(10)
C59	C60	O52	105.18(9)
C61	C60	O52	110.09(10)
C61	C60	C59	107.38(9)
C63	C60	O52	109.21(9)
C63	C60	C59	111.87(10)
C63	C60	C61	112.82(9)
C62	C61	C60	111.05(10)
C61	C62	N51	112.15(10)
C64	C63	C60	119.33(11)
C68	C63	C60	122.91(10)
C68	C63	C64	117.76(12)
C65	C64	C63	121.56(14)
C66	C65	C64	119.12(13)
C65	C66	Cl51	119.16(11)
C67	C66	Cl51	120.10(12)
C67	C66	C65	120.74(13)
C68	C67	C66	119.30(14)
C67	C68	C63	121.51(13)
C70	C69	C54	121.40(10)
C74	C69	C54	120.99(9)

Atom	Atom	Atom	Angle/˚
C74	C69	C70	117.47(11)
C71	C70	C69	121.93(12)
C72	C71	C70	118.40(12)
C71	C72	F51	119.12(11)
C73	C72	F51	119.07(11)
C73	C72	C71	121.81(11)
C74	C73	C72	118.78(12)
C73	C74	C69	121.61(11)

Atom	Atom	Atom	Angle/˚
C76	C75	C55	109.88(9)
C80	C75	C55	116.05(9)
C80	C75	C76	108.50(10)
C77	C76	C75	112.30(11)
C78	C77	C76	111.32(11)
C79	C78	C77	111.18(11)
C80	C79	C78	111.61(12)
C79	C80	C75	110.52(10)

Table S7: Torsion Angles in ˚ for **3af**.

Atom	Atom	Atom	Atom	Angle/˚
Cl1	C16	C15	C14	-179.66(10)
Cl1	C16	C17	C18	179.13(11)
F1	C22	C21	C20	-179.18(10)
F1	C22	C23	C24	178.71(11)
O1	C4	C3	C2	-67.48(10)
O1	C4	C5	C6	-175.90(9)
O1	C4	C5	C25	53.54(9)
O1	C4	C19	C20	-7.71(11)
O1	C4	C19	C24	167.46(9)
O2	C10	C9	C8	-57.84(10)
O2	C10	C11	C12	58.76(9)
O2	C10	C13	C14	-141.07(9)
O2	C10	C13	C18	37.14(11)
N1	C1	C2	C3	-67.11(12)
N1	C8	C9	C10	-56.38(11)
N1	C12	C11	C10	60.56(10)
C1	C2	C3	C4	85.95(12)
C2	C3	C4	C5	178.32(10)
C2	C3	C4	C19	52.65(11)
C3	C4	C5	C6	-58.70(10)
C3	C4	C5	C25	170.74(9)
C3	C4	C19	C20	-128.06(9)
C3	C4	C19	C24	47.10(11)
C4	C5	C6	C7	118.94(12)
C4	C5	C25	C26	79.41(11)
C4	C5	C25	C30	-156.84(10)
C4	C19	C20	C21	174.91(10)
C4	C19	C24	C23	-175.38(11)
C5	C25	C26	C27	-178.13(11)
C5	C25	C30	C29	176.09(10)
C8	C9	C10	C11	56.38(11)
C8	C9	C10	C13	-179.85(9)
C9	C10	C11	C12	-58.62(9)
C9	C10	C13	C14	-18.21(12)
C9	C10	C13	C18	160.00(10)
C10	C13	C14	C15	177.03(11)
C10	C13	C18	C17	-177.66(11)
C13	C14	C15	C16	0.65(14)
C13	C18	C17	C16	0.45(15)
C14	C15	C16	C17	0.51(16)
C15	C16	C17	C18	-1.04(15)
C19	C20	C21	C22	0.51(13)
C19	C24	C23	C22	0.45(15)
C20	C21	C22	C23	-0.09(14)
C21	C22	C23	C24	-0.38(14)
C25	C26	C27	C28	-59.02(12)

Atom	Atom	Atom	Atom	Angle/˚
C25	C30	C29	C28	54.37(12)
C26	C27	C28	C29	55.90(14)
C27	C28	C29	C30	-53.04(14)
Cl51	C66	C65	C64	179.07(11)
Cl51	C66	C67	C68	-179.60(12)
F51	C72	C71	C70	178.79(11)
F51	C72	C73	C74	-179.20(10)
O51	C54	C53	C52	-68.31(10)
O51	C54	C55	C56	-178.67(9)
O51	C54	C55	C75	50.74(9)
O51	C54	C69	C70	168.61(9)
O51	C54	C69	C74	-7.13(11)
O52	C60	C59	C58	60.38(10)
O52	C60	C61	C62	-57.66(10)
O52	C60	C63	C64	32.97(12)
O52	C60	C63	C68	-145.70(10)
N51	C51	C52	C53	-68.31(12)
N51	C58	C59	C60	58.54(10)
N51	C62	C61	C60	-57.63(11)
C51	C52	C53	C54	85.01(12)
C52	C53	C54	C55	177.62(11)
C52	C53	C54	C69	52.12(11)
C53	C54	C55	C56	-61.80(11)
C53	C54	C55	C75	167.60(9)
C53	C54	C69	C70	48.22(11)
C53	C54	C69	C74	-127.52(9)
C54	C55	C56	C57	113.36(12)
C54	C55	C75	C76	-159.84(10)
C54	C55	C75	C80	76.63(10)
C54	C69	C70	C71	-176.06(11)
C54	C69	C74	C73	175.66(10)
C55	C75	C76	C77	174.73(10)
C55	C75	C80	C79	-177.57(11)
C58	C59	C60	C61	-56.87(11)
C58	C59	C60	C63	178.83(9)
C59	C60	C61	C62	56.34(10)
C59	C60	C63	C64	-83.05(11)
C59	C60	C63	C68	98.28(11)
C60	C63	C64	C65	-178.97(12)
C60	C63	C68	C67	178.38(13)
C63	C64	C65	C66	0.83(17)
C63	C68	C67	C66	0.25(16)
C64	C65	C66	C67	-0.89(18)
C65	C66	C67	C68	0.36(17)
C69	C70	C71	C72	0.49(14)
C69	C74	C73	C72	0.34(13)
C70	C71	C72	C73	-0.40(15)
C71	C72	C73	C74	-0.01(14)
C75	C76	C77	C78	55.41(12)
C75	C80	C79	C78	-58.32(12)
C76	C77	C78	C79	-53.09(13)
C77	C78	C79	C80	55.03(13)

Table S8: Hydrogen Fractional Atomic Coordinates (×10^4) and Equivalent Isotropic Displacement Parameters (Å^2×10^3) for **3af**. U_{eq} is defined as 1/3 of the trace of the orthogonalised U_{ij}.

Atom	x	y	z	U_{eq}
H1	1760(6)	410(16)	1474(9)	30(5)
H2	3119(7)	-2842(15)	3081(8)	48(5)

Atom	x	y	z	U_{eq}
H1a	1199(5)	-868(15)	2322(8)	47(4)
H1b	1312(5)	-2384(14)	2050(8)	50(5)
H2a	572(5)	-1568(14)	1236(7)	49(4)
H2b	1051(5)	-1602(13)	801(7)	35(4)
H3a	807(6)	811(13)	1485(7)	42(4)
H3b	420(5)	351(12)	677(8)	36(4)
H5	953(5)	2695(12)	1041(7)	28(3)
H6	348(5)	2104(14)	-477(8)	49(4)
H7a	87(6)	3336(15)	756(9)	60(5)
H7b	-367(5)	3032(15)	-110(9)	68(6)
H8a	2136(5)	-1940(12)	2952(7)	34(4)
H8b	1972(5)	-336(15)	3012(8)	49(4)
H9a	2758(5)	383(12)	2721(7)	28(3)
H9b	2940(5)	-701(14)	3414(7)	43(4)
H11a	2870(6)	-1920(13)	1358(8)	46(4)
H11b	2707(5)	-399(11)	1509(7)	34(4)
H12a	1898(5)	-1700(12)	1046(7)	33(4)
H12b	2113(5)	-2776(12)	1746(8)	40(4)
H14	3561(5)	743(12)	2960(9)	49(5)
H15	4506(5)	1182(14)	3166(8)	52(5)
H17	4711(6)	-2462(15)	2396(10)	66(5)
H18	3771(6)	-2907(14)	2208(9)	54(5)
H20	2072(5)	278(13)	350(7)	37(4)
H21	2149(6)	-1107(14)	-636(8)	53(5)
H23	497(6)	-1386(16)	-1356(8)	66(5)
H24	410(5)	-85(14)	-391(8)	46(4)
H25	1738(5)	3082(12)	595(7)	32(4)
H26a	949(6)	3402(14)	-819(7)	48(4)
H26b	1421(7)	2218(15)	-618(9)	68(5)
H27a	1732(7)	3999(15)	-1193(9)	72(6)
H27b	2134(6)	3878(15)	-302(9)	56(5)
H28a	1235(6)	5711(14)	-836(8)	58(5)
H28b	1884(6)	6127(15)	-614(8)	52(5)
H29a	1969(6)	5450(15)	589(8)	54(5)
H29b	1462(6)	6553(13)	330(9)	58(5)
H30a	1211(6)	4719(14)	969(8)	54(5)
H30b	807(5)	4848(12)	108(8)	43(4)
H51	3267(5)	5509(15)	3660(8)	26(4)
H52	1985(7)	2101(15)	1974(8)	41(5)
H51a	3816(5)	2937(14)	3022(8)	47(4)
H51b	3871(5)	4517(14)	2786(7)	43(4)
H52a	4063(5)	3648(13)	4288(7)	38(4)
H52b	4540(5)	3836(13)	3849(8)	45(4)
H53a	4224(5)	6152(13)	3636(8)	42(4)
H53b	4624(5)	5726(12)	4452(7)	36(4)
H55	4029(5)	7966(12)	4122(6)	29(3)
H56	4600(6)	7424(15)	5656(8)	58(5)
H57a	4920(6)	8575(17)	4454(9)	70(6)
H57b	5332(5)	8347(15)	5352(9)	66(6)
H58a	3220(5)	3259(13)	4047(7)	36(4)
H58b	3053(5)	2271(12)	3320(7)	37(4)
H59a	2367(5)	4358(13)	3638(7)	39(4)
H59b	2275(5)	2749(13)	3708(8)	44(4)
H61a	2291(5)	5370(13)	2466(8)	44(4)
H61b	2128(5)	4407(13)	1732(7)	40(4)
H62a	3087(5)	4941(15)	2153(7)	46(4)
H62b	2974(5)	3274(13)	2140(7)	39(4)
H64	1382(6)	1703(15)	2747(10)	70(6)
H65	434(6)	1958(15)	2617(10)	78(6)
H67	548(6)	5872(15)	2180(11)	79(7)
H68	1491(6)	5611(14)	2332(10)	67(6)
H70	4649(5)	5243(14)	5521(8)	47(4)

Atom	x	y	z	U_{eq}
H71	4591(6)	3814(15)	6466(8)	63(5)
H73	2931(6)	3743(14)	5709(8)	56(5)
H74	2975(5)	5209(13)	4750(8)	46(4)
H75	3215(5)	8216(11)	4483(7)	27(3)
H76a	3754(6)	9959(14)	4238(8)	49(4)
H76b	4086(5)	10037(13)	5107(8)	46(4)
H77a	2928(6)	10504(15)	4510(7)	52(5)
H77b	3388(6)	11648(14)	4844(8)	62(5)
H78a	3565(6)	10717(13)	6009(8)	52(5)
H78b	2903(6)	11013(14)	5696(9)	59(5)
H79a	2746(5)	8765(14)	5344(8)	50(5)
H79b	3075(6)	8869(14)	6233(8)	56(5)
H80a	3479(6)	7221(13)	5664(7)	43(4)
H80b	3926(5)	8455(13)	5965(7)	40(4)

Table S9: Hydrogen Bond information for **3af**.

D	H	A	d(D-H)/Å	d(H-A)/Å	d(D-A)/Å	D-H-A/deg
O1	H1	N1	0.948(17)	1.769(18)	2.7160(12)	176.1(14)
O2	H2	O51[1]	0.874(15)	1.930(15)	2.7505(12)	155.8(16)
O51	H51	N51	0.957(16)	1.763(17)	2.7169(12)	174.2(13)
O52	H52	O1	0.897(15)	1.890(16)	2.7203(12)	153.1(15)

––––

[1]+x,-1+y,+z

3ab

THE UNIVERSITY *of* EDINBURGH
Crystal Structure Service

Dr Gary S. Nichol
Crystallography Service Manager
Phone: +44 (0)131 650 4806
E-mail: g.s.nichol@ed.ac.uk

Submitted by: **Kieran Nicholson**

R_1=2.31%

Solved by: **Gary S Nichol**

Sample ID: **KN07-113**

Compound KN07-113 was provided as crystals suitable for single crystal X-ray diffraction, yielding structure 3ab.

Crystal Data and Experimental

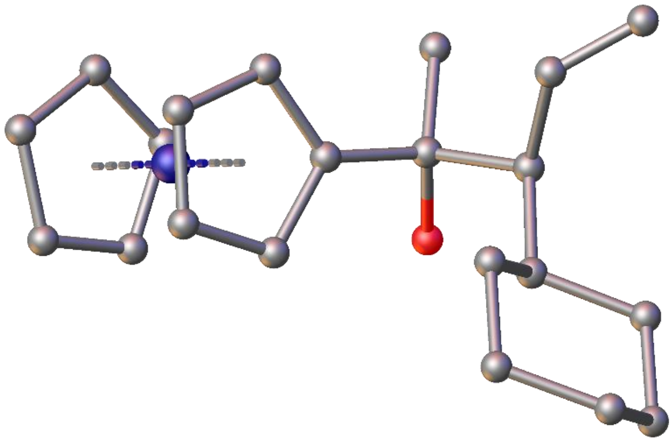

Experimental. Single translucent dark orange block-shaped crystals of **3ab** recrystallised from a mixture of petroleum ether and dichloromethane by slow evaporation. A suitable crystal with dimensions 0.37 × 0.27 × 0.14 mm^3 was selected and mounted on a MITIGEN holder in Paratone oil on a Rigaku Oxford Diffraction XCalibur diffractometer. The crystal was kept at a steady T = 120.00(11) K during data collection. The structure was solved with the **ShelXT** 2018/2 (Sheldrick, 2018) solution program using dual methods and by using **Olex2** 1.5-beta (Dolomanov et al., 2009) as the graphical interface. The model was refined with **olex2.refine** 1.5-beta (Bourhis et al., 2015) using full matrix least squares minimisation on $\boldsymbol{F^2}$.

Crystal Data. $C_{21}H_{28}FeO$, M_r = 352.301, monoclinic, $P2_1/c$ (No. 14), a = 9.7712(1) Å, b = 18.1209(2) Å, c = 10.2807(1) Å, β = 102.609(1)°, α = γ = 90°, V = 1776.43(3) Å^3, T = 120.00(11) K, Z = 4, Z' = 1, μ(Mo K_α) = 0.851, 56276 reflections measured, 6290 unique (R_{int} = 0.0358) which were used in all calculations. The final wR_2 was 0.0404 (all data) and R_1 was 0.0231 (I≥2 σ(I)).

**Compound**	**3ab**
Formula	$C_{21}H_{28}FeO$
$D_{calc.}$/ g cm^{-3}	1.317
μ/mm^{-1}	0.851
Formula Weight	352.301
Colour	translucent dark orange
Shape	block-shaped
Size/mm^3	0.37×0.27×0.14
T/K	120.00(11)
Crystal System	monoclinic
Space Group	$P2_1/c$
a/Å	9.7712(1)
b/Å	18.1209(2)
c/Å	10.2807(1)
α/°	90
β/°	102.609(1)
γ/°	90
V/Å^3	1776.43(3)
Z	4
Z'	1
Wavelength/Å	0.71073
Radiation type	Mo K_α
Θ_{min}/°	3.44
Θ_{max}/°	32.81
Measured Refl's.	56276
Indep't Refl's	6290
Refl's I≥2 σ(I)	5715
R_{int}	0.0358
Parameters	460
Restraints	0
Largest Peak	0.2623
Deepest Hole	-0.2935
GooF	1.0728
wR_2 (all data)	0.0404
wR_2	0.0389
R_1 (all data)	0.0280
R_1	0.0231

Structure Quality Indicators

**Reflections:**	d min (Mo) 2Θ=65.6° 0.66	I/σ(I) 43.3	Rint 3.58%	Full 50.5° 96% to 65.6° 99.8
**Refinement:**	Shift -0.001	Max Peak 0.3	Min Peak -0.3	GooF 1.073

A translucent dark orange block-shaped-shaped crystal with dimensions 0.37 × 0.27 × 0.14 mm^3 was mounted on a MITIGEN holder in Paratone oil. Data were collected using a Rigaku Oxford Diffraction XCalibur diffractometer equipped with an Oxford Cryosystems Cryostream 700+ low-temperature device operating at T = 120.00(11) K.

Data were measured using ω scans with Mo K_{α} radiation. The diffraction pattern was indexed and the total number of runs and images was based on the strategy calculation from the program CrysAlisPro 1.171.41.99a (Rigaku OD, 2021). The maximum resolution that was achieved was Θ = 32.81° (0.66 Å).

The unit cell was refined using CrysAlisPro 1.171.41.99a (Rigaku OD, 2021) on 24383 reflections, 43% of the observed reflections.

Data reduction, scaling and absorption corrections were performed using CrysAlisPro 1.171.41.99a (Rigaku OD, 2021). The final completeness is 99.78 % out to 32.81° in Θ. A multi-scan absorption correction was performed using CrysAlisPro 1.171.41.99a (Rigaku Oxford Diffraction, 2021) Empirical absorption correction using spherical harmonics, implemented in SCALE3 ABSPACK scaling algorithm.. The absorption coefficient μ of this material is 0.851 mm^{-1} at this wavelength (λ = 0.71073Å) and the minimum and maximum transmissions are 0.889 and 1.000.

The structure was solved and the space group $P2_1/c$ (# 14) determined by the ShelXT 2018/2 (Sheldrick, 2018) structure solution program using using dual methods and refined by full matrix least squares minimisation on $\boldsymbol{F}^2$ using version of **olex2.refine** 1.5-beta (Bourhis et al., 2015). All non-hydrogen atoms were refined anisotropically. Hydrogen atom positions were calculated geometrically and refined using the riding model.

_refine_special_details: H atoms were identified from a difference map and freely refined.The NoSpherA2 routine of Olex2 was used for final refinement and details are given elsewhere.

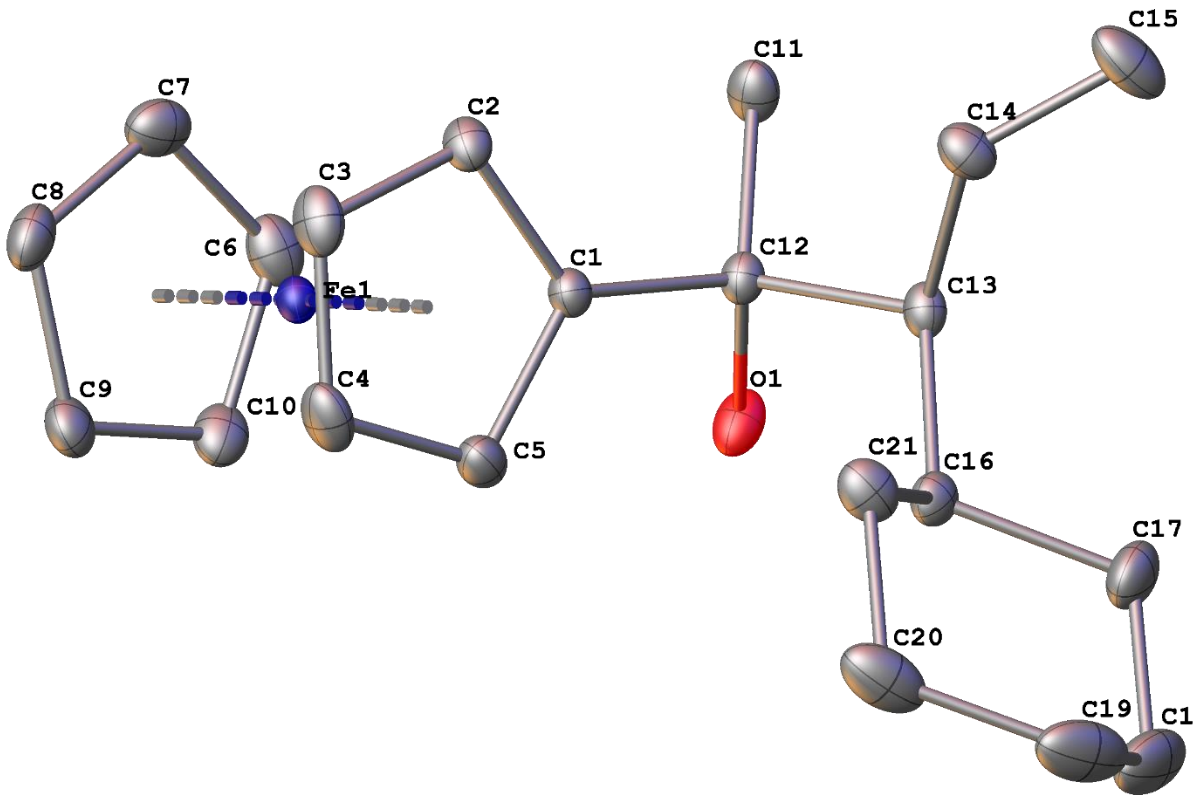

Figure S2: The molecular structure of 3ab. Displacement ellipsoids are at the 50% probability level and H atoms are not shown.

Data Plots: Diffraction Data

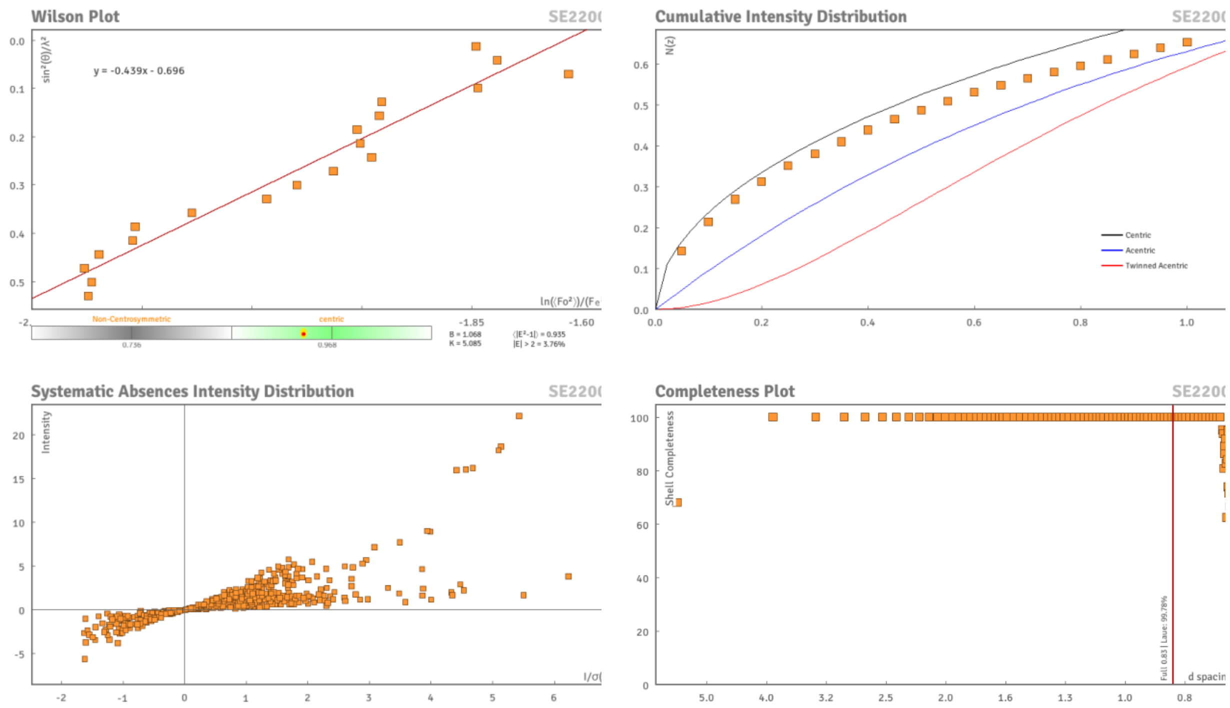

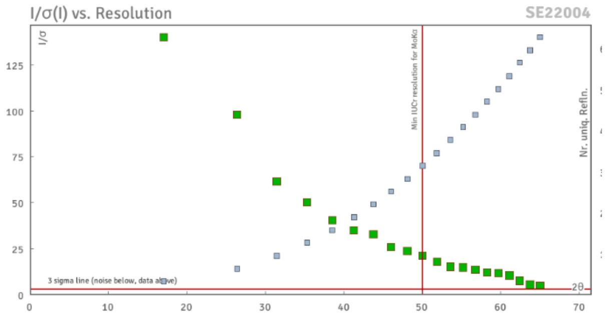

Data Plots: Refinement and Data

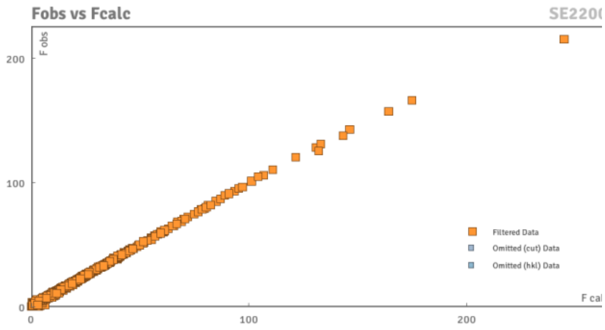

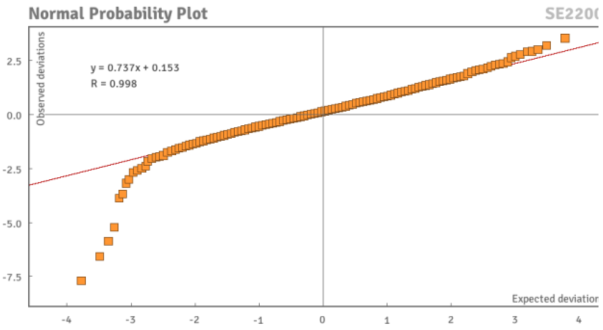

Reflection Statistics

Total reflections (after filtering)	57152	Unique reflections	6290
Completeness	0.955	Mean I/σ	33.28
hkl_{max} collected	(14, 27, 15)	hkl_{min} collected	(-14, -27, -14)
hkl_{max} used	(14, 27, 15)	hkl_{min} used	(-14, 0, 0)
Lim d_{max} collected	100.0	Lim d_{min} collected	0.36
d_{max} used	6.04	d_{min} used	0.66
Friedel pairs	11202	Friedel pairs merged	1
Inconsistent equivalents	3	R_{int}	0.0358
R_{sigma}	0.0231	Intensity transformed	0
Omitted reflections	0	Omitted by user (OMIT hkl)	0
Multiplicity	(6297, 8157, 4693, 2454, 1183, 424, 205, 58, 24, 5, 2)	Maximum multiplicity	36
Removed systematic absences	876	Filtered off (Shel/OMIT)	0

Table S10: Fractional Atomic Coordinates (×10⁴) and Equivalent Isotropic Displacement Parameters (Å^2×10^3) for **3ab.** U_{eq} is defined as 1/3 of the trace of the orthogonalised U_{ij}.

Atom	x	y	z	U_{eq}
Fe1	1149.02(10)	3463.54(5)	2565.46(10)	11.60(3)
O1	812.2(6)	5260.1(3)	1214.6(5)	18.84(11)
C1	2086.3(7)	4484.3(4)	2944.1(7)	12.16(12)
C2	2183.3(7)	4078.6(4)	4155.6(7)	14.91(13)
C3	2915.9(8)	3407.3(4)	4058.5(8)	19.41(14)
C4	3272.6(8)	3390.6(4)	2789.3(8)	20.71(15)
C5	2755.8(7)	4050.2(4)	2099.6(8)	17.08(13)
C6	-981.2(8)	3565.8(4)	1826.3(8)	21.38(15)
C7	-701.3(8)	3084.1(4)	2950.5(8)	22.38(15)
C8	127.2(8)	2490.0(4)	2648.9(8)	20.95(15)
C9	360.9(8)	2603.3(4)	1346.5(8)	19.57(14)
C10	-321.7(8)	3269.8(4)	837.4(8)	20.01(15)

Atom	x	y	z	U_{eq}
C11	316.6(8)	5398.8(4)	3397.7(8)	18.15(14)
C12	1442.1(7)	5236.5(4)	2611.9(7)	12.71(12)
C13	2542.3(7)	5871.3(4)	2841.8(7)	13.52(12)
C14	3122.5(7)	6009.2(4)	4300.5(7)	17.84(14)
C15	3159.8(9)	6666.7(5)	4880.7(10)	27.67(18)
C16	3728.2(7)	5784.9(4)	2063.3(7)	15.19(13)
C17	4118.2(9)	6541.8(4)	1586.6(9)	23.94(16)
C18	5268.7(10)	6488.5(6)	796.9(10)	33.7(2)
C19	6572.1(10)	6104.0(6)	1600.1(10)	36.2(2)
C20	6192.7(9)	5345.1(5)	2059.9(10)	30.58(19)
C21	5053.0(7)	5405.0(4)	2859.3(8)	19.63(14)

Table S11: Anisotropic Displacement Parameters (×10[4]) for **3ab.** The anisotropic displacement factor exponent takes the form: $-2\pi^2[h^2a^{*2} \times U_{11}+ ... +2hka^* \times b^* \times U_{12}]$

Atom	U_{11}	U_{22}	U_{33}	U_{23}	U_{13}	U_{12}
Fe1	12.95(5)	9.63(4)	11.83(5)	-0.53(3)	1.88(3)	-0.39(3)
O1	19.4(3)	17.6(3)	16.2(2)	-4.4(2)	-3.3(2)	4.4(2)
H1	81(10)	24(7)	46(8)	-31(7)	-10(7)	3(6)
C1	11.9(3)	11.0(3)	13.1(3)	-0.3(2)	1.6(2)	-0.5(2)
C2	16.8(3)	13.3(3)	13.0(3)	1.0(2)	-0.3(2)	0.4(2)
H2	55(7)	43(7)	14(5)	9(5)	21(5)	-10(5)
C3	18.4(3)	13.9(3)	22.8(4)	3.0(3)	-2.6(3)	1.7(3)
H3	59(8)	29(6)	41(7)	7(5)	-14(6)	29(5)
C4	16.2(3)	14.7(3)	31.5(4)	2.8(2)	6.0(3)	-4.5(3)
H4	48(8)	39(7)	88(10)	28(6)	29(7)	-11(6)
C5	17.4(3)	15.2(3)	20.2(4)	-2.2(2)	7.8(3)	-2.8(3)
H5	68(8)	42(7)	25(6)	-9(6)	25(6)	1(5)
C6	16.2(3)	19.0(4)	26.4(4)	-0.4(3)	-1.0(3)	-3.9(3)
H6	38(7)	21(6)	82(9)	19(5)	1(6)	-7(6)
C7	19.4(3)	27.3(4)	21.5(4)	-6.8(3)	6.9(3)	-3.1(3)
H7	49(8)	92(10)	31(7)	-8(7)	28(6)	-6(6)
C8	24.3(4)	15.5(3)	21.7(4)	-6.1(3)	2.1(3)	2.6(3)
H8	55(7)	35(7)	47(7)	3(6)	-1(6)	24(6)
C9	23.9(4)	14.8(3)	19.2(3)	-2.9(3)	2.8(3)	-4.2(3)
H9	53(7)	40(7)	48(7)	9(6)	20(6)	-22(6)
C10	23.6(4)	18.5(3)	15.2(3)	-3.8(3)	-1.5(3)	-0.3(3)
H10	72(9)	50(8)	20(6)	-2(6)	1(6)	20(5)
C11	14.4(3)	14.7(3)	25.8(4)	1.6(3)	5.5(3)	0.3(3)
H11a	41(7)	55(8)	27(7)	-6(6)	2(5)	-8(6)
H11b	29(6)	40(7)	73(9)	-15(5)	16(6)	-7(6)
H11c	47(7)	24(6)	73(9)	18(5)	21(6)	11(6)
C12	12.3(3)	11.2(3)	13.5(3)	-0.7(2)	0.4(2)	1.0(2)
C13	13.0(3)	10.5(3)	15.5(3)	0.1(2)	-0.1(2)	0.5(2)
H13	34(6)	19(5)	47(7)	10(5)	4(5)	7(5)
C14	15.9(3)	19.5(3)	17.0(3)	-0.6(3)	1.2(3)	-4.3(3)
H14	59(8)	28(6)	43(7)	13(5)	-5(6)	8(5)
C15	20.5(4)	29.7(5)	31.6(5)	-1.2(3)	2.9(3)	-17.1(4)
H15a	64(9)	31(7)	79(10)	22(6)	-7(7)	-4(7)
H15b	48(8)	91(10)	29(7)	-3(7)	-1(6)	-29(7)
C16	15.2(3)	14.3(3)	15.3(3)	-4.1(2)	1.7(2)	-0.9(2)
H16	44(7)	42(7)	20(6)	-16(5)	6(5)	-9(5)
C17	28.0(4)	18.9(4)	24.9(4)	-9.7(3)	5.7(3)	1.6(3)
H17a	59(8)	49(8)	44(7)	3(6)	-5(6)	24(6)
H17b	62(8)	27(6)	40(7)	-15(6)	17(6)	-11(5)
C18	39.1(5)	36.9(5)	28.2(5)	-23.1(4)	14.2(4)	-4.5(4)
H18a	90(10)	57(9)	62(9)	-39(8)	30(8)	3(7)
H18b	63(8)	68(9)	32(7)	-14(7)	8(6)	-11(7)
C19	25.0(4)	55.0(6)	32.4(5)	-20.1(4)	14.7(4)	-17.8(4)

Atom	*U11*	*U22*	*U33*	*U23*	*U13*	*U12*
H19a	51(8)	116(12)	70(9)	-35(8)	47(7)	-41(8)
H19b	65(9)	62(9)	48(8)	-26(7)	2(7)	-29(7)
C20	17.1(4)	41.6(5)	33.4(5)	-3.0(3)	6.4(3)	-17.1(4)
H20a	22(6)	77(9)	67(9)	8(6)	-2(6)	-3(7)
H20b	52(8)	65(9)	53(8)	-10(6)	11(6)	-35(7)
C21	13.5(3)	21.6(4)	22.6(4)	-0.9(3)	1.3(3)	-4.2(3)
H21a	39(7)	26(6)	59(8)	1(5)	7(6)	10(6)
H21b	30(6)	51(7)	28(6)	-11(5)	-4(5)	-16(5)

Table S12: Bond Lengths in Å for **3ab**.

Atom	Atom	Length/Å
Fe1	C1	2.0630(6)
Fe1	C2	2.0530(7)
Fe1	C3	2.0470(7)
Fe1	C4	2.0415(7)
Fe1	C5	2.0378(7)
Fe1	C6	2.0625(7)
Fe1	C7	2.0520(8)
Fe1	C8	2.0381(7)
Fe1	C9	2.0426(7)
Fe1	C10	2.0567(7)
O1	C12	1.4346(8)
C1	C2	1.4314(9)
C1	C5	1.4312(10)
C1	C12	1.5089(9)
C2	C3	1.4259(10)
C3	C4	1.4225(11)
C4	C5	1.4248(10)

Atom	Atom	Length/Å
C6	C7	1.4263(11)
C6	C10	1.4221(11)
C7	C8	1.4213(11)
C8	C9	1.4215(11)
C9	C10	1.4230(11)
C11	C12	1.5286(10)
C12	C13	1.5569(9)
C13	C14	1.5038(10)
C13	C16	1.5533(10)
C14	C15	1.3293(11)
C16	C17	1.5325(10)
C16	C21	1.5352(10)
C17	C18	1.5266(13)
C18	C19	1.5266(15)
C19	C20	1.5260(14)
C20	C21	1.5254(11)

Table S13: Bond Angles in ° for **3ab**.

Atom	Atom	Atom	Angle/°
C2	Fe1	C1	40.70(3)
C3	Fe1	C1	68.66(3)
C3	Fe1	C2	40.70(3)
C4	Fe1	C1	68.80(3)
C4	Fe1	C2	68.52(3)
C4	Fe1	C3	40.72(3)
C5	Fe1	C1	40.85(3)
C5	Fe1	C2	68.49(3)
C5	Fe1	C3	68.61(3)
C5	Fe1	C4	40.89(3)
C6	Fe1	C1	111.08(3)
C6	Fe1	C2	121.76(3)
C6	Fe1	C3	154.02(3)
C6	Fe1	C4	165.14(3)
C6	Fe1	C5	129.34(3)
C7	Fe1	C1	129.61(3)
C7	Fe1	C2	109.25(3)
C7	Fe1	C3	118.24(3)
C7	Fe1	C4	150.84(3)
C7	Fe1	C5	167.58(3)
C7	Fe1	C6	40.57(3)
C8	Fe1	C1	165.82(3)
C8	Fe1	C2	126.65(3)
C8	Fe1	C3	105.43(3)

Atom	Atom	Atom	Angle/°
C8	Fe1	C4	115.75(3)
C8	Fe1	C5	150.58(3)
C8	Fe1	C6	68.22(3)
C8	Fe1	C7	40.67(3)
C9	Fe1	C1	153.18(3)
C9	Fe1	C2	163.14(3)
C9	Fe1	C3	124.43(3)
C9	Fe1	C4	104.92(3)
C9	Fe1	C5	117.42(3)
C9	Fe1	C6	68.13(3)
C9	Fe1	C7	68.44(3)
C9	Fe1	C8	40.77(3)
C10	Fe1	C1	120.92(3)
C10	Fe1	C2	155.53(3)
C10	Fe1	C3	162.77(3)
C10	Fe1	C4	125.99(3)
C10	Fe1	C5	108.36(3)
C10	Fe1	C6	40.39(3)
C10	Fe1	C7	68.31(3)
C10	Fe1	C8	68.42(3)
C10	Fe1	C9	40.62(3)
C2	C1	Fe1	69.27(4)
C5	C1	Fe1	68.63(4)
C5	C1	C2	107.06(6)

Atom	Atom	Atom	Angle/˚
C12	C1	Fe1	128.40(4)
C12	C1	C2	127.30(6)
C12	C1	C5	125.63(6)
C1	C2	Fe1	70.03(4)
C3	C2	Fe1	69.42(4)
C3	C2	C1	108.43(6)
C2	C3	Fe1	69.87(4)
C4	C3	Fe1	69.43(4)
C4	C3	C2	108.04(6)
C3	C4	Fe1	69.85(4)
C5	C4	Fe1	69.42(4)
C5	C4	C3	107.90(6)
C1	C5	Fe1	70.52(4)
C4	C5	Fe1	69.70(4)
C4	C5	C1	108.57(7)
C7	C6	Fe1	69.32(4)
C10	C6	Fe1	69.58(4)
C10	C6	C7	108.17(7)
C6	C7	Fe1	70.11(4)
C8	C7	Fe1	69.14(4)
C8	C7	C6	107.72(7)
C7	C8	Fe1	70.19(4)
C9	C8	Fe1	69.78(4)
C9	C8	C7	108.20(7)

Atom	Atom	Atom	Angle/˚
C8	C9	Fe1	69.45(4)
C10	C9	Fe1	70.22(4)
C10	C9	C8	108.07(7)
C6	C10	Fe1	70.02(4)
C9	C10	Fe1	69.16(4)
C9	C10	C6	107.84(7)
C1	C12	O1	108.67(5)
C11	C12	O1	108.82(6)
C11	C12	C1	111.53(6)
C13	C12	O1	105.13(5)
C13	C12	C1	113.14(5)
C13	C12	C11	109.28(6)
C14	C13	C12	111.82(6)
C16	C13	C12	114.54(5)
C16	C13	C14	111.67(6)
C15	C14	C13	124.33(8)
C17	C16	C13	109.88(6)
C21	C16	C13	114.05(6)
C21	C16	C17	109.41(6)
C18	C17	C16	112.14(7)
C19	C18	C17	111.44(8)
C20	C19	C18	110.40(7)
C21	C20	C19	110.99(8)
C20	C21	C16	111.99(7)

Table S14: Torsion Angles in ˚ for **3ab**.

Atom	Atom	Atom	Atom	Angle/˚
Fe1	C1	C2	C3	58.98(4)
Fe1	C1	C5	C4	-59.53(4)
Fe1	C1	C12	O1	-53.96(6)
Fe1	C1	C12	C11	66.00(6)
Fe1	C1	C12	C13	-170.33(6)
Fe1	C2	C1	C5	-58.42(4)
Fe1	C2	C1	C12	123.13(4)
Fe1	C2	C3	C4	59.14(5)
Fe1	C3	C2	C1	-59.36(4)
Fe1	C3	C4	C5	59.20(4)
Fe1	C4	C3	C2	-59.42(4)
Fe1	C4	C5	C1	60.04(4)
Fe1	C5	C1	C2	58.83(4)
Fe1	C5	C1	C12	-122.69(4)
Fe1	C5	C4	C3	-59.47(5)
Fe1	C6	C7	C8	-59.13(5)
Fe1	C6	C10	C9	59.02(5)
Fe1	C7	C6	C10	58.92(5)
Fe1	C7	C8	C9	-59.66(5)
Fe1	C8	C7	C6	59.74(5)
Fe1	C8	C9	C10	-59.83(5)
Fe1	C9	C8	C7	59.92(5)
Fe1	C9	C10	C6	-59.57(5)
Fe1	C10	C6	C7	-58.76(5)
Fe1	C10	C9	C8	59.35(5)
O1	C12	C1	C2	-146.03(5)
O1	C12	C1	C5	35.80(7)
O1	C12	C13	C14	169.96(5)
O1	C12	C13	C16	-61.72(6)
C1	C2	C3	C4	-0.22(6)

Atom	Atom	Atom	Atom	Angle/˚
C1	C5	C4	C3	0.57(6)
C1	C12	C13	C14	-71.60(6)
C1	C12	C13	C16	56.73(6)
C2	C3	C4	C5	-0.22(6)
C6	C7	C8	C9	0.08(7)
C6	C10	C9	C8	-0.22(7)
C7	C8	C9	C10	0.09(7)
C11	C12	C13	C14	53.31(6)
C11	C12	C13	C16	-178.37(5)
C12	C13	C14	C15	-127.92(7)
C12	C13	C16	C17	143.00(6)
C12	C13	C16	C21	-93.74(6)
C13	C16	C17	C18	-179.23(6)
C13	C16	C21	C20	-179.05(6)
C16	C17	C18	C19	-55.93(8)
C16	C21	C20	C19	57.14(7)
C17	C18	C19	C20	55.67(8)
C18	C19	C20	C21	-56.20(8)

Table S15: Hydrogen Fractional Atomic Coordinates (×10^4) and Equivalent Isotropic Displacement Parameters (Å^2×10^3) for **3ab**. U_{eq} is defined as 1/3 of the trace of the orthogonalised U_{ij}.

Atom	x	y	z	U_{eq}
H1	359(14)	4835(6)	1013(12)	54(4)
H2	1756(11)	4237(5)	5005(9)	35(3)
H3	3138(11)	2985(5)	4832(10)	47(3)
H4	3801(11)	2951(6)	2386(12)	56(4)
H5	2850(11)	4206(6)	1118(10)	43(3)
H6	-1573(11)	4069(5)	1730(12)	49(3)
H7	-1031(11)	3176(7)	3878(10)	54(3)
H8	532(11)	2037(6)	3304(11)	47(3)
H9	975(11)	2255(6)	842(10)	45(3)
H10	-329(12)	3513(6)	-124(10)	48(3)
H11a	752(11)	5427(6)	4459(10)	42(3)
H11b	-487(10)	4982(6)	3219(12)	47(3)
H11c	-194(11)	5921(6)	3082(12)	47(3)
H13	1930(10)	6367(5)	2436(10)	34(3)
H14	3572(11)	5539(5)	4901(10)	46(3)
H15a	2730(12)	7152(6)	4325(12)	61(4)
H15b	3630(12)	6742(7)	5928(11)	57(4)
H16	3310(10)	5449(5)	1169(9)	36(3)
H17a	3183(12)	6815(6)	1002(11)	53(3)
H17b	4471(12)	6895(6)	2457(10)	42(3)
H18a	5501(13)	7047(7)	476(12)	67(4)
H18b	4879(12)	6176(7)	-122(11)	55(3)
H19a	7390(12)	6055(8)	1042(12)	74(4)
H19b	7008(13)	6430(7)	2481(12)	60(4)
H20a	7126(11)	5068(7)	2658(12)	57(4)
H20b	5844(11)	5000(7)	1186(11)	57(3)
H21a	4809(10)	4856(6)	3206(11)	42(3)
H21b	5477(10)	5724(6)	3752(10)	38(3)

Citations

CrysAlisPro (Rigaku, V1.171.41.99a, 2021)

CrysAlisPro (ROD), Rigaku Oxford Diffraction, Poland (?).

L.J. Bourhis and O.V. Dolomanov and R.J. Gildea and J.A.K. Howard and H. Puschmann, The Anatomy of a Comprehensive Constrained, Restrained, Refinement Program for the Modern Computing Environment - Olex2 Disected, *Acta Cryst. A*, (2015), **A71**, 59-71.

O.V. Dolomanov and L.J. Bourhis and R.J. Gildea and J.A.K. Howard and H. Puschmann, Olex2: A complete structure solution, refinement and analysis program, *J. Appl. Cryst.*, (2009), **42**, 339-341.

Sheldrick, G.M., ShelXT-Integrated space-group and crystal-structure determination, *Acta Cryst.*, (2015), **A71**, 3-8.

S12 References

1. Chanthamath, S.; Chua, H. W.; Kimura, S.; Shibatomi, K.; Iwasa, S., Highly Regio- and Stereoselective Synthesis of Alkylidenecyclopropanes via Ru(II)-Pheox Catalyzed Asymmetric Inter- and Intramolecular Cyclopropanation of Allenes. *Organic Letters* **2014,** *16* (12), 3408-3411.
2. Kuang, J.; Ma, S., An Efficient Synthesis of Terminal Allenes from Terminal 1-Alkynes. *The Journal of Organic Chemistry* **2009,** *74* (4), 1763-1765.
3. Chausset-Boissarie, L.; Ghozati, K.; LaBine, E.; Chen, J. L. Y.; Aggarwal, V. K.; Crudden, C. M., Enantiospecific, Regioselective Cross-Coupling Reactions of Secondary Allylic Boronic Esters. *Chemistry – A European Journal* **2013,** *19* (52), 17698-17701.
4. Gonzalez, A. Z.; Román, J. G.; Gonzalez, E.; Martinez, J.; Medina, J. R.; Matos, K.; Soderquist, J. A., 9-Borabicyclo[3.3.2]decanes and the Asymmetric Hydroboration of 1,1-Disubstituted Alkenes. *Journal of the American Chemical Society* **2008,** *130* (29), 9218-9219.
5. Tsai, E. Y.; Liu, R. Y.; Yang, Y.; Buchwald, S. L., A Regio- and Enantioselective CuH-Catalyzed Ketone Allylation with Terminal Allenes. *Journal of the American Chemical Society* **2018,** *140* (6), 2007-2011.
6. Rice, L. E.; Boston, M. C.; Finklea, H. O.; Suder, B. J.; Frazier, J. O.; Hudlicky, T., Regioselectivity in the Reformatskii reaction of 4-bromocrotonate. Role of the catalyst and the solvent in the normal vs. abnormal modes of addition to carbonyl substrates. *The Journal of Organic Chemistry* **1984,** *49* (10), 1845-1848.
7. Bartolo, N. D.; Woerpel, K. A., Mechanistic Insight into Additions of Allylic Grignard Reagents to Carbonyl Compounds. *The Journal of Organic Chemistry* **2018,** *83* (17), 10197-10206.
8. Nagashima, Y.; Sasaki, K.; Suto, T.; Sato, T.; Chida, N., Stereodivergent Hydroboration of Allenes. *Chemistry – An Asian Journal* **2018,** *13* (8), 1024-1028.
