# Supplementary material for: Boron-Catalyzed, Diastereo- and Enantioselective Allylation of Ketones with Allenes
Source: ACS Catal. 2022 Aug 22;12(17):10887–93. doi: 10.1021/acscatal.2c03158 (PMC9442582; doi:10.1021/acscatal.2c03158)
Supplement: Supplementary file 2 — cs2c03158_si_002.zip [file cs2c03158_si_002.zip › SE22001.docx]

**SE22001**


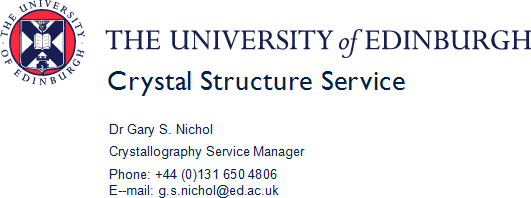


Submitted by: **Kieran Nicholson**

Solved by: **Gary S Nichol**

Sample ID: **KN07-120**

***R_1_*=4.92%**

Compound KN07-120 was provided as crystals suitable for single crystal X-ray diffraction, yielding structure SE22001.

Crystal Data and Experimental


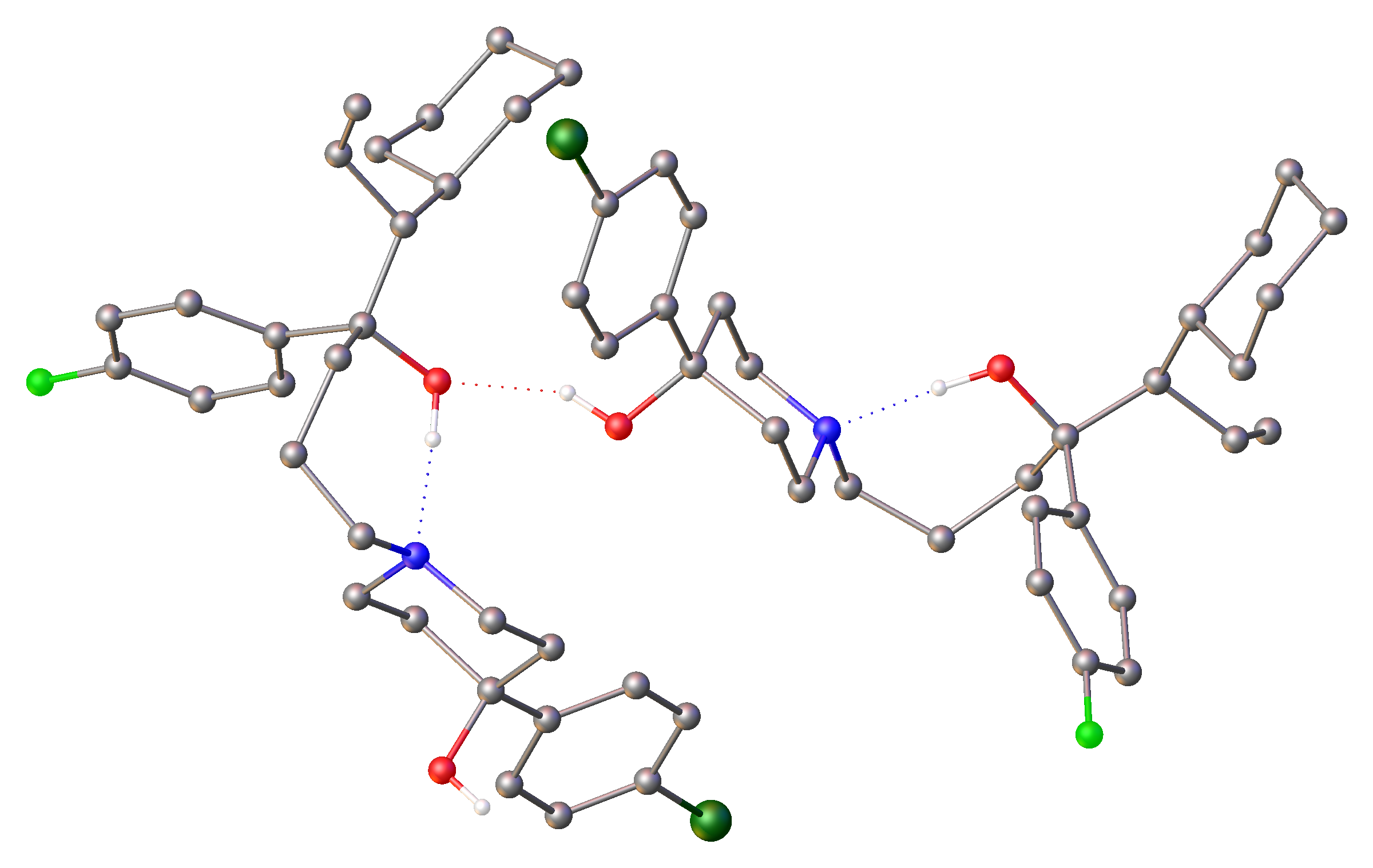


**Experimental.** Single colourless block-shaped crystals of **SE22001** recrystallised from a mixture of dichloromethane and petroleum ether by slow evaporation. A suitable crystal with dimensions 0.28 × 0.23 × 0.21 mm^3^ was selected and mounted on a MITIGEN holder in Paratone oil on a Rigaku Oxford Diffraction XCalibur diffractometer. The crystal was kept at a steady *T* = 120.01(12) K during data collection. The structure was solved with the **ShelXT** 2018/2 (Sheldrick, 2018) solution program using dual methods and by using **Olex2** 1.5-beta (Dolomanov et al., 2009) as the graphical interface. The model was refined with **olex2.refine** 1.5-beta (Bourhis et al., 2015) using full matrix least squares minimisation on ***F*^2^**.

**Crystal Data.**  C_30_H_39_ClFNO_2_, *M_r_* = 500.100, monoclinic, *P*2_1_/*c* (No. 14), a = 26.4305(9) Å, b = 10.5769(3) Å, c = 19.9763(8) Å, *β* = 106.275(4)^°^, *α* = *γ* = 90^°^, *V* = 5360.6(3) Å^3^, *T* = 120.01(12) K, *Z* = 8, *Z'* = 2, *μ*(Mo K*_α_*) = 0.177, 64971 reflections measured, 15011 unique (R_int_ = 0.0500) which were used in all calculations. The final *wR_2_* was 0.0784 (all data) and *R_1_* was 0.0492 (I≥2 *σ*(I)).

| **Compound** | **SE22001** |
| --- | --- |
|  |  |
| Formula | C_30_H_39_ClFNO_2_ |
| *D_calc._*/ g cm^-3^ | 1.239 |
| *μ*/mm^-1^ | 0.177 |
| Formula Weight | 500.100 |
| Colour | colourless |
| Shape | block-shaped |
| Size/mm^3^ | 0.28×0.23×0.21 |
| *T*/K | 120.01(12) |
| Crystal System | monoclinic |
| Space Group | *P*2_1_/*c* |
| *a*/Å | 26.4305(9) |
| *b*/Å | 10.5769(3) |
| *c*/Å | 19.9763(8) |
| *α*/^°^ | 90 |
| *β*/^°^ | 106.275(4) |
| *γ*/^°^ | 90 |
| V/Å^3^ | 5360.6(3) |
| *Z* | 8 |
| *Z'* | 2 |
| Wavelength/Å | 0.71073 |
| Radiation type | Mo K*_α_* |
| *Θ_min_*/^°^ | 3.34 |
| *Θ_max_*/^°^ | 29.57 |
| Measured Refl's. | 64971 |
| Indep't Refl's | 15011 |
| Refl's I≥2 *σ*(I) | 11607 |
| *R*_int_ | 0.0500 |
| Parameters | 1333 |
| Restraints | 6 |
| Largest Peak | 0.4704 |
| Deepest Hole | -0.5670 |
| GooF | 1.0921 |
| *wR_2_* (all data) | 0.0784 |
| *wR_2_* | 0.0719 |
| *R_1_* (all data) | 0.0724 |
| *R_1_* | 0.0492 |

**Structure Quality Indicators**

| **Reflections:** | 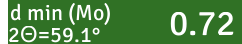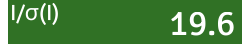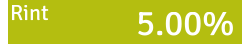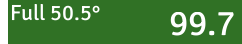 |
| --- | --- |
| **Refinement:** | 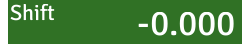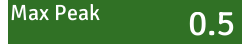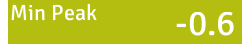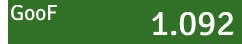 |

A colourless block-shaped-shaped crystal with dimensions 0.28 × 0.23 × 0.21 mm^3^ was mounted on a MITIGEN holder in Paratone oil. Data were collected using a Rigaku Oxford Diffraction XCalibur diffractometer equipped with an Oxford Cryosystems Cryostream 700+ low-temperature device operating at *T* = 120.01(12) K.

Data were measured using *ω* scans with Mo K*_α_* radiation. The diffraction pattern was indexed and the total number of runs and images was based on the strategy calculation from the program CrysAlisPro 1.171.41.99a (Rigaku OD, 2021). The maximum resolution that was achieved was *Θ* = 29.57^°^ (0.72 Å).

The unit cell was refined using CrysAlisPro 1.171.41.99a (Rigaku OD, 2021) on 12631 reflections, 19% of the observed reflections.

Data reduction, scaling and absorption corrections were performed using CrysAlisPro 1.171.41.99a (Rigaku OD, 2021). The final completeness is 99.74 % out to 29.57^°^ in *Θ*. A multi-scan absorption correction was performed using CrysAlisPro 1.171.41.99a (Rigaku Oxford Diffraction, 2021) Empirical absorption correction using spherical harmonics, implemented in SCALE3 ABSPACK scaling algorithm.. The absorption coefficient *μ* of this material is 0.177 mm^-1^ at this wavelength (*λ* = 0.71073Å) and the minimum and maximum transmissions are 0.981 and 1.000.

The structure was solved and the space group *P*2_1_/*c* (# 14) determined by the ShelXT 2018/2 (Sheldrick, 2018) structure solution program using using dual methods and refined by full matrix least squares minimisation on ***F*^2^** using version of **olex2.refine** 1.5-beta (Bourhis et al., 2015). All non-hydrogen atoms were refined anisotropically. Hydrogen atom positions were calculated geometrically and refined using the riding model.

*_refine_special_details*: H atoms were all identified from a difference map. The NoSpherA2 routine of Olex2 was used for refinement and details are given elsewhere.#===============================================================================#>>> The Following Improvement and Query ALERTS were generated - (Acta-Mode) <<<#=============================================================================== Format: alert-number_ALERT_alert-type_alert-level text417_ALERT_2_B Short Inter D-H..H-D H1 ..H52 . 2.05 Ang. x,y,z = 1_555 Check417_ALERT_2_B Short Inter D-H..H-D H2 ..H51 . 2.07 Ang. x,-1+y,z = 1_545 CheckH atoms identified from a difference map and refined using NoSpherA2910_ALERT_3_B Missing # of FCF Reflection(s) Below Theta(Min). 24 Note#===============================================================================351_ALERT_3_C Long C-H (X0.96,N1.08A) C5 - H5 . 1.11 Ang.351_ALERT_3_C Long C-H (X0.96,N1.08A) C12 - H12B . 1.11 Ang.351_ALERT_3_C Long C-H (X0.96,N1.08A) C27 - H27B . 1.11 Ang.411_ALERT_2_C Short Inter H...H Contact H3B ..H24 . 2.13 Ang. -x,-y,-z = 3_555 CheckH atoms identified from a difference map and refined using NoSpherA2906_ALERT_3_C Large K Value in the Analysis of Variance ...... 9.347 Check906_ALERT_3_C Large K Value in the Analysis of Variance ...... 2.068 Check#===============================================================================

The value of Z' is 2. This means that there are two independent molecules in the asymmetric unit.


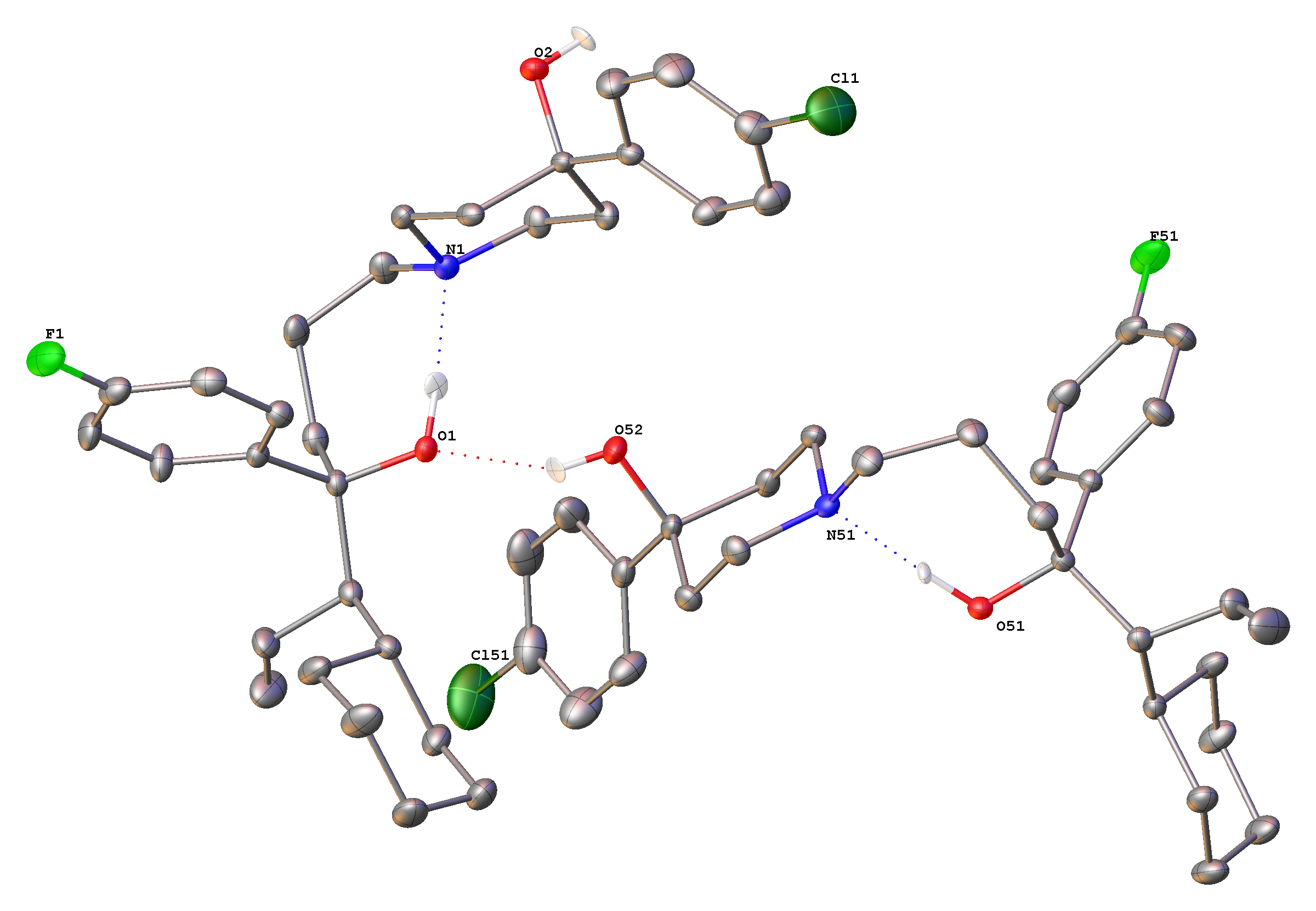


**Figure 1**: The asymmetric unit of SE22001. Displacment ellipsoids are at the 50% probability level; C-bound H atoms are not shown.

**Reflection Statistics**

| Total reflections (after filtering) | 66728 | Unique reflections | 15011 |
| --- | --- | --- | --- |
| Completeness | 1.0 | Mean I/*σ* | 13.17 |
| hkl_max_ collected | (36, 14, 25) | hkl_min_ collected | (-36, -14, -27) |
| hkl_max_ used | (35, 14, 27) | hkl_min_ used | (-36, 0, 0) |
| Lim d_max_ collected | 100.0 | Lim d_min_ collected | 0.36 |
| d_max_ used | 6.1 | d_min_ used | 0.72 |
| Friedel pairs | 4598 | Friedel pairs merged | 1 |
| Inconsistent equivalents | 5 | R_int_ | 0.0504 |
| R_sigma_ | 0.0511 | Intensity transformed | 0 |
| Omitted reflections | 0 | Omitted by user (OMIT hkl) | 5 |
| Multiplicity | (12118, 12967, 6073, 1970, 381, 112) | Maximum multiplicity | 20 |
| Removed systematic absences | 1757 | Filtered off (Shel/OMIT) | 0 |

**Table 1**: Fractional Atomic Coordinates (×10^4^) and Equivalent Isotropic Displacement Parameters (Å^2^×10^3^) for **SE22001**. *U_eq_* is defined as 1/3 of the trace of the orthogonalised *U_ij_*.

| **Atom** | **x** | **y** | **z** | ***U_eq_*** |
| --- | --- | --- | --- | --- |
| Cl1 | 5332.86(13) | -310.3(4) | 2951.5(2) | 46.22(11) |
| F1 | 1377.9(3) | -2052.6(7) | -1568.0(4) | 31.35(18) |
| O1 | 1689.8(3) | 1115.8(8) | 1169.4(4) | 15.06(17) |
| O2 | 2978.1(3) | -2747.1(8) | 2633.0(5) | 20.51(19) |
| N1 | 1865.9(3) | -969.6(8) | 1995.9(5) | 14.88(19) |
| C1 | 1322.6(5) | -1374.4(13) | 1927.4(7) | 20.4(3) |
| C2 | 931.4(5) | -1123.5(12) | 1219.8(7) | 19.9(3) |
| C3 | 811.4(5) | 269.2(12) | 1028.4(7) | 18.6(2) |
| C4 | 1183.4(4) | 978.3(10) | 683.0(6) | 13.8(2) |
| C5 | 969.1(4) | 2357.0(11) | 519.9(6) | 16.1(2) |
| C6 | 415.0(5) | 2435.6(12) | 48.6(7) | 23.1(3) |
| C7 | 16.6(5) | 2953.2(13) | 231.3(9) | 33.1(3) |
| C8 | 2162.6(5) | -992.3(13) | 2739.8(6) | 18.6(2) |
| C9 | 2736.5(5) | -616.7(11) | 2863.5(6) | 16.7(2) |
| C10 | 3010.9(4) | -1458.9(10) | 2449.5(6) | 13.6(2) |
| C11 | 2691.7(5) | -1359.0(11) | 1681.5(6) | 15.7(2) |
| C12 | 2124.2(5) | -1774.0(11) | 1587.5(6) | 16.4(2) |
| C13 | 3590.4(4) | -1116.5(10) | 2559.8(6) | 15.3(2) |
| C14 | 3805.2(5) | 37.0(11) | 2827.4(7) | 21.1(3) |
| C15 | 4340.7(5) | 293.7(13) | 2950.4(7) | 26.6(3) |
| C16 | 4663.2(5) | -617.2(12) | 2795.8(7) | 25.9(3) |
| C17 | 4462.0(5) | -1771.2(13) | 2516.8(7) | 29.4(3) |
| C18 | 3928.4(5) | -2013.0(12) | 2403.5(7) | 24.1(3) |
| C19 | 1234.6(4) | 224.7(10) | 47.9(6) | 14.0(2) |
| C20 | 1728.0(4) | -81.9(11) | -29.7(6) | 16.3(2) |
| C21 | 1779.4(5) | -848.4(11) | -574.4(6) | 20.8(3) |
| C22 | 1330.1(5) | -1298.5(11) | -1045.7(6) | 21.7(3) |
| C23 | 834.6(5) | -1015.2(12) | -993.3(7) | 24.6(3) |
| C24 | 791.6(5) | -258.4(12) | -444.5(6) | 21.6(3) |
| C25 | 1340.0(4) | 3276.1(10) | 278.4(6) | 15.9(2) |
| C26 | 1339.9(6) | 3170.0(13) | -486.5(7) | 26.0(3) |
| C27 | 1733.3(7) | 4092.9(13) | -645.9(8) | 31.4(3) |
| C28 | 1606.6(7) | 5453.7(13) | -501.7(7) | 30.7(3) |
| C29 | 1583.1(6) | 5601.5(13) | 245.8(7) | 26.4(3) |
| C30 | 1209.1(5) | 4644.3(12) | 424.2(7) | 22.7(3) |
| Cl51 | -242.43(14) | 4084.2(5) | 2263.1(3) | 58.40(14) |
| F51 | 3737.2(3) | 2925.2(7) | 6654.0(4) | 37.3(2) |
| O51 | 3329.0(3) | 6243.9(8) | 3951.3(4) | 14.64(17) |
| O52 | 2177.5(3) | 2202.9(8) | 2417.7(5) | 23.7(2) |
| N51 | 3219.5(3) | 4166.3(8) | 3123.1(5) | 14.61(19) |
| C51 | 3775.0(5) | 3918.4(13) | 3171.2(7) | 20.6(3) |
| C52 | 4164.8(5) | 4191.9(12) | 3880.1(7) | 19.7(3) |
| C53 | 4232.5(5) | 5589.7(11) | 4088.3(7) | 18.4(2) |
| C54 | 3837.2(4) | 6172.3(10) | 4441.2(6) | 13.3(2) |
| C55 | 4008.4(4) | 7580.1(10) | 4627.2(6) | 15.0(2) |
| C56 | 4547.2(5) | 7729.6(12) | 5132.4(7) | 22.8(3) |
| C57 | 4955.7(6) | 8242.5(14) | 4972.3(10) | 35.0(4) |
| C58 | 3002.9(5) | 3216.8(11) | 3503.8(6) | 16.9(2) |
| C59 | 2420.3(5) | 3451.3(12) | 3426.9(6) | 17.6(2) |
| C60 | 2100.3(4) | 3430.2(10) | 2658.6(6) | 16.2(2) |
| C61 | 2337.2(5) | 4424.6(11) | 2279.4(6) | 17.9(2) |
| C62 | 2919.3(5) | 4183.6(13) | 2380.8(6) | 18.9(2) |
| C63 | 1512.1(4) | 3636.6(11) | 2569.4(6) | 18.6(2) |
| C64 | 1199.7(5) | 2614.3(14) | 2641.5(7) | 31.0(3) |
| C65 | 662.1(6) | 2747.9(15) | 2554.8(8) | 36.4(4) |
| C66 | 431.6(5) | 3921.0(15) | 2384.1(7) | 33.6(3) |
| C67 | 729.5(6) | 4950.8(15) | 2309.8(8) | 36.0(3) |
| C68 | 1268.9(5) | 4800.5(13) | 2404.6(7) | 27.6(3) |
| C69 | 3810.1(4) | 5350.2(10) | 5062.7(5) | 13.4(2) |
| C70 | 4266.8(5) | 4941.4(12) | 5557.8(6) | 21.7(3) |
| C71 | 4248.2(5) | 4134.5(12) | 6099.5(7) | 27.0(3) |
| C72 | 3761.4(5) | 3724.6(11) | 6140.4(6) | 24.6(3) |
| C73 | 3300.1(5) | 4100.2(11) | 5664.0(6) | 22.3(3) |
| C74 | 3328.2(5) | 4914.7(11) | 5128.8(6) | 16.7(2) |
| C75 | 3596.9(4) | 8410.7(10) | 4842.4(6) | 14.8(2) |
| C76 | 3712.2(5) | 9813.6(11) | 4755.9(7) | 20.5(3) |
| C77 | 3290.9(6) | 10675.2(13) | 4895.7(7) | 27.1(3) |
| C78 | 3217.9(6) | 10431.3(12) | 5614.9(7) | 26.7(3) |
| C79 | 3116.4(6) | 9040.6(12) | 5714.6(8) | 25.7(3) |
| C80 | 3550.8(5) | 8205.8(12) | 5583.2(6) | 20.5(3) |

**Table 2**: Anisotropic Displacement Parameters (×10^4^) for **SE22001**. The anisotropic displacement factor exponent takes the form: *-2π^2^[h^2^a*^2^ × U_11_+ ... +2hka* × b* × U_12_]*

| **Atom** | ***U_11_*** | ***U_22_*** | ***U_33_*** | ***U_23_*** | ***U_13_*** | ***U_12_*** |
| --- | --- | --- | --- | --- | --- | --- |
| Cl1 | 19.93(17) | 55.0(3) | 65.4(3) | 1.23(16) | 14.74(18) | 2.2(2) |
| F1 | 52.1(5) | 24.3(4) | 20.2(4) | 5.2(3) | 14.4(4) | -3.7(3) |
| O1 | 15.3(4) | 13.4(4) | 14.6(4) | -2.4(3) | 1.1(3) | -0.9(3) |
| H1 | 19(9) | 36(12) | 37(12) | -17(9) | 10(9) | 3(10) |
| O2 | 26.3(5) | 12.9(4) | 18.5(4) | 0.4(3) | -0.0(4) | 3.2(4) |
| H2 | 68(13) | 45(12) | 17(10) | 9(9) | -14(10) | 12(9) |
| N1 | 18.7(5) | 12.6(5) | 13.0(5) | -2.4(4) | 3.8(4) | 1.5(4) |
| C1 | 22.4(6) | 21.9(7) | 16.8(6) | -5.9(5) | 5.5(5) | 3.4(5) |
| H1a | 28(9) | 78(13) | 38(10) | -6(8) | 17(8) | -14(9) |
| H1b | 37(9) | 37(10) | 70(13) | -5(8) | 7(9) | 26(9) |
| C2 | 17.7(6) | 21.8(6) | 18.9(6) | -8.0(5) | 3.0(5) | -0.3(5) |
| H2a | 35(9) | 66(12) | 42(10) | -22(8) | 5(8) | 12(9) |
| H2b | 52(10) | 36(9) | 24(9) | 1(8) | 19(8) | -15(7) |
| C3 | 14.7(6) | 23.5(6) | 16.9(6) | -2.4(5) | 3.5(5) | -2.0(5) |
| H3a | 57(10) | 47(10) | 30(10) | -5(8) | 23(8) | -6(8) |
| H3b | 9(7) | 46(10) | 51(10) | -2(7) | 4(7) | 18(8) |
| C4 | 12.8(5) | 14.8(5) | 12.7(5) | -0.6(4) | 1.8(4) | -1.8(4) |
| C5 | 15.5(5) | 16.8(6) | 14.6(6) | 1.6(4) | 1.9(5) | -2.6(5) |
| H5 | 34(8) | 28(8) | 24(9) | -6(6) | 12(7) | -10(7) |
| C6 | 17.0(6) | 22.4(6) | 25.1(7) | 3.2(5) | -2.0(5) | -3.4(5) |
| H6 | 36(9) | 66(12) | 35(10) | 3(8) | -5(8) | -11(9) |
| C7 | 16.2(7) | 28.7(7) | 50.3(10) | 3.2(6) | 2.6(7) | -6.8(7) |
| H7a | 61(11) | 59(12) | 65(13) | 4(9) | 26(10) | -39(11) |
| H7b | 16(8) | 67(12) | 99(15) | 3(8) | -19(9) | -2(11) |
| C8 | 20.8(6) | 23.0(7) | 12.3(5) | -1.9(5) | 5.2(5) | -0.3(5) |
| H8a | 42(9) | 26(9) | 33(9) | -15(7) | 9(7) | 17(7) |
| H8b | 42(10) | 69(12) | 40(10) | -9(9) | 20(8) | -13(9) |
| C9 | 19.9(6) | 17.3(6) | 12.0(6) | 0.1(5) | 3.1(5) | -2.4(5) |
| H9a | 52(9) | 13(8) | 25(9) | 4(7) | 20(7) | 5(7) |
| H9b | 35(9) | 72(12) | 22(9) | -21(8) | 5(7) | 0(8) |
| C10 | 18.6(6) | 10.1(5) | 10.7(5) | 1.3(4) | 2.0(4) | 0.6(4) |
| C11 | 23.1(6) | 12.9(6) | 11.6(5) | 3.5(5) | 5.7(5) | 0.6(4) |
| H11a | 66(11) | 40(10) | 38(10) | 15(8) | 22(9) | -10(8) |
| H11b | 44(9) | 9(8) | 50(10) | 4(7) | 14(8) | 15(7) |
| C12 | 22.6(6) | 12.7(6) | 11.8(5) | -0.9(5) | 1.2(5) | 0.4(4) |
| H12a | 31(8) | 42(9) | 19(9) | 1(7) | -1(7) | -11(7) |
| H12b | 62(10) | 6(8) | 50(11) | -15(7) | 11(8) | 6(7) |
| C13 | 19.4(6) | 13.5(5) | 12.7(5) | 3.0(4) | 4.3(4) | 1.4(4) |
| C14 | 21.0(6) | 15.8(6) | 28.9(7) | 1.4(5) | 11.2(5) | -1.5(5) |
| H14 | 47(10) | 22(9) | 90(14) | -3(7) | 39(9) | -28(9) |
| C15 | 22.8(7) | 22.6(7) | 35.7(8) | -2.7(5) | 10.4(6) | -1.2(6) |
| H15 | 57(10) | 31(10) | 71(13) | -27(8) | 24(9) | -23(9) |
| C16 | 20.4(6) | 30.7(7) | 27.3(7) | 3.7(5) | 8.0(5) | 3.9(6) |
| C17 | 24.5(7) | 29.3(7) | 36.0(8) | 11.0(6) | 11.1(6) | -1.2(6) |
| H17 | 62(11) | 51(11) | 101(16) | 37(9) | 47(11) | 0(10) |
| C18 | 24.3(7) | 20.1(7) | 27.5(7) | 5.2(5) | 6.8(5) | -3.6(5) |
| H18 | 57(11) | 32(10) | 73(13) | -20(8) | 21(9) | -26(9) |
| C19 | 14.4(5) | 12.7(5) | 13.8(5) | -2.0(4) | 2.3(4) | -0.6(4) |
| C20 | 15.4(6) | 16.2(6) | 17.4(6) | -1.1(4) | 4.5(5) | 0.8(4) |
| H20 | 23(8) | 57(10) | 27(9) | -1(7) | 3(7) | -19(8) |
| C21 | 24.4(7) | 20.4(6) | 21.2(6) | 4.3(5) | 12.5(5) | 2.4(5) |
| H21 | 54(10) | 60(11) | 65(12) | 8(9) | 45(9) | -12(9) |
| C22 | 33.9(7) | 16.2(6) | 16.7(6) | 1.7(5) | 9.9(5) | -1.1(5) |
| C23 | 27.0(7) | 25.8(7) | 18.5(6) | -4.7(5) | 2.4(5) | -9.6(5) |
| H23 | 60(11) | 78(13) | 44(11) | -15(10) | -10(9) | -43(10) |
| C24 | 16.0(6) | 27.6(7) | 20.1(6) | -3.8(5) | 3.2(5) | -9.0(5) |
| H24 | 28(9) | 56(11) | 55(11) | -11(8) | 12(8) | -22(9) |
| C25 | 16.9(6) | 15.3(5) | 14.2(5) | 3.0(4) | 1.9(5) | 0.7(4) |
| H25 | 13(4) | 44(9) | 34(8) | 5(3) | -1(3) | 9(6) |
| C26 | 45.5(9) | 15.5(6) | 16.9(6) | 0.3(6) | 8.5(6) | -0.1(5) |
| H26a | 55(11) | 47(11) | 28(10) | -8(9) | -12(8) | 8(8) |
| H26b | 127(16) | 29(10) | 68(13) | -10(10) | 60(12) | -2(9) |
| C27 | 53.7(10) | 22.4(7) | 23.0(7) | -1.8(7) | 18.6(7) | 4.2(6) |
| H27a | 145(17) | 49(12) | 34(11) | -23(11) | 43(12) | -6(9) |
| H27b | 44(11) | 66(13) | 60(13) | -1(9) | 19(10) | 11(10) |
| C28 | 46.8(9) | 17.4(7) | 24.6(7) | -3.6(6) | 4.4(7) | 5.3(6) |
| H28a | 61(11) | 63(12) | 38(11) | 15(9) | -10(9) | 10(9) |
| H28b | 76(12) | 49(11) | 41(11) | -23(10) | 32(10) | -2(9) |
| C29 | 33.7(8) | 16.9(7) | 25.7(7) | -4.0(6) | 3.5(6) | -2.5(5) |
| H29a | 35(10) | 71(13) | 44(11) | -17(9) | -7(9) | 14(9) |
| H29b | 85(13) | 12(9) | 80(14) | 0(8) | 29(11) | 5(9) |
| C30 | 23.8(7) | 18.2(6) | 24.4(7) | 0.6(5) | 4.1(6) | -6.0(5) |
| H30a | 83(13) | 58(12) | 31(10) | 4(9) | 35(10) | -12(9) |
| H30b | 34(9) | 30(9) | 64(12) | 6(7) | 11(9) | -4(8) |
| Cl51 | 25.90(19) | 79.9(3) | 74.4(3) | -8.09(19) | 22.3(2) | -25.4(3) |
| F51 | 70.6(6) | 21.1(4) | 20.4(4) | -7.8(4) | 12.7(4) | 4.1(3) |
| O51 | 16.2(4) | 12.1(4) | 14.3(4) | 1.9(3) | 1.9(3) | 0.2(3) |
| H51 | 20(9) | 26(11) | 22(11) | 4(8) | -9(8) | -7(9) |
| O52 | 28.1(5) | 14.2(4) | 22.0(5) | 0.3(4) | -4.0(4) | -4.9(4) |
| H52 | 72(14) | 38(11) | 3(9) | 9(10) | -8(9) | -3(8) |
| N51 | 19.4(5) | 11.5(5) | 12.7(4) | 1.3(4) | 4.1(4) | -1.3(4) |
| C51 | 23.6(6) | 20.5(7) | 19.0(6) | 4.4(5) | 8.0(5) | -3.2(5) |
| H51a | 46(10) | 37(10) | 53(11) | 13(8) | 5(8) | -22(9) |
| H51b | 33(9) | 59(11) | 36(10) | -3(8) | 11(8) | 13(9) |
| C52 | 16.9(6) | 20.9(6) | 20.7(6) | 6.6(5) | 4.2(5) | -1.0(5) |
| H52a | 47(9) | 43(10) | 27(9) | -9(8) | 15(8) | 9(8) |
| H52b | 18(8) | 46(10) | 67(12) | 17(7) | 8(8) | -13(9) |
| C53 | 16.5(6) | 19.7(6) | 19.6(6) | 2.0(5) | 5.9(5) | 0.2(5) |
| H53a | 52(10) | 40(10) | 38(10) | 6(8) | 21(8) | 17(8) |
| H53b | 25(8) | 43(10) | 35(10) | 9(7) | 1(7) | -9(8) |
| C54 | 12.4(5) | 13.1(5) | 13.1(5) | 0.8(4) | 1.7(4) | 0.8(4) |
| C55 | 15.6(5) | 14.5(5) | 14.3(6) | -1.3(4) | 3.3(5) | 1.6(4) |
| H55 | 32(8) | 47(10) | 10(8) | 9(7) | 9(7) | 5(7) |
| C56 | 16.9(6) | 21.8(6) | 25.9(7) | -3.9(5) | -0.4(5) | 1.6(5) |
| H56 | 71(12) | 74(13) | 19(10) | -8(9) | -4(9) | 8(9) |
| C57 | 18.2(7) | 30.3(8) | 52.8(10) | -6.0(6) | 3.8(7) | 7.1(7) |
| H57a | 54(11) | 87(15) | 62(13) | -3(10) | 8(10) | 35(12) |
| H57b | 7(8) | 72(13) | 103(15) | -19(8) | -9(9) | 5(11) |
| C58 | 24.2(6) | 12.0(6) | 12.3(6) | 0.0(5) | 1.6(5) | 1.1(5) |
| H58a | 42(9) | 45(10) | 14(8) | 1(7) | -2(7) | 1(7) |
| H58b | 44(9) | 24(9) | 39(10) | 12(7) | 5(8) | -6(7) |
| C59 | 24.4(6) | 15.3(6) | 11.9(5) | -5.3(5) | 3.3(5) | 0.8(5) |
| H59a | 38(9) | 32(9) | 42(10) | -1(7) | 4(8) | -25(8) |
| H59b | 42(9) | 46(10) | 48(11) | -22(8) | 19(8) | 23(8) |
| C60 | 21.4(6) | 12.9(5) | 11.5(5) | -2.5(4) | 0.3(5) | -1.5(4) |
| C61 | 22.6(6) | 17.0(6) | 12.5(6) | 0.8(5) | 2.6(5) | 2.2(5) |
| H61a | 49(10) | 26(9) | 52(11) | 7(8) | 4(8) | -7(8) |
| H61b | 38(9) | 58(11) | 12(8) | 3(7) | -12(7) | 5(7) |
| C62 | 23.3(6) | 20.8(6) | 12.7(6) | 1.0(5) | 5.5(5) | 1.0(5) |
| H62a | 44(10) | 66(12) | 26(9) | 11(8) | 8(8) | 16(8) |
| H62b | 56(10) | 34(10) | 31(9) | 2(8) | 19(8) | -21(8) |
| C63 | 22.4(6) | 18.6(6) | 13.4(5) | -4.7(5) | 2.7(5) | -2.5(4) |
| C64 | 27.8(7) | 28.2(8) | 32.8(8) | -9.3(6) | 1.7(6) | 7.2(6) |
| H64 | 58(12) | 44(12) | 101(16) | 0(9) | 10(11) | 32(11) |
| C65 | 30.2(8) | 43.1(9) | 35.6(8) | -16.5(7) | 8.9(7) | 0.6(7) |
| H65 | 57(11) | 54(12) | 128(18) | -28(9) | 35(12) | 38(12) |
| C66 | 26.3(7) | 45.8(9) | 30.9(7) | -6.9(6) | 11.5(6) | -13.3(7) |
| C67 | 28.2(8) | 31.8(8) | 51.2(9) | 2.5(6) | 16.4(7) | -10.2(7) |
| H67 | 46(11) | 39(12) | 160(20) | 7(9) | 51(12) | -17(12) |
| C68 | 25.6(7) | 20.1(7) | 40.0(8) | -0.9(5) | 13.8(6) | -6.5(6) |
| H68 | 43(10) | 20(10) | 146(19) | 4(8) | 42(11) | 8(10) |
| C69 | 14.4(5) | 12.1(5) | 12.7(5) | 1.4(4) | 2.1(4) | 0.2(4) |
| C70 | 19.0(6) | 23.1(6) | 19.9(6) | 2.1(5) | 0.3(5) | 4.8(5) |
| H70 | 22(8) | 56(11) | 58(11) | -9(8) | 3(8) | 18(9) |
| C71 | 34.0(8) | 22.7(7) | 18.2(6) | 5.0(6) | -2.6(6) | 5.4(5) |
| H71 | 53(11) | 70(13) | 50(12) | 15(9) | -13(9) | 31(10) |
| C72 | 44.4(8) | 14.2(6) | 14.8(6) | -1.9(5) | 7.9(6) | 0.2(5) |
| C73 | 32.6(7) | 16.7(6) | 21.3(6) | -6.5(5) | 13.8(6) | -2.1(5) |
| H73 | 61(11) | 56(11) | 61(12) | -21(9) | 33(9) | 14(9) |
| C74 | 18.9(6) | 15.2(6) | 16.2(6) | -0.6(5) | 5.4(5) | -0.6(4) |
| H74 | 29(9) | 53(11) | 46(10) | -1(7) | -5(8) | 24(8) |
| C75 | 16.0(6) | 13.8(5) | 13.0(5) | -1.3(4) | 1.6(5) | -0.5(4) |
| H75 | 18(7) | 32(9) | 31(9) | 8(6) | 7(7) | -13(7) |
| C76 | 28.2(7) | 15.0(6) | 18.3(6) | -0.9(5) | 6.3(5) | 2.6(5) |
| H76a | 79(11) | 51(11) | 25(5) | 5(9) | 27(3) | 12(4) |
| H76b | 32(9) | 46(10) | 49(11) | -15(7) | -8(8) | -3(8) |
| C77 | 43.5(9) | 15.6(6) | 21.9(7) | 6.9(6) | 8.6(6) | 3.0(5) |
| H77a | 51(11) | 77(13) | 18(9) | 22(9) | -7(8) | -8(9) |
| H77b | 106(14) | 18(9) | 68(13) | -2(9) | 37(11) | 11(9) |
| C78 | 43.8(9) | 15.5(6) | 20.7(7) | 3.7(6) | 9.1(6) | -2.8(5) |
| H78a | 70(12) | 35(10) | 45(11) | -5(9) | 8(10) | -11(8) |
| H78b | 88(13) | 32(10) | 73(13) | 26(9) | 50(11) | 1(9) |
| C79 | 40.7(8) | 17.6(6) | 23.9(7) | -0.6(6) | 17.4(6) | -3.3(5) |
| H79a | 30(9) | 62(12) | 57(12) | -2(8) | 12(9) | -36(10) |
| H79b | 110(14) | 39(10) | 35(10) | 2(9) | 45(10) | 5(8) |
| C80 | 34.0(7) | 11.9(6) | 17.2(6) | -1.2(5) | 9.9(6) | 0.3(5) |
| H80a | 78(11) | 21(9) | 40(10) | -5(8) | 35(9) | -1(7) |
| H80b | 47(10) | 47(10) | 21(9) | -7(8) | 2(8) | -8(8) |

**Table 3**: Bond Lengths in Å for **SE22001**.

| **Atom** | **Atom** | **Length/Å** | |
| --- | --- | --- | --- |
| Cl1 | C16 | 1.7396(12) |  |
| F1 | C22 | 1.3471(13) |  |
| O1 | C4 | 1.4239(12) |  |
| O2 | C10 | 1.4200(13) |  |
| N1 | C1 | 1.4675(15) |  |
| N1 | C8 | 1.4735(14) |  |
| N1 | C12 | 1.4724(15) |  |
| C1 | C2 | 1.5227(17) |  |
| C2 | C3 | 1.5325(17) |  |
| C3 | C4 | 1.5446(16) |  |
| C4 | C5 | 1.5654(15) |  |
| C4 | C19 | 1.5360(15) |  |
| C5 | C6 | 1.5051(16) |  |
| C5 | C25 | 1.5506(16) |  |
| C6 | C7 | 1.3256(19) |  |
| C8 | C9 | 1.5195(16) |  |
| C9 | C10 | 1.5297(16) |  |
| C10 | C11 | 1.5333(15) |  |
| C10 | C13 | 1.5283(15) |  |
| C11 | C12 | 1.5231(16) |  |
| C13 | C14 | 1.3880(16) |  |
| C13 | C18 | 1.3969(16) |  |
| C14 | C15 | 1.3937(17) |  |
| C15 | C16 | 1.3777(18) |  |
| C16 | C17 | 1.3845(18) |  |
| C17 | C18 | 1.3881(18) |  |
| C19 | C20 | 1.3941(15) |  |
| C19 | C24 | 1.3978(15) |  |
| C20 | C21 | 1.3936(16) |  |
| C21 | C22 | 1.3772(17) |  |
| C22 | C23 | 1.3758(17) |  |
| C23 | C24 | 1.3877(17) |  |
| C25 | C26 | 1.5320(17) |  |
| C25 | C30 | 1.5348(16) |  |
| C26 | C27 | 1.5235(19) |  |
| C27 | C28 | 1.5234(19) |  |
| C28 | C29 | 1.5198(19) |  |
| C29 | C30 | 1.5253(19) |  |
| Cl51 | C66 | 1.7380(13) |  |
| F51 | C72 | 1.3449(13) |  |
| O51 | C54 | 1.4252(12) |  |
| O52 | C60 | 1.4189(13) |  |
| N51 | C51 | 1.4677(15) |  |
| N51 | C58 | 1.4693(14) |  |
| N51 | C62 | 1.4729(14) |  |
| C51 | C52 | 1.5273(17) |  |
| C52 | C53 | 1.5327(17) |  |
| C53 | C54 | 1.5431(16) |  |
| C54 | C55 | 1.5702(15) |  |
| C54 | C69 | 1.5338(15) |  |
| C55 | C56 | 1.5054(16) |  |
| C55 | C75 | 1.5496(16) |  |
| C56 | C57 | 1.3251(19) |  |
| C58 | C59 | 1.5243(17) |  |
| C59 | C60 | 1.5309(15) |  |
| C60 | C61 | 1.5290(16) |  |
| C60 | C63 | 1.5301(16) |  |
| C61 | C62 | 1.5161(17) |  |
| C63 | C64 | 1.3922(17) |  |
| C63 | C68 | 1.3850(17) |  |
| C64 | C65 | 1.390(2) |  |
| C65 | C66 | 1.382(2) |  |
| C66 | C67 | 1.376(2) |  |
| C67 | C68 | 1.3937(19) |  |
| C69 | C70 | 1.3976(15) |  |
| C69 | C74 | 1.3949(15) |  |
| C70 | C71 | 1.3898(17) |  |
| C71 | C72 | 1.3814(18) |  |
| C72 | C73 | 1.3784(17) |  |
| C73 | C74 | 1.3912(16) |  |
| C75 | C76 | 1.5342(16) |  |
| C75 | C80 | 1.5339(16) |  |
| C76 | C77 | 1.5239(18) |  |
| C77 | C78 | 1.5241(19) |  |
| C78 | C79 | 1.5184(18) |  |
| C79 | C80 | 1.5284(18) |  |

**Table 4**: Bond Angles in ^°^ for **SE22001**.

| **Atom** | **Atom** | **Atom** | **Angle/^°^** | |
| --- | --- | --- | --- | --- |
| C8 | N1 | C1 | 108.64(9) |  |
| C12 | N1 | C1 | 111.92(9) |  |
| C12 | N1 | C8 | 110.55(9) |  |
| C2 | C1 | N1 | 115.33(10) |  |
| C3 | C2 | C1 | 115.99(10) |  |
| C4 | C3 | C2 | 117.71(10) |  |
| C3 | C4 | O1 | 109.82(9) |  |
| C5 | C4 | O1 | 105.25(8) |  |
| C5 | C4 | C3 | 107.86(9) |  |
| C19 | C4 | O1 | 109.46(8) |  |
| C19 | C4 | C3 | 109.36(9) |  |
| C19 | C4 | C5 | 114.96(9) |  |
| C6 | C5 | C4 | 114.43(9) |  |
| C25 | C5 | C4 | 115.16(9) |  |
| C25 | C5 | C6 | 111.10(10) |  |
| C7 | C6 | C5 | 124.00(13) |  |
| C9 | C8 | N1 | 112.62(10) |  |
| C10 | C9 | C8 | 111.27(10) |  |
| C9 | C10 | O2 | 110.28(9) |  |
| C11 | C10 | O2 | 105.55(9) |  |
| C11 | C10 | C9 | 106.83(9) |  |
| C13 | C10 | O2 | 108.75(8) |  |
| C13 | C10 | C9 | 113.07(9) |  |
| C13 | C10 | C11 | 112.09(9) |  |
| C12 | C11 | C10 | 110.50(10) |  |
| C11 | C12 | N1 | 111.03(9) |  |
| C14 | C13 | C10 | 123.30(10) |  |
| C18 | C13 | C10 | 119.00(10) |  |
| C18 | C13 | C14 | 117.68(11) |  |
| C15 | C14 | C13 | 121.65(12) |  |
| C16 | C15 | C14 | 119.07(12) |  |
| C15 | C16 | Cl1 | 119.46(10) |  |
| C17 | C16 | Cl1 | 119.55(10) |  |
| C17 | C16 | C15 | 120.98(12) |  |
| C18 | C17 | C16 | 119.09(12) |  |
| C17 | C18 | C13 | 121.52(12) |  |
| C20 | C19 | C4 | 120.95(9) |  |
| C24 | C19 | C4 | 121.37(10) |  |
| C24 | C19 | C20 | 117.51(11) |  |
| C21 | C20 | C19 | 121.40(11) |  |
| C22 | C21 | C20 | 118.74(11) |  |
| C21 | C22 | F1 | 118.96(11) |  |
| C23 | C22 | F1 | 119.07(11) |  |
| C23 | C22 | C21 | 121.97(11) |  |
| C24 | C23 | C22 | 118.44(11) |  |
| C23 | C24 | C19 | 121.93(12) |  |
| C26 | C25 | C5 | 116.17(10) |  |
| C30 | C25 | C5 | 109.64(10) |  |
| C30 | C25 | C26 | 108.74(10) |  |
| C27 | C26 | C25 | 110.61(11) |  |
| C28 | C27 | C26 | 111.51(13) |  |
| C29 | C28 | C27 | 110.77(11) |  |
| C30 | C29 | C28 | 111.65(11) |  |
| C29 | C30 | C25 | 112.71(11) |  |
| C58 | N51 | C51 | 111.83(9) |  |
| C62 | N51 | C51 | 108.36(9) |  |
| C62 | N51 | C58 | 110.57(9) |  |
| C52 | C51 | N51 | 115.43(10) |  |
| C53 | C52 | C51 | 115.56(10) |  |
| C54 | C53 | C52 | 117.82(10) |  |
| C53 | C54 | O51 | 109.63(9) |  |
| C55 | C54 | O51 | 105.41(8) |  |
| C55 | C54 | C53 | 107.44(9) |  |
| C69 | C54 | O51 | 109.70(8) |  |
| C69 | C54 | C53 | 109.55(9) |  |
| C69 | C54 | C55 | 114.95(9) |  |
| C56 | C55 | C54 | 114.45(9) |  |
| C75 | C55 | C54 | 115.16(9) |  |
| C75 | C55 | C56 | 111.11(10) |  |
| C57 | C56 | C55 | 124.26(13) |  |
| C59 | C58 | N51 | 111.46(9) |  |
| C60 | C59 | C58 | 110.94(10) |  |
| C59 | C60 | O52 | 105.18(9) |  |
| C61 | C60 | O52 | 110.09(10) |  |
| C61 | C60 | C59 | 107.38(9) |  |
| C63 | C60 | O52 | 109.21(9) |  |
| C63 | C60 | C59 | 111.87(10) |  |
| C63 | C60 | C61 | 112.82(9) |  |
| C62 | C61 | C60 | 111.05(10) |  |
| C61 | C62 | N51 | 112.15(10) |  |
| C64 | C63 | C60 | 119.33(11) |  |
| C68 | C63 | C60 | 122.91(10) |  |
| C68 | C63 | C64 | 117.76(12) |  |
| C65 | C64 | C63 | 121.56(14) |  |
| C66 | C65 | C64 | 119.12(13) |  |
| C65 | C66 | Cl51 | 119.16(11) |  |
| C67 | C66 | Cl51 | 120.10(12) |  |
| C67 | C66 | C65 | 120.74(13) |  |
| C68 | C67 | C66 | 119.30(14) |  |
| C67 | C68 | C63 | 121.51(13) |  |
| C70 | C69 | C54 | 121.40(10) |  |
| C74 | C69 | C54 | 120.99(9) |  |
| C74 | C69 | C70 | 117.47(11) |  |
| C71 | C70 | C69 | 121.93(12) |  |
| C72 | C71 | C70 | 118.40(12) |  |
| C71 | C72 | F51 | 119.12(11) |  |
| C73 | C72 | F51 | 119.07(11) |  |
| C73 | C72 | C71 | 121.81(11) |  |
| C74 | C73 | C72 | 118.78(12) |  |
| C73 | C74 | C69 | 121.61(11) |  |
| C76 | C75 | C55 | 109.88(9) |  |
| C80 | C75 | C55 | 116.05(9) |  |
| C80 | C75 | C76 | 108.50(10) |  |
| C77 | C76 | C75 | 112.30(11) |  |
| C78 | C77 | C76 | 111.32(11) |  |
| C79 | C78 | C77 | 111.18(11) |  |
| C80 | C79 | C78 | 111.61(12) |  |
| C79 | C80 | C75 | 110.52(10) |  |

**Table 5**: Torsion Angles in ^°^ for **SE22001**.

| **Atom** | **Atom** | **Atom** | **Atom** | **Angle/^°^** |  |
| --- | --- | --- | --- | --- | --- |
| Cl1 | C16 | C15 | C14 | -179.66(10) | |
| Cl1 | C16 | C17 | C18 | 179.13(11) | |
| F1 | C22 | C21 | C20 | -179.18(10) | |
| F1 | C22 | C23 | C24 | 178.71(11) | |
| O1 | C4 | C3 | C2 | -67.48(10) | |
| O1 | C4 | C5 | C6 | -175.90(9) | |
| O1 | C4 | C5 | C25 | 53.54(9) | |
| O1 | C4 | C19 | C20 | -7.71(11) | |
| O1 | C4 | C19 | C24 | 167.46(9) | |
| O2 | C10 | C9 | C8 | -57.84(10) | |
| O2 | C10 | C11 | C12 | 58.76(9) | |
| O2 | C10 | C13 | C14 | -141.07(9) | |
| O2 | C10 | C13 | C18 | 37.14(11) | |
| N1 | C1 | C2 | C3 | -67.11(12) | |
| N1 | C8 | C9 | C10 | -56.38(11) | |
| N1 | C12 | C11 | C10 | 60.56(10) | |
| C1 | C2 | C3 | C4 | 85.95(12) | |
| C2 | C3 | C4 | C5 | 178.32(10) | |
| C2 | C3 | C4 | C19 | 52.65(11) | |
| C3 | C4 | C5 | C6 | -58.70(10) | |
| C3 | C4 | C5 | C25 | 170.74(9) | |
| C3 | C4 | C19 | C20 | -128.06(9) | |
| C3 | C4 | C19 | C24 | 47.10(11) | |
| C4 | C5 | C6 | C7 | 118.94(12) | |
| C4 | C5 | C25 | C26 | 79.41(11) | |
| C4 | C5 | C25 | C30 | -156.84(10) | |
| C4 | C19 | C20 | C21 | 174.91(10) | |
| C4 | C19 | C24 | C23 | -175.38(11) | |
| C5 | C25 | C26 | C27 | -178.13(11) | |
| C5 | C25 | C30 | C29 | 176.09(10) | |
| C8 | C9 | C10 | C11 | 56.38(11) | |
| C8 | C9 | C10 | C13 | -179.85(9) | |
| C9 | C10 | C11 | C12 | -58.62(9) | |
| C9 | C10 | C13 | C14 | -18.21(12) | |
| C9 | C10 | C13 | C18 | 160.00(10) | |
| C10 | C13 | C14 | C15 | 177.03(11) | |
| C10 | C13 | C18 | C17 | -177.66(11) | |
| C13 | C14 | C15 | C16 | 0.65(14) | |
| C13 | C18 | C17 | C16 | 0.45(15) | |
| C14 | C15 | C16 | C17 | 0.51(16) | |
| C15 | C16 | C17 | C18 | -1.04(15) | |
| C19 | C20 | C21 | C22 | 0.51(13) | |
| C19 | C24 | C23 | C22 | 0.45(15) | |
| C20 | C21 | C22 | C23 | -0.09(14) | |
| C21 | C22 | C23 | C24 | -0.38(14) | |
| C25 | C26 | C27 | C28 | -59.02(12) | |
| C25 | C30 | C29 | C28 | 54.37(12) | |
| C26 | C27 | C28 | C29 | 55.90(14) | |
| C27 | C28 | C29 | C30 | -53.04(14) | |
| Cl51 | C66 | C65 | C64 | 179.07(11) | |
| Cl51 | C66 | C67 | C68 | -179.60(12) | |
| F51 | C72 | C71 | C70 | 178.79(11) | |
| F51 | C72 | C73 | C74 | -179.20(10) | |
| O51 | C54 | C53 | C52 | -68.31(10) | |
| O51 | C54 | C55 | C56 | -178.67(9) | |
| O51 | C54 | C55 | C75 | 50.74(9) | |
| O51 | C54 | C69 | C70 | 168.61(9) | |
| O51 | C54 | C69 | C74 | -7.13(11) | |
| O52 | C60 | C59 | C58 | 60.38(10) | |
| O52 | C60 | C61 | C62 | -57.66(10) | |
| O52 | C60 | C63 | C64 | 32.97(12) | |
| O52 | C60 | C63 | C68 | -145.70(10) | |
| N51 | C51 | C52 | C53 | -68.31(12) | |
| N51 | C58 | C59 | C60 | 58.54(10) | |
| N51 | C62 | C61 | C60 | -57.63(11) | |
| C51 | C52 | C53 | C54 | 85.01(12) | |
| C52 | C53 | C54 | C55 | 177.62(11) | |
| C52 | C53 | C54 | C69 | 52.12(11) | |
| C53 | C54 | C55 | C56 | -61.80(11) | |
| C53 | C54 | C55 | C75 | 167.60(9) | |
| C53 | C54 | C69 | C70 | 48.22(11) | |
| C53 | C54 | C69 | C74 | -127.52(9) | |
| C54 | C55 | C56 | C57 | 113.36(12) | |
| C54 | C55 | C75 | C76 | -159.84(10) | |
| C54 | C55 | C75 | C80 | 76.63(10) | |
| C54 | C69 | C70 | C71 | -176.06(11) | |
| C54 | C69 | C74 | C73 | 175.66(10) | |
| C55 | C75 | C76 | C77 | 174.73(10) | |
| C55 | C75 | C80 | C79 | -177.57(11) | |
| C58 | C59 | C60 | C61 | -56.87(11) | |
| C58 | C59 | C60 | C63 | 178.83(9) | |
| C59 | C60 | C61 | C62 | 56.34(10) | |
| C59 | C60 | C63 | C64 | -83.05(11) | |
| C59 | C60 | C63 | C68 | 98.28(11) | |
| C60 | C63 | C64 | C65 | -178.97(12) | |
| C60 | C63 | C68 | C67 | 178.38(13) | |
| C63 | C64 | C65 | C66 | 0.83(17) | |
| C63 | C68 | C67 | C66 | 0.25(16) | |
| C64 | C65 | C66 | C67 | -0.89(18) | |
| C65 | C66 | C67 | C68 | 0.36(17) | |
| C69 | C70 | C71 | C72 | 0.49(14) | |
| C69 | C74 | C73 | C72 | 0.34(13) | |
| C70 | C71 | C72 | C73 | -0.40(15) | |
| C71 | C72 | C73 | C74 | -0.01(14) | |
| C75 | C76 | C77 | C78 | 55.41(12) | |
| C75 | C80 | C79 | C78 | -58.32(12) | |
| C76 | C77 | C78 | C79 | -53.09(13) | |
| C77 | C78 | C79 | C80 | 55.03(13) | |

**Table 6**: Hydrogen Fractional Atomic Coordinates (×10^4^) and Equivalent Isotropic Displacement Parameters (Å^2^×10^3^) for **SE22001**. *U_eq_* is defined as 1/3 of the trace of the orthogonalised *U_ij_*.

| **Atom** | **x** | **y** | **z** | ***U_eq_*** |
| --- | --- | --- | --- | --- |
| H1 | 1760(6) | 410(16) | 1474(9) | 30(5) |
| H2 | 3119(7) | -2842(15) | 3081(8) | 48(5) |
| H1a | 1199(5) | -868(15) | 2322(8) | 47(4) |
| H1b | 1312(5) | -2384(14) | 2050(8) | 50(5) |
| H2a | 572(5) | -1568(14) | 1236(7) | 49(4) |
| H2b | 1051(5) | -1602(13) | 801(7) | 35(4) |
| H3a | 807(6) | 811(13) | 1485(7) | 42(4) |
| H3b | 420(5) | 351(12) | 677(8) | 36(4) |
| H5 | 953(5) | 2695(12) | 1041(7) | 28(3) |
| H6 | 348(5) | 2104(14) | -477(8) | 49(4) |
| H7a | 87(6) | 3336(15) | 756(9) | 60(5) |
| H7b | -367(5) | 3032(15) | -110(9) | 68(6) |
| H8a | 2136(5) | -1940(12) | 2952(7) | 34(4) |
| H8b | 1972(5) | -336(15) | 3012(8) | 49(4) |
| H9a | 2758(5) | 383(12) | 2721(7) | 28(3) |
| H9b | 2940(5) | -701(14) | 3414(7) | 43(4) |
| H11a | 2870(6) | -1920(13) | 1358(8) | 46(4) |
| H11b | 2707(5) | -399(11) | 1509(7) | 34(4) |
| H12a | 1898(5) | -1700(12) | 1046(7) | 33(4) |
| H12b | 2113(5) | -2776(12) | 1746(8) | 40(4) |
| H14 | 3561(5) | 743(12) | 2960(9) | 49(5) |
| H15 | 4506(5) | 1182(14) | 3166(8) | 52(5) |
| H17 | 4711(6) | -2462(15) | 2396(10) | 66(5) |
| H18 | 3771(6) | -2907(14) | 2208(9) | 54(5) |
| H20 | 2072(5) | 278(13) | 350(7) | 37(4) |
| H21 | 2149(6) | -1107(14) | -636(8) | 53(5) |
| H23 | 497(6) | -1386(16) | -1356(8) | 66(5) |
| H24 | 410(5) | -85(14) | -391(8) | 46(4) |
| H25 | 1738(5) | 3082(12) | 595(7) | 32(4) |
| H26a | 949(6) | 3402(14) | -819(7) | 48(4) |
| H26b | 1421(7) | 2218(15) | -618(9) | 68(5) |
| H27a | 1732(7) | 3999(15) | -1193(9) | 72(6) |
| H27b | 2134(6) | 3878(15) | -302(9) | 56(5) |
| H28a | 1235(6) | 5711(14) | -836(8) | 58(5) |
| H28b | 1884(6) | 6127(15) | -614(8) | 52(5) |
| H29a | 1969(6) | 5450(15) | 589(8) | 54(5) |
| H29b | 1462(6) | 6553(13) | 330(9) | 58(5) |
| H30a | 1211(6) | 4719(14) | 969(8) | 54(5) |
| H30b | 807(5) | 4848(12) | 108(8) | 43(4) |
| H51 | 3267(5) | 5509(15) | 3660(8) | 26(4) |
| H52 | 1985(7) | 2101(15) | 1974(8) | 41(5) |
| H51a | 3816(5) | 2937(14) | 3022(8) | 47(4) |
| H51b | 3871(5) | 4517(14) | 2786(7) | 43(4) |
| H52a | 4063(5) | 3648(13) | 4288(7) | 38(4) |
| H52b | 4540(5) | 3836(13) | 3849(8) | 45(4) |
| H53a | 4224(5) | 6152(13) | 3636(8) | 42(4) |
| H53b | 4624(5) | 5726(12) | 4452(7) | 36(4) |
| H55 | 4029(5) | 7966(12) | 4122(6) | 29(3) |
| H56 | 4600(6) | 7424(15) | 5656(8) | 58(5) |
| H57a | 4920(6) | 8575(17) | 4454(9) | 70(6) |
| H57b | 5332(5) | 8347(15) | 5352(9) | 66(6) |
| H58a | 3220(5) | 3259(13) | 4047(7) | 36(4) |
| H58b | 3053(5) | 2271(12) | 3320(7) | 37(4) |
| H59a | 2367(5) | 4358(13) | 3638(7) | 39(4) |
| H59b | 2275(5) | 2749(13) | 3708(8) | 44(4) |
| H61a | 2291(5) | 5370(13) | 2466(8) | 44(4) |
| H61b | 2128(5) | 4407(13) | 1732(7) | 40(4) |
| H62a | 3087(5) | 4941(15) | 2153(7) | 46(4) |
| H62b | 2974(5) | 3274(13) | 2140(7) | 39(4) |
| H64 | 1382(6) | 1703(15) | 2747(10) | 70(6) |
| H65 | 434(6) | 1958(15) | 2617(10) | 78(6) |
| H67 | 548(6) | 5872(15) | 2180(11) | 79(7) |
| H68 | 1491(6) | 5611(14) | 2332(10) | 67(6) |
| H70 | 4649(5) | 5243(14) | 5521(8) | 47(4) |
| H71 | 4591(6) | 3814(15) | 6466(8) | 63(5) |
| H73 | 2931(6) | 3743(14) | 5709(8) | 56(5) |
| H74 | 2975(5) | 5209(13) | 4750(8) | 46(4) |
| H75 | 3215(5) | 8216(11) | 4483(7) | 27(3) |
| H76a | 3754(6) | 9959(14) | 4238(8) | 49(4) |
| H76b | 4086(5) | 10037(13) | 5107(8) | 46(4) |
| H77a | 2928(6) | 10504(15) | 4510(7) | 52(5) |
| H77b | 3388(6) | 11648(14) | 4844(8) | 62(5) |
| H78a | 3565(6) | 10717(13) | 6009(8) | 52(5) |
| H78b | 2903(6) | 11013(14) | 5696(9) | 59(5) |
| H79a | 2746(5) | 8765(14) | 5344(8) | 50(5) |
| H79b | 3075(6) | 8869(14) | 6233(8) | 56(5) |
| H80a | 3479(6) | 7221(13) | 5664(7) | 43(4) |
| H80b | 3926(5) | 8455(13) | 5965(7) | 40(4) |

**Table 7**: Hydrogen Bond information for **SE22001**.

| **D** | **H** | **A** | **d(D-H)/Å** | **d(H-A)/Å** | **d(D-A)/Å** | **D-H-A/deg** |
| --- | --- | --- | --- | --- | --- | --- |
| O1 | H1 | N1 | 0.948(17) | 1.769(18) | 2.7160(12) | 176.1(14) |
| O2 | H2 | O51^1^ | 0.874(15) | 1.930(15) | 2.7505(12) | 155.8(16) |
| O51 | H51 | N51 | 0.957(16) | 1.763(17) | 2.7169(12) | 174.2(13) |
| O52 | H52 | O1 | 0.897(15) | 1.890(16) | 2.7203(12) | 153.1(15) |

––––

^1^+x,-1+y,+z

**Citations**

**CrysAlisPro** (Rigaku, V1.171.41.99a, 2021)

CrysAlisPro (ROD), Rigaku Oxford Diffraction, Poland (?).

L.J. Bourhis and O.V. Dolomanov and R.J. Gildea and J.A.K. Howard and H. Puschmann, The Anatomy of a Comprehensive Constrained, Restrained, Refinement Program for the Modern Computing Environment - Olex2 Disected, *Acta Cryst. A*, (2015), **A71**, 59-71.

O.V. Dolomanov and L.J. Bourhis and R.J. Gildea and J.A.K. Howard and H. Puschmann, Olex2: A complete structure solution, refinement and analysis program, *J. Appl. Cryst.*, (2009), **42**, 339-341.

Sheldrick, G.M., ShelXT-Integrated space-group and crystal-structure determination, *Acta Cryst.*, (2015), **A71**, 3-8.

#===============================================================================

# PLATON/CHECK-(181221) versus check.def version 211218, Entry: SE22001

# Data: SE22001.cif - Type: CIF Bond Precision C-C = 0.0018 A

# Refl: SE22001.fcf - Type: LIST4 Temp = 120 K

# Audit:OLEX2 1.5-BETA (COMPILED 2021.12.09 SVN.R5202D8CF FOR OLEXSYS, GUI SVN.R

# Refin:OLEX2.REFINE 1.5-BETA (BOURHIS ET AL., 2015)

# X-ray MoKa R(int) = 0.050, wR2/R(int) = 1.6, Nref/Npar = 11.3

# Cell 26.4305(9) 10.5769(3) 19.9763(8) 90 106.275(4) 90

# Wavelength 0.71073 Volume Reported 5360.6(3) Calculated 5360.7(3)

# SpaceGroup from Symmetry P 21/c Hall: -P 2ybc monoclinic

# Reported P 1 21/c 1 -P 2ybc monoclinic

# MoietyFormula C30 H39 Cl F N O2

# Reported C30 H39 Cl F N O2

# SumFormula C30 H39 Cl F N O2

# Reported C30 H39 Cl F N O2

# Mr = 500.07[Calc], 500.10[Rep] Volume/NonHatoms = 19 Ang**3

# Dx,gcm-3 = 1.239[Calc], 1.239[Rep]

# Z = 8[Calc], 8[Rep]

# Mu (mm-1) = 0.177[Calc], 0.177[Rep] Xtal Size = 0.207x0.234x0.284 mm

# F000 = 2144.0[Calc], 2146.4[Rep] or F000' = 2146.07[Calc]

# Reported T Limits: Tmin=0.981 Tmax=1.000 AbsCorr = MULTI-SCAN

# Calculated T Limits: Tmin=0.952 Tmin'=0.951 Tmax=0.964

# Measured HKL: Reported 64971, Embedded 0, <Mult> 0.0

# Reported Hmax= 36, Kmax= 14, Lmax= 27, Nref= 15011 , Th(max)= 29.570

# Obs in FCF Hmax= 36, Kmax= 14, Lmax= 27, Nref= 15011[ 15011], Th(max)= 29.574

# Calculated Hmax= 36, Kmax= 14, Lmax= 27, Nref= 15032 , Ratio = 0.999

# Reported Rho(min) = -0.57, Rho(max) = 0.47 e/Ang**3 (From CIF)

# Calculated Rho(min) = -0.37, Rho(max) = 0.57 e/Ang**3 (From CIF+FCF data)

# w=1/[<sup>2</sup>(Fo<sup>2</sup>)+(0.0196P)<sup>2</sup>+0.5306P], P=(Fo<sup>2</sup>+2Fc<sup>2</sup>)/3

# R= 0.0685( 11607), wR2= 0.1267( 15011), S = 1.765 (From CIF+FCF data)

# R= 0.0492( 11607), wR2= 0.0784( 15011), S = 1.092 (From FCF data only)

# R= 0.0492( 11607), wR2= 0.0784( 15011), S = 1.092, Npar= 1333

#===============================================================================

# For Documentation:http://www.platonsoft.nl/CIF-VALIDATION.pdf

#===============================================================================

*

#===============================================================================

#>>> The Following Improvement and Query ALERTS were generated - (Acta-Mode) <<<

#===============================================================================

Format: alert-number_ALERT_alert-type_alert-level text

417_ALERT_2_B Short Inter D-H..H-D H1 ..H52 . 2.05 Ang.

x,y,z = 1_555 Check

417_ALERT_2_B Short Inter D-H..H-D H2 ..H51 . 2.07 Ang.

x,-1+y,z = 1_545 Check

910_ALERT_3_B Missing # of FCF Reflection(s) Below Theta(Min). 24 Note

#===============================================================================

351_ALERT_3_C Long C-H (X0.96,N1.08A) C5 - H5 . 1.11 Ang.

351_ALERT_3_C Long C-H (X0.96,N1.08A) C12 - H12B . 1.11 Ang.

351_ALERT_3_C Long C-H (X0.96,N1.08A) C27 - H27B . 1.11 Ang.

411_ALERT_2_C Short Inter H...H Contact H3B ..H24 . 2.13 Ang.

-x,-y,-z = 3_555 Check

906_ALERT_3_C Large K Value in the Analysis of Variance ...... 9.347 Check

906_ALERT_3_C Large K Value in the Analysis of Variance ...... 2.068 Check

#===============================================================================

003_ALERT_2_G Number of Uiso or Uij Restrained non-H Atoms ... 2 Report

068_ALERT_1_G Reported F000 Differs from Calcd (or Missing)... Please Check

187_ALERT_4_G The CIF-Embedded .res File Contains RIGU Records 2 Report

793_ALERT_4_G Model has Chirality at C4 (Centro SPGR) S Verify

793_ALERT_4_G Model has Chirality at C5 (Centro SPGR) R Verify

793_ALERT_4_G Model has Chirality at C54 (Centro SPGR) S Verify

793_ALERT_4_G Model has Chirality at C55 (Centro SPGR) R Verify

802_ALERT_4_G CIF Input Record(s) with more than 80 Characters 1 Info

860_ALERT_3_G Number of Least-Squares Restraints ............. 6 Note

912_ALERT_4_G Missing # of FCF Reflections Above STh/L= 0.600 2 Note

933_ALERT_2_G Number of HKL-OMIT Records in Embedded .res File 1 Note

960_ALERT_3_G Number of Intensities with I < - 2*sig(I) ... 2 Check

978_ALERT_2_G Number C-C Bonds with Positive Residual Density. 8 Info

979_ALERT_1_G NoSpherA2 Scattering Factors Used .............. Please Note

#===============================================================================

ALERT_Level and ALERT_Type Summary

==================================

3 ALERT_Level_B = A Potentially Serious Problem - Consider Carefully

6 ALERT_Level_C = Check. Ensure it is Not caused by an Omission or Oversight

14 ALERT_Level_G = General Info/Check that it is not Something Unexpected

2 ALERT_Type_1 CIF Construction/Syntax Error, Inconsistent or Missing Data.

6 ALERT_Type_2 Indicator that the Structure Model may be Wrong or Deficient.

8 ALERT_Type_3 Indicator that the Structure Quality may be Low.

7 ALERT_Type_4 Improvement, Methodology, Query or Suggestion.

#===============================================================================

0 Missing Experimental Info Issue(s) (Out of 64 Tests) - 100 % Satisfied

0 Experimental Data Related Issue(s) (Out of 35 Tests) - 100 % Satisfied

9 Structural Model Related Issue(s) (Out of 136 Tests) - 93 % Satisfied

14 Unresolved or to be Checked Issue(s) (Out of 272 Tests) - 95 % Satisfied

*
