# Supplementary material for: Boron-Catalyzed, Diastereo- and Enantioselective Allylation of Ketones with Allenes
Source: ACS Catal. 2022 Aug 22;12(17):10887–93. doi: 10.1021/acscatal.2c03158 (PMC9442582; doi:10.1021/acscatal.2c03158)

checkCIF/PLATON report

Structure factors have been supplied for datablock(s) SE22001

THIS REPORT IS FOR GUIDANCE ONLY. IF USED AS PART OF A REVIEW PROCEDURE FOR PUBLICATION, IT SHOULD NOT REPLACE THE EXPERTISE OF AN EXPERIENCED CRYSTALLOGRAPHIC REFEREE.

No syntax errors found. CIF dictionary Interpreting this report

Datablock: SE22001

Bond precision: C-C = 0.0018 A Wavelength=0.71073

Cell:	a=26.4305(9)	b=10.5769(3)	c=19.9763(8)
	alpha=90	beta=106.275(4)	gamma=90
Temperature:	120 K		

	Calculated	Reported
Volume	5360.7(3)	5360.6(3)
Space group	P 21/c	P 1 21/c 1
Hall group	-P 2ybc	-P 2ybc
Moiety formula	C30 H39 Cl F N O2	C30 H39 Cl F N O2
Sum formula	C30 H39 Cl F N O2	C30 H39 Cl F N O2
Mr	500.07	500.10
Dx,g cm-3	1.239	1.239
Z	8	8
Mu (mm-1)	0.177	0.177
F000	2144.0	2146.4
F000'	2146.07	
h,k,lmax	36,14,27	36,14,27
Nref	15032	15011
Tmin,Tmax	0.952,0.964	0.981,1.000
Tmin'	0.951	

Correction method= # Reported T Limits: Tmin=0.981 Tmax=1.000
AbsCorr = MULTI-SCAN

Data completeness= 0.999 Theta(max)= 29.570

R(reflections)= 0.0492(11607) wR2(reflections)= 0.0784(15011)

S = 1.092 Npar= 1333

The following ALERTS were generated. Each ALERT has the format
test-name_ALERT_alert-type_alert-level.
Click on the hyperlinks for more details of the test.

Alert level B

```
PLAT417_ALERT_2_B Short Inter D-H..H-D       H1        ..H52      .       2.05 Ang.
                                                   x,y,z  =      1_555 Check
PLAT417_ALERT_2_B Short Inter D-H..H-D       H2        ..H51      .       2.07 Ang.
                                                x,-1+y,z  =      1_545 Check
PLAT910_ALERT_3_B Missing # of FCF Reflection(s) Below Theta(Min).         24 Note
```

Alert level C

```
PLAT351_ALERT_3_C Long   C-H (X0.96,N1.08A)  C5        - H5       .       1.11 Ang.
PLAT351_ALERT_3_C Long   C-H (X0.96,N1.08A)  C12       - H12B     .       1.11 Ang.
PLAT351_ALERT_3_C Long   C-H (X0.96,N1.08A)  C27       - H27B     .       1.11 Ang.
PLAT411_ALERT_2_C Short Inter H...H Contact  H3B       ..H24      .       2.13 Ang.
                                                -x,-y,-z  =      3_555 Check
PLAT906_ALERT_3_C Large K Value in the Analysis of Variance ......      9.355 Check
PLAT906_ALERT_3_C Large K Value in the Analysis of Variance ......      2.068 Check
```

Alert level G

```
PLAT003_ALERT_2_G Number of Uiso or Uij Restrained non-H Atoms ...          2 Report
PLAT068_ALERT_1_G Reported F000 Differs from Calcd (or Missing)...     Please Check
PLAT187_ALERT_4_G The CIF-Embedded .res File Contains RIGU Records          2 Report
PLAT793_ALERT_4_G Model has Chirality at C4          (Centro SPGR)          S Verify
PLAT793_ALERT_4_G Model has Chirality at C5          (Centro SPGR)          R Verify
PLAT793_ALERT_4_G Model has Chirality at C54         (Centro SPGR)          S Verify
PLAT793_ALERT_4_G Model has Chirality at C55         (Centro SPGR)          R Verify
PLAT802_ALERT_4_G CIF Input Record(s) with more than 80 Characters          1 Info
PLAT860_ALERT_3_G Number of Least-Squares Restraints .............          6 Note
PLAT912_ALERT_4_G Missing # of FCF Reflections Above STh/L=  0.600          2 Note
PLAT933_ALERT_2_G Number of HKL-OMIT Records in Embedded .res File          1 Note
PLAT960_ALERT_3_G Number of Intensities with I  <   - 2*sig(I) ...          2 Check
PLAT978_ALERT_2_G Number C-C Bonds with Positive Residual Density.          8 Info
PLAT979_ALERT_1_G NoSpherA2 Scattering Factors Used ..............     Please Note
```

```
   0 ALERT level A = Most likely a serious problem - resolve or explain
   3 ALERT level B = A potentially serious problem, consider carefully
   6 ALERT level C = Check. Ensure it is not caused by an omission or oversight
  14 ALERT level G = General information/check it is not something unexpected

   2 ALERT type 1 CIF construction/syntax error, inconsistent or missing data
   6 ALERT type 2 Indicator that the structure model may be wrong or deficient
   8 ALERT type 3 Indicator that the structure quality may be low
   7 ALERT type 4 Improvement, methodology, query or suggestion
   0 ALERT type 5 Informative message, check
```

It is advisable to attempt to resolve as many as possible of the alerts in all categories. Often the minor alerts point to easily fixed oversights, errors and omissions in your CIF or refinement strategy, so attention to these fine details can be worthwhile. In order to resolve some of the more serious problems it may be necessary to carry out additional measurements or structure refinements. However, the purpose of your study may justify the reported deviations and the more serious of these should normally be commented upon in the discussion or experimental section of a paper or in the "special_details" fields of the CIF. checkCIF was carefully designed to identify outliers and unusual parameters, but every test has its limitations and alerts that are not important in a particular case may appear. Conversely, the absence of alerts does not guarantee there are no aspects of the results needing attention. It is up to the individual to critically assess their own results and, if necessary, seek expert advice.

Publication of your CIF in IUCr journals

A basic structural check has been run on your CIF. These basic checks will be run on all CIFs submitted for publication in IUCr journals (*Acta Crystallographica*, *Journal of Applied Crystallography*, *Journal of Synchrotron Radiation*); however, if you intend to submit to *Acta Crystallographica Section C* or *E* or *IUCrData*, you should make sure that full publication checks are run on the final version of your CIF prior to submission.

Publication of your CIF in other journals

Please refer to the *Notes for Authors* of the relevant journal for any special instructions relating to CIF submission.

PLATON version of 18/12/2021; check.def file version of 18/12/2021

Datablock SE22001 - ellipsoid plot

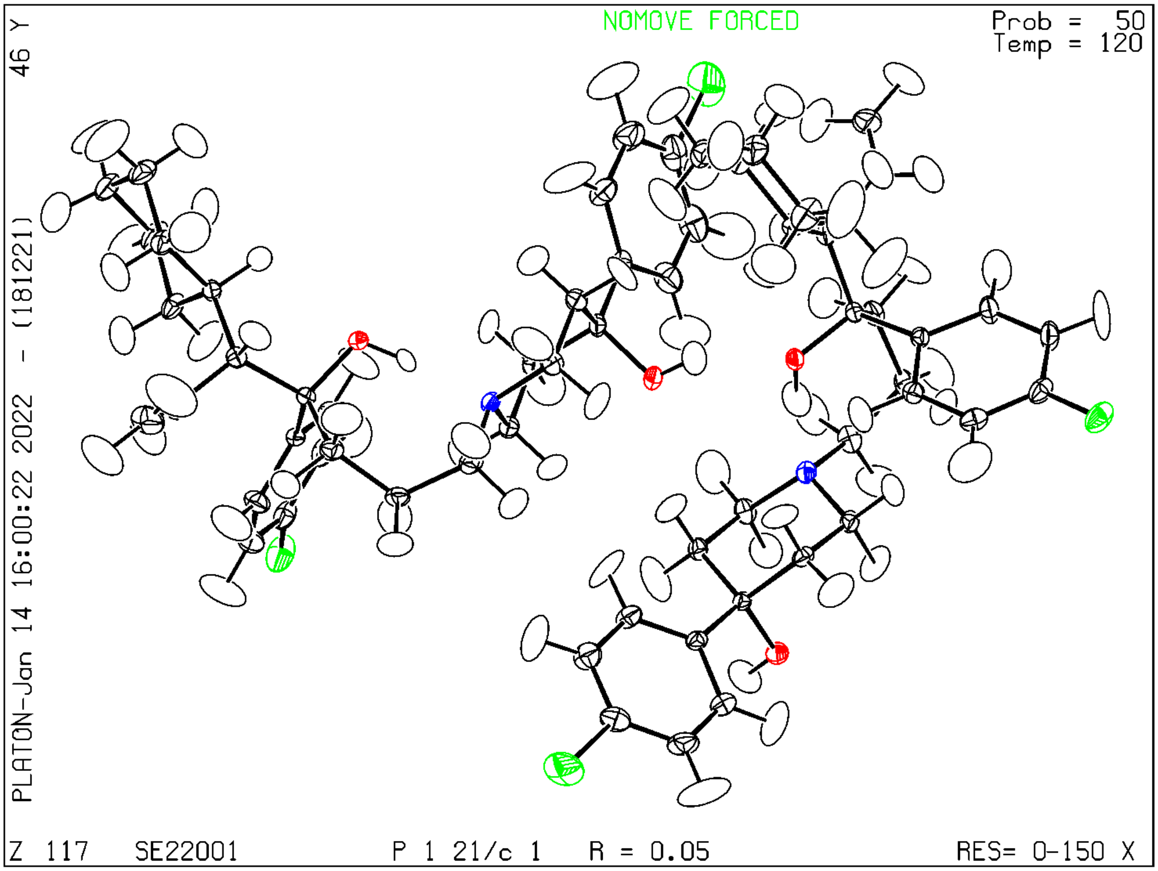

Supplement: Supplementary file 2 — cs2c03158_si_002.zip [file cs2c03158_si_002.zip › SE22001_cifreport.pdf]
