# Supplementary figures and images for: Boron-Catalyzed, Diastereo- and Enantioselective Allylation of Ketones with Allenes
Source: ACS Catal. 2022 Aug 22;12(17):10887–93. doi: 10.1021/acscatal.2c03158 (PMC9442582; doi:10.1021/acscatal.2c03158)

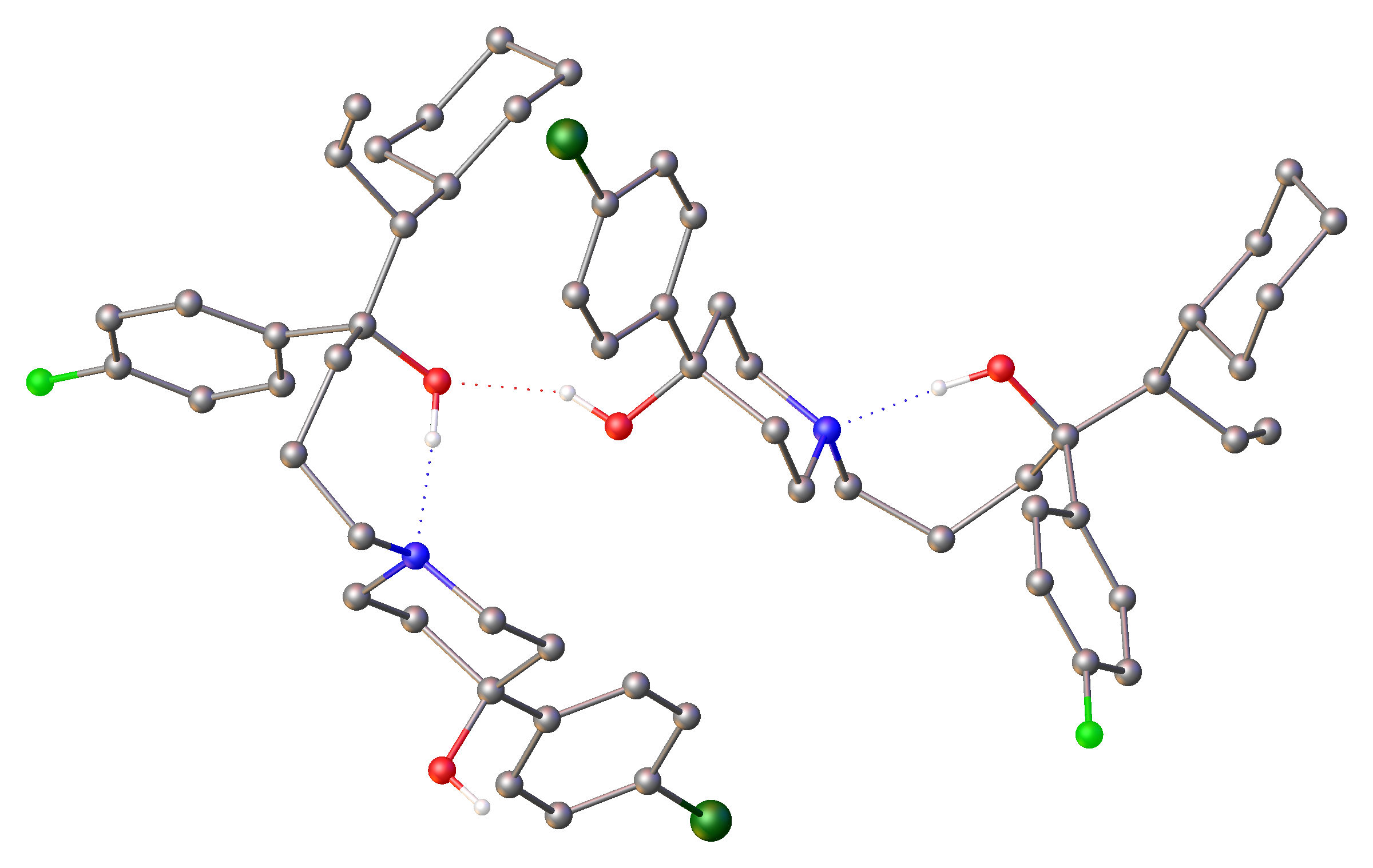

Supplement: Supplementary file 2 — cs2c03158_si_002.zip [file cs2c03158_si_002.zip › SE22001.png]

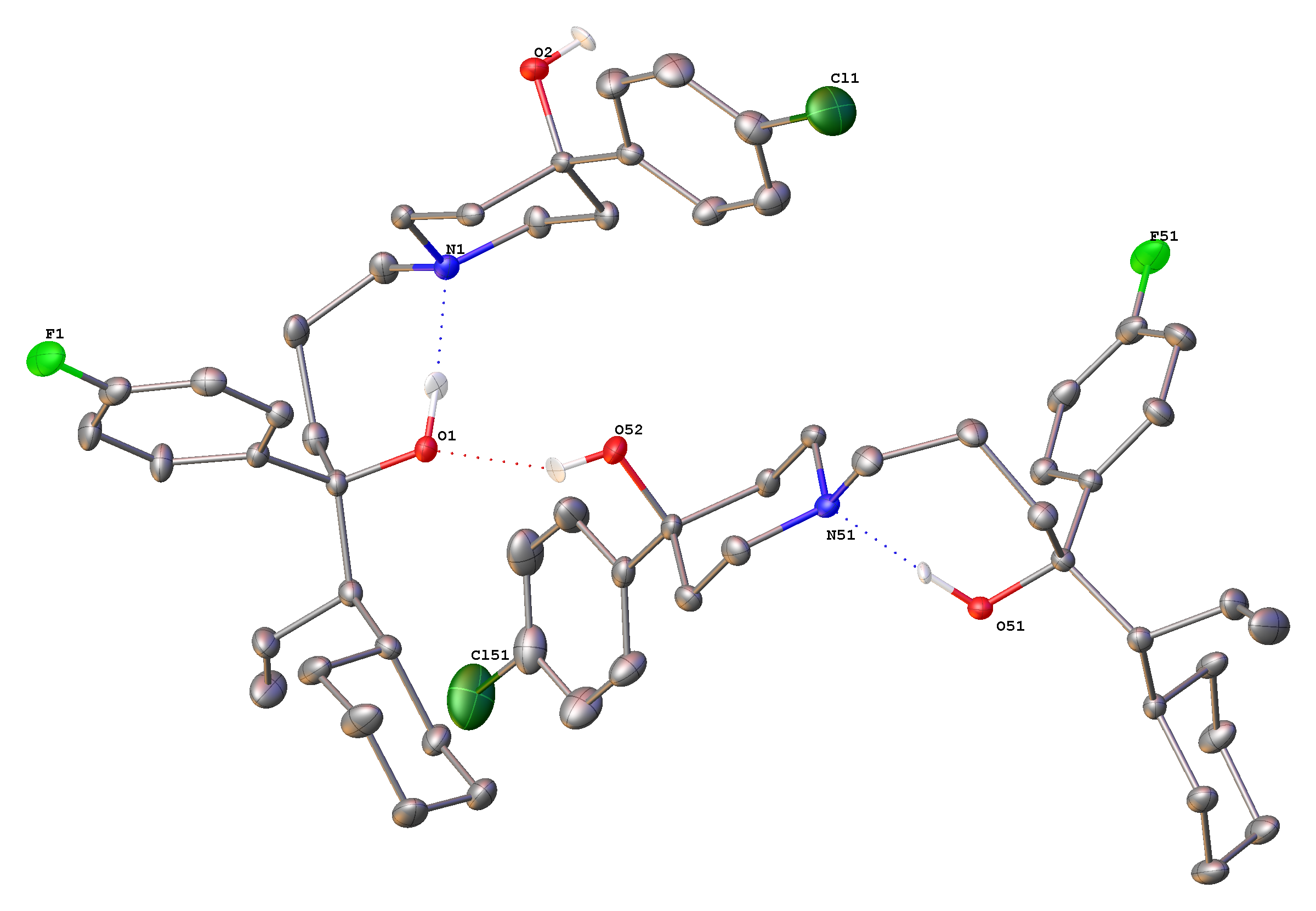

Supplement: Supplementary file 2 — cs2c03158_si_002.zip [file cs2c03158_si_002.zip › SE22001_telp50.png]

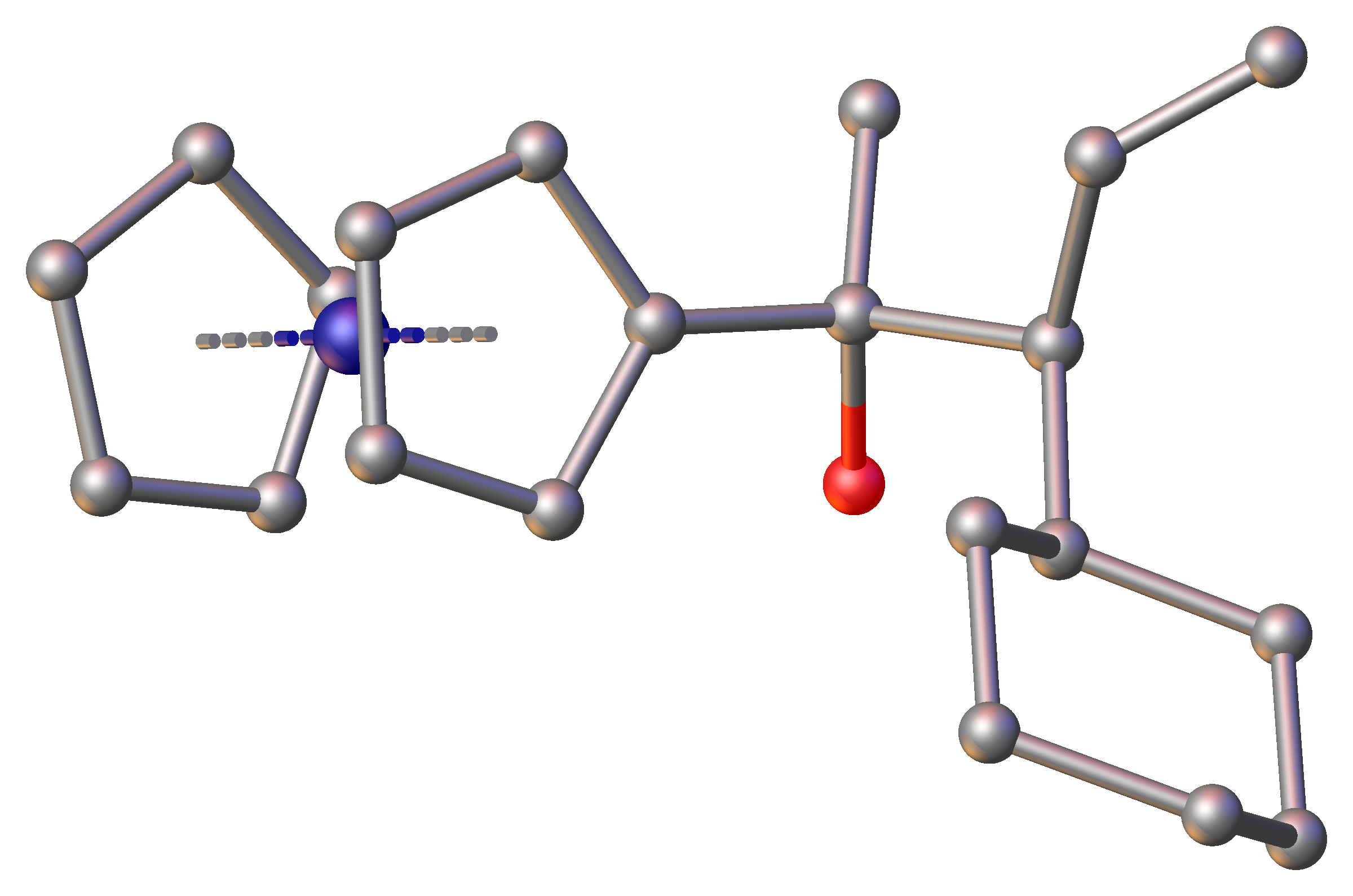

Supplement: Supplementary file 3 — cs2c03158_si_003.zip [file cs2c03158_si_003.zip › SE22004.png]

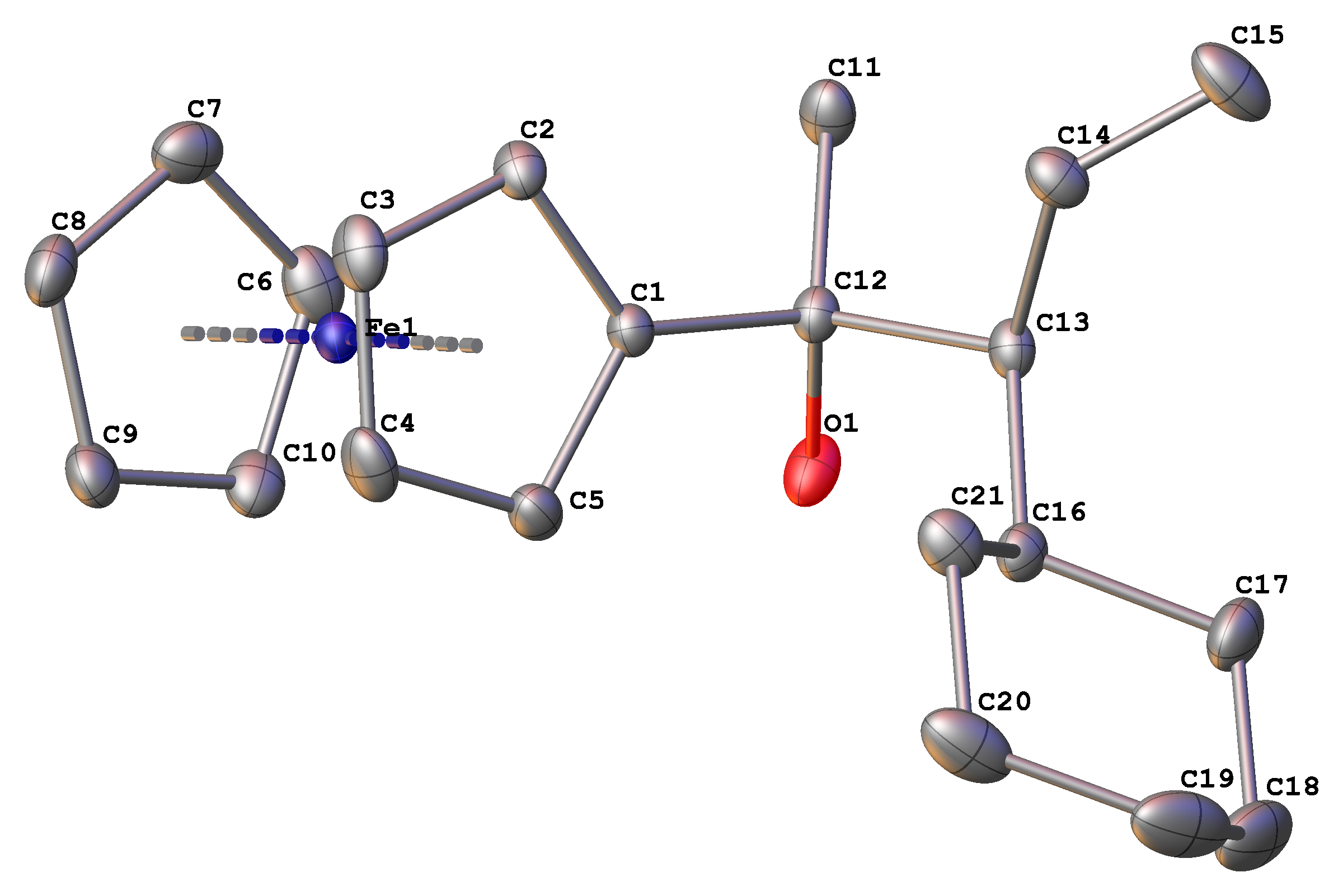

Supplement: Supplementary file 3 — cs2c03158_si_003.zip [file cs2c03158_si_003.zip › SE22004_telp50.png]
