# Supplementary material for: Boron-Catalyzed, Diastereo- and Enantioselective Allylation of Ketones with Allenes
Source: ACS Catal. 2022 Aug 22;12(17):10887–93. doi: 10.1021/acscatal.2c03158 (PMC9442582; doi:10.1021/acscatal.2c03158)
Supplement: Supplementary file 3 — cs2c03158_si_003.zip [file cs2c03158_si_003.zip › SE22004.docx]

**SE22004**


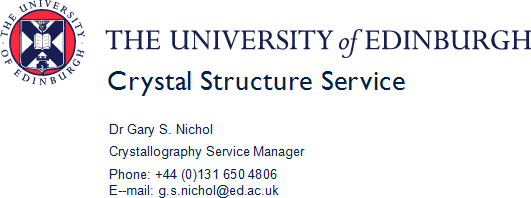


Submitted by: **Kieran Nicholson**

Solved by: **Gary S Nichol**

Sample ID: **KN07-113**

***R_1_*=2.31%**

Compound KN07-113 was provided as crystals suitable for single crystal X-ray diffraction, yielding structure SE22004.

Crystal Data and Experimental


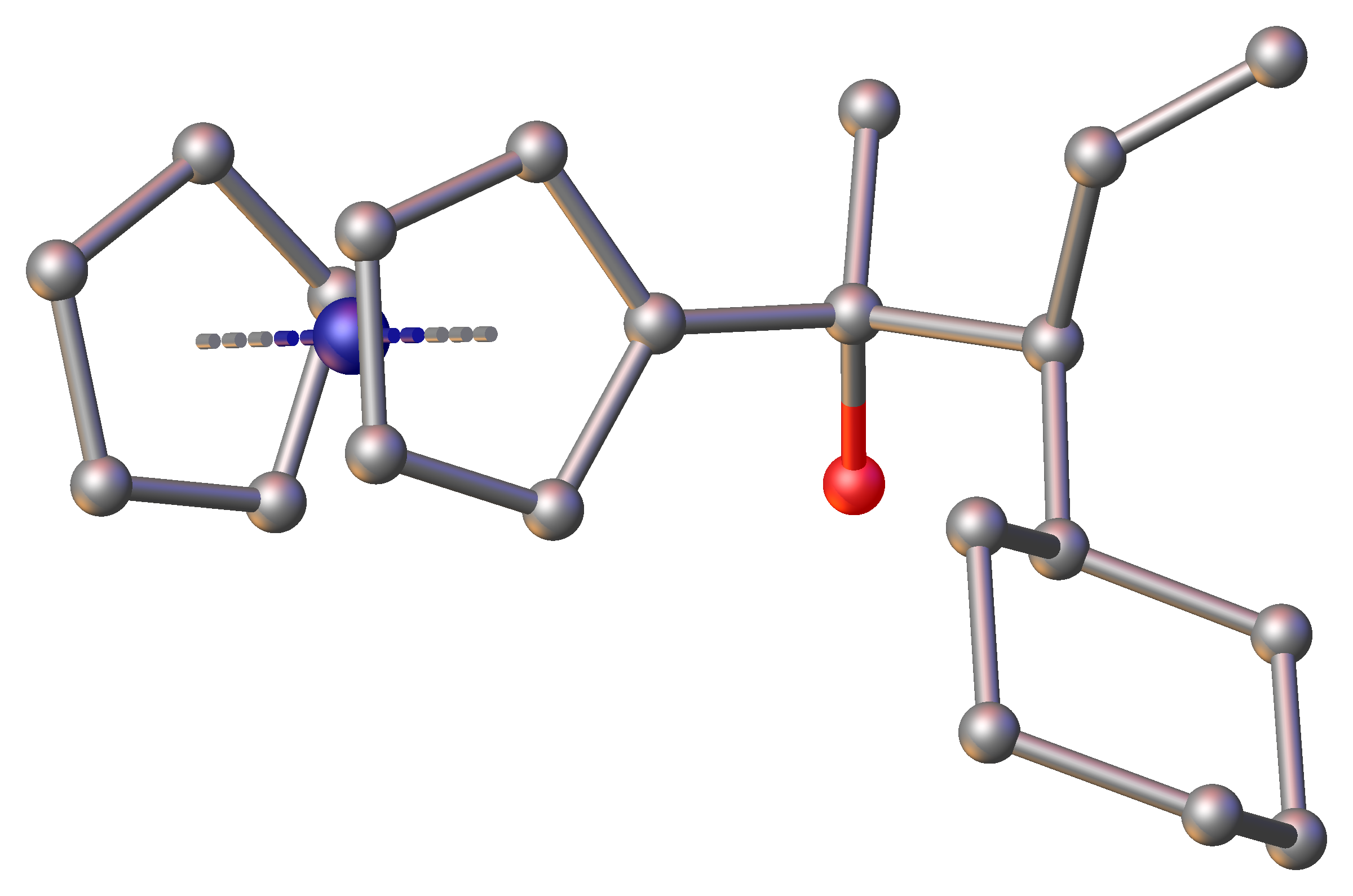


**Experimental.** Single translucent dark orange block-shaped crystals of **SE22004** recrystallised from a mixture of petroleum ether and dichloromethane by slow evaporation. A suitable crystal with dimensions 0.37 × 0.27 × 0.14 mm^3^ was selected and mounted on a MITIGEN holder in Paratone oil on a Rigaku Oxford Diffraction XCalibur diffractometer. The crystal was kept at a steady *T* = 120.00(11) K during data collection. The structure was solved with the **ShelXT** 2018/2 (Sheldrick, 2018) solution program using dual methods and by using **Olex2** 1.5-beta (Dolomanov et al., 2009) as the graphical interface. The model was refined with **olex2.refine** 1.5-beta (Bourhis et al., 2015) using full matrix least squares minimisation on ***F*^2^**.

**Crystal Data.**  C_21_H_28_FeO, *M_r_* = 352.301, monoclinic, *P*2_1_/*c* (No. 14), a = 9.7712(1) Å, b = 18.1209(2) Å, c = 10.2807(1) Å, *β* = 102.609(1)^°^, *α* = *γ* = 90^°^, *V* = 1776.43(3) Å^3^, *T* = 120.00(11) K, *Z* = 4, *Z'* = 1, *μ*(Mo K*_α_*) = 0.851, 56276 reflections measured, 6290 unique (R_int_ = 0.0358) which were used in all calculations. The final *wR_2_* was 0.0404 (all data) and *R_1_* was 0.0231 (I≥2 *σ*(I)).

| **Compound** | **SE22004** |
| --- | --- |
|  |  |
| Formula | C_21_H_28_FeO |
| *D_calc._*/ g cm^-3^ | 1.317 |
| *μ*/mm^-1^ | 0.851 |
| Formula Weight | 352.301 |
| Colour | translucent dark orange |
| Shape | block-shaped |
| Size/mm^3^ | 0.37×0.27×0.14 |
| *T*/K | 120.00(11) |
| Crystal System | monoclinic |
| Space Group | *P*2_1_/*c* |
| *a*/Å | 9.7712(1) |
| *b*/Å | 18.1209(2) |
| *c*/Å | 10.2807(1) |
| *α*/^°^ | 90 |
| *β*/^°^ | 102.609(1) |
| *γ*/^°^ | 90 |
| V/Å^3^ | 1776.43(3) |
| *Z* | 4 |
| *Z'* | 1 |
| Wavelength/Å | 0.71073 |
| Radiation type | Mo K*_α_* |
| *Θ_min_*/^°^ | 3.44 |
| *Θ_max_*/^°^ | 32.81 |
| Measured Refl's. | 56276 |
| Indep't Refl's | 6290 |
| Refl's I≥2 *σ*(I) | 5715 |
| *R*_int_ | 0.0358 |
| Parameters | 460 |
| Restraints | 0 |
| Largest Peak | 0.2623 |
| Deepest Hole | -0.2935 |
| GooF | 1.0728 |
| *wR_2_* (all data) | 0.0404 |
| *wR_2_* | 0.0389 |
| *R_1_* (all data) | 0.0280 |
| *R_1_* | 0.0231 |

**Structure Quality Indicators**

| **Reflections:** | 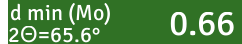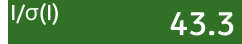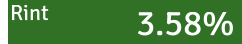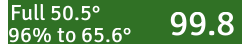 |
| --- | --- |
| **Refinement:** | 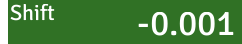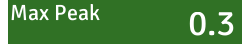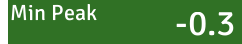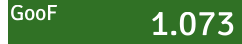 |

A translucent dark orange block-shaped-shaped crystal with dimensions 0.37 × 0.27 × 0.14 mm^3^ was mounted on a MITIGEN holder in Paratone oil. Data were collected using a Rigaku Oxford Diffraction XCalibur diffractometer equipped with an Oxford Cryosystems Cryostream 700+ low-temperature device operating at *T* = 120.00(11) K.

Data were measured using *ω* scans with Mo K*_α_* radiation. The diffraction pattern was indexed and the total number of runs and images was based on the strategy calculation from the program CrysAlisPro 1.171.41.99a (Rigaku OD, 2021). The maximum resolution that was achieved was *Θ* = 32.81^°^ (0.66 Å).

The unit cell was refined using CrysAlisPro 1.171.41.99a (Rigaku OD, 2021) on 24383 reflections, 43% of the observed reflections.

Data reduction, scaling and absorption corrections were performed using CrysAlisPro 1.171.41.99a (Rigaku OD, 2021). The final completeness is 99.78 % out to 32.81^°^ in *Θ*. A multi-scan absorption correction was performed using CrysAlisPro 1.171.41.99a (Rigaku Oxford Diffraction, 2021) Empirical absorption correction using spherical harmonics, implemented in SCALE3 ABSPACK scaling algorithm.. The absorption coefficient *μ* of this material is 0.851 mm^-1^ at this wavelength (*λ* = 0.71073Å) and the minimum and maximum transmissions are 0.889 and 1.000.

The structure was solved and the space group *P*2_1_/*c* (# 14) determined by the ShelXT 2018/2 (Sheldrick, 2018) structure solution program using using dual methods and refined by full matrix least squares minimisation on ***F*^2^** using version of **olex2.refine** 1.5-beta (Bourhis et al., 2015). All non-hydrogen atoms were refined anisotropically. Hydrogen atom positions were calculated geometrically and refined using the riding model.

*_refine_special_details*: H atoms were identified from a difference map and freely refined.The NoSpherA2 routine of Olex2 was used for final refinement and details are given elsewhere.


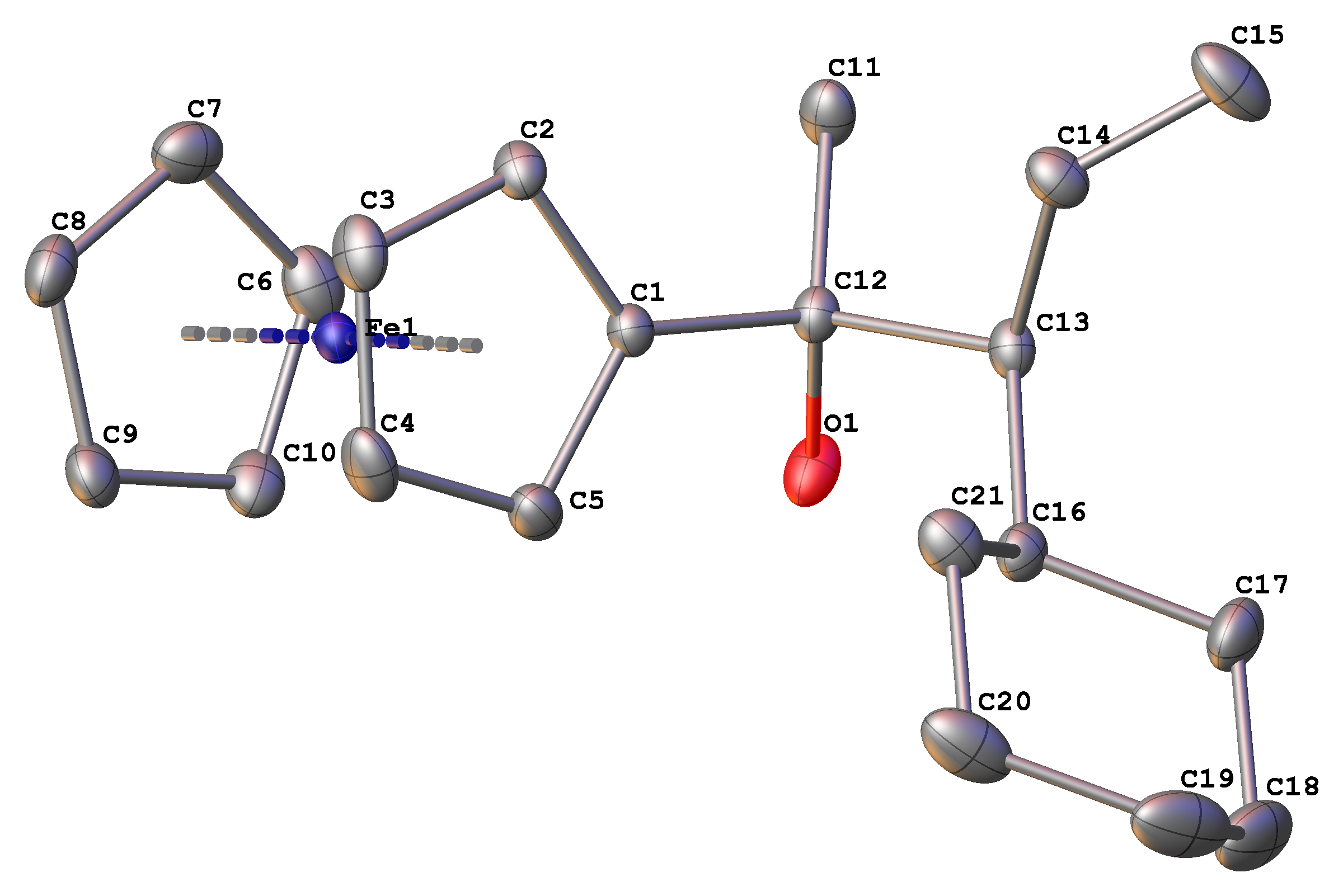


**Figure 1**: The molecular structure of SE22004. Displacement ellipsoids are at the 50% probability level and H atoms are not shown.

**Data Plots: Diffraction Data**

| 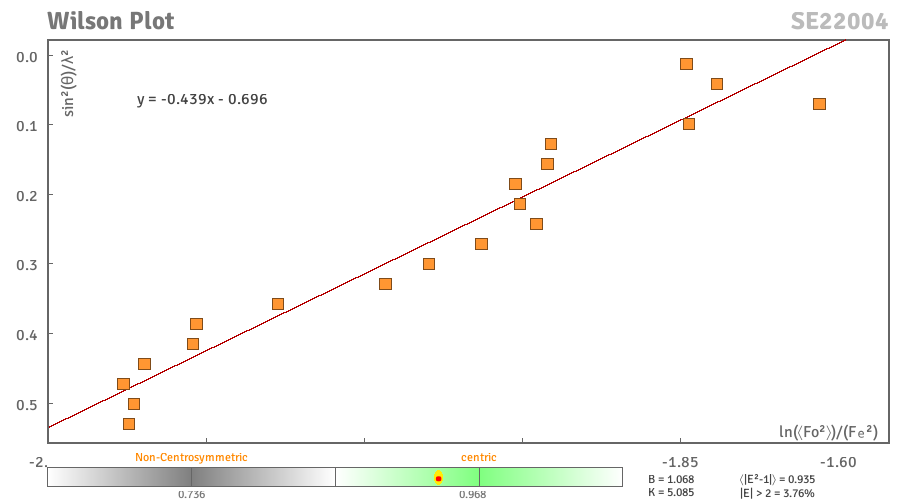 | 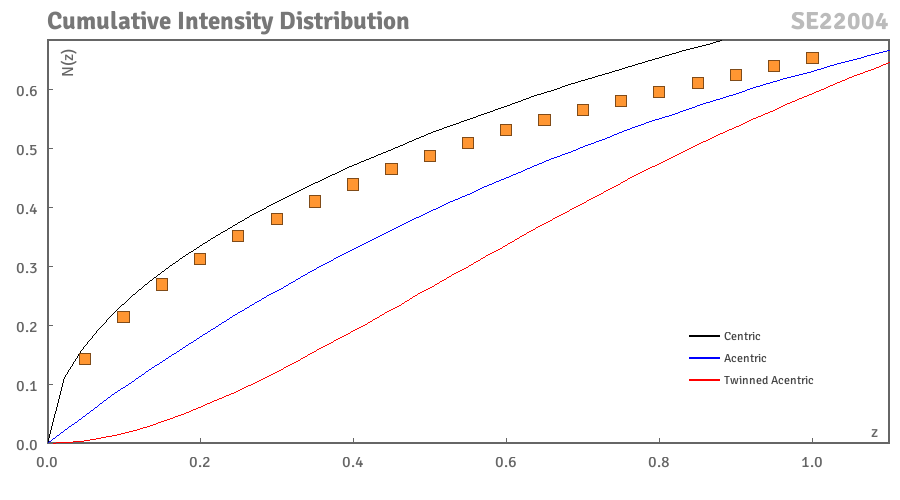 |
| --- | --- |
| 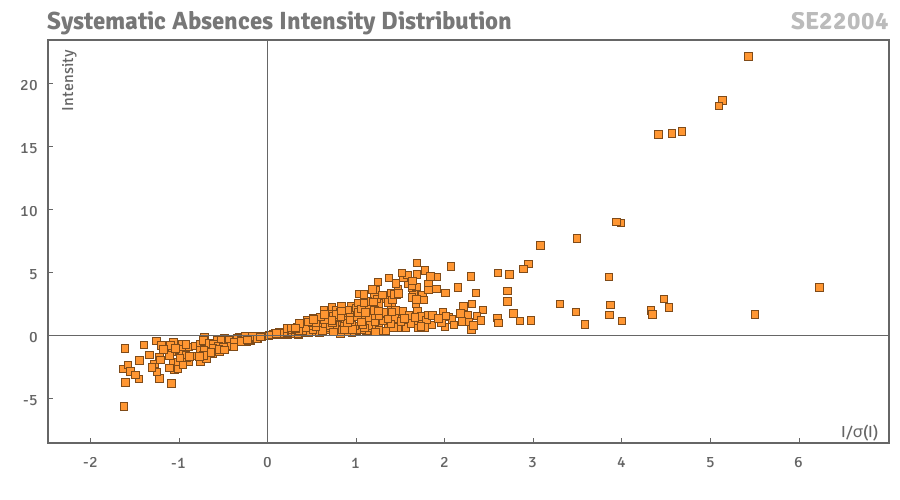 | 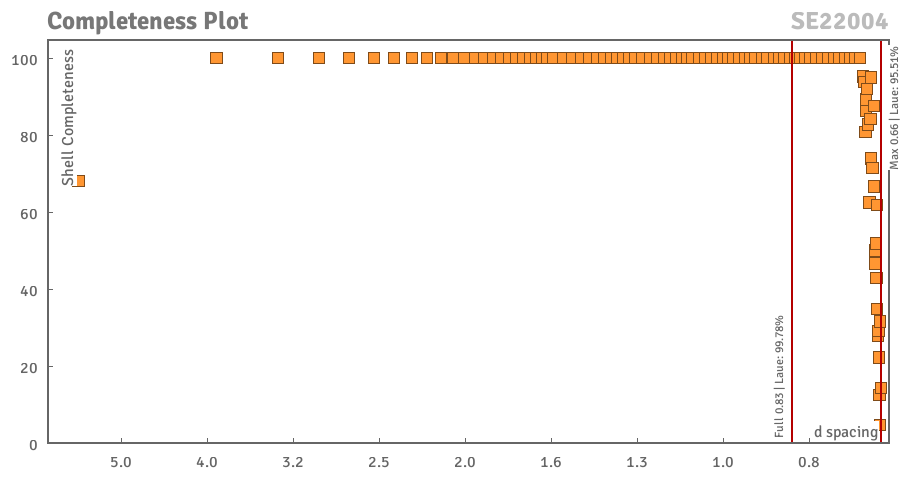 |
| 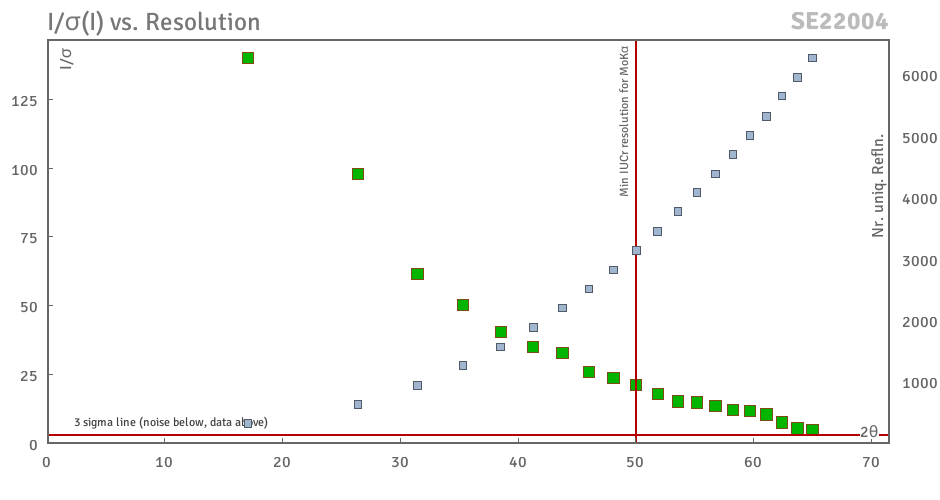 |  |

**Data Plots: Refinement and Data**

| 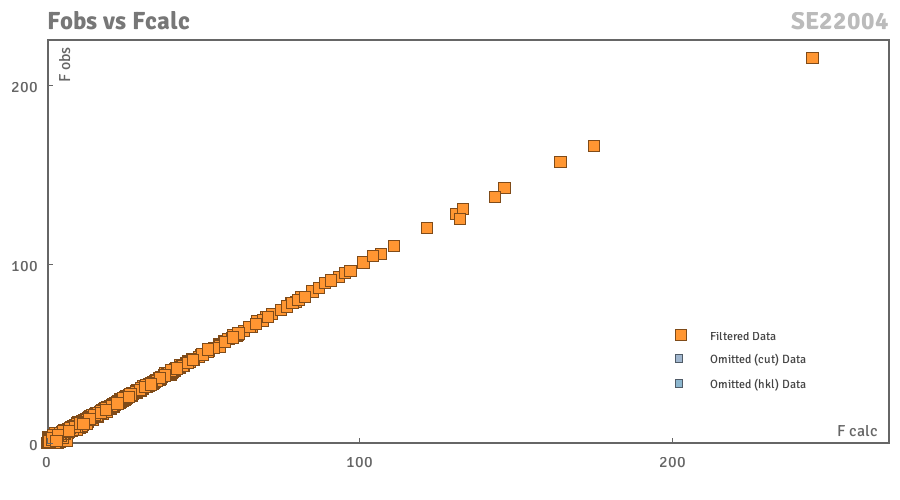 | 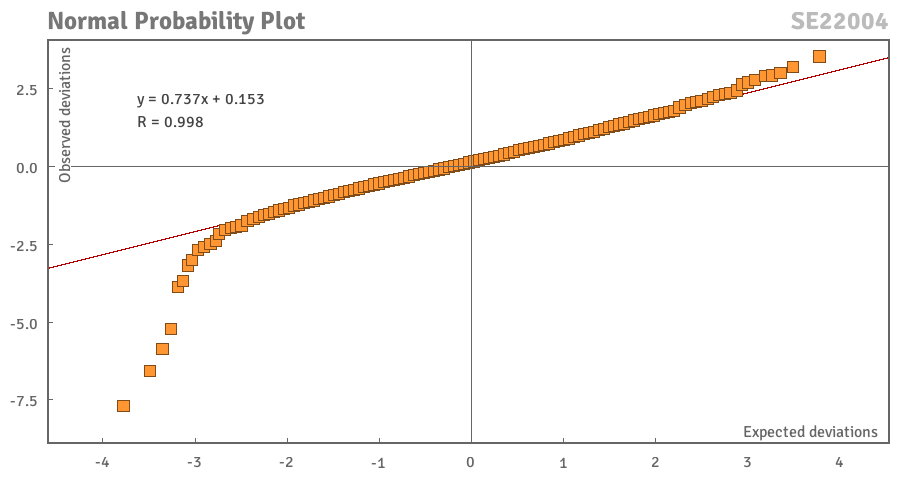 |
| --- | --- |

**Reflection Statistics**

| Total reflections (after filtering) | 57152 | Unique reflections | 6290 |
| --- | --- | --- | --- |
| Completeness | 0.955 | Mean I/*σ* | 33.28 |
| hkl_max_ collected | (14, 27, 15) | hkl_min_ collected | (-14, -27, -14) |
| hkl_max_ used | (14, 27, 15) | hkl_min_ used | (-14, 0, 0) |
| Lim d_max_ collected | 100.0 | Lim d_min_ collected | 0.36 |
| d_max_ used | 6.04 | d_min_ used | 0.66 |
| Friedel pairs | 11202 | Friedel pairs merged | 1 |
| Inconsistent equivalents | 3 | R_int_ | 0.0358 |
| R_sigma_ | 0.0231 | Intensity transformed | 0 |
| Omitted reflections | 0 | Omitted by user (OMIT hkl) | 0 |
| Multiplicity | (6297, 8157, 4693, 2454, 1183, 424, 205, 58, 24, 5, 2) | Maximum multiplicity | 36 |
| Removed systematic absences | 876 | Filtered off (Shel/OMIT) | 0 |

**Table 1**: Fractional Atomic Coordinates (×10^4^) and Equivalent Isotropic Displacement Parameters (Å^2^×10^3^) for **SE22004**. *U_eq_* is defined as 1/3 of the trace of the orthogonalised *U_ij_*.

| **Atom** | **x** | **y** | **z** | ***U_eq_*** |
| --- | --- | --- | --- | --- |
| Fe1 | 1149.02(10) | 3463.54(5) | 2565.46(10) | 11.60(3) |
| O1 | 812.2(6) | 5260.1(3) | 1214.6(5) | 18.84(11) |
| C1 | 2086.3(7) | 4484.3(4) | 2944.1(7) | 12.16(12) |
| C2 | 2183.3(7) | 4078.6(4) | 4155.6(7) | 14.91(13) |
| C3 | 2915.9(8) | 3407.3(4) | 4058.5(8) | 19.41(14) |
| C4 | 3272.6(8) | 3390.6(4) | 2789.3(8) | 20.71(15) |
| C5 | 2755.8(7) | 4050.2(4) | 2099.6(8) | 17.08(13) |
| C6 | -981.2(8) | 3565.8(4) | 1826.3(8) | 21.38(15) |
| C7 | -701.3(8) | 3084.1(4) | 2950.5(8) | 22.38(15) |
| C8 | 127.2(8) | 2490.0(4) | 2648.9(8) | 20.95(15) |
| C9 | 360.9(8) | 2603.3(4) | 1346.5(8) | 19.57(14) |
| C10 | -321.7(8) | 3269.8(4) | 837.4(8) | 20.01(15) |
| C11 | 316.6(8) | 5398.8(4) | 3397.7(8) | 18.15(14) |
| C12 | 1442.1(7) | 5236.5(4) | 2611.9(7) | 12.71(12) |
| C13 | 2542.3(7) | 5871.3(4) | 2841.8(7) | 13.52(12) |
| C14 | 3122.5(7) | 6009.2(4) | 4300.5(7) | 17.84(14) |
| C15 | 3159.8(9) | 6666.7(5) | 4880.7(10) | 27.67(18) |
| C16 | 3728.2(7) | 5784.9(4) | 2063.3(7) | 15.19(13) |
| C17 | 4118.2(9) | 6541.8(4) | 1586.6(9) | 23.94(16) |
| C18 | 5268.7(10) | 6488.5(6) | 796.9(10) | 33.7(2) |
| C19 | 6572.1(10) | 6104.0(6) | 1600.1(10) | 36.2(2) |
| C20 | 6192.7(9) | 5345.1(5) | 2059.9(10) | 30.58(19) |
| C21 | 5053.0(7) | 5405.0(4) | 2859.3(8) | 19.63(14) |

**Table 2**: Anisotropic Displacement Parameters (×10^4^) for **SE22004**. The anisotropic displacement factor exponent takes the form: *-2π^2^[h^2^a*^2^ × U_11_+ ... +2hka* × b* × U_12_]*

| **Atom** | ***U_11_*** | ***U_22_*** | ***U_33_*** | ***U_23_*** | ***U_13_*** | ***U_12_*** |
| --- | --- | --- | --- | --- | --- | --- |
| Fe1 | 12.95(5) | 9.63(4) | 11.83(5) | -0.53(3) | 1.88(3) | -0.39(3) |
| O1 | 19.4(3) | 17.6(3) | 16.2(2) | -4.4(2) | -3.3(2) | 4.4(2) |
| H1 | 81(10) | 24(7) | 46(8) | -31(7) | -10(7) | 3(6) |
| C1 | 11.9(3) | 11.0(3) | 13.1(3) | -0.3(2) | 1.6(2) | -0.5(2) |
| C2 | 16.8(3) | 13.3(3) | 13.0(3) | 1.0(2) | -0.3(2) | 0.4(2) |
| H2 | 55(7) | 43(7) | 14(5) | 9(5) | 21(5) | -10(5) |
| C3 | 18.4(3) | 13.9(3) | 22.8(4) | 3.0(3) | -2.6(3) | 1.7(3) |
| H3 | 59(8) | 29(6) | 41(7) | 7(5) | -14(6) | 29(5) |
| C4 | 16.2(3) | 14.7(3) | 31.5(4) | 2.8(2) | 6.0(3) | -4.5(3) |
| H4 | 48(8) | 39(7) | 88(10) | 28(6) | 29(7) | -11(6) |
| C5 | 17.4(3) | 15.2(3) | 20.2(4) | -2.2(2) | 7.8(3) | -2.8(3) |
| H5 | 68(8) | 42(7) | 25(6) | -9(6) | 25(6) | 1(5) |
| C6 | 16.2(3) | 19.0(4) | 26.4(4) | -0.4(3) | -1.0(3) | -3.9(3) |
| H6 | 38(7) | 21(6) | 82(9) | 19(5) | 1(6) | -7(6) |
| C7 | 19.4(3) | 27.3(4) | 21.5(4) | -6.8(3) | 6.9(3) | -3.1(3) |
| H7 | 49(8) | 92(10) | 31(7) | -8(7) | 28(6) | -6(6) |
| C8 | 24.3(4) | 15.5(3) | 21.7(4) | -6.1(3) | 2.1(3) | 2.6(3) |
| H8 | 55(7) | 35(7) | 47(7) | 3(6) | -1(6) | 24(6) |
| C9 | 23.9(4) | 14.8(3) | 19.2(3) | -2.9(3) | 2.8(3) | -4.2(3) |
| H9 | 53(7) | 40(7) | 48(7) | 9(6) | 20(6) | -22(6) |
| C10 | 23.6(4) | 18.5(3) | 15.2(3) | -3.8(3) | -1.5(3) | -0.3(3) |
| H10 | 72(9) | 50(8) | 20(6) | -2(6) | 1(6) | 20(5) |
| C11 | 14.4(3) | 14.7(3) | 25.8(4) | 1.6(3) | 5.5(3) | 0.3(3) |
| H11a | 41(7) | 55(8) | 27(7) | -6(6) | 2(5) | -8(6) |
| H11b | 29(6) | 40(7) | 73(9) | -15(5) | 16(6) | -7(6) |
| H11c | 47(7) | 24(6) | 73(9) | 18(5) | 21(6) | 11(6) |
| C12 | 12.3(3) | 11.2(3) | 13.5(3) | -0.7(2) | 0.4(2) | 1.0(2) |
| C13 | 13.0(3) | 10.5(3) | 15.5(3) | 0.1(2) | -0.1(2) | 0.5(2) |
| H13 | 34(6) | 19(5) | 47(7) | 10(5) | 4(5) | 7(5) |
| C14 | 15.9(3) | 19.5(3) | 17.0(3) | -0.6(3) | 1.2(3) | -4.3(3) |
| H14 | 59(8) | 28(6) | 43(7) | 13(5) | -5(6) | 8(5) |
| C15 | 20.5(4) | 29.7(5) | 31.6(5) | -1.2(3) | 2.9(3) | -17.1(4) |
| H15a | 64(9) | 31(7) | 79(10) | 22(6) | -7(7) | -4(7) |
| H15b | 48(8) | 91(10) | 29(7) | -3(7) | -1(6) | -29(7) |
| C16 | 15.2(3) | 14.3(3) | 15.3(3) | -4.1(2) | 1.7(2) | -0.9(2) |
| H16 | 44(7) | 42(7) | 20(6) | -16(5) | 6(5) | -9(5) |
| C17 | 28.0(4) | 18.9(4) | 24.9(4) | -9.7(3) | 5.7(3) | 1.6(3) |
| H17a | 59(8) | 49(8) | 44(7) | 3(6) | -5(6) | 24(6) |
| H17b | 62(8) | 27(6) | 40(7) | -15(6) | 17(6) | -11(5) |
| C18 | 39.1(5) | 36.9(5) | 28.2(5) | -23.1(4) | 14.2(4) | -4.5(4) |
| H18a | 90(10) | 57(9) | 62(9) | -39(8) | 30(8) | 3(7) |
| H18b | 63(8) | 68(9) | 32(7) | -14(7) | 8(6) | -11(7) |
| C19 | 25.0(4) | 55.0(6) | 32.4(5) | -20.1(4) | 14.7(4) | -17.8(4) |
| H19a | 51(8) | 116(12) | 70(9) | -35(8) | 47(7) | -41(8) |
| H19b | 65(9) | 62(9) | 48(8) | -26(7) | 2(7) | -29(7) |
| C20 | 17.1(4) | 41.6(5) | 33.4(5) | -3.0(3) | 6.4(3) | -17.1(4) |
| H20a | 22(6) | 77(9) | 67(9) | 8(6) | -2(6) | -3(7) |
| H20b | 52(8) | 65(9) | 53(8) | -10(6) | 11(6) | -35(7) |
| C21 | 13.5(3) | 21.6(4) | 22.6(4) | -0.9(3) | 1.3(3) | -4.2(3) |
| H21a | 39(7) | 26(6) | 59(8) | 1(5) | 7(6) | 10(6) |
| H21b | 30(6) | 51(7) | 28(6) | -11(5) | -4(5) | -16(5) |

**Table 3**: Bond Lengths in Å for **SE22004**.

| **Atom** | **Atom** | **Length/Å** | |
| --- | --- | --- | --- |
| Fe1 | C1 | 2.0630(6) |  |
| Fe1 | C2 | 2.0530(7) |  |
| Fe1 | C3 | 2.0470(7) |  |
| Fe1 | C4 | 2.0415(7) |  |
| Fe1 | C5 | 2.0378(7) |  |
| Fe1 | C6 | 2.0625(7) |  |
| Fe1 | C7 | 2.0520(8) |  |
| Fe1 | C8 | 2.0381(7) |  |
| Fe1 | C9 | 2.0426(7) |  |
| Fe1 | C10 | 2.0567(7) |  |
| O1 | C12 | 1.4346(8) |  |
| C1 | C2 | 1.4314(9) |  |
| C1 | C5 | 1.4312(10) |  |
| C1 | C12 | 1.5089(9) |  |
| C2 | C3 | 1.4259(10) |  |
| C3 | C4 | 1.4225(11) |  |
| C4 | C5 | 1.4248(10) |  |
| C6 | C7 | 1.4263(11) |  |
| C6 | C10 | 1.4221(11) |  |
| C7 | C8 | 1.4213(11) |  |
| C8 | C9 | 1.4215(11) |  |
| C9 | C10 | 1.4230(11) |  |
| C11 | C12 | 1.5286(10) |  |
| C12 | C13 | 1.5569(9) |  |
| C13 | C14 | 1.5038(10) |  |
| C13 | C16 | 1.5533(10) |  |
| C14 | C15 | 1.3293(11) |  |
| C16 | C17 | 1.5325(10) |  |
| C16 | C21 | 1.5352(10) |  |
| C17 | C18 | 1.5266(13) |  |
| C18 | C19 | 1.5266(15) |  |
| C19 | C20 | 1.5260(14) |  |
| C20 | C21 | 1.5254(11) |  |

**Table 4**: Bond Angles in ^°^ for **SE22004**.

| **Atom** | **Atom** | **Atom** | **Angle/^°^** | |
| --- | --- | --- | --- | --- |
| C2 | Fe1 | C1 | 40.70(3) |  |
| C3 | Fe1 | C1 | 68.66(3) |  |
| C3 | Fe1 | C2 | 40.70(3) |  |
| C4 | Fe1 | C1 | 68.80(3) |  |
| C4 | Fe1 | C2 | 68.52(3) |  |
| C4 | Fe1 | C3 | 40.72(3) |  |
| C5 | Fe1 | C1 | 40.85(3) |  |
| C5 | Fe1 | C2 | 68.49(3) |  |
| C5 | Fe1 | C3 | 68.61(3) |  |
| C5 | Fe1 | C4 | 40.89(3) |  |
| C6 | Fe1 | C1 | 111.08(3) |  |
| C6 | Fe1 | C2 | 121.76(3) |  |
| C6 | Fe1 | C3 | 154.02(3) |  |
| C6 | Fe1 | C4 | 165.14(3) |  |
| C6 | Fe1 | C5 | 129.34(3) |  |
| C7 | Fe1 | C1 | 129.61(3) |  |
| C7 | Fe1 | C2 | 109.25(3) |  |
| C7 | Fe1 | C3 | 118.24(3) |  |
| C7 | Fe1 | C4 | 150.84(3) |  |
| C7 | Fe1 | C5 | 167.58(3) |  |
| C7 | Fe1 | C6 | 40.57(3) |  |
| C8 | Fe1 | C1 | 165.82(3) |  |
| C8 | Fe1 | C2 | 126.65(3) |  |
| C8 | Fe1 | C3 | 105.43(3) |  |
| C8 | Fe1 | C4 | 115.75(3) |  |
| C8 | Fe1 | C5 | 150.58(3) |  |
| C8 | Fe1 | C6 | 68.22(3) |  |
| C8 | Fe1 | C7 | 40.67(3) |  |
| C9 | Fe1 | C1 | 153.18(3) |  |
| C9 | Fe1 | C2 | 163.14(3) |  |
| C9 | Fe1 | C3 | 124.43(3) |  |
| C9 | Fe1 | C4 | 104.92(3) |  |
| C9 | Fe1 | C5 | 117.42(3) |  |
| C9 | Fe1 | C6 | 68.13(3) |  |
| C9 | Fe1 | C7 | 68.44(3) |  |
| C9 | Fe1 | C8 | 40.77(3) |  |
| C10 | Fe1 | C1 | 120.92(3) |  |
| C10 | Fe1 | C2 | 155.53(3) |  |
| C10 | Fe1 | C3 | 162.77(3) |  |
| C10 | Fe1 | C4 | 125.99(3) |  |
| C10 | Fe1 | C5 | 108.36(3) |  |
| C10 | Fe1 | C6 | 40.39(3) |  |
| C10 | Fe1 | C7 | 68.31(3) |  |
| C10 | Fe1 | C8 | 68.42(3) |  |
| C10 | Fe1 | C9 | 40.62(3) |  |
| C2 | C1 | Fe1 | 69.27(4) |  |
| C5 | C1 | Fe1 | 68.63(4) |  |
| C5 | C1 | C2 | 107.06(6) |  |
| C12 | C1 | Fe1 | 128.40(4) |  |
| C12 | C1 | C2 | 127.30(6) |  |
| C12 | C1 | C5 | 125.63(6) |  |
| C1 | C2 | Fe1 | 70.03(4) |  |
| C3 | C2 | Fe1 | 69.42(4) |  |
| C3 | C2 | C1 | 108.43(6) |  |
| C2 | C3 | Fe1 | 69.87(4) |  |
| C4 | C3 | Fe1 | 69.43(4) |  |
| C4 | C3 | C2 | 108.04(6) |  |
| C3 | C4 | Fe1 | 69.85(4) |  |
| C5 | C4 | Fe1 | 69.42(4) |  |
| C5 | C4 | C3 | 107.90(6) |  |
| C1 | C5 | Fe1 | 70.52(4) |  |
| C4 | C5 | Fe1 | 69.70(4) |  |
| C4 | C5 | C1 | 108.57(7) |  |
| C7 | C6 | Fe1 | 69.32(4) |  |
| C10 | C6 | Fe1 | 69.58(4) |  |
| C10 | C6 | C7 | 108.17(7) |  |
| C6 | C7 | Fe1 | 70.11(4) |  |
| C8 | C7 | Fe1 | 69.14(4) |  |
| C8 | C7 | C6 | 107.72(7) |  |
| C7 | C8 | Fe1 | 70.19(4) |  |
| C9 | C8 | Fe1 | 69.78(4) |  |
| C9 | C8 | C7 | 108.20(7) |  |
| C8 | C9 | Fe1 | 69.45(4) |  |
| C10 | C9 | Fe1 | 70.22(4) |  |
| C10 | C9 | C8 | 108.07(7) |  |
| C6 | C10 | Fe1 | 70.02(4) |  |
| C9 | C10 | Fe1 | 69.16(4) |  |
| C9 | C10 | C6 | 107.84(7) |  |
| C1 | C12 | O1 | 108.67(5) |  |
| C11 | C12 | O1 | 108.82(6) |  |
| C11 | C12 | C1 | 111.53(6) |  |
| C13 | C12 | O1 | 105.13(5) |  |
| C13 | C12 | C1 | 113.14(5) |  |
| C13 | C12 | C11 | 109.28(6) |  |
| C14 | C13 | C12 | 111.82(6) |  |
| C16 | C13 | C12 | 114.54(5) |  |
| C16 | C13 | C14 | 111.67(6) |  |
| C15 | C14 | C13 | 124.33(8) |  |
| C17 | C16 | C13 | 109.88(6) |  |
| C21 | C16 | C13 | 114.05(6) |  |
| C21 | C16 | C17 | 109.41(6) |  |
| C18 | C17 | C16 | 112.14(7) |  |
| C19 | C18 | C17 | 111.44(8) |  |
| C20 | C19 | C18 | 110.40(7) |  |
| C21 | C20 | C19 | 110.99(8) |  |
| C20 | C21 | C16 | 111.99(7) |  |

**Table 5**: Torsion Angles in ^°^ for **SE22004**.

| **Atom** | **Atom** | **Atom** | **Atom** | **Angle/^°^** |  |
| --- | --- | --- | --- | --- | --- |
| Fe1 | C1 | C2 | C3 | 58.98(4) | |
| Fe1 | C1 | C5 | C4 | -59.53(4) | |
| Fe1 | C1 | C12 | O1 | -53.96(6) | |
| Fe1 | C1 | C12 | C11 | 66.00(6) | |
| Fe1 | C1 | C12 | C13 | -170.33(6) | |
| Fe1 | C2 | C1 | C5 | -58.42(4) | |
| Fe1 | C2 | C1 | C12 | 123.13(4) | |
| Fe1 | C2 | C3 | C4 | 59.14(5) | |
| Fe1 | C3 | C2 | C1 | -59.36(4) | |
| Fe1 | C3 | C4 | C5 | 59.20(4) | |
| Fe1 | C4 | C3 | C2 | -59.42(4) | |
| Fe1 | C4 | C5 | C1 | 60.04(4) | |
| Fe1 | C5 | C1 | C2 | 58.83(4) | |
| Fe1 | C5 | C1 | C12 | -122.69(4) | |
| Fe1 | C5 | C4 | C3 | -59.47(5) | |
| Fe1 | C6 | C7 | C8 | -59.13(5) | |
| Fe1 | C6 | C10 | C9 | 59.02(5) | |
| Fe1 | C7 | C6 | C10 | 58.92(5) | |
| Fe1 | C7 | C8 | C9 | -59.66(5) | |
| Fe1 | C8 | C7 | C6 | 59.74(5) | |
| Fe1 | C8 | C9 | C10 | -59.83(5) | |
| Fe1 | C9 | C8 | C7 | 59.92(5) | |
| Fe1 | C9 | C10 | C6 | -59.57(5) | |
| Fe1 | C10 | C6 | C7 | -58.76(5) | |
| Fe1 | C10 | C9 | C8 | 59.35(5) | |
| O1 | C12 | C1 | C2 | -146.03(5) | |
| O1 | C12 | C1 | C5 | 35.80(7) | |
| O1 | C12 | C13 | C14 | 169.96(5) | |
| O1 | C12 | C13 | C16 | -61.72(6) | |
| C1 | C2 | C3 | C4 | -0.22(6) | |
| C1 | C5 | C4 | C3 | 0.57(6) | |
| C1 | C12 | C13 | C14 | -71.60(6) | |
| C1 | C12 | C13 | C16 | 56.73(6) | |
| C2 | C3 | C4 | C5 | -0.22(6) | |
| C6 | C7 | C8 | C9 | 0.08(7) | |
| C6 | C10 | C9 | C8 | -0.22(7) | |
| C7 | C8 | C9 | C10 | 0.09(7) | |
| C11 | C12 | C13 | C14 | 53.31(6) | |
| C11 | C12 | C13 | C16 | -178.37(5) | |
| C12 | C13 | C14 | C15 | -127.92(7) | |
| C12 | C13 | C16 | C17 | 143.00(6) | |
| C12 | C13 | C16 | C21 | -93.74(6) | |
| C13 | C16 | C17 | C18 | -179.23(6) | |
| C13 | C16 | C21 | C20 | -179.05(6) | |
| C16 | C17 | C18 | C19 | -55.93(8) | |
| C16 | C21 | C20 | C19 | 57.14(7) | |
| C17 | C18 | C19 | C20 | 55.67(8) | |
| C18 | C19 | C20 | C21 | -56.20(8) | |

**Table 6**: Hydrogen Fractional Atomic Coordinates (×10^4^) and Equivalent Isotropic Displacement Parameters (Å^2^×10^3^) for **SE22004**. *U_eq_* is defined as 1/3 of the trace of the orthogonalised *U_ij_*.

| **Atom** | **x** | **y** | **z** | ***U_eq_*** |
| --- | --- | --- | --- | --- |
| H1 | 359(14) | 4835(6) | 1013(12) | 54(4) |
| H2 | 1756(11) | 4237(5) | 5005(9) | 35(3) |
| H3 | 3138(11) | 2985(5) | 4832(10) | 47(3) |
| H4 | 3801(11) | 2951(6) | 2386(12) | 56(4) |
| H5 | 2850(11) | 4206(6) | 1118(10) | 43(3) |
| H6 | -1573(11) | 4069(5) | 1730(12) | 49(3) |
| H7 | -1031(11) | 3176(7) | 3878(10) | 54(3) |
| H8 | 532(11) | 2037(6) | 3304(11) | 47(3) |
| H9 | 975(11) | 2255(6) | 842(10) | 45(3) |
| H10 | -329(12) | 3513(6) | -124(10) | 48(3) |
| H11a | 752(11) | 5427(6) | 4459(10) | 42(3) |
| H11b | -487(10) | 4982(6) | 3219(12) | 47(3) |
| H11c | -194(11) | 5921(6) | 3082(12) | 47(3) |
| H13 | 1930(10) | 6367(5) | 2436(10) | 34(3) |
| H14 | 3572(11) | 5539(5) | 4901(10) | 46(3) |
| H15a | 2730(12) | 7152(6) | 4325(12) | 61(4) |
| H15b | 3630(12) | 6742(7) | 5928(11) | 57(4) |
| H16 | 3310(10) | 5449(5) | 1169(9) | 36(3) |
| H17a | 3183(12) | 6815(6) | 1002(11) | 53(3) |
| H17b | 4471(12) | 6895(6) | 2457(10) | 42(3) |
| H18a | 5501(13) | 7047(7) | 476(12) | 67(4) |
| H18b | 4879(12) | 6176(7) | -122(11) | 55(3) |
| H19a | 7390(12) | 6055(8) | 1042(12) | 74(4) |
| H19b | 7008(13) | 6430(7) | 2481(12) | 60(4) |
| H20a | 7126(11) | 5068(7) | 2658(12) | 57(4) |
| H20b | 5844(11) | 5000(7) | 1186(11) | 57(3) |
| H21a | 4809(10) | 4856(6) | 3206(11) | 42(3) |
| H21b | 5477(10) | 5724(6) | 3752(10) | 38(3) |

**Citations**

**CrysAlisPro** (Rigaku, V1.171.41.99a, 2021)

CrysAlisPro (ROD), Rigaku Oxford Diffraction, Poland (?).

L.J. Bourhis and O.V. Dolomanov and R.J. Gildea and J.A.K. Howard and H. Puschmann, The Anatomy of a Comprehensive Constrained, Restrained, Refinement Program for the Modern Computing Environment - Olex2 Disected, *Acta Cryst. A*, (2015), **A71**, 59-71.

O.V. Dolomanov and L.J. Bourhis and R.J. Gildea and J.A.K. Howard and H. Puschmann, Olex2: A complete structure solution, refinement and analysis program, *J. Appl. Cryst.*, (2009), **42**, 339-341.

Sheldrick, G.M., ShelXT-Integrated space-group and crystal-structure determination, *Acta Cryst.*, (2015), **A71**, 3-8.

#===============================================================================

# PLATON/CHECK-(181221) versus check.def version 211218, Entry: SE22004

# Data: SE22004.cif - Type: CIF Bond Precision C-C = 0.0011 A

# Refl: SE22004.fcf - Type: LIST4 Temp = 120 K

# Audit:OLEX2 1.5-BETA (COMPILED 2021.12.09 SVN.R5202D8CF FOR OLEXSYS, GUI SVN.R

# Refin:OLEX2.REFINE 1.5-BETA (BOURHIS ET AL., 2015)

# X-ray MoKa R(int) = 0.036, wR2/R(int) = 1.1, Nref/Npar = 13.7

# Cell 9.7712(1) 18.1209(2) 10.2807(1) 90 102.609(1) 90

# Wavelength 0.71073 Volume Reported 1776.43(3) Calculated 1776.43(3)

# SpaceGroup from Symmetry P 21/c Hall: -P 2ybc monoclinic

# Reported P 1 21/c 1 -P 2ybc monoclinic

# MoietyFormula C21 H28 Fe O

# Reported C21 H28 Fe O

# SumFormula C21 H28 Fe O

# Reported C21 H28 Fe O

# Mr = 352.28[Calc], 352.30[Rep] Volume/NonHatoms = 19 Ang**3

# Dx,gcm-3 = 1.317[Calc], 1.317[Rep]

# Z = 4[Calc], 4[Rep]

# Mu (mm-1) = 0.851[Calc], 0.851[Rep] Xtal Size = 0.140x0.270x0.370 mm

# F000 = 752.0[Calc], 753.8[Rep] or F000' = 753.60[Calc]

# Reported T Limits: Tmin=0.889 Tmax=1.000 AbsCorr = MULTI-SCAN

# Calculated T Limits: Tmin=0.759 Tmin'=0.730 Tmax=0.888

# Measured HKL: Reported 56276, Embedded 0, <Mult> 0.0

# Reported Hmax= 14, Kmax= 27, Lmax= 15, Nref= 6290 , Th(max)= 32.810

# Obs in FCF Hmax= 14, Kmax= 27, Lmax= 15, Nref= 6290[ 6290], Th(max)= 32.810

# Calculated Hmax= 14, Kmax= 27, Lmax= 15, Nref= 6593 , Ratio = 0.954

# Reported Rho(min) = -0.29, Rho(max) = 0.26 e/Ang**3 (From CIF)

# Calculated Rho(min) = -0.98, Rho(max) = 0.54 e/Ang**3 (From CIF+FCF data)

# w=1/[<sup>2</sup>(Fo<sup>2</sup>)+(0.0117P)<sup>2</sup>+0.2190P], P=(Fo<sup>2</sup>+2Fc<sup>2</sup>)/3

# R= 0.0358( 5715), wR2= 0.0787( 6290), S = 2.090 (From CIF+FCF data)

# R= 0.0231( 5715), wR2= 0.0404( 6290), S = 1.073 (From FCF data only)

# R= 0.0231( 5715), wR2= 0.0404( 6290), S = 1.073, Npar= 460

#===============================================================================

# For Documentation:http://www.platonsoft.nl/CIF-VALIDATION.pdf

#===============================================================================

*

#===============================================================================

#>>> The Following Improvement and Query ALERTS were generated - (Acta-Mode) <<<

#===============================================================================

Format: alert-number_ALERT_alert-type_alert-level text

351_ALERT_3_C Long C-H (X0.96,N1.08A) C13 - H13 . 1.11 Ang.

417_ALERT_2_C Short Inter D-H..H-D H1 ..H1 . 2.13 Ang.

-x,1-y,-z = 3_565 Check

906_ALERT_3_C Large K Value in the Analysis of Variance ...... 2.199 Check

910_ALERT_3_C Missing # of FCF Reflection(s) Below Theta(Min). 7 Note

#===============================================================================

068_ALERT_1_G Reported F000 Differs from Calcd (or Missing)... Please Check

143_ALERT_4_G s.u. on c - Axis Small or Missing .............. 0.00010 Ang.

164_ALERT_4_G Nr. of Refined C-H H-Atoms in Heavy-Atom Struct. 27 Note

232_ALERT_2_G Hirshfeld Test Diff (M-X) Fe1 --C2 . 7.3 s.u.

232_ALERT_2_G Hirshfeld Test Diff (M-X) Fe1 --C3 . 7.7 s.u.

232_ALERT_2_G Hirshfeld Test Diff (M-X) Fe1 --C4 . 7.3 s.u.

232_ALERT_2_G Hirshfeld Test Diff (M-X) Fe1 --C5 . 5.3 s.u.

232_ALERT_2_G Hirshfeld Test Diff (M-X) Fe1 --C6 . 6.5 s.u.

232_ALERT_2_G Hirshfeld Test Diff (M-X) Fe1 --C7 . 5.5 s.u.

232_ALERT_2_G Hirshfeld Test Diff (M-X) Fe1 --C9 . 7.0 s.u.

232_ALERT_2_G Hirshfeld Test Diff (M-X) Fe1 --C10 . 6.3 s.u.

793_ALERT_4_G Model has Chirality at C12 (Centro SPGR) R Verify

793_ALERT_4_G Model has Chirality at C13 (Centro SPGR) S Verify

794_ALERT_5_G Tentative Bond Valency for Fe1 (II) . 2.09 Info

802_ALERT_4_G CIF Input Record(s) with more than 80 Characters 1 Info

912_ALERT_4_G Missing # of FCF Reflections Above STh/L= 0.600 296 Note

978_ALERT_2_G Number C-C Bonds with Positive Residual Density. 9 Info

979_ALERT_1_G NoSpherA2 Scattering Factors Used .............. Please Note

#===============================================================================

ALERT_Level and ALERT_Type Summary

==================================

4 ALERT_Level_C = Check. Ensure it is Not caused by an Omission or Oversight

18 ALERT_Level_G = General Info/Check that it is not Something Unexpected

2 ALERT_Type_1 CIF Construction/Syntax Error, Inconsistent or Missing Data.

10 ALERT_Type_2 Indicator that the Structure Model may be Wrong or Deficient.

3 ALERT_Type_3 Indicator that the Structure Quality may be Low.

6 ALERT_Type_4 Improvement, Methodology, Query or Suggestion.

1 ALERT_Type_5 Informative Message, Check.

#===============================================================================

1 Missing Experimental Info Issue(s) (Out of 64 Tests) - 98 % Satisfied

0 Experimental Data Related Issue(s) (Out of 35 Tests) - 100 % Satisfied

12 Structural Model Related Issue(s) (Out of 136 Tests) - 91 % Satisfied

9 Unresolved or to be Checked Issue(s) (Out of 272 Tests) - 97 % Satisfied

*
