# Supplementary material for: Boron-Catalyzed, Diastereo- and Enantioselective Allylation of Ketones with Allenes
Source: ACS Catal. 2022 Aug 22;12(17):10887–93. doi: 10.1021/acscatal.2c03158 (PMC9442582; doi:10.1021/acscatal.2c03158)

checkCIF/PLATON report

Structure factors have been supplied for datablock(s) SE22004

THIS REPORT IS FOR GUIDANCE ONLY. IF USED AS PART OF A REVIEW PROCEDURE FOR PUBLICATION, IT SHOULD NOT REPLACE THE EXPERTISE OF AN EXPERIENCED CRYSTALLOGRAPHIC REFEREE.

No syntax errors found. CIF dictionary Interpreting this report

Datablock: SE22004

Bond precision:	C-C = 0.0011 A		Wavelength=0.71073
Cell:	a=9.7712(1)	b=18.1209(2)	c=10.2807(1)
	alpha=90	beta=102.609(1)	gamma=90
Temperature:	120 K		

	Calculated	Reported
Volume	1776.43(3)	1776.43(3)
Space group	P 21/c	P 1 21/c 1
Hall group	-P 2ybc	-P 2ybc
Moiety formula	C21 H28 Fe O	C21 H28 Fe O
Sum formula	C21 H28 Fe O	C21 H28 Fe O
Mr	352.28	352.30
Dx,g cm-3	1.317	1.317
Z	4	4
Mu (mm-1)	0.851	0.851
F000	752.0	753.8
F000’	753.60	
h,k,lmax	14,27,15	14,27,15
Nref	6593	6290
Tmin,Tmax	0.759,0.888	0.889,1.000
Tmin’	0.730	

Correction method= # Reported T Limits: Tmin=0.889 Tmax=1.000
AbsCorr = MULTI-SCAN

Data completeness= 0.954 Theta(max)= 32.810

R(reflections)= 0.0232(5715) wR2(reflections)= 0.0404(6290)

S = 1.080 Npar= 460

The following ALERTS were generated. Each ALERT has the format
test-name_ALERT_alert-type_alert-level.
Click on the hyperlinks for more details of the test.

Alert level C

```
PLAT351_ALERT_3_C Long   C-H (X0.96,N1.08A)  C13      - H13      .       1.11 Ang.
PLAT417_ALERT_2_C Short Inter D-H..H-D       H1       ..H1       .       2.13 Ang.
                                                -x,1-y,-z  =      3_565 Check
PLAT906_ALERT_3_C Large K Value in the Analysis of Variance ......      2.199 Check
PLAT910_ALERT_3_C Missing # of FCF Reflection(s) Below Theta(Min).          7 Note
```

Alert level G

```
PLAT068_ALERT_1_G Reported F000 Differs from Calcd (or Missing)...     Please Check
PLAT143_ALERT_4_G s.u. on c - Axis Small or Missing .............    0.00010 Ang.
PLAT164_ALERT_4_G Nr. of Refined C-H H-Atoms in Heavy-Atom Struct.         27 Note
PLAT232_ALERT_2_G Hirshfeld Test Diff (M-X)  Fe1      --C2       .        7.3 s.u.
PLAT232_ALERT_2_G Hirshfeld Test Diff (M-X)  Fe1      --C3       .        7.7 s.u.
PLAT232_ALERT_2_G Hirshfeld Test Diff (M-X)  Fe1      --C4       .        7.3 s.u.
PLAT232_ALERT_2_G Hirshfeld Test Diff (M-X)  Fe1      --C5       .        5.3 s.u.
PLAT232_ALERT_2_G Hirshfeld Test Diff (M-X)  Fe1      --C6       .        6.5 s.u.
PLAT232_ALERT_2_G Hirshfeld Test Diff (M-X)  Fe1      --C7       .        5.5 s.u.
PLAT232_ALERT_2_G Hirshfeld Test Diff (M-X)  Fe1      --C9       .        7.0 s.u.
PLAT232_ALERT_2_G Hirshfeld Test Diff (M-X)  Fe1      --C10      .        6.3 s.u.
PLAT793_ALERT_4_G Model has Chirality at C12         (Centro SPGR)          R Verify
PLAT793_ALERT_4_G Model has Chirality at C13         (Centro SPGR)          S Verify
PLAT794_ALERT_5_G Tentative Bond Valency for Fe1       (II)      .       2.09 Info
PLAT802_ALERT_4_G CIF Input Record(s) with more than 80 Characters          1 Info
PLAT912_ALERT_4_G Missing # of FCF Reflections Above STh/L=  0.600        296 Note
PLAT978_ALERT_2_G Number C-C Bonds with Positive Residual Density.         10 Info
PLAT979_ALERT_1_G NoSpherA2 Scattering Factors Used .............     Please Note
```

0 **ALERT level A** = Most likely a serious problem - resolve or explain
0 **ALERT level B** = A potentially serious problem, consider carefully
4 **ALERT level C** = Check. Ensure it is not caused by an omission or oversight
18 **ALERT level G** = General information/check it is not something unexpected

2 ALERT type 1 CIF construction/syntax error, inconsistent or missing data
10 ALERT type 2 Indicator that the structure model may be wrong or deficient
3 ALERT type 3 Indicator that the structure quality may be low
6 ALERT type 4 Improvement, methodology, query or suggestion
1 ALERT type 5 Informative message, check

It is advisable to attempt to resolve as many as possible of the alerts in all categories. Often the minor alerts point to easily fixed oversights, errors and omissions in your CIF or refinement strategy, so attention to these fine details can be worthwhile. In order to resolve some of the more serious problems it may be necessary to carry out additional measurements or structure refinements. However, the purpose of your study may justify the reported deviations and the more serious of these should normally be commented upon in the discussion or experimental section of a paper or in the "special_details" fields of the CIF. checkCIF was carefully designed to identify outliers and unusual parameters, but every test has its limitations and alerts that are not important in a particular case may appear. Conversely, the absence of alerts does not guarantee there are no aspects of the results needing attention. It is up to the individual to critically assess their own results and, if necessary, seek expert advice.

Publication of your CIF in IUCr journals

A basic structural check has been run on your CIF. These basic checks will be run on all CIFs submitted for publication in IUCr journals (*Acta Crystallographica*, *Journal of Applied Crystallography*, *Journal of Synchrotron Radiation*); however, if you intend to submit to *Acta Crystallographica Section C* or *E* or *IUCrData*, you should make sure that full publication checks are run on the final version of your CIF prior to submission.

Publication of your CIF in other journals

Please refer to the *Notes for Authors* of the relevant journal for any special instructions relating to CIF submission.

PLATON version of 20/01/2022; check.def file version of 19/01/2022

Datablock SE22004 - ellipsoid plot

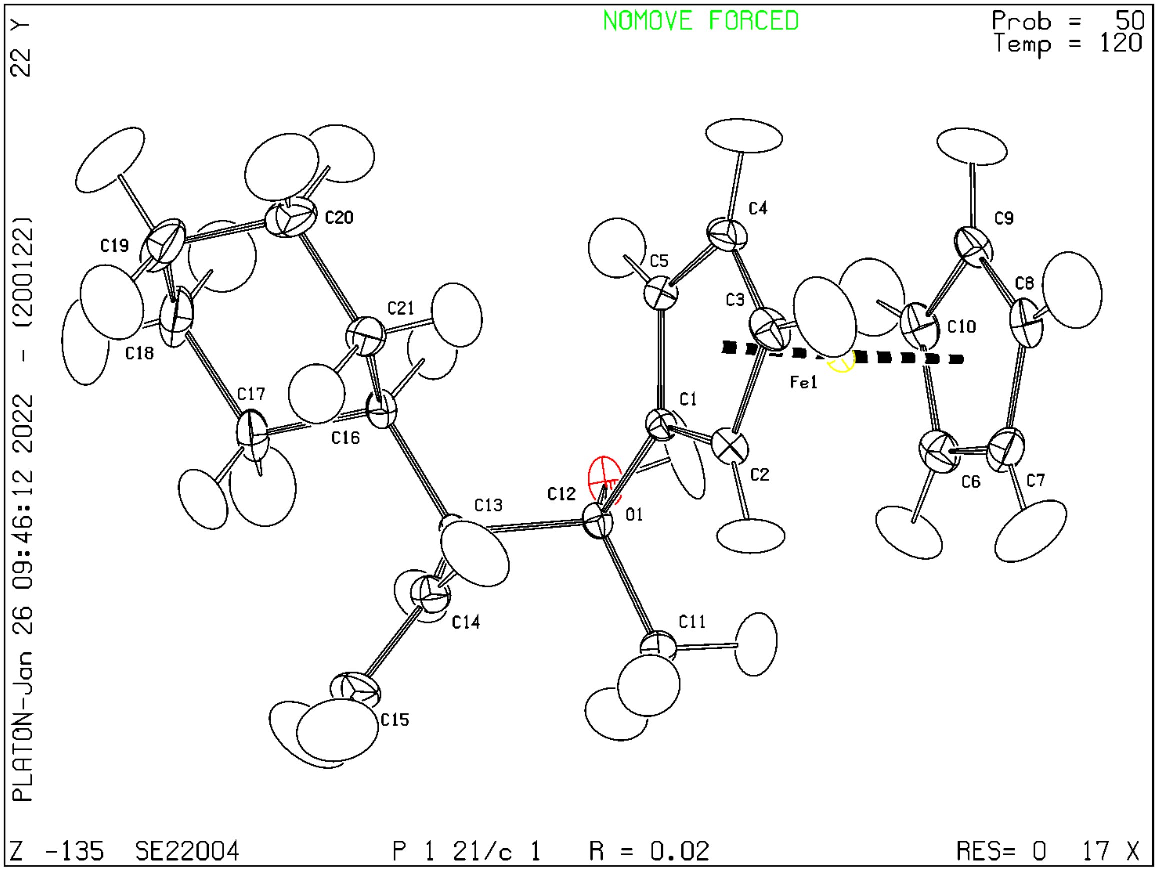

Supplement: Supplementary file 3 — cs2c03158_si_003.zip [file cs2c03158_si_003.zip › SE22004_cifreport.pdf]
